# HNRNPH1 regulates the neuroprotective cold‐shock protein RBM3 expression through poison exon exclusion

**DOI:** 10.15252/embj.2022113168

**Published:** 2023-05-30

**Authors:** Julie Qiaojin Lin, Deepak Khuperkar, Sofia Pavlou, Stanislaw Makarchuk, Nikolaos Patikas, Flora CY Lee, Julia M Zbiegly, Jianning Kang, Sarah F Field, David MD Bailey, Joshua L Freeman, Jernej Ule, Emmanouil Metzakopian, Marc‐David Ruepp, Giovanna R Mallucci

**Affiliations:** ^1^ UK Dementia Research Institute and Department of Clinical Neurosciences University of Cambridge Cambridge Biomedical Campus Cambridge UK; ^2^ UK Dementia Research Institute King's College London London UK; ^3^ Open Targets Cambridgeshire UK; ^4^ The Francis Crick Institute London UK; ^5^ Tsinghua‐Peking Joint Center for Life Sciences, School of Medicine and School of Life Sciences Tsinghua University Beijing China; ^6^ Altos Labs Cambridge Institute of Science Cambridge UK

**Keywords:** alternative splicing, cold‐shock protein, CRISPR screen, poison exon, RBM3, Neuroscience, RNA Biology

## Abstract

Enhanced expression of the cold‐shock protein RNA binding motif 3 (RBM3) is highly neuroprotective both *in vitro* and *in vivo*. Whilst upstream signalling pathways leading to RBM3 expression have been described, the precise molecular mechanism of RBM3 cold induction remains elusive. To identify temperature‐dependent modulators of RBM3, we performed a genome‐wide CRISPR‐Cas9 knockout screen using RBM3‐reporter human iPSC‐derived neurons. We found that RBM3 mRNA and protein levels are robustly regulated by several splicing factors, with heterogeneous nuclear ribonucleoprotein H1 (HNRNPH1) being the strongest positive regulator. Splicing analysis revealed that moderate hypothermia significantly represses the inclusion of a poison exon, which, when retained, targets the mRNA for nonsense‐mediated decay. Importantly, we show that HNRNPH1 mediates this cold‐dependent exon skipping via its thermosensitive interaction with a G‐rich motif within the poison exon. Our study provides novel mechanistic insights into the regulation of RBM3 and provides further targets for neuroprotective therapeutic strategies.

## Graphical abstract

The splicing factor HNRNPH1 interacts with G‐rich motifs in RBM3 poison exon in a temperature‐dependent manner to regulate poison exon inclusion and thereby RBM3 expression.

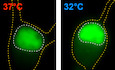

## Introduction

Lowering brain temperature during therapeutic hypothermia is robustly neuroprotective in clinical practice for several patient groups, including neonatal hypoxic–ischemic encephalopathy (Jacobs *et al*, 2011) and cardiac arrest (Arrich & European Resuscitation Council Hypothermia After Cardiac Arrest Registry Study Group, 2007). In preclinical studies, early cooling has been shown to restore memory, prevent synapse and neuronal loss and extend the survival of animal models of prion and Alzheimer's diseases (Peretti *et al*, 2015). This cold‐mediated neuroprotection is orchestrated by the elevated expression of a cold‐shock protein, RNA‐binding motif 3 (RBM3) (Peretti *et al*, 2015, 2021; Bastide *et al*, 2017). In addition, RBM3 stimulates neurogenesis in the rodent brain after hypoxic–ischemic brain injury and improves outcomes *in vivo (*Zhu *et al*, 2019 *)*, and protects neuronal cell lines from apoptosis during hypothermia *in vitro* (Chip *et al*, 2011). Clinically, high blood RBM3 levels are associated with good stroke prognosis independent of body temperature (Ávila‐Gómez *et al*, 2020). These findings have highlighted RBM3 as an attractive therapeutic target for neuroprotection—with the aim to induce its expression without cooling. However, in this respect, a mechanistic understanding of its regulatory network is required.

In addition to hypothermia, RBM3 expression has been found to be modulated by hypoxia (Wellmann *et al*, 2004; Yan *et al*, 2019), serum starvation (Wellmann *et al*, 2010), metformin (Laustriat *et al*, 2015) and morphine exposure (Koo *et al*, 2012; Lefevre *et al*, 2020). Hypothermia induces RBM3 protein expression in association with TrkB activation *in vivo* (Peretti *et al*, 2021), but the exact molecular mechanism controlling RBM3 expression downstream of these signalling cascades and other modulators remains unclear. Interestingly, hypothermia, metformin and hypoxia all alter genome‐wide alternative splicing (Laustriat *et al*, 2015; Neumann *et al*, 2020; Natua *et al*, 2021) and differential splicing leading to altered mRNA and protein expression, which is common among many RNA‐binding proteins (Lareau *et al*, 2007; Sun *et al*, 2017; Müller‐McNicoll *et al*, 2019). In particular, hypothermia‐induced alternative splicing is observed in cold‐inducible RNA‐binding protein (CIRBP) transcripts (Gotic *et al*, 2016). These findings raise an interesting possibility that RBM3 gene expression could be fine‐tuned on cooling in part by alternative splicing of its mRNA transcripts.

In this study, we uncovered the molecular mechanism involved in the cold induction of RBM3. An unbiased CRISPR/Cas9 whole‐genome knockout screen in human‐induced pluripotent stem cell (iPSC)‐derived neurons (i‐neurons) identified several splicing factors, including heterogeneous nuclear ribonucleoprotein H1 (HNRNPH1), as trans‐acting regulators of neuronal RBM3. We showed that HNRNPH1 mediates temperature‐dependent RBM3 mRNA alternative splicing in multiple cell types and maintains RBM3 transcript and protein expression in cooperation with the nonsense‐mediated mRNA decay (NMD) pathway. Additionally, we located temperature‐dependent cis‐regulatory elements in the RBM3 mRNA and demonstrated that its functional interaction with HNRNPH1 is a key determinant of RBM3 differential splicing. These findings increase the range of therapeutic targets for RBM3 induction.

## Results

### Pooled CRISPR knockout screen identifies RNA splicing as a key regulatory pathway for RBM3

**Figure 1:**
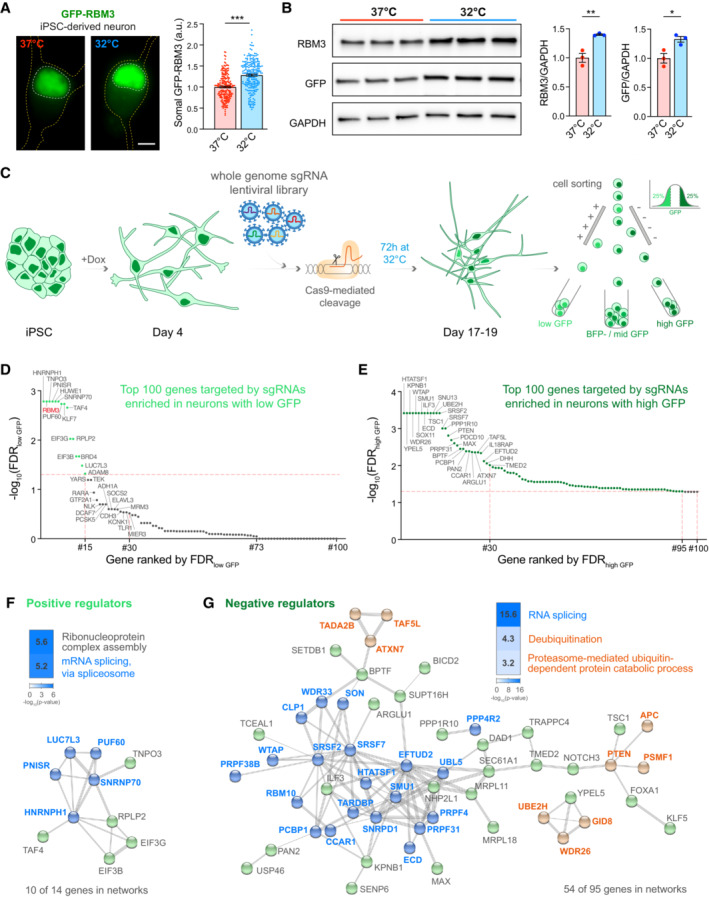
RBM3 CRISPR knockout screen identifies splicing factors as key RBM3 regulators. See also Fig EV1 ARepresentative images and quantification of somal intensity per unit area of GFP‐RBM3 i‐neurons at 37°C or after 72 h cooling at 32°C. Nuclei and cells are outlined by white and yellow dashed lines, respectively. *N* = 207 (37°C) and 220 (32°C) cells. Scale bar: 5 μm.BWestern blots and quantification of RBM3 and GFP normalised to GAPDH in GFP‐RBM3 i‐neurons at 37 or 32°C (72 h).CSchematic of experimental steps in RBM3 CRISPR screen in i‐neurons. GFP‐RBM3 iPSCs stably expressing Cas9 after 4 days of Dox‐induced differentiation are transduced with a whole‐genome lentiviral sgRNA library expressing a BFP reporter. 10–12 days after transduction, the i‐neuron cultures are incubated at 32°C for 72 h, followed by FACS to sort BFP‐positive i‐neurons with the highest and lowest 25% GFP fluorescence intensity into separate pools. *N* = 2 GFP‐RBM3 clones and 3 biological replicates.D, ETop 100 RBM3 positive regulator candidates with their sgRNAs enriched in the low‐GFP i‐neuron pool (D). Top 100 RBM3 negative regulator candidates with their sgRNAs enriched in the high‐GFP i‐neuron pool (E). Genes ranked by statistical significance (FDR). Horizontal dashed line: FDR = 0.05.FThe top‐ranked Gene Ontology terms and STRING networks of 14 positive regulator candidates (FDR <0.05, RBM3 is excluded). Genes related to RNA splicing are indicated in blue.GThe top‐ranked Gene Ontology terms and STRING networks of 95 positive regulator candidates (FDR <0.05). Genes related to RNA splicing are coloured in blue. Genes involved in deubiquitination or proteasome‐mediated ubiquitin‐dependent protein catabolic processes are coloured in orange. Data information: *N* = 3 biological replicates. Mean ± SEM; *(*P* < 0.05), **(*P* < 0.01), ***(*P* < 0.001); unpaired *t*‐tests in (A) and (B). Source data are available online for this figure.

**Figure EV1:**
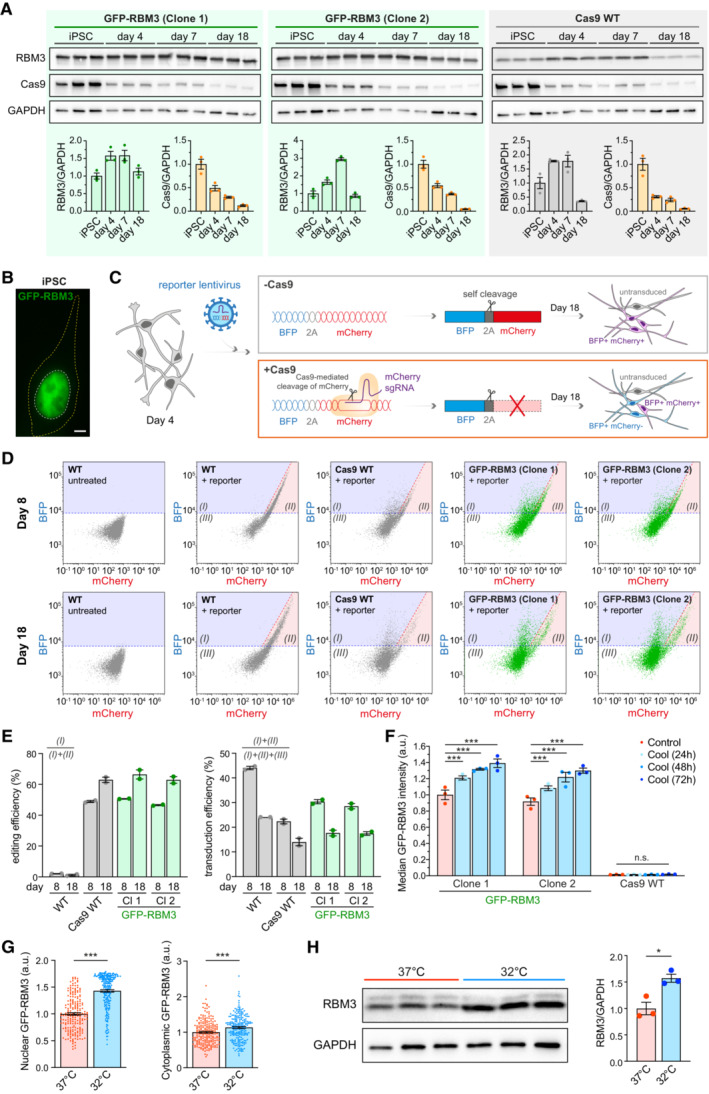
Characterisation of GFP‐RBM3 human iPSC reporter line for CRISPR knockout screen. Related to Fig 1 Western blots and quantification of RBM3, Cas9 and GAPDH in two GFP‐RBM3 clones and Cas9 WT iPSCs and i‐neurons 4, 7 and 18 days after dox‐induced differentiation.Representative image of GFP‐RBM3 iPSCs. The nucleus and soma are outlined by white and yellow dashed lines, respectively. Scale bar: 5 μm.Schematic of the reporter lentivirus design and expected fluorescent protein expression in transduced WT (‐Cas9) and Cas9 WT (+Cas9) i‐neurons. Transduced WT i‐neurons (top grey box) show high levels of BFP and mCherry. Transduced Cas9 WT i‐neurons (bottom orange box) that are successfully edited by the mCherry sgRNA express reduced levels of mCherry compared to the unedited ones.Representative BFP vs. mCherry plots measured by flow cytometry for measuring editing and transduction efficiency in WT, Cas9 WT, two clones of GFP‐RBM3 i‐neurons 4 days (Day 8) and 14 days (Day 14) after reporter lentivirus transduction. Region (I), (II) and (III) denote BFP+/mCherry‐, BFP+/mCherry+ and BFP‐/mCherry‐ populations, respectively.Editing and transduction efficiency of WT, Cas9 WT, two clones of GFP‐RBM3 i‐neurons at day 8 and 18 post differentiation. The calculation is based on the cell numbers within each area labelled in (D) and the formulas are shown in the graph.Median GFP intensity of two GFP‐RBM3 clones and Cas9 WT i‐neurons at 37°C or at 32°C for 24‐72 h, measured by flow cytometry.Nuclear and cytoplasmic GFP intensity per unit area in GFP‐RBM3 i‐neurons at 37 or 32°C (72 h). Each data point represents one cell.Western blots and quantification of RBM3 normalised to GAPDH in Cas9 WT i‐neurons at 37 or 32°C (72 h). Data information: *N* = 3 biological replicates, except (E), which has *N* = 2. Mean ± SEM; n.s. (not significant), *(*P* < 0.05), ***(*P* < 0.001); one‐way ANOVA with multiple comparisons in (F), unpaired *t*‐tests in (G) and (H). Source data are available online for this figure.

For the fluorescence‐activated cell sorting (FACS)‐based whole‐genome CRISPR/Cas9 knockout screen, a fluorescence RBM3 reporter iPSC line was generated by Cas9‐mediated homology‐directed repair to insert GFP at the N‐terminus of the single copy of RBM3 on chromosome X in wild‐type (WT) iPSCs, which contain a doxycycline (dox)‐inducible neurogenin 2 expression cassette (Pawlowski *et al*, 2017) and express Cas9 driven by the GAPDH promoter (Cas9 WT) (Pavlou *et al*, 2023) (Fig EV1A). The iPSCs can be differentiated into excitatory cortical neurons after continuous dox treatment for over 4 days (Pawlowski *et al*, 2017). GFP‐RBM3 predominantly localised to the nucleus in iPSCs (Fig EV1B) and i‐neurons (Fig 1A), consistent with previous reports in human cortical neurons (Rzechorzek *et al*, 2015). In this study, all Cas9‐mediated gene editing in i‐neurons was conducted by lentiviral transduction 4 days of postdifferentiation, when Cas9 expression is optimal (Fig EV1A). Editing efficiencies were measured by transducing reporter lentivirus expressing BFP, mCherry and mCherry‐targeted single‐guide RNA (sgRNA) 4 days after differentiation (Fig EV1C). BFP‐positive, Cas9‐expressing cells with a reduced mCherry expression, compared with mCherry intensities of BFP‐positive WT (Cas9‐negative) cells, indicated successful mCherry gene editing (Fig EV1D). 50% editing efficiencies were observed for both GFP‐RBM3 clones 4 days after transduction (Day 8) and over 60% on Day 18, similar to Cas9 WT cells (Fig EV1E).

GFP fluorescence intensities in both clones of GFP‐RBM3 i‐neurons were significantly enhanced by cooling at 32°C for 72 h (Figs 1A and EV1F) as a result of increased nuclear and cytosolic GFP‐RBM3 levels (Fig EV1G). 30–40% cold‐induction of GFP‐RBM3 protein levels was also observed by Western blots (Fig 1B), comparable to the 50% increase in endogenous RBM3 in cooled Cas9 WT i‐neurons (Fig EV1H). These observations validated that endogenously GFP‐tagged RBM3 is temperature‐responsive, consistent with behaviours of unmodified RBM3 both seen with the Cas9 WT cells in this study and reported RBM3 hypothermic induction (Jackson *et al*, 2015; Peretti *et al*, 2015).

Using both clones of this characterised GFP‐RBM3 reporter line, we performed an RBM3 CRISPR knockout pooled screen (Fig 1C) by transducing 4‐day differentiated cells with a custom‐made whole‐genome sgRNA lentiviral library, targeting critical exons of 18,466 genes across the human genome, co‐expressing a BFP reporter. Day 18 i‐neurons were incubated at 32°C for 72 h before dissociation and FACS. BFP‐positive cells with the highest and lowest GFP‐RBM3 expression, which fell in the top and bottom quartile of GFP intensity profiles, were separately collected, denoted as high GFP and low GFP populations. Genomic DNA was sequenced to identify sgRNAs enriched in high or low GFP populations (see Appendix Table S2 for all significant genes). Ranked by the false discovery rate (FDR), 15 genes, including RBM3, were enriched in low GFP i‐neurons, suggesting they likely positively regulate RBM3 expression (Fig 1D). In contrast, 95 genes, likely to negatively affect RBM3 expression, were enriched in high GFP populations (Fig 1E).

Gene ontology (GO) and network analysis revealed that a major group of RBM3 regulator candidates are involved in RNA splicing (Fig 1F and G), including spliceosomal proteins, for example U1 small nuclear ribonucleoprotein 70 kDa (SNRNP70), heterogeneous nuclear ribonucleoproteins (hnRNPs) and serine and arginine rich splicing factors (SRSFs). Other potential RBM3 regulators play roles in nucleocytoplasmic transport, for example transportin 3 (TNPO3) and karyopherin‐β1 (KPBN1); translation, for example 60S acidic ribosomal protein P2 (RPLP2) and eukaryotic translation initiation factor 3 subunit B (EIF3B); transcription, for example transcription initiation factor TFIID subunit 4 (TAF4); ubiquitination, for example ubiquitin‐conjugating enzyme E2 H (UBE2H) (see Appendix Table S3 for a full list of GO terms).

### Arrayed target validation reveals regulators modulating neuronal RBM3 protein and transcript abundance

**Figure 2:**
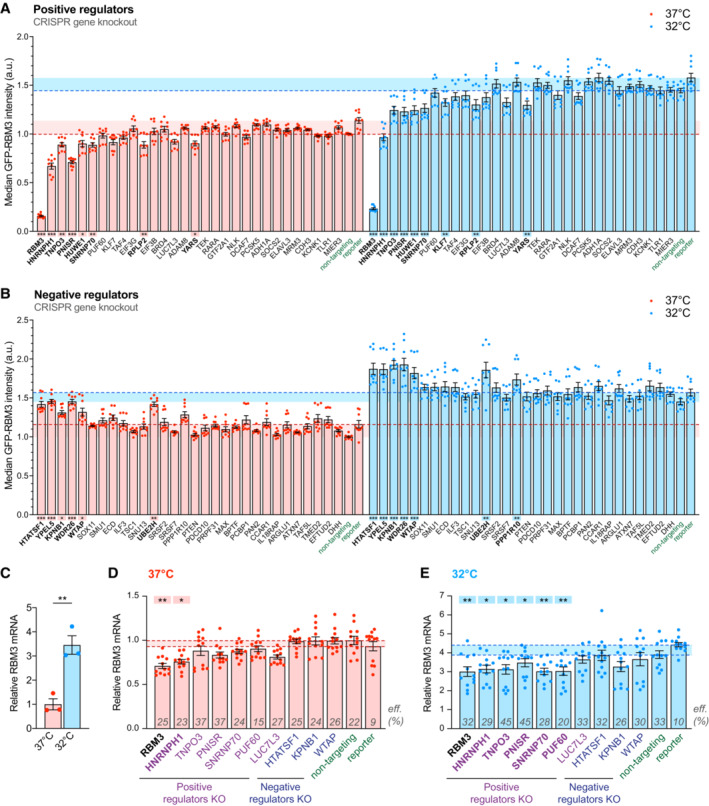
Depleting RBM3 positive regulators that function in RNA splicing reduces RBM3 protein and mRNA levels. See also Fig EV2 A, BMedian GFP intensity of BFP‐positive GFP‐RBM3 i‐neurons measured by flow cytometry upon the sgRNA/Cas9‐mediated KO of top 30 positive (A) or negative (B) regulator candidates. Statistical analysis is performed between the specific and non‐targeting sgRNA groups for positive regulators (A) or between the specific sgRNA and reporter groups for negative regulators (B) within the 37 or 32°C (72 h) population.CqRT‐PCR of RBM3 mRNA level normalised to 18 s rRNA in i‐neurons at 37 or 32°C (72 h).D, EqRT‐PCR of RBM3 mRNA level normalised to 18 s rRNA in GFP‐RBM3 i‐neurons at 37°C (D) or 32°C for 72 h (E) transduced with lentivirus containing specific, non‐targeting sgRNA or the reporter. Statistical analysis is performed between the specific and non‐targeting sgRNA for negative regulators in (D) and positive regulators in (E), or between the specific and reporter groups for positive regulators in (D) and negative regulators in (E). Transduction efficiencies are indicated in corresponding bars. Data information: *N* = 3 biological replicates. Each data point represents one well of culture. Mean ± SEM; *(*P* < 0.05), **(*P* < 0.01), ***(*P* < 0.001); One‐way ANOVA with multiple comparisons in (A), (B), (D), (E), unpaired *t*‐tests in (C). Source data are available online for this figure.

**Figure EV2:**
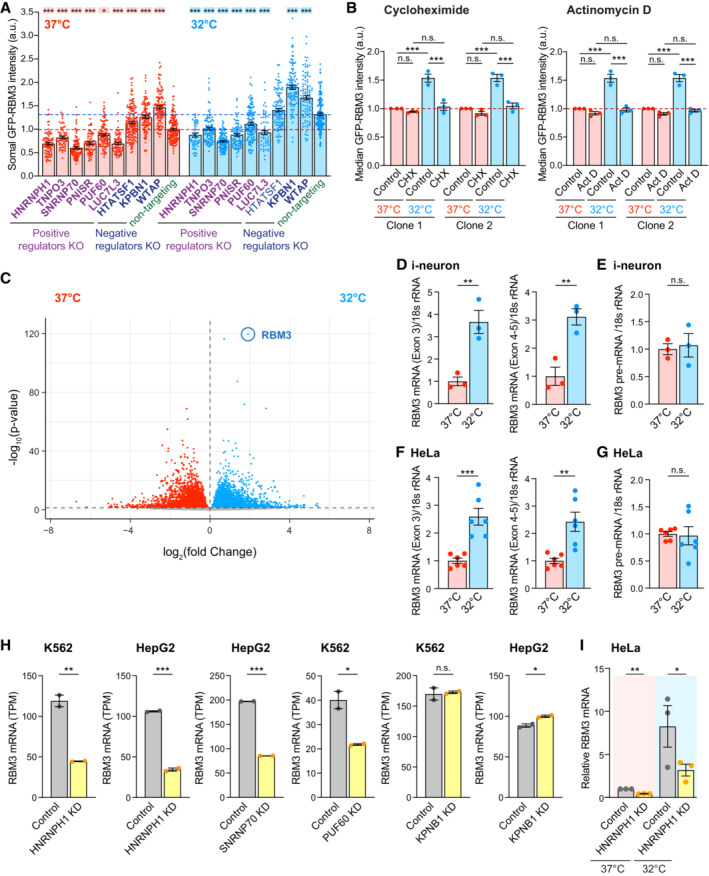
Cooling and regulators involved in mRNA splicing change RBM3 transcript levels. Related to Fig 2 ASomal GFP intensity per unit area in GFP‐RBM3 i‐neurons transduced with lentivirus containing specific or non‐targeting sgRNA at 37 or 32°C (72 h) imaged by wide‐field microscopy. Only BFP‐positive (transduced) cells are included. Statistical analysis is performed between the specific and non‐targeting sgRNA within the temperature groups. Each data point represents one cell.BMedian GFP intensity per unit area of GFP‐RBM3 i‐neurons at 37 or 32°C (72 h) treated with cycloheximide (CHX) at 50 μM for 72 h or actinomycin D (Act D) at 1 μM for 72 h.CVolcano plot showing differential expression analysis of all transcripts identified in i‐neurons at 37 and 32°C (72 h) from RNA‐Seq data.D, EqRT‐PCR of RBM3 Exon 3, Exon 4–5 (D) and pre‐mRNA (E) normalised to 18 s rRNA in i‐neurons at 37 and 32°C (72 h).F, GqRT‐PCR of RBM3 Exon 3, Exon 4–5 (F) and pre‐mRNA (G) normalised to 18 s rRNA in HeLa cells at 37 and 32°C (48 h).HNormalised RBM3 mRNA abundance (TPM) of control and selective regulator candidates knocked‐down K562 or HepG2 cells. Data are extracted from ENCODE project. 2 isogenic replicates are included in each condition.IqRT‐PCR of RBM3 mRNA normalised to GAPDH in control and HNRNPH1 KD HeLa cells at 37 or 32°C (48 h). Data information: *N* = 3 biological replicates. Mean ± SEM; n.s. (not significant), *(*P* < 0.05), **(*P* < 0.01), ***(*P* < 0.001); one‐way ANOVA with multiple comparisons in (A), unpaired *t*‐tests in (B), (D)‐(H); paired *t*‐test in (I). Source data are available online for this figure.

To validate the top hits identified by the pooled screen, we individually depleted the top 30 RBM3‐positive and ‐negative regulator candidates using 1–3 sgRNAs per gene from the whole‐genome library (see Appendix Table S4). I‐neurons transduced with three nontargeting sgRNAs from the pooled library or the reporter lentivirus for editing efficiency calculation served as controls. As a positive control, RBM3 sgRNAs reduced GFP fluorescence by over 80% compared with nontargeting sgRNAs at 37 or 32°C (Fig 2A). Moderate spectral crossover and activation of specific signalling pathways due to target‐specific genome editing may account for the discrepancy between nontargeting sgRNA and reporter controls. To apply a more stringent standard, we performed statistical analysis between specific gene knockout (KO) groups and one of the two control groups with high P‐values, for instance, comparing to the nontargeting sgRNA control showing lower GFP intensity to identify positive regulators, and to the reporter control for negative regulators. Knocking out seven out of 29 positive regulator candidates tested significantly reduced GFP‐RBM3 levels at 37 and 32°C (Fig 2A). HNRNPH1 (Chou *et al*, 1999), TNPO3 (Kataoka *et al*, 1999), PNN Interacting Serine And Arginine Rich Protein (PNISR) (Zimowska *et al*, 2003) and SNRNP70 (Pomeranz Krummel *et al*, 2009) are associated with RNA splicing. Among 30 tested RBM3‐negative regulator candidates, KO of six genes significantly increased GFP‐RBM3 expression in i‐neurons at 37 and 32°C (Fig 2B). Specifically, HIV‐1 Tat‐Specific Factor 1 (HTATSF1) and WT1‐Associated Protein (WTAP) are splicing regulators, while Yippee Like 5 (YEPL5), WD Repeat Domain 26 (WDR26) and UBE2H mediate ubiquitination and proteasomal degradation, the depletion of which is more likely to result in generic instead of RBM3‐specific protein accumulation. Changes in GFP‐RBM3 levels upon selective RBM3 regulator KO were also confirmed using fluorescence microscopy (Fig EV2A). Collectively, the arrayed CRISPR knockout assay validated hits associated with the lowest FDR in the pooled screen (Fig 1D and E), and reiterated that multiple components of the splicing machinery are key to RBM3 protein expression.

To investigate whether the cold‐induced GFP‐RBM3 protein expression is controlled at the translational or transcriptional level, the translation inhibitor cycloheximide or the RNA polymerase inhibitor actinomycin D was applied to i‐neurons at 37 or 32°C. The elevation of GFP‐RBM3 in cooled cells was completely abolished by either inhibitor (Fig EV2B), indicating the cold‐increased GFP‐RBM3 expression relies on the *de novo* transcription and translation of RBM3 transcripts. In line with this finding, differential expression analysis comparing RNA‐Seq data between i‐neurons at 37 and 32°C showed RBM3 increased fourfold on cooling: this was the most significant increase across the entire transcriptome (Fig EV2C). Furthermore, real‐time PCR (quantitative PCR, qPCR) of RBM3 mRNA supports cold‐induction of RBM3 mRNA by 3–5 folds in Cas9 WT i‐neurons (Figs 2C and EV2D) and HeLa cells (Fig EV2F), without apparent transcriptional activation as RBM3 pre‐mRNA levels remained unchanged upon cooling (Fig EV2E and G).

We next explored whether the changes in RBM3 protein expression upon individual regulator KO were due to altered RBM3 mRNA levels. We focussed on the validated splicing regulating genes (HNRNPH1, PNISR, SNRNP70, HTATSF1, WTAP), potential regulators marginally affecting RBM3 expression (Poly(U) Binding Splicing Factor 60 (PUF60) and LUC7 Like 3 Pre‐mRNA Splicing Factor (LUC7L3)) and the two nuclear protein importers (TNPO3 and KPNB1) (Fig 2D and E). Due to cell death and promoter silencing, only 15–45% of the transduced cells remained BFP‐positive, as approximates to transduction efficiencies (labelled on bars, Fig 2D and E), on Day 18 when RNA was extracted from i‐neuron cultures at 37 or 32°C. Remarkably, the extent of RBM3 transcript reduction due to HNRNPH1 KO at 37 or 32°C was similar to RBM3 KO as a positive control (Fig 2D and E), revealing HNRNPH1 as a strong positive regulator of RBM3 mRNA expression. TNPO3, PNISR, SNRNP70 and PUF60 KO also significantly lowered RBM3 transcript levels at 32°C (Fig 2E), suggesting they are key regulators for cold induction of RBM3 transcripts. On the contrary, none of the tested negative regulator KO affected RBM3 mRNA expression, which implies that their regulation may be at the translational or post‐translational level. In support of our findings in i‐neurons, RNA‐Seq analysis of public data also showed reduced RBM3 mRNA levels upon HNRNPH1, SNRNP70 and PUF60 KD in K562 or HepG2 cells, while no difference was seen upon KPNB1 KD (Fig EV2H; Appendix Table S5) (ENCODE Project Consortium, 2012; Luo *et al*, 2020). In HNRNPH1‐knocked down HeLa cells, RBM3 is among the top 5% downregulated genes detected by RNA‐Seq and proteomics, with its mRNA and protein levels decreased by 3 and 0.55 folds, respectively (Uren *et al*, 2016), demonstrating a strong regulatory effect of HNRNPH1 on RBM3 transcripts and proteins. We further showed that HNRNPH1 KD significantly decreased RBM3 mRNA in HeLa cells by over 50% both at 37 and 32°C (Fig EV2I). To summarise, depleting specific splicing factors, particularly HNRNPH1, abolishes the cold‐induced increase of RBM3 mRNA expression in i‐neurons and other cell types.

### RBM3 poison exon that triggers nonsense‐mediated decay is silenced during hypothermia

**Figure 3:**
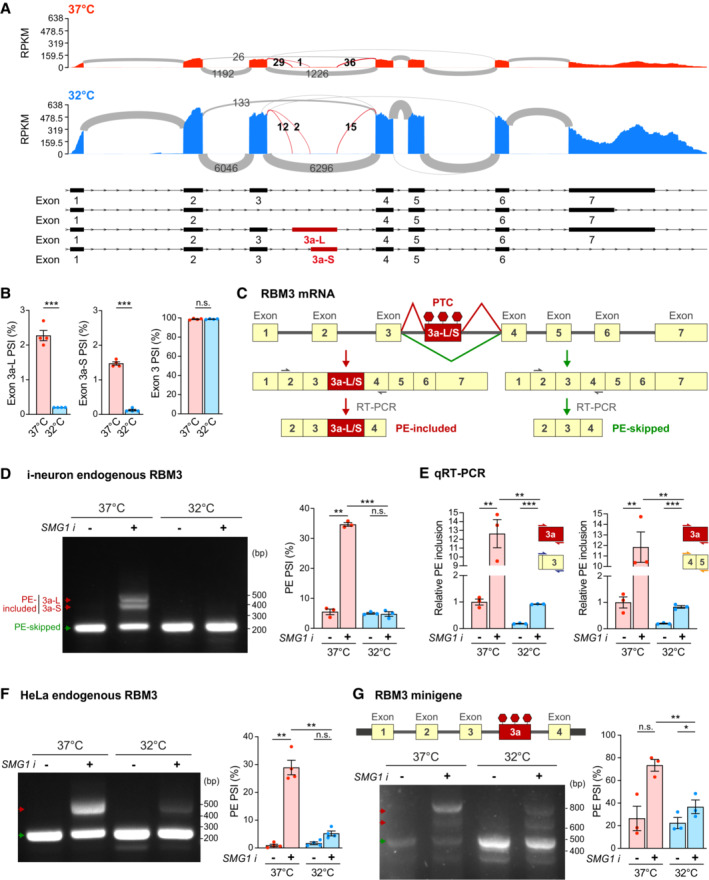
Hypothermia represses RBM3 mRNA poison exon inclusion. See also Fig EV3 Sashimi plots of RBM3 transcripts in WT i‐neurons at 37 and 32°C (72 h), showing major alternatively spliced isoforms. Differentially spliced Exon 3a‐L and 3a‐S junctions between 37 and 32°C conditions are shown in red. *N* = 4.PSI values of RBM3 Exon 3a‐L and 3a‐S relative to Exon 3 and 4, and Exon 3 relative to Exon 2 and 4 in i‐neurons at 37 or 32°C (72 h).Schematics of RBM3 Exon 3a, or poison exon (PE), alternative splicing and the resulting PE‐included (left) or PE‐skipped (right) mRNA products. RT‐PCR primer pair amplifying Exon 2–4 are indicated by grey arrows.RT‐PCR of RBM3 mRNA (Exon 2–4) in i‐neurons at 37 or 32°C (72 h) in the presence or absence of SMG1 inhibitor. PSI values of RBM3 PE are calculated based on the intensity of PE‐included (red arrows) and PE‐skipped (green arrow) isoforms.qRT‐PCR using a combination of primers targeting Exon 3a, Exon 3 or Exon 4–5 quantifies the PSI values of RBM3 PE (Exon 3a, including both 3a‐L and 3a‐S) relative to Exon 3 or Exon 4–5 at 37 or 32°C (72 h) in the presence or absence of SMG1 inhibitor.RT‐PCR of RBM3 mRNA (Exon 2–4) in HeLa cells at 37 and 32°C (48 h) in the presence or absence of SMG1 inhibitor. PSI values of RBM3 PE are depicted in the graph on the right.Schematics and RT‐PCR of RBM3 minigene (Exon 1–4), flanked by unique sequences (thick black bars) to distinguish it from endogenous transcripts during PCR amplification, expressed in HeLa cells at 37 or 32°C (48 h), in the presence or absence of SMG1 inhibitor. PSI values of RBM3 PE are depicted in the graph on the right. Data information: *N* = 4 biological replicates in (A), (B) and (F), *N* = 3 in (D), (E) and (G). Mean ± SEM; n.s. (not significant), *(*P* < 0.05), **(*P* < 0.01), ***(*P* < 0.001); FDR calculated by rMATS program in (B); unpaired *t*‐tests in (E); paired *t*‐tests in (D), (F), (G). Source data are available online for this figure.

**Figure EV3:**
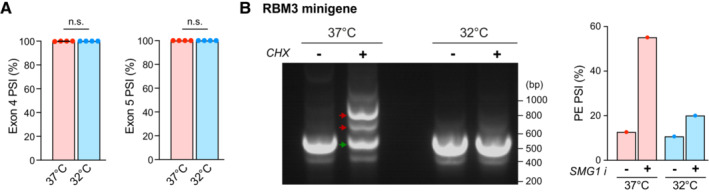
Cooling represses RBM3 poison exon inclusion. Related to Fig 3 PSI values of RBM3 Exon 4 relative to Exon 3 and 6, and Exon 5 relative to Exon 4 and 6 in i‐neurons at 37 or 32°C (72 h). *N* = 4 biological replicates.RT‐PCR of RBM3 minigene expressed in HeLa cells at 37 and 32°C (48 h) in the presence or absence of cycloheximide (CHX) at 200 μg/ml concentration. PSI values of RBM3 PE are calculated based on the intensity of PE‐included (red arrows) and PE‐skipped (green arrow) isoforms. *N* = 1 biological replicate. Data information: Mean ± SEM; n.s. (not significant); FDR calculated by rMATS program in (A). Source data are available online for this figure.

Given that RBM3 mRNA expression is tightly regulated by splicing factors in i‐neurons, we searched for RBM3 alternatively spliced isoforms with RNA‐Seq data acquired from Cas9 WT i‐neurons at 37 and 32°C (Fig 3A). Multiple isoforms of RBM3 transcripts were identified and the most abundant four isoforms could be distinguished by either Exon 3, Exon 3a‐L or Exon 3a‐S inclusion (Fig 3A). Differential splicing analysis showed RBM3 Exon 3a inclusion levels drastically reduced upon cooling, from the percent spliced in (PSI) index of 0.023 to 0.002 for Exon 3a‐L, or from 0.015 to 0.001 for Exon 3a‐S (Fig 3B; Appendix Table S6), while inclusion levels of Exon 3 and constitutive Exons 4 and 5 remained unchanged at 37 and 32°C (Figs 3B and EV3A; Appendix Table S6), demonstrating that cooling effectively repressed the production of Exon 3a‐L/S‐containing RBM3 transcripts.

A close examination of Exon 3a of RBM3 revealed multiple stop codons in‐frame with the coding sequence, potentially leading to premature translational termination (Llorian *et al*, 2016). Thus, the inclusion of Exon 3a makes the RBM3 transcript a susceptible target for degradation by nonsense‐mediated mRNA decay (NMD), an mRNA surveillance pathway that degrades mRNAs that harbour premature termination codons (PTCs) (He & Jacobson, 2015). Exon 3a could therefore serve as a poison exon (PE) in RBM3 mRNA, leading to a reduction of the transcript level if retained. Such post‐transcriptional regulation mediated by PEs is key to fine‐tuning expression levels for many proteins, especially RNA‐binding proteins (Neumann *et al*, 2020). To further verify whether Exon 3a indeed acts as a PE, and to explore the temperature‐dependent inclusion of RBM3 PE quantitatively, we designed isoform‐sensitive primers to amplify regions between RBM3 exon 3 and exon 4 to identify PE‐included or PE‐skipped isoforms of RBM3 transcripts (Fig 3C). In order to detect the NMD‐sensitive PE‐included isoforms, we blocked NMD using a chemical inhibitor of SMG1 kinase, a key member of the NMD pathway (Langer *et al*, 2021). When i‐neurons were incubated with SMG1 inhibitor for 24 h at 37°C, both PE‐included and PE‐skipped isoforms were detected with RT‐PCR. Interestingly, in 72 h‐cooled i‐neurons, only the PE‐skipped isoform was detected, even after SMG1 inhibitor treatment, clearly supporting that the RBM3 PE was preferentially excluded in cooled i‐neurons (Fig 3D). This finding was further validated by qPCR using primers against Exon 3a (detecting both 3a‐L and 3a‐S) and the constitutive exons, for example Exon 3 and Exon 4–5 (Fig 3E). The fraction of PE‐contained transcripts to the total transcripts at 37°C was over five times more compared with the fraction at 32°C. When NMD was inhibited by the SMG1 inhibitor, the differences rose to 13–15 folds (Fig 3E).

Likewise, PE of endogenous RBM3 mRNA was excluded in NMD‐inhibited HeLa cells on cooling (Fig 3F). Additionally, an RBM3 minigene spanning RBM3 Exons 1–4, which was sensitive to NMD when expressed in HeLa cells, also showed a significantly lower fraction of PE‐retained isoform at 32°C compared to 37°C, when NMD was blocked by SMG1 inhibitor (Fig 3G) or cycloheximide (Fig EV3B).

### HNRNPH1 represses RBM3 poison exon inclusion at low temperatures

**Figure 4:**
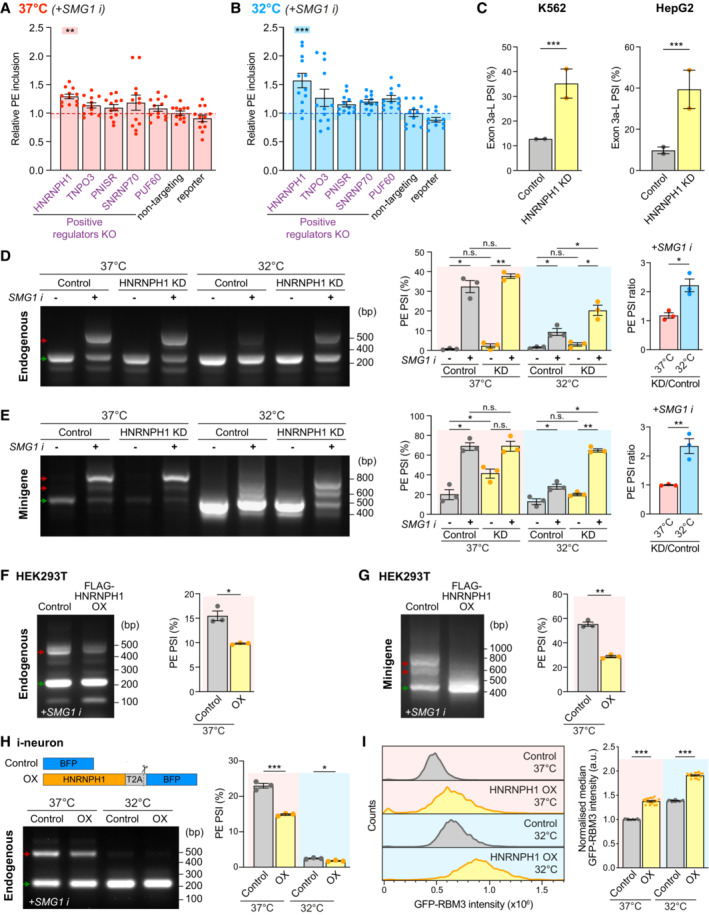
HNRNPH1 increases RBM3 expression by promoting poison exon skipping. See also Fig EV4 A, BqRT‐PCR quantifying the PSI values of RBM3 PE relative to RBM3 mRNA in WT i‐neurons at 37°C (A) or 32°C (72 h) (B), when NMD is blocked by SMG1 inhibitor. Statistical analysis is performed between the specific and non‐targeting sgRNA groups.CPSI values of RBM3 Exon 3a‐L in control and HNRNPH1‐knocked down K562 and HepG2 cells. RNA‐Seq data from ENCODE Project, 2 isogenic replicates are included.D, ERT‐PCR of endogenous RBM3 (D) and expressed RBM3 minigene (E) in control (scramble siRNA) or HNRNPH1‐knocked down HeLa cells at 37 or 32°C (48 h), in the presence or absence of SMG1 inhibitor. The ratio of PSI values between HNRNPH1 KD and control is shown for only the SMG1 i‐treated conditions.F, GRT‐PCR of endogenous RBM3 (F) and expressed RBM3 minigene (G) in SMG1 inhibitor‐treated control (untransfected) or FLAG‐HNRNPH1‐overexpressing (OX) HEK293T cells at 37°C. PSI values of RBM3 PE are shown in the graphs on the right respectively.HWT i‐neurons are transduced with lentiviral constructs expressing BFP (Control) or HNRNPH1‐T2A‐BFP (HNRNPH1 overexpression, OX) at 37 and 32°C, followed by RT‐PCR of endogenous RBM3 with 24 h SMG1 inhibitor treatment. PSI values of RBM3 PE are shown in the graph on the right.IGFP‐RBM3 intensity histogram and control (37°C)‐normalised median GFP intensity indicating GFP intensity of control and HNRNPH1‐overexpressing (OX) GFP‐RBM3 i‐neurons measured by flow cytometry. Only cells with top 5% BFP levels among all BFP‐positive cells in the well are included. Data information: *N* = 3 biological replicates. Each data point represents one well of culture in (A) and (I). Mean ± SEM; n.s. (not significant), *(*P* < 0.05), **(*P* < 0.01), ***(*P* < 0.001); one‐way ANOVA with multiple comparisons in (A), (B); FDR calculated by rMATS program in (C), paired *t*‐tests in (D–G), unpaired *t*‐tests in (H), (I). Source data are available online for this figure.

**Figure EV4:**
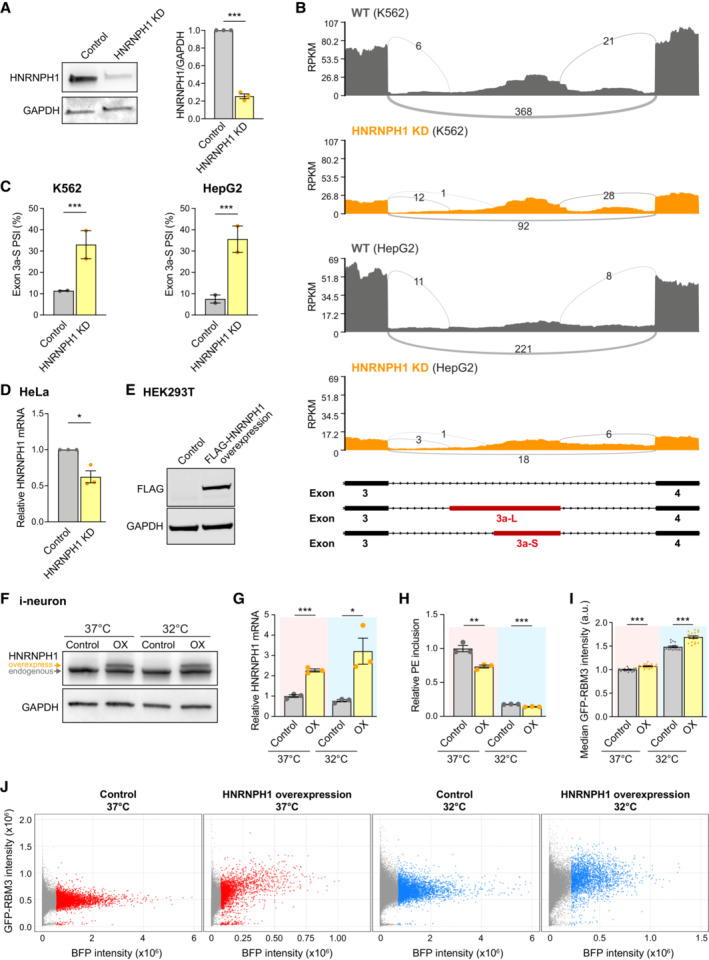
HNRNPH1 enhances RBM3 expression and RBM3 mRNA poison exon skipping on cooling. Related to Fig 4 Western blots and quantification of HNRNPH1 normalised to GAPDH in Control and HNRNPH1 KD Cas9WT i‐neurons.Sashimi plots of the region between Exon 3 and 4 of RBM3 transcripts in control and HNRNPH1 knocked‐down K562 and HepG2 cells, showing major alternatively spliced isoforms. Data are from ENCODE Project. 2 isogenic replicates are included.PSI values of RBM3 Exon 3a‐S in control and HNRNPH1‐knocked down K562 and HepG2 cells. RNA‐Seq data from ENCODE Project, 2 isogenic replicates are included.qRT‐PCR of HNRNPH1 mRNA normalised to GAPDH upon HNRNPH1 KD in HeLa cells.Western blots of FLAG‐HNRNPH1 and GAPDH (loading control) in control and FLAG‐HNRNPH1‐expressed HEK293T cells at 37°C.Western blot of WT i‐neurons transduced with lentivirus expressing BFP (control) or HNRNPH1‐T2A‐BFP (OX) at 37 and 32°C. The larger molecular weight of overexpressed HNRNPH1 is due to the additional amino acids between the C‐terminus of HNRNPH1 and the T2A cleavage site.qRT‐PCR of HNRNPH1 mRNA normalised to 18 s rRNA of WT i‐neurons transduced with lentivirus expressing BFP (control) or HNRNPH1‐T2A‐BFP (OX) at 37 and 32°C.qRT‐PCR quantifying the PSI values of RBM3 PE relative to RBM3 mRNA (mean value of RBM3 exon 3 and exon 4–5) in SMG1 inhibitor‐treated control and HNRNPH1‐overexpressing (OX) WT i‐neurons at 37 and 32°C.Median GFP intensity of control and HNRNPH1‐overexpressing (OX) GFP‐RBM3 i‐neurons measured by flow cytometry.Representative dot plots of flow cytometry data showing BFP or HNRNPH1‐T2A‐BFP expression (X‐axis) and GFP intensity of successfully transduced (BFP‐positive) GFP‐RBM3 i‐neurons. Each data point represents one cell. Cells with high levels of BFP expression (top 5% in each well) are coloured in red (37°C) or blue (32°C), and the rest of BFP‐positive cells are in grey. Y‐axes are in the same scale and X‐axes are scaled to the sample. Data information: *N* = 3 biological replicates. Mean ± SEM; n.s. (not significant), *(*P* < 0.05), **(*P* < 0.01), ***(*P* < 0.001); unpaired *t*‐tests in (A), (G), (H), (I), FDR calculated by rMATS program in (C), paired *t*‐test in (D). Source data are available online for this figure.

We next investigated whether positive RBM3 mRNA regulators control RBM3 transcript abundance through PE skipping. When NMD was inhibited, knocking down HNRNPH1 (Fig EV4A) resulted in RBM3 PE retention in Cas9 WT i‐neurons, with a 29% increase at 37°C and a 57% increase at 32°C compared with the respective nontargeting control (Fig 4A and B). RBM3 PE‐retention upon HNRNPH1 KD was also found in published RNA‐Seq analysis using K562 and HepG2 cells, demonstrating its conserved role across multiple cell types (Figs 4C and EV4B and C; Appendix Table S6) (ENCODE Project Consortium, 2012; Luo *et al*, 2020). In HeLa cells treated with SMG1 inhibitor, RNAi‐mediated HNRNPH1 knockdown (Fig EV4D) increased PE retention in endogenous RBM3 transcripts (Fig 4D) and in RBM3 minigene (Fig 4E) only at 32°C when NMD was inhibited, suggesting that the role of HNRNPH1 in repressing RBM3 PE inclusion is context‐dependent, being more important under the cooled condition. Moreover, overexpression of FLAG‐tagged HNRNPH1 in HEK293T cells (Fig EV4E) repressed PE inclusion in endogenous RBM3 mRNA (Fig 4F) and in the minigene (Fig 4G) at 37°C, and a comparable extent of PE skipping was observed in HNRNPH1‐overexpressing i‐neurons at 37 and 32°C (Figs 4H and EV4F–H), confirming the splicing‐regulatory function of HNRNPH1 in RBM3 PE exclusion. As expected, overexpression of HNRNPH1 in GFP‐RBM3 i‐neurons with HNRNPH1‐T2A‐BFP lentiviral transduction resulted in GFP‐RBM3 upregulation at 37 and 32°C, compared to the BFP control (Fig EV4I). While moderate HNRNPH1 overexpression led to limited GFP‐RBM3 increases (Fig EV4J, grey data points), i‐neurons associated with high levels of HNRNPH1‐T2A‐BFP expression (Fig EV4J, coloured data points indicate cells with top 5% BFP levels) showed significant GFP‐RBM3 elevation (Fig 4I, only cells with top 5% BFP levels are shown).

**Figure 5:**
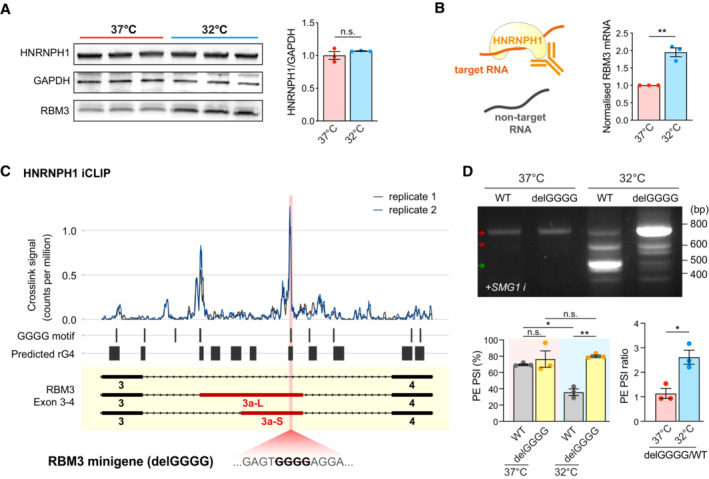
HNRNPH1 interacts with G‐rich sequences in RBM3 poison exon in a temperature‐dependent manner. See also Fig EV5 Western blot and quantification of HNRNPH1 normalised to GAPDH in WT i‐neurons at 37 and 32°C (72 h). RBM3 blots of the same samples are shown for comparison.Schematic of HNRNPH1 RNA Immunoprecipitation (RIP) in HeLa cells at 37 or 32°C. The graph on the right shows the fold change in HNRNPH1‐pulled down RBM3 mRNA after normalisation.Analysis of public HNRNPH1 iCLIP dataset in two replicates, mapped to RBM3 Exon 3–4. Crosslink counts are normalised to library size. RNA G quadruplexes (rG4) are predicted using QGRS mapper. The position of the GGGG motif deleted in the mutant RBM3 minigene is shown in pink.RT‐PCR of WT and delGGGG RBM3 minigenes in HeLa cells at 37 or 32°C (48 h) treated with SMG1 inhibitor. PSI values of RBM3 PE are shown in the graphs on the right. Data information: *N* = 3 biological replicates. Mean ± SEM; n.s. (not significant), *(*P* < 0.05), **(*P* < 0.01); unpaired *t*‐tests in (A), (B), (F), paired *t*‐tests in (D). Source data are available online for this figure.

**Figure EV5:**
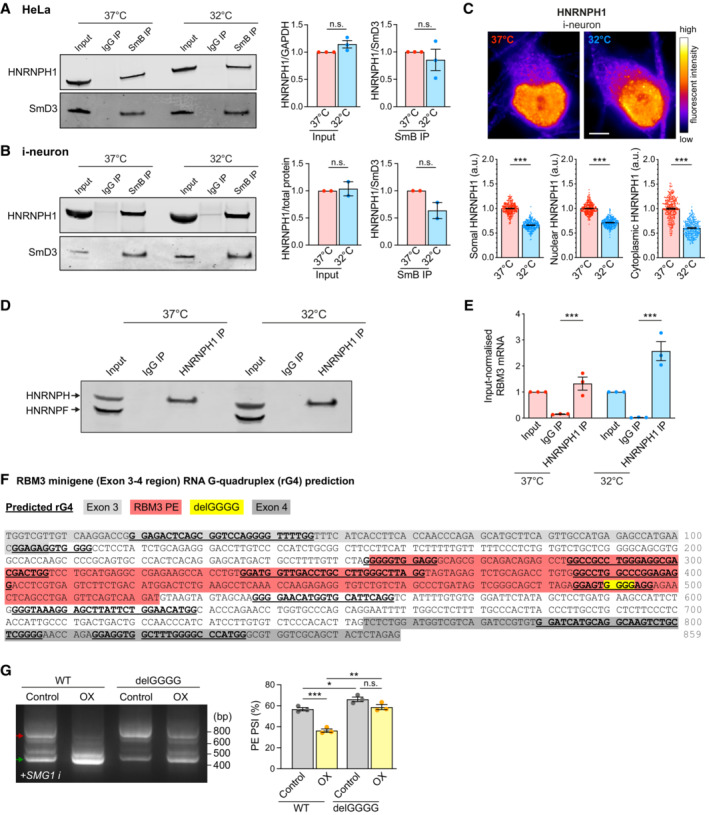
HNRNPH1 interacts with G‐rich sequences in RBM3 poison exon in a temperature‐dependent manner. Related to Fig 5 Western blot and quantification of HNRNPH1 total protein levels (input HNRNPH1 normalised to GAPDH) and its abundance in spliceosomal protein SmB pulldown (normalised to spliceosomal protein SmD3) in HeLa cells at 37 and 32°C (72 h).Western blot and quantification of HNRNPH1 total protein levels (input HNRNPH1 normalised to ponceau measured total protein abundance) and its abundance in spliceosomal protein SmB pulldown (normalised to spliceosomal protein SmD3) in i‐neurons at 37 and 32°C (72 h).Representative images of HNRNPH1 staining of GFP‐RBM3 i‐neurons at 37°C or after 72 h cooling at 32°C. Graphs below the images show quantification of somal, nuclear and cytoplasmic intensity per unit area respectively. The decrease of HNRNPH1 signals at 32°C is due to the global attenuation of protein production, which is not seen after total protein or GAPDH normalisation in western blot in (B) and Fig 4A. *N* = 280 (37°C) and 262 (32°C) cells. Scale bar: 5 μm.Western blot of HNRNPH/F in HeLa cell input (total) lysate, IgG‐pulled down and HNRNPH1‐pulled down eluates at 37 and 32°C (48 h).Quantification of input‐normalised RBM3 mRNA levels in HeLa cell input (total), IgG‐pulled down and HNRNPH1‐pulled down RNA at 37 and 32°C (48 h).RNA G quadruplexes (rG4) within the RBM3 Exon 3–4 region are predicted using QGRS mapper. Deletion of the GGGG motif in the mutant RBM3 minigene is predicted to disrupt the rG4 structure overlapping this region.RT‐PCR of WT and delGGGG RBM3 minigenes in control or HNRNPH1‐overexpressing (OX) HEK293T cells treated with SMG1 inhibitor at 37°C. PSI values of RBM3 PE are shown in the graphs on the right. Data information: *N* = 3 biological replicates, except (B), which has *N* = 2. Mean ± SEM; n.s. (not significant), *(*P* < 0.05), **(*P* < 0.01), ***(*P* < 0.001); paired *t*‐test in (A), (B), (E), (G), unpaired *t*‐tests in (C). Source data are available online for this figure.

Given the strong correlation between HNRNPH1 and RBM3 expression, we speculated that HNRNPH1 expression is also cold‐inducible. To our surprise, HNRNPH1 protein levels, its interaction with spliceosomes shown by core spliceosomal protein SmB pulldown, and its nucleo‐cytoplasmic localisation were not altered by cooling (Figs 5A and EV5A–C), suggesting that cooling‐related regulation of RBM3 mRNA by HNRNPH1 is not driven by generic changes in HNRNPH1 spliceosomal association. Next, to explore the temperature‐dependent physical association between HNRNPH1 and RBM3 mRNA, we performed HNRNPH1 RNA immunoprecipitation (RIP) in HeLa cells, which were incubated at 37 or 32°C and supplemented with SMG1 inhibitor. We found a twofold increase in the RBM3 mRNA levels in HNRNPH1 immunoprecipitates at 32°C compared to 37°C (Figs 5B and EV5D and E), indicating an increased interaction between HNRNPH1 and RBM3 mRNA on cooling.

With evidence for a cold‐enhanced HNRNPH1‐RBM3 mRNA interaction, we next searched for potential HNRNPH1 binding sites around RBM3 PE that regulate its alternative splicing. The intronic sequence between RBM3 Exon 3 and 4 contains multiple poly‐G stretches with potentials to form RNA G‐quadruplex structures (rG4s) (Kharel *et al*, 2020) (Fig 5C), which are known as HNRNPH1 consensus binding sites (Caputi & Zahler, 2001; Uren *et al*, 2016). Indeed, analysis of published HNRNPH1 Individual‐nucleotide resolution UV crosslinking and immunoprecipitation (iCLIP) data set in HEK293T cells (Braun *et al*, 2018) revealed that HNRNPH1 strongly interacts with GGGG motifs at the 5′ end of PE (near 3′ splice site) and 30 nucleotides upstream of the 5′ splice site between RBM3 Exon 3 and 4 (Fig 5C). Strikingly, the removal of this single GGGG motif, which is predicted to disrupt the rG4 (Fig EV5F), was sufficient to dramatically increase PE inclusion and almost completely incapacitate its skipping upon cooling, but not at 37°C (Fig 5D), recapitulating PE inclusion due to HNRNPH1 KD at 32°C (Fig 4E). Unlike in RBM3 WT minigene, HNRNPH1 overexpression in HEK293T cells at 37°C was unable to repress PE inclusion in the delGGGG minigene when the primary HNRNPH1 binding site was abolished (Fig EV5G). Taken together, the results show that HNRNPH1 interacts with the G‐rich motif within the PE of RBM3 mRNA to repress PE inclusion most efficiently upon cooling.

## Discussion

Using a genome‐wide CRISPR/Cas9 gene KO screen, we identified key transregulatory factors for neuronal RBM3 cold induction. The strength of our screen lies in its closely recapitulating the physiological scenario, because we chose to (i) GFP‐tag endogenous RBM3 loci to account for any regulatory element beyond the coding sequence, (ii) use differentiated i‐neurons with functional synapses, and (iii) precool at 32°C to extend the dynamic range of GFP‐RBM3 fluorescent readings affected by positive and negative regulator depletion. While splicing regulating proteins were identified among the strongest RBM3 regulators, our screen also indicated that RBM3 protein expression is controlled at multiple levels, from transcription, translation to protein degradation. It will be interesting to explore the overlap between cooling‐dependent modulators of RBM3 identified in this study and regulators involved in changes in RBM3 levels by other known stimuli, such as BDNF/TrkB signalling cascade we previously reported (Peretti *et al*, 2021).

The appearance of HNRNPH1 as the top positive regulator of RBM3 together with other splicing factors prompted us to investigate cold‐induced splicing changes in RBM3 mRNA. In line with this, we report cooling‐dependent PE exclusion as a level of regulation governing RBM3 induction. Interestingly, this alternative splicing regulation is conserved in different cell types and between human and mouse (Preußner *et al*, 2023). Depletion of HNRNPH1 using different methods disrupted the alternative splicing control around RBM3 PE in endogenous RBM3 mRNA and in an externally introduced minigene. Moreover, removal of the poly‐G stretches within the PE significantly repressed the removal of PE in RBM3 transcript, further conclusively establishing the role of HNRNPH1 binding.

Although HNRNPH1 KD resulted in a proportionally similar reduction in RBM3 mRNA and protein expression at 37 and 32°C (Figs 2A, D, and E, and EV2I), it is interesting that depletion of HNRNPH1 enhanced RBM3 PE retention at 32°C but exerted little impact at 37°C in HeLa cells (Fig 4D and E), supporting a cold‐dependent efficacy of HNRNPH1 in RBM3 PE repression. The discrepancy between the temperature‐independent regulation of RBM3 expression and the temperature‐dependent control of RBM3 PE inclusion by HNRNPH1 may be a result of nonsplicing regulatory factors, such as the NMD pathway, which may also function with different capacities in a temperature‐sensitive manner. Notably, the extent of HNRNPH1‐mediated PE repression also varies between different cell types: in HeLa cells, HNRNPH1 KD only led to PE inclusion at 32°C (Fig 4D and E), while HNRNPH1 depletion in i‐neurons enhanced PE inclusion by 30% at 37°C and > 50% at 32°C (Fig 4A and B). The variable endogenous protein levels of HNRNPH1 and other relevant RBPs may contribute to the cross‐cell‐type differences.

The lower efficiency of HNRNPH1 in RBM3 PE repression at 37°C can be compensated by its overexpression (Fig 4F–H), but for now it remains unclear why it is more efficient at 32°C, since its expression is similar at both temperatures (Fig 5A). Interestingly, we observed higher binding affinity between HNRNPH1 and RBM3 mRNA (Fig 5B) in cooled cells, which could explain its higher activity, and may be explained by either or both of the following mechanisms. First, HNRNPH1 interacts with G‐tracts and rG4s to promote exon skipping, as reported for HNRNPH1‐mediated exon exclusion in RNA‐binding protein EWS (EWSR1) transcripts (Neckles *et al*, 2019; Vo *et al*, 2022). The potential formation of rG4s at the HNRNPH1 binding sites (Fig 5C) suggests that temperature‐regulated rG4 stability may account for HNRNPH1‐mediated RBM3 PE skipping. Alternatively, post‐translational modification (PTM) and/or temperature‐driven condensation of HNRNPH1 (Kim & Kwon, 2021) might modify its affinity for targeted RNA regions, which would be interesting to explore. Temperature variation can result in markedly different PTM patterns shaped by temperature‐sensitive PTM enzymes (Cai *et al*, 2018), including kinases (Haltenhof *et al*, 2020) and arginine methyltransferases (Hong *et al*, 2010). In fact, *in vitro* and *in vivo* evidence supports temperature‐dependent changes in RNA‐RBP condensation (Molliex *et al*, 2015; Riback *et al*, 2017; Iserman *et al*, 2020; Pullara *et al*, 2022) as a result of differential intermolecular interactions (Tauber *et al*, 2020), possibly linked to distinct PTM of the embedded RBPs (Hofweber & Dormann, 2019; Sridharan *et al*, 2022).

Interestingly, HNRNPH1 missense mutations in the nuclear localisation sequence and nonsense mutations leading to reduced protein levels have been identified in individuals with neurodevelopmental disorders and intellectual impairment (Reichert *et al*, 2020; Gillentine *et al*, 2021). A possibility is that these mutations may act, in part, through impaired HNRNPH1 induction of RBM3 expression, affecting its roles in neurogenesis (Zhu *et al*, 2019) and/or synaptic maintenance (Peretti *et al*, 2015, 2021). In addition, HNRNPH1 sequestration in RNA foci has been implicated in neurodegenerative disease models of frontotemporal dementia, associated with impaired splicing functions (Bampton *et al*, 2020). It would be interesting to explore the effect of these HNRNPH1 mutations, and its sequestration in RNA foci, on RBM3 splicing and hence RBM3 protein levels.

The fate of inclusion or exclusion of alternative exons is a consequence of the interplay between the recruited splicing factors and the cis‐acting elements with varying affinities, opening up opportunities for targeting different steps during mRNA splicing for therapeutic intervention (El Marabti & Abdel‐Wahab, 2021). While small molecules affecting global splicing activity have been applied to cancer treatments (Agrawal *et al*, 2018), modulating specific splicing events by targeting cis‐acting elements using splice‐switching antisense oligonucleotides (ASOs) show therapeutic benefit in several genetic diseases, including spinal muscular atrophy and Duchenne muscular dystrophy (Havens & Hastings, 2016). Most recently, ASOs designed to repress RBM3 PE inclusion successfully upregulated RBM3 expression, without cooling, leading to marked neuroprotection in mice with prion neurodegeneration (Preußner *et al*, 2023), consistent with the protective effects of inducing RBM3 by hypothermia (Peretti *et al*, 2015) or other means (Peretti *et al*, 2021). Beyond ASO technology, the past two decades have witnessed the development of splicing control using bifunctional oligonucleotides, consisting of an antisense domain complementary to the mRNA region close to the splice site, and a tail domain recruiting RBPs to promote exon inclusion or exclusion as an alternative approach (Skordis *et al*, 2003; Zhou, 2022). The identification of HNRNPH1 in this study as an RBM3 PE repressor, among other splicing factors, brings the potential to apply such technology to novel targets for RBM3 therapeutic induction in clinical scenarios ranging from acute brain injury to neurodegeneration.

## Materials and Methods

### Human iPSC culture

NGN2‐OPTi‐OX was generated by the Kotter laboratory at the University of Cambridge (Pawlowski *et al*, 2017), where the original iPSC line was sourced from the University of Cambridge (https://hpscreg.eu/cell‐line/CAMi014‐A). iPSCs with Neurogenin‐2 (NGN2) transgene stably integrated into a “safe‐harbour” locus under doxycycline (Dox)‐inducible promoter (Pavlou *et al*, 2023) were maintained under feeder‐free conditions in TeSR‐E8 medium in a 37°C, 5% CO_2_ tissue culture incubator. They were cultured on vitronectin (3.3 μg/ml)‐coated culture plates or glass‐bottom dishes and fed every day with TeSR‐E8 medium or every 2 days with StemFlex Medium. 0.5 mM EDTA was used for routine dissociation to maintain colony growth. iPSCs were frozen in Cryostor Cs10 Cryopreservation.

### iPSCs differentiation into iPSC‐derived neurons (i‐neurons)

iPSCs were enzymatically detached and dissociated into single cells using Accutase and plated into GelTrex (1:100 dilution)‐coated culture plates in TeSR‐E8 medium supplemented with 10 μM Rho‐associated protein kinase (ROCK) inhibitor. After 24 h (Day 1), TeSR‐E8 medium was changed to DMEM/F12 medium with GlutaMAX, supplemented with 1x N‐2 supplement, 1x Non‐Essential Amino Acids, 50 nM 2‐Mercaptoethanol, 100 U/ml Penicillin–Streptomycin, and 1 μg/ml Doxycycline Hyclate (Dox) (iN‐1 medium). After 24 h (Day 2), the medium was replaced with the same medium as the previous day. From Day 3 to Day 6, the culture was fed daily with Neurobasal medium supplemented with 1x B‐27 supplement (minus vitamin A), 1x GlutaMAX, 50 nM 2‐Mercaptoethanol, 100 U/ml Penicillin–Streptomycin, and 1 μg/ml Dox, 10 ng/ml Neurotrophin‐3 (NT‐3), and 10 ng/ml Brain‐derived neurotrophic factor (BDNF) (iN‐2 medium). After Day 6, the medium was changed every other day. Cultures used for flow cytometry were prepared by dissociating Day 4 iPSCs with Accutase and plating 80,000 cells per well in a 96‐well culture plate precoated with GelTrex (1:100 dilution). The same feeding schedule as previously described was followed from Day 5. To prepare cultures for live fluorescent imaging, Day 4 iPSCs were dissociated with Accutase and plated 10,000–20,000 cells/dish on 35 mm MatTek glass‐bottom dishes precoated with 0.1 mg/ml poly‐L‐lysine and 10 μg/ml laminin in iN‐2 medium with ROCK inhibitor. The same feeding schedule as previously described was followed from Day 5. I‐neurons in the 32°C conditions were placed in a 5% CO_2_ incubator set to 32°C for 72 h between Day 15–18 post‐differentiation. 1 μM actinomycin D (ActD) and 50 μM cycloheximide (CHX) were incubated for 72 h, and SMG1 inhibitor for 24 h before collection.

### HeLa and HEK293T cell cultures and transfection

HeLa and HEK 293 T cells were grown in Dulbecco's modified Eagle's medium (DMEM) + Ham's F12 (1:1) supplemented with 10% fetal calf serum (FCS), penicillin (100 IU/ml), and streptomycin (100 μg/ml) and grown at 37°C and 5% CO_2_. Plasmid DNA transfection was performed with Lipofectamine 2000 (Thermo Fisher Scientific) or TransIT‐LT1 (Mirus) according to the manufacturer's instructions.

For HNRNPH1 siRNA transfections, 250,000 HeLa cells were seeded in a six‐well plate and the next day cells were transfected with two siRNAs 10 nM siRNA each (siRNA#1 + siRNA#2) using Lipofectamine 2000. After 24 h, a second round of siRNA transfection was done in fresh medium with or without RBM3 minigene cotransfection. One set of the cells was moved to 32°C while the other set was kept at 37°C. 24 h later, medium was changed again with fresh medium with or without SMG1 inhibitor (1 μM). SMG1 inhibitor (1 μM) and cycloheximide (200 μg/ml) were incubated for 24 h before harvesting the cells.

### Plasmids, oligonucleotides and guide RNAs

All primers used in this study are listed in Appendix Table S1, and guide RNAs (gRNAs) and siRNAs in Appendix Table S7.

GFP‐RBM3 repair template plasmid: To prepare iPSC cDNA, RNA was purified from iPSC pellets using the RNeasy Mini kit and reverse transcribed into cDNA using the oligo(dT) primer and the SuperScript IV First‐Strand Synthesis System. The following fragments were amplified by PCR using Q5 DNA Polymerase and the indicated templates and primers (Appendix Table S1): (i) The 5′ homology arm (1 kb sequence immediately upstream of RBM3 start codon) was amplified from iPSC cDNA using Primers Pr1 and Pr2; (ii)The GFP fragment was amplified from pcDNA3‐EGFP using Primers Pr3 and Pr4; (iii) The 3′ homology arm (start codon and 1 kb sequence at it immediate downstream) was amplified from iPSC cDNA using Primers Pr5 and Pr6; (iv) the origin of replication and ampicillin‐resistant gene were amplified using Primers Pr7 and Pr8. The repair template plasmid for CRISPR knocked in of GFP to the N‐terminus of the RBM3 coding region (GFP‐RBM3 repair template) was generated by assembling Fragments a‐b‐c‐d in the indicated order using NEBuilder. Plasmids were validated by Sanger sequencing. Single‐stranded DNA (ssDNA) were subsequently synthesised from these repair template plasmids to improve CRISPR knock‐in efficiency using Guide‐it Long ssDNA Production System with Primers Pr9 and Pr10 according to manufacturer instructions. The 19‐nucleotide (nt) guide RNA (gRNA) RBM3‐N gRNA#1 (5′‐CUGCCAUGUCCUCUGAAGA‐3′) and #2 (5′‐UUUCCUUCUUCAGAGGACA‐3′) followed by the protospacer adjacent motif (PAM) targeting the region adjacent to the RBM3 start codon were resuspended in water to the concentration of 4 μg/μl.

RBM3 minigene expression plasmids: RBM3 minigene was cloned by amplifying genomic regions from exon 1–4 using forward primer – AAGAATTCATGTCCTCTGAAGAAGGAAAGC and reverse primer – TTTGCGGCCGCCTCTAGAGTAGCTGCGACCACGCC and then inserting in EcoRI‐NotI sites in pcDNA3.1(+) vector backbone. Minigene Del GGGG mutant was generated using site‐directed mutagenesis (QuickChange Lightning Multi Site‐Directed Mutagenesis Kit) using the primer‐ GTCGGGCAGCTTAGAGGAGTAGGAGAACTCAG.

FLAG‐HNRNPH1 plasmid: FLAG‐HNRNPH1 expression construct was generated by inserting HNRNPH1 coding sequence, obtained as a string synthesised (GeneArt, Thermo Fisher Scientific), in XbaI‐NotI sites in pcDNA6F vector backbone.

hPGK:BFP lentiviral plasmid: The BFP‐expressing control plasmid is ordered from VectorBuilder according to a custom design. Briefly, a TagBFP2 (VectorBuilder) is cloned into a lentiviral gene expression vector pLV (VectorBuilder) containing a human PGK promoter.

hPGK:HNRNPH1‐T2A‐BFP lentiviral plasmid: All fragments were amplified using Q5 Hotstart master mix from New England Biolabs. The vector was amplified from pLV‐hPGK:BFP in two halves using primers Pr15 and Pr16 giving a 3,329 bp product and primers Pr17 and Pr18 giving a 3,383 bp product. The HNRNPH1 insert was amplified with primers Pr19 and Pr20 from pcDNA3‐HNRNPH plasmid, giving a product of 1,400 bp. T2A‐TagBFP insert was amplified using primers Pr21 and Pr22 from a previously generated pLV‐EIF1A:RBM3‐T2A‐BFP plasmid, yielding a product of 790 bp. All fragments were gel purified using a Qiagen kit following the manufacturer's guidelines. The final vector was assembled using the Klenow Assembly Method. Sequences within the promoter and insert regions were confirmed by Sanger sequencing.

### Generation of GFP‐RBM3 iPSCs by CRISPR

Half a million wild‐type iPSCs were electroporated with Cas9 protein, RBM3 gRNAs and GFP‐RBM3 repair template using Lonza Nucleofector Technology according to the manufacturer's instructions. Briefly, Cas9‐RNP complex mixture containing 0.2 μl 3 M NaCl, 1 μl RBM3‐N gRNA#1, 1 μl RBM3‐N gRNA#2, 1 μl Cas9 protein (HiFi Cas9 nuclease V3, 10 μg/μl) were assembled and incubated at room temperature for 45 min. 0.5 million iPSCs were resuspended with the nucleofector solution (90 μl P3 solution and 20 μl supplement). 3 μl of the repair template ssDNA (4 μg/μl) was added to the pre‐assembled Cas9‐RNP, mixed with the iPSC suspension, and transferred to the Nucleocuvette Vessels. Placed the vessel into the nucleofector unit and started the programme to complete electroporation. Immediately after, the electroporated cells were gently transferred to the 4 ml prewarmed StemFlex medium supplemented with 10 μM ROCK inhibitor and 40 μl homology‐directed repair (HDR) enhancer and plated into 2 vitronectin‐coated wells in a six‐well plate. The cells were then incubated at 32°C for 48 h. The following day after electroporation, the medium was replaced with StemFlex medium and replaced every other day. When reaching 70–80% confluency, cells in one well were frozen in Cryostor Cs10 Cryopreservation at −80°C and those in the other were detached and dissociated with Accutase.

Isolated iPSCs were plated in a vitronectin‐coated 96‐well plate in StemFlex medium supplemented with 10 μM ROCK inhibitor, with 30–50 cells per well. From the next day, the iPSCs were fed with StemFlex medium every other day until confluent. Confluent wells were dissociated and half of the cells were subject to flow cytometry measurement (see Flow cytometry) to determine the GFP intensity of iPSCs in each well. 500–750 cells from each of the wells with GFP‐positive cells were plated in a vitronectin‐coated 10‐cm Petri dish in StemFlex medium supplemented with 10 μM ROCK inhibitor. From the next day, the hiPSCs were fed with StemFlex medium every other day until colonies were formed (1–2 weeks). Imaged with a wild‐field fluorescent microscope, GFP‐positive colonies were picked by gentle aspirating with P1000 pipette tips and transferred to a well in round‐bottom 96‐well plates with 200 μl StemFlex medium with 10 μM ROCK inhibitor and 100 U/ml Penicillin–Streptomycin per well. Once finished colony picking, each well was split into two vitronectin‐coated flat‐bottom 96‐well plates by gentle pipetting to break colonies into small clusters and transferring 100 μl cell suspension in each well into a well in new plates. Each well was fed StemFlex medium every other day until the majority of the wells reached 50–60% confluency. Genomic DNA of individual colonies in one of the duplicated plates was extracted (see Genomic DNA extraction) and PCR‐genotyped using GoTaq Taq G2 Green Master Mix and primer pairs Pr11/12, Pr11/14, and Pr12/13. The correctly genotyped clones in the corresponding second plates were expanded in six‐well plates to be further validated by Sanger sequencing. Sequence‐verified clones were aliquoted and frozen at −80°C.

### Genomic DNA extraction

iPSCs were detached using Accutase and pelleted by centrifugation at 250 *g* for 5 min. After removing the supernatant, 50 μl (each well of a 96‐well plate) or 5–10 μl (every 1,000 iPSCs) lysis buffer (and 1:40 freshly added Proteinase K) was added to the pellet and lysed at 55°C overnight in a shaker. The next day, 10% volume of 3 M sodium acetate (pH 5.2) and an equal volume of isopropanol were added to the lysate and mixed by brief vortexing. Genomic DNA was pelleted by 15‐min centrifugation at maximum speed, followed by two washes with 200–1,000 μl of 80% ethanol. After the last centrifugation to remove the residual ethanol, pellets were left at room temperature to air dry, and resuspended in 50–500 μl TE buffer (10 mM Tris–HCl, pH 8.0 and 0.1 mM EDTA).

### Western blotting

Protein concentrations of lysates were determined by BCA assay following the manufacturer's instruction. Samples were diluted with Laemmli protein sample buffer with 100 mM DTT. 10–15 μg protein was loaded into each well in precast 12% gels or 4–15% gradient gels and ran at 125 V. Proteins were transferred to a 0.2 μm nitrocellulose membrane at 70 V for 2 h in a wet blot system. Membranes were blocked with 5% BSA or 5% non‐fat milk in 1x TBS‐T for 1 h rotating at room temperature. The primary antibody solution was incubated overnight at 4°C while rotating. The next day, membranes were washed three times with 1x TBS‐T, then incubated for 1 h in secondary antibody solution (1:10,000 in 5% BSA or 5% nonfat milk in 1x TBS‐T) and washed three times with 1x TBS‐T before adding HRP chemiluminescent substrate to develop on a ChemiDox MP Imaging System or directly on LI‐COR Odyssey CLx Primary and secondary antibodies were used in the following concentrations: rabbit anti‐RBM3 (1:1000), mouse anti‐Cas9 (1:1,000), mouse anti‐GAPDH (1:2,000), mouse anti‐Cas9 (1:2,000), rabbit anti‐HNRNPH1 (1:1,000, Abcam), rabbit anti‐HNRNPH1 (1:1,000, ProteinTech, Fig EV4F), mouse anti‐FLAG (1:2,000), mouse anti‐HNRNPH/F (1:1,000 Santa Cruz), rabbit anti‐SmD3 (1:1,000), donkey anti‐rabbit 680LT (1:10,000), donkey anti‐mouse 680LT (1:10,000), donkey anti‐rabbit 800CW (1:10,000), donkey anti‐mouse 800CW (1:10,000), goat anti‐rabbit and goat anti‐mouse HRP conjugated secondary antibodies (1:10,000, Biorad).

### Lentiviral production

96‐well plates or T75 flasks precoated with 25 μg/ml PLL at 37°C overnight, followed by three times washes with distilled water and left to dry in tissue culture hoods. HEK293FT were plated at 10^6^ cells/cm^2^ density and incubated at 37°C overnight. Lentiviral expression and packaging plasmids were transfected using Lipofectamine LTX. For each well of a 96‐well plate (upscale proportionally for T75 flask transfection), Mix A: 20 μl Opti‐MEM, 19.12 ng psPax2, 12.5 ng pMD2.G, 25 ng expression plasmid, 0.1 μl Plus Reagent; Mix B: 5 μl Opti‐MEM, 0.3 μl Lipofectamine LTX were assembled and incubated at room temperature for 20 min before adding to the HEK293FT cultures. After 24 h, media were replaced with fresh media and checked for BFP expression. Viruses were harvested 72 h post‐transfection by centrifuging at 6,000 *g* at 4°C overnight. The pellets were resuspended with PBS and frozen at −80°C for storage.

### Flow cytometry

Dissociated cells were resuspended in culture media in 96‐well plates and fluorescent intensities of GFP, mCherry and/or BFP were measured at room temperature with a CytoFLEX S System (Beckman Coulter) and CytExpert (v2.4) Program. Acquired data were analysed with FlowJo (v10.7.2). Briefly, successfully transduced cells were gated sequentially for cells (FSC‐A vs SSC‐A), singlets (FSC‐H vs FSC‐A) and BFP (FSC‐A vs PB450‐A). The background fluorescence was gated with non‐transduced WT cells. For editing efficiency measurement, mCherry (ECD‐A) intensities were measured within the BFP‐positive Cas9 WT or GFP‐RBM3 reporter i‐neurons and unedited cells were gated with the WT i‐neurons expressing the reporter lentivirus. Median GFP (FITC‐A) intensities within the gated population were automatically calculated by FlowJo.

### GFP‐RBM3 i‐neuron live imaging

GFP‐RBM3 iPSCs and i‐neurons cultured at low density on vitronectin (iPSC) or PLL and laminin (i‐neuron)‐coated 35‐mm glass bottom dishes were imaged in TeSR‐E8 (iPSC) or phenol red‐free iN‐2 media (i‐neuron, to minimise autofluorescence) in OkoLab temperature‐controlled chamber. Cells were imaged at 37 or 32°C with 5% CO_2_ on a custom‐built wide‐field microscope in Epi fluorescence mode with a 100x oil TIRF objective (Olympus). GFP was excited with a 488 nm laser at 5 mW. Hoechst nuclear stain or BFP of lentiviral transduced cells was excited with a 504 nm laser at 10 mW. Both channels were imaged simultaneously and separated with OptoSplit III System (Cairn Research) and a Prime BSI camera (Photometrics).

### RBM3 CRISPR knockout screen

Both clones of GFP‐RBM3 iPSCs were differentiated in GelTrex‐coated T75 flasks. The total number of i‐neurons used for each clone in each replicate was approximately 60,000,000 to ensure sufficient copies of individual sgRNAs could be obtained from the pool. 4 days of postdifferentiation, the cultures were transduced with a whole‐genome sgRNA lentiviral library (399 non‐targeting sgRNAs and 91,138 sgRNAs targeting 18,466 genes across the human genome), co‐expressing a BFP fluorescent reporter to indicate successfully transduced cells. The viral titre was determined in pilot experiments, resulting in 20% of the i‐neurons becoming BFP‐positive on Day 18 to minimise multiple sgRNA entries into a single cell. I‐neurons at 14, 15 or 16 days of postdifferentiation (or 11, 12, 13 days of post‐transduction) were cooled at 32°C for 72 h before dissociation with 20 min Accutase and 10 min Trypsin–EDTA incubation, followed by fluorescence‐activated cell sorting (FACS). The GFP intensity of all BFP‐positive cells was manually separated into four quartiles, and only cells with the GFP intensity falling in the top or the bottom quartile were collected in separate tubes and their genomic DNA was extracted (see Genomic DNA extraction) immediately after. Around 3,000,000 i‐neurons were collected in the low GFP or high GFP tube for each clone in each replicate.

Purified DNA was sequenced to identify sgRNAs enriched in high or low GFP populations. The sequencing library was created in a two‐stage PCR reaction. The first stage amplifies the enriched gRNA cassettes: 2 μg DNA, 1.5 μl Forward Primer (10 μM), 1.5 μl Reverse Primer (10 μM), 25 μl NEB Q5 High‐Fidelity 2x Master Mix in a 50 μl reaction. The PCR condition was: 98C for 30 s, 25 cycles of (98C for 10 s, 62C for 30 s, 72C for 15 s), and 72C for 2 min. The PCR product was purified using Ampure XP Beckman magnetic beads and diluted to 200 pg/μl. The second PCR attached the Illumina adaptors and barcodes: 1 μl PCR product from the first reaction, 0.75 μl Forwards Primer (20 uM), 0.75 μl Reverse Primer (20 uM), 10 μl Roche 2X KAPA HiFi ReadyMix in a 20 μl reaction. The PCR condition was: 95C for 3 min, 9 cycles of (98C for 20 s, 66C for 15 s, 72C for 20 s), and 72C for 1 min. PCR products were purified using Ampure XP Beckman magnetic beads and eluted in 35 μl TE buffer. PCR products were quantified with NEBNext Library Quant Kit for Illumina. The library was run on an Illumina NextSeq 550, using an Illumina 75 cycle, high output kit at 1.4 pM.

### Arrayed CRISPR target validation

Inserts containing the candidate gene targeting sgRNA (1–3 sgRNAs per gene) sequences and non‐targeting sgRNA sequences (see Appendix Table S4) were cloned into a lentiviral expression vector pLVPB‐U6‐sgRNAv2fl_shortccdB_PGK_Puro_BFP linearised with BbsI, which removed the suicide ccdB cassette. The sgRNA expression plasmids were verified by Sanger sequencing and used to generate lentiviral particles (see Lentiviral production) in a 96‐well array format. GFP‐RBM3 Clone 1 iPSCs at 4 days after differentiation in 96‐well plates were transduced with the arrayed lentiviral library at a viral concentration predetermined in pilot experiments to obtain maximal transduction efficiency. 15 days of postdifferentiation, GFP‐RBM3 i‐neurons were either cooled at 32°C for 72 h or continue to grow at 37°C. On day 18, BFP and GFP intensities of the transduced cultures were measured using flow cytometry (see Flow cytometry).

### RNA‐seq

Four biological replicates of WT i‐neurons in control (37°C) and cooled (72 h at 32°C, Days 15–18) were included in this study. Total RNA was extracted with RNeasy Plus Mini Kit. RNA concentrations of individual samples were measured using Qubit RNA Broad Range Assay Kit and their integrity was determined using Agilent TapeStation System. The library was prepared using Illumina TruSeq Stranded mRNA Library Prep following the manufacturer's instructions and sequenced paired‐end 150 bp on Novaseq.

### HNRNPH1 RNA immunoprecipitation

HeLa cells were seeded at 30% confluency in two T75 flasks. 24 h of postseeding, one flask was moved to a 32°C incubator and the other one was maintained at 37°C for the next 48 h until harvesting. Cells were treated with SMG1 inhibitor (1 μM) 24 h before harvesting. Cells were harvested by scraping and washed with 1x Phosphate buffered saline (PBS). To prepare cell extracts, the cell pellets were dissolved in 800 μl Gentle Hypotonic Lysis Buffer (10 mM Tris pH 7.2, 10 mM NaCl, 2 mM EDTA, 0.5% Triton‐X‐100) supplemented with 1x HALT protease and phosphatase inhibitor cocktail (Thermo Scientific, 78442), 0.06 U/μl DNase1 (Zymo, E1011‐A) and 0.5 U/μl RiboLock RNase inhibitor (Thermo Scientific, EO0381) and incubated on ice for 10 min. After adjusting the NaCl concentration to 150 mM, the extracts were incubated for another 5 min on ice and then cleared by centrifugation at 15,000 *g* and 4°C for 15 min. 50 μl Protein G Dynabeads (Life Technologies, 100090) per Immunoprecipitation were washed two times with TBS supplemented with 0.05% NP‐40 (IGEPAL CA‐630) and incubated in 600 μl total volume of TBS‐0.05% NP‐40 with 5 μg of rabbit anti‐HNRNPH1 (Abcam, ab13074) or rabbit IgG (Santa Cruz, sc2027) head‐over‐tail for 2 h at 4°C. The beads were subsequently washed three times with 1 ml TBS‐0.05% NP‐40 (supplemented with RNase and protease inhibitors). To keep protein input fractions, 30 μl of the cleared extracts were boiled with 2x LDS loading buffer. For RNA input fractions, 50 μl were transferred to 1 ml TRIzol (Invitrogen, 15596018) supplemented with 0.14 M β‐mercaptoethanol (Applichem, A1108). 700 μl of the extracts were then distributed to Eppendorf tubes containing antibody‐bound beads and incubated on a rotary wheel for 2 h at 4°C. After washing the beads five times with 1 ml TBS‐0.05% (NP‐40), 10% of the beads were transferred into a separate Eppendorf tube and boiled in 2× LDS loading buffer for 5 min at 95°C, and 1 ml TRIzol supplemented with 0.14 M β‐mercaptoethanol was added to the remaining beads. Proteins were analysed using 4–12% Bis‐Tris polyacrylamide gels followed by western blot using the following antibodies: Mouse anti‐HNRNPH/F (Santa Cruz, sc‐32310, 1:1,000). RNA was isolated from TRIzol according to the manufacturer's protocol and then reverse transcribed and analysed by RT‐qPCR. The fold change of HNRNPH1‐pulled down RBM3 mRNA on cooling is normalised to the change of total RBM3 mRNA and HNRNPH1 immunoprecipitation efficiency (the ratio between HNRNPH1 pulled down and total HNRNPH1) at 37°C vs. 32°C of individual replicates.

### RT‐PCR and qRT‐PCR

RNA of i‐neurons, HeLa and HEK293T cells was extracted using the RNeasy Plus Mini kit or Absolute RNA miniprep kit and reverse transcribed into cDNA using random hexamers and the SuperScript IV First‐Strand Synthesis System or AffinityScript Multiple Temperature Reverse Transcriptase. When using the latter method, 1–2 μg of isolated RNA was incubated with random hexamers (900 ng) in a total volume of 67 μl in DEPC water at 65°C for 5 min and then for 10 min at room temperature. Then, the mixtures were separated into 2 parts – RT and no‐RT control samples (33.5 μl each). To this 13.5 μl master mix was added to supplement 1x reverse transcriptase buffer (Agilent), 10 mM DTT (Agilent), 400 μM dNTPs (Thermo Scientific), 40 units RiboLock RNase inhibitor (Thermo Scientific) and 1 reaction equivalent AffinityScript Multiple Temperature Reverse Transcriptase (Agilent). The RT enzyme was absent in the no‐RT condition. The samples were then incubated at 65°C for 1 h followed by heat‐inactivation at 75°C for 20 min.

For RT‐PCR, 20–40 ng cDNA was amplified using GoTaq Polymerase mix using the indicated primers (Appendix Table S1). Quantification of the bands on agarose gels was done using FIJI software. Percent spliced in (PSI) indexes of RBM3 poison exon (PE) are calculated based on the intensity of PE‐included (red arrows) and PE‐skipped (green arrows) isoforms visualised in agarose gels:

- One PE‐included isoform is detected: (I_Inc_/L_Inc_) / (I_Inc_/L_Inc_ + I_skip_/L_skip_), or
- Two PE‐included isoforms are detected: (I_Inc1_/L_Inc1_ + I_Inc2_/L_Inc2_) / (I_Inc1_/L_Inc1_ + I_Inc2_/L_Inc2_ + I_skip_/L_skip_)

Where I_Inc_ = Intensity of PE‐included isoform, L_Inc_ = length of PE‐included isoform in base pairs, I_Inc_ = Intensity of PE‐skipped isoform, L_skip_ = length of PE‐skipped isoform in base pairs.

For qRT‐PCR, cDNA of each sample (diluted 10–1,000 times) was mixed with SYBR Green PCR Master Mix and PCR primers in 4 technical replicates in 384‐well plates. Reactions were performed in a QuantStudio Real‐Time PCR system (ThermoFisher Scientific). Analysis was performed with Design and Analysis Software (v2.6.0, ThermoFisher Scientific). Relative PE inclusion is the ratio of the relative expression levels between RBM3 Exon 3a amplicon and RBM3 Exon 3 or Exon 4–5 amplicon (or their geometric mean values). 18 s rRNA served as a loading control when quantifying RBM3 and HNRNPH1 mRNA levels.

### Immunocytochemistry and wild‐field imaging

I‐neurons cultured at low density on PLL and laminin‐coated 35‐mm glass bottom dishes were washed once with PBS before fixed with fixation buffer (4% PFA, 4% sucrose in PBS). Cell membrane was permeabilized with 0.5% Triton in PBS for 15 min and incubated in blocking solution (5% donkey serum in washing buffer) for 30 min at room temperature. After washing once with PBS, samples were incubated overnight with primary antibodies against HNRNPH1 (1: 500, ab10374, Abcam) at 4°C. On the next day, cell cultures were washed 3 times with PBS and incubated with secondary antibodies (1:1,000, A11011, Alexa Fluor™ 568, Invitrogen) for 1 h at room temperature. The neurons were washed three times and Hoechst (20 mM, 1:1,000) was added for 10 min before washing three more times and kept in PBS for imaging.

Images were acquired on a Ti2‐E High Content Microscope (Nikon) with a 60x oil objective (Nikon). A bright‐field, a 560 nm (HNRNPH1) and a 405 nm (Hoechst) images were taken sequentially with pre‐optimised imaging settings, which remained constant through the imaging session.

### SmB immunoprecipitation

For SmB immunoprecipitation, HeLa cells were seeded at 30% confluency in two T75 flasks. 24‐h postseeding, one flask was moved to a 32°C incubator and the other one was maintained at 37°C for the next 72 h until harvesting. Cells were harvested by scraping and washed with 1x Phosphate buffered saline (PBS). Lysis was done in 600 μl RIPA lysis buffer supplemented with 1x Halt Protease Inhibitor cocktail (Thermo Scientific, 1861278), followed by sonication at 40% amplitude on ice. The extracts were then cleared by centrifugation at 15,000 *g* and 4°C for 15 min. For input fractions, 60 μl of the cleared extracts (1/10^th^) were boiled with 2x LDS loading buffer. 80 μl Protein G Dynabeads (Life Technologies, 100090) per immunoprecipitation were washed twice with TBS supplemented with 0.05% NP‐40 (IGEPAL CA‐630) and incubated in 600 ul total volume of TBS‐0.05% NP‐40 with 10 μg of mouse anti‐SmB or mouse IgG (Santa Cruz, sc2025) head over tail for 1.5 h at 4°C. The beads were then washed twice times with 1 ml TBS‐0.05% NP‐40 and were then resuspended in 270 μl of lysate and incubated on a rotary wheel for 2 h at 4°C. Subsequently, the beads were washed three times with RIPA lysis buffer and once with TBS‐0.05% NP‐40. With the final wash, the beads were transferred to new tubes, wash buffer was removed, and the beads were resuspended in 70 μl of 2x LDS‐loading buffer, boiled for 5 min at 95°C and loaded on a 4–12% Bis‐Tris polyacrylamide gels followed by western blotting. For SmB immunoprecipitation in I‐neurons, the same protocol was followed as HeLa except that the lysis was done in 700 μl RIPA lysis buffer, and the input fractions and lysates for incubation with the beads were 70 and 300 μl, respectively.

### Data analysis

#### Image quantification of live or fixed iPSCs and i‐neurons

Regions with single cells were cropped from the full‐size images with a custom‐made program. Then, each single‐cell image was analysed with a custom‐made MatLab pipeline.

- For Fig 1A, nuclei were defined by Hoechst signals excited at 405 nm, and cell boundaries were defined by GFP‐RBM3 signals acquired with 488 nm excitation.
- For Fig EV2A, lentiviral BFP expression indicated successfully transduced cells and the BFP fluorescence was used to define soma boundary. Nuclei were defined as areas where GFP was expressed at higher intensities, as RBM3 is predominantly nuclear. This was achieved by applying automatic global Otsu's threshold to the GFP channel after background removal.
- For Fig EV5C, nuclei were defined by Hoechst signals excited at 405 nm, and cell boundaries were defined by HNRNPH1 signals acquired with 560 nm excitation.

Cytoplasm was defined as the area outside of the nucleus, within the soma. After segmentation, average intensities (total intensity divided by unit area) of GFP‐RBM3 or HNRNPH1 (Alexa 568) fluorescence in each of three cellular compartments, soma, cytoplasm and nuclei, were calculated.

#### Whole‐genome CRISPR screen next‐generation sequencing analysis

21 nt long sequencing reads were exported from bcl files using bcl2fastq v2.2.0. These reads were counted by converting them to k‐mers and mapping them to a set of 91,536 valid CRISPR library 20 nt gRNA sequences. Reads without an exact match in the library were discarded. After merging the sample counts into a count table, the samples were inspected for proper gRNA infection and coverage. With all the samples passing QC, the MAGeCK RRA (Li *et al*, 2014) was used to perform gene essentiality and enrichment inference. To run MAGeCK, we used the paired samples option, which contained the sorted samples: low GFP sample in control group and high GFP samples in the treatment group and a set of nontargeting gRNAs as controls. Functional and network analyses of top RBM3 regulator candidates with FDR <0.05 were performed with Metascape (Zhou *et al*, 2019) and STRING (Szklarczyk *et al*, 2020).

#### RNA‐seq analysis

Fastq files of sequencing reads were processed directly with the nf‐core (Ewels *et al*, 2020) rnaseq pipeline (v3.3) (Patel *et al*, 2021), mapped to hg38 genome with the gencode v38 annotation (GRCh38.p13). A gene expression quantification table was generated from the salmon output gene counts using the star_salmon aligner option within the nf‐core/rnaseq pipeline. Differential expression analysis was performed using DESeq2 (v3.15). Grouped sashimi plots to visualise alternative splicing of RBM3 mRNA between control and cooled i‐neurons were generated using rmats2sashimiplot (v2.0.4) and BAM files.

#### ENCODE project data analysis

RBM3 TPM values in RNA‐Seq experiments following targeted CRISPR editing in HepG2 and K562 cell lines, together with the matching control samples, were extracted from the publicly available HNRNPH1 (ENCFF039DFP, ENCFF713MXN, ENCFF616BYI, ENCFF586TGE, ENCFF293ODK, ENCFF266YWO, ENCFF053QJC, ENCFF200BWY), SNRNP70 (ENCFF367WUN, ENCFF058OGQ, ENCFF873KLR, ENCFF311ACC), PUF60 (ENCFF682SFK, ENCFF461EMF, ENCFF585BAL, ENCFF643OYR) and KPNB1 (ENCFF819AUU, ENCFF628VDB, ENCFF565TSG, ENCFF073IFA)‐knockdown datasets of the ENCODE Project.

To perform splicing analysis on RBM3 mRNA in control and HNRNPH1 knocked‐down HepG2 and K562 cells. Raw Fastq files were downloaded from the European Nucleotide Archive (ENA). Fastq files were processed through FastQC (v0.11.8) for quality control. The data quality of each Fastq file was reviewed manually. Reads were mapped to GRCh38.p13 human reference genome by STAR (v2.6.1b). Alignment results were sorted by the coordinate. Output BAM files were indexed by SAMtools (v1.9).

Splicing analysis was performed using rMATS (v4.1.2). Control and knockdown samples were grouped separately to serve as inputs of rMATS. The gene annotation file (GENECODE Human Release 34, GRCh38.p13) was used for analysis. Inclusion levels and FDR values of RBM3 Exon 3a relative to Exon 3 and 4 in the skipped exon output tables (SE.MATS.JCEC) were used to generate bar graphs indicating RBM3 Exon 3a inclusion levels (see Appendix Table S6 for corresponding entries). Grouped sashimi plots to visualise alternative splicing of RBM3 Exon 3 and 4 between control and HNRNPH1 knocked‐down samples were generated using rmats2sashimiplot (v2.0.4) and BAM files.

#### HNRNPH1 public iCLIP analysis

Sequence fastq files from public HNRNPH1 iCLIP datasets from HEK293T cells were accessed from ArrayExpress with accession numbers ERR2201859 & ERR2201860 (Braun *et al*, 2018). Samples were processed with the nf‐core/clipseq pipeline v1.0.0 (Ewels *et al*, 2020), mapped to hg38 genome with the gencode v38 annotation to obtain crosslink counts. Crosslink signal was visualised with library size normalisation and rollmean smoothing (window 5) with clipplotr (preprint: Chakrabarti *et al*, 2021) between exons 3 and 4 of RBM3 (chrX:48575560‐48576419:+). RNA G4 prediction was performed with QGRS Mapper using default settings (Kikin *et al*, 2006).

## Author contributions

**Julie Qiaojin Lin:** Conceptualization; investigation; methodology; writing – original draft. **Deepak Khuperkar:** Investigation; methodology; writing – original draft; writing – review and editing. **Sofia Pavlou:** Investigation; methodology; writing – review and editing. **Stanislaw Makarchuk:** Formal analysis; investigation; writing – review and editing. **Nikolaos Patikas:** Formal analysis; writing – review and editing. **Flora CY Lee:** Formal analysis; writing – review and editing. **Julia M Zbiegly:** Investigation; writing – review and editing. **Jianning Kang:** Formal analysis; writing – review and editing. **Sarah F Field:** Investigation. **David MD Bailey:** Investigation. **Joshua L Freeman:** Investigation. **Jernej Ule:** Resources; supervision; methodology; writing – review and editing. **Emmanouil Metzakopian:** Conceptualization; resources; supervision; methodology; writing – review and editing. **Marc‐David Ruepp:** Conceptualization; resources; supervision; methodology; writing – review and editing. **Giovanna R Mallucci:** Conceptualization; resources; supervision; methodology; writing – review and editing.

## Disclosure and competing interests statement

S.P. is now an AstraZeneca employee. E.M. is an employee and shareholder of bit.bio.

## Supporting information



Appendix S1

Expanded View Figures PDF

Source Data for Expanded View

PDF+

Source Data for Figure 1

Source Data for Figure 2

Source Data for Figure 3

Source Data for Figure 4

Source Data for Figure 5

## Data Availability

The CRISPR knockout screen data were deposited to GEO (GSE217789; http://www.ncbi.nlm.nih.gov/geo/query/acc.cgi?acc=GSE217789). The i‐neuron RNA‐seq data were deposited to ArrayExpress (E‐MTAB‐12402; http://www.ebi.ac.uk/arrayexpress/experiments/E‐MTAB‐12402/). Protocols and materials are available upon request.
