# Supplementary material for: HNRNPH1 regulates the neuroprotective cold‐shock protein RBM3 expression through poison exon exclusion
Source: EMBO J. 2023 May 30;42(14):e113168. doi: 10.15252/embj.2022113168 (PMC10350819; doi:10.15252/embj.2022113168)
Supplement: Supplementary file 1 — Appendix S1 [file EMBJ-42-e113168-s003.pdf]

Appendix Supplementary Tables

Appendix Table S1 Single-guide RNA (sgRNA) and primer (Pr) sequences used in this study (Page 2)

Appendix Table S2 RBM3 positive and negative regulator candidates identified by pooled CRISPR knockout screen (Page 5)

Appendix Table S3 A complete list of significant Gene Ontology (GO) terms of RBM3 positive and negative regulators (Page 16)

Appendix Table S4 sgRNA sequences used in arrayed target validation (Page 23)

Appendix Table S5 TPM of RBM3 mRNA upon specified RBM3 regulator KD compared to the control in K562 and HepG2 cells from ENCODE (Page 33)

Appendix Table S6 Analysis of RBM3 transcript alternative splicing in HNRNPH1 KD versus WT i-neurons, K562 and HepG2 cells by rMATS (Page 34)

Appendix Table S7 Key resources used in this study (Page 35)

Appendix Table S1 Single-guide RNA (sgRNA) and primer (Pr) sequences used in this study

Number	Name	Sequence (5' -> 3')
Pr1	AmpOri_RBM3-N-5HA_Fw	GGGTTCCGCGCACATTTCCCCGAAAAGTGCGGTGAAGGAGGTTAACATCAGAGAATGGCA
Pr2	RBM3-N-5HA_GFP_Rv	GGTGAACAGCTCCTCGCCCTTGCTCACCATGGCAGTTCAAGTCCTGTGGGAAGAACATGA
Pr3	RBM3-N-5HA_GFP_Fw	TCATGTTCTTCCCACAGGACTTGAACTGCCATGGTGAGCAAGGGCGAGGAGCTGTTCACC
Pr4	GFP_linker_RBM3-N-3HA_Rv	TCCCACGAAGAGCTTTCCTTCTTCAGAGGAGAATTCTCCTGAACCGGCCGCCGATCCTGCGGAGCCCTTGTACAGCTCGTCCATGCCGAGAG
Pr5	GFP_linker_RBM3-N-3HA_Fw	ATCACTCTCGGCATGGACGAGCTGTACAAGGGCTCCGCAGGATCGGCGGCCGGTTCAGGAGAATTCTCCTCTGAAGAAGGAAAGCTCTTCGTGGGA
Pr6	RBM3-N-3HA_AmpOri_Rv	CTGGCACGACAGGTTTCCCGACTGGAAAGCAAGCTGCCCGACTATCAGGGCTAGACAGAC
Pr7	RBM3-N-3HA_AmpOri_Fw	GTCTGTCTAGCCCTGATAGTCGGGCAGCTTGCTTTCCAGTCGGGAAACCTGTCGTGCCAG
Pr8	AmpOri_RBM3-N-5HA_Rv	TGCCATTCTCTGATGTTAACCTCCTTCACCGCACTTTTCGGGGAAATGTGCGCGGAACCC
Pr9	RBM3-N_ssDNA_PF	(5'-phosphorylated) CGTGCTTGGAGGTGAAACATTTCG
Pr10	RBM3-N_ssDNA_R	GCTCTCATGGCAACTGAAGCA
Pr11	GT_RBM3-N_ssDNA_F	TGCATATGTCCTGCGAAACG
Pr12	GT_RBM3-N_ssDNA_R	AGTCATGCTCCTGTGAGTGG
Pr13	GT_GFP_F	GCGAGGAGCTGTTCACCGG
Pr14	GT_GFP_R	CCATGTGATCGCGCTTCTCGT
Pr15	Vector 1st half_Fw	CGACCGGTAGCGCTAGGATCCCCCCTGGGGAGAGAGGTCGGTGATTCG
Pr16	Vector 1st half_Rv	CTCTGCTAATCCTGTTACCAGTGGCTGC
Pr17	Vector 2nd half_Fw	GCAGCCACTGGTAACAGGATTAGCAGAG
Pr18	Vector 2nd half_Rv	ACCCAGCTTTCTTGTACAAAGTGGT
Pr19	HNRNPH1_Fw	GGGGATCCTAGCGCTACCGGTCGCCACCATGATGTTGGGCACGGAAGGTGGAG
Pr20	HNRNPH1_Rv	GACTTCCTCTGCCCTCACCGGATCCTGCAATGTTTGATTGAAAATCACTGGAGTTTTCC

Pr21	T2A-TagBFP_Fw	GGATCCGGTGAGGGCAGAGGAAGTC
Pr22	T2A-TagBFP_Rv	ACCACTTTGTACAAGAAAGCTGGGTTCAATTAAGCTTGTGCCCCAGTTTGCTAGGG
Pr23	qPCR_h18S_F	GTAACCCGTTGAACCCCATT
Pr24	qPCR_h18S_R	CCATCCAATCGGTAGTAGCG
Pr25	qPCR_hRBM3_e3_F	GGACCTATCTCTGAGGTGGTCG
Pr26	qPCR_hRBM3_e3_R	TTCATGGCTCTCATGGCAACTG
Pr27	qPCR_hRBM3_e4-5_F	TCAGATCCGTGTGGATCATG
Pr28	qPCR_hRBM3_e4-5_R	TAATACCTGCCACTCCCATAGCC
Pr29	qPCR_hRBM3_e3a_F	GTAGTAGAGTCTGCAGACCTGTGG
Pr30	qPCR_hRBM3_e3a_R	TCCTCTAAGCTGCCCGACTATC
Pr31	hRBM3_RTPCR_fwd_DK79	GTGGGAGGGCTCAACTTTAAC
Pr32	hRBM3_RTPCR_rev_DK80	CATGATCCACACGGATCTGAC
Pr33	hRBM3_RTPCR_fwd pair 2_DK81	AGGACCGGGAGACTCAGC
Pr34	hRBM3_RTPCR_rev pair 2_DK82	AGCCACCTCCTCTGGTTC
Pr35	hRBM3_Minigene_fwd_DK20F	GCTCGGATCCACTAGTCCAGTGT
Pr36	hRBM3_Minigene_rev_DK21R	GTTTAAACGGGCCCTCTAGACT
Pr37	hRBM3 qPCR ex2_fwd_DK30	TCAGCAGTTTCGGACCTATC
Pr38	hRBM3 qPCR ex3_rev_DK31	CATGCTCTGGGTTGGTGAAG
Pr39	GAPDH qpCR_fwd	CCAGAACATCATCCCTGCCT
Pr40	GAPDH qpCR_rev	GGTCAGGTCCACCACTGACA
Pr41	mRBM3 ex2 f _DK184	CTGCCATGTCGTCTG
Pr42	mRBM3 ex4 r_DK185	GATTTGGCGCCCATC
Pr43	HNRNPH qPCR_fwd	GGCCAATGATGGCTTTGTACG

Pr44	HNRNPH qPCR_rev	AGTCCACCGGCAATGTTATCC
Pr45	hRBM3 qPCR_premRNA_ex2_fwd	TGGGAGGGCTCAACTTTAAC
Pr46	hRBM3 qPCR_premRNA_in2_rev	TGCAAGGATCCCAGACTTTC
Pr47	qPCR_hHNRNPH1e11_F	GTGGTGCTTACGAACACAGATATG
Pr48	qPCR_hHNRNPH1e11_R	GCTGGACTGGTTTGACAAGC
Pr49	Pi7_PLVPBnewSeq	GTCTCGTGGGCTCGGAGATGTGTATAAGAGACAGACTCGGTGCCACTTTTTCAA
Pr50	Pi5_PLVPBnobarcode	TCGTCGGCAGCGTCAGATGTGTATAAGAGACAGTCTTGTGGAAAGGACGAAACA
iP7_1n	Q7005	CAAGCAGAAGACGGCATACGAGATATATTCACGTCTCGTGGGCTCGG
iP7_2n	Q7006	CAAGCAGAAGACGGCATACGAGATGCGCCTGTGTCTCGTGGGCTCGG
iP7_3n	Q7007	CAAGCAGAAGACGGCATACGAGATACTCTATGGTCTCGTGGGCTCGG
iP7_4n	Q7008	CAAGCAGAAGACGGCATACGAGATGTCTCGCAGTCTCGTGGGCTCGG
iP7_5n	Q7015	CAAGCAGAAGACGGCATACGAGATAGTAGAGAGTCTCGTGGGCTCGG
iP7_6n	Q7016	CAAGCAGAAGACGGCATACGAGATGACGAGAGGTCTCGTGGGCTCGG
iP7_7n	Q7017	CAAGCAGAAGACGGCATACGAGATAGACTTGGGTCTCGTGGGCTCGG
iP7_8n	Q7018	CAAGCAGAAGACGGCATACGAGATGAGTCCAAGTCTCGTGGGCTCGG
iP7_9n	Q7023	CAAGCAGAAGACGGCATACGAGATAATTCTGCGTCTCGTGGGCTCGG
iP7_10n	Q7024	CAAGCAGAAGACGGCATACGAGATGGCCTCATGTCTCGTGGGCTCGG
iP7_11n	Q7025	CAAGCAGAAGACGGCATACGAGATATCTTAGTGTCTCGTGGGCTCGG
iP7_12n	Q7026	CAAGCAGAAGACGGCATACGAGATGCTCCGACGTCTCGTGGGCTCGG
iP5_1n	Q5001	AATGATACGGCGACCACCGAGATCTACACAGCGCTAGTCGTCGGCAGCGTC
iP5_2n	Q5002	AATGATACGGCGACCACCGAGATCTACACGATATCGATCGTCGGCAGCGTC
iP5_3n	Q5003	AATGATACGGCGACCACCGAGATCTACACCGCAGACGTCGTCGGCAGCGTC
iP5_4n	Q5004	AATGATACGGCGACCACCGAGATCTACACTATGAGTATCGTCGGCAGCGTC

iP5_5n	Q5007	AATGATACGGCGACCACCGAGATCTACACACATAGCGTCGTCGGCAGCGTC
iP5_6n	Q5008	AATGATACGGCGACCACCGAGATCTACACGTGCGATATCGTCGGCAGCGTC
iP5_7n	Q5009	AATGATACGGCGACCACCGAGATCTACACCCAACAGATCGTCGGCAGCGTC
iP5_8n	Q5010	AATGATACGGCGACCACCGAGATCTACACTTGGTGAGTCGTCGGCAGCGTC

Appendix Table S2 RBM3 positive and negative regulator candidates identified by pooled CRISPR knockout screen

**Positive/Negative regulator**	**Rank**	**ID**	**Gene symbol**	**FDR**
Positive	1	ENSG00000169045	HNRNPH1	0.00165
Positive	2	ENSG00000102317	RBM3	0.00165
Positive	3	ENSG00000064419	TNPO3	0.00165
Positive	4	ENSG00000132424	PNISR	0.00165
Positive	5	ENSG00000086758	HUWE1	0.00165
Positive	6	ENSG00000104852	SNRNP70	0.00165
Positive	7	ENSG00000179950	PUF60	0.001856
Positive	8	ENSG00000118263	KLF7	0.001856
Positive	9	ENSG00000130699	TAF4	0.0022
Positive	10	ENSG00000130811	EIF3G	0.009406
Positive	11	ENSG00000177600	RPLP2	0.009451
Positive	12	ENSG00000106263	EIF3B	0.021325
Positive	13	ENSG00000141867	BRD4	0.021325
Positive	14	ENSG00000108848	LUC7L3	0.032885
Positive	15	ENSG00000151651	ADAM8	0.047855

Positive	16	ENSG00000134684	YARS	0.064356
Positive	17	ENSG00000120156	TEK	0.064356
Positive	18	ENSG00000131759	RARA	0.115787
Positive	19	ENSG00000165417	GTF2A1	0.16493
Positive	20	ENSG00000087095	NLK	0.200495
Positive	21	ENSG00000136485	DCAF7	0.200495
Positive	22	ENSG00000099139	PCSK5	0.200495
Positive	23	ENSG00000187758	ADH1A	0.251031
Positive	24	ENSG00000120833	SOCS2	0.251031
Positive	25	ENSG00000196361	ELAVL3	0.252277
Positive	26	ENSG00000171861	MRM3	0.255331
Positive	27	ENSG00000062038	CDH3	0.281628
Positive	28	ENSG00000135750	KCNK1	0.291396
Positive	29	ENSG00000174125	TLR1	0.291396
Positive	30	ENSG00000155545	MIER3	0.306106
Positive	31	ENSG00000100393	EP300	0.331219
Positive	32	ENSG00000182831	C16orf72	0.331219
Positive	33	ENSG00000170852	KBTBD2	0.358836
Positive	34	ENSG00000105327	BBC3	0.479663
Positive	35	ENSG00000172935	MRGPRF	0.479663
Positive	36	ENSG00000084623	EIF3I	0.479663
Positive	37	ENSG00000056097	ZFR	0.479663
Positive	38	ENSG00000126247	CAPNS1	0.54703

Positive	39	ENSG00000080618	CPB2	0.547728
Positive	40	ENSG00000156531	PHF6	0.606312
Positive	41	ENSG00000172530	BANP	0.613258
Positive	42	ENSG00000065427	KARS	0.69791
Positive	43	ENSG00000153046	CDYL	0.69791
Positive	44	ENSG00000174080	CTSF	0.69791
Positive	45	ENSG00000122741	DCAF10	0.69791
Positive	46	ENSG00000198380	GFPT1	0.704162
Positive	47	ENSG00000184254	ALDH1A3	0.704162
Positive	48	ENSG00000175197	DDIT3	0.704162
Positive	49	ENSG00000176896	TCEANC	0.704162
Positive	50	ENSG00000138741	TRPC3	0.704162
Positive	51	ENSG00000170091	NSG2	0.704162
Positive	52	ENSG00000028203	VEZT	0.704162
Positive	53	ENSG00000131469	RPL27	0.704162
Positive	54	ENSG00000160678	S100A1	0.704162
Positive	55	ENSG00000099957	P2RX6	0.709361
Positive	56	ENSG00000169230	PRELID1	0.751149
Positive	57	ENSG00000130032	PRRG3	0.798072
Positive	58	ENSG00000176946	THAP4	0.801087
Positive	59	ENSG00000144035	NAT8	0.801087
Positive	60	ENSG00000119714	GPR68	0.801087
Positive	61	ENSG00000133067	LGR6	0.801087

Positive	62	ENSG00000177963	RIC8A	0.824098
Positive	63	ENSG00000090861	AARS	0.82418
Positive	64	ENSG00000184156	KCNQ3	0.82418
Positive	65	ENSG00000213988	ZNF90	0.824476
Positive	66	ENSG00000125648	SLC25A23	0.824476
Positive	67	ENSG00000107331	ABCA2	0.824476
Positive	68	ENSG00000108443	RPS6KB1	0.824476
Positive	69	ENSG00000106615	RHEB	0.834912
Positive	70	ENSG00000181222	POLR2A	0.856223
Positive	71	ENSG00000115942	ORC2	0.85783
Positive	72	ENSG00000186487	MYT1L	0.88937
Positive	73	ENSG00000038002	AGA	0.898617
Negative	1	ENSG00000102241	HTATSF1	0.000381
Negative	2	ENSG00000119801	YPEL5	0.000381
Negative	3	ENSG00000108424	KPNB1	0.000381
Negative	4	ENSG00000162923	WDR26	0.000381
Negative	5	ENSG00000146457	WTAP	0.000381
Negative	6	ENSG00000176887	SOX11	0.000381
Negative	7	ENSG00000122692	SMU1	0.000381
Negative	8	ENSG00000122882	ECD	0.000381
Negative	9	ENSG00000129351	ILF3	0.000381
Negative	10	ENSG00000165699	TSC1	0.000381
Negative	11	ENSG00000100138	SNU13	0.000381

Negative	12	ENSG00000186591	UBE2H	0.000381
Negative	13	ENSG00000161547	SRSF2	0.000381
Negative	14	ENSG00000115875	SRSF7	0.00099
Negative	15	ENSG00000204569	PPP1R10	0.00099
Negative	16	ENSG00000171862	PTEN	0.001547
Negative	17	ENSG00000114209	PDCD10	0.002038
Negative	18	ENSG00000105618	PRPF31	0.002475
Negative	19	ENSG00000125952	MAX	0.002866
Negative	20	ENSG00000171634	BPTF	0.003536
Negative	21	ENSG00000169564	PCBP1	0.003536
Negative	22	ENSG00000135473	PAN2	0.00409
Negative	23	ENSG00000060339	CCAR1	0.00409
Negative	24	ENSG00000134884	ARGLU1	0.004332
Negative	25	ENSG00000163635	ATXN7	0.004379
Negative	26	ENSG00000135801	TAF5L	0.004379
Negative	27	ENSG00000115607	IL18RAP	0.004584
Negative	28	ENSG00000108883	EFTUD2	0.007603
Negative	29	ENSG00000139549	DHH	0.008706
Negative	30	ENSG00000086598	TMED2	0.009736
Negative	31	ENSG00000141568	FOXK2	0.011019
Negative	32	ENSG00000182591	KRTAP11-1	0.011551
Negative	33	ENSG00000172927	MYEOV	0.011551
Negative	34	ENSG00000163605	PPP4R2	0.011794

Negative	35	ENSG00000175550	DRAP1	0.01372
Negative	36	ENSG00000105393	BABAM1	0.015264
Negative	37	ENSG00000198258	UBL5	0.015763
Negative	38	ENSG00000101193	GID8	0.015763
Negative	39	ENSG00000134186	PRPF38B	0.016882
Negative	40	ENSG00000112110	MRPL18	0.019431
Negative	41	ENSG00000058262	SEC61A1	0.023304
Negative	42	ENSG00000174547	MRPL11	0.024163
Negative	43	ENSG00000141526	SLC16A3	0.026665
Negative	44	ENSG00000156471	PTDSS1	0.026665
Negative	45	ENSG00000173011	TADA2B	0.027741
Negative	46	ENSG00000183091	NEB	0.027741
Negative	47	ENSG00000109189	USP46	0.027741
Negative	48	ENSG00000100427	MLC1	0.027741
Negative	49	ENSG00000229549	TSPY8	0.027741
Negative	50	ENSG00000164151	ICE1	0.027741
Negative	51	ENSG00000162300	ZFPL1	0.027741
Negative	52	ENSG00000143569	UBAP2L	0.027741
Negative	53	ENSG00000167088	SNRPD1	0.027741
Negative	54	ENSG00000129562	DAD1	0.028144
Negative	55	ENSG00000172409	CLP1	0.029433
Negative	56	ENSG00000198754	OXCT2	0.031206
Negative	57	ENSG00000111859	NEDD9	0.033959

Negative	58	ENSG00000136875	PRPF4	0.034568
Negative	59	ENSG00000008118	CAMK1G	0.035891
Negative	60	ENSG00000160993	ALKBH4	0.035891
Negative	61	ENSG00000185963	BICD2	0.036114
Negative	62	ENSG00000011083	SLC6A7	0.037325
Negative	63	ENSG00000196655	TRAPPC4	0.037325
Negative	64	ENSG00000172967	XKR3	0.038029
Negative	65	ENSG00000156171	DRAM2	0.038029
Negative	66	ENSG00000120948	TARDBP	0.038029
Negative	67	ENSG00000129514	FOXA1	0.040607
Negative	68	ENSG00000140534	TICRR	0.040607
Negative	69	ENSG00000074181	NOTCH3	0.040607
Negative	70	ENSG00000158966	CACHD1	0.040607
Negative	71	ENSG00000112701	SENP6	0.040607
Negative	72	ENSG00000136709	WDR33	0.040607
Negative	73	ENSG00000166189	HPS6	0.040607
Negative	74	ENSG00000102554	KLF5	0.040607
Negative	75	ENSG00000172465	TCEAL1	0.043708
Negative	76	ENSG00000134982	APC	0.043708
Negative	77	ENSG00000185278	ZBTB37	0.044109
Negative	78	ENSG00000105662	CRTC1	0.044109
Negative	79	ENSG00000092201	SUPT16H	0.044109
Negative	80	ENSG00000125818	PSMF1	0.044109

Negative	81	ENSG00000103647	CORO2B	0.044109
Negative	82	ENSG00000196236	XPNPEP3	0.044109
Negative	83	ENSG00000057704	TMCC3	0.044109
Negative	84	ENSG00000182872	RBM10	0.044109
Negative	85	ENSG00000143379	SETDB1	0.044109
Negative	86	ENSG00000176909	MAMSTR	0.044109
Negative	87	ENSG00000157322	CLEC18A	0.044109
Negative	88	ENSG00000182325	FBXL6	0.044109
Negative	89	ENSG00000104921	FCER2	0.044109
Negative	90	ENSG00000189253	TRIM64B	0.04648
Negative	91	ENSG00000159140	SON	0.047492
Negative	92	ENSG00000176020	AMIGO3	0.048913
Negative	93	ENSG00000007866	TEAD3	0.04987
Negative	94	ENSG00000065911	MTHFD2	0.04987
Negative	95	ENSG00000131899	LLGL1	0.04987
Negative	96	ENSG00000095321	CRAT	0.051337
Negative	97	ENSG00000170255	MRGPRX1	0.051337
Negative	98	ENSG00000157110	RBPMS	0.051337
Negative	99	ENSG00000160226	C21orf2	0.051337
Negative	100	ENSG00000161940	BCL6B	0.051337
Negative	101	ENSG00000185352	HS6ST3	0.055632
Negative	102	ENSG00000254709	IGLL5	0.057416
Negative	103	ENSG00000176476	SGF29	0.062722

Negative	104	ENSG00000164048	ZNF589	0.064074
Negative	105	ENSG00000143995	MEIS1	0.064074
Negative	106	ENSG00000204070	SYS1	0.066365
Negative	107	ENSG00000112983	BRD8	0.069446
Negative	108	ENSG00000102265	TIMP1	0.069536
Negative	109	ENSG00000110651	CD81	0.070297
Negative	110	ENSG00000101191	DIDO1	0.070297
Negative	111	ENSG00000145216	FIP1L1	0.070297
Negative	112	ENSG00000110063	DCPS	0.070297
Negative	113	ENSG00000164944	VIRMA	0.070297
Negative	114	ENSG00000259956	RBM15B	0.070297
Negative	115	ENSG00000179564	LSMEM2	0.070297
Negative	116	ENSG00000101104	PABPC1L	0.074091
Negative	117	ENSG00000126226	PCID2	0.074091
Negative	118	ENSG00000160963	COL26A1	0.074091
Negative	119	ENSG00000176978	DPP7	0.074091
Negative	120	ENSG00000186105	LRRC70	0.074413
Negative	121	ENSG00000125449	ARMC7	0.074413
Negative	122	ENSG00000069869	NEDD4	0.074413
Negative	123	ENSG00000168906	MAT2A	0.074413
Negative	124	ENSG00000204228	HSD17B8	0.074413
Negative	125	ENSG00000244405	ETV5	0.074413
Negative	126	ENSG00000110321	EIF4G2	0.074413

Negative	127	ENSG00000175198	PCCA	0.074413
Negative	128	ENSG00000185272	RBM11	0.075688
Negative	129	ENSG00000153113	CAST	0.07702
Negative	130	ENSG00000160310	PRMT2	0.078945
Negative	131	ENSG00000187189	TSPYL4	0.078945
Negative	132	ENSG00000075856	SART3	0.078945
Negative	133	ENSG00000106245	BUD31	0.079318
Negative	134	ENSG00000170613	FAM71B	0.079318
Negative	135	ENSG00000189319	FAM53B	0.079318
Negative	136	ENSG00000079102	RUNX1T1	0.079528
Negative	137	ENSG00000117461	PIK3R3	0.079528
Negative	138	ENSG00000185176	AQP12B	0.079528
Negative	139	ENSG00000184916	JAG2	0.079528
Negative	140	ENSG00000140691	ARMC5	0.079562
Negative	141	ENSG00000159217	IGF2BP1	0.079581
Negative	142	ENSG00000128815	WDFY4	0.079581
Negative	143	ENSG00000105829	BET1	0.079581
Negative	144	ENSG00000128951	DUT	0.079581
Negative	145	ENSG00000184678	HIST2H2BE	0.079581
Negative	146	ENSG00000089818	NECAP1	0.079581
Negative	147	ENSG00000149260	CAPN5	0.08376
Negative	148	ENSG00000103326	CAPN15	0.08376
Negative	149	ENSG00000213937	CLDN9	0.08376

Negative	150	ENSG00000187555	USP7	0.083765
Negative	151	ENSG00000047410	TPR	0.083765
Negative	152	ENSG00000135363	LMO2	0.083998
Negative	153	ENSG00000114784	EIF1B	0.083998
Negative	154	ENSG00000126752	SSX1	0.083998
Negative	155	ENSG00000103510	KAT8	0.084286
Negative	156	ENSG00000029364	SLC39A9	0.086983
Negative	157	ENSG00000134809	TIMM10	0.088699
Negative	158	ENSG00000236334	PPIAL4G	0.089139
Negative	159	ENSG00000135045	C9orf40	0.089139
Negative	160	ENSG00000198133	TMEM229B	0.089139
Negative	161	ENSG00000215704	CELA2B	0.089139
Negative	162	ENSG00000061987	MON2	0.089139
Negative	163	ENSG00000177732	SOX12	0.089139
Negative	164	ENSG00000186086	NBPF6	0.090226
Negative	165	ENSG00000104731	KLHDC4	0.092185
Negative	166	ENSG00000172888	ZNF621	0.092185
Negative	167	ENSG00000011485	PPP5C	0.092185
Negative	168	ENSG00000109101	FOXN1	0.092185
Negative	169	ENSG00000134453	RBM17	0.092185
Negative	170	ENSG00000177519	RPRM	0.092691
Negative	171	ENSG00000100218	RSPH14	0.092901
Negative	172	ENSG00000196890	HIST3H2BB	0.093858

Negative	173	ENSG00000136541	ERMN	0.095433
Negative	174	ENSG00000161800	RACGAP1	0.099409
Negative	175	ENSG00000101412	E2F1	0.099409
Negative	176	ENSG00000111906	HDDC2	0.099409
Negative	177	ENSG00000196666	FAM180B	0.099409
Negative	178	ENSG00000164091	WDR82	0.099409
Negative	179	ENSG00000184863	RBM33	0.099409
Negative	180	ENSG00000100320	RBFOX2	0.099409
Negative	181	ENSG00000168484	SFTPC	0.099409
Negative	182	ENSG00000143493	INTS7	0.099409
Negative	183	ENSG00000235194	PPP1R3E	0.099409
Negative	184	ENSG00000082438	COBLL1	0.099409
Negative	185	ENSG00000137601	NEK1	0.099409
Negative	186	ENSG00000176571	CNBD1	0.099409

Appendix Table S3 A complete list of significant Gene Ontology (GO) terms of RBM3 positive and negative regulators

Positive/Negative regulator	GO	Category	Description	LogP	Hits
**Positive**	**GO:0022618**	**GO Biological Processes**	**ribonucleoprotein complex assembly**	**-5.602**	**EIF3B|EIF3G|PUF60|LUC7L3**
Positive	GO:0071826	GO Biological Processes	ribonucleoprotein complex subunit organization	-5.540	EIF3B|EIF3G|PUF60|LUC7L3
Positive	R-HSA-72689	Reactome Gene Sets	Formation of a pool of free 40S subunits	-4.890	RPLP2|EIF3B|EIF3G
Positive	R-HSA-156827	Reactome Gene Sets	L13a-mediated translational silencing of Ceruloplasmin expression	-4.767	RPLP2|EIF3B|EIF3G

Positive	R-HSA-72706	Reactome Gene Sets	GTP hydrolysis and joining of the 60S ribosomal subunit	-4.756	RPLP2|EIF3B|EIF3G
Positive	R-HSA-72613	Reactome Gene Sets	Eukaryotic Translation Initiation	-4.677	RPLP2|EIF3B|EIF3G
Positive	R-HSA-72737	Reactome Gene Sets	Cap-dependent Translation Initiation	-4.677	RPLP2|EIF3B|EIF3G
Positive	GO:0002181	GO Biological Processes	cytoplasmic translation	-4.645	RPLP2|EIF3B|EIF3G
Positive	GO:0022613	GO Biological Processes	ribonucleoprotein complex biogenesis	-4.338	EIF3B|EIF3G|PUF60|LUC7L3
Positive	R-HSA-72766	Reactome Gene Sets	Translation	-3.526	RPLP2|EIF3B|EIF3G
Positive	GO:0006412	GO Biological Processes	translation	-3.225	RPLP2|EIF3B|EIF3G
Positive	GO:0043043	GO Biological Processes	peptide biosynthetic process	-3.136	RPLP2|EIF3B|EIF3G
Positive	GO:0043604	GO Biological Processes	amide biosynthetic process	-2.816	RPLP2|EIF3B|EIF3G
Positive	GO:0006518	GO Biological Processes	peptide metabolic process	-2.773	RPLP2|EIF3B|EIF3G
Positive	GO:0034645	GO Biological Processes	cellular macromolecule biosynthetic process	-2.346	RPLP2|EIF3B|EIF3G
Positive	GO:0043603	GO Biological Processes	cellular amide metabolic process	-2.278	RPLP2|EIF3B|EIF3G
Positive	GO:0000377	GO Biological Processes	RNA splicing, via transesterification reactions with bulged adenosine as nucleophile	-5.247	HNRNPH1|SNRNP70|PUF60|LUC7L3
**Positive**	**GO:0000398**	**GO Biological Processes**	**mRNA splicing, via spliceosome**	**-5.247**	**HNRNPH1|SNRNP70|PUF60|LUC7L3**
Positive	GO:0000375	GO Biological Processes	RNA splicing, via transesterification reactions	-5.222	HNRNPH1|SNRNP70|PUF60|LUC7L3
Positive	GO:0016071	GO Biological Processes	mRNA metabolic process	-5.220	HNRNPH1|SNRNP70|TAF4|PUF60|LUC7L3
Positive	GO:0008380	GO Biological Processes	RNA splicing	-4.620	HNRNPH1|SNRNP70|PUF60|LUC7L3
Positive	CORUM:351	CORUM	Spliceosome	-4.457	SNRNP70|PUF60|LUC7L3
Positive	GO:0006397	GO Biological Processes	mRNA processing	-4.319	HNRNPH1|SNRNP70|PUF60|LUC7L3
Positive	GO:0043484	GO Biological Processes	regulation of RNA splicing	-4.156	HNRNPH1|SNRNP70|PUF60
Positive	R-HSA-72163	Reactome Gene Sets	mRNA Splicing - Major Pathway	-4.113	HNRNPH1|SNRNP70|PUF60
Positive	R-HSA-72172	Reactome Gene Sets	mRNA Splicing	-4.058	HNRNPH1|SNRNP70|PUF60
Positive	R-HSA-72203	Reactome Gene Sets	Processing of Capped Intron-Containing Pre-mRNA	-3.745	HNRNPH1|SNRNP70|PUF60
Positive	R-HSA-8953854	Reactome Gene Sets	Metabolism of RNA	-3.686	HNRNPH1|RPLP2|SNRNP70|PUF60
Positive	GO:0042176	GO Biological Processes	regulation of protein catabolic process	-3.133	ADAM8|SNRNP70|HUWE1
Positive	GO:0051223	GO Biological Processes	regulation of protein transport	-2.856	ADAM8|KLF7|HUWE1
Positive	GO:0070201	GO Biological Processes	regulation of establishment of protein localization	-2.795	ADAM8|KLF7|HUWE1

Negative	GO:0006397	GO Biological Processes	mRNA processing	-16.709	SNU13|SRSF2|SRSF7|SNRPD1|SON|RBM10|PRPF4|EFTUD2|WTAP|PAN2|CLP1|ECD|TARDBP|PRPF31|HTATSF1|PRPF38B|SMU1|WDR33|UBL5|PPP4R2
**Negative**	**GO:0008380**	**GO Biological Processes**	**RNA splicing**	**-15.577**	**SNU13|SRSF2|SRSF7|SNRPD1|SON|RBM10|PRPF4|EFTUD2|WTAP|CLP1|ECD|TARDBP|PRPF31|HTATSF1|PRPF38B|SMU1|UBL5|PPP4R2**
Negative	GO:0016071	GO Biological Processes	mRNA metabolic process	-14.312	SNU13|SRSF2|SRSF7|SNRPD1|SON|RBM10|PRPF4|EFTUD2|WTAP|PAN2|CLP1|ECD|TARDBP|PRPF31|HTATSF1|PRPF38B|SMU1|WDR33|UBL5|PPP4R2
Negative	R-HSA-72163	Reactome Gene Sets	mRNA Splicing - Major Pathway	-10.798	SNU13|PCBP1|SRSF2|SRSF7|SNRPD1|PRPF4|EFTUD2|CLP1|PRPF31|WDR33|CCAR1
Negative	R-HSA-72203	Reactome Gene Sets	Processing of Capped Intron-Containing Pre-mRNA	-10.728	SNU13|PCBP1|SRSF2|SRSF7|SNRPD1|PRPF4|EFTUD2|WTAP|CLP1|PRPF31|WDR33|CCAR1
Negative	R-HSA-72172	Reactome Gene Sets	mRNA Splicing	-10.598	SNU13|PCBP1|SRSF2|SRSF7|SNRPD1|PRPF4|EFTUD2|CLP1|PRPF31|WDR33|CCAR1
Negative	CORUM:351	CORUM	Spliceosome	-10.590	SNU13|SRSF2|SRSF7|SNRPD1|PRPF4|EFTUD2|WTAP|PRPF31|HTATSF1|SMU1
Negative	GO:0000377	GO Biological Processes	RNA splicing, via transesterification reactions with bulged adenosine as nucleophile	-9.053	SNU13|SRSF2|SRSF7|SNRPD1|RBM10|PRPF4|EFTUD2|PRPF31|HTATSF1|SMU1|UBL5
Negative	GO:0000398	GO Biological Processes	mRNA splicing, via spliceosome	-9.053	SNU13|SRSF2|SRSF7|SNRPD1|RBM10|PRPF4|EFTUD2|PRPF31|HTATSF1|SMU1|UBL5
Negative	hsa03040	KEGG Pathway	Spliceosome	-9.006	SNU13|PCBP1|SRSF2|SRSF7|SNRPD1|PRPF4|EFTUD2|PRPF31|PRPF38B
Negative	GO:0000375	GO Biological Processes	RNA splicing, via transesterification reactions	-8.985	SNU13|SRSF2|SRSF7|SNRPD1|RBM10|PRPF4|EFTUD2|PRPF31|HTATSF1|SMU1|UBL5
Negative	R-HSA-8953854	Reactome Gene Sets	Metabolism of RNA	-7.589	SNU13|PCBP1|SRSF2|SRSF7|SNRPD1|PRPF4|EFTUD2|PSMF1|WTAP|PAN2|CLP1|PRPF31|WDR33|CCAR1
Negative	WP411	WikiPathways	mRNA processing	-6.659	SNU13|SRSF2|SRSF7|SNRPD1|PRPF4|EFTUD2|CLP1
Negative	R-HSA-72165	Reactome Gene Sets	mRNA Splicing - Minor Pathway	-6.193	SNU13|SRSF2|SRSF7|SNRPD1|EFTUD2
Negative	GO:0022618	GO Biological Processes	ribonucleoprotein complex assembly	-2.298	SNU13|SNRPD1|CLP1|PRPF31
Negative	GO:0071826	GO Biological Processes	ribonucleoprotein complex subunit organization	-2.243	SNU13|SNRPD1|CLP1|PRPF31
Negative	GO:0043484	GO Biological Processes	regulation of RNA splicing	-8.273	ATXN7|SRSF2|SRSF7|SON|RBM10|WTAP|TAF5L|SMU1|TADA2B
Negative	GO:0048024	GO Biological Processes	regulation of mRNA splicing, via spliceosome	-5.961	SRSF2|SRSF7|SON|RBM10|WTAP|SMU1
Negative	GO:1903311	GO Biological Processes	regulation of mRNA metabolic process	-5.251	SRSF2|SRSF7|SON|RBM10|WTAP|PAN2|TARDBP|SMU1
Negative	GO:0050684	GO Biological Processes	regulation of mRNA processing	-5.223	SRSF2|SRSF7|SON|RBM10|WTAP|SMU1
Negative	GO:0000381	GO Biological Processes	regulation of alternative mRNA splicing, via spliceosome	-4.407	SRSF2|RBM10|WTAP|SMU1
Negative	WP4352	WikiPathways	Ciliary landscape	-5.210	APC|UBE2H|EFTUD2|YPEL5|GID8|XPNPEP3|WDR26

**Negative**	**GO:0043161**	**GO Biological Processes**	**proteasome-mediated ubiquitin-dependent protein catabolic process**	**-3.170**	**APC|UBE2H|PSMF1|FBXL6|GID8|WDR26**
Negative	GO:0010498	GO Biological Processes	proteasomal protein catabolic process	-2.971	APC|UBE2H|PSMF1|FBXL6|GID8|WDR26
Negative	GO:0006511	GO Biological Processes	ubiquitin-dependent protein catabolic process	-2.719	APC|UBE2H|PSMF1|FBXL6|GID8|USP46|WDR26
Negative	GO:0019941	GO Biological Processes	modification-dependent protein catabolic process	-2.676	APC|UBE2H|PSMF1|FBXL6|GID8|USP46|WDR26
Negative	GO:0043632	GO Biological Processes	modification-dependent macromolecule catabolic process	-2.629	APC|UBE2H|PSMF1|FBXL6|GID8|USP46|WDR26
Negative	GO:0051603	GO Biological Processes	proteolysis involved in cellular protein catabolic process	-2.434	APC|UBE2H|PSMF1|FBXL6|GID8|USP46|WDR26
Negative	GO:0044257	GO Biological Processes	cellular protein catabolic process	-2.328	APC|UBE2H|PSMF1|FBXL6|GID8|USP46|WDR26
Negative	GO:0030163	GO Biological Processes	protein catabolic process	-2.142	APC|UBE2H|PSMF1|FBXL6|GID8|USP46|WDR26
Negative	GO:0016578	GO Biological Processes	histone deubiquitination	-4.946	ATXN7|TAF5L|BABAM1|TADA2B
Negative	GO:0070646	GO Biological Processes	protein modification by small protein removal	-4.820	ATXN7|SENP6|TAF5L|BABAM1|USP46|TADA2B
Negative	GO:0080135	GO Biological Processes	regulation of cellular response to stress	-4.762	PPP1R10|PTEN|ATXN7|TSC1|CORO2B|TMED2|PDCD10|TAF5L|BABAM1|TADA2B|PPP4R2
Negative	CORUM:6664	CORUM	STAGA complex, SPT3-linked	-4.550	ATXN7|TAF5L|TADA2B
**Negative**	**R-HSA-5688426**	**Reactome Gene Sets**	**Deubiquitination**	**-4.350**	**APC|FOXK2|PTEN|ATXN7|PSMF1|BABAM1|TADA2B**
Negative	GO:0035521	GO Biological Processes	monoubiquitinated histone deubiquitination	-3.984	ATXN7|TAF5L|TADA2B
Negative	GO:0035522	GO Biological Processes	monoubiquitinated histone H2A deubiquitination	-3.984	ATXN7|TAF5L|TADA2B
Negative	GO:0016579	GO Biological Processes	protein deubiquitination	-3.955	ATXN7|TAF5L|BABAM1|USP46|TADA2B
Negative	GO:0035520	GO Biological Processes	monoubiquitinated protein deubiquitination	-3.774	ATXN7|TAF5L|TADA2B
Negative	GO:2001020	GO Biological Processes	regulation of response to DNA damage stimulus	-3.368	PPP1R10|ATXN7|TAF5L|BABAM1|TADA2B|PPP4R2
Negative	GO:0006282	GO Biological Processes	regulation of DNA repair	-3.267	ATXN7|TAF5L|BABAM1|TADA2B|PPP4R2
Negative	GO:0043966	GO Biological Processes	histone H3 acetylation	-3.022	ATXN7|TAF5L|TADA2B
Negative	GO:0051052	GO Biological Processes	regulation of DNA metabolic process	-2.809	PPP1R10|ATXN7|TAF5L|BABAM1|TICRR|TADA2B|PPP4R2
Negative	GO:0016570	GO Biological Processes	histone modification	-2.775	ATXN7|SETDB1|TAF5L|BABAM1|TADA2B|PPP4R2
Negative	GO:0018205	GO Biological Processes	peptidyl-lysine modification	-2.545	ATXN7|SETDB1|SENP6|TAF5L|TADA2B
Negative	R-HSA-5689880	Reactome Gene Sets	Ub-specific processing proteases	-2.284	PTEN|ATXN7|PSMF1|TADA2B
Negative	GO:0016573	GO Biological Processes	histone acetylation	-2.178	ATXN7|TAF5L|TADA2B
Negative	GO:0018393	GO Biological Processes	internal peptidyl-lysine acetylation	-2.120	ATXN7|TAF5L|TADA2B

Negative	GO:0006475	GO Biological Processes	internal protein amino acid acetylation	-2.101	ATXN7|TAF5L|TADA2B
Negative	GO:0018394	GO Biological Processes	peptidyl-lysine acetylation	-2.092	ATXN7|TAF5L|TADA2B
Negative	R-HSA-72187	Reactome Gene Sets	mRNA 3'-end processing	-4.436	SRSF2|SRSF7|CLP1|WDR33
Negative	R-HSA-73856	Reactome Gene Sets	RNA Polymerase II Transcription Termination	-4.193	SRSF2|SRSF7|CLP1|WDR33
Negative	R-HSA-72202	Reactome Gene Sets	Transport of Mature Transcript to Cytoplasm	-2.622	SRSF2|SRSF7|WDR33
Negative	hsa03015	KEGG Pathway	mRNA surveillance pathway	-2.445	CLP1|TARDBP|WDR33
Negative	GO:0050657	GO Biological Processes	nucleic acid transport	-3.823	KPNB1|SRSF7|TSC1|BICD2|MRPL18
Negative	GO:0050658	GO Biological Processes	RNA transport	-3.823	KPNB1|SRSF7|TSC1|BICD2|MRPL18
Negative	GO:0051236	GO Biological Processes	establishment of RNA localization	-3.798	KPNB1|SRSF7|TSC1|BICD2|MRPL18
Negative	GO:0006403	GO Biological Processes	RNA localization	-3.595	KPNB1|SRSF7|TSC1|BICD2|MRPL18
Negative	GO:0015931	GO Biological Processes	nucleobase-containing compound transport	-3.221	KPNB1|SRSF7|TSC1|BICD2|MRPL18
Negative	GO:0032388	GO Biological Processes	positive regulation of intracellular transport	-3.383	PDCD10|MLC1|ICE1|TARDBP|UBL5
Negative	GO:0090316	GO Biological Processes	positive regulation of intracellular protein transport	-2.820	PDCD10|ICE1|TARDBP|UBL5
Negative	GO:1903829	GO Biological Processes	positive regulation of protein localization	-2.530	APC|CORO2B|PDCD10|ICE1|TARDBP|UBL5
Negative	GO:1904951	GO Biological Processes	positive regulation of establishment of protein localization	-2.527	CORO2B|PDCD10|ICE1|TARDBP|UBL5
Negative	GO:0032386	GO Biological Processes	regulation of intracellular transport	-2.395	PDCD10|MLC1|ICE1|TARDBP|UBL5
Negative	GO:0033157	GO Biological Processes	regulation of intracellular protein transport	-2.256	PDCD10|ICE1|TARDBP|UBL5
Negative	GO:0070201	GO Biological Processes	regulation of establishment of protein localization	-2.218	LLGL1|CORO2B|PDCD10|ICE1|TARDBP|UBL5
Negative	GO:0060341	GO Biological Processes	regulation of cellular localization	-2.073	APC|PTEN|PDCD10|MLC1|ICE1|TARDBP|UBL5
Negative	GO:0001655	GO Biological Processes	urogenital system development	-3.176	FOXA1|NOTCH3|PTEN|SOX11|TSC1|SEC61A1
Negative	GO:0035239	GO Biological Processes	tube morphogenesis	-2.929	KLF5|FOXA1|NOTCH3|PTEN|SOX11|TSC1|TMED2|PDCD10
Negative	GO:0021915	GO Biological Processes	neural tube development	-2.851	FOXA1|SOX11|TSC1|TMED2
Negative	GO:0002065	GO Biological Processes	columnar/cuboidal epithelial cell differentiation	-2.682	KLF5|FOXA1|SOX11
Negative	GO:0060537	GO Biological Processes	muscle tissue development	-2.527	KLF5|NEB|PTEN|SOX11|TSC1
Negative	GO:0001841	GO Biological Processes	neural tube formation	-2.372	SOX11|TSC1|TMED2
Negative	GO:0001838	GO Biological Processes	embryonic epithelial tube formation	-2.168	SOX11|TSC1|TMED2
Negative	GO:0072175	GO Biological Processes	epithelial tube formation	-2.092	SOX11|TSC1|TMED2

Negative	GO:0006325	GO Biological Processes	chromatin organization	-3.152	BPTF|FOXA1|SETDB1|SUPT16H|BABAM1|ALKBH4|TADA2B|TSPY8
Negative	GO:0048608	GO Biological Processes	reproductive structure development	-2.653	BPTF|FOXA1|PTEN|TMED2|DHH|TSPY8
Negative	GO:0061458	GO Biological Processes	reproductive system development	-2.638	BPTF|FOXA1|PTEN|TMED2|DHH|TSPY8
Negative	GO:0006338	GO Biological Processes	chromatin remodeling	-2.601	BPTF|FOXA1|SUPT16H|TADA2B|TSPY8
Negative	M145	Canonical Pathways	PID P53 DOWNSTREAM PATHWAY	-3.028	APC|FOXA1|PTEN|TADA2B
Negative	GO:0045165	GO Biological Processes	cell fate commitment	-2.974	APC|FOXA1|NOTCH3|TEAD3|DHH
Negative	WP2853	WikiPathways	Endoderm differentiation	-2.926	APC|BPTF|FOXA1|CRTC1
Negative	GO:0001708	GO Biological Processes	cell fate specification	-2.622	APC|FOXA1|DHH
Negative	GO:0043068	GO Biological Processes	positive regulation of programmed cell death	-2.129	APC|FCER2|FOXA1|PTEN|RBM10|CCAR1
Negative	GO:0051091	GO Biological Processes	positive regulation of DNA-binding transcription factor activity	-2.005	FOXA1|PTEN|IL18RAP|CRTC1
Negative	GO:0051640	GO Biological Processes	organelle localization	-2.986	KPNB1|LLGL1|TMED2|PDCD10|BICD2|TRAPPC4|HPS6
Negative	GO:0051656	GO Biological Processes	establishment of organelle localization	-2.923	KPNB1|LLGL1|TMED2|PDCD10|BICD2|TRAPPC4
Negative	GO:0007346	GO Biological Processes	regulation of mitotic cell cycle	-2.903	APC|FOXA1|PPP1R10|PTEN|ECD|BABAM1|TICRR
Negative	GO:0010948	GO Biological Processes	negative regulation of cell cycle process	-2.665	APC|PPP1R10|PTEN|BABAM1|TICRR
Negative	GO:1901991	GO Biological Processes	negative regulation of mitotic cell cycle phase transition	-2.611	APC|PTEN|BABAM1|TICRR
Negative	GO:0000278	GO Biological Processes	mitotic cell cycle	-2.519	APC|KPNB1|SRSF2|SON|FBXL6|BABAM1|TICRR
Negative	GO:1901987	GO Biological Processes	regulation of cell cycle phase transition	-2.502	APC|PPP1R10|PTEN|ECD|BABAM1|TICRR
Negative	GO:1901990	GO Biological Processes	regulation of mitotic cell cycle phase transition	-2.273	APC|PTEN|ECD|BABAM1|TICRR
Negative	GO:1903047	GO Biological Processes	mitotic cell cycle process	-2.241	APC|KPNB1|SON|FBXL6|BABAM1|TICRR
Negative	GO:0045930	GO Biological Processes	negative regulation of mitotic cell cycle	-2.236	APC|PTEN|BABAM1|TICRR
Negative	GO:0045786	GO Biological Processes	negative regulation of cell cycle	-2.174	APC|PPP1R10|PTEN|BABAM1|TICRR
Negative	GO:1901988	GO Biological Processes	negative regulation of cell cycle phase transition	-2.165	APC|PTEN|BABAM1|TICRR
Negative	GO:0007093	GO Biological Processes	mitotic cell cycle checkpoint signaling	-2.158	APC|BABAM1|TICRR
Negative	GO:0010564	GO Biological Processes	regulation of cell cycle process	-2.070	APC|PPP1R10|PTEN|ECD|SENP6|BABAM1|TICRR
Negative	GO:0070507	GO Biological Processes	regulation of microtubule cytoskeleton organization	-2.840	APC|ATXN7|BICD2|SENP6
Negative	GO:0051493	GO Biological Processes	regulation of cytoskeleton organization	-2.837	APC|NEB|ATXN7|TSC1|CORO2B|BICD2|SENP6

Negative	GO:0032886	GO Biological Processes	regulation of microtubule-based process	-2.091	APC|ATXN7|BICD2|SENP6
Negative	GO:0033044	GO Biological Processes	regulation of chromosome organization	-2.091	APC|PPP1R10|SETDB1|SENP6
Negative	GO:0031330	GO Biological Processes	negative regulation of cellular catabolic process	-2.788	FOXK2|TSC1|RBM10|PSMF1|TARDBP
Negative	GO:1903312	GO Biological Processes	negative regulation of mRNA metabolic process	-2.483	SRSF7|RBM10|TARDBP
Negative	GO:0009895	GO Biological Processes	negative regulation of catabolic process	-2.417	FOXK2|TSC1|RBM10|PSMF1|TARDBP
Negative	WP4949	WikiPathways	16p11.2 proximal deletion syndrome	-2.746	BPTF|PTEN|PPP4R2
Negative	GO:0031647	GO Biological Processes	regulation of protein stability	-2.457	DAD1|PTEN|TSC1|PDCD10|TARDBP
Negative	GO:0001933	GO Biological Processes	negative regulation of protein phosphorylation	-2.351	APC|PTEN|TMED2|TARDBP|PPP4R2
Negative	GO:0010256	GO Biological Processes	endomembrane system organization	-2.156	PTEN|TMED2|PDCD10|TARDBP|SEC61A1|HPS6
Negative	GO:0042326	GO Biological Processes	negative regulation of phosphorylation	-2.141	APC|PTEN|TMED2|TARDBP|PPP4R2
Negative	WP5087	WikiPathways	Malignant pleural mesothelioma	-2.535	APC|MAX|PTEN|TEAD3|TSC1|SETDB1
Negative	hsa05165	KEGG Pathway	Human papillomavirus infection	-2.400	APC|LLGL1|NOTCH3|PTEN|TSC1
Negative	GO:0042552	GO Biological Processes	myelination	-2.303	PTEN|TSC1|DHH
Negative	GO:0010810	GO Biological Processes	regulation of cell-substrate adhesion	-2.298	NEDD9|PTEN|TSC1|CORO2B
Negative	GO:0007272	GO Biological Processes	ensheathment of neurons	-2.281	PTEN|TSC1|DHH
Negative	GO:0008366	GO Biological Processes	axon ensheathment	-2.281	PTEN|TSC1|DHH
Negative	GO:0030534	GO Biological Processes	adult behavior	-2.039	PTEN|TSC1|USP46
Negative	GO:0010977	GO Biological Processes	negative regulation of neuron projection development	-2.030	PTEN|TSC1|AMIGO3
Negative	GO:1990830	GO Biological Processes	cellular response to leukemia inhibitory factor	-2.420	KLF5|SRSF7|PDCD10
Negative	GO:1990823	GO Biological Processes	response to leukemia inhibitory factor	-2.408	KLF5|SRSF7|PDCD10
Negative	GO:0061640	GO Biological Processes	cytoskeleton-dependent cytokinesis	-2.337	APC|SON|ALKBH4
Negative	GO:0000910	GO Biological Processes	cytokinesis	-2.314	APC|SON|ALKBH4
Negative	GO:0051301	GO Biological Processes	cell division	-2.286	APC|NEDD9|SON|TEAD3|BABAM1|ALKBH4
Negative	GO:0044089	GO Biological Processes	positive regulation of cellular component biogenesis	-2.332	APC|TSC1|SETDB1|PAN2|ICE1|AMIGO3
Negative	GO:0017148	GO Biological Processes	negative regulation of translation	-2.140	ILF3|TSC1|PAN2|TARDBP
Negative	GO:0042752	GO Biological Processes	regulation of circadian rhythm	-2.208	CRTC1|TARDBP|FBXL6
Negative	R-HSA-157118	Reactome Gene Sets	Signaling by NOTCH	-2.177	FCER2|NOTCH3|PSMF1|TMED2

Negative	GO:0006366	GO Biological Processes	transcription by RNA polymerase II	-2.127	DRAP1|SUPT16H|ICE1|TAF5L

Appendix Table S4 sgRNA sequences used in arrayed target validation

**Gene ID**	**Gene symbol**	**Sequence (5' -> 3')**
NonTargetingGuide001	Non-Targeting	CTTATAGCGGCCTTAGCTCA
NonTargetingGuide002	Non-Targeting	CAACCTTATAGCCCCGGACT
NonTargetingGuide003	Non-Targeting	CATGTAGCAGAAGGCGGCTA
ENSG00000169045	HNRNPH1	TTGGCCGTGTCAGGACTATT
ENSG00000169045	HNRNPH1	AATGTCTGATCACAGATACG
ENSG00000169045	HNRNPH1	AGCAAACCAGTCCAGCTACG
ENSG00000102317	RBM3	GGATGGTCGTCAGATCCGTG
ENSG00000102317	RBM3	GGTGGTTATGACCGCTACTC
ENSG00000102317	RBM3	CATGAGAGCCATGAACGGAG
ENSG00000064419	TNPO3	CCTGCCGGATCTGTAACAAC
ENSG00000064419	TNPO3	TAGCAATGAGTCCCGTAAAG
ENSG00000064419	TNPO3	TTGGGAAGTTGGTTTAACTT
ENSG00000132424	PNISR	CCAATGCAGCCCAATCAATC
ENSG00000132424	PNISR	CCCACTGCAAAATTATCGGG
ENSG00000132424	PNISR	GGTGGCATCCATGGCTGATC
ENSG00000086758	HUWE1	GGACCGCTTCGATGGAATAC
ENSG00000086758	HUWE1	CAGCACCACTTGCATATCAG

ENSG00000104852	SNRNP70	TACGGCGCAATGCCACAATA
ENSG00000104852	SNRNP70	AGGTATGGAATAGGGTCACG
ENSG00000104852	SNRNP70	TGAGCATTGGGATCATTGTG
ENSG00000179950	PUF60	ATGCTCTTGATGGGGCCAAA
ENSG00000179950	PUF60	GGGGACCTCATACTCCACGA
ENSG00000179950	PUF60	CAGATGAACTCGGTGATGCT
ENSG00000118263	KLF7	GCAGCAGGGGGTCTAAGCGA
ENSG00000118263	KLF7	GAAGCCTTATAAGTGCTCAT
ENSG00000118263	KLF7	ACCTACAGACGGAGCCCCGG
ENSG00000130699	TAF4	AACACCTATCATTGCACGGC
ENSG00000130699	TAF4	GAGCGCTGCAGGGTTGCACT
ENSG00000130699	TAF4	CTCCTCAGCGCACGGTACCA
ENSG00000130811	EIF3G	TCCACCTGGTCGGCCCAACT
ENSG00000130811	EIF3G	TCACCAGCGAGCTCCTCAAG
ENSG00000130811	EIF3G	GTTTCCGTTGATGACCTCCT
ENSG00000177600	RPLP2	CGTCCACATCAACTCACCCT
ENSG00000177600	RPLP2	TACCTGCTGGCTGCCCTAGG
ENSG00000177600	RPLP2	CTTGGACAGCGTGGGTATCG
ENSG00000106263	EIF3B	TGTACTCAAAGATCGGCCCC
ENSG00000106263	EIF3B	ATTCCGGGTCAACCTCTTTA
ENSG00000106263	EIF3B	CTTGTCAAGCTTGTAGCCGT
ENSG00000141867	BRD4	GATTTCTCAATCTCGTCCCA
ENSG00000141867	BRD4	ATTAAAACGCCTATGGATAT

ENSG00000108848	LUC7L3	GTAGAACGTAGGATCAGACG
ENSG00000108848	LUC7L3	TTGATGGGAAAACAACACAT
ENSG00000108848	LUC7L3	TAGTAGGAGATGCCCAGTCC
ENSG00000151651	ADAM8	GAGAGGGTGAGCTACGTCCT
ENSG00000151651	ADAM8	CTTCTACCAGGGCCACGTAG
ENSG00000151651	ADAM8	CTGATCGAGCCCCTGGATGA
ENSG00000134684	YARS	TTACTGGGGAACGGCAACCA
ENSG00000134684	YARS	TCATGTTATCCAGGTATGCG
ENSG00000134684	YARS	GAGAAGCTCAAGTTCATCAA
ENSG00000120156	TEK	AGGTGTACTTCTAGAATATC
ENSG00000120156	TEK	GTACAGAGATGGTTGCATTC
ENSG00000120156	TEK	CTACTGAGAAATGATCCGTA
ENSG00000131759	RARA	GAAGGTGCGCAAAGCGCACC
ENSG00000131759	RARA	GAGGGTGATCTGGTCGGCGA
ENSG00000131759	RARA	AGATGGATGATGCGGAGACG
ENSG00000165417	GTF2A1	CTGAGGCTGAAAGATATATT
ENSG00000165417	GTF2A1	ACAAGCTCCTGTTATACAGC
ENSG00000165417	GTF2A1	AGTGGATGAACAAGTACTGA
ENSG00000087095	NLK	ACCAGATTCTGGAAGACGTT
ENSG00000087095	NLK	TCTCTTGCAAAAGGGTCTTC
ENSG00000087095	NLK	GTGGAGGTTGGAGTATGTCA
ENSG00000136485	DCAF7	TTGATACGACATGCACCATC
ENSG00000136485	DCAF7	TTTGTCATGGGCGATCAGCT

ENSG00000099139	PCSK5	ACCATAGCAGGACGATTAAA
ENSG00000099139	PCSK5	AGAAGTGGTAGTAGTCCTTC
ENSG00000099139	PCSK5	ATTGCTTTCAACGCCAAGAT
ENSG00000187758	ADH1A	ACAGACTTTCTCTAGAGGCG
ENSG00000187758	ADH1A	GGCCCATGAAGTTCGTATTA
ENSG00000187758	ADH1A	GTCCAGTGTAAGCAATCCTC
ENSG00000120833	SOCS2	TGCTGACGTGTAGAGCGGTT
ENSG00000120833	SOCS2	AGGTGAACAGTGCCGTTCCG
ENSG00000120833	SOCS2	CGCCATTCCCGGAGGGCTCA
ENSG00000196361	ELAVL3	GTTGGTCTTGCTGTCGTCAG
ENSG00000196361	ELAVL3	TCTGTAATTTGAGGCCGTTG
ENSG00000196361	ELAVL3	ATGCTAACCTGTACGTCAGC
ENSG00000171861	MRM3	TTATTCCTTGTGGCGTTACG
ENSG00000171861	MRM3	GCCACATAGACCCGAGTGTC
ENSG00000062038	CDH3	GATGACTTCACTGTGCGGAA
ENSG00000062038	CDH3	GTGTCTTCGTAAGATACGTT
ENSG00000062038	CDH3	CACCATTCTCTGACACAGCG
ENSG00000135750	KCNK1	GAAGTAGAGGACCGGCCTGC
ENSG00000135750	KCNK1	TATTGCCATGTTGGTAGTTC
ENSG00000135750	KCNK1	TTCAGAGAGCTCTATAAGAT
ENSG00000174125	TLR1	AATTTCAAACGTGAAGCTAC
ENSG00000174125	TLR1	GGAATGGAGTACTGCGGAAT
ENSG00000174125	TLR1	ATAGTGGGCACGATTCTTTC

ENSG00000155545	MIER3	ACTTGGGGAACTATTTGCAC
ENSG00000155545	MIER3	TACTGCATGTGATGGTGATA
ENSG00000155545	MIER3	ACCTTGTTGAGACTTCATTA
ENSG00000102241	HTATSF1	GACGGACACTCCCTACGAGT
ENSG00000102241	HTATSF1	GCTACATATCAGGCCAATTA
ENSG00000102241	HTATSF1	TCTGGCATCAGTGGGTTCCG
ENSG00000119801	YPEL5	TCACATCTCGAACCATGTGG
ENSG00000119801	YPEL5	GACAGCCAGCGATATAAGGA
ENSG00000119801	YPEL5	AGCTCACATCTCGAACCATG
ENSG00000108424	KPNB1	GCACATGAAGGAGTCGACAT
ENSG00000108424	KPNB1	ACCAGCTGAGGAATGAGTTC
ENSG00000108424	KPNB1	GCTAGCTTCACATTATTACT
ENSG00000162923	WDR26	GCATGAGGAGATCAACAGTC
ENSG00000162923	WDR26	CATGACATGATTTCGGAATT
ENSG00000162923	WDR26	GAAGTACCTAGAATACCTGG
ENSG00000146457	WTAP	CTTGGGAAGAGGTTCTTCGT
ENSG00000146457	WTAP	CAAGTTGTGCAATACGTCCC
ENSG00000176887	SOX11	GTCTGGGTCGCTCTCGTCCA
ENSG00000122692	SMU1	ATCCTTAGAAAAGCTTAGAC
ENSG00000122692	SMU1	CTTTTTCAGGCATACCCAGA
ENSG00000122692	SMU1	CCTTCAAGTACTGCATAATA
ENSG00000122882	ECD	GCTGTGAATAGGCGCATCAG
ENSG00000122882	ECD	GCAGGTCAATAGGGTCTCGT

ENSG00000122882	ECD	AGAAGGTTCTGCTCAGTACC
ENSG00000129351	ILF3	TTCCTGCTCGTCTATCCAGT
ENSG00000129351	ILF3	CATCACTCCCCGCAGGGTTC
ENSG00000129351	ILF3	TGCCTGTCCAGAACGTCCGG
ENSG00000165699	TSC1	ATGCTGGACTCCCCCATGCT
ENSG00000165699	TSC1	CACCTTGGTGGATTATTACC
ENSG00000165699	TSC1	GACGTCGTTGTCCTCACAAC
ENSG00000100138	SNU13	CTTGGAGCGCACAAACACGT
ENSG00000186591	UBE2H	GCTTGTATTCTTCTGGTCGG
ENSG00000186591	UBE2H	CATCCAGAAATACGCCACGG
ENSG00000161547	SRSF2	CCAAGTCCAGATCCGCACGA
ENSG00000161547	SRSF2	CCTGGACCGCGAACGAGATC
ENSG00000161547	SRSF2	TCACGACAAGCGCGACGCTG
ENSG00000115875	SRSF7	CATTTGGATCAAAGGGACGT
ENSG00000115875	SRSF7	GATACTCTCGCTCACGCAGC
ENSG00000204569	PPP1R10	AGAAGTTCTTTGGGGTCTAT
ENSG00000204569	PPP1R10	AGTGAGCGGTAGATGCTGCA
ENSG00000171862	PTEN	AATCCCATAGCAATAATGTT
ENSG00000171862	PTEN	AACTTGTCTTCCCGTCGTGT
ENSG00000171862	PTEN	CAATTCAGGACCCACACGAC
ENSG00000114209	PDCD10	GGTTTGATGAATTAGTCGGT
ENSG00000114209	PDCD10	ATATCAATACCAGAACCGCA
ENSG00000105618	PRPF31	TCCTGCACATCCTCGATCGC

ENSG00000105618	PRPF31	CAGCTCGTTTTCGATCTCCA
ENSG00000105618	PRPF31	ACTTATCCCGGATGAACTTA
ENSG00000125952	MAX	ATGCACTGGAACGAAAACGT
ENSG00000125952	MAX	CAGGAAGAAGCTCCGGATGG
ENSG00000125952	MAX	CACCATCTCTGCCTTCGATG
ENSG00000171634	BPTF	GTTCCGCAGTACCTCGTAAA
ENSG00000171634	BPTF	TATATTCGACATGAACCTAT
ENSG00000171634	BPTF	GCTATCTGGATTCCGCAATC
ENSG00000169564	PCBP1	GGAGTCAATTCCGGCGAATC
ENSG00000169564	PCBP1	TGGCGCGCGGATCAACATCT
ENSG00000169564	PCBP1	CGCTATGATCATCGACAAGC
ENSG00000135473	PAN2	CTCCTCGTGCAAGTCAAAGT
ENSG00000135473	PAN2	CTACTCTGAATTGCACAGCG
ENSG00000135473	PAN2	GCAGCACTCTACTCGTTGGT
ENSG00000060339	CCAR1	GAGGACAGAAGAATCCGCCA
ENSG00000060339	CCAR1	TGTGTTAAGAGGGTTTGCTG
ENSG00000060339	CCAR1	GTAGACAGGCTAAGGCTTGT
ENSG00000115607	IL18RAP	GTTGCAGGAGAGCGAATTAA
ENSG00000115608	IL18RAP	TAAAATCATCTTGACACAAC
ENSG00000115609	IL18RAP	CTCACAGGATGCATTTGTCT
ENSG00000134884	ARGLU1	TGAACGAGAAGTTCTCCGAA
ENSG00000134884	ARGLU1	AATAGAAGAAAAACTCATCG
ENSG00000134884	ARGLU1	TGAGGAGCTAGAGCGAATAC

ENSG00000163635	ATXN7	AACGGCCAAAGGCGGCTTGC
ENSG00000163635	ATXN7	GGAAGCAACCGTTCTTCCAG
ENSG00000163635	ATXN7	GCATGTCAAAGACCGGGTGC
ENSG00000135801	TAF5L	CATCTGAGTCCACGTACTGC
ENSG00000135801	TAF5L	CTGTGCTCTTCGGACTGTTT
ENSG00000135801	TAF5L	GGACAACTCGGTGCGCGTCT
ENSG00000086598	TMED2	TTGGAGTCATGGTGGACATC
ENSG00000086598	TMED2	AAAGCACGAACAGGAATACA
ENSG00000086598	TMED2	AACAGGAATACATGGAAGTC
ENSG00000108883	EFTUD2	ATCATGACGATGACCACCCT
ENSG00000108883	EFTUD2	CTTTGTGGACATCTCCACCA
ENSG00000108883	EFTUD2	CAAGATTGACCGGCTGATCC
ENSG00000139549	DHH	TAAGGAGCGGGTGAACGCTT
ENSG00000139549	DHH	AACACCGGTGCAAAGTCGCC
ENSG00000139549	DHH	AGTCACTCGTAGGCGCACTC
ENSG00000083312	TNPO1	TAAAGTCTGTTACACCATTT
ENSG00000083312	TNPO1	GATCACAACTATAGCCTCCA
ENSG00000083312	TNPO1	TTTTCACCGATCGAGGACGG
ENSG00000105576	TNPO2	GTCTGGTCAGGACGAAAATC
ENSG00000105576	TNPO2	AATGGTGGCTCGGATGAGCG
ENSG00000105576	TNPO2	GTGCCTTCACGTTGTTCTTG
ENSG00000114030	KPNA1	AGGCGAAAGTTCTCTTTTCC
ENSG00000114030	KPNA1	GCACACCAGGAGTAGTGGCC

ENSG00000114030	KPNA1	CCATGACCCGGAATGCAGTA
ENSG00000182481	KPNA2	AGCCCGGATTATGTTGTCTA
ENSG00000182481	KPNA2	ACCATGCCAATTCGTTCATT
ENSG00000182481	KPNA2	ATCTGCTCAACAGCATCTAT
ENSG00000102753	KPNA3	CATTACAGTTTGAAGCTGCT
ENSG00000102753	KPNA3	AACAACCTGGGTCTGCTCGT
ENSG00000102753	KPNA3	TTACAGCTTGAACTTGTTGC
ENSG00000186432	KPNA4	AATGCTTCAAGTGATAACCA
ENSG00000186432	KPNA4	TTCTTCTATAAGATTGCCTA
ENSG00000186432	KPNA4	TATAGATGGTGATTATAGAG
ENSG00000196911	KPNA5	TGACCCCAGCATTGTACCTC
ENSG00000196911	KPNA5	TAAAGTTGAAATCCATCCAT
ENSG00000196911	KPNA5	ACTTGATCTATTGGTGGATT
ENSG00000025800	KPNA6	CATGAGAAGACTATCGAACA
ENSG00000025800	KPNA6	ACTTCATCTATTGGAGGACT
ENSG00000025800	KPNA6	GGCAATATTCGTTAGAGCCC
ENSG00000185467	KPNA7	GTTCAGCATACCCGCATCAA
ENSG00000185467	KPNA7	CCCCGCTGCTACGTTGCTCA
ENSG00000196497	IPO4	GACTGAACACCCGCTGGCGA
ENSG00000196497	IPO4	CTGCAGGGCCGTCAGGATCA
ENSG00000196497	IPO4	CTCAGCCACCATTTTTCGAA
ENSG00000065150	IPO5	CAAGAGACGTCTTAGGAGAA
ENSG00000065150	IPO5	GATGGCAGTCTGAACATCAG

ENSG00000205339	IPO7	TAGCCAACAAGCACTGTTAT
ENSG00000205339	IPO7	AACATGATTATCCAAGCCGC
ENSG00000205339	IPO7	ACCAATGGACTCCGCTCCTC
ENSG00000133704	IPO8	TCTTCCGAACTATTATCGAC
ENSG00000133704	IPO8	TGAGGATGATAGACCAGAAC
ENSG00000133704	IPO8	CTTTTTGAAAACCTATGCAG
ENSG00000198700	IPO9	AATCTCCACGGCTCGGGAAC
ENSG00000198700	IPO9	GCTGTACCACGGGAAAGATC
ENSG00000198700	IPO9	ATGGCCCCACATCTGACAGT
ENSG00000086200	IPO11	AACATGGAATTGATCGCTAC
ENSG00000086200	IPO11	GAGAAAACTACTCTGCGTGC
ENSG00000086200	IPO11	TGTGCTGTCGAAGATCATCC
ENSG00000117408	IPO13	CAGCCGAGTCAGTACAATCT
ENSG00000117408	IPO13	CGCCTACCCCAGTACCGCAA
ENSG00000117408	IPO13	TGGCATCTGTCGCATCGCTG
ENSG00000047410	TPR	GCCGCAAGCTTTGACACTCT
ENSG00000047410	TPR	GTGGAATTTCTCTTCCATAC
ENSG00000047410	TPR	AAAGAGCAATGAACTAACCC

Appendix Table S5 TPM of RBM3 mRNA upon specified RBM3 regulator KD compared to the control in K562 and HepG2 cells from ENCODE.

Gene (knocked down)	RBM3 TPM	Gene TPM	Experiment ID	Cell Type
HNRNPH1	44.21	472.5	ENCFF039DFP	K562
HNRNPH1	44.93	412.45	ENCFF713MXN	
Control	111.84	447.63	ENCFF616BYI	
Control	126.13	466.88	ENCFF586TGE	
HNRNPH1	32.46	392.48	ENCFF293ODK	HepG2
HNRNPH1	36.26	361.24	ENCFF266YWO	
Control	105.37	650.81	ENCFF053QJC	
Control	106.98	657.38	ENCFF200BWY	
SNRNP70	22.15	43.97	ENCFF367WUN	HepG2
SNRNP70	21.45	29.54	ENCFF058OGQ	
Control	36.55	125.33	ENCFF873KLR	
Control	43.65	159.84	ENCFF311ACC	
PUF60	85.57	32.65	ENCFF682SFK	K562
PUF60	85.62	23.87	ENCFF461EMF	
Control	197.6	75.15	ENCFF585BAL	
Control	196.86	73.91	ENCFF643OYR	
KPNB1	170.59	104.44	ENCFF933IUO	K562
KPNB1	174.8	120.52	ENCFF298FLZ	

Control	179.89	373.34	ENCFF160UOH	
Control	159.96	356.98	ENCFF116ZHL	
KPNB1	101.47	102.87	ENCFF819AUU	HepG2
KPNB1	98.51	107.14	ENCFF628VDB	
Control	86.41	251.29	ENCFF565TSG	
Control	90.62	253.73	ENCFF073IFA	

Appendix Table S6 Analysis of RBM3 transcript alternative splicing in HNRNPH1 KD versus WT i-neurons, K562 and HepG2 cells by rMATS

Cell type	exonStart_0base	exonEnd	upstreamES	upstreamEE	downstreamES	downstreamEE	FDR	IncLevel1	IncLevel2	IncLevelDifference	Note
i-neuron	48575560	48575630	48575167	48575283	48576313	48576419	1	0.942,0.932,0.988,0.969	0.967,0.971,0.952,0.956	-0.004	
i-neuron	48575560	48575667	48574951	48575283	48575814	48576083	1	1.0,0.996,1.0,1.0	1.0,1.0,1.0,1.0	-0.001	
i-neuron	48575560	48575667	48574951	48575283	48576313	48576419	1	0.984,0.978,0.996,0.991	0.99,0.991,0.984,0.986	0	Exon 3 Inclusion (relative to Exon 2 and 4)
i-neuron	48575560	48575667	48574951	48575283	48577025	48577109	1	1.0,1.0,1.0,0.997	1.0,1.0,1.0,1.0	-0.001	Exon 3 Inclusion (relative to Exon 2 and 6)
i-neuron	48575560	48576083	48575167	48575283	48576313	48576419	1	0.912,0.883,0.979,0.948	0.943,0.947,0.913,0.921	-0.001	
i-neuron	48575814	48576083	48575167	48575283	48576313	48576419	1.09E-14	0.46,0.416,0.793,0.592	0.154,0.114,0.075,0.067	0.463	
i-neuron	48575814	48576083	48575560	48575667	48576313	48576419	0	0.022,0.027,0.02,0.022	0.002,0.002,0.002,0.002	0.021	Exon 3a-L Inclusion
i-neuron	48575921	48576083	48575560	48575667	48576313	48576419	0.03201581	0.014,0.016,0.015,0.014	0.001,0.001,0.002,0.001	0.013	Exon 3a-S Inclusion
i-neuron	48576313	48576419	48575167	48575283	48577025	48577109	1	1.0,1.0,1.0,0.724	1.0,1.0,1.0,1.0	-0.069	Exon 4 Inclusion (relative to Exon 2 and 6)
i-neuron	48576313	48576419	48575560	48575667	48577025	48577109	1	1.0,1.0,1.0,0.997	1.0,1.0,1.0,1.0	-0.001	Exon 4 Inclusion (relative to Exon 3 and 6)
i-neuron	48576507	48576604	48575898	48576419	48577025	48577109	1	1.0,1.0,1.0,1.0	1.0,1.0,1.0,1.0	0	Exon 5 Inclusion (relative to Exon 4 and 6)
K562	48575560	48575630	48575167	48575283	48576313	48576419	1	1.0,1.0	1.0,0.98	0.01	
K562	48575560	48575667	48574951	48575283	48575814	48576083	1	1.0,1.0	1.0,0.985	0.008	

K562	48575560	48575667	48574951	48575283	48576313	48576419	1	1.0,1.0	1.0,0.991	0.004	
K562	48575560	48576083	48575167	48575283	48576313	48576419	1	1.0,1.0	1.0,0.98	0.01	
K562	48575814	48576083	48575167	48575283	48576313	48576419	1	1.0,1.0	1.0,0.973	0.014	
K562	48575814	48576083	48575560	48575667	48576313	48576419	6.78E-09	0.127,0.128	0.411,0.293	-0.224	Exon 3a-L Inclusion
K562	48575921	48576083	48575560	48575667	48576313	48576419	1.46E-07	0.112,0.115	0.396,0.264	-0.217	Exon 3a-S Inclusion
HepG2	48575560	48575630	48575167	48575283	48576313	48576419	1	1.0,1.0	0.833,1.0	0.084	
HepG2	48575560	48575667	48574951	48575283	48576313	48576419	1	1.0,1.0	0.899,1.0	0.05	
HepG2	48575560	48576083	48575167	48575283	48576313	48576419	1	1.0,1.0	0.811,1.0	0.095	
HepG2	48575814	48576083	48575560	48575667	48576313	48576419	2.03E-06	0.081,0.114	0.301,0.487	-0.296	Exon 3a-L Inclusion
HepG2	48575921	48576083	48575560	48575667	48576313	48576419	3.44E-08	0.056,0.094	0.294,0.418	-0.281	Exon 3a-S Inclusion

Appendix Table S7 Key resources used in this study

REAGENT or RESOURCE	SOURCE	IDENTIFIER
**Antibodies**		
Rabbit anti-RBM3 antibody	Proteintech	Cat#14363-1-AP
Rabbit anti-GFP antibody	Abcam	Cat#ab290
Mouse anti-Cas9 antibody	Cell Signaling	Cat#14597S
Mouse anti-GAPDH antibody	Santa Cruz	Cat#sc-32233
Rabbit anti-HNRNPH1 antibody	Bethyl	Cat#A300-511A
Mouse anti-FLAG	Sigma	Cat#F1804
Rabbit anti-HNRNPH1 antibody	Abcam	Cat#ab13074
Rabbit anti-HNRNPH1 antibody	ProteinTech	Cat#14774-1-AP
Mouse anti-HNRNPH/F antibody	Santa Cruz	Cat#sc-32310
Rabbit IgG	Santa Cruz	Cat#sc-2027
Mouse IgG	Santa Cruz	Cat#sc-2025
Rabbit anti-SmD3 antibody	Antibodies Online	Cat#ABIN653228
Mouse anti-SmB (Sm proteins) antibody	(Lerner *et al*, 1981)	N/A

Goat anti-rabbit HRP secondary antibody	Biorad	Cat#1706515
Goat anti-mouse HRP secondary antibody	Biorad	Cat#1706516
Donkey anti-rabbit 680LT	LI-COR	Cat#926-68023
Donkey anti-mouse 680LT	LI-COR	Cat#926-68022
Donkey anti-rabbit 800CW	LI-COR	Cat#926-32213
Donkey anti-mouse 800CW	LI-COR	Cat#926-32212
**Bacterial and virus strains**		
5-alpha Competent E. coli	NEB	Cat#C2987
One Shot ccdB Survival 2 T1R Competent Cells	ThermoFisher Scientific	Cat#A10460
Stable Competent E. coli (High Efficiency)	NEB	Cat#C3040I
Lentivirus: pLVPB-gCherry-PGK-BFP-2A-mCherry	This study	N/A
Lentivirus: whole-genome CRISPR sgRNA library	This study	N/A
Lentivirus: sgRNA-expressing lentivirus (each contains one of the sgRNAs listed in Table S1)	This study	N/A
**Chemicals, peptides, and recombinant proteins**		
TeSR-E8 Medium	Stemcell Technologies	Cat#05990
StemFlex Medium	ThermoFisher Scientific	Cat#A3349401
EDTA, pH8.0	ThermoFisher Scientific	Cat#15575020
Vitronectin	ThermoFisher Scientific	Cat#A14700
Cryostor Cs10 Cryopreservation	Merck	Cat#C2874-100ML
StemPro Accutase Cell Dissociation Reagent	ThermoFisher Scientific	Cat#A1110501
0.25% Trypsin-EDTA	Merck	Cat#T4049
Rho-associated protein kinase (ROCK) inhibitor	BD Biosciences	Cat#Y-27632

Geltrex LDEV-Free, hESC-Qualified, Reduced Growth	Life Technologies	Cat#A1413302
DMEM/F12, GlutaMAX Supplement	Thermo Fisher Scientific	Cat#31331028
N-2 Supplement (100X)	Thermo Fisher Scientific	Cat#17502048
MEM Non-Essential Amino Acids Solution (100X)	Thermo Fisher Scientific	Cat# 11140050
2-Mercaptoethanol (50 mM)	Thermo Fisher Scientific	Cat#31350010
Doxycycline hyclate	Merck	Cat#D9891
Neurobasal medium	Thermo Fisher Scientific	Cat#21103049
Neurobasal medium, minus phenol red	Thermo Fisher Scientific	Cat#12348017
B-27 Supplement (50X), minus vitamin A	Thermo Fisher Scientific	Cat#12587010
GlutaMAX Supplement	Thermo Fisher Scientific	Cat# 35050061
Neurotrophin-3 (NT-3)	PeproTech	Cat#450-03
Brain Derived Neurotrophic Factor (BDNF)	PeproTech	Cat#450-02
Penicillin-Streptomycin	Thermo Fisher Scientific	Cat#15140122
HiFi Cas9 nuclease V3	Integrated DNA Technologies	Cat#1081060
Proteinase K, recombinant, PCR grade	Thermo Fisher Scientific	Cat#EO0491
Sodium Acetate, 3M, pH 5.2	Merck	Cat#567422-100ML
Poly-L-lysine	Merck	Cat#P4707
Laminin	Merck	Cat#L2020-1MG
Dithiothreitol (DTT)	Thermo Fisher Scientific	Cat#R0861, 11896744

Tween-20	Merck	Cat#P9416-50ML
RIPA buffer	Merck	Cat#R0278-500ML
DNase 1	Zymo	Cat#E1011-A
Protein G Dynabeads	Life Technologies	Cat#100090
HALT protease inhibitor cocktail	Thermo Fisher Scientific	Cat#1861278
TRIzol	Invitrogen	Cat#15596018
HALT protease and phosphatase inhibitor cocktail	Thermo Fisher Scientific	Cat#78442
cOmplete, EDTA-free Protease Inhibitor Cocktail	Merck/Roche	Cat#11873580001
Cycloheximide	Biovision	Cat#1041-1
Actinomycin D	Merck	Cat#A1410-2MG
SMG1 inhibitor	ProbeChem	Cat#PC-35788
Pyridostatin hydrochloride	Merck	Cat#SML2690-5MG
Donkey serum	Merck	Cat#D9663
Hoechst	Thermo Fisher Scientific	Cat#62249
**Critical commercial assays**		
RNeasy Plus Mini Kit	Qiagen	Cat#74134
Absolutely RNA Minprep Kit	Agilent	Cat#400800
SuperScript IV First-Strand Synthesis System	Thermo Fisher Scientific	Cat#18091050
Q5 High-Fidelity DNA Polymerase	NEB	Cat#M0492L
NEBuilder HiFi DNA Assembly Master Mix	NEB	Cat#E2621L
GoTaq Taq G2 Green Master Mix	Promega	Cat#M7823
Guide-it Long ssDNA Production System, 25 Rxns	Takara Bio	Cat#632644
P3 Primary Cell 4D-Nucleofector Kit L (12 RXN)	Lonza	Cat#V4XP-3012

Alt-R HDR Enhancer, 500 µL	Integrated DNA Technologies	Cat#226687070
Qubit RNA Broad Range Assay Kit	Thermo Fisher Scientific	Cat#Q10211
RNA Screentape	Agilent	Cat#5067-5576
RNA Screentape Ladder	Agilent	Cat#5067-5578
RNA Screentape Buffer	Agilent	Cat#5067-5577
Loading tips, 10 pk	Agilent	Cat#5067-5599
DNA HS D1000 Screentape	Agilent	Cat#5067-5584
DNA HS D1000 Screentape Buffer	Agilent	Cat#5067-5585
DNA HS D1000 Screentape Ladder	Agilent	Cat#5067-5587
TruSeq Stranded mRNA Library Prep	Illumina	Cat#20020594
Qubit RNA BR Assay Kit	Thermo Fisher Scientific	Cat#Q10211
Qubit dsDNA BR Assay Kit	Thermo Fisher Scientific	Cat#Q32853
BCA Protein Assay Kit	Thermo Fisher Scientific	Cat#23225
Immobilon Western Chemiluminescent HRP Substrate	Merck	Cat#WBKLS0500
Lipofectamine LTX Reagent with Plus Reagent	Thermo Fisher Scientific	Cat#A12621
NEBNext Library Quant Kit for Illumina	NEB	Cat#E7630L
AMPure XP Reagent for PCR Purification	Beckman Coulter	Cat#A63881
NextSeq 500/550 High Output Kit v2.5 (75 Cycles)	Illumina	Cat#20024906
Lipofectamine 2000 reagent	Thermo Fisher Scientific	Cat#11668-027
TransIT-LT1	Mirus	Cat#22043217
dNTP Mix	Thermo Fisher Scientific	Cat#R0192

RiboLock RNAse inhibitor	Thermo Fisher Scientific	Cat#EO0381
AffinityScript Multi temperature Multiple Reverse Transcriptase	Agilent	Cat#600107-51
AffinityScript RT buffer	Agilent	Cat#600100-52
DTT	Agilent	Cat#600100-53
QuickChange Lightning Multi Site-Directed Mutagenesis Kit	Agilent	Cat#210513
**Experimental models: Cell lines**		
Human iPSC/iPSC-derived neuron: Bob (wild-type)	(Pawlowski *et al*, 2017)	N/A
Human iPSC/iPSC-derived neuron: Cas9	This study	N/A
Human iPSC/iPSC-derived neuron: GFP-RBM3	This study	N/A
HeLa	ECACC	Cat#93021013
HEK293T (Lenti-X 293T)	Clontech Takara	Cat#632180
**Oligonucleotides**		
Primers used in this study are listed in Table S1	This study	N/A
RBM3-N gRNA#1 - 5' CUGCCAUGUCCUCUGAAGA 3'	This study	N/A
RBM3-N gRNA#2 - 5' UUUCCUUCUUCAGAGGACA 3'	This study	N/A
HNRNPH siRNA#1 - 5' GGAAAUAGCUGAAAAGGCUdTdT 3'	Microsynth	Cat#2288931
HNRNPH siRNA#2 - 5' GAGAGUACACAUUGAAAUUdTdT 3'	Microsynth	Cat#2276387
Control siRNA	Dharmacon	Cat#D-001810-10-20
**Recombinant DNA**		
Plasmid: pcDNA3-EGFP	Addgene	Cat#13031
Plasmid: GFP-RBM3 repair template	This study	N/A
Plasmid: pLVPB-gCherry-PGK-BFP-2A-mCherry	This study	N/A
Lentiviral sgRNA cloning vector: pLVPB_U6_sgRNAv2fl_shortccdB_PGK_Puro_BFP	(Metzakopian *et al*, 2017)	N/A

Lentiviral sgRNA expression plasmids (all sgRNA sequences listed in Table S1 individually cloned into the cloning vector)	This study	N/A
Lentiviral packaging plasmid: psPax2	Addgene	Cat#12260
Lentiviral packaging plasmid: pMD2.G	Addgene	Cat#12259
Plasmid: RBM3 Minigene	This study	N/A
Plasmid: delGGGG-RBM3 Minigene	This study	N/A
**Software and algorithms**		
Prism (v9.0.2)	GraphPad	https://www.graphpad.com
Design and Analysis Software (v2.6.0)	Thermo Fisher Scientific	N/A
FIJI (v2.1.0)	(Schindelin *et al*, 2012)	https://fiji.sc/
MATLAB (R2020a)	MathWorks	https://www.mathworks.com
MAGeCK RRA	(Li *et al*, 2014)	https://sourceforge.net/projects/mageck/
Bcl2fastq (v2.2.0)	Illumina	https://emea.support.illumina.com/downloads/bcl2fastq-conversion-software-v2-20.html
Metascape (v3.5)	(Zhou *et al*, 2019)	https://metascape.org/
STRING (v11.5)	(Szklarczyk *et al*, 2020)	https://string-db.org/
FlowJo (v10.7.2)	BD Biosciences	https://www.flowjo.com
nf-core/rnaseq pipeline (v3.3)	(Patel *et al*, 2021)	https://nf-co.re/rnaseq
nf-core/clipseq pipeline (v1.0.0)	(Ewels *et al*, 2020)	https://nf-co.re/clipseq
DESeq2 (v3.15)	(Love *et al*, 2014)	https://bioconductor.org/packages/release/bioc/html/DESeq2.html
rmats2sashimiplot (v2.0.4)	(Shen *et al*, 2014)	https://github.com/Xinglab/rmats2sashimiplot
FastQC (v0.11.8)	Babraham Institute	https://www.bioinformatics.babraham.ac.uk/projects/fastqc/
STAR (v2.6.1b)	(Dobin *et al*, 2013)	https://github.com/alexdobin/STAR
SAMtools (v1.9)	(Li *et al*, 2009)	http://www.htslib.org/
rMATS (v4.1.2)	(Shen *et al*, 2014)	https://github.com/Xinglab/rmats-turbo

clipplotr (v1.0.0)	(Chakrabarti *et al*, 2021)	https://github.com/ulelab/clipplotr
QGRS Mapper	(Kikin *et al*, 2006)	https://bioinformatics.ramapo.edu/QGRS/index.php
Other		
MatTek glass-bottom dishes	MatTek	Cat#P35G-1.0-14-C

References

Chakrabarti AM, Capitanchik C, Ule J & Luscombe NM (2021) clipplotr - a comparative visualisation and analysis tool for CLIP data. *bioRxiv*: 2021.09.10.459763

Dobin A, Davis CA, Schlesinger F, Drenkow J, Zaleski C, Jha S, Batut P, Chaisson M & Gingeras TR (2013) STAR: ultrafast universal RNA-seq aligner. *Bioinformatics* 29: 15–21

Ewels PA, Peltzer A, Fillinger S, Patel H, Alneberg J, Wilm A, Garcia MU, Di Tommaso P & Nahnsen S (2020) The nf-core framework for community-curated bioinformatics pipelines. *Nat Biotechnol* 38: 276–278

Kikin O, D'Antonio L & Bagga PS (2006) QGRS Mapper: a web-based server for predicting G-quadruplexes in nucleotide sequences. *Nucleic Acids Res* 34: W676–82

Lerner MR, Boyle JA, Hardin JA & Steitz JA (1981) Two novel classes of small ribonucleoproteins detected by antibodies associated with lupus erythematosus. *Science* 211: 400–402

Li H, Handsaker B, Wysoker A, Fennell T, Ruan J, Homer N, Marth G, Abecasis G, Durbin R & Others (2009) 1000 genome project data processing subgroup. The sequence alignment/map format and SAMtools. *Bioinformatics* 25: 2078–2079

Li W, Xu H, Xiao T, Cong L, Love MI, Zhang F, Irizarry RA, Liu JS, Brown M & Liu XS (2014) MAGeCK enables robust identification of essential genes from genome-scale CRISPR/Cas9 knockout screens. *Genome Biol* 15: 554

Love MI, Huber W & Anders S (2014) Moderated estimation of fold change and dispersion for RNA-seq data with DESeq2. *Genome Biol* 15: 550

Metzakopian E, Strong A, Iyer V, Hodgkins A, Tzelepis K, Antunes L, Friedrich MJ, Kang Q, Davidson T, Lamberth J, *et al* (2017) Enhancing the genome editing toolbox: genome wide CRISPR arrayed libraries. *Sci Rep* 7: 2244

Patel H, Ewels P, Peltzer A, Hammarén R, Botvinnik O, Sturm G, Moreno D, Vemuri P, silviamorins, Pantano L, *et al* (2021) nf-core/rnaseq: nf-core/rnaseq v3.5 - Copper Chameleon Zenodo

Pawlowski M, Ortmann D, Bertero A, Tavares JM, Pedersen RA, Vallier L & Kotter MRN (2017) Inducible and Deterministic Forward Programming of Human Pluripotent Stem Cells into Neurons, Skeletal Myocytes, and Oligodendrocytes. *Stem Cell Reports* 8: 803–812

Schindelin J, Arganda-Carreras I, Frise E, Kaynig V, Longair M, Pietzsch T, Preibisch S, Rueden C, Saalfeld S, Schmid B, *et al* (2012) Fiji: an open-source platform for biological-image analysis. *Nat Methods* 9: 676–682

Shen S, Park JW, Lu Z-X, Lin L, Henry MD, Wu YN, Zhou Q & Xing Y (2014) rMATS: robust and flexible detection of differential alternative splicing from replicate RNA-Seq data. *Proc Natl Acad Sci U S A* 111: E5593–601

Szklarczyk D, Gable AL, Nastou KC, Lyon D, Kirsch R, Pyysalo S, Doncheva NT, Legeay M, Fang T, Bork P, *et al* (2020) The STRING database in 2021: customizable protein–protein networks, and functional characterization of user-uploaded gene/measurement sets. *Nucleic Acids Res* 49: D605–D612

Zhou Y, Zhou B, Pache L, Chang M, Khodabakhshi AH, Tanaseichuk O, Benner C & Chanda SK (2019) Metascape provides a biologist-oriented resource for the analysis of systems-level datasets. *Nat Commun* 10: 1523
