# Supplementary material for: HNRNPH1 regulates the neuroprotective cold‐shock protein RBM3 expression through poison exon exclusion
Source: EMBO J. 2023 May 30;42(14):e113168. doi: 10.15252/embj.2022113168 (PMC10350819; doi:10.15252/embj.2022113168)
Supplement: Supplementary file 2 — Expanded View Figures PDF [file EMBJ-42-e113168-s001.pdf]

Expanded View Figures

Figure EV1. Characterisation of GFP-RBM3 human iPSC reporter line for CRISPR knockout screen. Related to Fig 1.

A Western blots and quantification of RBM3, Cas9 and GAPDH in two GFP-RBM3 clones and Cas9 WT iPSCs and i-neurons 4, 7 and 18 days after dox-induced differentiation.
B Representative image of GFP-RBM3 iPSCs. The nucleus and soma are outlined by white and yellow dashed lines, respectively. Scale bar: 5 μm.
C Schematic of the reporter lentivirus design and expected fluorescent protein expression in transduced WT (-Cas9) and Cas9 WT (+Cas9) i-neurons. Transduced WT i-neurons (top grey box) show high levels of BFP and mCherry. Transduced Cas9 WT i-neurons (bottom orange box) that are successfully edited by the mCherry sgRNA express reduced levels of mCherry compared to the unedited ones.
D Representative BFP vs. mCherry plots measured by flow cytometry for measuring editing and transduction efficiency in WT, Cas9 WT, two clones of GFP-RBM3 i-neurons 4 days (Day 8) and 14 days (Day 14) after reporter lentivirus transduction. Region (I), (II) and (III) denote BFP+/mCherry-, BFP+/mCherry+ and BFP-/mCherry- populations, respectively.
E Editing and transduction efficiency of WT, Cas9 WT, two clones of GFP-RBM3 i-neurons at day 8 and 18 post differentiation. The calculation is based on the cell numbers within each area labelled in (D) and the formulas are shown in the graph.
F Median GFP intensity of two GFP-RBM3 clones and Cas9 WT i-neurons at 37°C or at 32°C for 24-72 h, measured by flow cytometry.
G Nuclear and cytoplasmic GFP intensity per unit area in GFP-RBM3 i-neurons at 37 or 32°C (72 h). Each data point represents one cell.
H Western blots and quantification of RBM3 normalised to GAPDH in Cas9 WT i-neurons at 37 or 32°C (72 h).

Data information: $N = 3$ biological replicates, except (E), which has $N = 2$. Mean ± SEM; n.s. (not significant), *($P < 0.05$), ***($P < 0.001$); one-way ANOVA with multiple comparisons in (F), unpaired t-tests in (G) and (H).
Source data are available online for this figure.

© 2023 The Authors

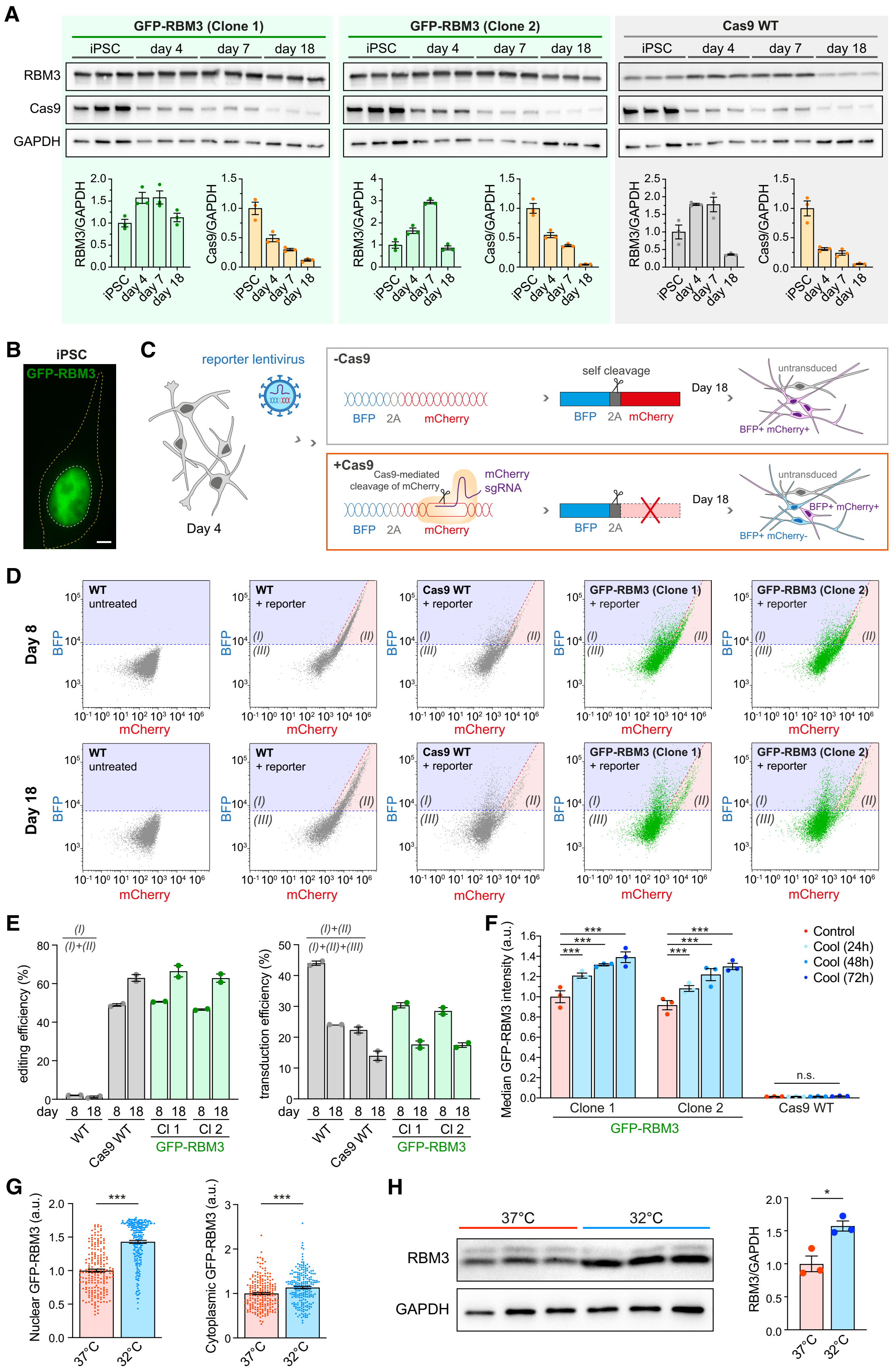

Figure EV1.

 © 2023 The Authors

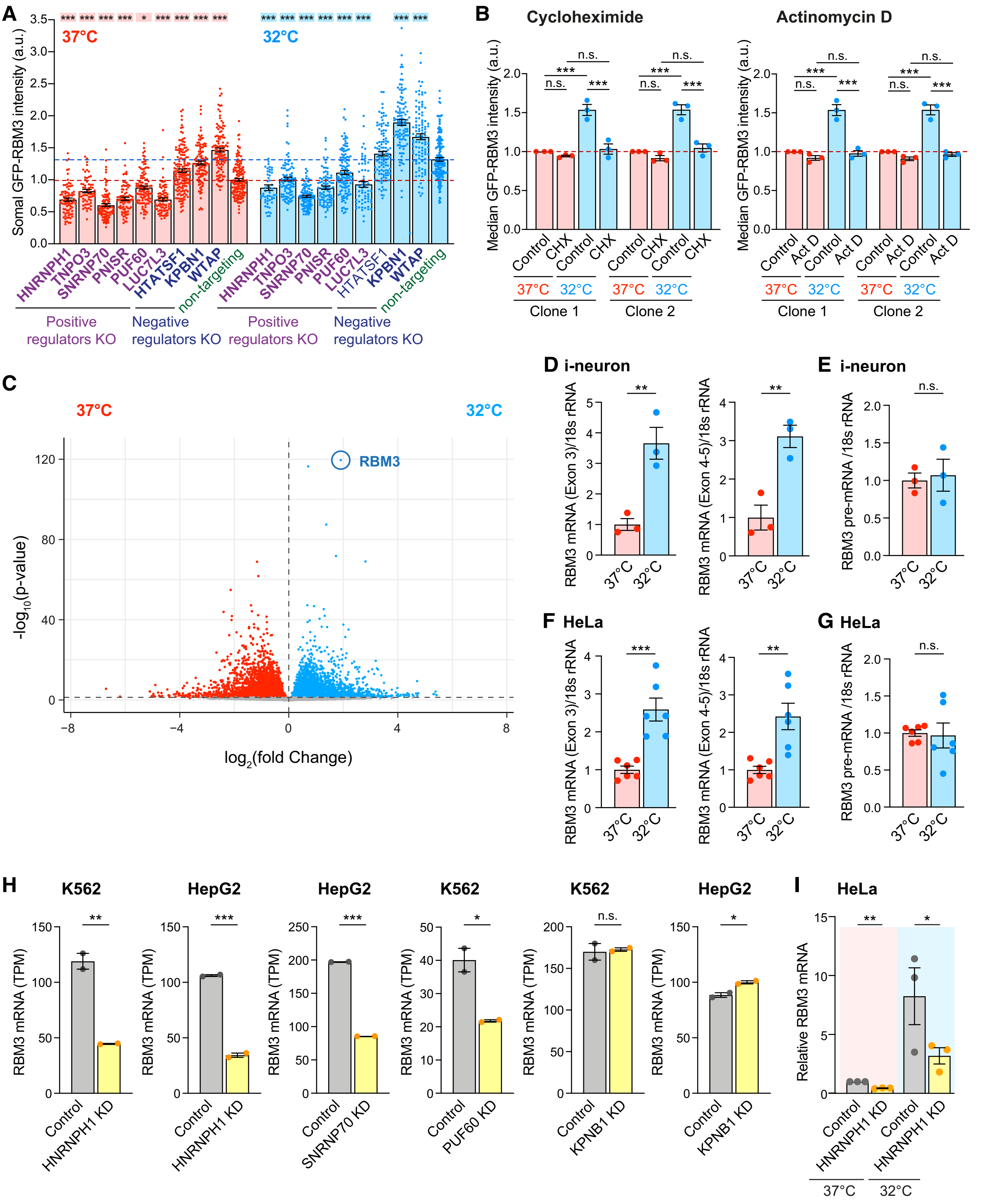
A
37°C
32°C
Somal GFP-RBM3 intensity (a.u.)
HNRNPH1
TNPO3
SNRNP70
PNISR
PUF60
LUC7L3
HTATSF1
KPBN1
WTAP
non-targeting
Positive regulators KO
Negative regulators KO
B
Cycloheximide
Actinomycin D
Median GFP-RBM3 intensity (a.u.)
Control
CHX
Act D
Clone 1
Clone 2
n.s.
C
RBM3
-log10(p-value)
log2(fold Change)
D i-neuron
RBM3 mRNA (Exon 3)/18s rRNA
RBM3 mRNA (Exon 4-5)/18s rRNA
E i-neuron
RBM3 pre-mRNA /18s rRNA
F HeLa
G HeLa
H
K562
HepG2
RBM3 mRNA (TPM)
HNRNPH1 KD
SNRNP70 KD
PUF60 KD
KPNB1 KD
I
HeLa
Relative RBM3 mRNA

Figure EV2.

Figure EV2. Cooling and regulators involved in mRNA splicing change RBM3 transcript levels. Related to Fig 2.

A Somal GFP intensity per unit area in GFP-RBM3 i-neurons transduced with lentivirus containing specific or non-targeting sgRNA at 37 or 32°C (72 h) imaged by wide-field microscopy. Only BFP-positive (transduced) cells are included. Statistical analysis is performed between the specific and non-targeting sgRNA within the temperature groups. Each data point represents one cell.

B Median GFP intensity per unit area of GFP-RBM3 i-neurons at 37 or 32°C (72 h) treated with cycloheximide (CHX) at 50 μM for 72 h or actinomycin D (Act D) at 1 μM for 72 h.

C Volcano plot showing differential expression analysis of all transcripts identified in i-neurons at 37 and 32°C (72 h) from RNA-Seq data.

D, E qRT-PCR of RBM3 Exon 3, Exon 4–5 (D) and pre-mRNA (E) normalised to 18 s rRNA in i-neurons at 37 and 32°C (72 h).

F, G qRT-PCR of RBM3 Exon 3, Exon 4–5 (F) and pre-mRNA (G) normalised to 18 s rRNA in HeLa cells at 37 and 32°C (48 h).

H Normalised RBM3 mRNA abundance (TPM) of control and selective regulator candidates knocked-down K562 or HepG2 cells. Data are extracted from ENCODE project. 2 isogenic replicates are included in each condition.

I qRT-PCR of RBM3 mRNA normalised to GAPDH in control and HNRNPH1 KD HeLa cells at 37 or 32°C (48 h).

Data information: $N = 3$ biological replicates. Mean ± SEM; n.s. (not significant), *($P < 0.05$), **($P < 0.01$), ***($P < 0.001$); one-way ANOVA with multiple comparisons in (A), unpaired t-tests in (B), (D)-(H); paired t-test in (I).
Source data are available online for this figure.

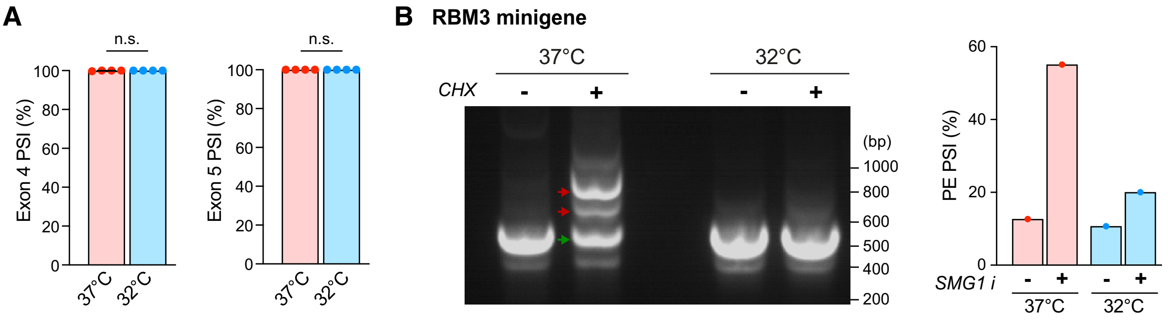

Figure EV3. Cooling represses RBM3 poison exon inclusion. Related to Fig 3.

A PSI values of RBM3 Exon 4 relative to Exon 3 and 6, and Exon 5 relative to Exon 4 and 6 in i-neurons at 37 or 32°C (72 h). $N = 4$ biological replicates.

B RT-PCR of RBM3 minigene expressed in HeLa cells at 37 and 32°C (48 h) in the presence or absence of cycloheximide (CHX) at 200 μg/ml concentration. PSI values of RBM3 PE are calculated based on the intensity of PE-included (red arrows) and PE-skipped (green arrow) isoforms. $N = 1$ biological replicate.

Data information: Mean ± SEM; n.s. (not significant); FDR calculated by rMATS program in (A).
Source data are available online for this figure.

Figure EV4. HNRNPH1 enhances RBM3 expression and RBM3 mRNA poison exon skipping on cooling. Related to Fig 4.

A Western blots and quantification of HNRNPH1 normalised to GAPDH in Control and HNRNPH1 KD Cas9WT i-neurons.

B Sashimi plots of the region between Exon 3 and 4 of RBM3 transcripts in control and HNRNPH1 knocked-down K562 and HepG2 cells, showing major alternatively spliced isoforms. Data are from ENCODE Project. 2 isogenic replicates are included.

C PSI values of RBM3 Exon 3a-S in control and HNRNPH1-knocked down K562 and HepG2 cells. RNA-Seq data from ENCODE Project, 2 isogenic replicates are included.

D qRT-PCR of HNRNPH1 mRNA normalised to GAPDH upon HNRNPH1 KD in HeLa cells.

E Western blots of FLAG-HNRNPH1 and GAPDH (loading control) in control and FLAG-HNRNPH1-expressed HEK293T cells at 37°C.

F Western blot of WT i-neurons transduced with lentivirus expressing BFP (control) or HNRNPH1-T2A-BFP (OX) at 37 and 32°C. The larger molecular weight of overexpressed HNRNPH1 is due to the additional amino acids between the C-terminus of HNRNPH1 and the T2A cleavage site.

G qRT-PCR of HNRNPH1 mRNA normalised to 18 s rRNA of WT i-neurons transduced with lentivirus expressing BFP (control) or HNRNPH1-T2A-BFP (OX) at 37 and 32°C.

H qRT-PCR quantifying the PSI values of RBM3 PE relative to RBM3 mRNA (mean value of RBM3 exon 3 and exon 4–5) in SMG1 inhibitor-treated control and HNRNPH1-overexpressing (OX) WT i-neurons at 37 and 32°C.

I Median GFP intensity of control and HNRNPH1-overexpressing (OX) GFP-RBM3 i-neurons measured by flow cytometry.

J Representative dot plots of flow cytometry data showing BFP or HNRNPH1-T2A-BFP expression (X-axis) and GFP intensity of successfully transduced (BFP-positive) GFP-RBM3 i-neurons. Each data point represents one cell. Cells with high levels of BFP expression (top 5% in each well) are coloured in red (37°C) or blue (32°C), and the rest of BFP-positive cells are in grey. Y-axes are in the same scale and X-axes are scaled to the sample.

Data information: $N = 3$ biological replicates. Mean ± SEM; n.s. (not significant), *($P < 0.05$), **($P < 0.01$), ***($P < 0.001$); unpaired t-tests in (A), (G), (H), (I), FDR calculated by rMATS program in (C), paired t-test in (D).
Source data are available online for this figure.

 © 2023 The Authors

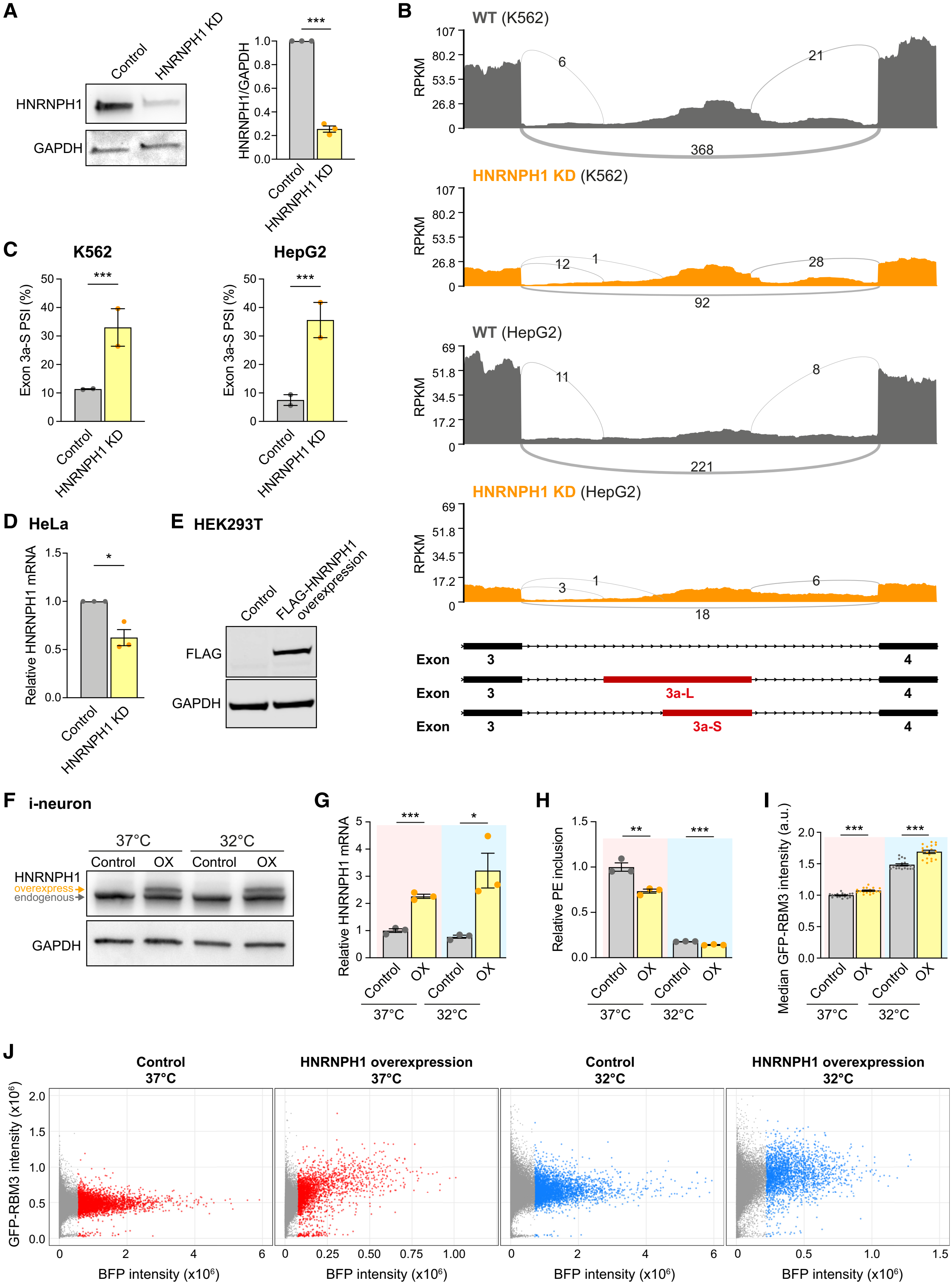
A
Control
HNRNPH1 KD
HNRNPH1
GAPDH
HNRNPH1/GAPDH
***
B
WT (K562)
HNRNPH1 KD (K562)
WT (HepG2)
HNRNPH1 KD (HepG2)
RPKM
Exon 3
Exon 3 3a-L 4
Exon 3 3a-S 4
C
K562
HepG2
Exon 3a-S PSI (%)
D HeLa
Relative HNRNPH1 mRNA
E HEK293T
FLAG-HNRNPH1 overexpression
FLAG
GAPDH
F i-neuron
37°C
32°C
Control
OX
HNRNPH1
overexpress
endogenous
GAPDH
G
Relative HNRNPH1 mRNA
H
Relative PE inclusion
I
Median GFP-RBM3 intensity (a.u.)
J
Control 37°C
HNRNPH1 overexpression 37°C
Control 32°C
HNRNPH1 overexpression 32°C
GFP-RBM3 intensity (x10⁶)
BFP intensity (x10⁶)

Figure EV4.

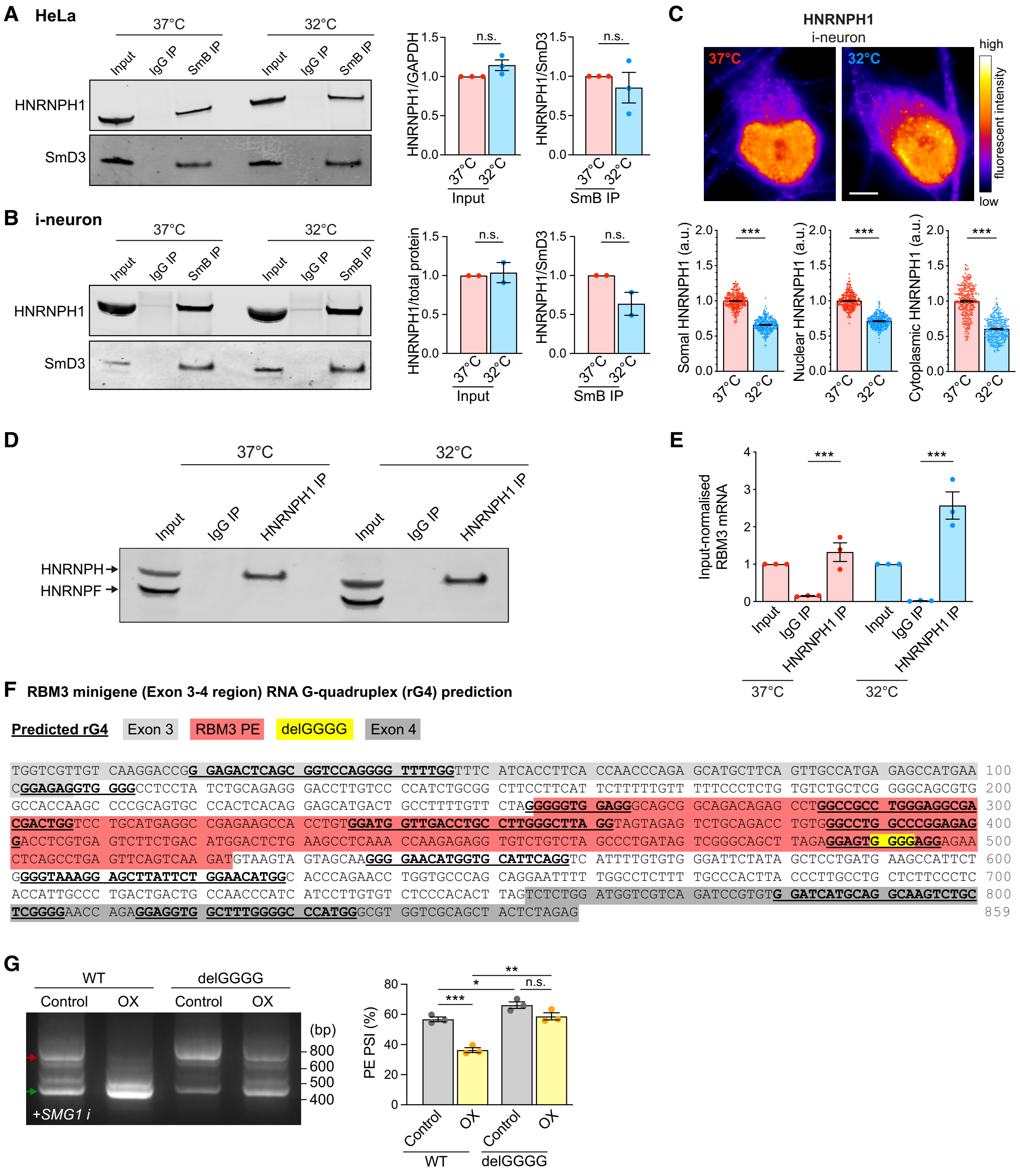

Figure EV5.

© 2023 The Authors

Figure EV5. HNRNPH1 interacts with G-rich sequences in RBM3 poison exon in a temperature-dependent manner. Related to Fig 5.

A Western blot and quantification of HNRNPH1 total protein levels (input HNRNPH1 normalised to GAPDH) and its abundance in spliceosomal protein SmB pulldown (normalised to spliceosomal protein SmD3) in HeLa cells at 37 and 32°C (72 h).

B Western blot and quantification of HNRNPH1 total protein levels (input HNRNPH1 normalised to ponceau measured total protein abundance) and its abundance in spliceosomal protein SmB pulldown (normalised to spliceosomal protein SmD3) in i-neurons at 37 and 32°C (72 h).

C Representative images of HNRNPH1 staining of GFP-RBM3 i-neurons at 37°C or after 72 h cooling at 32°C. Graphs below the images show quantification of somal, nuclear and cytoplasmic intensity per unit area respectively. The decrease of HNRNPH1 signals at 32°C is due to the global attenuation of protein production, which is not seen after total protein or GAPDH normalisation in western blot in (B) and Fig 4A. $N = 280$ (37°C) and 262 (32°C) cells. Scale bar: 5 μm.

D Western blot of HNRNPH/F in HeLa cell input (total) lysate, IgG-pulled down and HNRNPH1-pulled down eluates at 37 and 32°C (48 h).

E Quantification of input-normalised RBM3 mRNA levels in HeLa cell input (total), IgG-pulled down and HNRNPH1-pulled down RNA at 37 and 32°C (48 h).

F RNA G quadruplexes (rG4) within the RBM3 Exon 3–4 region are predicted using QGRS mapper. Deletion of the GGGG motif in the mutant RBM3 minigene is predicted to disrupt the rG4 structure overlapping this region.

G RT-PCR of WT and delGGGG RBM3 minigenes in control or HNRNPH1-overexpressing (OX) HEK293T cells treated with SMG1 inhibitor at 37°C. PSI values of RBM3 PE are shown in the graphs on the right.

Data information: $N = 3$ biological replicates, except (B), which has $N = 2$. Mean ± SEM; n.s. (not significant), *($P < 0.05$), **($P < 0.01$), ***($P < 0.001$); paired t-test in (A), (B), (E), (G), unpaired t-tests in (C).
Source data are available online for this figure.

© 2023 The Authors
