# Supplementary figures and images for: HNRNPH1 regulates the neuroprotective cold‐shock protein RBM3 expression through poison exon exclusion (part 1 of 3)
Source: EMBO J. 2023 May 30;42(14):e113168. doi: 10.15252/embj.2022113168 (PMC10350819; doi:10.15252/embj.2022113168)

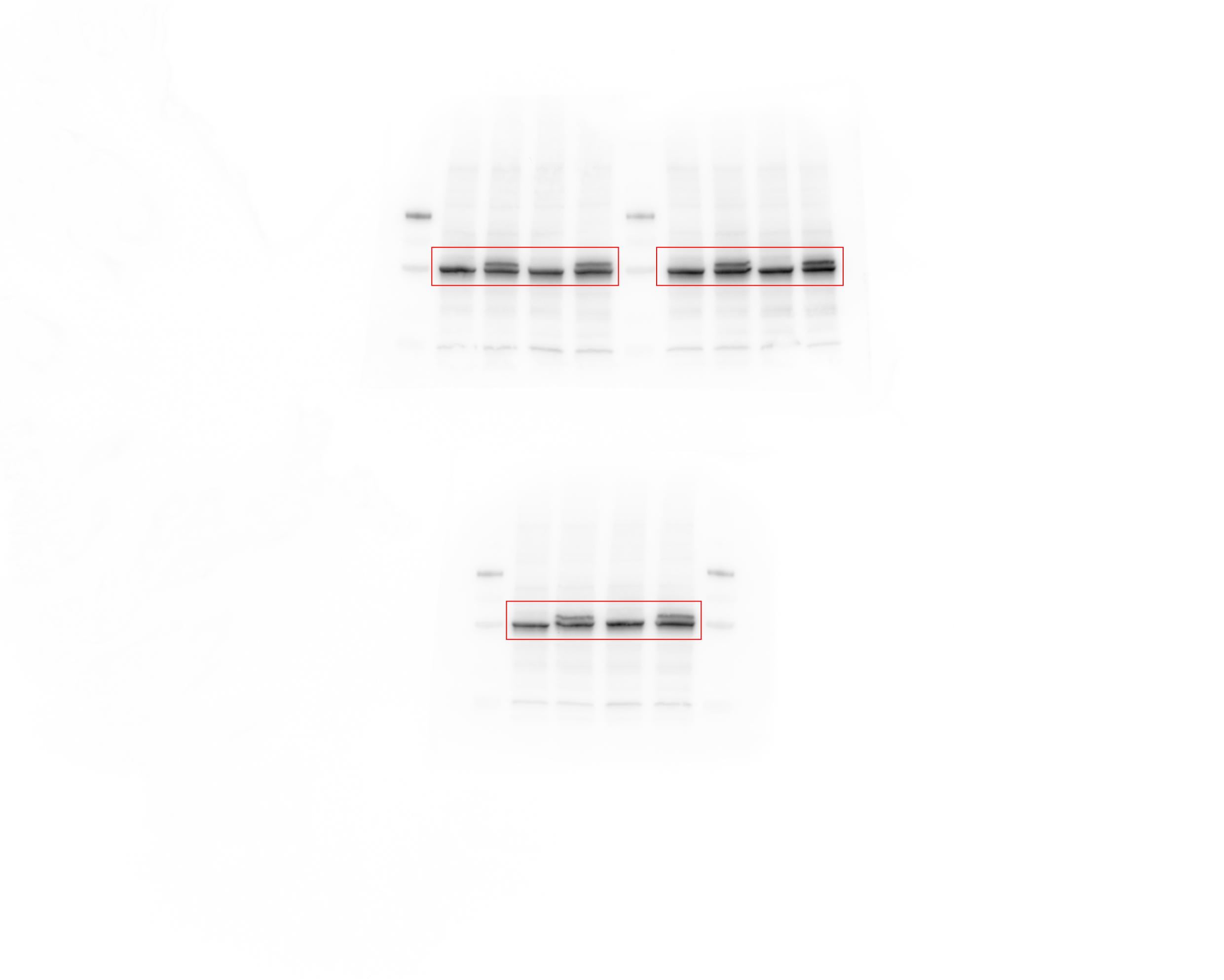

Supplement: Supplementary file 3 — Source Data for Expanded View [file EMBJ-42-e113168-s009.zip › Source data EV1-EV5/Figure EV4/EV4F/western HNRNPH1.tif]

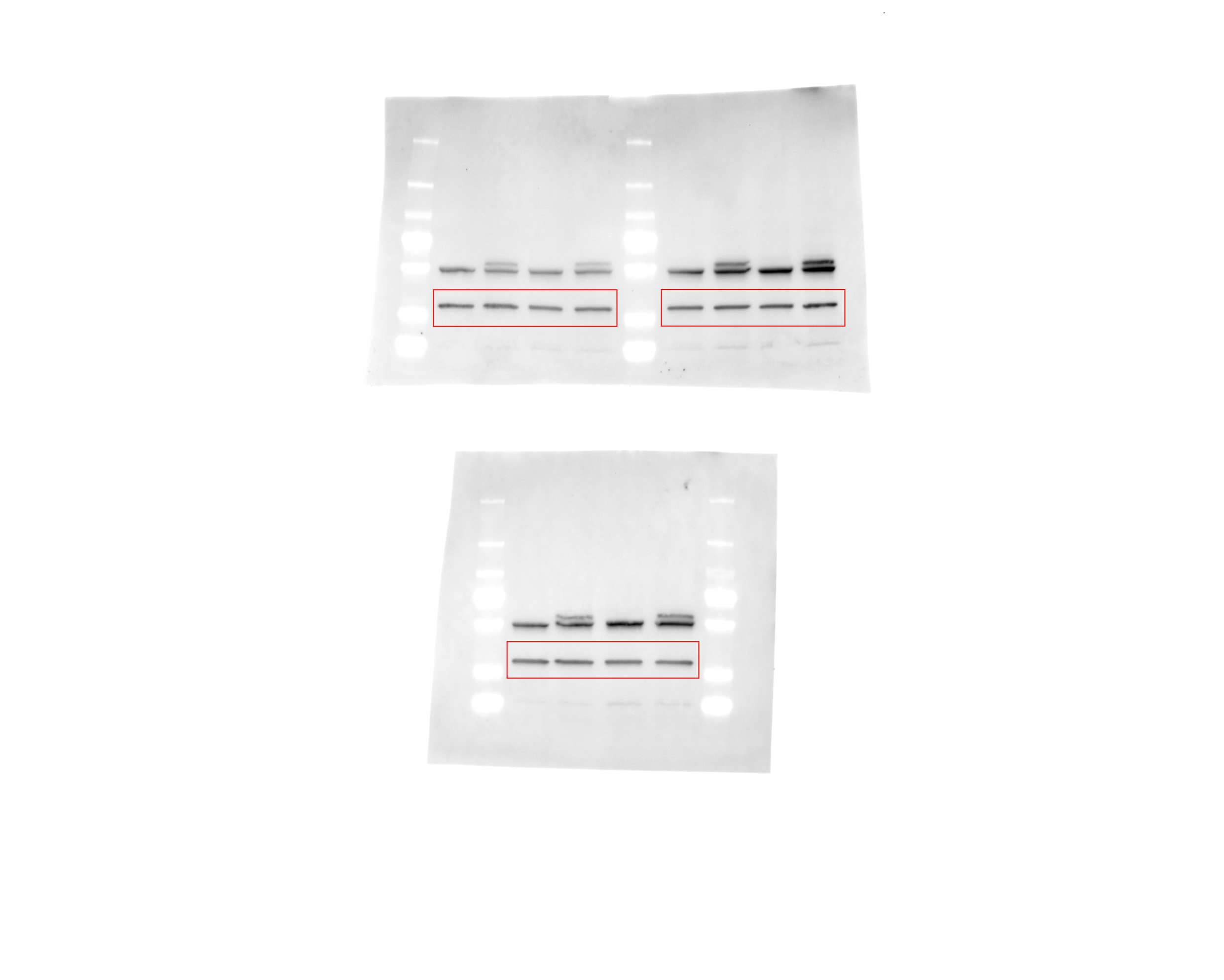

Supplement: Supplementary file 3 — Source Data for Expanded View [file EMBJ-42-e113168-s009.zip › Source data EV1-EV5/Figure EV4/EV4F/western GAPDH.tif]

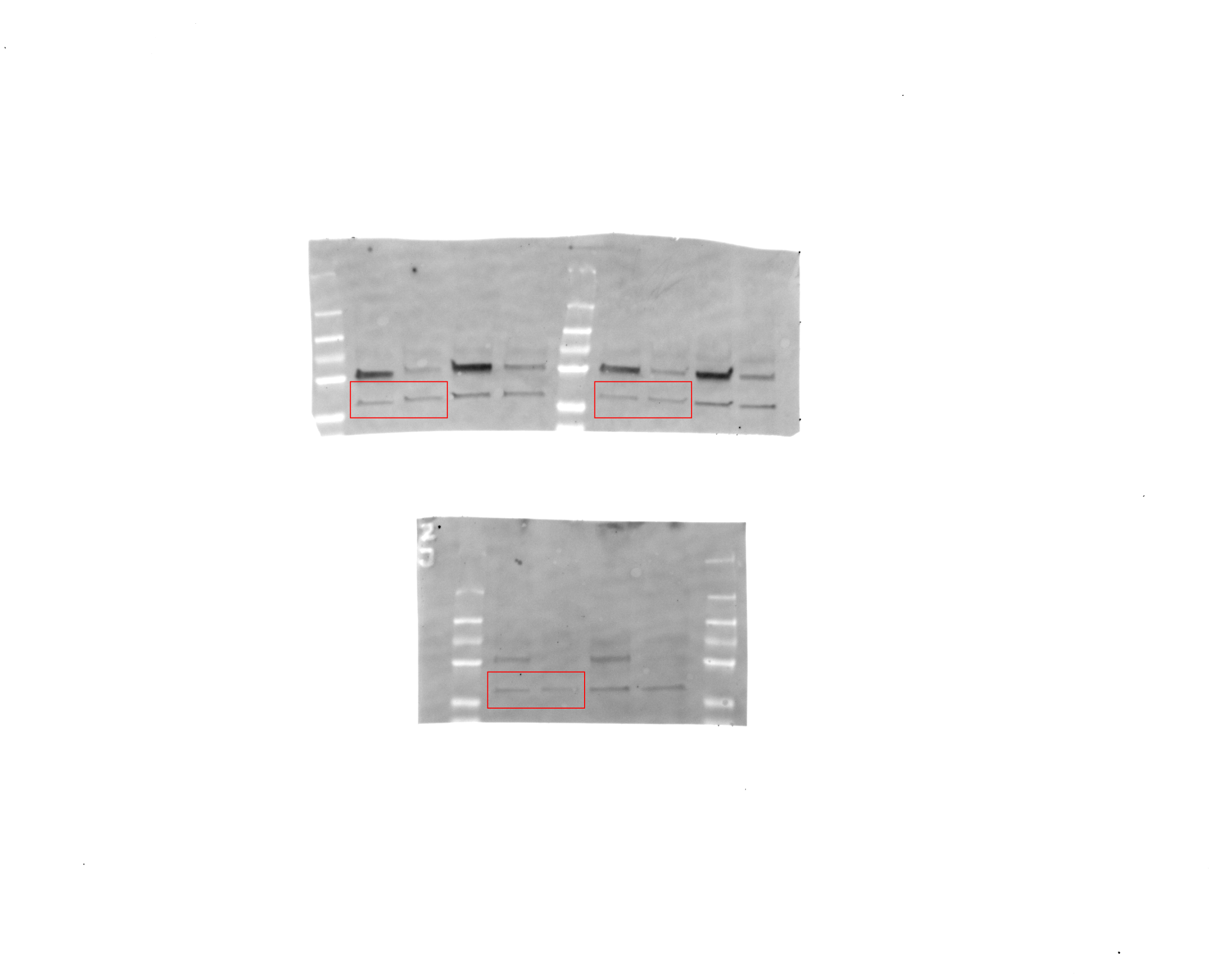

Supplement: Supplementary file 3 — Source Data for Expanded View [file EMBJ-42-e113168-s009.zip › Source data EV1-EV5/Figure EV4/EV4A/western_anti-GAPDH_Set1-3.tif]

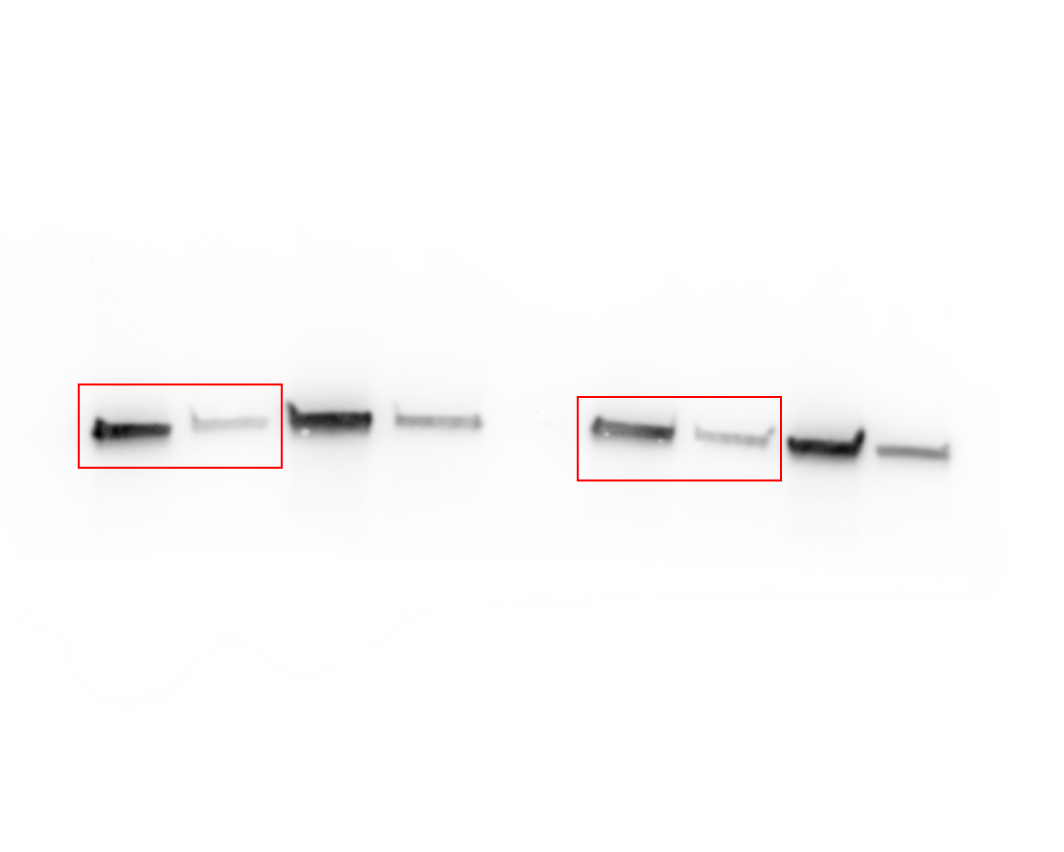

Supplement: Supplementary file 3 — Source Data for Expanded View [file EMBJ-42-e113168-s009.zip › Source data EV1-EV5/Figure EV4/EV4A/western_anti-HNRNPH1_Set1-2.tif]

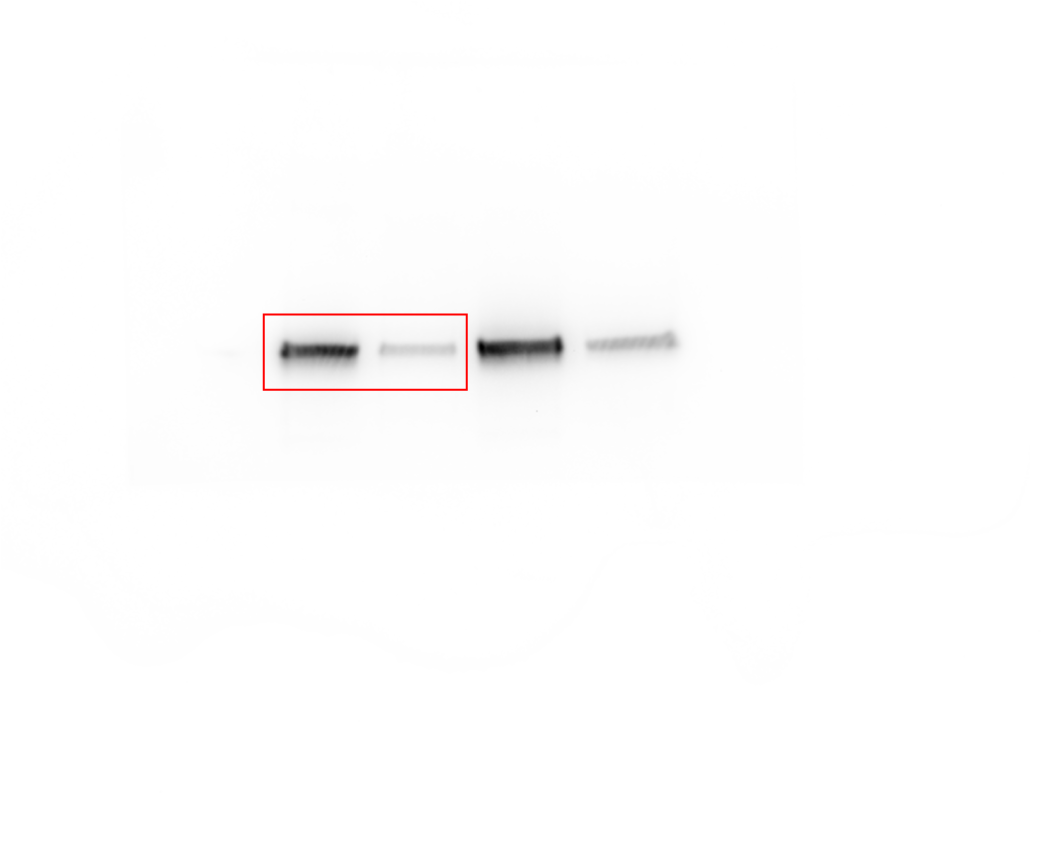

Supplement: Supplementary file 3 — Source Data for Expanded View [file EMBJ-42-e113168-s009.zip › Source data EV1-EV5/Figure EV4/EV4A/western_anti-HNRNPH1_Set3.tif]

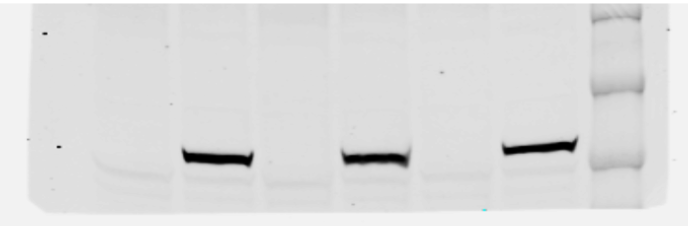

Supplement: Supplementary file 3 — Source Data for Expanded View [file EMBJ-42-e113168-s009.zip › Source data EV1-EV5/Figure EV4/EV4E/WB FLAG HNRNPH1 Sets 1 2 3.png]

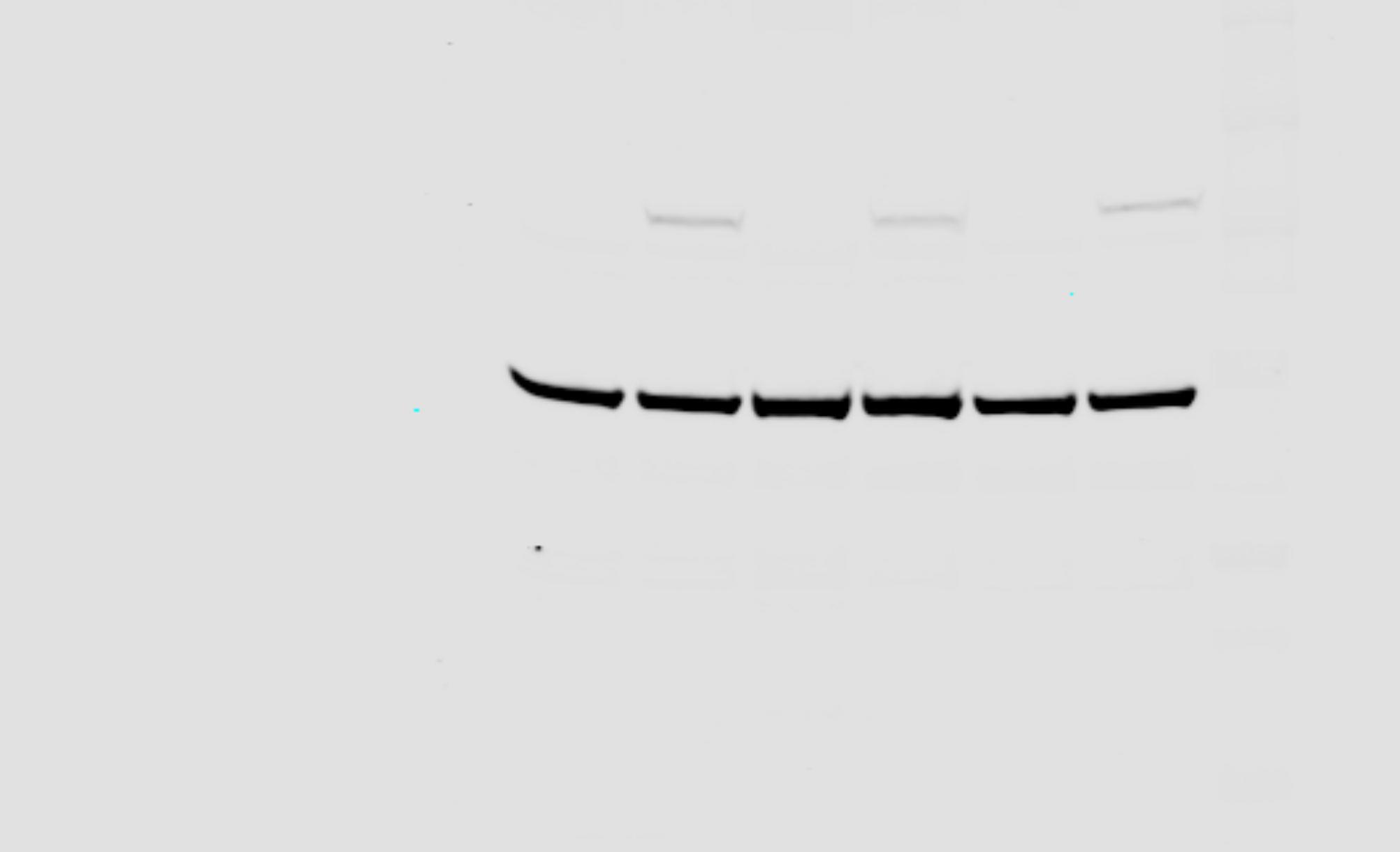

Supplement: Supplementary file 3 — Source Data for Expanded View [file EMBJ-42-e113168-s009.zip › Source data EV1-EV5/Figure EV4/EV4E/WB GAPDH Sets 1 2 3.tif]

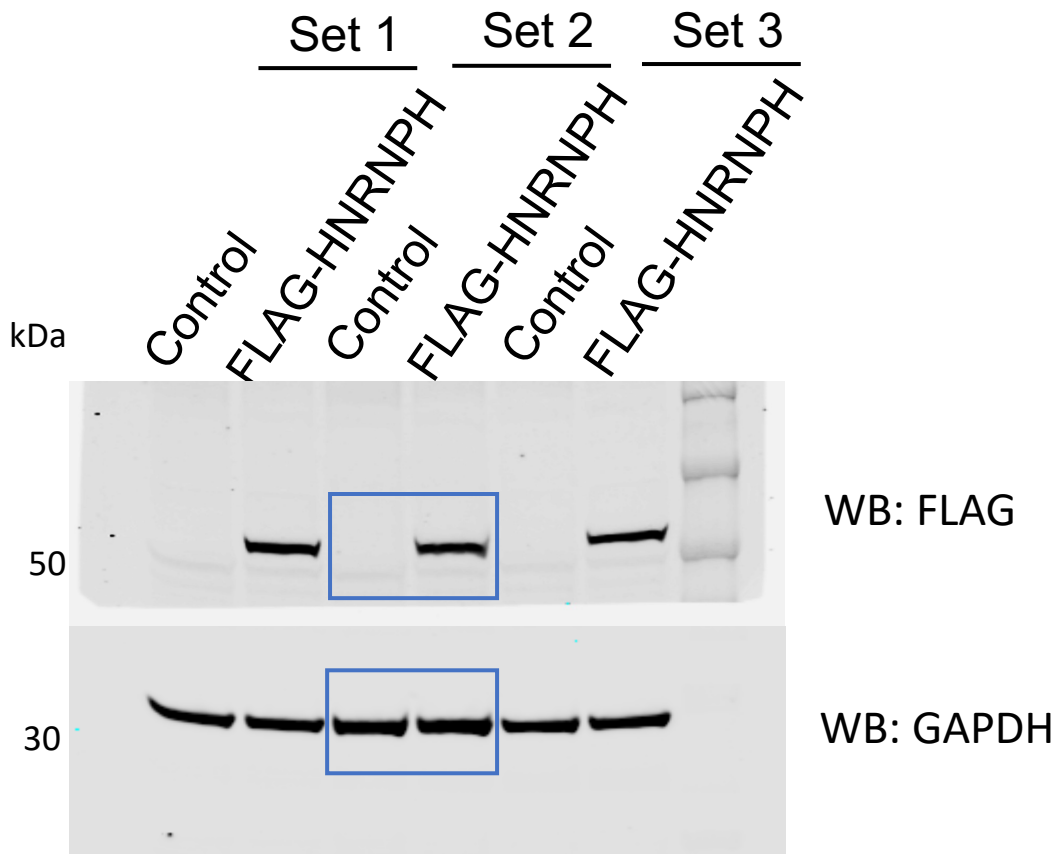

Supplement: Supplementary file 3 — Source Data for Expanded View [file EMBJ-42-e113168-s009.zip › Source data EV1-EV5/Figure EV4/EV4E/Labelled Western (WB) for FLAG and GAPDH sets 1 2 3 used in the figure.pdf]

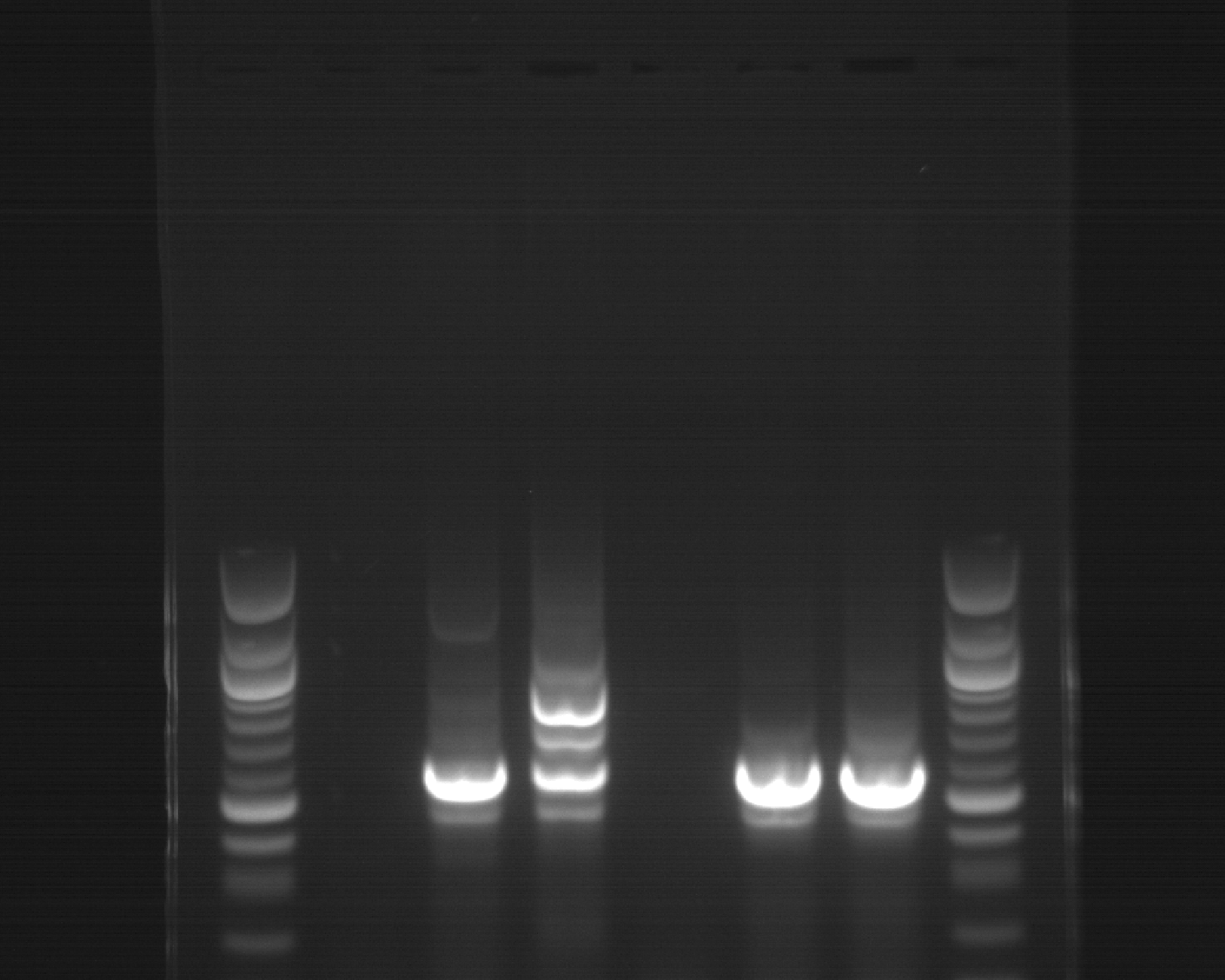

Supplement: Supplementary file 3 — Source Data for Expanded View [file EMBJ-42-e113168-s009.zip › Source data EV1-EV5/Figure EV3/EV3B/RBM3 minigene Hela CHX set 1.png]

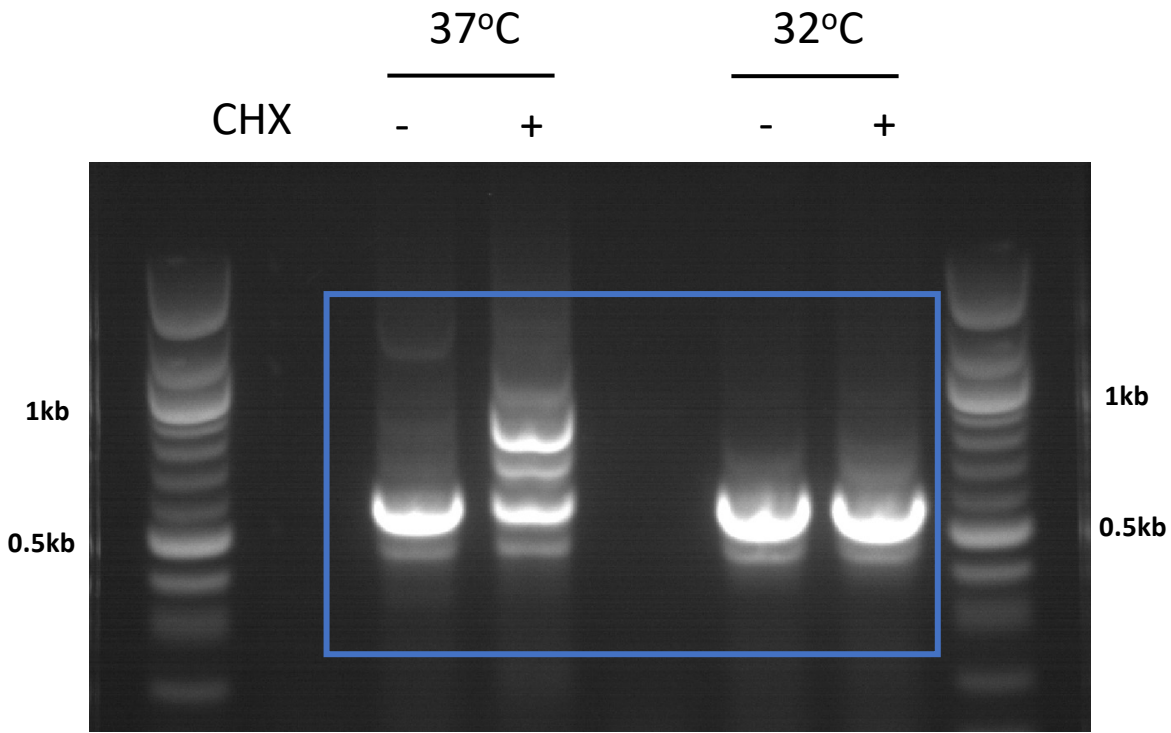

Supplement: Supplementary file 3 — Source Data for Expanded View [file EMBJ-42-e113168-s009.zip › Source data EV1-EV5/Figure EV3/EV3B/Labelled RBM3 minigene Hela CHX set 1 Used in the figure.pdf]

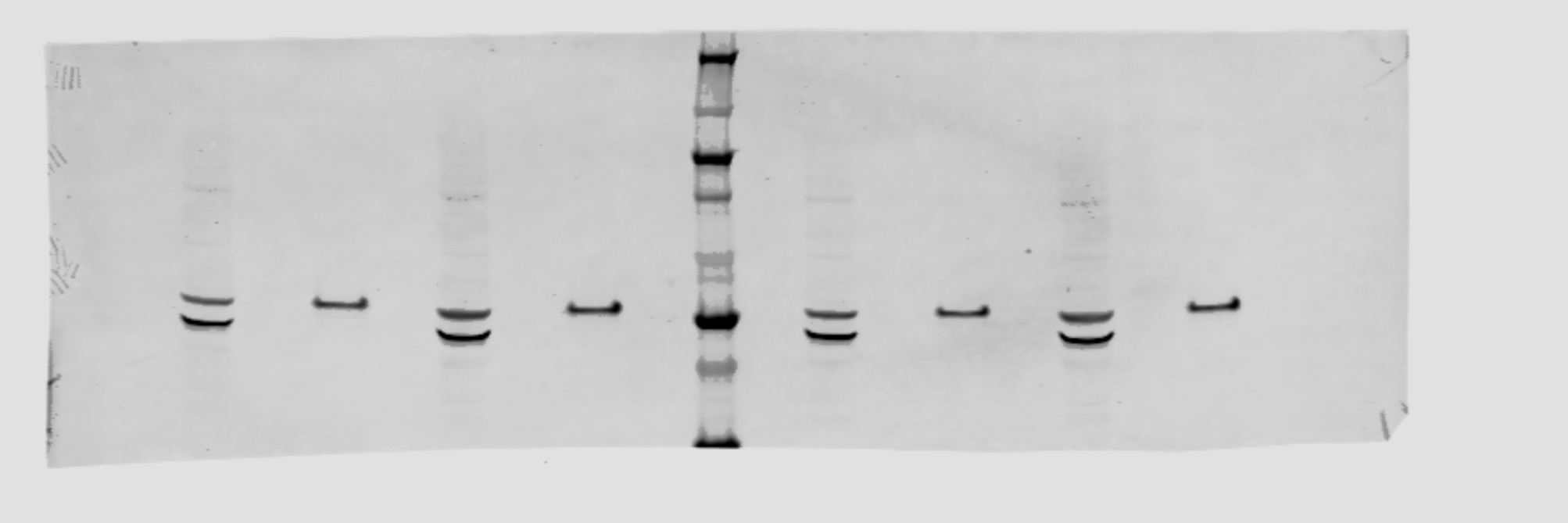

Supplement: Supplementary file 3 — Source Data for Expanded View [file EMBJ-42-e113168-s009.zip › Source data EV1-EV5/Figure EV5/EV5D EV5E/RIP WB HNRNPH set 2 and 3 Used in the figure.tif]

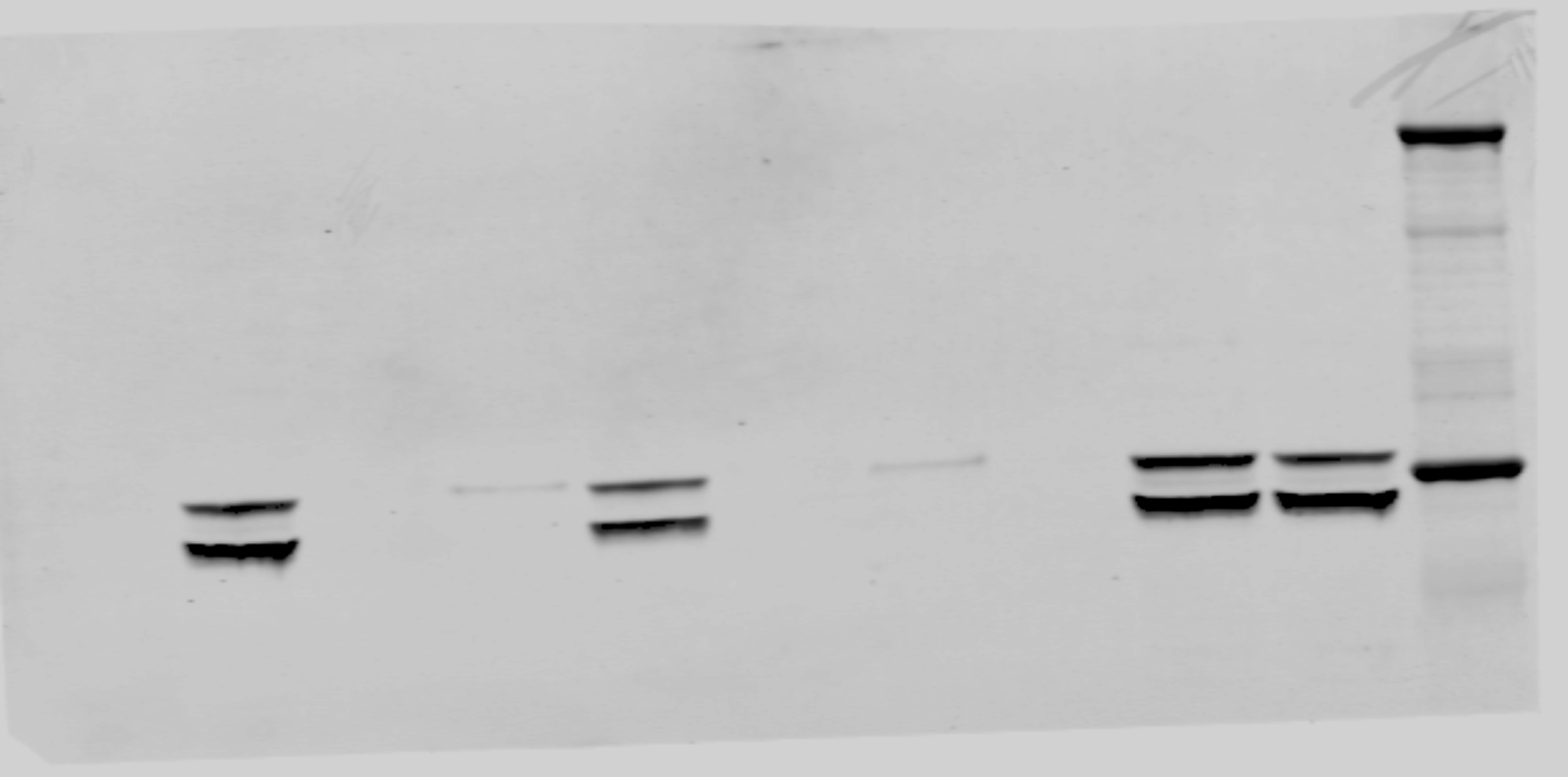

Supplement: Supplementary file 3 — Source Data for Expanded View [file EMBJ-42-e113168-s009.zip › Source data EV1-EV5/Figure EV5/EV5D EV5E/RIP WB HNRNPH1 set 1.tif]

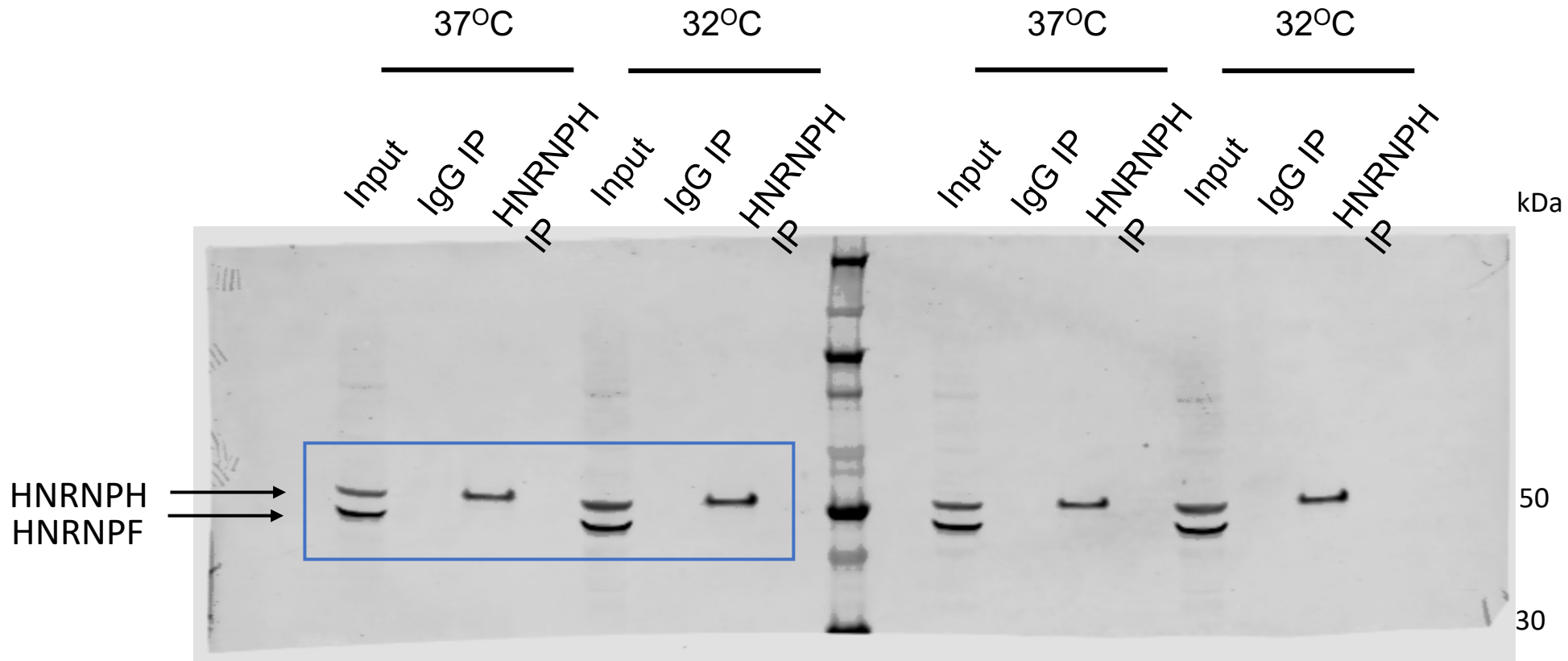

Supplement: Supplementary file 3 — Source Data for Expanded View [file EMBJ-42-e113168-s009.zip › Source data EV1-EV5/Figure EV5/EV5D EV5E/Labelled RIP WB HNRNPH set 2 and set 3 Used in the figure.pdf]

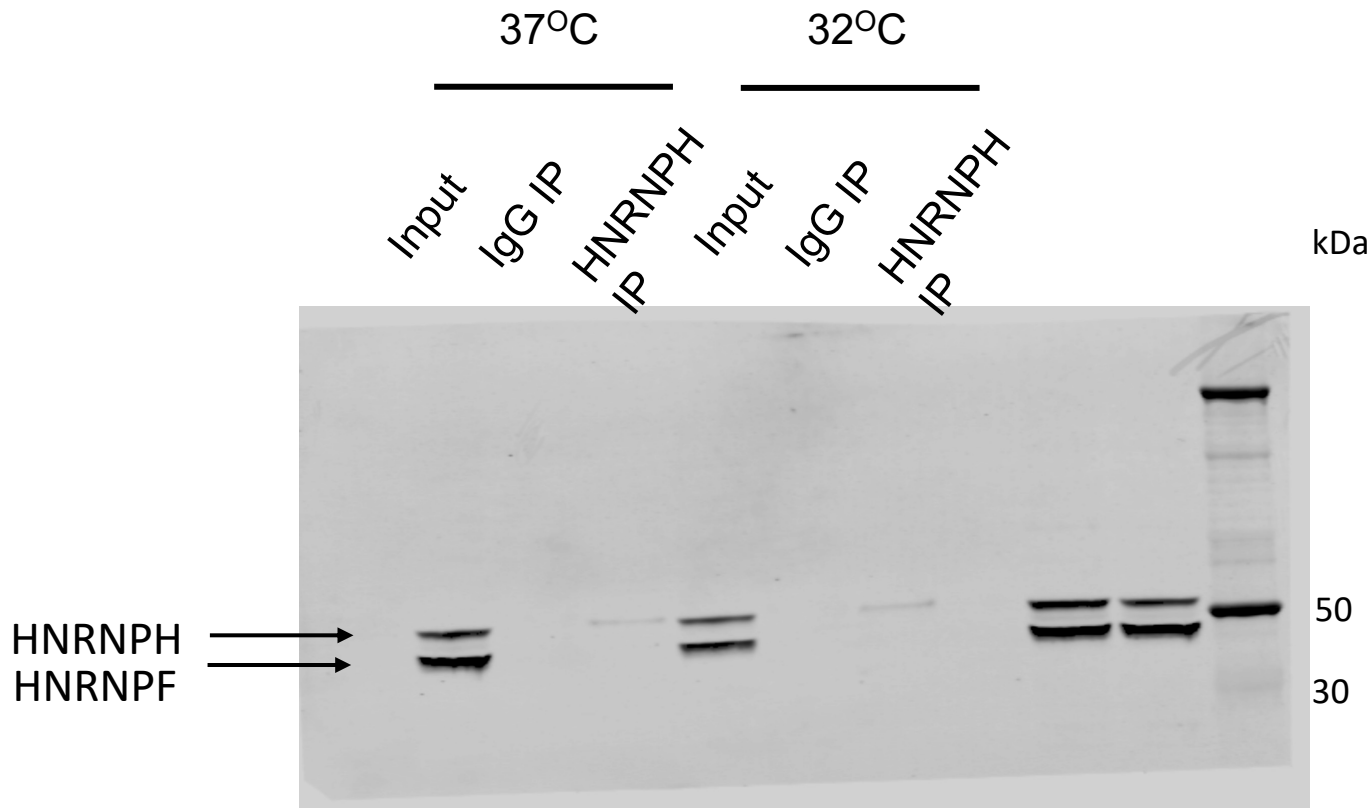

Supplement: Supplementary file 3 — Source Data for Expanded View [file EMBJ-42-e113168-s009.zip › Source data EV1-EV5/Figure EV5/EV5D EV5E/Labelled RIP WB HNRNPH set 1.pdf]

Set 1

WT

delGGGG

Control

FLAG-HNRNPH

Control

FLAG-HNRNPH

SMG1i

-

-

+

+

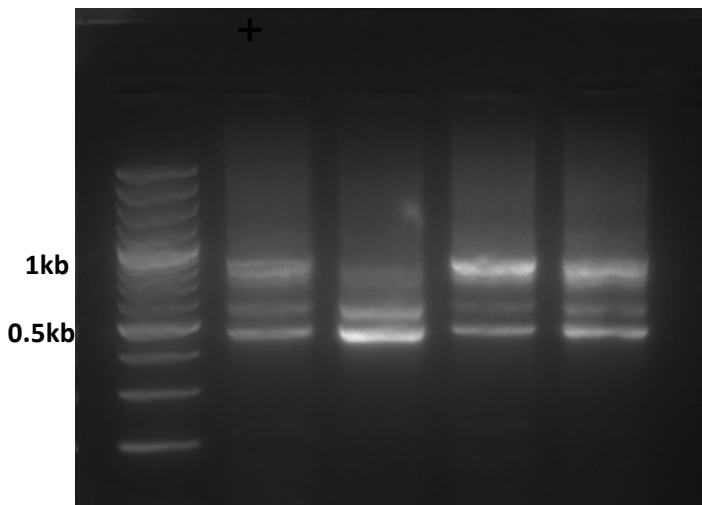

Supplement: Supplementary file 3 — Source Data for Expanded View [file EMBJ-42-e113168-s009.zip › Source data EV1-EV5/Figure EV5/EV5G/Labelled HNRNPH OX Minigene WT delGGGG set 1.pdf]

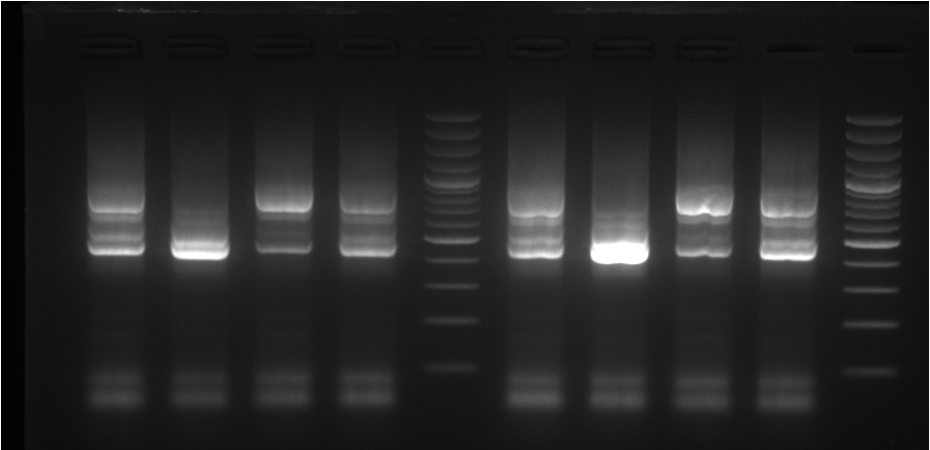

Supplement: Supplementary file 3 — Source Data for Expanded View [file EMBJ-42-e113168-s009.zip › Source data EV1-EV5/Figure EV5/EV5G/HNRNPH OX Minigene WT delGGGG set 2 and 3 Used in the figure.png]

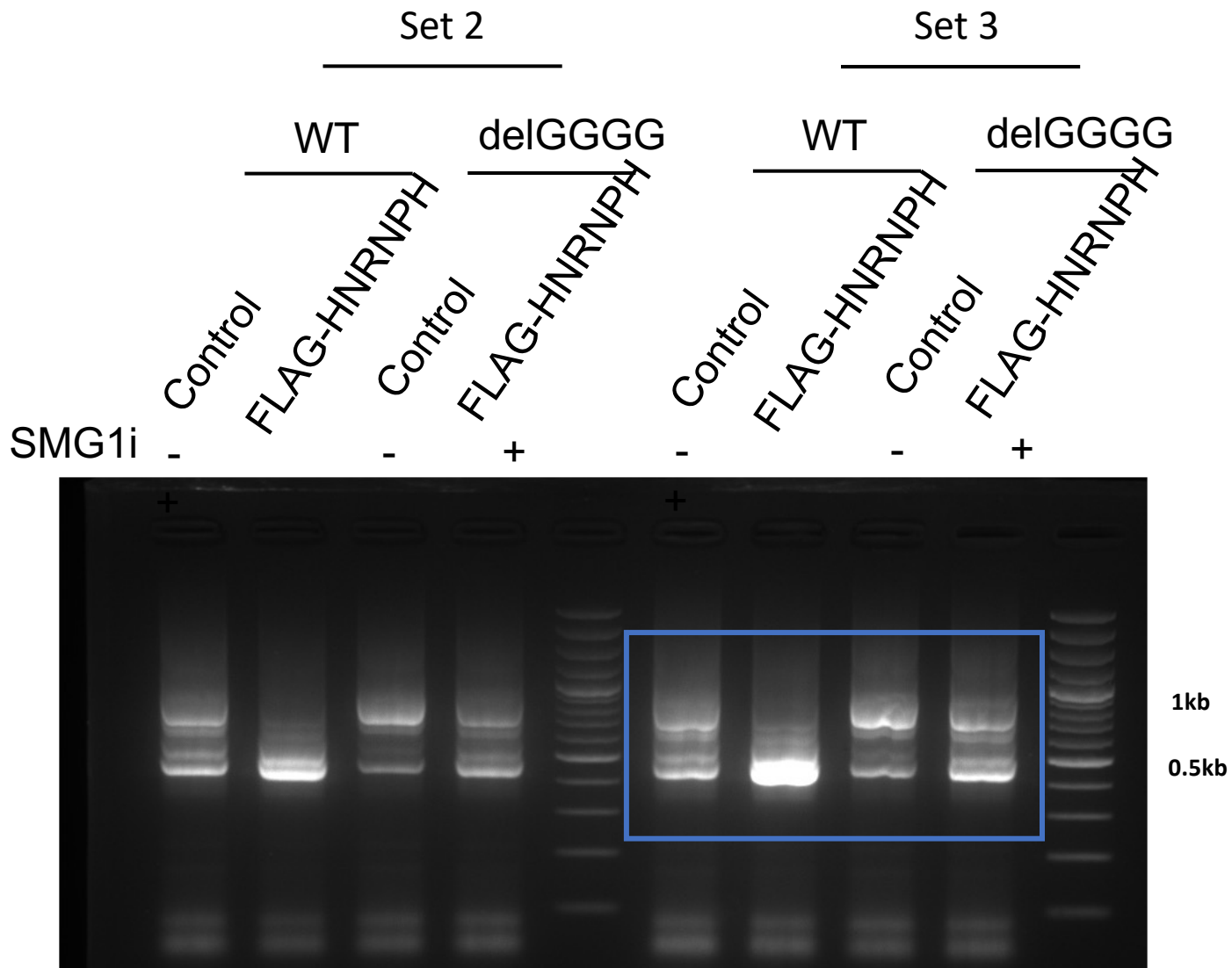

Supplement: Supplementary file 3 — Source Data for Expanded View [file EMBJ-42-e113168-s009.zip › Source data EV1-EV5/Figure EV5/EV5G/Labelled HNRNPH OX Minigene WT delGGGG set 2 and 3 Used in the figure.pdf]

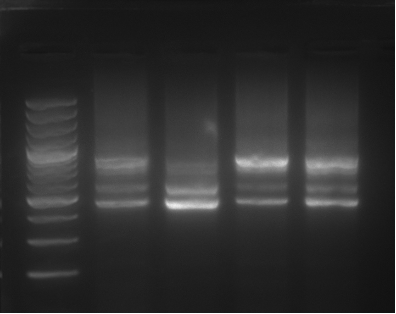

Supplement: Supplementary file 3 — Source Data for Expanded View [file EMBJ-42-e113168-s009.zip › Source data EV1-EV5/Figure EV5/EV5G/HNRNPH OX Minigene WT delGGGG set 1.png]

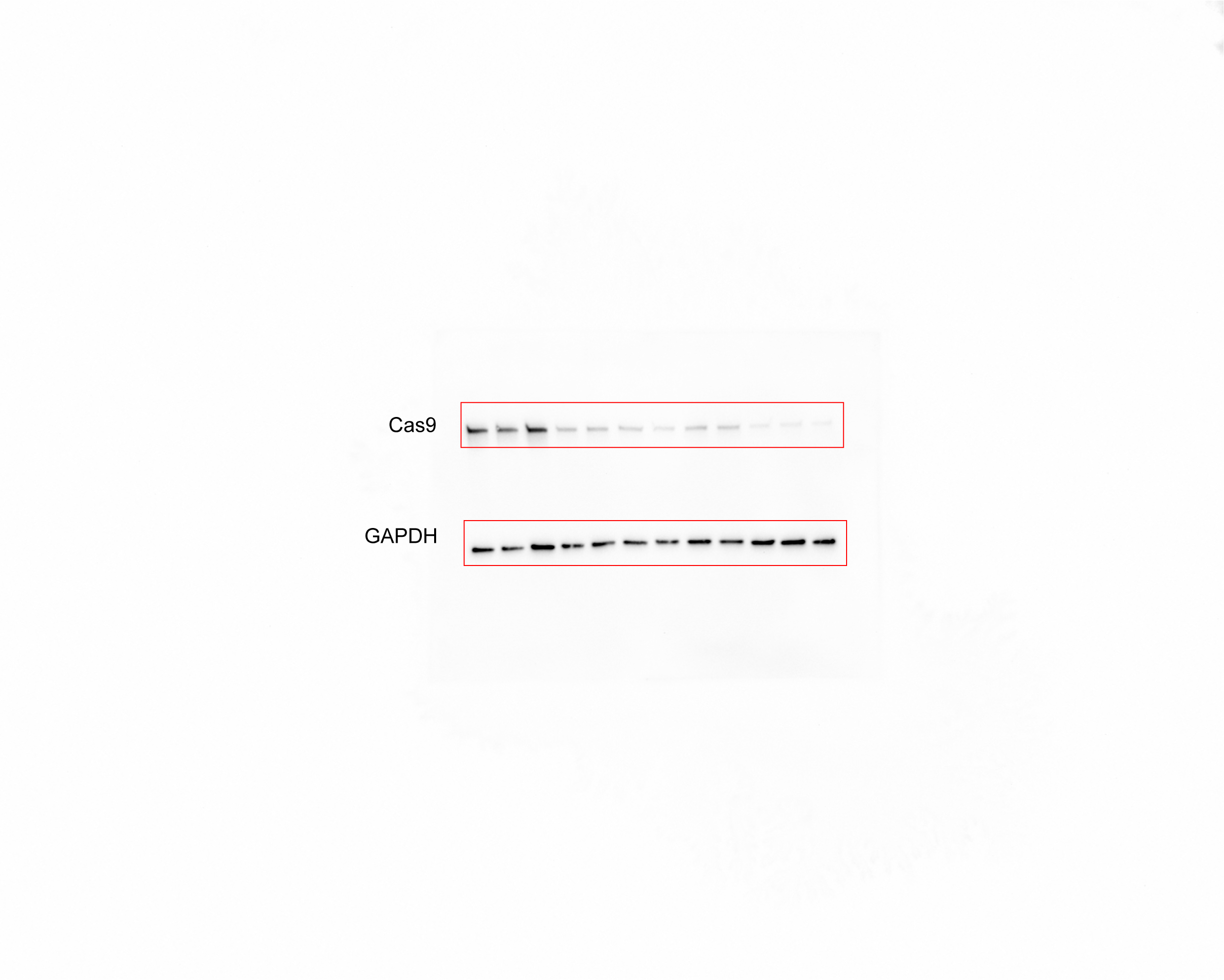

Supplement: Supplementary file 3 — Source Data for Expanded View [file EMBJ-42-e113168-s009.zip › Source data EV1-EV5/Figure EV1/EV1A/western_Cas9WT i-neuron_anti-Cas9 GAPDH.tif]

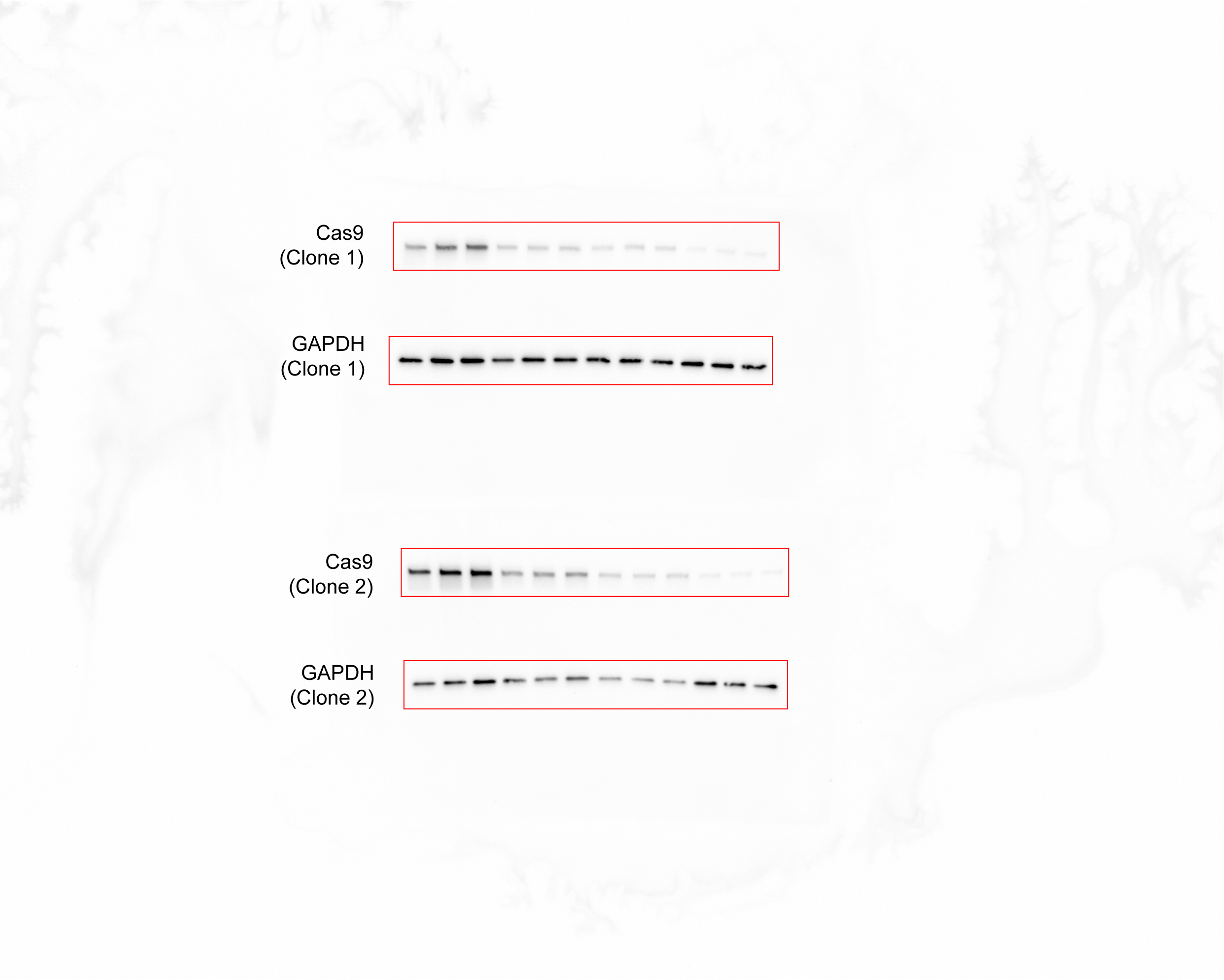

Supplement: Supplementary file 3 — Source Data for Expanded View [file EMBJ-42-e113168-s009.zip › Source data EV1-EV5/Figure EV1/EV1A/western_GFP-RBM3 i-neuron_anti-Cas9 GAPDH.tif]

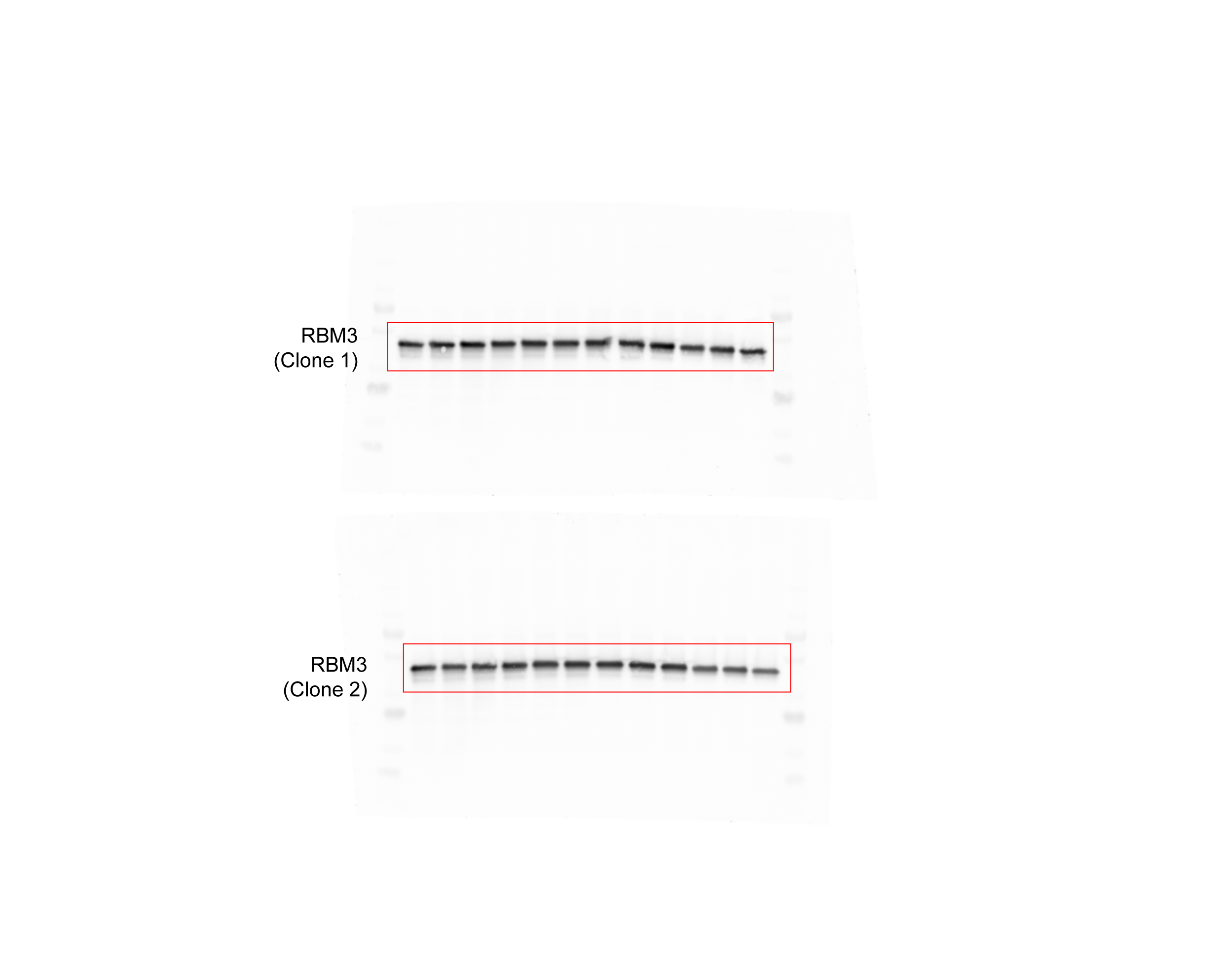

Supplement: Supplementary file 3 — Source Data for Expanded View [file EMBJ-42-e113168-s009.zip › Source data EV1-EV5/Figure EV1/EV1A/western_GFP-RBM3 i-neuron_anti-RBM3.tif]

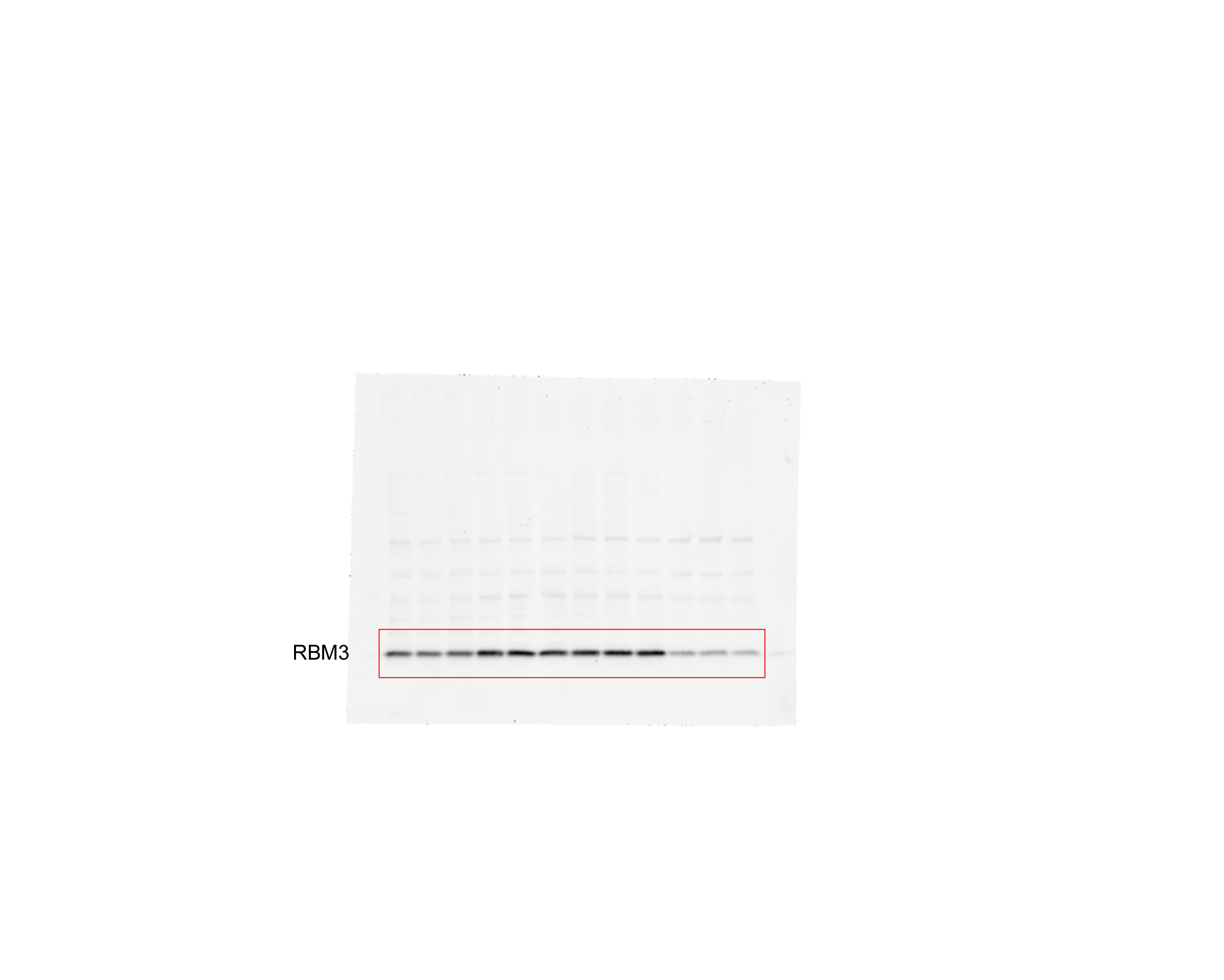

Supplement: Supplementary file 3 — Source Data for Expanded View [file EMBJ-42-e113168-s009.zip › Source data EV1-EV5/Figure EV1/EV1A/western_Cas9WT i-neuron_anti-RBM3.tif]

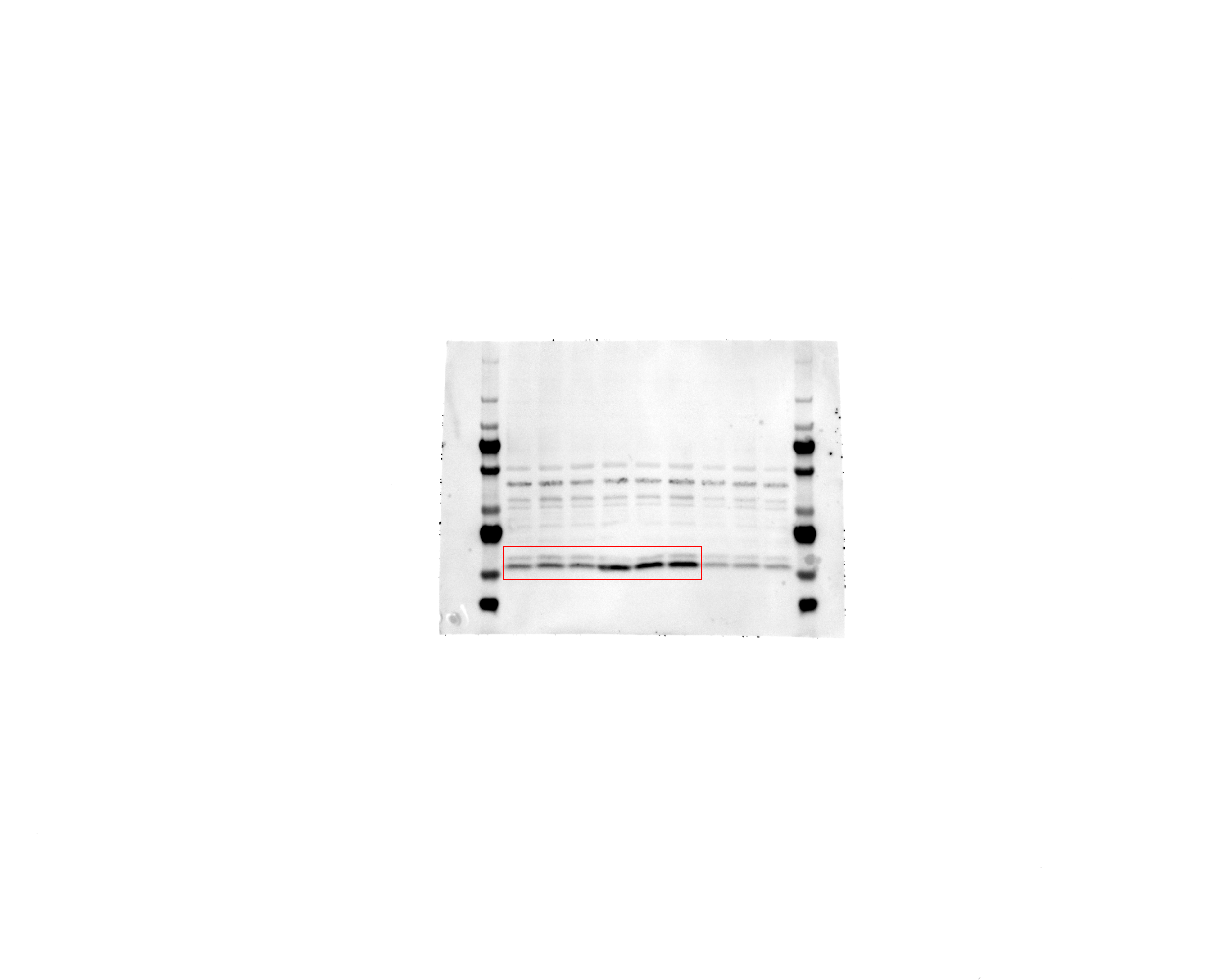

Supplement: Supplementary file 3 — Source Data for Expanded View [file EMBJ-42-e113168-s009.zip › Source data EV1-EV5/Figure EV1/EV1H/western_RBM3.tif]

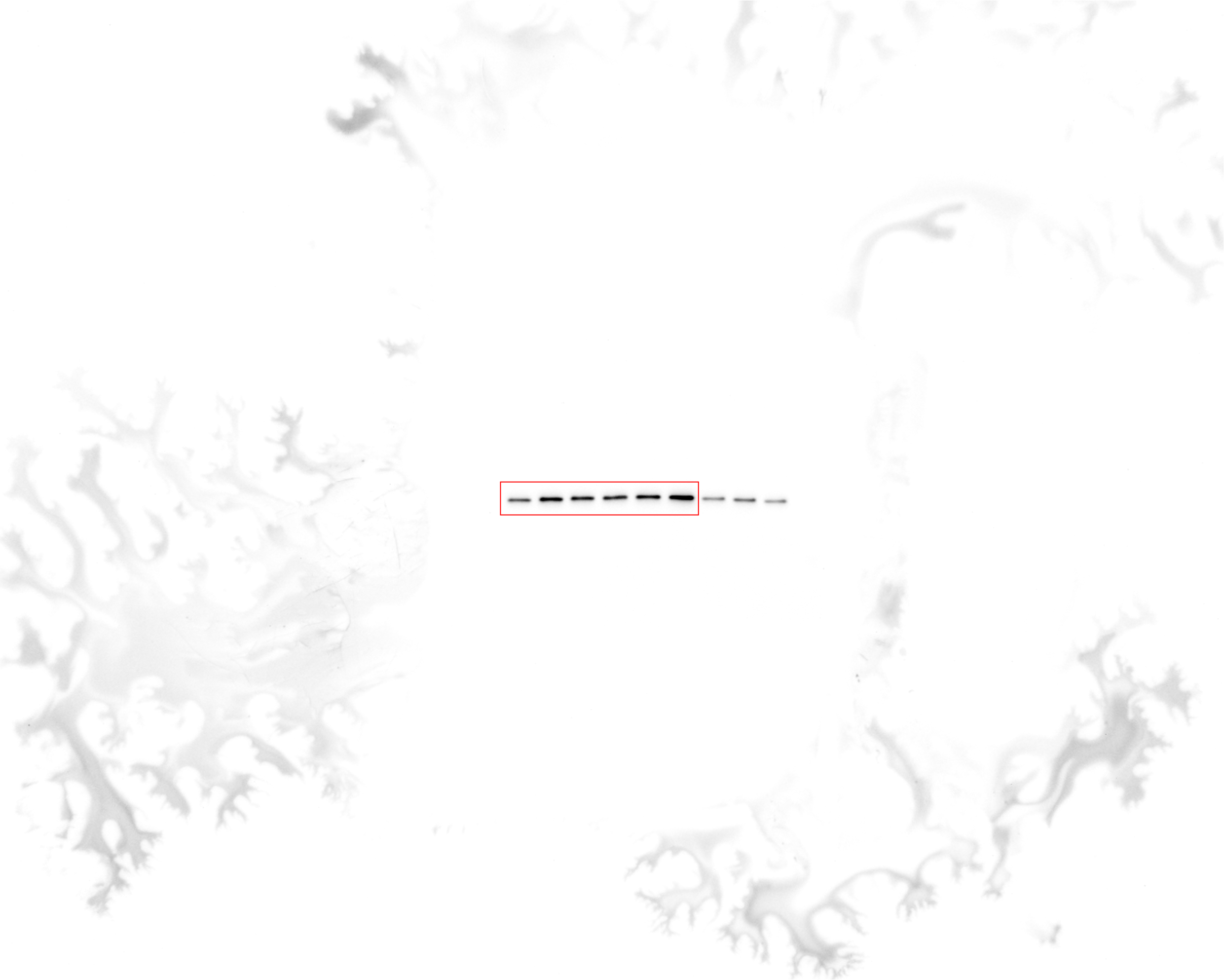

Supplement: Supplementary file 3 — Source Data for Expanded View [file EMBJ-42-e113168-s009.zip › Source data EV1-EV5/Figure EV1/EV1H/western_GAPDH.tif]

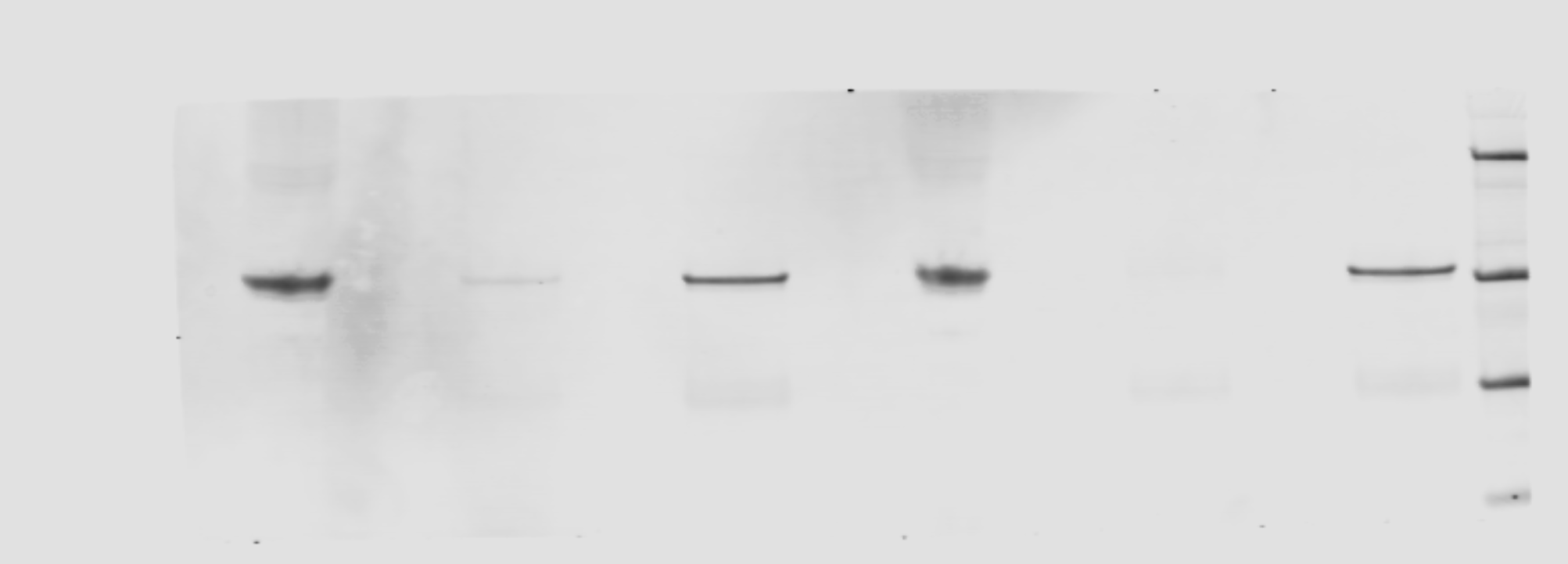

Supplement: Supplementary file 3 — Source Data for Expanded View [file EMBJ-42-e113168-s009.zip › Source data EV1-EV5/Figure EV5/EV5B/Set 2/HNRNPH1 WB Y12 IP ineurons set 2.tif]

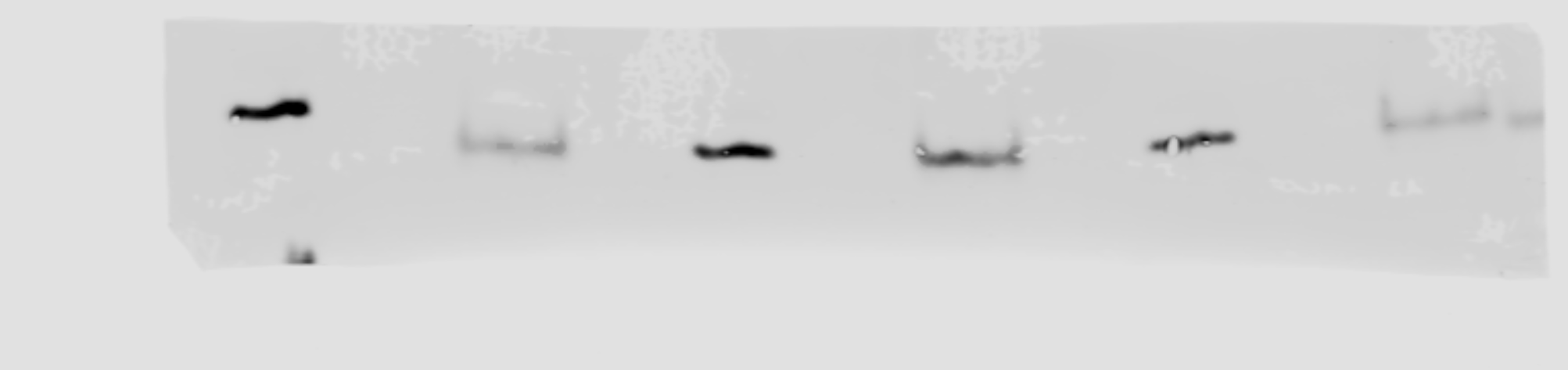

Supplement: Supplementary file 3 — Source Data for Expanded View [file EMBJ-42-e113168-s009.zip › Source data EV1-EV5/Figure EV5/EV5B/Set 2/SMD3 WB Y12 IP ineurons set 2.tif]

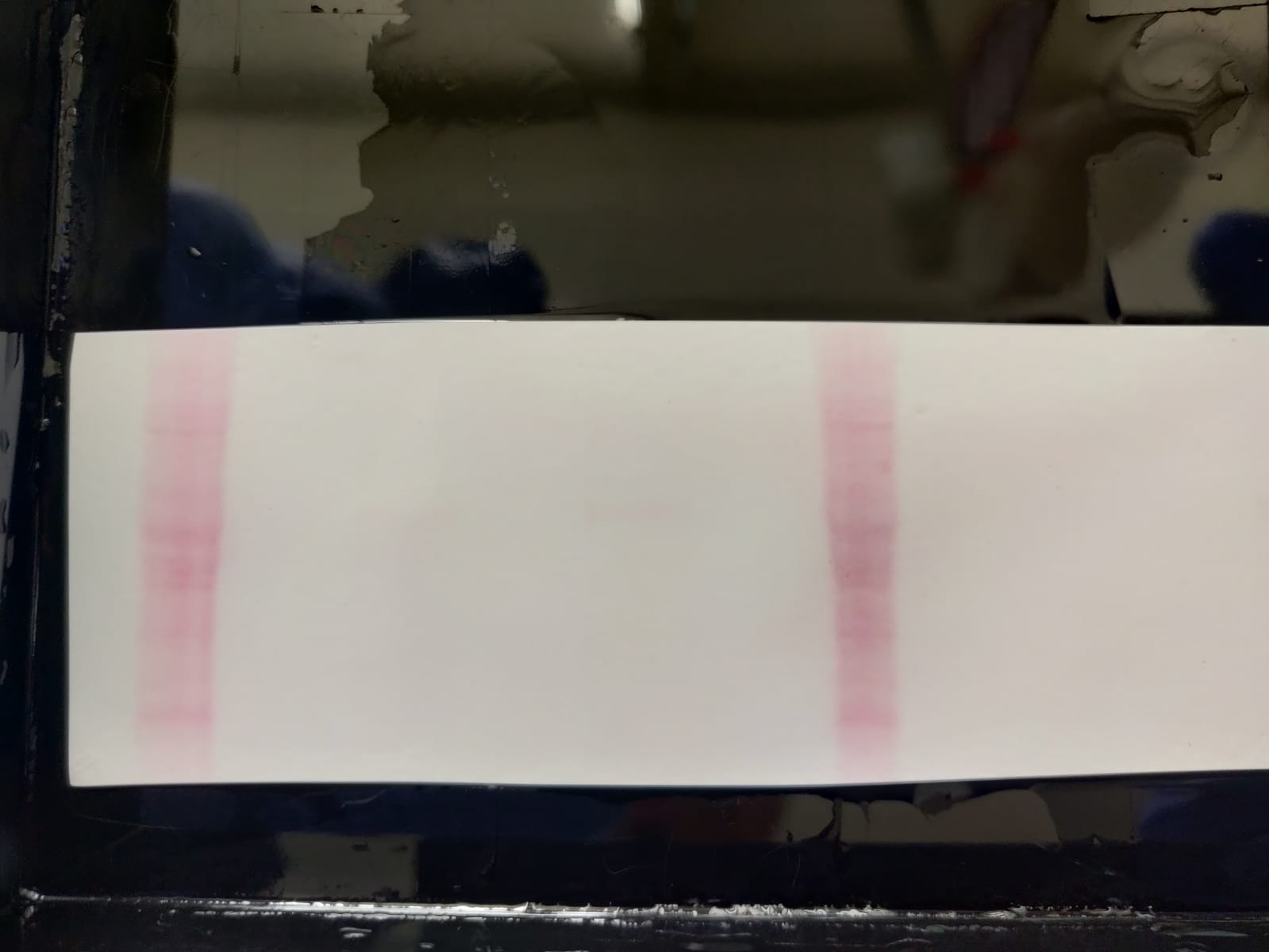

Supplement: Supplementary file 3 — Source Data for Expanded View [file EMBJ-42-e113168-s009.zip › Source data EV1-EV5/Figure EV5/EV5B/Set 2/Ponceau Y12 IP ineurons set 2.jpeg]

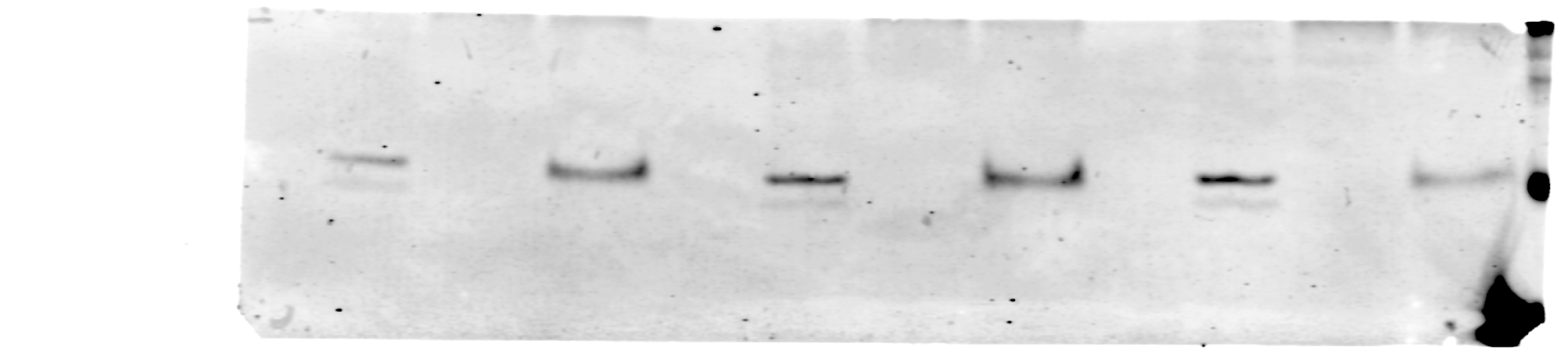

Supplement: Supplementary file 3 — Source Data for Expanded View [file EMBJ-42-e113168-s009.zip › Source data EV1-EV5/Figure EV5/EV5B/Set 1/SMD3 WB Y12 IP ineurons set 1.tif]

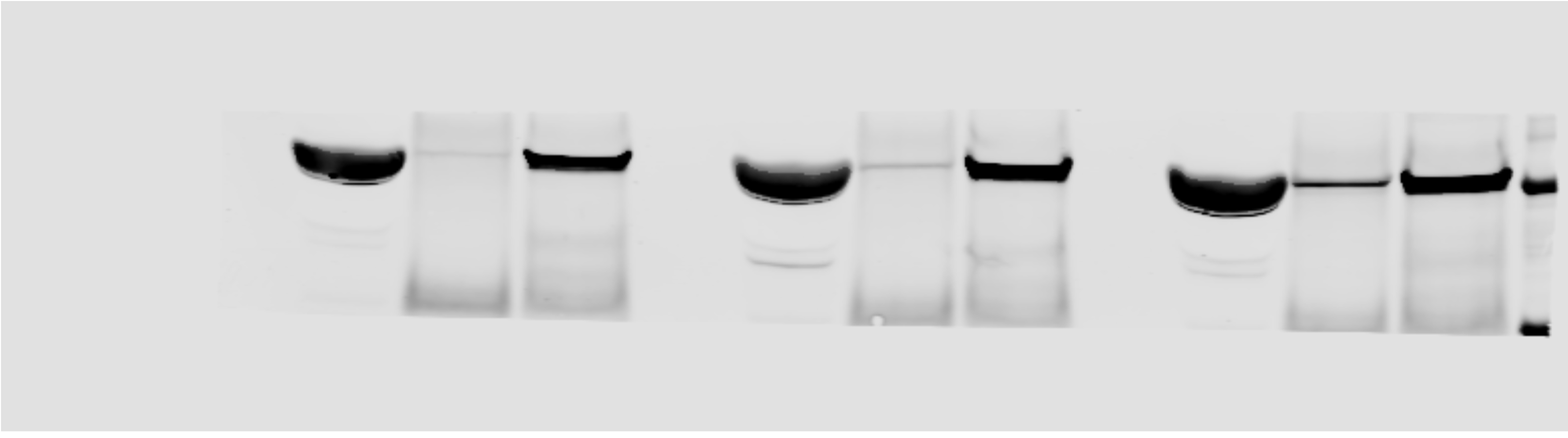

Supplement: Supplementary file 3 — Source Data for Expanded View [file EMBJ-42-e113168-s009.zip › Source data EV1-EV5/Figure EV5/EV5B/Set 1/HNRNPH1 WB Y12 IP ineurons set 1.png]

# Set 1

37°C

32°C

Input IgG IP SmB IP    Input IgG IP SmB IP

kDa

50

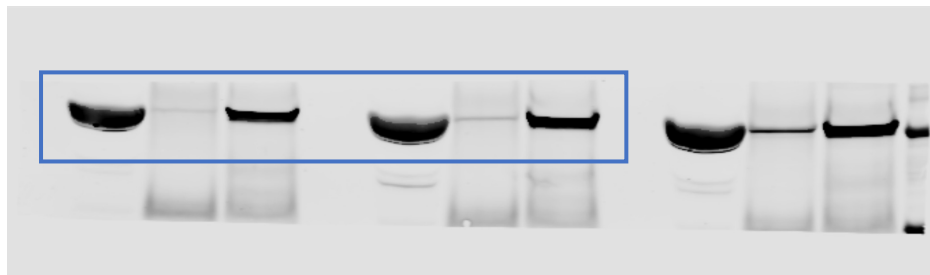

WB: HNRNPH

15

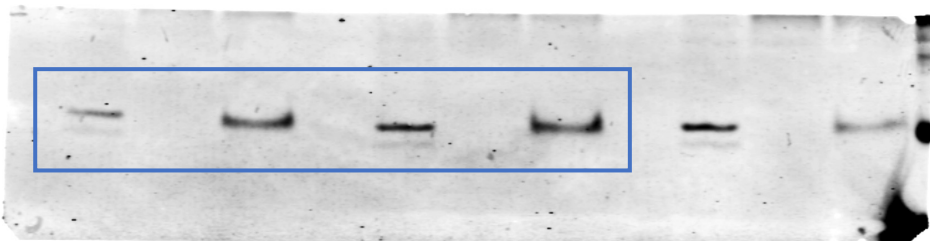

WB: SmD3

Supplement: Supplementary file 3 — Source Data for Expanded View [file EMBJ-42-e113168-s009.zip › Source data EV1-EV5/Figure EV5/EV5B/Set 1/Labelled WB GAPDH HNRNPH1 SMD3 SMB IP i-neurons Set 1 Used in the figure.pdf]

## Set 2

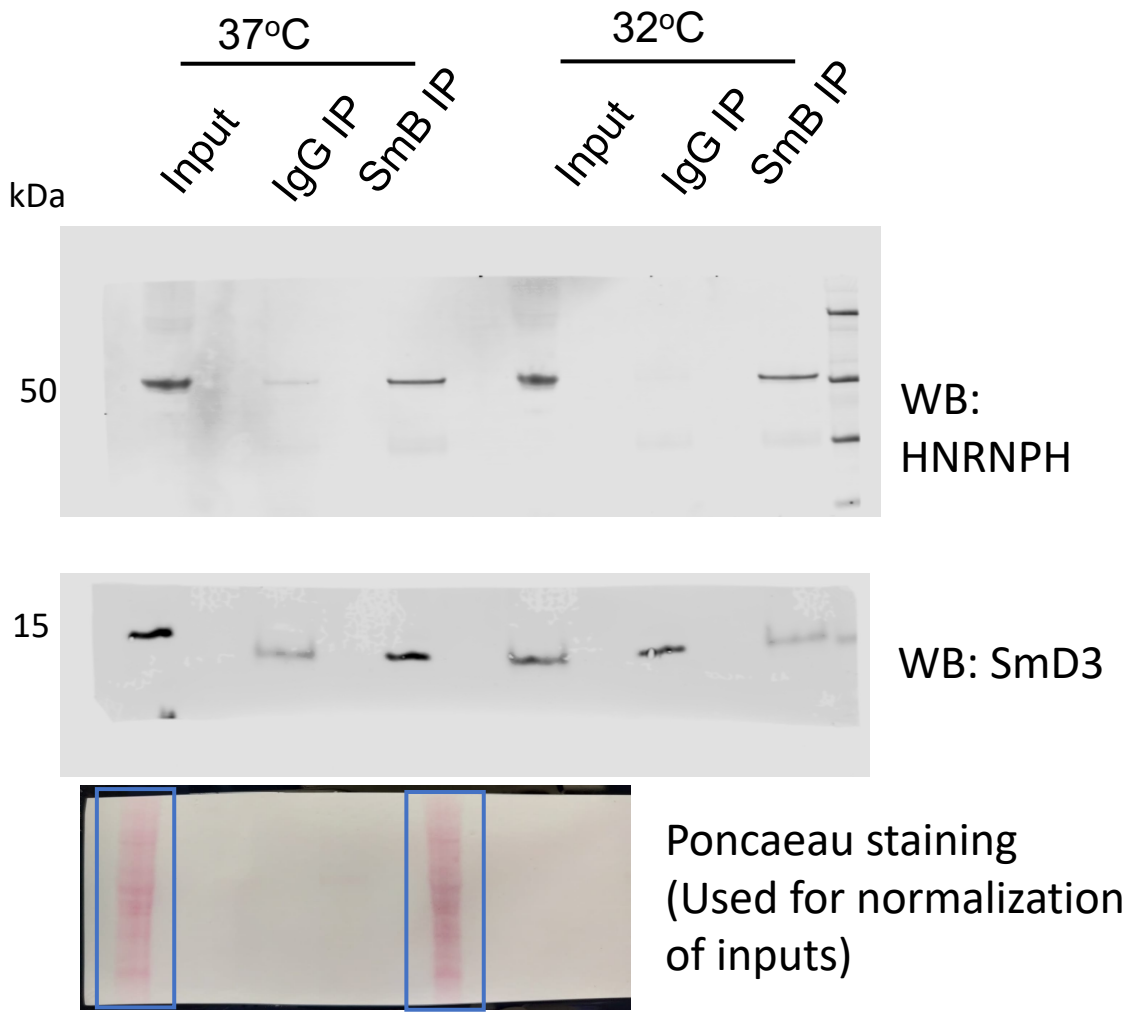

Supplement: Supplementary file 3 — Source Data for Expanded View [file EMBJ-42-e113168-s009.zip › Source data EV1-EV5/Figure EV5/EV5B/Set 1/Labelled WB GAPDH HNRNPH1 SMD3 SMB IP i-neurons Set 2.pdf]

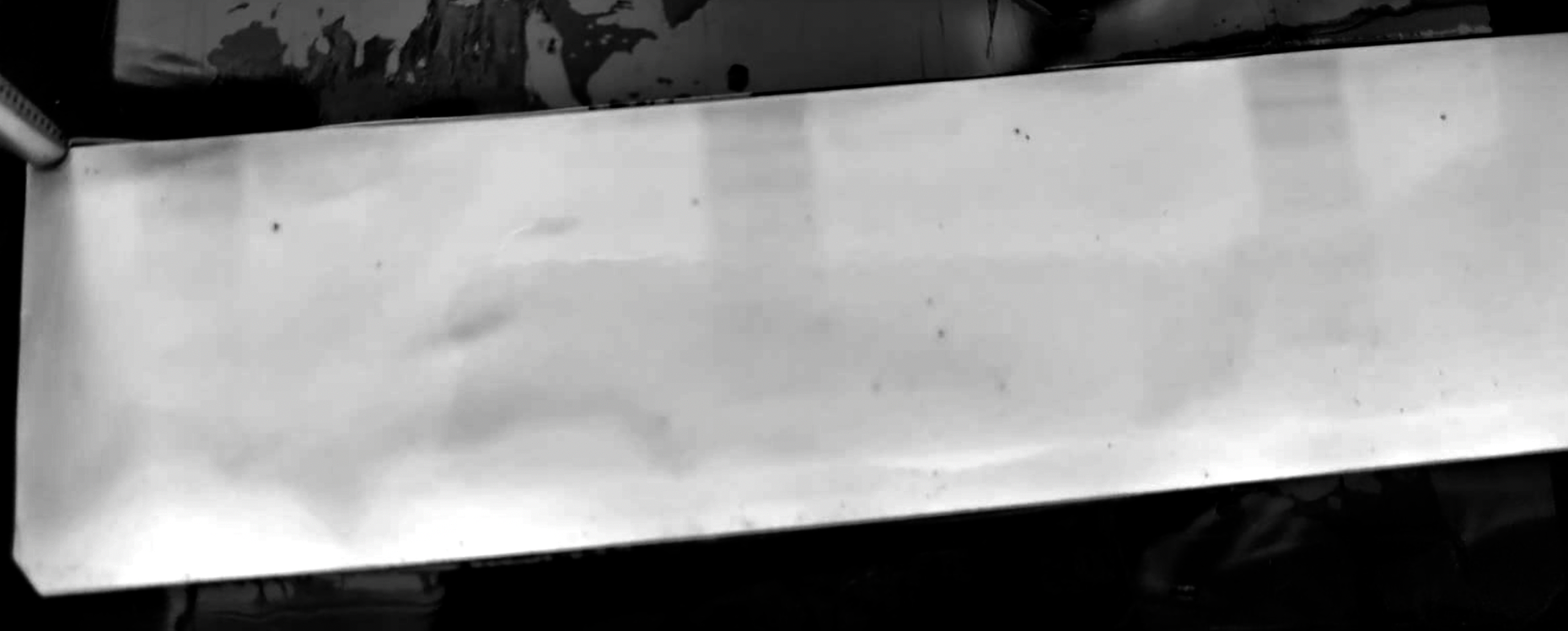

Supplement: Supplementary file 3 — Source Data for Expanded View [file EMBJ-42-e113168-s009.zip › Source data EV1-EV5/Figure EV5/EV5B/Set 1/Ponceau Y12 IP ineurons set 1.png]

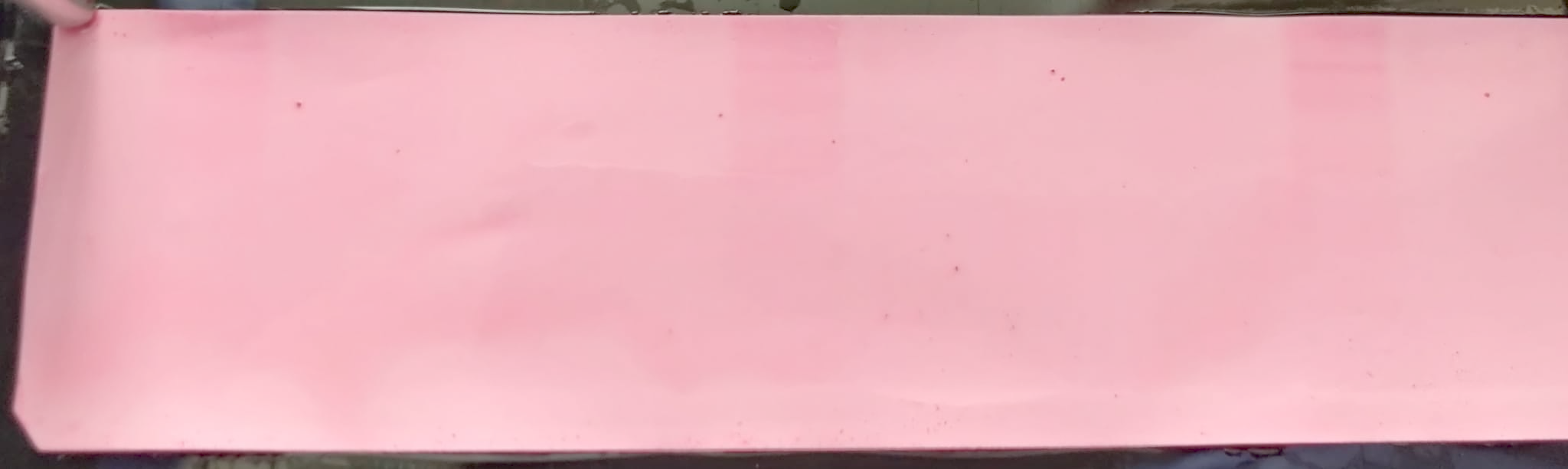

Supplement: Supplementary file 3 — Source Data for Expanded View [file EMBJ-42-e113168-s009.zip › Source data EV1-EV5/Figure EV5/EV5B/Set 1/Ponceau Y12 IP ineurons set 1 b.tiff]

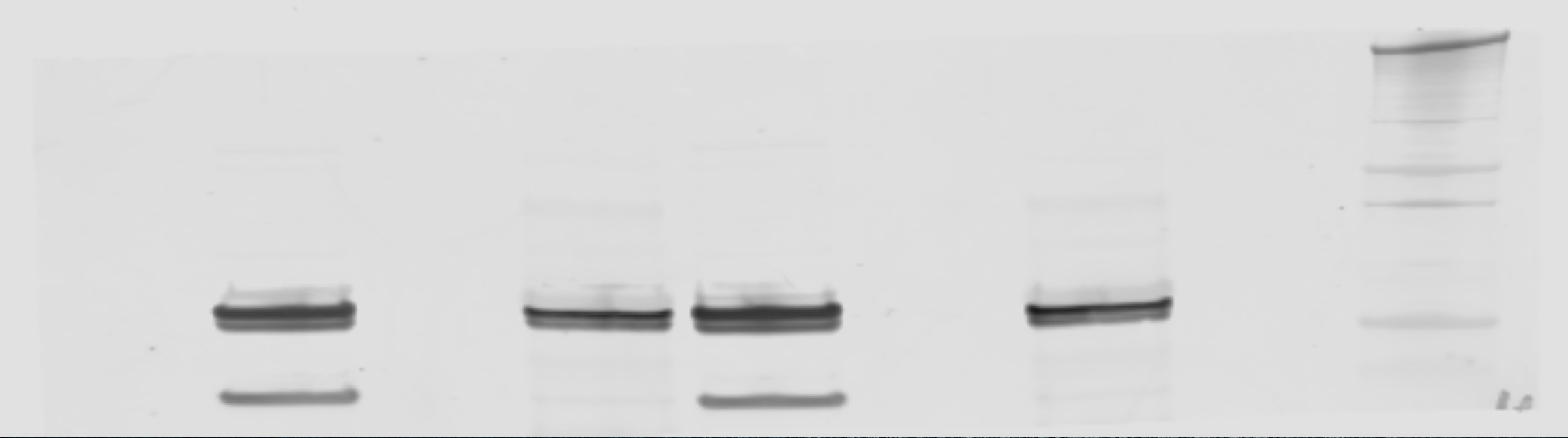

Supplement: Supplementary file 3 — Source Data for Expanded View [file EMBJ-42-e113168-s009.zip › Source data EV1-EV5/Figure EV5/EV5A/Set 2/GAPDH WB Y12 IP Set 2 HeLa.png]

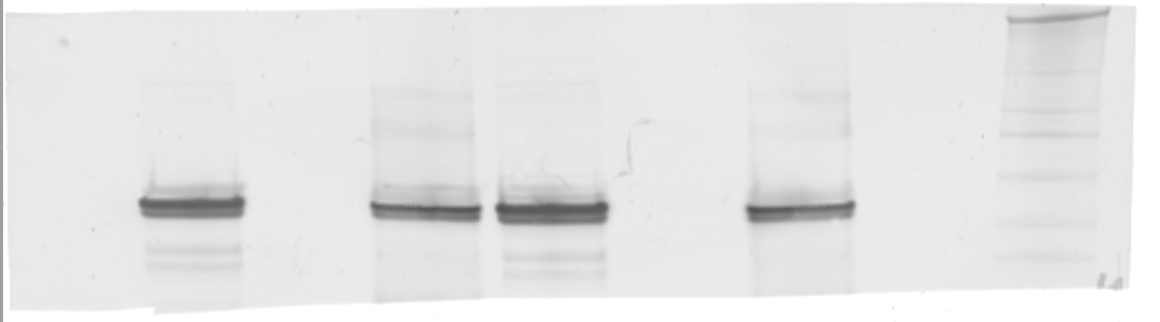

Supplement: Supplementary file 3 — Source Data for Expanded View [file EMBJ-42-e113168-s009.zip › Source data EV1-EV5/Figure EV5/EV5A/Set 2/HNRNPH1 WB Y12 IP Hela set 2.png]

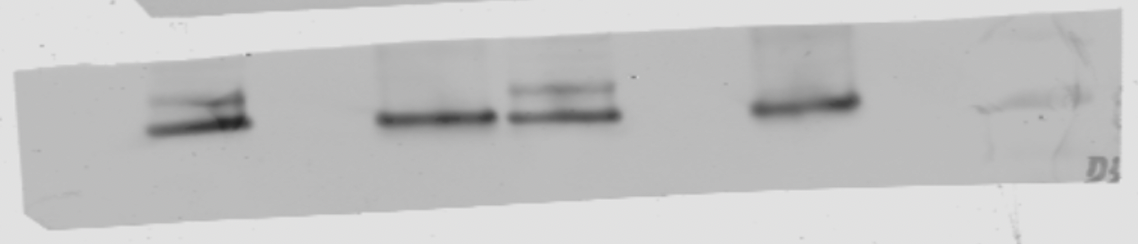

Supplement: Supplementary file 3 — Source Data for Expanded View [file EMBJ-42-e113168-s009.zip › Source data EV1-EV5/Figure EV5/EV5A/Set 2/SMD3 WB Y12 IP Hela set 2.png]

## Set 2

37°C                      32°C

Input   IgG IP   SmB IP   Input   IgG IP   SmB IP

kDa

50  
40

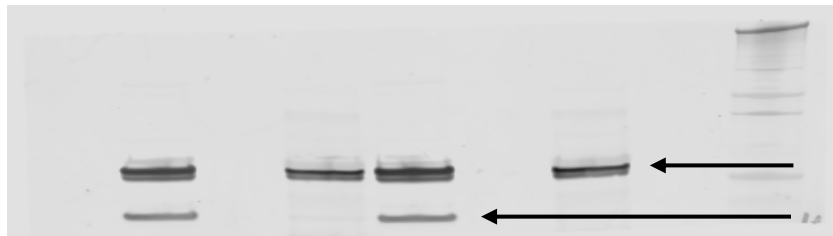

WB: HNRNPH  
WB: GAPDH

15

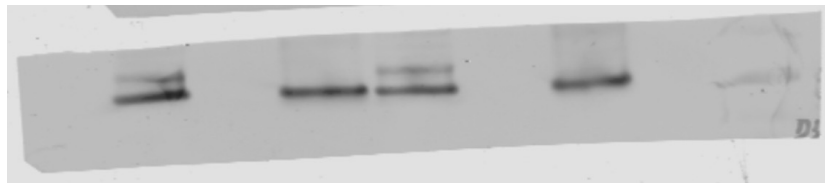

WB: SmD3

Supplement: Supplementary file 3 — Source Data for Expanded View [file EMBJ-42-e113168-s009.zip › Source data EV1-EV5/Figure EV5/EV5A/Set 2/Labelled WB GAPDH HNRNPH1 SMD3 SMB IP HeLa Set 2.pdf]

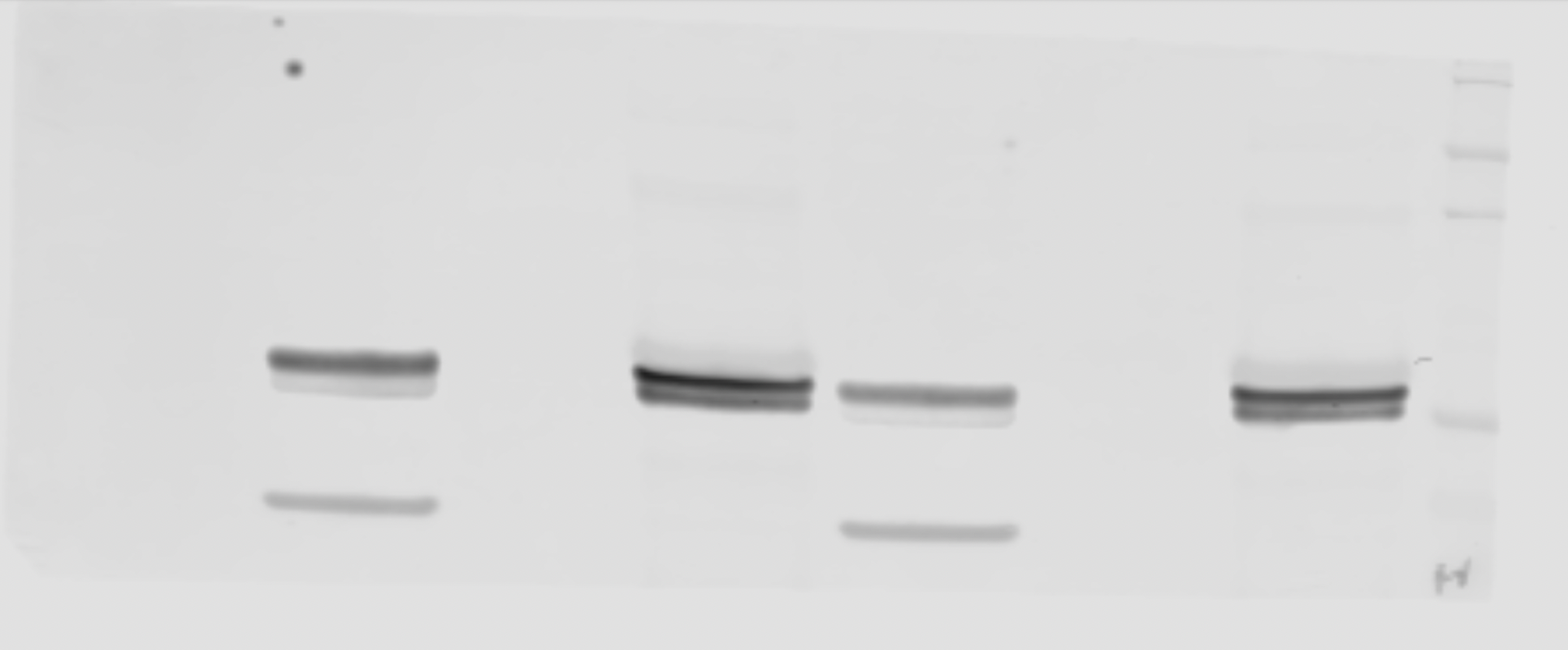

Supplement: Supplementary file 3 — Source Data for Expanded View [file EMBJ-42-e113168-s009.zip › Source data EV1-EV5/Figure EV5/EV5A/Set 3/GAPDH WB Y12 IP Set 3 HeLa.png]

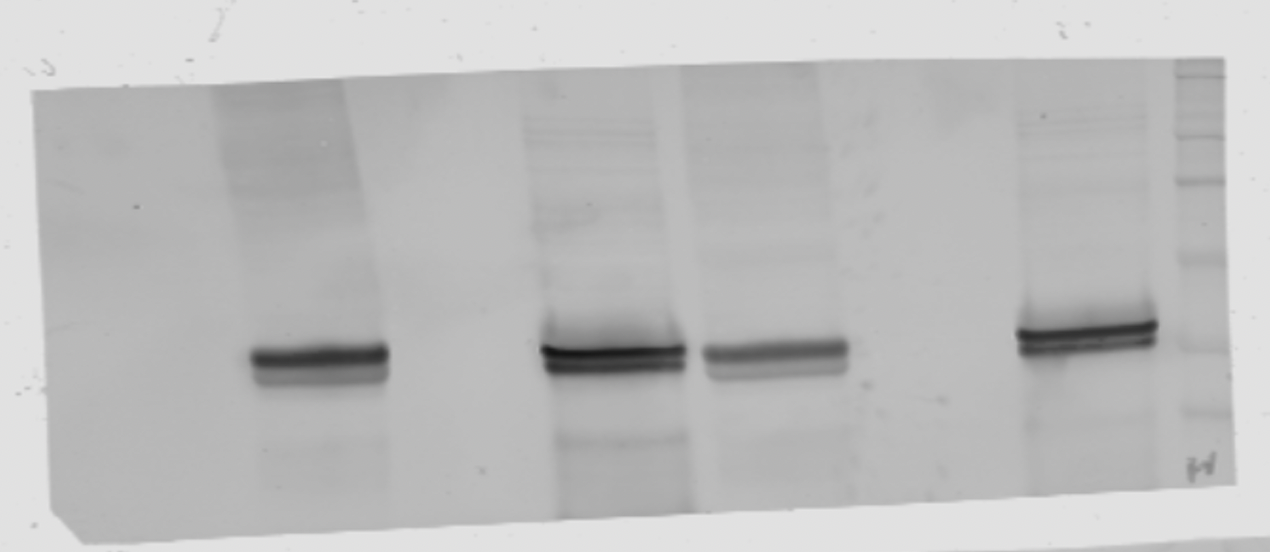

Supplement: Supplementary file 3 — Source Data for Expanded View [file EMBJ-42-e113168-s009.zip › Source data EV1-EV5/Figure EV5/EV5A/Set 3/HNRNPH WB Y12 IP Hela set 3.png]

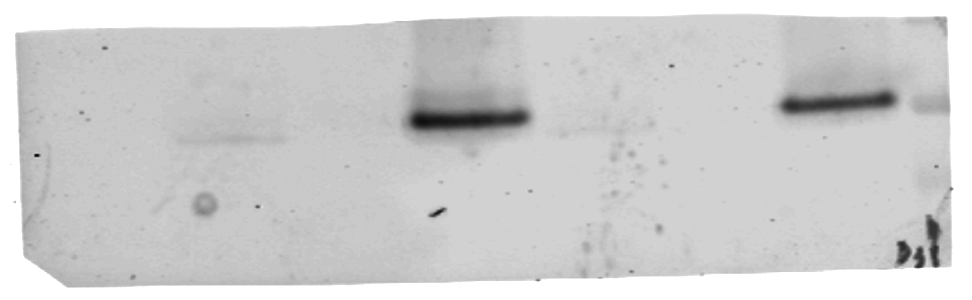

Supplement: Supplementary file 3 — Source Data for Expanded View [file EMBJ-42-e113168-s009.zip › Source data EV1-EV5/Figure EV5/EV5A/Set 3/SMD3 WB Y12 IP Hela set 3.png]

# Set 3

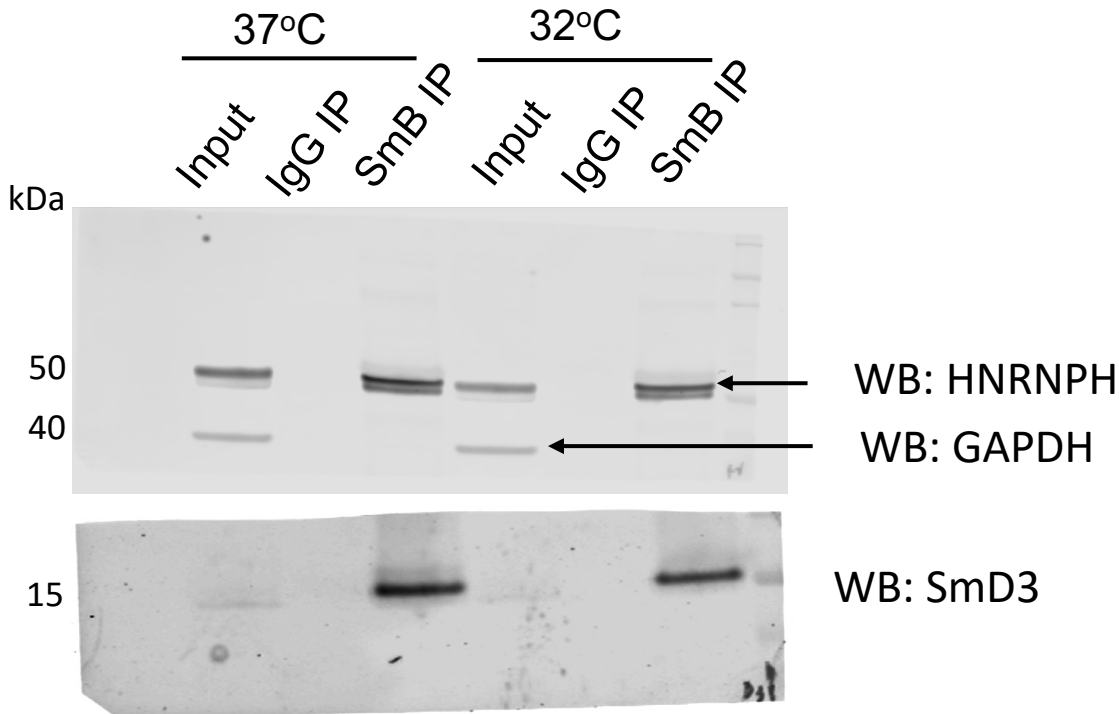

Supplement: Supplementary file 3 — Source Data for Expanded View [file EMBJ-42-e113168-s009.zip › Source data EV1-EV5/Figure EV5/EV5A/Set 3/Labelled WB GAPDH HNRNPH1 SMD3 SMB IP HeLa Set 3.pdf]

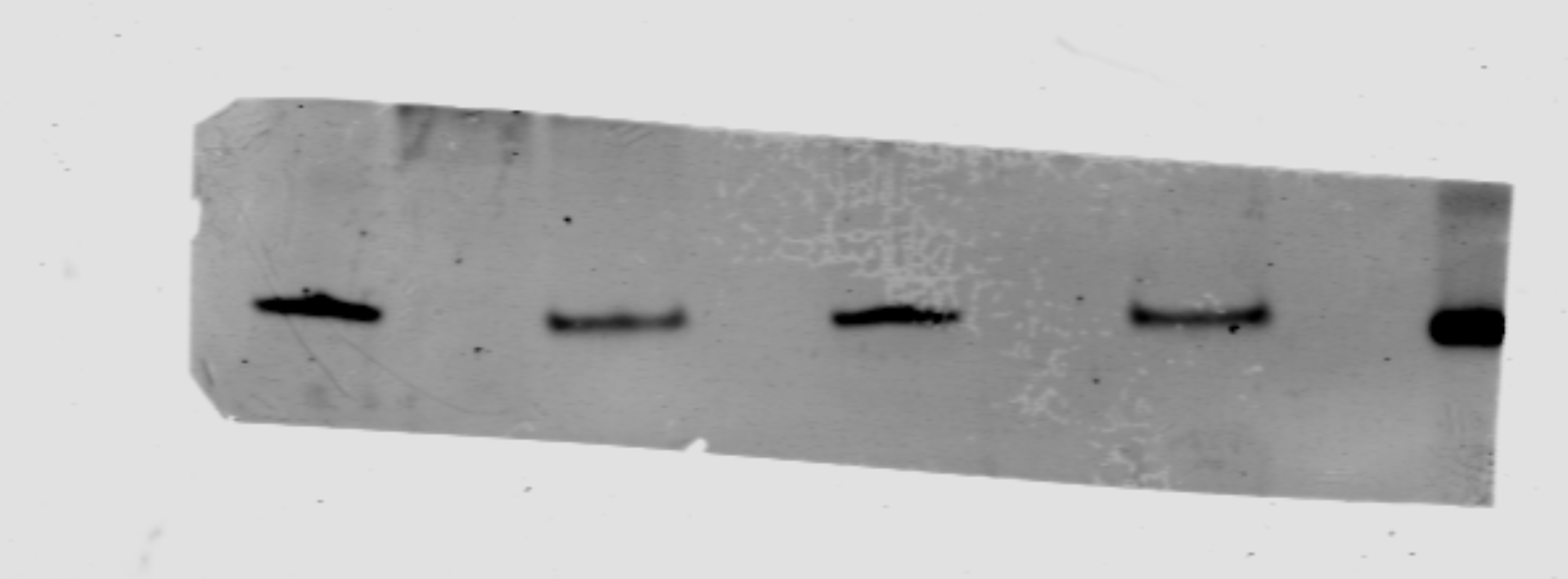

Supplement: Supplementary file 3 — Source Data for Expanded View [file EMBJ-42-e113168-s009.zip › Source data EV1-EV5/Figure EV5/EV5A/Set 1/SMD3 WB Y12IP set 1 HeLa.png]

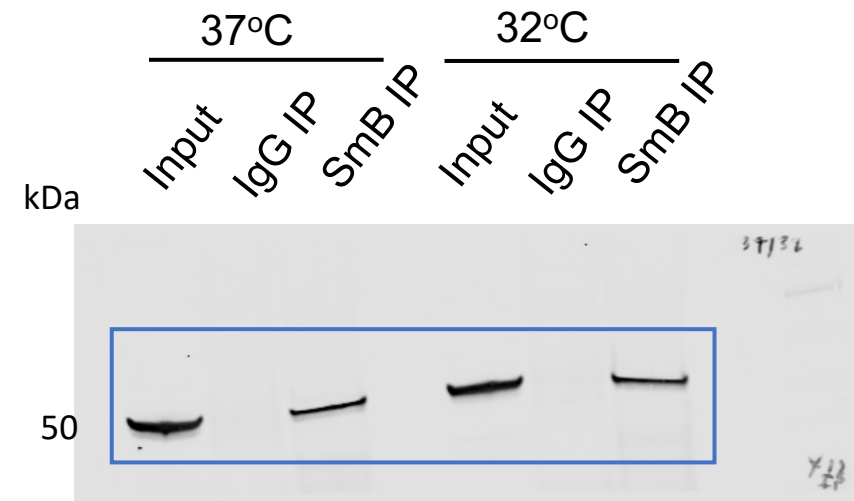

WB: HNRNPH

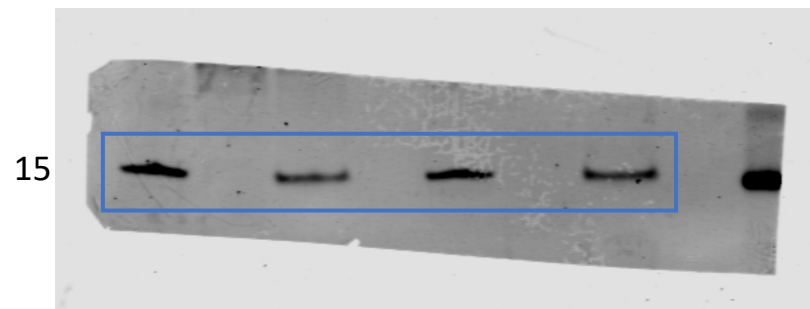

WB: SmD3

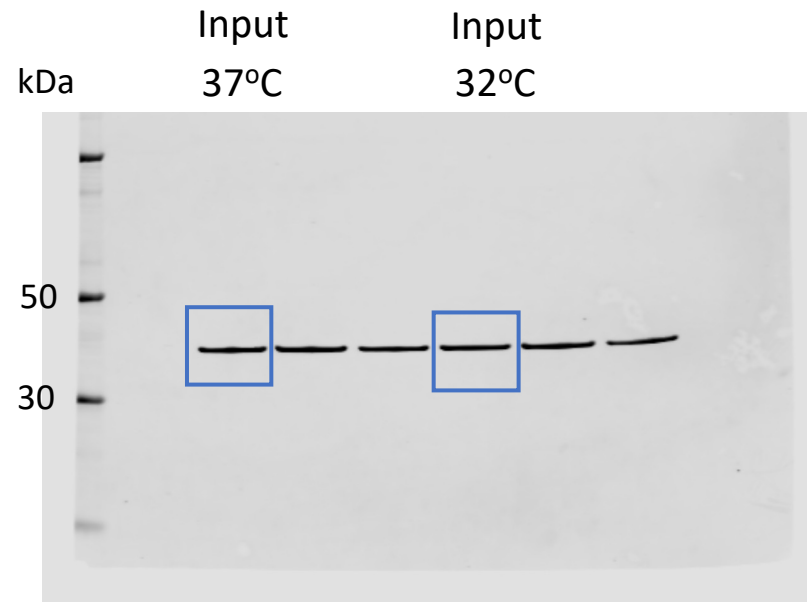

WB: GAPDH  
(Used for normalization  
of inputs)

Supplement: Supplementary file 3 — Source Data for Expanded View [file EMBJ-42-e113168-s009.zip › Source data EV1-EV5/Figure EV5/EV5A/Set 1/Labelled WB GAPDH HNRNPH1 SMD3 SMB IP HeLa Set 1 Used in the figure.pdf]

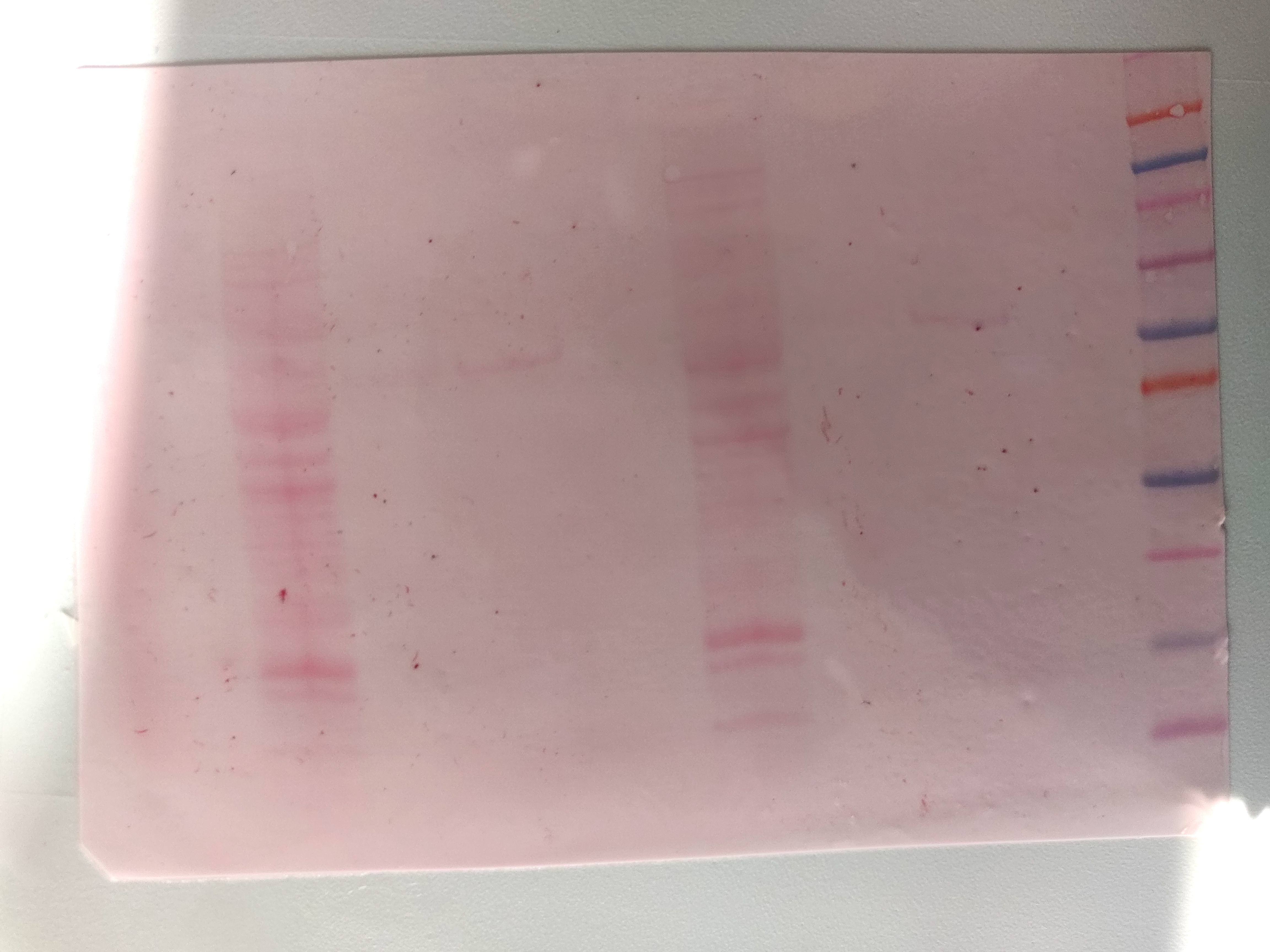

Supplement: Supplementary file 3 — Source Data for Expanded View [file EMBJ-42-e113168-s009.zip › Source data EV1-EV5/Figure EV5/EV5A/Set 1/Ponceau smB Ip IMG_20210929_154054.png]

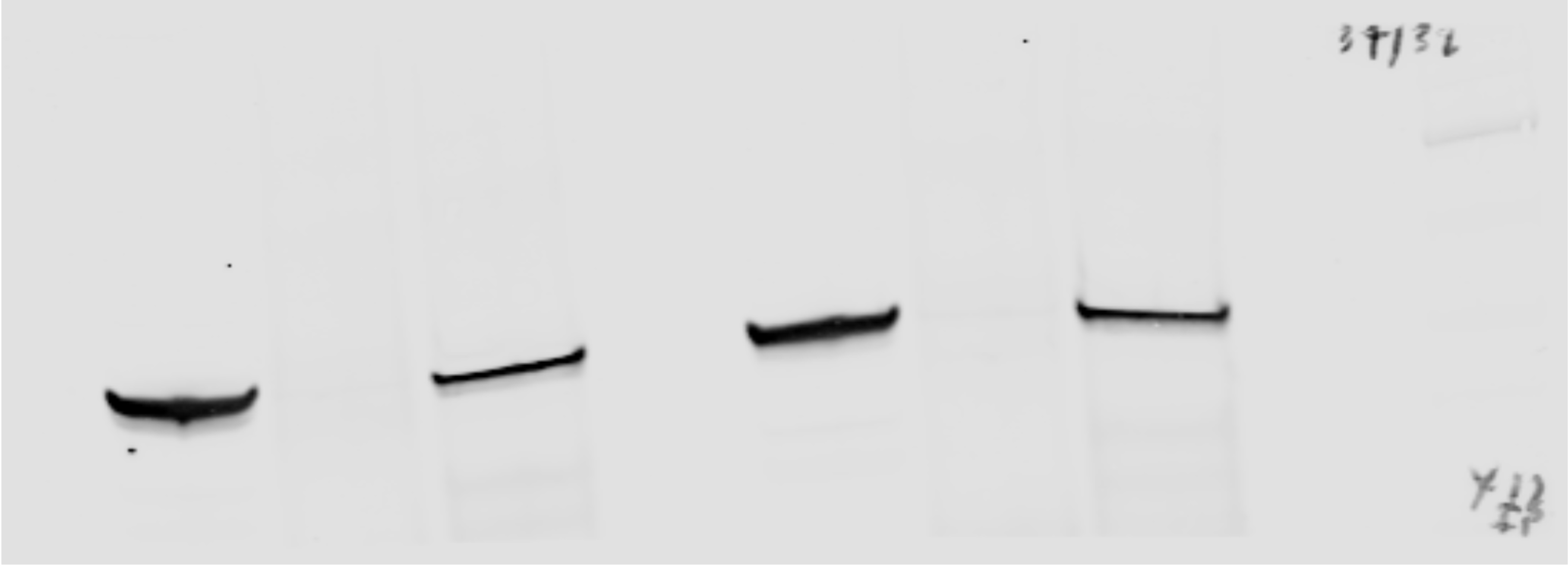

Supplement: Supplementary file 3 — Source Data for Expanded View [file EMBJ-42-e113168-s009.zip › Source data EV1-EV5/Figure EV5/EV5A/Set 1/HNRNPH WB Y12IP set 1 HeLa.png]

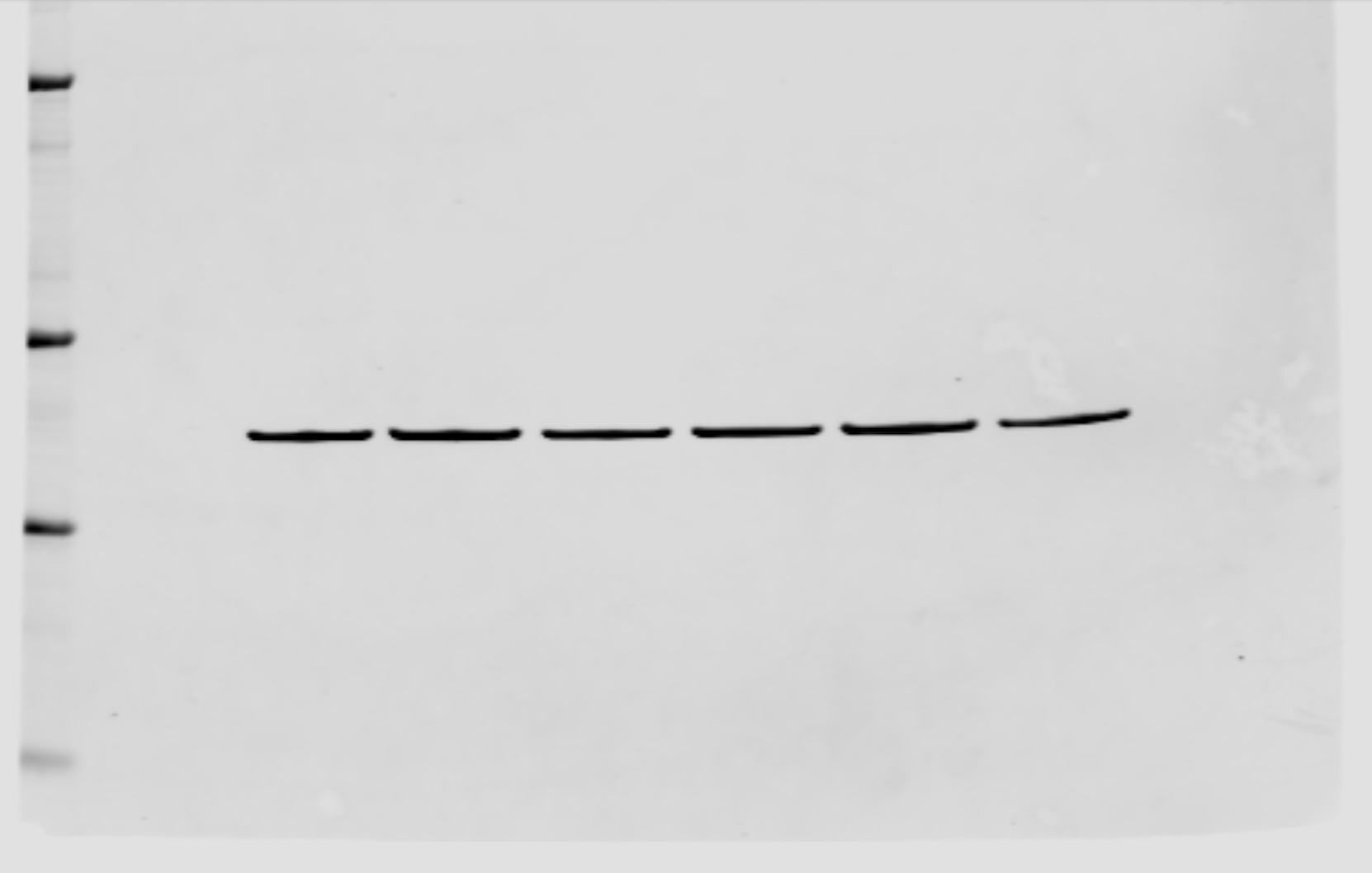

Supplement: Supplementary file 3 — Source Data for Expanded View [file EMBJ-42-e113168-s009.zip › Source data EV1-EV5/Figure EV5/EV5A/Set 1/GAPDH WB Y12 IP Set 1 HeLa.png]

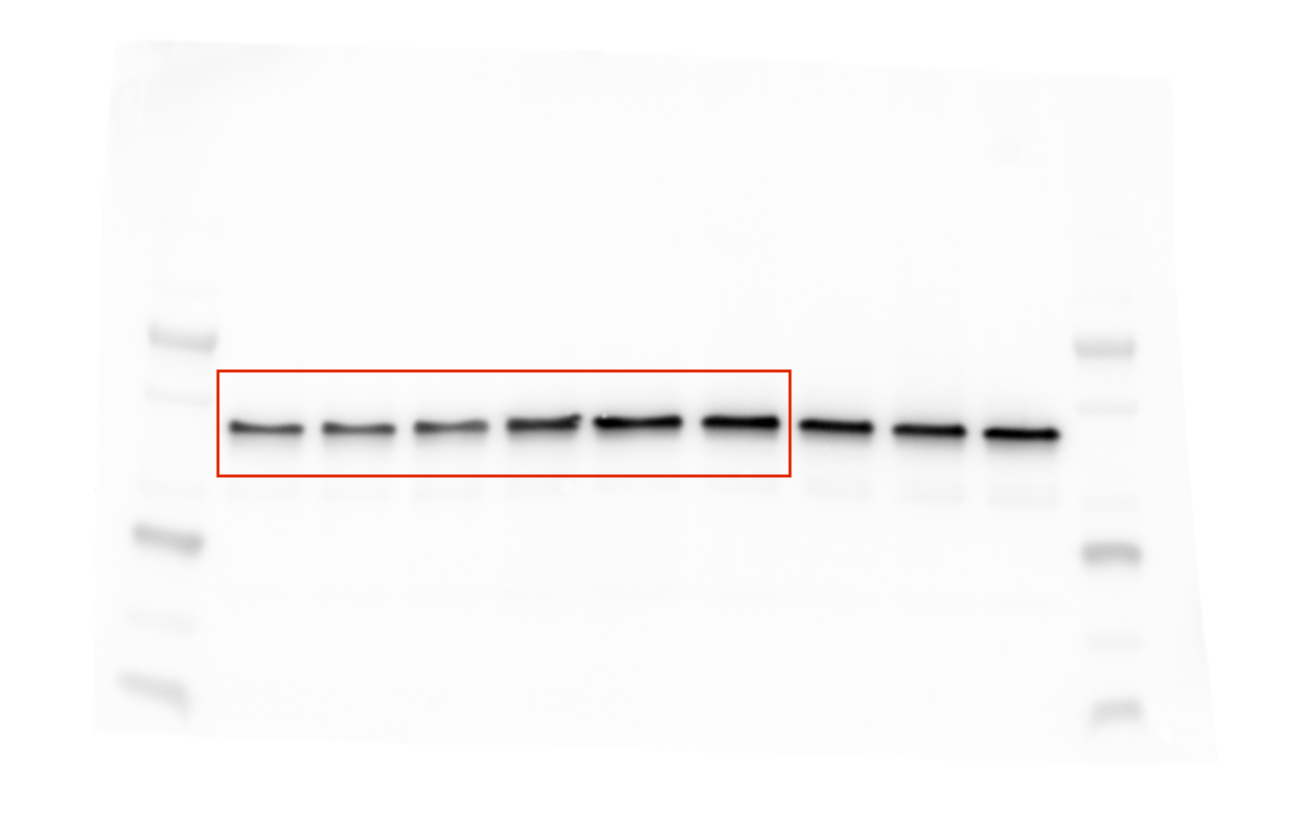

Supplement: Supplementary file 5 — Source Data for Figure 1 [file EMBJ-42-e113168-s008.zip › Figure 1/1B/western_RBM3.tif]

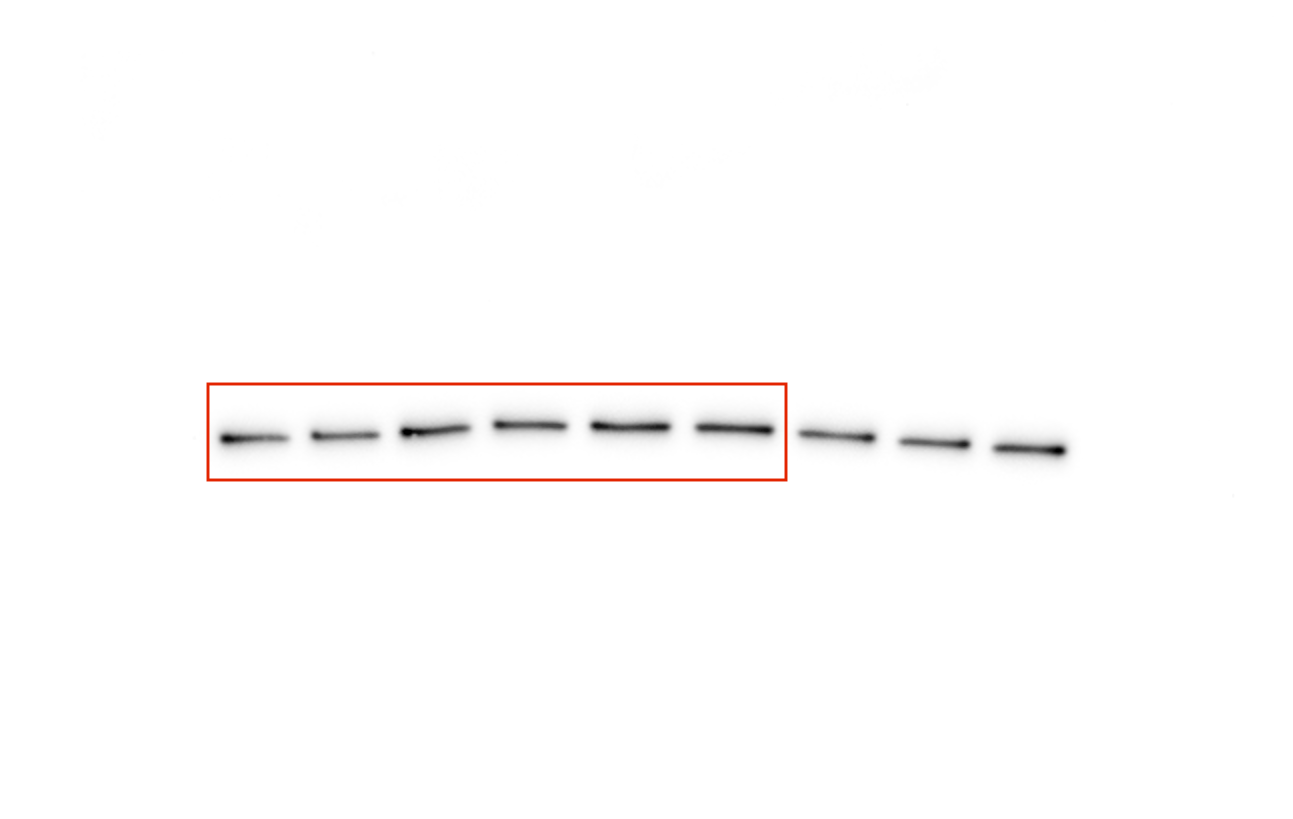

Supplement: Supplementary file 5 — Source Data for Figure 1 [file EMBJ-42-e113168-s008.zip › Figure 1/1B/western_GAPDH.tif]

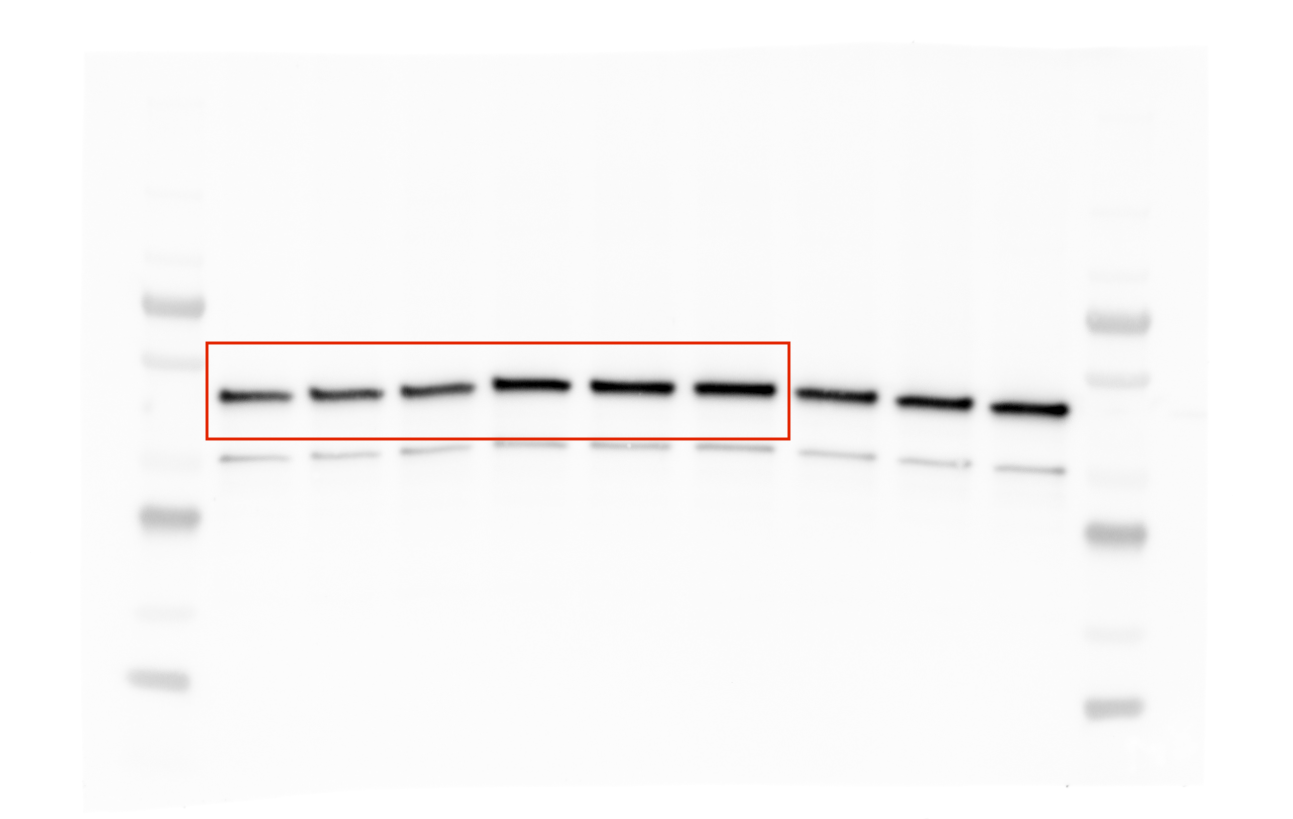

Supplement: Supplementary file 5 — Source Data for Figure 1 [file EMBJ-42-e113168-s008.zip › Figure 1/1B/western_GFP.tif]

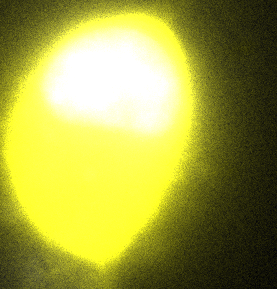

Supplement: Supplementary file 5 — Source Data for Figure 1 [file EMBJ-42-e113168-s008.zip › Figure 1/1A/Images used in the figure/32C/0823_Control3_Frame4_Area1.tif]

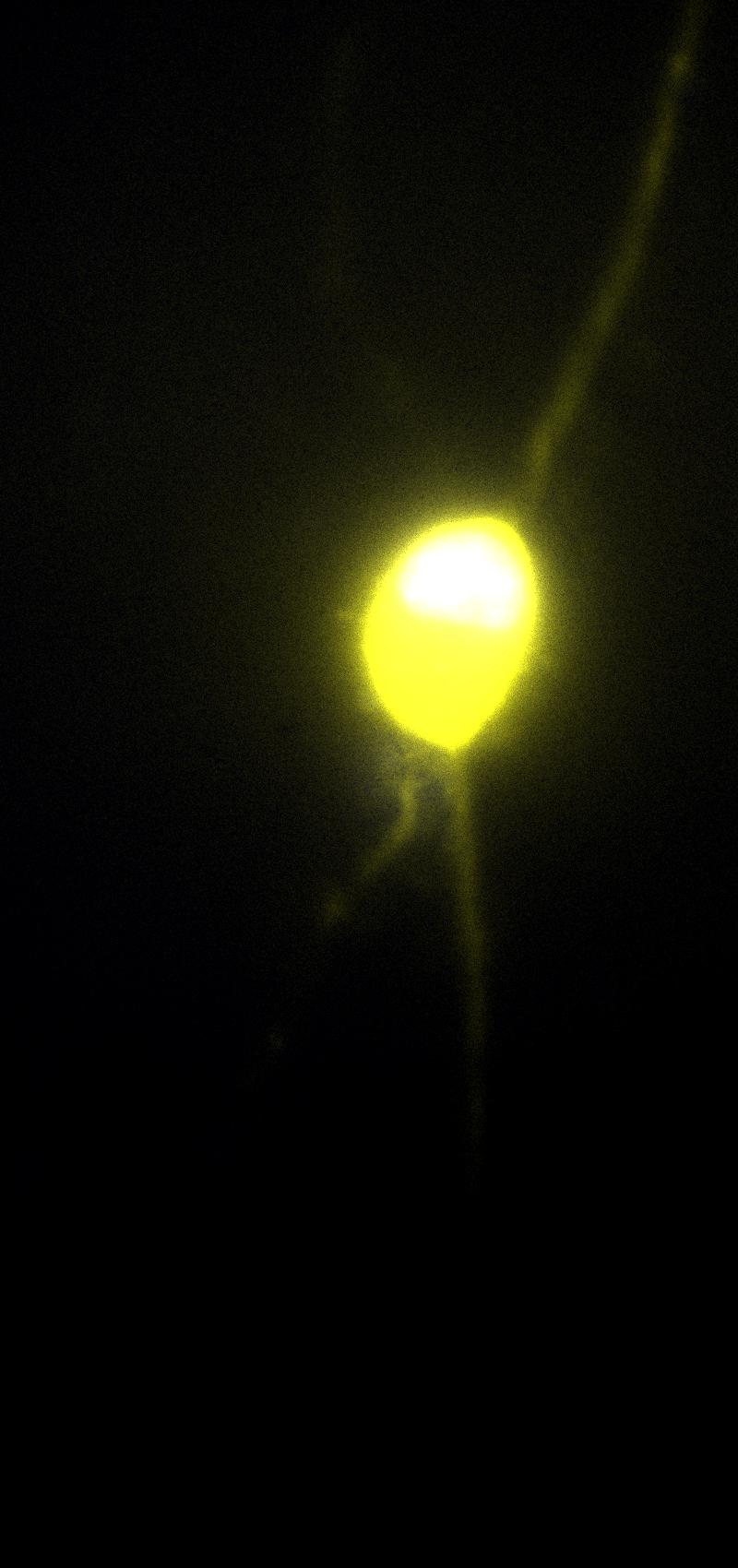

Supplement: Supplementary file 5 — Source Data for Figure 1 [file EMBJ-42-e113168-s008.zip › Figure 1/1A/Images used in the figure/32C/Frame4.tif]

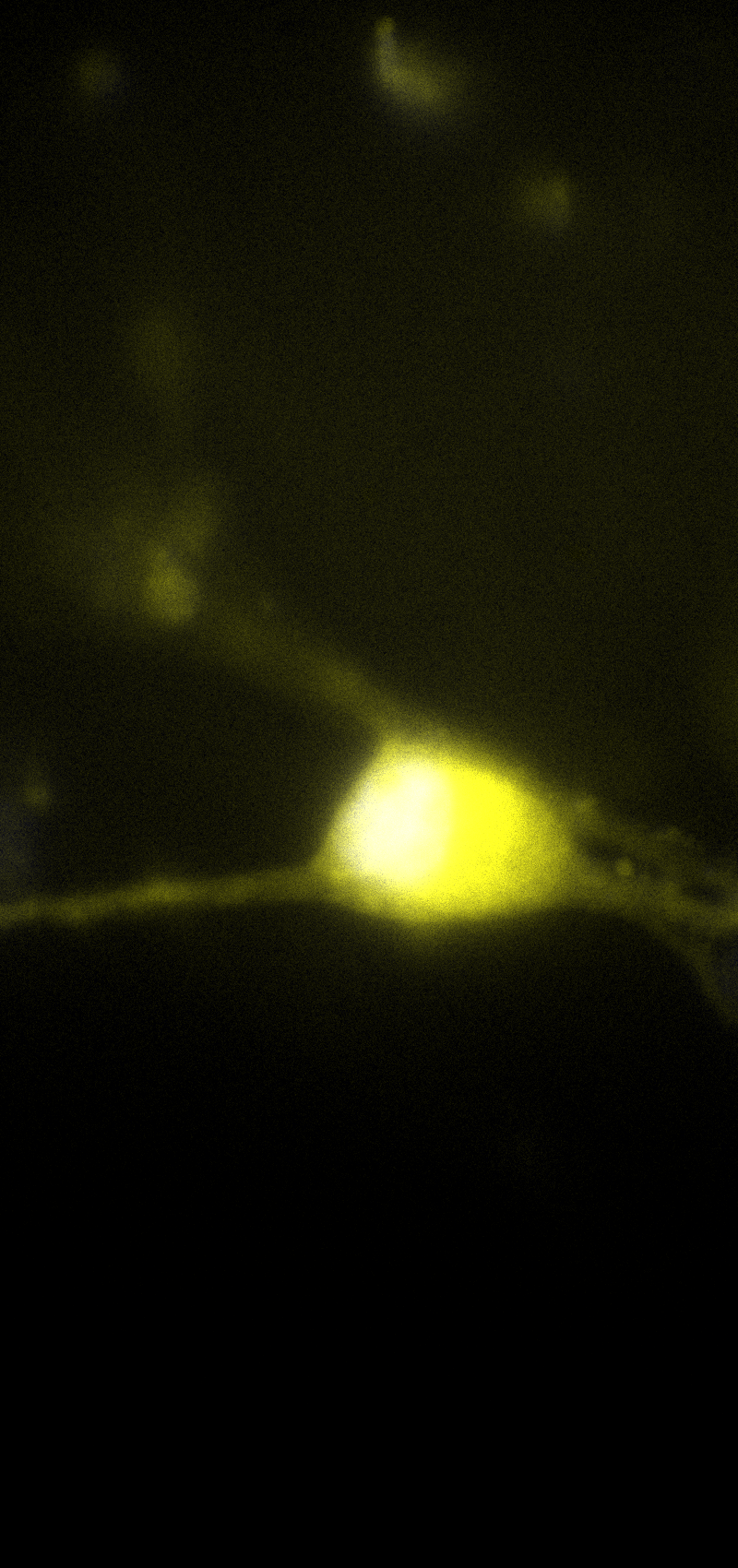

Supplement: Supplementary file 5 — Source Data for Figure 1 [file EMBJ-42-e113168-s008.zip › Figure 1/1A/Images used in the figure/37C/Frame8.tif]

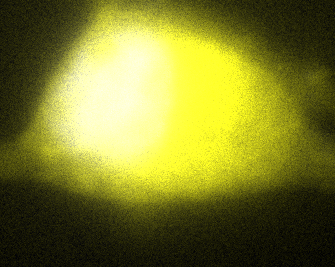

Supplement: Supplementary file 5 — Source Data for Figure 1 [file EMBJ-42-e113168-s008.zip › Figure 1/1A/Images used in the figure/37C/37C_S1r2__038.tif]

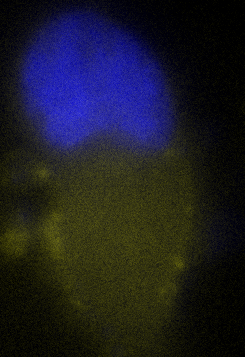

Supplement: Supplementary file 5 — Source Data for Figure 1 [file EMBJ-42-e113168-s008.zip › Figure 1/1A/Images/Replicate 2/32C/32C_S2r1__057.tif]

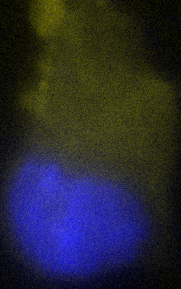

Supplement: Supplementary file 5 — Source Data for Figure 1 [file EMBJ-42-e113168-s008.zip › Figure 1/1A/Images/Replicate 2/32C/32C_S2r1__056.tif]

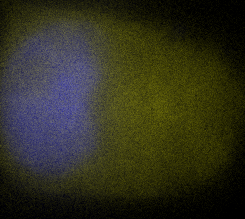

Supplement: Supplementary file 5 — Source Data for Figure 1 [file EMBJ-42-e113168-s008.zip › Figure 1/1A/Images/Replicate 2/32C/32C_S2r1__054.tif]

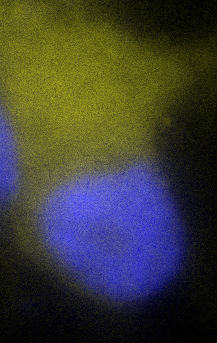

Supplement: Supplementary file 5 — Source Data for Figure 1 [file EMBJ-42-e113168-s008.zip › Figure 1/1A/Images/Replicate 2/32C/32C_S2r1__068.tif]

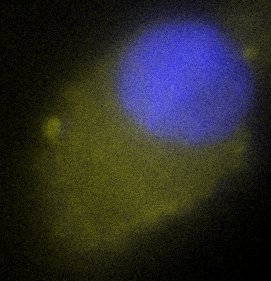

Supplement: Supplementary file 5 — Source Data for Figure 1 [file EMBJ-42-e113168-s008.zip › Figure 1/1A/Images/Replicate 2/32C/32C_S2r1__069.tif]

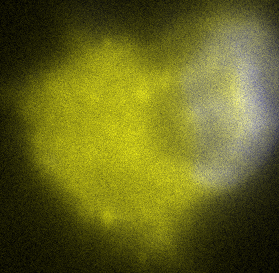

Supplement: Supplementary file 5 — Source Data for Figure 1 [file EMBJ-42-e113168-s008.zip › Figure 1/1A/Images/Replicate 2/32C/32C_S2r1__055.tif]

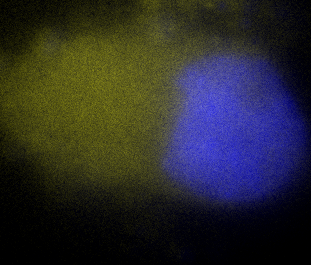

Supplement: Supplementary file 5 — Source Data for Figure 1 [file EMBJ-42-e113168-s008.zip › Figure 1/1A/Images/Replicate 2/32C/32C_S2r1__053.tif]

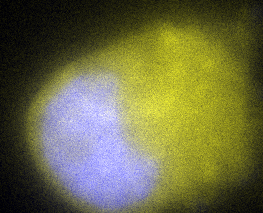

Supplement: Supplementary file 5 — Source Data for Figure 1 [file EMBJ-42-e113168-s008.zip › Figure 1/1A/Images/Replicate 2/32C/32C_S2r2__089.tif]

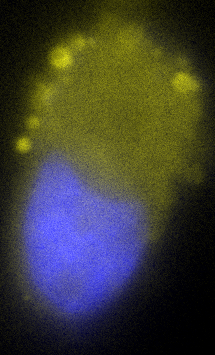

Supplement: Supplementary file 5 — Source Data for Figure 1 [file EMBJ-42-e113168-s008.zip › Figure 1/1A/Images/Replicate 2/32C/32C_S2r2__076.tif]

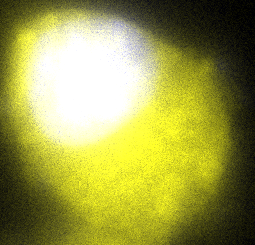

Supplement: Supplementary file 5 — Source Data for Figure 1 [file EMBJ-42-e113168-s008.zip › Figure 1/1A/Images/Replicate 2/32C/32C_S2r2__077.tif]

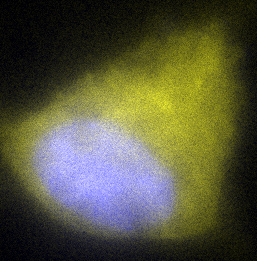

Supplement: Supplementary file 5 — Source Data for Figure 1 [file EMBJ-42-e113168-s008.zip › Figure 1/1A/Images/Replicate 2/32C/32C_S2r2__088.tif]

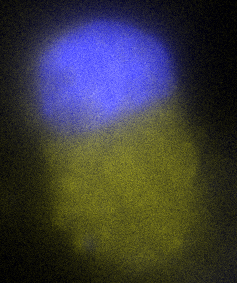

Supplement: Supplementary file 5 — Source Data for Figure 1 [file EMBJ-42-e113168-s008.zip › Figure 1/1A/Images/Replicate 2/32C/32C_S2r2__085.tif]

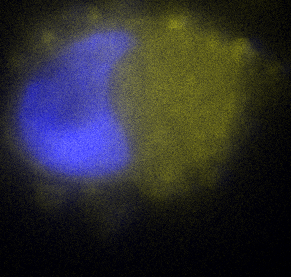

Supplement: Supplementary file 5 — Source Data for Figure 1 [file EMBJ-42-e113168-s008.zip › Figure 1/1A/Images/Replicate 2/32C/32C_S2r2__091.tif]

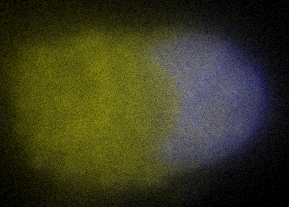

Supplement: Supplementary file 5 — Source Data for Figure 1 [file EMBJ-42-e113168-s008.zip › Figure 1/1A/Images/Replicate 2/32C/32C_S2r2__090.tif]

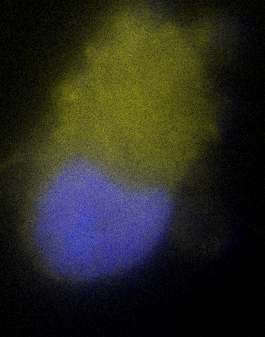

Supplement: Supplementary file 5 — Source Data for Figure 1 [file EMBJ-42-e113168-s008.zip › Figure 1/1A/Images/Replicate 2/32C/32C_S2r2__084.tif]

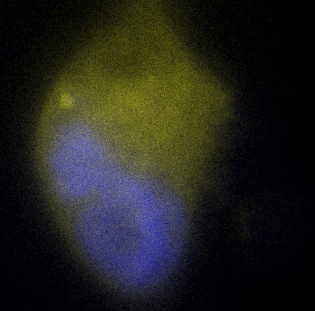

Supplement: Supplementary file 5 — Source Data for Figure 1 [file EMBJ-42-e113168-s008.zip › Figure 1/1A/Images/Replicate 2/32C/32C_S2r2__092.tif]

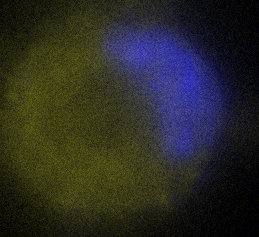

Supplement: Supplementary file 5 — Source Data for Figure 1 [file EMBJ-42-e113168-s008.zip › Figure 1/1A/Images/Replicate 2/32C/32C_S2r2__086.tif]

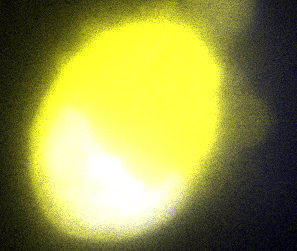

Supplement: Supplementary file 5 — Source Data for Figure 1 [file EMBJ-42-e113168-s008.zip › Figure 1/1A/Images/Replicate 2/32C/32C_S2r2__079.tif]

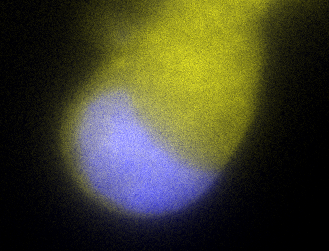

Supplement: Supplementary file 5 — Source Data for Figure 1 [file EMBJ-42-e113168-s008.zip › Figure 1/1A/Images/Replicate 2/32C/32C_S2r2__078.tif]

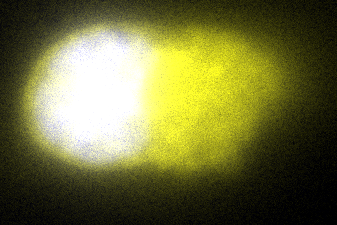

Supplement: Supplementary file 5 — Source Data for Figure 1 [file EMBJ-42-e113168-s008.zip › Figure 1/1A/Images/Replicate 2/32C/32C_S2r2__087.tif]

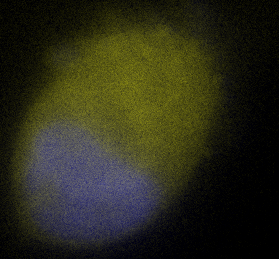

Supplement: Supplementary file 5 — Source Data for Figure 1 [file EMBJ-42-e113168-s008.zip › Figure 1/1A/Images/Replicate 2/32C/32C_S2r2__093.tif]

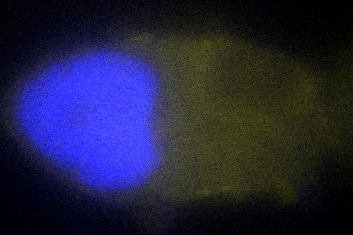

Supplement: Supplementary file 5 — Source Data for Figure 1 [file EMBJ-42-e113168-s008.zip › Figure 1/1A/Images/Replicate 2/32C/32C_S2r2__083.tif]

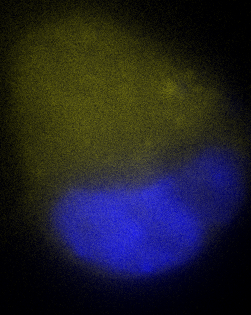

Supplement: Supplementary file 5 — Source Data for Figure 1 [file EMBJ-42-e113168-s008.zip › Figure 1/1A/Images/Replicate 2/32C/32C_S2r2__082.tif]

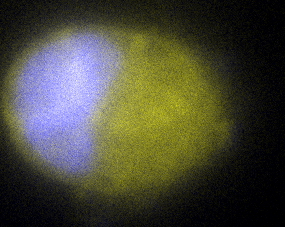

Supplement: Supplementary file 5 — Source Data for Figure 1 [file EMBJ-42-e113168-s008.zip › Figure 1/1A/Images/Replicate 2/32C/32C_S2r2__080.tif]

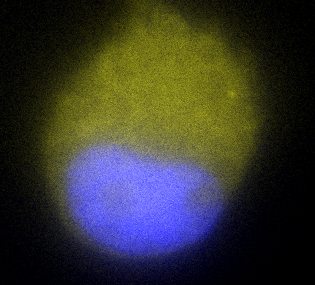

Supplement: Supplementary file 5 — Source Data for Figure 1 [file EMBJ-42-e113168-s008.zip › Figure 1/1A/Images/Replicate 2/32C/32C_S2r2__094.tif]

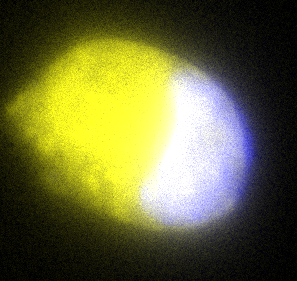

Supplement: Supplementary file 5 — Source Data for Figure 1 [file EMBJ-42-e113168-s008.zip › Figure 1/1A/Images/Replicate 2/32C/32C_S2r2__095.tif]

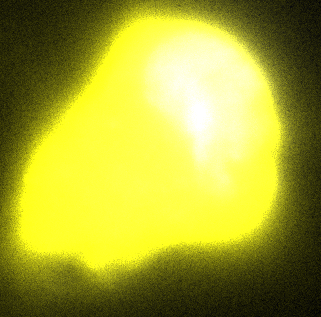

Supplement: Supplementary file 5 — Source Data for Figure 1 [file EMBJ-42-e113168-s008.zip › Figure 1/1A/Images/Replicate 2/32C/32C_S2r2__081.tif]

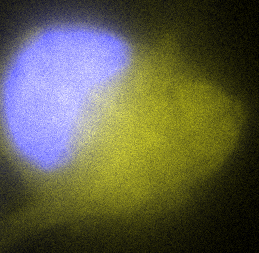

Supplement: Supplementary file 5 — Source Data for Figure 1 [file EMBJ-42-e113168-s008.zip › Figure 1/1A/Images/Replicate 2/32C/32C_S2r1__062.tif]

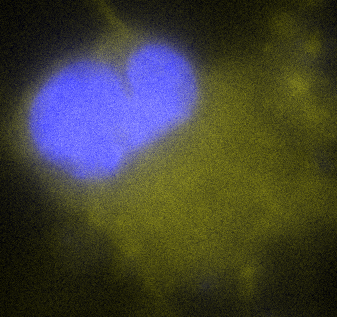

Supplement: Supplementary file 5 — Source Data for Figure 1 [file EMBJ-42-e113168-s008.zip › Figure 1/1A/Images/Replicate 2/32C/32C_S2r1__063.tif]

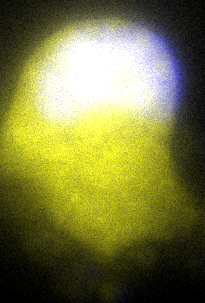

Supplement: Supplementary file 5 — Source Data for Figure 1 [file EMBJ-42-e113168-s008.zip › Figure 1/1A/Images/Replicate 2/32C/32C_S2r1__075.tif]

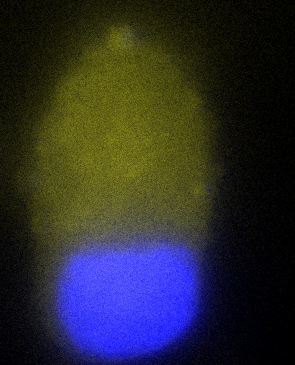

Supplement: Supplementary file 5 — Source Data for Figure 1 [file EMBJ-42-e113168-s008.zip › Figure 1/1A/Images/Replicate 2/32C/32C_S2r1__061.tif]

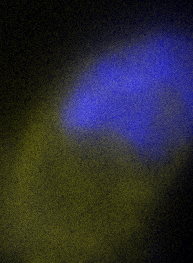

Supplement: Supplementary file 5 — Source Data for Figure 1 [file EMBJ-42-e113168-s008.zip › Figure 1/1A/Images/Replicate 2/32C/32C_S2r1__060.tif]

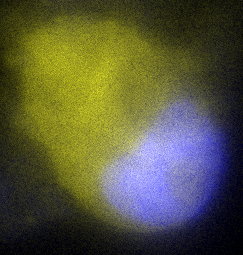

Supplement: Supplementary file 5 — Source Data for Figure 1 [file EMBJ-42-e113168-s008.zip › Figure 1/1A/Images/Replicate 2/32C/32C_S2r1__074.tif]

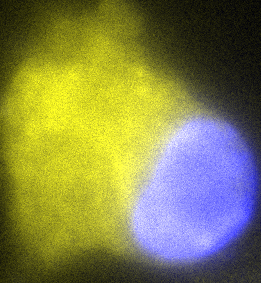

Supplement: Supplementary file 5 — Source Data for Figure 1 [file EMBJ-42-e113168-s008.zip › Figure 1/1A/Images/Replicate 2/32C/32C_S2r1__058.tif]

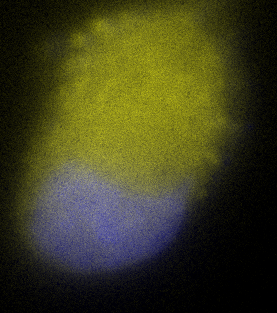

Supplement: Supplementary file 5 — Source Data for Figure 1 [file EMBJ-42-e113168-s008.zip › Figure 1/1A/Images/Replicate 2/32C/32C_S2r1__070.tif]

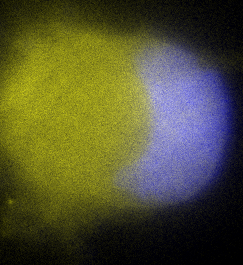

Supplement: Supplementary file 5 — Source Data for Figure 1 [file EMBJ-42-e113168-s008.zip › Figure 1/1A/Images/Replicate 2/32C/32C_S2r1__064.tif]

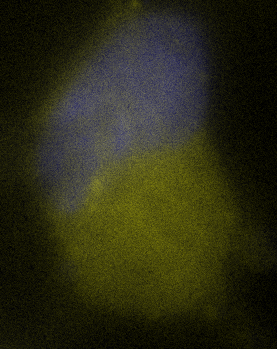

Supplement: Supplementary file 5 — Source Data for Figure 1 [file EMBJ-42-e113168-s008.zip › Figure 1/1A/Images/Replicate 2/32C/32C_S2r1__065.tif]

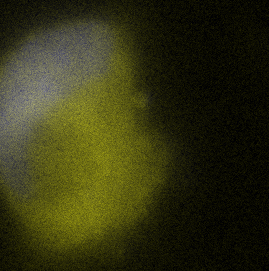

Supplement: Supplementary file 5 — Source Data for Figure 1 [file EMBJ-42-e113168-s008.zip › Figure 1/1A/Images/Replicate 2/32C/32C_S2r1__071.tif]

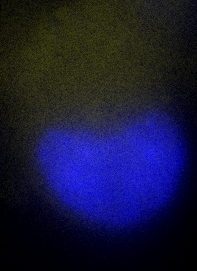

Supplement: Supplementary file 5 — Source Data for Figure 1 [file EMBJ-42-e113168-s008.zip › Figure 1/1A/Images/Replicate 2/32C/32C_S2r1__059.tif]

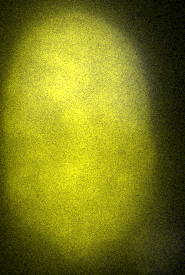

Supplement: Supplementary file 5 — Source Data for Figure 1 [file EMBJ-42-e113168-s008.zip › Figure 1/1A/Images/Replicate 2/32C/32C_S2r1__067.tif]

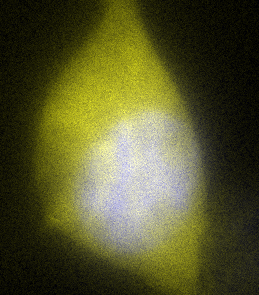

Supplement: Supplementary file 5 — Source Data for Figure 1 [file EMBJ-42-e113168-s008.zip › Figure 1/1A/Images/Replicate 2/32C/32C_S2r1__073.tif]

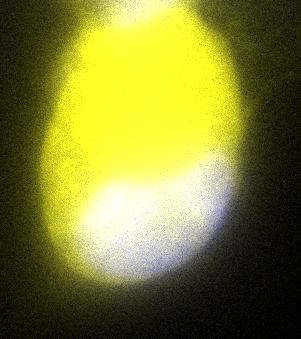

Supplement: Supplementary file 5 — Source Data for Figure 1 [file EMBJ-42-e113168-s008.zip › Figure 1/1A/Images/Replicate 2/32C/32C_S2r1__072.tif]

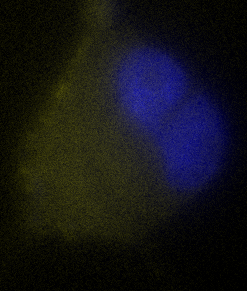

Supplement: Supplementary file 5 — Source Data for Figure 1 [file EMBJ-42-e113168-s008.zip › Figure 1/1A/Images/Replicate 2/32C/32C_S2r1__066.tif]

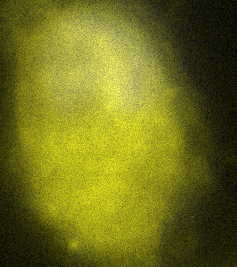

Supplement: Supplementary file 5 — Source Data for Figure 1 [file EMBJ-42-e113168-s008.zip › Figure 1/1A/Images/Replicate 2/37C/37C_S2r2__088.tif]

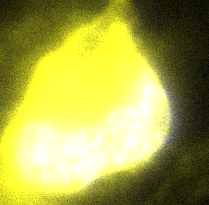

Supplement: Supplementary file 5 — Source Data for Figure 1 [file EMBJ-42-e113168-s008.zip › Figure 1/1A/Images/Replicate 2/37C/37C_S2r1__078.tif]

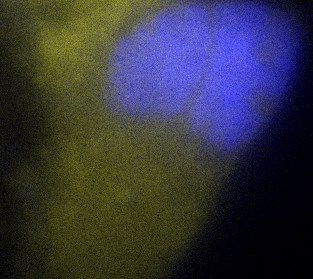

Supplement: Supplementary file 5 — Source Data for Figure 1 [file EMBJ-42-e113168-s008.zip › Figure 1/1A/Images/Replicate 2/37C/37C_S2r1__068.tif]

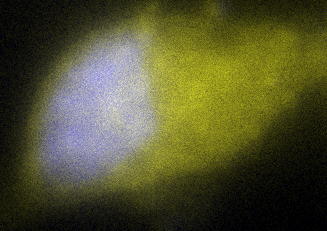

Supplement: Supplementary file 5 — Source Data for Figure 1 [file EMBJ-42-e113168-s008.zip › Figure 1/1A/Images/Replicate 2/37C/37C_S2r1__069.tif]
