# Supplementary material for: HNRNPH1 regulates the neuroprotective cold‐shock protein RBM3 expression through poison exon exclusion
Source: EMBO J. 2023 May 30;42(14):e113168. doi: 10.15252/embj.2022113168 (PMC10350819; doi:10.15252/embj.2022113168)
Supplement: Supplementary file 4 — PDF+ [file EMBJ-42-e113168-s007.pdf]

SOURCE DATA TRANSPARENT PROCESS OPEN ACCESS

THE EMBO JOURNAL

Article

HNRNPH1 regulates the neuroprotective cold-shock protein RBM3 expression through poison exon exclusion

Julie Qiaojin Lin[1,2,*,†], Deepak Khuperkar[1,2,†], Sofia Pavlou[1,3], Stanislaw Makarchuk[1], Nikolaos Patikas[1], Flora CY Lee[2,4], Julia M Zbiegly[1], Jianning Kang[5], Sarah F Field[1,3], David MD Bailey[1], Joshua L Freeman[1,6], Jernej Ule[2,4], Emmanouil Metzakopian[1], Marc-David Ruepp[2,**] & Giovanna R Mallucci[1,6,***]

Abstract

Enhanced expression of the cold-shock protein RNA binding motif 3 (RBM3) is highly neuroprotective both *in vitro* and *in vivo*. Whilst upstream signalling pathways leading to RBM3 expression have been described, the precise molecular mechanism of RBM3 cold induction remains elusive. To identify temperature-dependent modulators of RBM3, we performed a genome-wide CRISPR-Cas9 knockout screen using RBM3-reporter human iPSC-derived neurons. We found that RBM3 mRNA and protein levels are robustly regulated by several splicing factors, with heterogeneous nuclear ribonucleoprotein H1 (HNRNPH1) being the strongest positive regulator. Splicing analysis revealed that moderate hypothermia significantly represses the inclusion of a poison exon, which, when retained, targets the mRNA for nonsense-mediated decay. Importantly, we show that HNRNPH1 mediates this cold-dependent exon skipping via its thermosensitive interaction with a G-rich motif within the poison exon. Our study provides novel mechanistic insights into the regulation of RBM3 and provides further targets for neuroprotective therapeutic strategies.

Keywords alternative splicing; cold-shock protein; CRISPR screen; poison exon; RBM3

Subject Categories Neuroscience; RNA Biology

DOI 10.15252/embj.2022113168 | Received 29 November 2022 | Revised 7 May 2023 | Accepted 11 May 2023 | Published online 30 May 2023

The EMBO Journal (2023) 42: e113168

Introduction

Lowering brain temperature during therapeutic hypothermia is robustly neuroprotective in clinical practice for several patient groups, including neonatal hypoxic–ischemic encephalopathy (Jacobs *et al*, 2011) and cardiac arrest (Arrich & European Resuscitation Council Hypothermia After Cardiac Arrest Registry Study Group, 2007). In preclinical studies, early cooling has been shown to restore memory, prevent synapse and neuronal loss and extend the survival of animal models of prion and Alzheimer's diseases (Peretti *et al*, 2015). This cold-mediated neuroprotection is orchestrated by the elevated expression of a cold-shock protein, RNA-binding motif 3 (RBM3) (Peretti *et al*, 2015, 2021; Bastide *et al*, 2017). In addition, RBM3 stimulates neurogenesis in the rodent brain after hypoxic–ischemic brain injury and improves outcomes *in vivo* (Zhu *et al*, 2019), and protects neuronal cell lines from apoptosis during hypothermia *in vitro* (Chip *et al*, 2011). Clinically, high blood RBM3 levels are associated with good stroke prognosis independent of body temperature (Ávila-Gómez *et al*, 2020). These findings have highlighted RBM3 as an attractive therapeutic target for neuroprotection—with the aim to induce its expression without cooling. However, in this respect, a mechanistic understanding of its regulatory network is required.

In addition to hypothermia, RBM3 expression has been found to be modulated by hypoxia (Wellmann *et al*, 2004; Yan *et al*, 2019), serum starvation (Wellmann *et al*, 2010), metformin (Laustriat *et al*, 2015) and morphine exposure (Koo *et al*, 2012; Lefevre *et al*, 2020). Hypothermia induces RBM3 protein expression in association with TrkB activation *in vivo* (Peretti *et al*, 2021), but the

1 UK Dementia Research Institute and Department of Clinical Neurosciences, University of Cambridge, Cambridge Biomedical Campus, Cambridge, UK
2 UK Dementia Research Institute, King's College London, London, UK
3 Open Targets, Cambridgeshire, UK
4 The Francis Crick Institute, London, UK
5 Tsinghua-Peking Joint Center for Life Sciences, School of Medicine and School of Life Sciences, Tsinghua University, Beijing, China
6 Altos Labs Cambridge Institute of Science, Cambridge, UK
*Corresponding author. Tel: +86 (0) 2088335601; E-mail: qiaojinlin@hkust-gz.edu.cn
**Corresponding author. Tel: +44 (0) 2078485687; E-mail: marc-david.ruepp@kcl.ac.uk
***Corresponding author. Tel: +44 (0) 7766924461; E-mail: gmallucci@altoslabs.com
†These authors contributed equally to this work

© 2023 The Authors. Published under the terms of the CC BY 4.0 license

exact molecular mechanism controlling RBM3 expression downstream of these signalling cascades and other modulators remains unclear. Interestingly, hypothermia, metformin and hypoxia all alter genome-wide alternative splicing (Laustriat *et al*, 2015; Neumann *et al*, 2020; Natua *et al*, 2021) and differential splicing leading to altered mRNA and protein expression, which is common among many RNA-binding proteins (Lareau *et al*, 2007; Sun *et al*, 2017; Müller-McNicoll *et al*, 2019). In particular, hypothermia-induced alternative splicing is observed in cold-inducible RNA-binding protein (CIRBP) transcripts (Gotic *et al*, 2016). These findings raise an interesting possibility that RBM3 gene expression could be fine-tuned on cooling in part by alternative splicing of its mRNA transcripts.

In this study, we uncovered the molecular mechanism involved in the cold induction of RBM3. An unbiased CRISPR/Cas9 whole-genome knockout screen in human-induced pluripotent stem cell (iPSC)-derived neurons (i-neurons) identified several splicing factors, including heterogeneous nuclear ribonucleoprotein H1 (HNRNPH1), as trans-acting regulators of neuronal RBM3. We showed that HNRNPH1 mediates temperature-dependent RBM3 mRNA alternative splicing in multiple cell types and maintains RBM3 transcript and protein expression in cooperation with the nonsense-mediated mRNA decay (NMD) pathway. Additionally, we located temperature-dependent cis-regulatory elements in the RBM3 mRNA and demonstrated that its functional interaction with HNRNPH1 is a key determinant of RBM3 differential splicing. These findings increase the range of therapeutic targets for RBM3 induction.

Results

Pooled CRISPR knockout screen identifies RNA splicing as a key regulatory pathway for RBM3

For the fluorescence-activated cell sorting (FACS)-based whole-genome CRISPR/Cas9 knockout screen, a fluorescence RBM3 reporter iPSC line was generated by Cas9-mediated homology-directed repair to insert GFP at the N-terminus of the single copy of RBM3 on chromosome X in wild-type (WT) iPSCs, which contain a doxycycline (dox)-inducible neurogenin 2 expression cassette (Pawlowski *et al*, 2017) and express Cas9 driven by the GAPDH promoter (Cas9 WT) (Pavlou *et al*, 2023) (Fig EV1A). The iPSCs can be differentiated into excitatory cortical neurons after continuous dox treatment for over 4 days (Pawlowski *et al*, 2017). GFP-RBM3 predominantly localised to the nucleus in iPSCs (Fig EV1B) and i-neurons (Fig 1A), consistent with previous reports in human cortical neurons (Rzechorzek *et al*, 2015). In this study, all Cas9-mediated gene editing in i-neurons was conducted by lentiviral transduction 4 days of postdifferentiation, when Cas9 expression is optimal (Fig EV1A). Editing efficiencies were measured by transducing reporter lentivirus expressing BFP, mCherry and mCherry-targeted single-guide RNA (sgRNA) 4 days after differentiation (Fig EV1C). BFP-positive, Cas9-expressing cells with a reduced mCherry expression, compared with mCherry intensities of BFP-positive WT (Cas9-negative) cells, indicated successful mCherry gene editing (Fig EV1D). 50% editing efficiencies were observed for both GFP-RBM3 clones 4 days after transduction (Day 8) and over 60% on Day 18, similar to Cas9 WT cells (Fig EV1E).

GFP fluorescence intensities in both clones of GFP-RBM3 i-neurons were significantly enhanced by cooling at 32°C for 72 h (Figs 1A and EV1F) as a result of increased nuclear and cytosolic GFP-RBM3 levels (Fig EV1G). 30–40% cold-induction of GFP-RBM3 protein levels was also observed by Western blots (Fig 1B), comparable to the 50% increase in endogenous RBM3 in cooled Cas9 WT i-neurons (Fig EV1H). These observations validated that endogenously GFP-tagged RBM3 is temperature-responsive, consistent with behaviours of unmodified RBM3 both seen with the Cas9 WT cells in this study and reported RBM3 hypothermic induction (Jackson *et al*, 2015; Peretti *et al*, 2015).

Using both clones of this characterised GFP-RBM3 reporter line, we performed an RBM3 CRISPR knockout pooled screen (Fig 1C) by transducing 4-day differentiated cells with a custom-made whole-genome sgRNA lentiviral library, targeting critical exons of 18,466 genes across the human genome, co-expressing a BFP reporter. Day 18 i-neurons were incubated at 32°C for 72 h before dissociation and FACS. BFP-positive cells with the highest and lowest GFP-RBM3 expression, which fell in the top and bottom quartile of GFP intensity profiles, were separately collected, denoted as high GFP and low GFP populations. Genomic DNA was sequenced to identify sgRNAs enriched in high or low GFP populations (see Appendix Table S2 for all significant genes). Ranked by the false discovery rate (FDR), 15 genes, including RBM3, were enriched in

Figure 1. RBM3 CRISPR knockout screen identifies splicing factors as key RBM3 regulators. See also Fig EV1.

A Representative images and quantification of somal intensity per unit area of GFP-RBM3 i-neurons at 37°C or after 72 h cooling at 32°C. Nuclei and cells are outlined by white and yellow dashed lines, respectively. N = 207 (37°C) and 220 (32°C) cells. Scale bar: 5 µm.

B Western blots and quantification of RBM3 and GFP normalised to GAPDH in GFP-RBM3 i-neurons at 37 or 32°C (72 h).

C Schematic of experimental steps in RBM3 CRISPR screen in i-neurons. GFP-RBM3 iPSCs stably expressing Cas9 after 4 days of Dox-induced differentiation are transduced with a whole-genome lentiviral sgRNA library expressing a BFP reporter. 10–12 days after transduction, the i-neuron cultures are incubated at 32°C for 72 h, followed by FACS to sort BFP-positive i-neurons with the highest and lowest 25% GFP fluorescence intensity into separate pools. N = 2 GFP-RBM3 clones and 3 biological replicates.

D, E Top 100 RBM3 positive regulator candidates with their sgRNAs enriched in the low-GFP i-neuron pool (D). Top 100 RBM3 negative regulator candidates with their sgRNAs enriched in the high-GFP i-neuron pool (E). Genes ranked by statistical significance (FDR). Horizontal dashed line: FDR = 0.05.

F The top-ranked Gene Ontology terms and STRING networks of 14 positive regulator candidates (FDR <0.05, RBM3 is excluded). Genes related to RNA splicing are indicated in blue.

G The top-ranked Gene Ontology terms and STRING networks of 95 positive regulator candidates (FDR <0.05). Genes related to RNA splicing are coloured in blue. Genes involved in deubiquitination or proteasome-mediated ubiquitin-dependent protein catabolic processes are coloured in orange.

Data information: N = 3 biological replicates. Mean ± SEM; *(P < 0.05), **(P < 0.01), ***(P < 0.001); unpaired t-tests in (A) and (B).
Source data are available online for this figure.

 © 2023 The Authors

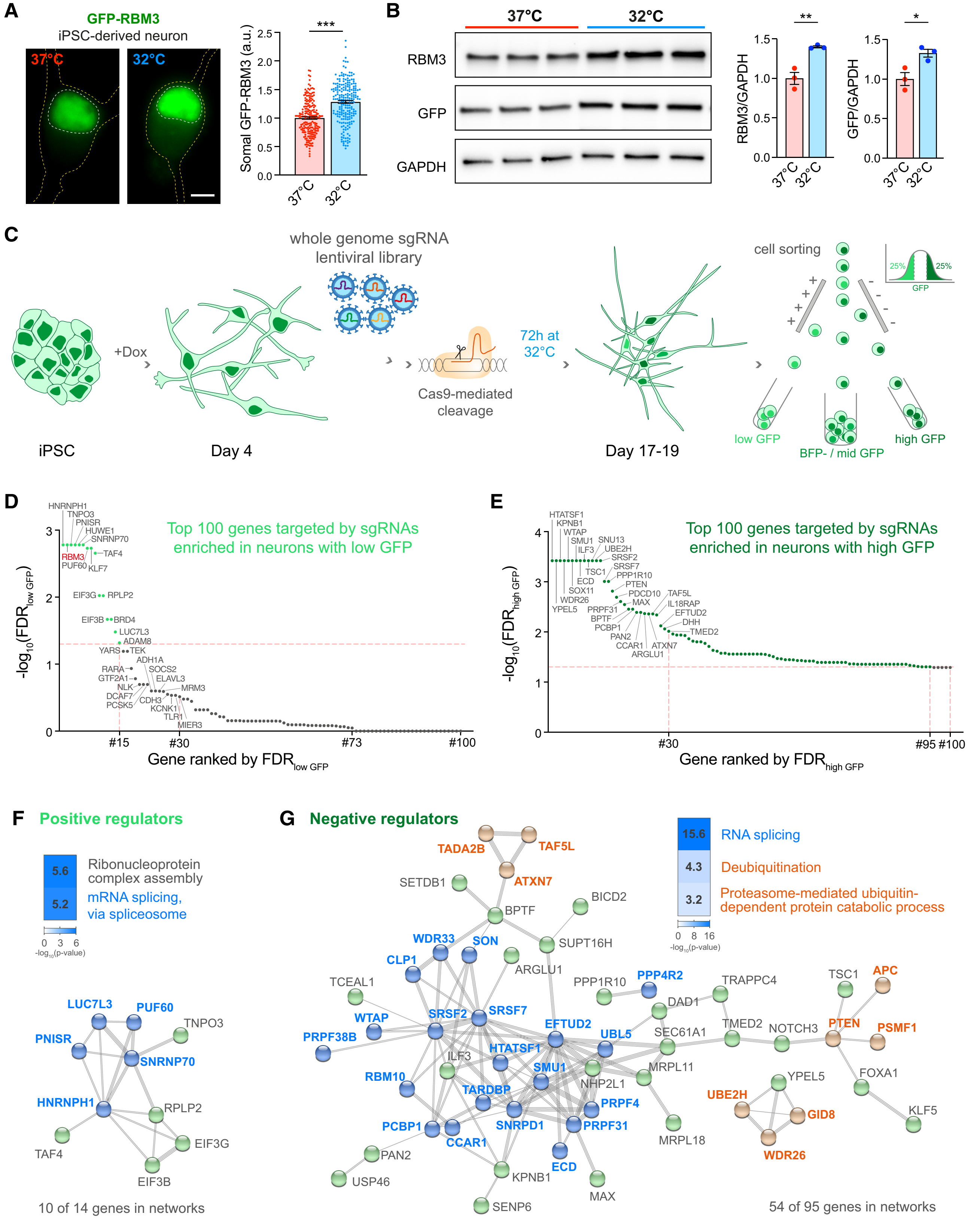
A
GFP-RBM3
iPSC-derived neuron
37°C
32°C
Somal GFP-RBM3 (a.u.)
***
B
37°C
32°C
RBM3
GFP
GAPDH
RBM3/GAPDH
GFP/GAPDH
**
*
C
whole genome sgRNA lentiviral library
+Dox
Cas9-mediated cleavage
72h at 32°C
cell sorting
GFP
25%
25%
low GFP
BFP- / mid GFP
high GFP
iPSC
Day 4
Day 17-19
D
Top 100 genes targeted by sgRNAs enriched in neurons with low GFP
Gene ranked by FDR low GFP
E
Top 100 genes targeted by sgRNAs enriched in neurons with high GFP
Gene ranked by FDR high GFP
F
Positive regulators
Ribonucleoprotein complex assembly
mRNA splicing, via spliceosome
10 of 14 genes in networks
G
Negative regulators
RNA splicing
Deubiquitination
Proteasome-mediated ubiquitin-dependent protein catabolic process
54 of 95 genes in networks

Figure 1.

low GFP i-neurons, suggesting they likely positively regulate RBM3 expression (Fig 1D). In contrast, 95 genes, likely to negatively affect RBM3 expression, were enriched in high GFP populations (Fig 1E).

Gene ontology (GO) and network analysis revealed that a major group of RBM3 regulator candidates are involved in RNA splicing (Fig 1F and G), including spliceosomal proteins, for example U1 small nuclear ribonucleoprotein 70 kDa (SNRNP70), heterogeneous nuclear ribonucleoproteins (hnRNPs) and serine and arginine rich splicing factors (SRSFs). Other potential RBM3 regulators play roles in nucleocytoplasmic transport, for example transportin 3 (TNPO3) and karyopherin-β1 (KPBN1); translation, for example 60S acidic ribosomal protein P2 (RPLP2) and eukaryotic translation initiation factor 3 subunit B (EIF3B); transcription, for example transcription initiation factor TFIID subunit 4 (TAF4); ubiquitination, for example ubiquitin-conjugating enzyme E2 H (UBE2H) (see Appendix Table S3 for a full list of GO terms).

Arrayed target validation reveals regulators modulating neuronal RBM3 protein and transcript abundance

To validate the top hits identified by the pooled screen, we individually depleted the top 30 RBM3-positive and -negative regulator candidates using 1–3 sgRNAs per gene from the whole-genome library (see Appendix Table S4). I-neurons transduced with three nontargeting sgRNAs from the pooled library or the reporter lentivirus for editing efficiency calculation served as controls. As a positive control, RBM3 sgRNAs reduced GFP fluorescence by over 80% compared with nontargeting sgRNAs at 37 or 32°C (Fig 2A). Moderate spectral crossover and activation of specific signalling pathways due to target-specific genome editing may account for the discrepancy between nontargeting sgRNA and reporter controls. To apply a more stringent standard, we performed statistical analysis between specific gene knockout (KO) groups and one of the two control groups with high P-values, for instance, comparing to the nontargeting sgRNA control showing lower GFP intensity to identify positive regulators, and to the reporter control for negative regulators. Knocking out seven out of 29 positive regulator candidates tested significantly reduced GFP-RBM3 levels at 37 and 32°C (Fig 2A). HNRNPH1 (Chou *et al*, 1999), TNPO3 (Kataoka *et al*, 1999), PNN Interacting Serine And Arginine Rich Protein (PNISR) (Zimowska *et al*, 2003) and SNRNP70 (Pomeranz Krummel *et al*, 2009) are associated with RNA splicing. Among 30 tested RBM3-negative regulator candidates, KO of six genes significantly increased GFP-RBM3 expression in i-neurons at 37 and 32°C (Fig 2B). Specifically, HIV-1 Tat-Specific Factor 1 (HTATSF1) and WT1-Associated Protein (WTAP) are splicing regulators, while Yippee Like 5 (YEPL5), WD Repeat Domain 26 (WDR26) and UBE2H mediate ubiquitination and proteasomal degradation, the depletion of which is more likely to result in generic instead of RBM3-specific protein accumulation. Changes in GFP-RBM3 levels upon selective RBM3 regulator KO were also confirmed using fluorescence microscopy (Fig EV2A). Collectively, the arrayed CRISPR knockout assay validated hits associated with the lowest FDR in the pooled screen (Fig 1D and E), and reiterated that multiple components of the splicing machinery are key to RBM3 protein expression.

To investigate whether the cold-induced GFP-RBM3 protein expression is controlled at the translational or transcriptional level, the translation inhibitor cycloheximide or the RNA polymerase inhibitor actinomycin D was applied to i-neurons at 37 or 32°C. The elevation of GFP-RBM3 in cooled cells was completely abolished by either inhibitor (Fig EV2B), indicating the cold-increased GFP-RBM3 expression relies on the *de novo* transcription and translation of RBM3 transcripts. In line with this finding, differential expression analysis comparing RNA-Seq data between i-neurons at 37 and 32°C showed RBM3 increased fourfold on cooling: this was the most significant increase across the entire transcriptome (Fig EV2C). Furthermore, real-time PCR (quantitative PCR, qPCR) of RBM3 mRNA supports cold-induction of RBM3 mRNA by 3–5 folds in Cas9 WT i-neurons (Figs 2C and EV2D) and HeLa cells (Fig EV2F), without apparent transcriptional activation as RBM3 pre-mRNA levels remained unchanged upon cooling (Fig EV2E and G).

We next explored whether the changes in RBM3 protein expression upon individual regulator KO were due to altered RBM3 mRNA levels. We focussed on the validated splicing regulating genes (HNRNPH1, PNISR, SNRNP70, HTATSF1, WTAP), potential regulators marginally affecting RBM3 expression (Poly(U) Binding Splicing Factor 60 (PUF60) and LUC7 Like 3 Pre-mRNA Splicing Factor (LUC7L3)) and the two nuclear protein importers (TNPO3 and KPNB1) (Fig 2D and E). Due to cell death and promoter silencing, only 15–45% of the transduced cells remained BFP-positive, as approximates to transduction efficiencies (labelled on bars, Fig 2D and E), on Day 18 when RNA was extracted from i-neuron cultures at 37 or 32°C. Remarkably, the extent of RBM3 transcript reduction due to HNRNPH1 KO at 37 or 32°C was similar to RBM3 KO as a positive control (Fig 2D and E), revealing HNRNPH1 as a strong positive regulator of RBM3 mRNA expression. TNPO3, PNISR, SNRNP70 and PUF60 KO also significantly lowered RBM3 transcript levels at 32°C (Fig 2E), suggesting they are key regulators for cold induction of RBM3 transcripts. On the contrary, none of the tested negative regulator KO affected RBM3 mRNA expression, which

Figure 2. Depleting RBM3 positive regulators that function in RNA splicing reduces RBM3 protein and mRNA levels. See also Fig EV2.

A, B Median GFP intensity of BFP-positive GFP-RBM3 i-neurons measured by flow cytometry upon the sgRNA/Cas9-mediated KO of top 30 positive (A) or negative (B) regulator candidates. Statistical analysis is performed between the specific and non-targeting sgRNA groups for positive regulators (A) or between the specific sgRNA and reporter groups for negative regulators (B) within the 37 or 32°C (72 h) population.

C qRT-PCR of RBM3 mRNA level normalised to 18 s rRNA in i-neurons at 37 or 32°C (72 h).

D, E qRT-PCR of RBM3 mRNA level normalised to 18 s rRNA in GFP-RBM3 i-neurons at 37°C (D) or 32°C for 72 h (E) transduced with lentivirus containing specific, non-targeting sgRNA or the reporter. Statistical analysis is performed between the specific and non-targeting sgRNA for negative regulators in (D) and positive regulators in (E), or between the specific and reporter groups for positive regulators in (D) and negative regulators in (E). Transduction efficiencies are indicated in corresponding bars.

Data information: $N = 3$ biological replicates. Each data point represents one well of culture. Mean ± SEM; *($P < 0.05$), **($P < 0.01$), ***($P < 0.001$); One-way ANOVA with multiple comparisons in (A), (B), (D), (E), unpaired *t*-tests in (C).
Source data are available online for this figure.

 © 2023 The Authors

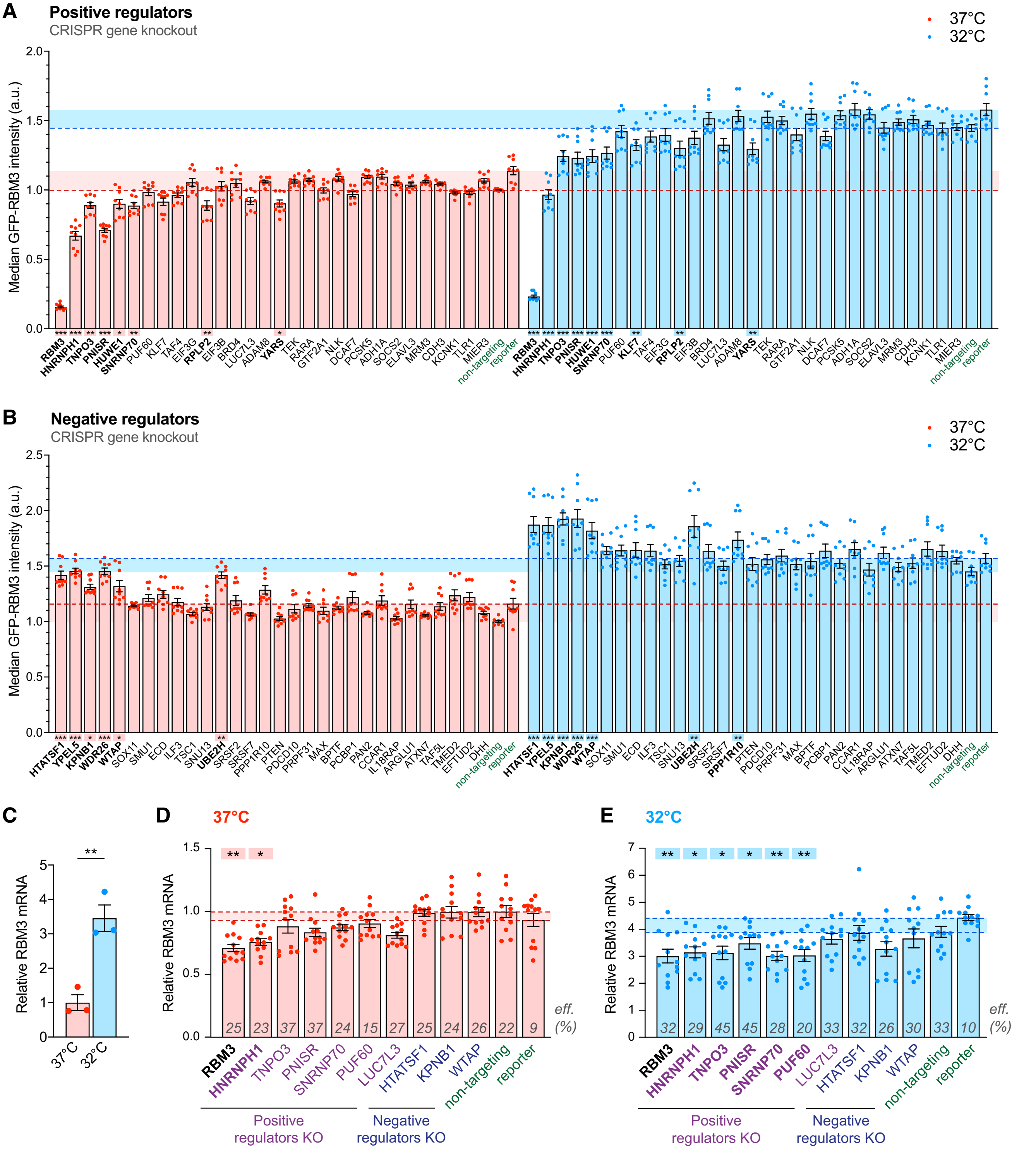

Figure 2.

implies that their regulation may be at the translational or post-translational level. In support of our findings in i-neurons, RNA-Seq analysis of public data also showed reduced RBM3 mRNA levels upon HNRNPH1, SNRNP70 and PUF60 KD in K562 or HepG2 cells, while no difference was seen upon KPNB1 KD (Fig EV2H; Appendix Table S5) (ENCODE Project Consortium, 2012; Luo *et al*, 2020). In HNRNPH1-knocked down HeLa cells, RBM3 is among the top 5% downregulated genes detected by RNA-Seq and proteomics, with its mRNA and protein levels decreased by 3 and 0.55 folds, respectively (Uren *et al*, 2016), demonstrating a strong regulatory effect of HNRNPH1 on RBM3 transcripts and proteins. We further showed that HNRNPH1 KD significantly decreased RBM3 mRNA in HeLa

© 2023 The Authors

cells by over 50% both at 37 and 32°C (Fig EV2I). To summarise, depleting specific splicing factors, particularly HNRNPH1, abolishes the cold-induced increase of RBM3 mRNA expression in i-neurons and other cell types.

RBM3 poison exon that triggers nonsense-mediated decay is silenced during hypothermia

Given that RBM3 mRNA expression is tightly regulated by splicing factors in i-neurons, we searched for RBM3 alternatively spliced isoforms with RNA-Seq data acquired from Cas9 WT i-neurons at 37 and 32°C (Fig 3A). Multiple isoforms of RBM3 transcripts were identified and the most abundant four isoforms could be distinguished by either Exon 3, Exon 3a-L or Exon 3a-S inclusion (Fig 3A). Differential splicing analysis showed RBM3 Exon 3a inclusion levels drastically reduced upon cooling, from the percent spliced in (PSI) index of 0.023 to 0.002 for Exon 3a-L, or from 0.015 to 0.001 for Exon 3a-S (Fig 3B; Appendix Table S6), while inclusion levels of Exon 3 and constitutive Exons 4 and 5 remained unchanged at 37 and 32°C (Figs 3B and EV3A; Appendix Table S6), demonstrating that cooling effectively repressed the production of Exon 3a-L/S-containing RBM3 transcripts.

A close examination of Exon 3a of RBM3 revealed multiple stop codons in-frame with the coding sequence, potentially leading to premature translational termination (Llorian *et al*, 2016). Thus, the inclusion of Exon 3a makes the RBM3 transcript a susceptible target for degradation by nonsense-mediated mRNA decay (NMD), an mRNA surveillance pathway that degrades mRNAs that harbour premature termination codons (PTCs) (He & Jacobson, 2015). Exon 3a could therefore serve as a poison exon (PE) in RBM3 mRNA, leading to a reduction of the transcript level if retained. Such post-transcriptional regulation mediated by PEs is key to fine-tuning expression levels for many proteins, especially RNA-binding proteins (Neumann *et al*, 2020). To further verify whether Exon 3a indeed acts as a PE, and to explore the temperature-dependent inclusion of RBM3 PE quantitatively, we designed isoform-sensitive primers to amplify regions between RBM3 exon 3 and exon 4 to identify PE-included or PE-skipped isoforms of RBM3 transcripts (Fig 3C). In order to detect the NMD-sensitive PE-included isoforms, we blocked NMD using a chemical inhibitor of SMG1 kinase, a key member of the NMD pathway (Langer *et al*, 2021). When i-neurons were incubated with SMG1 inhibitor for 24 h at 37°C, both PE-included and PE-skipped isoforms were detected with RT-PCR. Interestingly, in 72 h-cooled i-neurons, only the PE-skipped isoform was detected, even after SMG1 inhibitor treatment, clearly supporting that the RBM3 PE was preferentially excluded in cooled i-neurons (Fig 3D). This finding was further validated by qPCR using primers against Exon 3a (detecting both 3a-L and 3a-S) and the constitutive exons, for example Exon 3 and Exon 4–5 (Fig 3E). The fraction of PE-contained transcripts to the total transcripts at 37°C was over five times more compared with the fraction at 32°C. When NMD was inhibited by the SMG1 inhibitor, the differences rose to 13–15 folds (Fig 3E).

Likewise, PE of endogenous RBM3 mRNA was excluded in NMD-inhibited HeLa cells on cooling (Fig 3F). Additionally, an RBM3 minigene spanning RBM3 Exons 1–4, which was sensitive to NMD when expressed in HeLa cells, also showed a significantly lower fraction of PE-retained isoform at 32°C compared to 37°C, when NMD was blocked by SMG1 inhibitor (Fig 3G) or cycloheximide (Fig EV3B).

HNRNPH1 represses RBM3 poison exon inclusion at low temperatures

We next investigated whether positive RBM3 mRNA regulators control RBM3 transcript abundance through PE skipping. When NMD was inhibited, knocking down HNRNPH1 (Fig EV4A) resulted in RBM3 PE retention in Cas9 WT i-neurons, with a 29% increase at 37°C and a 57% increase at 32°C compared with the respective nontargeting control (Fig 4A and B). RBM3 PE-retention upon HNRNPH1 KD was also found in published RNA-Seq analysis using K562 and HepG2 cells, demonstrating its conserved role across multiple cell types (Figs 4C and EV4B and C; Appendix Table S6) (ENCODE Project Consortium, 2012; Luo *et al*, 2020). In HeLa cells treated with SMG1 inhibitor, RNAi-mediated HNRNPH1 knockdown (Fig EV4D) increased PE retention in endogenous RBM3 transcripts (Fig 4D) and in RBM3 minigene (Fig 4E) only at 32°C when NMD was inhibited, suggesting that the role of HNRNPH1 in repressing RBM3 PE inclusion is context-dependent, being more important under the cooled condition. Moreover, overexpression of FLAG-tagged HNRNPH1 in HEK293T cells (Fig EV4E) repressed PE inclusion in endogenous RBM3 mRNA (Fig 4F) and in the minigene

Figure 3. Hypothermia represses RBM3 mRNA poison exon inclusion. See also Fig EV3.

A Sashimi plots of RBM3 transcripts in WT i-neurons at 37 and 32°C (72 h), showing major alternatively spliced isoforms. Differentially spliced Exon 3a-L and 3a-S junctions between 37 and 32°C conditions are shown in red. $N = 4$.

B PSI values of RBM3 Exon 3a-L and 3a-S relative to Exon 3 and 4, and Exon 3 relative to Exon 2 and 4 in i-neurons at 37 or 32°C (72 h).

C Schematics of RBM3 Exon 3a, or poison exon (PE), alternative splicing and the resulting PE-included (left) or PE-skipped (right) mRNA products. RT-PCR primer pair amplifying Exon 2–4 are indicated by grey arrows.

D RT-PCR of RBM3 mRNA (Exon 2–4) in i-neurons at 37 or 32°C (72 h) in the presence or absence of SMG1 inhibitor. PSI values of RBM3 PE are calculated based on the intensity of PE-included (red arrows) and PE-skipped (green arrow) isoforms.

E qRT-PCR using a combination of primers targeting Exon 3a, Exon 3 or Exon 4–5 quantifies the PSI values of RBM3 PE (Exon 3a, including both 3a-L and 3a-S) relative to Exon 3 or Exon 4–5 at 37 or 32°C (72 h) in the presence or absence of SMG1 inhibitor.

F RT-PCR of RBM3 mRNA (Exon 2–4) in HeLa cells at 37 and 32°C (48 h) in the presence or absence of SMG1 inhibitor. PSI values of RBM3 PE are depicted in the graph on the right.

G Schematics and RT-PCR of RBM3 minigene (Exon 1–4), flanked by unique sequences (thick black bars) to distinguish it from endogenous transcripts during PCR amplification, expressed in HeLa cells at 37 or 32°C (48 h), in the presence or absence of SMG1 inhibitor. PSI values of RBM3 PE are depicted in the graph on the right.

Data information: $N = 4$ biological replicates in (A), (B) and (F), $N = 3$ in (D), (E) and (G). Mean ± SEM; n.s. (not significant), *($P < 0.05$), **($P < 0.01$), ***($P < 0.001$); FDR calculated by rMATS program in (B); unpaired t-tests in (E); paired t-tests in (D), (F), (G).
Source data are available online for this figure.

 © 2023 The Authors

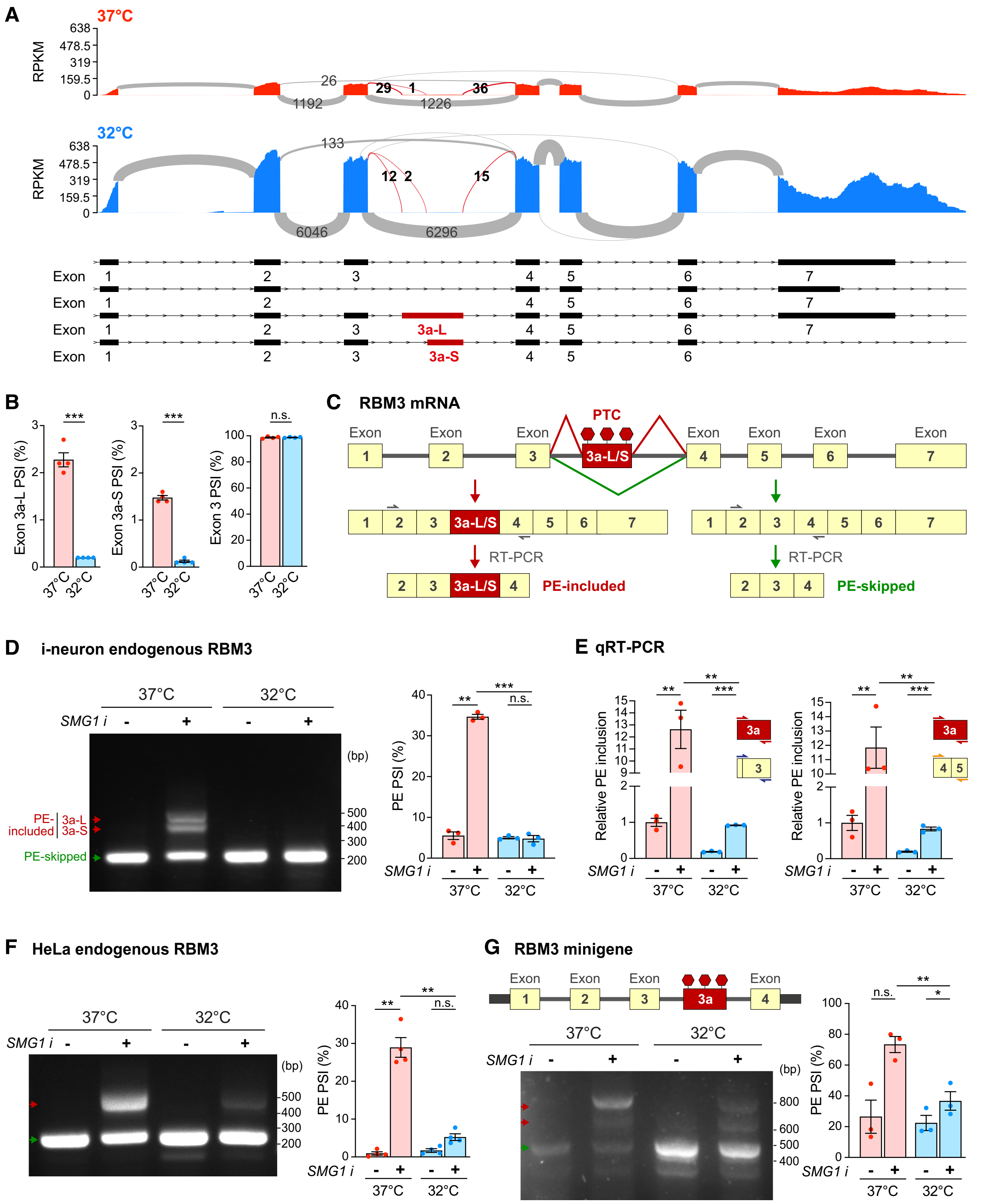

Figure 3.

(Fig 4G) at 37°C, and a comparable extent of PE skipping was observed in HNRNPH1-overexpressing i-neurons at 37 and 32°C (Figs 4H and EV4F–H), confirming the splicing-regulatory function of HNRNPH1 in RBM3 PE exclusion. As expected, overexpression of HNRNPH1 in GFP-RBM3 i-neurons with HNRNPH1-T2A-BFP lentiviral transduction resulted in GFP-RBM3 upregulation at 37 and

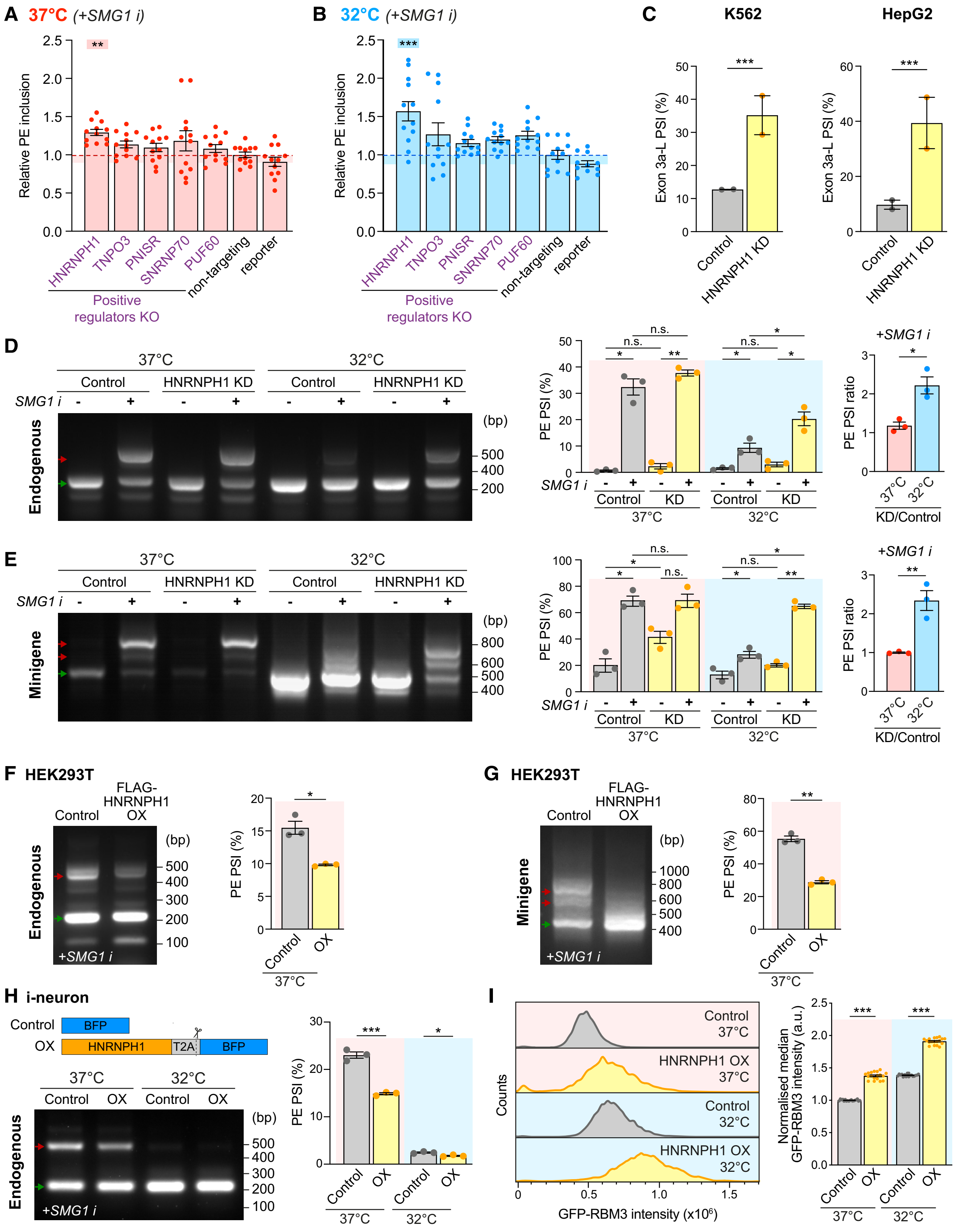

Figure 4.

© 2023 The Authors

Figure 4. HNRNPH1 increases RBM3 expression by promoting poison exon skipping. See also Fig EV4.

A, B qRT-PCR quantifying the PSI values of RBM3 PE relative to RBM3 mRNA in WT i-neurons at 37°C (A) or 32°C (72 h) (B), when NMD is blocked by SMG1 inhibitor. Statistical analysis is performed between the specific and non-targeting sgRNA groups.

C PSI values of RBM3 Exon 3a-L in control and HNRNPH1-knocked down K562 and HepG2 cells. RNA-Seq data from ENCODE Project, 2 isogenic replicates are included.

D, E RT-PCR of endogenous RBM3 (D) and expressed RBM3 minigene (E) in control (scramble siRNA) or HNRNPH1-knocked down HeLa cells at 37 or 32°C (48 h), in the presence or absence of SMG1 inhibitor. The ratio of PSI values between HNRNPH1 KD and control is shown for only the SMG1 i-treated conditions.

F, G RT-PCR of endogenous RBM3 (F) and expressed RBM3 minigene (G) in SMG1 inhibitor-treated control (untransfected) or FLAG-HNRNPH1-overexpressing (OX) HEK293T cells at 37°C. PSI values of RBM3 PE are shown in the graphs on the right respectively.

H WT i-neurons are transduced with lentiviral constructs expressing BFP (Control) or HNRNPH1-T2A-BFP (HNRNPH1 overexpression, OX) at 37 and 32°C, followed by RT-PCR of endogenous RBM3 with 24 h SMG1 inhibitor treatment. PSI values of RBM3 PE are shown in the graph on the right.

I GFP-RBM3 intensity histogram and control (37°C)-normalised median GFP intensity indicating GFP intensity of control and HNRNPH1-overexpressing (OX) GFP-RBM3 i-neurons measured by flow cytometry. Only cells with top 5% BFP levels among all BFP-positive cells in the well are included.

Data information: $N = 3$ biological replicates. Each data point represents one well of culture in (A) and (I). Mean ± SEM; n.s. (not significant), *($P < 0.05$), **($P < 0.01$), ***($P < 0.001$); one-way ANOVA with multiple comparisons in (A), (B); FDR calculated by rMATS program in (C), paired *t*-tests in (D–G), unpaired *t*-tests in (H), (I).
Source data are available online for this figure.

32°C, compared to the BFP control (Fig EV4I). While moderate HNRNPH1 overexpression led to limited GFP-RBM3 increases (Fig EV4J, grey data points), i-neurons associated with high levels of HNRNPH1-T2A-BFP expression (Fig EV4J, coloured data points indicate cells with top 5% BFP levels) showed significant GFP-RBM3 elevation (Fig 4I, only cells with top 5% BFP levels are shown).

Given the strong correlation between HNRNPH1 and RBM3 expression, we speculated that HNRNPH1 expression is also cold-inducible. To our surprise, HNRNPH1 protein levels, its interaction with spliceosomes shown by core spliceosomal protein SmB pull-down, and its nucleo-cytoplasmic localisation were not altered by cooling (Figs 5A and EV5A–C), suggesting that cooling-related regulation of RBM3 mRNA by HNRNPH1 is not driven by generic changes in HNRNPH1 spliceosomal association. Next, to explore the temperature-dependent physical association between HNRNPH1 and RBM3 mRNA, we performed HNRNPH1 RNA immunoprecipitation (RIP) in HeLa cells, which were incubated at 37 or 32°C and supplemented with SMG1 inhibitor. We found a twofold increase in the RBM3 mRNA levels in HNRNPH1 immunoprecipitates at 32°C compared to 37°C (Figs 5B and EV5D and E), indicating an increased interaction between HNRNPH1 and RBM3 mRNA on cooling.

With evidence for a cold-enhanced HNRNPH1-RBM3 mRNA interaction, we next searched for potential HNRNPH1 binding sites around RBM3 PE that regulate its alternative splicing. The intronic sequence between RBM3 Exon 3 and 4 contains multiple poly-G stretches with potentials to form RNA G-quadruplex structures (rG4s) (Kharel *et al*, 2020) (Fig 5C), which are known as HNRNPH1 consensus binding sites (Caputi & Zahler, 2001; Uren *et al*, 2016). Indeed, analysis of published HNRNPH1 Individual-nucleotide resolution UV crosslinking and immunoprecipitation (iCLIP) data set in HEK293T cells (Braun *et al*, 2018) revealed that HNRNPH1 strongly interacts with GGGG motifs at the 5′ end of PE (near 3′ splice site) and 30 nucleotides upstream of the 5′ splice site between RBM3 Exon 3 and 4 (Fig 5C). Strikingly, the removal of this single GGGG motif, which is predicted to disrupt the rG4 (Fig EV5F), was sufficient to dramatically increase PE inclusion and almost completely incapacitate its skipping upon cooling, but not at 37°C (Fig 5D), recapitulating PE inclusion due to HNRNPH1 KD at 32°C (Fig 4E). Unlike in RBM3 WT minigene, HNRNPH1 overexpression in HEK293T cells at 37°C was unable to repress PE inclusion in the delGGGG minigene when the primary HNRNPH1 binding site was abolished (Fig EV5G). Taken together, the results show that HNRNPH1 interacts with the G-rich motif within the PE of RBM3 mRNA to repress PE inclusion most efficiently upon cooling.

Discussion

Using a genome-wide CRISPR/Cas9 gene KO screen, we identified key transregulatory factors for neuronal RBM3 cold induction. The strength of our screen lies in its closely recapitulating the physiological scenario, because we chose to (i) GFP-tag endogenous RBM3 loci to account for any regulatory element beyond the coding sequence, (ii) use differentiated i-neurons with functional synapses, and (iii) precool at 32°C to extend the dynamic range of GFP-RBM3 fluorescent readings affected by positive and negative regulator depletion. While splicing regulating proteins were identified among the strongest RBM3 regulators, our screen also indicated that RBM3 protein expression is controlled at multiple levels, from transcription, translation to protein degradation. It will be interesting to explore the overlap between cooling-dependent modulators of RBM3 identified in this study and regulators involved in changes in RBM3 levels by other known stimuli, such as BDNF/TrkB signalling cascade we previously reported (Peretti *et al*, 2021).

The appearance of HNRNPH1 as the top positive regulator of RBM3 together with other splicing factors prompted us to investigate cold-induced splicing changes in RBM3 mRNA. In line with this, we report cooling-dependent PE exclusion as a level of regulation governing RBM3 induction. Interestingly, this alternative splicing regulation is conserved in different cell types and between human and mouse (Preußner *et al*, 2023). Depletion of HNRNPH1 using different methods disrupted the alternative splicing control around RBM3 PE in endogenous RBM3 mRNA and in an externally introduced minigene. Moreover, removal of the poly-G stretches within the PE significantly repressed the removal of PE in RBM3 transcript, further conclusively establishing the role of HNRNPH1 binding.

Although HNRNPH1 KD resulted in a proportionally similar reduction in RBM3 mRNA and protein expression at 37 and 32°C (Figs 2A, D, and E, and EV2I), it is interesting that depletion of HNRNPH1 enhanced RBM3 PE retention at 32°C but exerted little impact at 37°C in HeLa cells (Fig 4D and E), supporting a cold-

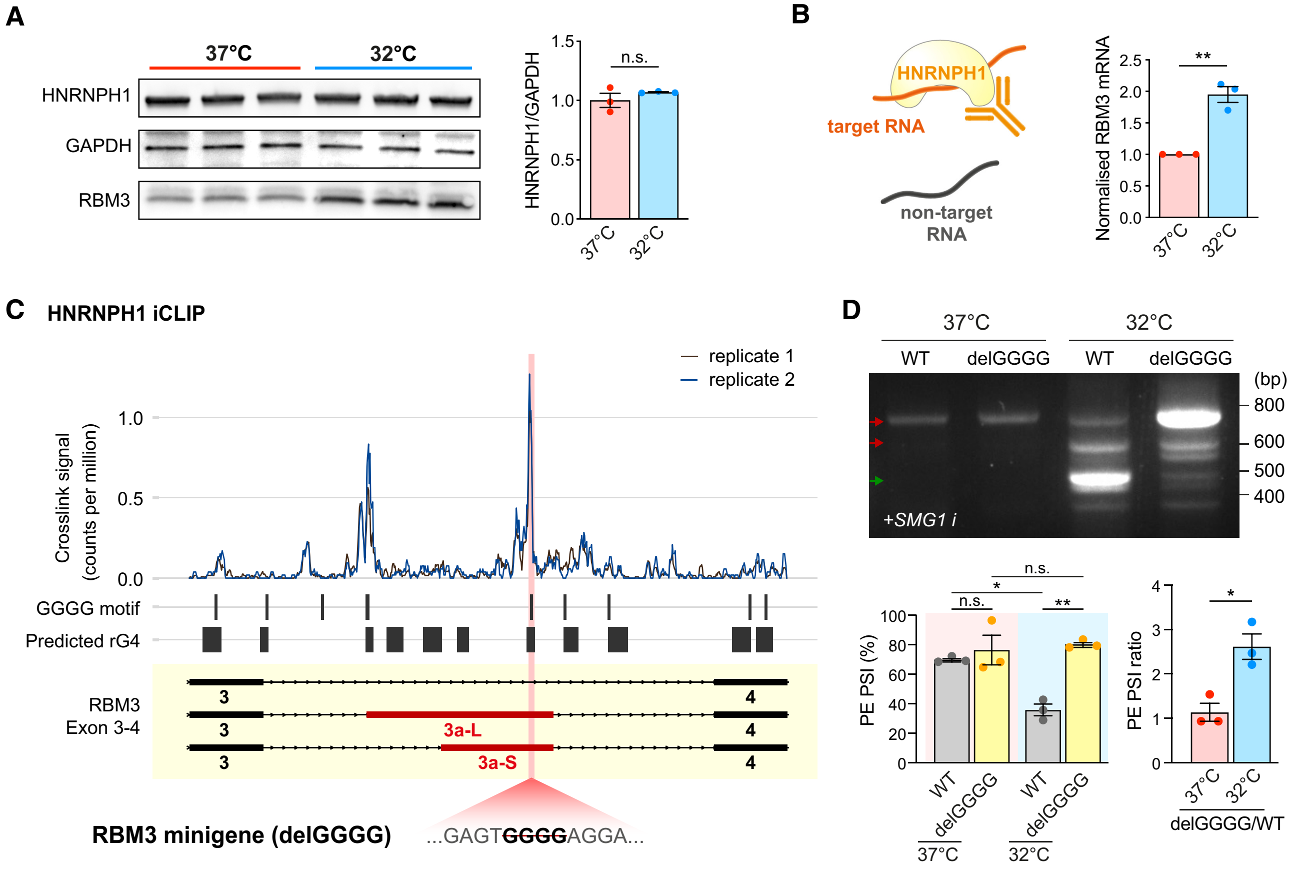

Figure 5. HNRNPH1 interacts with G-rich sequences in RBM3 poison exon in a temperature-dependent manner. See also Fig EV5.

A Western blot and quantification of HNRNPH1 normalised to GAPDH in WT i-neurons at 37 and 32°C (72 h). RBM3 blots of the same samples are shown for comparison.

B Schematic of HNRNPH1 RNA Immunoprecipitation (RIP) in HeLa cells at 37 or 32°C. The graph on the right shows the fold change in HNRNPH1-pulled down RBM3 mRNA after normalisation.

C Analysis of public HNRNPH1 iCLIP dataset in two replicates, mapped to RBM3 Exon 3–4. Crosslink counts are normalised to library size. RNA G quadruplexes (rG4) are predicted using QGRS mapper. The position of the GGGG motif deleted in the mutant RBM3 minigene is shown in pink.

D RT-PCR of WT and delGGGG RBM3 minigenes in HeLa cells at 37 or 32°C (48 h) treated with SMG1 inhibitor. PSI values of RBM3 PE are shown in the graphs on the right.

Data information: $N = 3$ biological replicates. Mean ± SEM; n.s. (not significant), *($P < 0.05$), **($P < 0.01$); unpaired *t*-tests in (A), (B), (F), paired *t*-tests in (D).
Source data are available online for this figure.

dependent efficacy of HNRNPH1 in RBM3 PE repression. The discrepancy between the temperature-independent regulation of RBM3 expression and the temperature-dependent control of RBM3 PE inclusion by HNRNPH1 may be a result of nonsplicing regulatory factors, such as the NMD pathway, which may also function with different capacities in a temperature-sensitive manner. Notably, the extent of HNRNPH1-mediated PE repression also varies between different cell types: in HeLa cells, HNRNPH1 KD only led to PE inclusion at 32°C (Fig 4D and E), while HNRNPH1 depletion in i-neurons enhanced PE inclusion by 30% at 37°C and > 50% at 32°C (Fig 4A and B). The variable endogenous protein levels of HNRNPH1 and other relevant RBPs may contribute to the cross-cell-type differences.

The lower efficiency of HNRNPH1 in RBM3 PE repression at 37°C can be compensated by its overexpression (Fig 4F–H), but for now it remains unclear why it is more efficient at 32°C, since its expression is similar at both temperatures (Fig 5A). Interestingly, we observed higher binding affinity between HNRNPH1 and RBM3 mRNA (Fig 5B) in cooled cells, which could explain its higher activity, and may be explained by either or both of the following mechanisms. First, HNRNPH1 interacts with G-tracts and rG4s to promote exon skipping, as reported for HNRNPH1-mediated exon exclusion in RNA-binding protein EWS (EWSR1) transcripts (Neckles *et al*, 2019; Vo *et al*, 2022). The potential formation of rG4s at the HNRNPH1 binding sites (Fig 5C) suggests that temperature-regulated rG4 stability may account for HNRNPH1-mediated RBM3 PE skipping. Alternatively, post-translational modification (PTM) and/or temperature-driven condensation of HNRNPH1 (Kim & Kwon, 2021) might modify its affinity for targeted RNA regions, which would be interesting to explore. Temperature variation can result in markedly different PTM patterns shaped by temperature-sensitive PTM enzymes (Cai *et al*, 2018), including kinases

 © 2023 The Authors

(Haltenhof *et al*, 2020) and arginine methyltransferases (Hong *et al*, 2010). In fact, *in vitro* and *in vivo* evidence supports temperature-dependent changes in RNA-RBP condensation (Molliex *et al*, 2015; Riback *et al*, 2017; Iserman *et al*, 2020; Pullara *et al*, 2022) as a result of differential intermolecular interactions (Tauber *et al*, 2020), possibly linked to distinct PTM of the embedded RBPs (Hofweber & Dormann, 2019; Sridharan *et al*, 2022).

Interestingly, HNRNPH1 missense mutations in the nuclear localisation sequence and nonsense mutations leading to reduced protein levels have been identified in individuals with neurodevelopmental disorders and intellectual impairment (Reichert *et al*, 2020; Gillentine *et al*, 2021). A possibility is that these mutations may act, in part, through impaired HNRNPH1 induction of RBM3 expression, affecting its roles in neurogenesis (Zhu *et al*, 2019) and/or synaptic maintenance (Peretti *et al*, 2015, 2021). In addition, HNRNPH1 sequestration in RNA foci has been implicated in neurodegenerative disease models of frontotemporal dementia, associated with impaired splicing functions (Bampton *et al*, 2020). It would be interesting to explore the effect of these HNRNPH1 mutations, and its sequestration in RNA foci, on RBM3 splicing and hence RBM3 protein levels.

The fate of inclusion or exclusion of alternative exons is a consequence of the interplay between the recruited splicing factors and the cis-acting elements with varying affinities, opening up opportunities for targeting different steps during mRNA splicing for therapeutic intervention (El Marabti & Abdel-Wahab, 2021). While small molecules affecting global splicing activity have been applied to cancer treatments (Agrawal *et al*, 2018), modulating specific splicing events by targeting cis-acting elements using splice-switching antisense oligonucleotides (ASOs) show therapeutic benefit in several genetic diseases, including spinal muscular atrophy and Duchenne muscular dystrophy (Havens & Hastings, 2016). Most recently, ASOs designed to repress RBM3 PE inclusion successfully upregulated RBM3 expression, without cooling, leading to marked neuroprotection in mice with prion neurodegeneration (Preußner *et al*, 2023), consistent with the protective effects of inducing RBM3 by hypothermia (Peretti *et al*, 2015) or other means (Peretti *et al*, 2021). Beyond ASO technology, the past two decades have witnessed the development of splicing control using bifunctional oligonucleotides, consisting of an antisense domain complementary to the mRNA region close to the splice site, and a tail domain recruiting RBPs to promote exon inclusion or exclusion as an alternative approach (Skordis *et al*, 2003; Zhou, 2022). The identification of HNRNPH1 in this study as an RBM3 PE repressor, among other splicing factors, brings the potential to apply such technology to novel targets for RBM3 therapeutic induction in clinical scenarios ranging from acute brain injury to neurodegeneration.

Materials and Methods

Human iPSC culture

NGN2-OPTi-OX was generated by the Kotter laboratory at the University of Cambridge (Pawlowski *et al*, 2017), where the original iPSC line was sourced from the University of Cambridge (https://hpscreg.eu/cell-line/CAMi014-A). iPSCs with Neurogenin-2 (NGN2) transgene stably integrated into a "safe-harbour" locus under doxycycline (Dox)-inducible promoter (Pavlou *et al*, 2023) were maintained under feeder-free conditions in TeSR-E8 medium in a 37°C, 5% CO_2 tissue culture incubator. They were cultured on vitronectin (3.3 μg/ml)-coated culture plates or glass-bottom dishes and fed every day with TeSR-E8 medium or every 2 days with StemFlex Medium. 0.5 mM EDTA was used for routine dissociation to maintain colony growth. iPSCs were frozen in Cryostor Cs10 Cryopreservation.

iPSCs differentiation into iPSC-derived neurons (i-neurons)

iPSCs were enzymatically detached and dissociated into single cells using Accutase and plated into GelTrex (1:100 dilution)-coated culture plates in TeSR-E8 medium supplemented with 10 μM Rho-associated protein kinase (ROCK) inhibitor. After 24 h (Day 1), TeSR-E8 medium was changed to DMEM/F12 medium with GlutaMAX, supplemented with 1x N-2 supplement, 1x Non-Essential Amino Acids, 50 nM 2-Mercaptoethanol, 100 U/ml Penicillin–Streptomycin, and 1 μg/ml Doxycycline Hyclate (Dox) (iN-1 medium). After 24 h (Day 2), the medium was replaced with the same medium as the previous day. From Day 3 to Day 6, the culture was fed daily with Neurobasal medium supplemented with 1x B-27 supplement (minus vitamin A), 1x GlutaMAX, 50 nM 2-Mercaptoethanol, 100 U/ml Penicillin–Streptomycin, and 1 μg/ml Dox, 10 ng/ml Neurotrophin-3 (NT-3), and 10 ng/ml Brain-derived neurotrophic factor (BDNF) (iN-2 medium). After Day 6, the medium was changed every other day. Cultures used for flow cytometry were prepared by dissociating Day 4 iPSCs with Accutase and plating 80,000 cells per well in a 96-well culture plate precoated with GelTrex (1:100 dilution). The same feeding schedule as previously described was followed from Day 5. To prepare cultures for live fluorescent imaging, Day 4 iPSCs were dissociated with Accutase and plated 10,000–20,000 cells/dish on 35 mm MatTek glass-bottom dishes precoated with 0.1 mg/ml poly-L-lysine and 10 μg/ml laminin in iN-2 medium with ROCK inhibitor. The same feeding schedule as previously described was followed from Day 5. I-neurons in the 32°C conditions were placed in a 5% CO_2 incubator set to 32°C for 72 h between Day 15–18 post-differentiation. 1 μM actinomycin D (ActD) and 50 μM cycloheximide (CHX) were incubated for 72 h, and SMG1 inhibitor for 24 h before collection.

HeLa and HEK293T cell cultures and transfection

HeLa and HEK 293 T cells were grown in Dulbecco's modified Eagle's medium (DMEM) + Ham's F12 (1:1) supplemented with 10% fetal calf serum (FCS), penicillin (100 IU/ml), and streptomycin (100 μg/ml) and grown at 37°C and 5% CO_2. Plasmid DNA transfection was performed with Lipofectamine 2000 (Thermo Fisher Scientific) or TransIT-LT1 (Mirus) according to the manufacturer's instructions.

For HNRNPH1 siRNA transfections, 250,000 HeLa cells were seeded in a six-well plate and the next day cells were transfected with two siRNAs 10 nM siRNA each (siRNA#1 + siRNA#2) using Lipofectamine 2000. After 24 h, a second round of siRNA transfection was done in fresh medium with or without RBM3 minigene cotransfection. One set of the cells was moved to 32°C while the other set was kept at 37°C. 24 h later, medium was changed again with fresh medium with or without SMG1 inhibitor (1 μM). SMG1

© 2023 The Authors

inhibitor (1 μM) and cycloheximide (200 μg/ml) were incubated for 24 h before harvesting the cells.

Plasmids, oligonucleotides and guide RNAs

All primers used in this study are listed in Appendix Table S1, and guide RNAs (gRNAs) and siRNAs in Appendix Table S7.

GFP-RBM3 repair template plasmid: To prepare iPSC cDNA, RNA was purified from iPSC pellets using the RNeasy Mini kit and reverse transcribed into cDNA using the oligo(dT) primer and the SuperScript IV First-Strand Synthesis System. The following fragments were amplified by PCR using Q5 DNA Polymerase and the indicated templates and primers (Appendix Table S1): (i) The 5′ homology arm (1 kb sequence immediately upstream of RBM3 start codon) was amplified from iPSC cDNA using Primers Pr1 and Pr2; (ii)The GFP fragment was amplified from pcDNA3-EGFP using Primers Pr3 and Pr4; (iii) The 3′ homology arm (start codon and 1 kb sequence at it immediate downstream) was amplified from iPSC cDNA using Primers Pr5 and Pr6; (iv) the origin of replication and ampicillin-resistant gene were amplified using Primers Pr7 and Pr8. The repair template plasmid for CRISPR knocked in of GFP to the N-terminus of the RBM3 coding region (GFP-RBM3 repair template) was generated by assembling Fragments a-b-c-d in the indicated order using NEBuilder. Plasmids were validated by Sanger sequencing. Single-stranded DNA (ssDNA) were subsequently synthesised from these repair template plasmids to improve CRISPR knock-in efficiency using Guide-it Long ssDNA Production System with Primers Pr9 and Pr10 according to manufacturer instructions. The 19-nucleotide (nt) guide RNA (gRNA) RBM3-N gRNA#1 (5′-CUGCCAUGUCCUCUGAAGA-3′) and #2 (5′-UUUCCUU CUUCAGAGGACA-3′) followed by the protospacer adjacent motif (PAM) targeting the region adjacent to the RBM3 start codon were resuspended in water to the concentration of 4 μg/μl.

RBM3 minigene expression plasmids: RBM3 minigene was cloned by amplifying genomic regions from exon 1–4 using forward primer – AAGAATTCATGTCCTCTGAAGAAGGAAAGC and reverse primer – TTTGCGGCCGCCTCTAGAGTAGCTGCGACCACGCC and then inserting in EcoRI-NotI sites in pcDNA3.1(+) vector backbone. Minigene Del GGGG mutant was generated using site-directed mutagenesis (QuickChange Lightning Multi Site-Directed Mutagenesis Kit) using the primer- GTCGGGCAGCTTAGAGGAGTAGGAGAACT CAG.

FLAG-HNRNPH1 plasmid: FLAG-HNRNPH1 expression construct was generated by inserting HNRNPH1 coding sequence, obtained as a string synthesised (GeneArt, Thermo Fisher Scientific), in XbaI-NotI sites in pcDNA6F vector backbone.

hPGK:BFP lentiviral plasmid: The BFP-expressing control plasmid is ordered from VectorBuilder according to a custom design. Briefly, a TagBFP2 (VectorBuilder) is cloned into a lentiviral gene expression vector pLV (VectorBuilder) containing a human PGK promoter.

hPGK:HNRNPH1-T2A-BFP lentiviral plasmid: All fragments were amplified using Q5 Hotstart master mix from New England Biolabs. The vector was amplified from pLV-hPGK:BFP in two halves using primers Pr15 and Pr16 giving a 3,329 bp product and primers Pr17 and Pr18 giving a 3,383 bp product. The HNRNPH1 insert was amplified with primers Pr19 and Pr20 from pcDNA3-HNRNPH plasmid, giving a product of 1,400 bp. T2A-TagBFP insert was amplified using primers Pr21 and Pr22 from a previously generated pLV-EIF1A:RBM3-T2A-BFP plasmid, yielding a product of 790 bp. All fragments were gel purified using a Qiagen kit following the manufacturer's guidelines. The final vector was assembled using the Klenow Assembly Method. Sequences within the promoter and insert regions were confirmed by Sanger sequencing.

Generation of GFP-RBM3 iPSCs by CRISPR

Half a million wild-type iPSCs were electroporated with Cas9 protein, RBM3 gRNAs and GFP-RBM3 repair template using Lonza Nucleofector Technology according to the manufacturer's instructions. Briefly, Cas9-RNP complex mixture containing 0.2 μl 3 M NaCl, 1 μl RBM3-N gRNA#1, 1 μl RBM3-N gRNA#2, 1 μl Cas9 protein (HiFi Cas9 nuclease V3, 10 μg/μl) were assembled and incubated at room temperature for 45 min. 0.5 million iPSCs were resuspended with the nucleofector solution (90 μl P3 solution and 20 μl supplement). 3 μl of the repair template ssDNA (4 μg/μl) was added to the pre-assembled Cas9-RNP, mixed with the iPSC suspension, and transferred to the Nucleocuvette Vessels. Placed the vessel into the nucleofector unit and started the programme to complete electroporation. Immediately after, the electroporated cells were gently transferred to the 4 ml prewarmed StemFlex medium supplemented with 10 μM ROCK inhibitor and 40 μl homology-directed repair (HDR) enhancer and plated into 2 vitronectin-coated wells in a six-well plate. The cells were then incubated at 32°C for 48 h. The following day after electroporation, the medium was replaced with StemFlex medium and replaced every other day. When reaching 70–80% confluency, cells in one well were frozen in Cryostor Cs10 Cryopreservation at −80°C and those in the other were detached and dissociated with Accutase.

Isolated iPSCs were plated in a vitronectin-coated 96-well plate in StemFlex medium supplemented with 10 μM ROCK inhibitor, with 30–50 cells per well. From the next day, the iPSCs were fed with StemFlex medium every other day until confluent. Confluent wells were dissociated and half of the cells were subject to flow cytometry measurement (see Flow cytometry) to determine the GFP intensity of iPSCs in each well. 500–750 cells from each of the wells with GFP-positive cells were plated in a vitronectin-coated 10-cm Petri dish in StemFlex medium supplemented with 10 μM ROCK inhibitor. From the next day, the hiPSCs were fed with StemFlex medium every other day until colonies were formed (1–2 weeks). Imaged with a wild-field fluorescent microscope, GFP-positive colonies were picked by gentle aspirating with P1000 pipette tips and transferred to a well in round-bottom 96-well plates with 200 μl StemFlex medium with 10 μM ROCK inhibitor and 100 U/ml Penicillin–Streptomycin per well. Once finished colony picking, each well was split into two vitronectin-coated flat-bottom 96-well plates by gentle pipetting to break colonies into small clusters and transferring 100 μl cell suspension in each well into a well in new plates. Each well was fed StemFlex medium every other day until the majority of the wells reached 50–60% confluency. Genomic DNA of individual colonies in one of the duplicated plates was extracted (see Genomic DNA extraction) and PCR-genotyped using GoTaq Taq G2 Green Master Mix and primer pairs Pr11/12, Pr11/14, and Pr12/13. The correctly genotyped clones in the corresponding second plates were expanded in six-well plates to be further validated by Sanger sequencing. Sequence-verified clones were aliquoted and frozen at −80°C.

 © 2023 The Authors

Genomic DNA extraction

iPSCs were detached using Accutase and pelleted by centrifugation at 250 *g* for 5 min. After removing the supernatant, 50 μl (each well of a 96-well plate) or 5–10 μl (every 1,000 iPSCs) lysis buffer (and 1:40 freshly added Proteinase K) was added to the pellet and lysed at 55°C overnight in a shaker. The next day, 10% volume of 3 M sodium acetate (pH 5.2) and an equal volume of isopropanol were added to the lysate and mixed by brief vortexing. Genomic DNA was pelleted by 15-min centrifugation at maximum speed, followed by two washes with 200–1,000 μl of 80% ethanol. After the last centrifugation to remove the residual ethanol, pellets were left at room temperature to air dry, and resuspended in 50–500 μl TE buffer (10 mM Tris–HCl, pH 8.0 and 0.1 mM EDTA).

Western blotting

Protein concentrations of lysates were determined by BCA assay following the manufacturer's instruction. Samples were diluted with Laemmli protein sample buffer with 100 mM DTT. 10–15 μg protein was loaded into each well in precast 12% gels or 4–15% gradient gels and ran at 125 V. Proteins were transferred to a 0.2 μm nitrocellulose membrane at 70 V for 2 h in a wet blot system. Membranes were blocked with 5% BSA or 5% non-fat milk in 1x TBS-T for 1 h rotating at room temperature. The primary antibody solution was incubated overnight at 4°C while rotating. The next day, membranes were washed three times with 1x TBS-T, then incubated for 1 h in secondary antibody solution (1:10,000 in 5% BSA or 5% non-fat milk in 1x TBS-T) and washed three times with 1x TBS-T before adding HRP chemiluminescent substrate to develop on a ChemiDox MP Imaging System or directly on LI-COR Odyssey CLx Primary and secondary antibodies were used in the following concentrations: rabbit anti-RBM3 (1:1000), mouse anti-Cas9 (1:1,000), mouse anti-GAPDH (1:2,000), mouse anti-Cas9 (1:2,000), rabbit anti-HNRNPH1 (1:1,000, Abcam), rabbit anti-HNRNPH1 (1:1,000, ProteinTech, Fig EV4F), mouse anti-FLAG (1:2,000), mouse anti-HNRNPH/F (1:1,000 Santa Cruz), rabbit anti-SmD3 (1:1,000), donkey anti-rabbit 680LT (1:10,000), donkey anti-mouse 680LT (1:10,000), donkey anti-rabbit 800CW (1:10,000), donkey anti-mouse 800CW (1:10,000), goat anti-rabbit and goat anti-mouse HRP conjugated secondary antibodies (1:10,000, Biorad).

Lentiviral production

96-well plates or T75 flasks precoated with 25 μg/ml PLL at 37°C overnight, followed by three times washes with distilled water and left to dry in tissue culture hoods. HEK293FT were plated at 10^6 cells/cm^2 density and incubated at 37°C overnight. Lentiviral expression and packaging plasmids were transfected using Lipofectamine LTX. For each well of a 96-well plate (upscale proportionally for T75 flask transfection), Mix A: 20 μl Opti-MEM, 19.12 ng psPax2, 12.5 ng pMD2.G, 25 ng expression plasmid, 0.1 μl Plus Reagent; Mix B: 5 μl Opti-MEM, 0.3 μl Lipofectamine LTX were assembled and incubated at room temperature for 20 min before adding to the HEK293FT cultures. After 24 h, media were replaced with fresh media and checked for BFP expression. Viruses were harvested 72 h post-transfection by centrifuging at 6,000 *g* at 4°C overnight. The pellets were resuspended with PBS and frozen at −80°C for storage.

Flow cytometry

Dissociated cells were resuspended in culture media in 96-well plates and fluorescent intensities of GFP, mCherry and/or BFP were measured at room temperature with a CytoFLEX S System (Beckman Coulter) and CytExpert (v2.4) Program. Acquired data were analysed with FlowJo (v10.7.2). Briefly, successfully transduced cells were gated sequentially for cells (FSC-A vs SSC-A), singlets (FSC-H vs FSC-A) and BFP (FSC-A vs PB450-A). The background fluorescence was gated with non-transduced WT cells. For editing efficiency measurement, mCherry (ECD-A) intensities were measured within the BFP-positive Cas9 WT or GFP-RBM3 reporter i-neurons and unedited cells were gated with the WT i-neurons expressing the reporter lentivirus. Median GFP (FITC-A) intensities within the gated population were automatically calculated by FlowJo.

GFP-RBM3 i-neuron live imaging

GFP-RBM3 iPSCs and i-neurons cultured at low density on vitronectin (iPSC) or PLL and laminin (i-neuron)-coated 35-mm glass bottom dishes were imaged in TeSR-E8 (iPSC) or phenol red-free iN-2 media (i-neuron, to minimise autofluorescence) in OkoLab temperature-controlled chamber. Cells were imaged at 37 or 32°C with 5% CO_2 on a custom-built wide-field microscope in Epi fluorescence mode with a 100x oil TIRF objective (Olympus). GFP was excited with a 488 nm laser at 5 mW. Hoechst nuclear stain or BFP of lentiviral transduced cells was excited with a 504 nm laser at 10 mW. Both channels were imaged simultaneously and separated with OptoSplit III System (Cairn Research) and a Prime BSI camera (Photometrics).

RBM3 CRISPR knockout screen

Both clones of GFP-RBM3 iPSCs were differentiated in GelTrex-coated T75 flasks. The total number of i-neurons used for each clone in each replicate was approximately 60,000,000 to ensure sufficient copies of individual sgRNAs could be obtained from the pool. 4 days of postdifferentiation, the cultures were transduced with a whole-genome sgRNA lentiviral library (399 non-targeting sgRNAs and 91,138 sgRNAs targeting 18,466 genes across the human genome), co-expressing a BFP fluorescent reporter to indicate successfully transduced cells. The viral titre was determined in pilot experiments, resulting in 20% of the i-neurons becoming BFP-positive on Day 18 to minimise multiple sgRNA entries into a single cell. I-neurons at 14, 15 or 16 days of postdifferentiation (or 11, 12, 13 days of post-transduction) were cooled at 32°C for 72 h before dissociation with 20 min Accutase and 10 min Trypsin–EDTA incubation, followed by fluorescence-activated cell sorting (FACS). The GFP intensity of all BFP-positive cells was manually separated into four quartiles, and only cells with the GFP intensity falling in the top or the bottom quartile were collected in separate tubes and their genomic DNA was extracted (see Genomic DNA extraction) immediately after. Around 3,000,000 i-neurons were collected in the low GFP or high GFP tube for each clone in each replicate.

Purified DNA was sequenced to identify sgRNAs enriched in high or low GFP populations. The sequencing library was created in a two-stage PCR reaction. The first stage amplifies the enriched gRNA

cassettes: 2 μg DNA, 1.5 μl Forward Primer (10 μM), 1.5 μl Reverse Primer (10 μM), 25 μl NEB Q5 High-Fidelity 2x Master Mix in a 50 μl reaction. The PCR condition was: 98C for 30 s, 25 cycles of (98C for 10 s, 62C for 30 s, 72C for 15 s), and 72C for 2 min. The PCR product was purified using Ampure XP Beckman magnetic beads and diluted to 200 pg/μl. The second PCR attached the Illumina adaptors and barcodes: 1 μl PCR product from the first reaction, 0.75 μl Forwards Primer (20 uM), 0.75 μl Reverse Primer (20 uM), 10 μl Roche 2X KAPA HiFi ReadyMix in a 20 μl reaction. The PCR condition was: 95C for 3 min, 9 cycles of (98C for 20 s, 66C for 15 s, 72C for 20 s), and 72C for 1 min. PCR products were purified using Ampure XP Beckman magnetic beads and eluted in 35 μl TE buffer. PCR products were quantified with NEBNext Library Quant Kit for Illumina. The library was run on an Illumina NextSeq 550, using an Illumina 75 cycle, high output kit at 1.4 pM.

Arrayed CRISPR target validation

Inserts containing the candidate gene targeting sgRNA (1–3 sgRNAs per gene) sequences and non-targeting sgRNA sequences (see Appendix Table S4) were cloned into a lentiviral expression vector pLVPB-U6-sgRNAv2fl_shortccdB_PGK_Puro_BFP linearised with BbsI, which removed the suicide ccdB cassette. The sgRNA expression plasmids were verified by Sanger sequencing and used to generate lentiviral particles (see Lentiviral production) in a 96-well array format. GFP-RBM3 Clone 1 iPSCs at 4 days after differentiation in 96-well plates were transduced with the arrayed lentiviral library at a viral concentration predetermined in pilot experiments to obtain maximal transduction efficiency. 15 days of postdifferentiation, GFP-RBM3 i-neurons were either cooled at 32°C for 72 h or continue to grow at 37°C. On day 18, BFP and GFP intensities of the transduced cultures were measured using flow cytometry (see Flow cytometry).

RNA-seq

Four biological replicates of WT i-neurons in control (37°C) and cooled (72 h at 32°C, Days 15–18) were included in this study. Total RNA was extracted with RNeasy Plus Mini Kit. RNA concentrations of individual samples were measured using Qubit RNA Broad Range Assay Kit and their integrity was determined using Agilent TapeStation System. The library was prepared using Illumina TruSeq Stranded mRNA Library Prep following the manufacturer's instructions and sequenced paired-end 150 bp on Novaseq.

HNRNPH1 RNA immunoprecipitation

HeLa cells were seeded at 30% confluency in two T75 flasks. 24 h of postseeding, one flask was moved to a 32°C incubator and the other one was maintained at 37°C for the next 48 h until harvesting. Cells were treated with SMG1 inhibitor (1 μM) 24 h before harvesting. Cells were harvested by scraping and washed with 1x Phosphate buffered saline (PBS). To prepare cell extracts, the cell pellets were dissolved in 800 μl Gentle Hypotonic Lysis Buffer (10 mM Tris pH 7.2, 10 mM NaCl, 2 mM EDTA, 0.5% Triton-X-100) supplemented with 1x HALT protease and phosphatase inhibitor cocktail (Thermo Scientific, 78442), 0.06 U/μl DNase1 (Zymo, E1011-A) and 0.5 U/μl RiboLock RNase inhibitor (Thermo Scientific, EO0381) and incubated on ice for 10 min. After adjusting the NaCl concentration to 150 mM, the extracts were incubated for another 5 min on ice and then cleared by centrifugation at 15,000 *g* and 4°C for 15 min. 50 μl Protein G Dynabeads (Life Technologies, 100090) per Immunoprecipitation were washed two times with TBS supplemented with 0.05% NP-40 (IGEPAL CA-630) and incubated in 600 μl total volume of TBS-0.05% NP-40 with 5 μg of rabbit anti-HNRNPH1 (Abcam, ab13074) or rabbit IgG (Santa Cruz, sc2027) head-over-tail for 2 h at 4°C. The beads were subsequently washed three times with 1 ml TBS-0.05% NP-40 (supplemented with RNase and protease inhibitors). To keep protein input fractions, 30 μl of the cleared extracts were boiled with 2x LDS loading buffer. For RNA input fractions, 50 μl were transferred to 1 ml TRIzol (Invitrogen, 15596018) supplemented with 0.14 M β-mercaptoethanol (Applichem, A1108). 700 μl of the extracts were then distributed to Eppendorf tubes containing antibody-bound beads and incubated on a rotary wheel for 2 h at 4°C. After washing the beads five times with 1 ml TBS-0.05% (NP-40), 10% of the beads were transferred into a separate Eppendorf tube and boiled in 2× LDS loading buffer for 5 min at 95°C, and 1 ml TRIzol supplemented with 0.14 M β-mercaptoethanol was added to the remaining beads. Proteins were analysed using 4–12% Bis-Tris polyacrylamide gels followed by western blot using the following antibodies: Mouse anti-HNRNPH/F (Santa Cruz, sc-32310, 1:1,000). RNA was isolated from TRIzol according to the manufacturer's protocol and then reverse transcribed and analysed by RT-qPCR. The fold change of HNRNPH1-pulled down RBM3 mRNA on cooling is normalised to the change of total RBM3 mRNA and HNRNPH1 immunoprecipitation efficiency (the ratio between HNRNPH1 pulled down and total HNRNPH1) at 37°C vs. 32°C of individual replicates.

RT-PCR and qRT-PCR

RNA of i-neurons, HeLa and HEK293T cells was extracted using the RNeasy Plus Mini kit or Absolute RNA miniprep kit and reverse transcribed into cDNA using random hexamers and the SuperScript IV First-Strand Synthesis System or AffinityScript Multiple Temperature Reverse Transcriptase. When using the latter method, 1–2 μg of isolated RNA was incubated with random hexamers (900 ng) in a total volume of 67 μl in DEPC water at 65°C for 5 min and then for 10 min at room temperature. Then, the mixtures were separated into 2 parts – RT and no-RT control samples (33.5 μl each). To this 13.5 μl master mix was added to supplement 1x reverse transcriptase buffer (Agilent), 10 mM DTT (Agilent), 400 μM dNTPs (Thermo Scientific), 40 units RiboLock RNase inhibitor (Thermo Scientific) and 1 reaction equivalent AffinityScript Multiple Temperature Reverse Transcriptase (Agilent). The RT enzyme was absent in the no-RT condition. The samples were then incubated at 65°C for 1 h followed by heat-inactivation at 75°C for 20 min.

For RT-PCR, 20–40 ng cDNA was amplified using GoTaq Polymerase mix using the indicated primers (Appendix Table S1). Quantification of the bands on agarose gels was done using FIJI software. Percent spliced in (PSI) indexes of RBM3 poison exon (PE) are calculated based on the intensity of PE-included (red arrows) and PE-skipped (green arrows) isoforms visualised in agarose gels:

i One PE-included isoform is detected: (I_{Inc}/L_{Inc}) / $(I_{Inc}/L_{Inc} + I_{skip}/L_{skip})$, or
ii Two PE-included isoforms are detected: $(I_{Inc1}/L_{Inc1} + I_{Inc2}/L_{Inc2})$ / $(I_{Inc1}/L_{Inc1} + I_{Inc2}/L_{Inc2} + I_{skip}/L_{skip})$

 © 2023 The Authors

Where I_{Inc} = Intensity of PE-included isoform, L_{Inc} = length of PE-included isoform in base pairs, I_{Inc} = Intensity of PE-skipped isoform, L_{skip} = length of PE-skipped isoform in base pairs.

For qRT-PCR, cDNA of each sample (diluted 10–1,000 times) was mixed with SYBR Green PCR Master Mix and PCR primers in 4 technical replicates in 384-well plates. Reactions were performed in a QuantStudio Real-Time PCR system (ThermoFisher Scientific). Analysis was performed with Design and Analysis Software (v2.6.0, ThermoFisher Scientific). Relative PE inclusion is the ratio of the relative expression levels between RBM3 Exon 3a amplicon and RBM3 Exon 3 or Exon 4–5 amplicon (or their geometric mean values). 18 s rRNA served as a loading control when quantifying RBM3 and HNRNPH1 mRNA levels.

Immunocytochemistry and wild-field imaging

I-neurons cultured at low density on PLL and laminin-coated 35-mm glass bottom dishes were washed once with PBS before fixed with fixation buffer (4% PFA, 4% sucrose in PBS). Cell membrane was permeabilized with 0.5% Triton in PBS for 15 min and incubated in blocking solution (5% donkey serum in washing buffer) for 30 min at room temperature. After washing once with PBS, samples were incubated overnight with primary antibodies against HNRNPH1 (1: 500, ab10374, Abcam) at 4°C. On the next day, cell cultures were washed 3 times with PBS and incubated with secondary antibodies (1:1,000, A11011, Alexa Fluor™ 568, Invitrogen) for 1 h at room temperature. The neurons were washed three times and Hoechst (20 mM, 1:1,000) was added for 10 min before washing three more times and kept in PBS for imaging.

Images were acquired on a Ti2-E High Content Microscope (Nikon) with a 60x oil objective (Nikon). A bright-field, a 560 nm (HNRNPH1) and a 405 nm (Hoechst) images were taken sequentially with pre-optimised imaging settings, which remained constant through the imaging session.

SmB immunoprecipitation

For SmB immunoprecipitation, HeLa cells were seeded at 30% confluency in two T75 flasks. 24-h postseeding, one flask was moved to a 32°C incubator and the other one was maintained at 37°C for the next 72 h until harvesting. Cells were harvested by scraping and washed with 1x Phosphate buffered saline (PBS). Lysis was done in 600 μl RIPA lysis buffer supplemented with 1x Halt Protease Inhibitor cocktail (Thermo Scientific, 1861278), followed by sonication at 40% amplitude on ice. The extracts were then cleared by centrifugation at 15,000 *g* and 4°C for 15 min. For input fractions, 60 μl of the cleared extracts ($1/10^{th}$) were boiled with 2x LDS loading buffer. 80 μl Protein G Dynabeads (Life Technologies, 100090) per immunoprecipitation were washed twice with TBS supplemented with 0.05% NP-40 (IGEPAL CA-630) and incubated in 600 ul total volume of TBS-0.05% NP-40 with 10 μg of mouse anti-SmB or mouse IgG (Santa Cruz, sc2025) head over tail for 1.5 h at 4°C. The beads were then washed twice times with 1 ml TBS-0.05% NP-40 and were then resuspended in 270 μl of lysate and incubated on a rotary wheel for 2 h at 4°C. Subsequently, the beads were washed three times with RIPA lysis buffer and once with TBS-0.05% NP-40. With the final wash, the beads were transferred to new tubes, wash buffer was removed, and the beads were resuspended in 70 μl of 2x LDS-loading buffer, boiled for 5 min at 95°C and loaded on a 4–12% Bis-Tris polyacrylamide gels followed by western blotting. For SmB immunoprecipitation in I-neurons, the same protocol was followed as HeLa except that the lysis was done in 700 μl RIPA lysis buffer, and the input fractions and lysates for incubation with the beads were 70 and 300 μl, respectively.

Data analysis

Image quantification of live or fixed iPSCs and i-neurons

Regions with single cells were cropped from the full-size images with a custom-made program. Then, each single-cell image was analysed with a custom-made MatLab pipeline.

- i For Fig 1A, nuclei were defined by Hoechst signals excited at 405 nm, and cell boundaries were defined by GFP-RBM3 signals acquired with 488 nm excitation.
- ii For Fig EV2A, lentiviral BFP expression indicated successfully transduced cells and the BFP fluorescence was used to define soma boundary. Nuclei were defined as areas where GFP was expressed at higher intensities, as RBM3 is predominantly nuclear. This was achieved by applying automatic global Otsu's threshold to the GFP channel after background removal.
- iii For Fig EV5C, nuclei were defined by Hoechst signals excited at 405 nm, and cell boundaries were defined by HNRNPH1 signals acquired with 560 nm excitation.

Cytoplasm was defined as the area outside of the nucleus, within the soma. After segmentation, average intensities (total intensity divided by unit area) of GFP-RBM3 or HNRNPH1 (Alexa 568) fluorescence in each of three cellular compartments, soma, cytoplasm and nuclei, were calculated.

Whole-genome CRISPR screen next-generation sequencing analysis

21 nt long sequencing reads were exported from bcl files using bcl2fastq v2.2.0. These reads were counted by converting them to k-mers and mapping them to a set of 91,536 valid CRISPR library 20 nt gRNA sequences. Reads without an exact match in the library were discarded. After merging the sample counts into a count table, the samples were inspected for proper gRNA infection and coverage. With all the samples passing QC, the MAGeCK RRA (Li *et al*, 2014) was used to perform gene essentiality and enrichment inference. To run MAGeCK, we used the paired samples option, which contained the sorted samples: low GFP sample in control group and high GFP samples in the treatment group and a set of nontargeting gRNAs as controls. Functional and network analyses of top RBM3 regulator candidates with FDR <0.05 were performed with Metascape (Zhou *et al*, 2019) and STRING (Szklarczyk *et al*, 2020).

RNA-seq analysis

Fastq files of sequencing reads were processed directly with the nf-core (Ewels *et al*, 2020) rnaseq pipeline (v3.3) (Patel *et al*, 2021), mapped to hg38 genome with the gencode v38 annotation (GRCh38.p13). A gene expression quantification table was generated from the salmon output gene counts using the star_salmon aligner option within the nf-core/rnaseq pipeline. Differential expression analysis was performed using DESeq2 (v3.15). Grouped sashimi plots to visualise alternative splicing of RBM3 mRNA between control and cooled i-neurons were generated using rmats2-sashimiplot (v2.0.4) and BAM files.

© 2023 The Authors

ENCODE project data analysis

RBM3 TPM values in RNA-Seq experiments following targeted CRISPR editing in HepG2 and K562 cell lines, together with the matching control samples, were extracted from the publicly available HNRNPH1 (ENCFF039DFP, ENCFF713MXN, ENCFF616BYI, ENCFF586TGE, ENCFF293ODK, ENCFF266YWO, ENCFF053QJC, ENCFF200BWY), SNRNP70 (ENCFF367WUN, ENCFF058OGQ, ENCFF873KLR, ENCFF311ACC), PUF60 (ENCFF682SFK, ENCFF461EMF, ENCFF585BAL, ENCFF643OYR) and KPNB1 (ENCFF819AUU, ENCFF628VDB, ENCFF565TSG, ENCFF073IFA)-knockdown datasets of the ENCODE Project.

To perform splicing analysis on RBM3 mRNA in control and HNRNPH1 knocked-down HepG2 and K562 cells. Raw Fastq files were downloaded from the European Nucleotide Archive (ENA). Fastq files were processed through FastQC (v0.11.8) for quality control. The data quality of each Fastq file was reviewed manually. Reads were mapped to GRCh38.p13 human reference genome by STAR (v2.6.1b). Alignment results were sorted by the coordinate. Output BAM files were indexed by SAMtools (v1.9).

Splicing analysis was performed using rMATS (v4.1.2). Control and knockdown samples were grouped separately to serve as inputs of rMATS. The gene annotation file (GENECODE Human Release 34, GRCh38.p13) was used for analysis. Inclusion levels and FDR values of RBM3 Exon 3a relative to Exon 3 and 4 in the skipped exon output tables (SE.MATS.JCEC) were used to generate bar graphs indicating RBM3 Exon 3a inclusion levels (see Appendix Table S6 for corresponding entries). Grouped sashimi plots to visualise alternative splicing of RBM3 Exon 3 and 4 between control and HNRNPH1 knocked-down samples were generated using rmats2sashimiplot (v2.0.4) and BAM files.

HNRNPH1 public iCLIP analysis

Sequence fastq files from public HNRNPH1 iCLIP datasets from HEK293T cells were accessed from ArrayExpress with accession numbers ERR2201859 & ERR2201860 (Braun *et al*, 2018). Samples were processed with the nf-core/clipseq pipeline v1.0.0 (Ewels *et al*, 2020), mapped to hg38 genome with the gencode v38 annotation to obtain crosslink counts. Crosslink signal was visualised with library size normalisation and rollmean smoothing (window 5) with clipplotr (preprint: Chakrabarti *et al*, 2021) between exons 3 and 4 of RBM3 (chrX:48575560-48576419:+). RNA G4 prediction was performed with QGRS Mapper using default settings (Kikin *et al*, 2006).

Data availability

The CRISPR knockout screen data were deposited to GEO (GSE217789; http://www.ncbi.nlm.nih.gov/geo/query/acc.cgi?acc=GSE217789). The i-neuron RNA-seq data were deposited to ArrayExpress (E-MTAB-12402; http://www.ebi.ac.uk/arrayexpress/experiments/E-MTAB-12402/). Protocols and materials are available upon request.

Expanded View for this article is available online.

Acknowledgements

The authors thank Sandeep Rajan and Stefanie Foskolou (University of Cambridge) for technical assistance, Nina M. Rzechorzek (University of Cambridge) for insightful discussions, Mark Kotter for NGN2-OPTi-OX iPSC line, Oliver Muehlemann (University of Bern) for sharing the SMG1 inhibitor, Alessio Vagnoni (King's College London) for HEK293T cells, Mark McNally (Medical College of Wisconsin) for HNRNPH1 construct. Cambridge CRUK Core Genomics Facility and CIMR Flow Cytometry Core Facility for services. This work is supported by a Sir Henry Wellcome Postdoctoral Fellowship [215943/Z/19/Z] (to J.Q.L.), an Open Targets grant [OTAR2054] (to S.P., S.F.F., E.M., G.R.M.), the UK Dementia Research Institute ([UK DRI-6005] to M.D.R), and to D.K., S.M., N.P., D.M.D.B., J.L.F., J.U., E.M., G.R.M., which receives its funding from UK DRI Ltd, funded by the UK Medical Research Council (MRC), Alzheimer's Society and Alzheimer's Research UK.

Author contributions

Julie Qiaojin Lin: Conceptualization; investigation; methodology; writing – original draft. **Deepak Khuperkar:** Investigation; methodology; writing – original draft; writing – review and editing. **Sofia Pavlou:** Investigation; methodology; writing – review and editing. **Stanislaw Makarchuk:** Formal analysis; investigation; writing – review and editing. **Nikolaos Patikas:** Formal analysis; writing – review and editing. **Flora CY Lee:** Formal analysis; writing – review and editing. **Julia M Zbiegly:** Investigation; writing – review and editing. **Jianning Kang:** Formal analysis; writing – review and editing. **Sarah F Field:** Investigation. **David MD Bailey:** Investigation. **Joshua L Freeman:** Investigation. **Jernej Ule:** Resources; supervision; methodology; writing – review and editing. **Emmanouil Metzakopian:** Conceptualization; resources; supervision; methodology; writing – review and editing. **Marc-David Ruepp:** Conceptualization; resources; supervision; methodology; writing – review and editing. **Giovanna R Mallucci:** Conceptualization; resources; supervision; methodology; writing – review and editing.

Disclosure and competing interests statement

S.P. is now an AstraZeneca employee. E.M. is an employee and shareholder of bit.bio.

References

Agrawal AA, Yu L, Smith PG, Buonamici S (2018) Targeting splicing abnormalities in cancer. *Curr Opin Genet Dev* 48: 67–74

Arrich J, European Resuscitation Council Hypothermia After Cardiac Arrest Registry Study Group (2007) Clinical application of mild therapeutic hypothermia after cardiac arrest. *Crit Care Med* 35: 1041–1047

Ávila-Gómez P, Vieites-Prado A, Dopico-López A, Bashir S, Fernández-Susavila H, Gubern C, Pérez-Mato M, Correa-Paz C, Iglesias-Rey R, Sobrino T *et al* (2020) Cold stress protein RBM3 responds to hypothermia and is associated with good stroke outcome. *Brain Commun* 2: fcaa078

Bampton A, Gittings LM, Fratta P, Lashley T, Gatt A (2020) The role of hnRNPs in frontotemporal dementia and amyotrophic lateral sclerosis. *Acta Neuropathol* 140: 599–623

Bastide A, Peretti D, Knight JRP, Grosso S, Spriggs RV, Pichon X, Sbarrato T, Roobol A, Roobol J, Vito D *et al* (2017) RTN3 is a novel cold-induced protein and mediates neuroprotective effects of RBM3. *Curr Biol* 27: 638–650

Braun S, Enculescu M, Setty ST, Cortés-López M, de Almeida BP, Sutandy FXR, Schulz L, Busch A, Seiler M, Ebersberger S *et al* (2018) Decoding a cancer-relevant splicing decision in the RON proto-oncogene using high-throughput mutagenesis. *Nat Commun* 9: 3315

Cai W, Hite ZL, Lyu B, Wu Z, Lin Z, Gregorich ZR, Messer AE, McIlwain SJ, Marston SB, Kohmoto T *et al* (2018) Temperature-sensitive sarcomeric

© 2023 The Authors

protein post-translational modifications revealed by top-down proteomics. *J Mol Cell Cardiol* 122: 11 – 22

Caputi M, Zahler AM (2001) Determination of the RNA binding specificity of the heterogeneous nuclear ribonucleoprotein (hnRNP) H/H′/F/2H9 family. *J Biol Chem* 276: 43850 – 43859

Chakrabarti AM, Capitanchik C, Ule J, Luscombe NM (2021) Clipplotr – a comparative visualisation and analysis tool for CLIP data. *bioRxiv* https://doi.org/10.1101/2021.09.10.459763 [PREPRINT]

Chip S, Zelmer A, Ogunshola OO, Felderhoff-Mueser U, Nitsch C, Bührer C, Wellmann S (2011) The RNA-binding protein RBM3 is involved in hypothermia induced neuroprotection. *Neurobiol Dis* 43: 388 – 396

Chou MY, Rooke N, Turck CW, Black DL (1999) hnRNP H is a component of a splicing enhancer complex that activates a c-src alternative exon in neuronal cells. *Mol Cell Biol* 19: 69 – 77

El Marabti E, Abdel-Wahab O (2021) Therapeutic modulation of RNA splicing in malignant and non-malignant disease. *Trends Mol Med* 27: 643 – 659

ENCODE Project Consortium (2012) An integrated encyclopedia of DNA elements in the human genome. *Nature* 489: 57 – 74

Ewels PA, Peltzer A, Fillinger S, Patel H, Alneberg J, Wilm A, Garcia MU, Di Tommaso P, Nahnsen S (2020) The nf-core framework for community-curated bioinformatics pipelines. *Nat Biotechnol* 38: 276 – 278

Gillentine MA, Wang T, Hoekzema K, Rosenfeld J, Liu P, Guo H, Kim CN, De Vries BBA, Vissers LELM, Nordenskjold M *et al* (2021) Rare deleterious mutations of HNRNP genes result in shared neurodevelopmental disorders. *Genome Med* 13: 63

Gotic I, Omidi S, Fleury-Olela F, Molina N, Naef F, Schibler U (2016) Temperature regulates splicing efficiency of the cold-inducible RNA-binding protein gene Cirbp. *Genes Dev* 30: 2005 – 2017

Haltenhof T, Kotte A, De Bortoli F, Schiefer S, Meinke S, Emmerichs A-K, Petermann KK, Timmermann B, Imhof P, Franz A *et al* (2020) A conserved kinase-based body-temperature sensor globally controls alternative splicing and gene expression. *Mol Cell* 78: 57 – 69.e4

Havens MA, Hastings ML (2016) Splice-switching antisense oligonucleotides as therapeutic drugs. *Nucleic Acids Res* 44: 6549 – 6563

He F, Jacobson A (2015) Nonsense-mediated mRNA decay: degradation of defective transcripts is only part of the story. *Annu Rev Genet* 49: 339 – 366

Hofweber M, Dormann D (2019) Friend or foe—post-translational modifications as regulators of phase separation and RNP granule dynamics. *J Biol Chem* 294: 7137 – 7150

Hong S, Song H-R, Lutz K, Kerstetter RA, Michael TP, McClung CR (2010) Type II protein arginine methyltransferase 5 (PRMT5) is required for circadian period determination in Arabidopsis thaliana. *Proc Natl Acad Sci USA* 107: 21211 – 21216

Iserman C, Desroches Altamirano C, Jegers C, Friedrich U, Zarin T, Fritsch AW, Mittasch M, Domingues A, Hersemann L, Jahnel M *et al* (2020) Condensation of Ded1p promotes a translational switch from housekeeping to stress protein production. *Cell* 181: 818 – 831.e19

Jackson TC, Manole MD, Kotermanski SE, Jackson EK, Clark RSB, Kochanek PM (2015) Cold stress protein RBM3 responds to temperature change in an ultra-sensitive manner in young neurons. *Neuroscience* 305: 268 – 278

Jacobs SE, Morley CJ, Inder TE, Stewart MJ, Smith KR, McNamara PJ, Wright IMR, Kirpalani HM, Darlow BA, Doyle LW *et al* (2011) Whole-body hypothermia for term and near-term newborns with hypoxic-ischemic encephalopathy: a randomized controlled trial. *Arch Pediatr Adolesc Med* 165: 692 – 700

Kataoka N, Bachorik JL, Dreyfuss G (1999) Transportin-SR, a nuclear import receptor for SR proteins. *J Cell Biol* 145: 1145 – 1152

Kharel P, Becker G, Tsvetkov V, Ivanov P (2020) Properties and biological impact of RNA G-quadruplexes: from order to turmoil and back. *Nucleic Acids Res* 48: 12534 – 12555

Kikin O, D'Antonio L, Bagga PS (2006) QGRS mapper: a web-based server for predicting G-quadruplexes in nucleotide sequences. *Nucleic Acids Res* 34: W676 – W682

Kim GH, Kwon I (2021) Distinct roles of hnRNPH1 low-complexity domains in splicing and transcription. *Proc Natl Acad Sci USA* 118: e2109668118

Koo JW, Mazei-Robison MS, Chaudhury D, Juarez B, LaPlant Q, Ferguson D, Feng J, Sun H, Scobie KN, Damez-Werno D *et al* (2012) BDNF is a negative modulator of morphine action. *Science* 338: 124 – 128

Langer LM, Bonneau F, Gat Y, Conti E (2021) Cryo-EM reconstructions of inhibitor-bound SMG1 kinase reveal an autoinhibitory state dependent on SMG8. *Elife* 10: e72353

Lareau LF, Inada M, Green RE, Wengrod JC, Brenner SE (2007) Unproductive splicing of SR genes associated with highly conserved and ultraconserved DNA elements. *Nature* 446: 926 – 929

Laustriat D, Gide J, Barrault L, Chautard E, Benoit C, Auboeuf D, Boland A, Battail C, Artiguenave F, Deleuze J-F *et al* (2015) In vitro and In vivo modulation of alternative splicing by the Biguanide metformin. *Mol Ther Nucleic Acids* 4: e262

Lefevre EM, Pisansky MT, Toddes C, Baruffaldi F, Pravetoni M, Tian L, Kono TJY, Rothwell PE (2020) Interruption of continuous opioid exposure exacerbates drug-evoked adaptations in the mesolimbic dopamine system. *Neuropsychopharmacology* 45: 1781 – 1792

Li W, Xu H, Xiao T, Cong L, Love MI, Zhang F, Irizarry RA, Liu JS, Brown M, Liu XS (2014) MAGeCK enables robust identification of essential genes from genome-scale CRISPR/Cas9 knockout screens. *Genome Biol* 15: 554

Llorian M, Gooding C, Bellora N, Hallegger M, Buckroyd A, Wang X, Rajgor D, Kayikci M, Feltham J, Ule J *et al* (2016) The alternative splicing program of differentiated smooth muscle cells involves concerted non-productive splicing of post-transcriptional regulators. *Nucleic Acids Res* 44: 8933 – 8950

Luo Y, Hitz BC, Gabdank I, Hilton JA, Kagda MS, Lam B, Myers Z, Sud P, Jou J, Lin K *et al* (2020) New developments on the encyclopedia of DNA elements (ENCODE) data portal. *Nucleic Acids Res* 48: D882 – D889

Molliex A, Temirov J, Lee J, Coughlin M, Kanagaraj AP, Kim HJ, Mittag T, Taylor JP (2015) Phase separation by low complexity domains promotes stress granule assembly and drives pathological fibrillization. *Cell* 163: 123 – 133

Müller-McNicoll M, Rossbach O, Hui J, Medenbach J (2019) Auto-regulatory feedback by RNA-binding proteins. *J Mol Cell Biol* 11: 930 – 939

Natua S, Ashok C, Shukla S (2021) Hypoxia-induced alternative splicing in human diseases: the pledge, the turn, and the prestige. *Cell Mol Life Sci* 78: 2729 – 2747

Neckles C, Boer RE, Aboreden N, Cross AM, Walker RL, Kim B-H, Kim S, Schneekloth JS Jr, Caplen NJ (2019) HNRNPH1-dependent splicing of a fusion oncogene reveals a targetable RNA G-quadruplex interaction. *RNA* 25: 1731 – 1750

Neumann A, Meinke S, Goldammer G, Strauch M, Schubert D, Timmermann B, Heyd F, Preußner M (2020) Alternative splicing coupled mRNA decay shapes the temperature-dependent transcriptome. *EMBO Rep* 21: e51369

Patel H, Ewels P, Peltzer A, Hammarén R, Botvinnik O, Sturm G, Moreno D, Vemuri P, silviamorins, Pantano L *et al* (2021) nf-core/rnaseq: nf-core/rnaseq v3.5 – Copper Chameleon. *Zenodo* https://doi.org/10.5281/zenodo.5789421

Pavlou S, Foskolou S, Patikas N, Field SF, Papachristou EK, Santos CD, Edwards AR, Kishore K, Ansari R, Rajan SS *et al* (2023) CRISPR-Cas9 genetic screen leads to the discovery of L-Moses, a KAT2B inhibitor that attenuates Tunicamycin-mediated neuronal cell death. *Sci Rep* 13: 3934

© 2023 The Authors

Pawlowski M, Ortmann D, Bertero A, Tavares JM, Pedersen RA, Vallier L, Kotter MRN (2017) Inducible and deterministic forward programming of human pluripotent stem cells into neurons, skeletal myocytes, and oligodendrocytes. *Stem Cell Reports* 8: 803 – 812

Peretti D, Bastide A, Radford H, Verity N, Molloy C, Martin MG, Moreno JA, Steinert JR, Smith T, Dinsdale D *et al* (2015) RBM3 mediates structural plasticity and protective effects of cooling in neurodegeneration. *Nature* 518: 236 – 239

Peretti D, Smith HL, Verity N, Humoud I, de Weerd L, Swinden DP, Hayes J, Mallucci GR (2021) TrkB signaling regulates the cold-shock protein RBM3-mediated neuroprotection. *Life Sci Alliance* 4: e202000884

Pomeranz Krummel DA, Oubridge C, Leung AKW, Li J, Nagai K (2009) Crystal structure of human spliceosomal U1 snRNP at 5.5 a resolution. *Nature* 458: 475 – 480

Preußner M, Smith HL, Hughes D, Zhang M, Emmerichs A-K, Scalzitti S, Peretti D, Swinden D, Neumann A, Haltenhof T *et al* (2023) ASO targeting RBM3 temperature-controlled poison exon splicing prevents neurodegeneration in vivo. *EMBO Mol Med* 15: e17157

Pullara P, Alshareedah I, Banerjee PR (2022) Temperature-dependent reentrant phase transition of RNA–polycation mixtures. *Soft Matter* 18: 1342 – 1349

Reichert SC, Li R, Turner S, Jaarsveld RH, Massink MPG, Boogaard MH, Toro M, Rodríguez-Palmero A, Fourcade S, Schlüter A *et al* (2020) *HNRNPH1* -related syndromic intellectual disability: seven additional cases suggestive of a distinct syndromic neurodevelopmental syndrome. *Clin Genet* 98: 91 – 98

Riback JA, Katanski CD, Kear-Scott JL, Pilipenko EV, Rojek AE, Sosnick TR, Drummond DA (2017) Stress-triggered phase separation is an adaptive, evolutionarily tuned response. *Cell* 168: 1028 – 1040.e19

Rzechorzek NM, Connick P, Patani R, Selvaraj BT, Chandran S (2015) Hypothermic preconditioning of human cortical neurons requires proteostatic priming. *EBioMedicine* 2: 528 – 535

Skordis LA, Dunckley MG, Yue B, Eperon IC, Muntoni F (2003) Bifunctional antisense oligonucleotides provide a trans-acting splicing enhancer that stimulates SMN2 gene expression in patient fibroblasts. *Proc Natl Acad Sci USA* 100: 4114 – 4119

Sridharan S, Hernandez-Armendariz A, Kurzawa N, Potel CM, Memon D, Beltrao P, Bantscheff M, Huber W, Cuylen-Haering S, Savitski MM (2022) Systematic discovery of biomolecular condensate-specific protein phosphorylation. *Nat Chem Biol* 18: 1104 – 1114

Sun Y, Bao Y, Han W, Song F, Shen X, Zhao J, Zuo J, Saffen D, Chen W, Wang Z *et al* (2017) Autoregulation of RBM10 and cross-regulation of RBM10/RBM5 via alternative splicing-coupled nonsense-mediated decay. *Nucleic Acids Res* 45: 8524 – 8540

Szklarczyk D, Gable AL, Nastou KC, Lyon D, Kirsch R, Pyysalo S, Doncheva NT, Legeay M, Fang T, Bork P *et al* (2020) The STRING database in 2021: customizable protein–protein networks, and functional characterization of user-uploaded gene/measurement sets. *Nucleic Acids Res* 49: D605 – D612

Tauber D, Tauber G, Parker R (2020) Mechanisms and regulation of RNA condensation in RNP granule formation. *Trends Biochem Sci* 45: 764 – 778

Uren PJ, Bahrami-Samani E, de Araujo PR, Vogel C, Qiao M, Burns SC, Smith AD, Penalva LOF (2016) High-throughput analyses of hnRNP H1 dissects its multi-functional aspect. *RNA Biol* 13: 400 – 411

Vo T, Brownmiller T, Hall K, Jones TL, Choudhari S, Grammatikakis I, Ludwig KR, Caplen NJ (2022) HNRNPH1 destabilizes the G-quadruplex structures formed by G-rich RNA sequences that regulate the alternative splicing of an oncogenic fusion transcript. *Nucleic Acids Res* 50: 6474 – 6496

Wellmann S, Bührer C, Moderegger E, Zelmer A, Kirschner R, Koehne P, Fujita J, Seeger K (2004) Oxygen-regulated expression of the RNA-binding proteins RBM3 and CIRP by a HIF-1-independent mechanism. *J Cell Sci* 117: 1785 – 1794

Wellmann S, Truss M, Bruder E, Tornillo L, Zelmer A, Seeger K, Bührer C (2010) The RNA-binding protein RBM3 is required for cell proliferation and protects against serum deprivation-induced cell death. *Pediatr Res* 67: 35 – 41

Yan J, Goerne T, Zelmer A, Guzman R, Kapfhammer JP, Wellmann S, Zhu X (2019) The RNA-binding protein RBM3 promotes neural stem cell (NSC) proliferation under hypoxia. *Front Cell Dev Biol* 7: 288

Zhou H (2022) Design of Bifunctional Antisense Oligonucleotides for exon inclusion. *Methods Mol Biol* 2434: 53 – 62

Zhou Y, Zhou B, Pache L, Chang M, Khodabakhshi AH, Tanaseichuk O, Benner C, Chanda SK (2019) Metascape provides a biologist-oriented resource for the analysis of systems-level datasets. *Nat Commun* 10: 1523

Zhu X, Yan J, Bregere C, Zelmer A, Goerne T, Kapfhammer JP, Guzman R, Wellmann S (2019) RBM3 promotes neurogenesis in a niche-dependent manner via IMP2-IGF2 signaling pathway after hypoxic-ischemic brain injury. *Nat Commun* 10: 3983

Zimowska G, Shi J, Munguba G, Jackson MR, Alpatov R, Simmons MN, Shi Y, Sugrue SP (2003) Pinin/DRS/memA interacts with SRp75, SRm300 and SRrp130 in corneal epithelial cells. *Invest Ophthalmol Vis Sci* 44: 4715 – 4723

License: This is an open access article under the terms of the Creative Commons Attribution License, which permits use, distribution and reproduction in any medium, provided the original work is properly cited.

© 2023 The Authors

Expanded View Figures

Figure EV1. Characterisation of GFP-RBM3 human iPSC reporter line for CRISPR knockout screen. Related to Fig 1.

A Western blots and quantification of RBM3, Cas9 and GAPDH in two GFP-RBM3 clones and Cas9 WT iPSCs and i-neurons 4, 7 and 18 days after dox-induced differentiation.
B Representative image of GFP-RBM3 iPSCs. The nucleus and soma are outlined by white and yellow dashed lines, respectively. Scale bar: 5 µm.
C Schematic of the reporter lentivirus design and expected fluorescent protein expression in transduced WT (-Cas9) and Cas9 WT (+Cas9) i-neurons. Transduced WT i-neurons (top grey box) show high levels of BFP and mCherry. Transduced Cas9 WT i-neurons (bottom orange box) that are successfully edited by the mCherry sgRNA express reduced levels of mCherry compared to the unedited ones.
D Representative BFP vs. mCherry plots measured by flow cytometry for measuring editing and transduction efficiency in WT, Cas9 WT, two clones of GFP-RBM3 i-neurons 4 days (Day 8) and 14 days (Day 14) after reporter lentivirus transduction. Region (I), (II) and (III) denote BFP+/mCherry-, BFP+/mCherry+ and BFP-/mCherry- populations, respectively.
E Editing and transduction efficiency of WT, Cas9 WT, two clones of GFP-RBM3 i-neurons at day 8 and 18 post differentiation. The calculation is based on the cell numbers within each area labelled in (D) and the formulas are shown in the graph.
F Median GFP intensity of two GFP-RBM3 clones and Cas9 WT i-neurons at 37°C or at 32°C for 24-72 h, measured by flow cytometry.
G Nuclear and cytoplasmic GFP intensity per unit area in GFP-RBM3 i-neurons at 37 or 32°C (72 h). Each data point represents one cell.
H Western blots and quantification of RBM3 normalised to GAPDH in Cas9 WT i-neurons at 37 or 32°C (72 h).

Data information: $N = 3$ biological replicates, except (E), which has $N = 2$. Mean ± SEM; n.s. (not significant), *($P < 0.05$), ***($P < 0.001$); one-way ANOVA with multiple comparisons in (F), unpaired t-tests in (G) and (H).
Source data are available online for this figure.

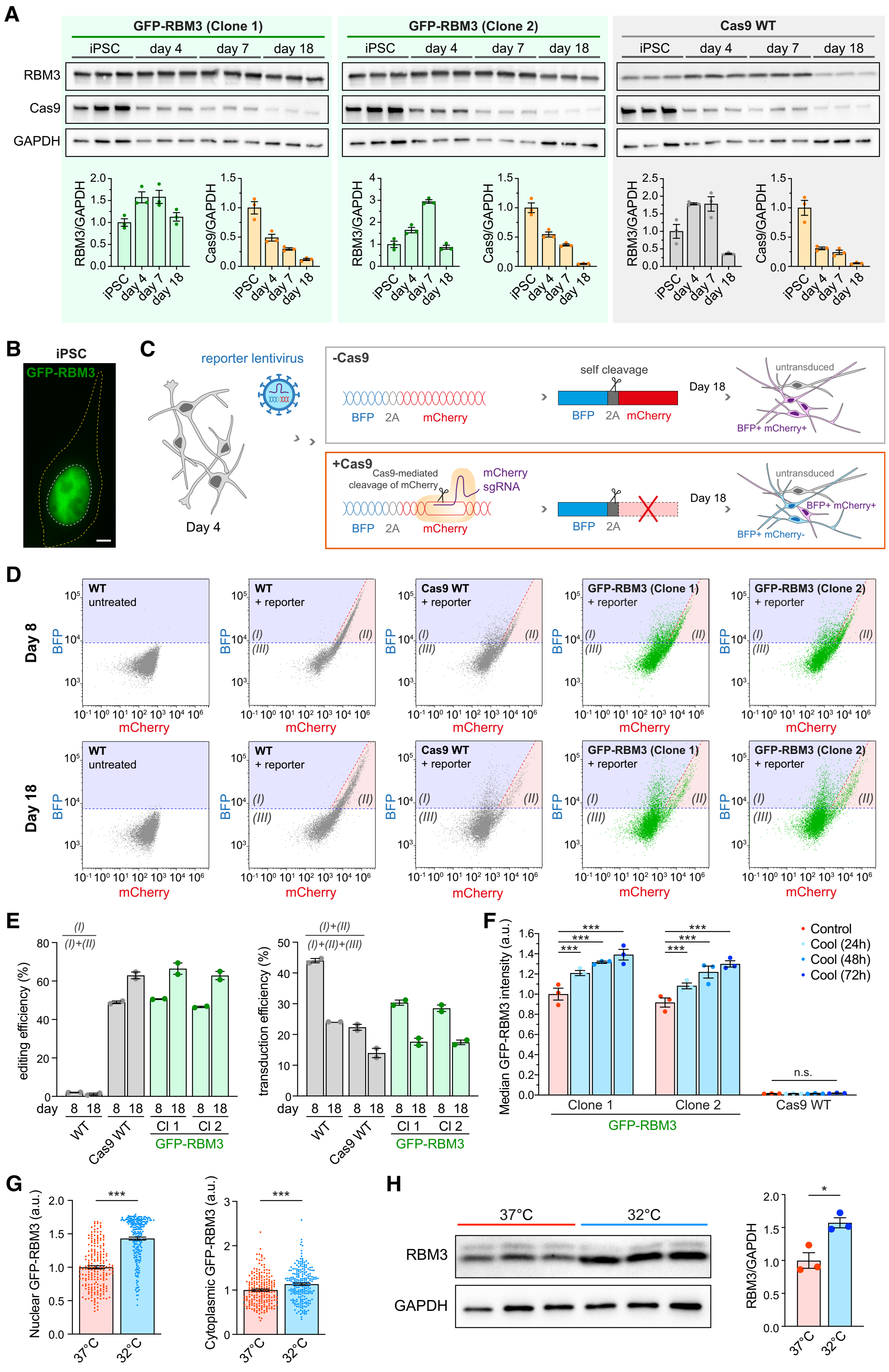

Figure EV1.

© 2023 The Authors

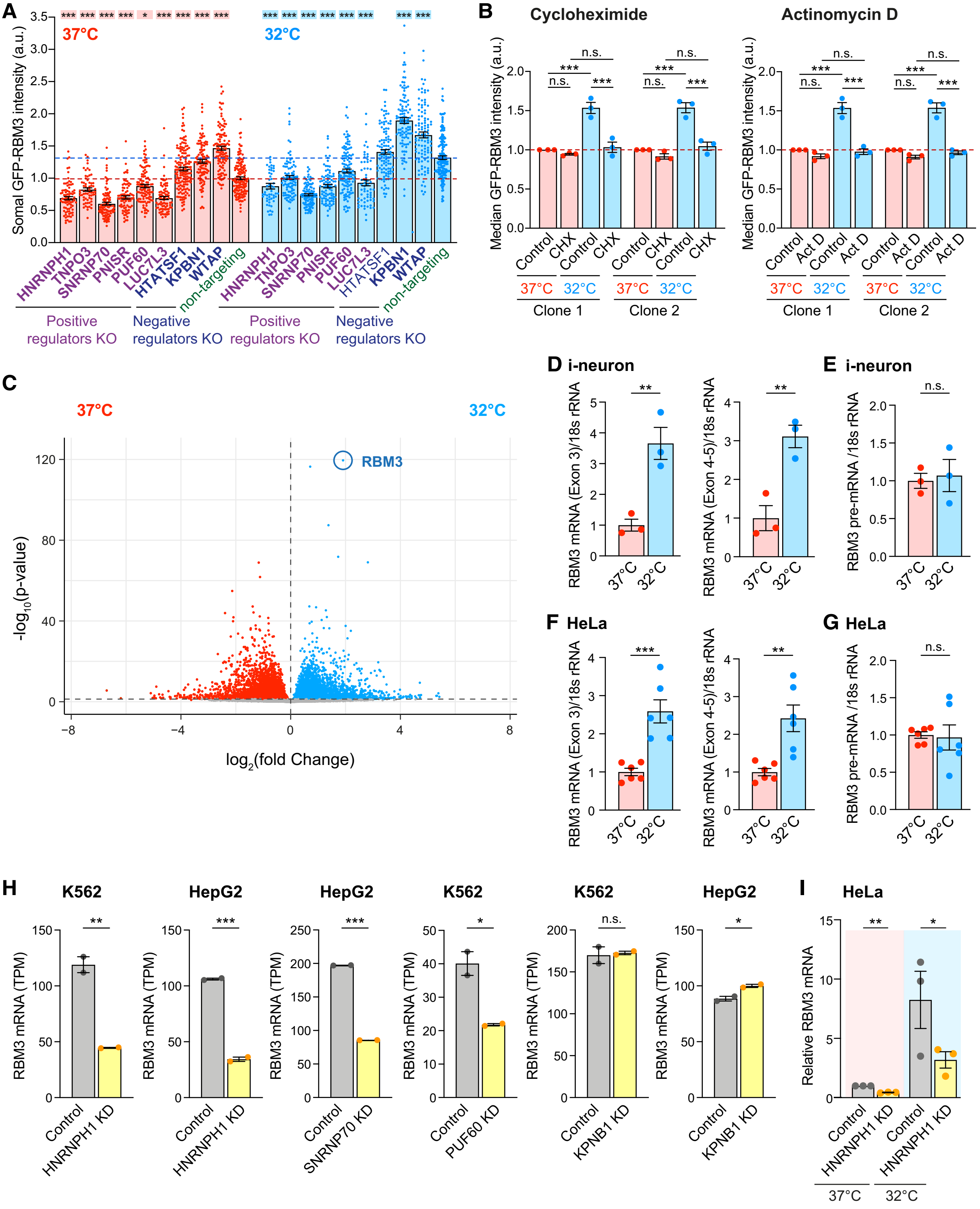
A
37°C
32°C
Somal GFP-RBM3 intensity (a.u.)
HNRNPH1
TNPO3
SNRNP70
PNISR
PUF60
LUC7L3
HTATSF1
KPBN1
WTAP
non-targeting
Positive regulators KO
Negative regulators KO
B
Cycloheximide
Actinomycin D
Median GFP-RBM3 intensity (a.u.)
Control
CHX
Act D
Clone 1
Clone 2
n.s.
C
RBM3
-log10(p-value)
log2(fold Change)
D i-neuron
E i-neuron
F HeLa
G HeLa
RBM3 mRNA (Exon 3)/18s rRNA
RBM3 mRNA (Exon 4-5)/18s rRNA
RBM3 pre-mRNA /18s rRNA
H
K562
HepG2
RBM3 mRNA (TPM)
HNRNPH1 KD
SNRNP70 KD
PUF60 KD
KPNB1 KD
I
HeLa
Relative RBM3 mRNA

Figure EV2.

Figure EV2. Cooling and regulators involved in mRNA splicing change RBM3 transcript levels. Related to Fig 2.

A Somal GFP intensity per unit area in GFP-RBM3 i-neurons transduced with lentivirus containing specific or non-targeting sgRNA at 37 or 32°C (72 h) imaged by wide-field microscopy. Only BFP-positive (transduced) cells are included. Statistical analysis is performed between the specific and non-targeting sgRNA within the temperature groups. Each data point represents one cell.

B Median GFP intensity per unit area of GFP-RBM3 i-neurons at 37 or 32°C (72 h) treated with cycloheximide (CHX) at 50 μM for 72 h or actinomycin D (Act D) at 1 μM for 72 h.

C Volcano plot showing differential expression analysis of all transcripts identified in i-neurons at 37 and 32°C (72 h) from RNA-Seq data.

D, E qRT-PCR of RBM3 Exon 3, Exon 4–5 (D) and pre-mRNA (E) normalised to 18 s rRNA in i-neurons at 37 and 32°C (72 h).

F, G qRT-PCR of RBM3 Exon 3, Exon 4–5 (F) and pre-mRNA (G) normalised to 18 s rRNA in HeLa cells at 37 and 32°C (48 h).

H Normalised RBM3 mRNA abundance (TPM) of control and selective regulator candidates knocked-down K562 or HepG2 cells. Data are extracted from ENCODE project. 2 isogenic replicates are included in each condition.

I qRT-PCR of RBM3 mRNA normalised to GAPDH in control and HNRNPH1 KD HeLa cells at 37 or 32°C (48 h).

Data information: $N = 3$ biological replicates. Mean ± SEM; n.s. (not significant), *($P < 0.05$), **($P < 0.01$), ***($P < 0.001$); one-way ANOVA with multiple comparisons in (A), unpaired t-tests in (B), (D)-(H); paired t-test in (I).
Source data are available online for this figure.

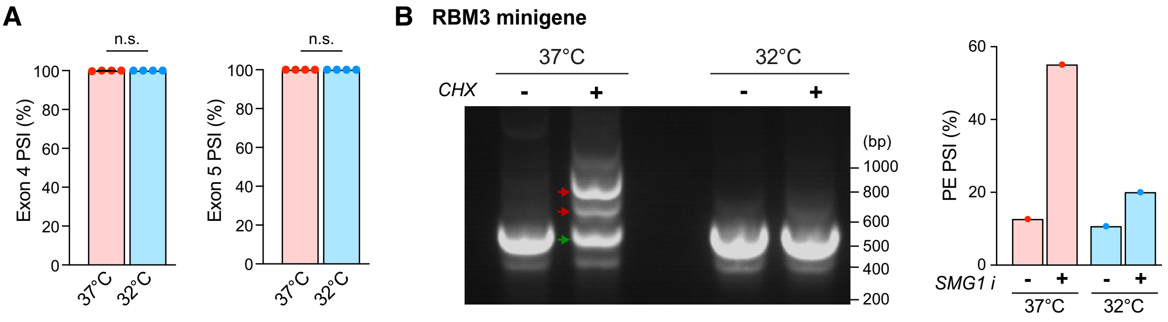

Figure EV3. Cooling represses RBM3 poison exon inclusion. Related to Fig 3.

A PSI values of RBM3 Exon 4 relative to Exon 3 and 6, and Exon 5 relative to Exon 4 and 6 in i-neurons at 37 or 32°C (72 h). $N = 4$ biological replicates.

B RT-PCR of RBM3 minigene expressed in HeLa cells at 37 and 32°C (48 h) in the presence or absence of cycloheximide (CHX) at 200 μg/ml concentration. PSI values of RBM3 PE are calculated based on the intensity of PE-included (red arrows) and PE-skipped (green arrow) isoforms. $N = 1$ biological replicate.

Data information: Mean ± SEM; n.s. (not significant); FDR calculated by rMATS program in (A).
Source data are available online for this figure.

Figure EV4. HNRNPH1 enhances RBM3 expression and RBM3 mRNA poison exon skipping on cooling. Related to Fig 4.

A Western blots and quantification of HNRNPH1 normalised to GAPDH in Control and HNRNPH1 KD Cas9WT i-neurons.

B Sashimi plots of the region between Exon 3 and 4 of RBM3 transcripts in control and HNRNPH1 knocked-down K562 and HepG2 cells, showing major alternatively spliced isoforms. Data are from ENCODE Project. 2 isogenic replicates are included.

C PSI values of RBM3 Exon 3a-S in control and HNRNPH1-knocked down K562 and HepG2 cells. RNA-Seq data from ENCODE Project, 2 isogenic replicates are included.

D qRT-PCR of HNRNPH1 mRNA normalised to GAPDH upon HNRNPH1 KD in HeLa cells.

E Western blots of FLAG-HNRNPH1 and GAPDH (loading control) in control and FLAG-HNRNPH1-expressed HEK293T cells at 37°C.

F Western blot of WT i-neurons transduced with lentivirus expressing BFP (control) or HNRNPH1-T2A-BFP (OX) at 37 and 32°C. The larger molecular weight of overexpressed HNRNPH1 is due to the additional amino acids between the C-terminus of HNRNPH1 and the T2A cleavage site.

G qRT-PCR of HNRNPH1 mRNA normalised to 18 s rRNA of WT i-neurons transduced with lentivirus expressing BFP (control) or HNRNPH1-T2A-BFP (OX) at 37 and 32°C.

H qRT-PCR quantifying the PSI values of RBM3 PE relative to RBM3 mRNA (mean value of RBM3 exon 3 and exon 4–5) in SMG1 inhibitor-treated control and HNRNPH1-overexpressing (OX) WT i-neurons at 37 and 32°C.

I Median GFP intensity of control and HNRNPH1-overexpressing (OX) GFP-RBM3 i-neurons measured by flow cytometry.

J Representative dot plots of flow cytometry data showing BFP or HNRNPH1-T2A-BFP expression (X-axis) and GFP intensity of successfully transduced (BFP-positive) GFP-RBM3 i-neurons. Each data point represents one cell. Cells with high levels of BFP expression (top 5% in each well) are coloured in red (37°C) or blue (32°C), and the rest of BFP-positive cells are in grey. Y-axes are in the same scale and X-axes are scaled to the sample.

Data information: $N = 3$ biological replicates. Mean ± SEM; n.s. (not significant), *($P < 0.05$), **($P < 0.01$), ***($P < 0.001$); unpaired t-tests in (A), (G), (H), (I), FDR calculated by rMATS program in (C), paired t-test in (D).
Source data are available online for this figure.

 © 2023 The Authors

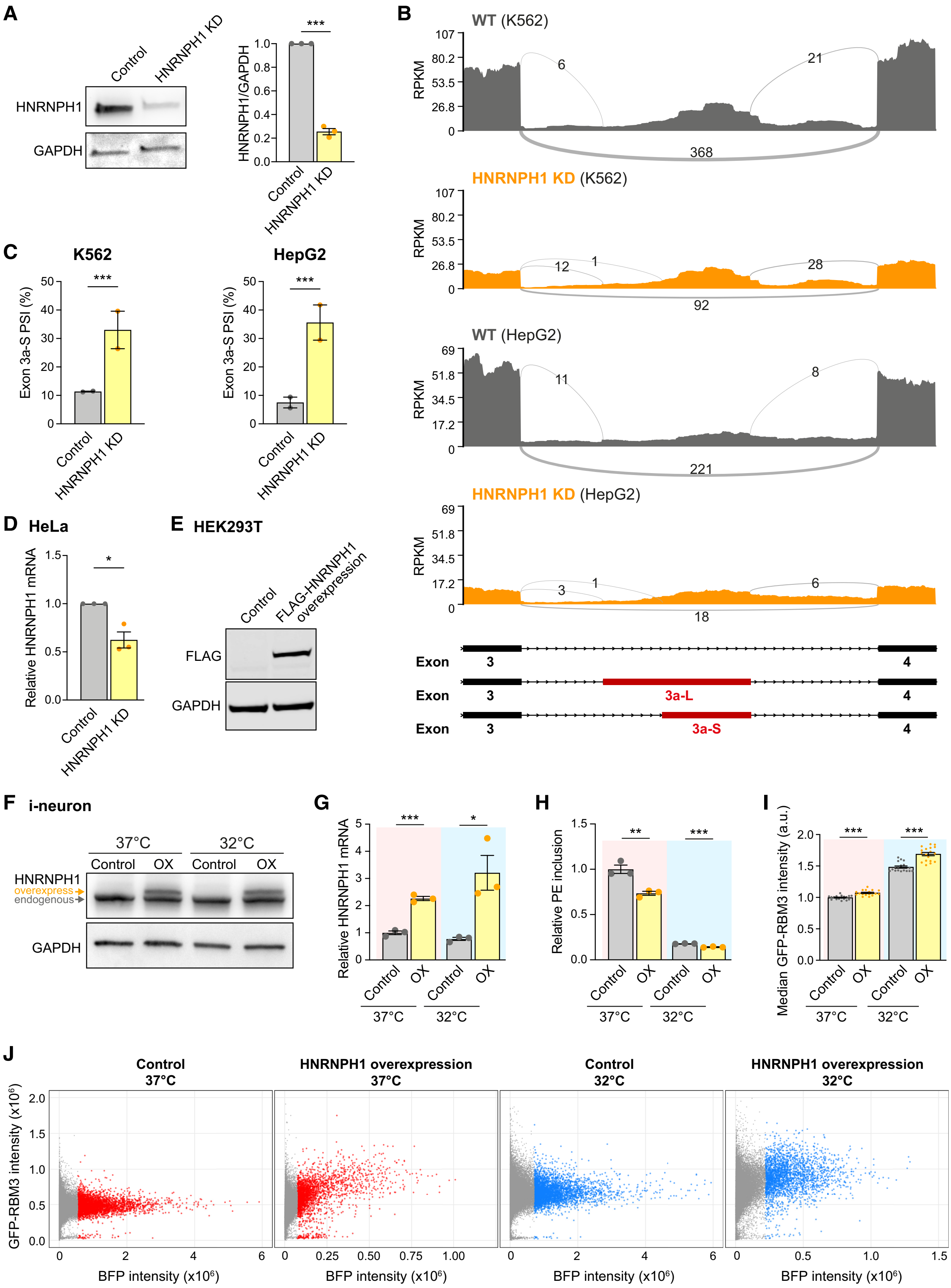

A
Control
HNRNPH1 KD
HNRNPH1
GAPDH
HNRNPH1/GAPDH
***
B
WT (K562)
HNRNPH1 KD (K562)
WT (HepG2)
HNRNPH1 KD (HepG2)
RPKM
Exon 3 4
3a-L
3a-S
C
K562
HepG2
Exon 3a-S PSI (%)
D HeLa
Relative HNRNPH1 mRNA
E HEK293T
FLAG-HNRNPH1 overexpression
FLAG
F i-neuron
37°C
32°C
Control OX
overexpress
endogenous
G
H
Relative PE inclusion
I
Median GFP-RBM3 intensity (a.u.)
J
Control 37°C
HNRNPH1 overexpression 37°C
Control 32°C
HNRNPH1 overexpression 32°C
GFP-RBM3 intensity (x10⁶)
BFP intensity (x10⁶)

Figure EV4.

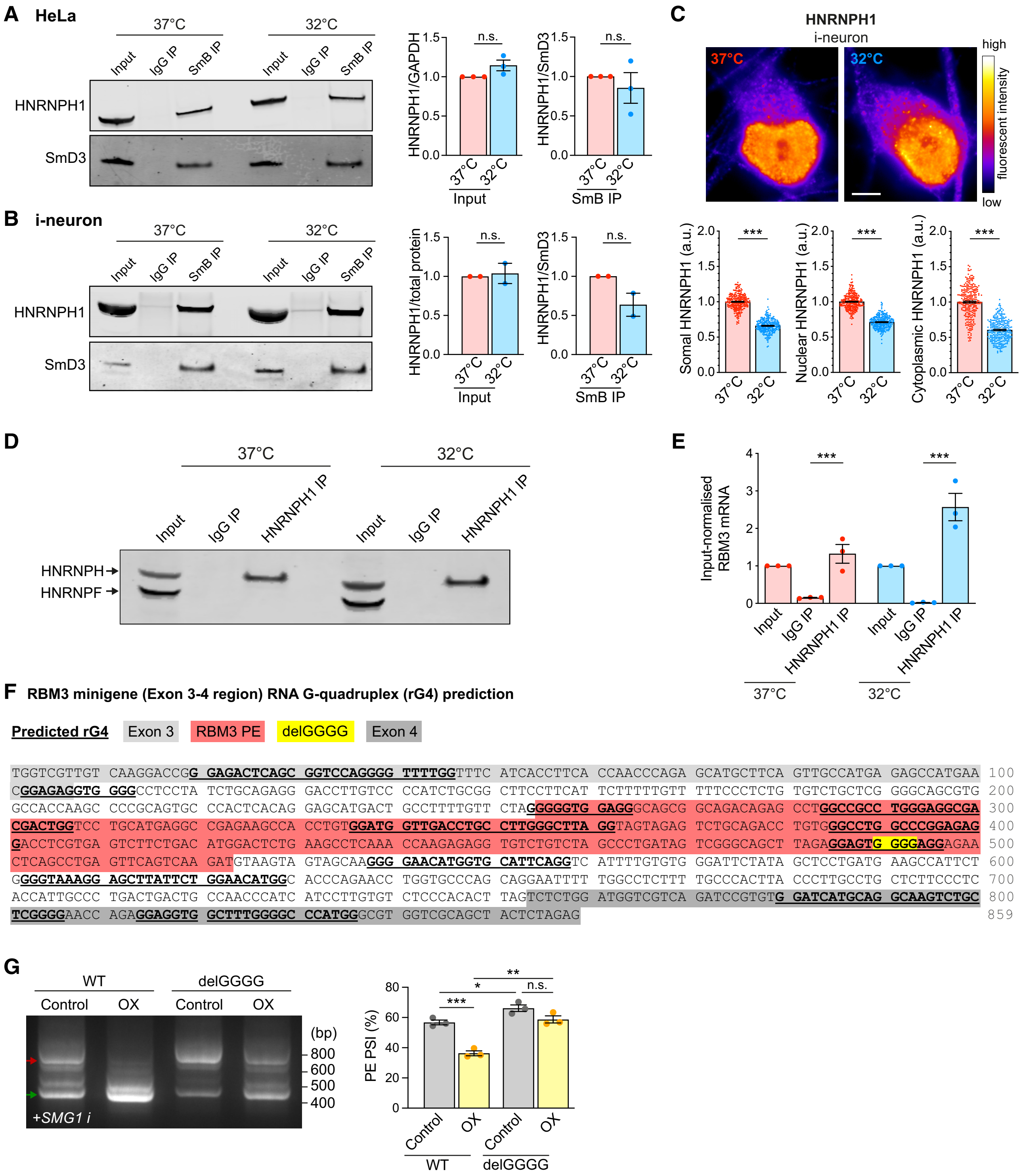

Figure EV5.

 © 2023 The Authors

Figure EV5. HNRNPH1 interacts with G-rich sequences in RBM3 poison exon in a temperature-dependent manner. Related to Fig 5.

A Western blot and quantification of HNRNPH1 total protein levels (input HNRNPH1 normalised to GAPDH) and its abundance in spliceosomal protein SmB pulldown (normalised to spliceosomal protein SmD3) in HeLa cells at 37 and 32°C (72 h).

B Western blot and quantification of HNRNPH1 total protein levels (input HNRNPH1 normalised to ponceau measured total protein abundance) and its abundance in spliceosomal protein SmB pulldown (normalised to spliceosomal protein SmD3) in i-neurons at 37 and 32°C (72 h).

C Representative images of HNRNPH1 staining of GFP-RBM3 i-neurons at 37°C or after 72 h cooling at 32°C. Graphs below the images show quantification of somal, nuclear and cytoplasmic intensity per unit area respectively. The decrease of HNRNPH1 signals at 32°C is due to the global attenuation of protein production, which is not seen after total protein or GAPDH normalisation in western blot in (B) and Fig 4A. $N = 280$ (37°C) and 262 (32°C) cells. Scale bar: 5 μm.

D Western blot of HNRNPH/F in HeLa cell input (total) lysate, IgG-pulled down and HNRNPH1-pulled down eluates at 37 and 32°C (48 h).

E Quantification of input-normalised RBM3 mRNA levels in HeLa cell input (total), IgG-pulled down and HNRNPH1-pulled down RNA at 37 and 32°C (48 h).

F RNA G quadruplexes (rG4) within the RBM3 Exon 3–4 region are predicted using QGRS mapper. Deletion of the GGGG motif in the mutant RBM3 minigene is predicted to disrupt the rG4 structure overlapping this region.

G RT-PCR of WT and delGGGG RBM3 minigenes in control or HNRNPH1-overexpressing (OX) HEK293T cells treated with SMG1 inhibitor at 37°C. PSI values of RBM3 PE are shown in the graphs on the right.

Data information: $N = 3$ biological replicates, except (B), which has $N = 2$. Mean ± SEM; n.s. (not significant), *($P < 0.05$), **($P < 0.01$), ***($P < 0.001$); paired t-test in (A), (B), (E), (G), unpaired t-tests in (C).
Source data are available online for this figure.

Appendix Supplementary Tables

Appendix Table S1 Single-guide RNA (sgRNA) and primer (Pr) sequences used in this study (Page 2)

Appendix Table S2 RBM3 positive and negative regulator candidates identified by pooled CRISPR knockout screen (Page 5)

Appendix Table S3 A complete list of significant Gene Ontology (GO) terms of RBM3 positive and negative regulators (Page 16)

Appendix Table S4 sgRNA sequences used in arrayed target validation (Page 23)

Appendix Table S5 TPM of RBM3 mRNA upon specified RBM3 regulator KD compared to the control in K562 and HepG2 cells from ENCODE (Page 33)

Appendix Table S6 Analysis of RBM3 transcript alternative splicing in HNRNPH1 KD versus WT i-neurons, K562 and HepG2 cells by rMATS (Page 34)

Appendix Table S7 Key resources used in this study (Page 35)

Appendix Table S1 Single-guide RNA (sgRNA) and primer (Pr) sequences used in this study

Number	Name	Sequence (5' -> 3')
Pr1	AmpOri_RBM3-N-5HA_Fw	GGGTTCCGCGCACATTTCCCCGAAAAGTGCGGTGAAGGAGGTTAACATCAGAGAATGGCA
Pr2	RBM3-N-5HA_GFP_Rv	GGTGAACAGCTCCTCGCCCTTGCTCACCATGGCAGTTCAAGTCCTGTGGGAAGAACATGA
Pr3	RBM3-N-5HA_GFP_Fw	TCATGTTCTTCCCACAGGACTTGAACTGCCATGGTGAGCAAGGGCGAGGAGCTGTTCACC
Pr4	GFP_linker_RBM3-N-3HA_Rv	TCCCACGAAGAGCTTTCCTTCTTCAGAGGAGAATTCTCCTGAACCGGCCGCCGATCCTGCGGAGCCCTTGTACAGCTCGTCCATGCCGAGAG
Pr5	GFP_linker_RBM3-N-3HA_Fw	ATCACTCTCGGCATGGACGAGCTGTACAAGGGCTCCGCAGGATCGGCGGCCGGTTCAGGAGAATTCTCCTCTGAAGAAGGAAAGCTCTTCGTGGGA
Pr6	RBM3-N-3HA_AmpOri_Rv	CTGGCACGACAGGTTTCCCGACTGGAAAGCAAGCTGCCCGACTATCAGGGCTAGACAGAC
Pr7	RBM3-N-3HA_AmpOri_Fw	GTCTGTCTAGCCCTGATAGTCGGGCAGCTTGCTTTCCAGTCGGGAAACCTGTCGTGCCAG
Pr8	AmpOri_RBM3-N-5HA_Rv	TGCCATTCTCTGATGTTAACCTCCTTCACCGCACTTTTCGGGGAAATGTGCGCGGAACCC
Pr9	RBM3-N_ssDNA_PF	(5'-phosphorylated) CGTGCTTGGAGGTGAAACATTTCG
Pr10	RBM3-N_ssDNA_R	GCTCTCATGGCAACTGAAGCA
Pr11	GT_RBM3-N_ssDNA_F	TGCATATGTCCTGCGAAACG
Pr12	GT_RBM3-N_ssDNA_R	AGTCATGCTCCTGTGAGTGG
Pr13	GT_GFP_F	GCGAGGAGCTGTTCACCGG
Pr14	GT_GFP_R	CCATGTGATCGCGCTTCTCGT
Pr15	Vector 1st half_Fw	CGACCGGTAGCGCTAGGATCCCCCCTGGGGAGAGAGGTCGGTGATTCG
Pr16	Vector 1st half_Rv	CTCTGCTAATCCTGTTACCAGTGGCTGC
Pr17	Vector 2nd half_Fw	GCAGCCACTGGTAACAGGATTAGCAGAG
Pr18	Vector 2nd half_Rv	ACCCAGCTTTCTTGTACAAAGTGGT
Pr19	HNRNPH1_Fw	GGGGATCCTAGCGCTACCGGTCGCCACCATGATGTTGGGCACGGAAGGTGGAG
Pr20	HNRNPH1_Rv	GACTTCCTCTGCCCTCACCGGATCCTGCAATGTTTGATTGAAAATCACTGGAGTTTTCC

Pr21	T2A-TagBFP_Fw	GGATCCGGTGAGGGCAGAGGAAGTC
Pr22	T2A-TagBFP_Rv	ACCACTTTGTACAAGAAAGCTGGGTTCAATTAAGCTTGTGCCCCAGTTTGCTAGGG
Pr23	qPCR_h18S_F	GTAACCCGTTGAACCCCATT
Pr24	qPCR_h18S_R	CCATCCAATCGGTAGTAGCG
Pr25	qPCR_hRBM3_e3_F	GGACCTATCTCTGAGGTGGTCG
Pr26	qPCR_hRBM3_e3_R	TTCATGGCTCTCATGGCAACTG
Pr27	qPCR_hRBM3_e4-5_F	TCAGATCCGTGTGGATCATG
Pr28	qPCR_hRBM3_e4-5_R	TAATACCTGCCACTCCCATAGCC
Pr29	qPCR_hRBM3_e3a_F	GTAGTAGAGTCTGCAGACCTGTGG
Pr30	qPCR_hRBM3_e3a_R	TCCTCTAAGCTGCCCGACTATC
Pr31	hRBM3_RTPCR_fwd_DK79	GTGGGAGGGCTCAACTTTAAC
Pr32	hRBM3_RTPCR_rev_DK80	CATGATCCACACGGATCTGAC
Pr33	hRBM3_RTPCR_fwd pair 2_DK81	AGGACCGGGAGACTCAGC
Pr34	hRBM3_RTPCR_rev pair 2_DK82	AGCCACCTCCTCTGGTTC
Pr35	hRBM3_Minigene_fwd_DK20F	GCTCGGATCCACTAGTCCAGTGT
Pr36	hRBM3_Minigene_rev_DK21R	GTTTAAACGGGCCCTCTAGACT
Pr37	hRBM3 qPCR ex2_fwd_DK30	TCAGCAGTTTCGGACCTATC
Pr38	hRBM3 qPCR ex3_rev_DK31	CATGCTCTGGGTTGGTGAAG
Pr39	GAPDH qpCR_fwd	CCAGAACATCATCCCTGCCT
Pr40	GAPDH qpCR_rev	GGTCAGGTCCACCACTGACA
Pr41	mRBM3 ex2 f _DK184	CTGCCATGTCGTCTG
Pr42	mRBM3 ex4 r_DK185	GATTTGGCGCCCATC
Pr43	HNRNPH qPCR_fwd	GGCCAATGATGGCTTTGTACG

Pr44	HNRNPH qPCR_rev	AGTCCACCGGCAATGTTATCC
Pr45	hRBM3 qPCR_premRNA_ex2_fwd	TGGGAGGGCTCAACTTTAAC
Pr46	hRBM3 qPCR_premRNA_in2_rev	TGCAAGGATCCCAGACTTTC
Pr47	qPCR_hHNRNPH1e11_F	GTGGTGCTTACGAACACAGATATG
Pr48	qPCR_hHNRNPH1e11_R	GCTGGACTGGTTTGACAAGC
Pr49	Pi7_PLVPBnewSeq	GTCTCGTGGGCTCGGAGATGTGTATAAGAGACAGACTCGGTGCCACTTTTTCAA
Pr50	Pi5_PLVPBnobarcode	TCGTCGGCAGCGTCAGATGTGTATAAGAGACAGTCTTGTGGAAAGGACGAAACA
iP7_1n	Q7005	CAAGCAGAAGACGGCATACGAGATATATTCACGTCTCGTGGGCTCGG
iP7_2n	Q7006	CAAGCAGAAGACGGCATACGAGATGCGCCTGTGTCTCGTGGGCTCGG
iP7_3n	Q7007	CAAGCAGAAGACGGCATACGAGATACTCTATGGTCTCGTGGGCTCGG
iP7_4n	Q7008	CAAGCAGAAGACGGCATACGAGATGTCTCGCAGTCTCGTGGGCTCGG
iP7_5n	Q7015	CAAGCAGAAGACGGCATACGAGATAGTAGAGAGTCTCGTGGGCTCGG
iP7_6n	Q7016	CAAGCAGAAGACGGCATACGAGATGACGAGAGGTCTCGTGGGCTCGG
iP7_7n	Q7017	CAAGCAGAAGACGGCATACGAGATAGACTTGGGTCTCGTGGGCTCGG
iP7_8n	Q7018	CAAGCAGAAGACGGCATACGAGATGAGTCCAAGTCTCGTGGGCTCGG
iP7_9n	Q7023	CAAGCAGAAGACGGCATACGAGATAATTCTGCGTCTCGTGGGCTCGG
iP7_10n	Q7024	CAAGCAGAAGACGGCATACGAGATGGCCTCATGTCTCGTGGGCTCGG
iP7_11n	Q7025	CAAGCAGAAGACGGCATACGAGATATCTTAGTGTCTCGTGGGCTCGG
iP7_12n	Q7026	CAAGCAGAAGACGGCATACGAGATGCTCCGACGTCTCGTGGGCTCGG
iP5_1n	Q5001	AATGATACGGCGACCACCGAGATCTACACAGCGCTAGTCGTCGGCAGCGTC
iP5_2n	Q5002	AATGATACGGCGACCACCGAGATCTACACGATATCGATCGTCGGCAGCGTC
iP5_3n	Q5003	AATGATACGGCGACCACCGAGATCTACACCGCAGACGTCGTCGGCAGCGTC
iP5_4n	Q5004	AATGATACGGCGACCACCGAGATCTACACTATGAGTATCGTCGGCAGCGTC

iP5_5n	Q5007	AATGATACGGCGACCACCGAGATCTACACACATAGCGTCGTCGGCAGCGTC
iP5_6n	Q5008	AATGATACGGCGACCACCGAGATCTACACGTGCGATATCGTCGGCAGCGTC
iP5_7n	Q5009	AATGATACGGCGACCACCGAGATCTACACCCAACAGATCGTCGGCAGCGTC
iP5_8n	Q5010	AATGATACGGCGACCACCGAGATCTACACTTGGTGAGTCGTCGGCAGCGTC

Appendix Table S2 RBM3 positive and negative regulator candidates identified by pooled CRISPR knockout screen

Positive/Negative regulator	Rank	ID	Gene symbol	FDR
Positive	1	ENSG00000169045	HNRNPH1	0.00165
Positive	2	ENSG00000102317	RBM3	0.00165
Positive	3	ENSG00000064419	TNPO3	0.00165
Positive	4	ENSG00000132424	PNISR	0.00165
Positive	5	ENSG00000086758	HUWE1	0.00165
Positive	6	ENSG00000104852	SNRNP70	0.00165
Positive	7	ENSG00000179950	PUF60	0.001856
Positive	8	ENSG00000118263	KLF7	0.001856
Positive	9	ENSG00000130699	TAF4	0.0022
Positive	10	ENSG00000130811	EIF3G	0.009406
Positive	11	ENSG00000177600	RPLP2	0.009451
Positive	12	ENSG00000106263	EIF3B	0.021325
Positive	13	ENSG00000141867	BRD4	0.021325
Positive	14	ENSG00000108848	LUC7L3	0.032885
Positive	15	ENSG00000151651	ADAM8	0.047855

Positive	16	ENSG00000134684	YARS	0.064356
Positive	17	ENSG00000120156	TEK	0.064356
Positive	18	ENSG00000131759	RARA	0.115787
Positive	19	ENSG00000165417	GTF2A1	0.16493
Positive	20	ENSG00000087095	NLK	0.200495
Positive	21	ENSG00000136485	DCAF7	0.200495
Positive	22	ENSG00000099139	PCSK5	0.200495
Positive	23	ENSG00000187758	ADH1A	0.251031
Positive	24	ENSG00000120833	SOCS2	0.251031
Positive	25	ENSG00000196361	ELAVL3	0.252277
Positive	26	ENSG00000171861	MRM3	0.255331
Positive	27	ENSG00000062038	CDH3	0.281628
Positive	28	ENSG00000135750	KCNK1	0.291396
Positive	29	ENSG00000174125	TLR1	0.291396
Positive	30	ENSG00000155545	MIER3	0.306106
Positive	31	ENSG00000100393	EP300	0.331219
Positive	32	ENSG00000182831	C16orf72	0.331219
Positive	33	ENSG00000170852	KBTBD2	0.358836
Positive	34	ENSG00000105327	BBC3	0.479663
Positive	35	ENSG00000172935	MRGPRF	0.479663
Positive	36	ENSG00000084623	EIF3I	0.479663
Positive	37	ENSG00000056097	ZFR	0.479663
Positive	38	ENSG00000126247	CAPNS1	0.54703

Positive	39	ENSG00000080618	CPB2	0.547728
Positive	40	ENSG00000156531	PHF6	0.606312
Positive	41	ENSG00000172530	BANP	0.613258
Positive	42	ENSG00000065427	KARS	0.69791
Positive	43	ENSG00000153046	CDYL	0.69791
Positive	44	ENSG00000174080	CTSF	0.69791
Positive	45	ENSG00000122741	DCAF10	0.69791
Positive	46	ENSG00000198380	GFPT1	0.704162
Positive	47	ENSG00000184254	ALDH1A3	0.704162
Positive	48	ENSG00000175197	DDIT3	0.704162
Positive	49	ENSG00000176896	TCEANC	0.704162
Positive	50	ENSG00000138741	TRPC3	0.704162
Positive	51	ENSG00000170091	NSG2	0.704162
Positive	52	ENSG00000028203	VEZT	0.704162
Positive	53	ENSG00000131469	RPL27	0.704162
Positive	54	ENSG00000160678	S100A1	0.704162
Positive	55	ENSG00000099957	P2RX6	0.709361
Positive	56	ENSG00000169230	PRELID1	0.751149
Positive	57	ENSG00000130032	PRRG3	0.798072
Positive	58	ENSG00000176946	THAP4	0.801087
Positive	59	ENSG00000144035	NAT8	0.801087
Positive	60	ENSG00000119714	GPR68	0.801087
Positive	61	ENSG00000133067	LGR6	0.801087

Positive	62	ENSG00000177963	RIC8A	0.824098
Positive	63	ENSG00000090861	AARS	0.82418
Positive	64	ENSG00000184156	KCNQ3	0.82418
Positive	65	ENSG00000213988	ZNF90	0.824476
Positive	66	ENSG00000125648	SLC25A23	0.824476
Positive	67	ENSG00000107331	ABCA2	0.824476
Positive	68	ENSG00000108443	RPS6KB1	0.824476
Positive	69	ENSG00000106615	RHEB	0.834912
Positive	70	ENSG00000181222	POLR2A	0.856223
Positive	71	ENSG00000115942	ORC2	0.85783
Positive	72	ENSG00000186487	MYT1L	0.88937
Positive	73	ENSG00000038002	AGA	0.898617
Negative	1	ENSG00000102241	HTATSF1	0.000381
Negative	2	ENSG00000119801	YPEL5	0.000381
Negative	3	ENSG00000108424	KPNB1	0.000381
Negative	4	ENSG00000162923	WDR26	0.000381
Negative	5	ENSG00000146457	WTAP	0.000381
Negative	6	ENSG00000176887	SOX11	0.000381
Negative	7	ENSG00000122692	SMU1	0.000381
Negative	8	ENSG00000122882	ECD	0.000381
Negative	9	ENSG00000129351	ILF3	0.000381
Negative	10	ENSG00000165699	TSC1	0.000381
Negative	11	ENSG00000100138	SNU13	0.000381

Negative	12	ENSG00000186591	UBE2H	0.000381
Negative	13	ENSG00000161547	SRSF2	0.000381
Negative	14	ENSG00000115875	SRSF7	0.00099
Negative	15	ENSG00000204569	PPP1R10	0.00099
Negative	16	ENSG00000171862	PTEN	0.001547
Negative	17	ENSG00000114209	PDCD10	0.002038
Negative	18	ENSG00000105618	PRPF31	0.002475
Negative	19	ENSG00000125952	MAX	0.002866
Negative	20	ENSG00000171634	BPTF	0.003536
Negative	21	ENSG00000169564	PCBP1	0.003536
Negative	22	ENSG00000135473	PAN2	0.00409
Negative	23	ENSG00000060339	CCAR1	0.00409
Negative	24	ENSG00000134884	ARGLU1	0.004332
Negative	25	ENSG00000163635	ATXN7	0.004379
Negative	26	ENSG00000135801	TAF5L	0.004379
Negative	27	ENSG00000115607	IL18RAP	0.004584
Negative	28	ENSG00000108883	EFTUD2	0.007603
Negative	29	ENSG00000139549	DHH	0.008706
Negative	30	ENSG00000086598	TMED2	0.009736
Negative	31	ENSG00000141568	FOXK2	0.011019
Negative	32	ENSG00000182591	KRTAP11-1	0.011551
Negative	33	ENSG00000172927	MYEOV	0.011551
Negative	34	ENSG00000163605	PPP4R2	0.011794

Negative	35	ENSG00000175550	DRAP1	0.01372
Negative	36	ENSG00000105393	BABAM1	0.015264
Negative	37	ENSG00000198258	UBL5	0.015763
Negative	38	ENSG00000101193	GID8	0.015763
Negative	39	ENSG00000134186	PRPF38B	0.016882
Negative	40	ENSG00000112110	MRPL18	0.019431
Negative	41	ENSG00000058262	SEC61A1	0.023304
Negative	42	ENSG00000174547	MRPL11	0.024163
Negative	43	ENSG00000141526	SLC16A3	0.026665
Negative	44	ENSG00000156471	PTDSS1	0.026665
Negative	45	ENSG00000173011	TADA2B	0.027741
Negative	46	ENSG00000183091	NEB	0.027741
Negative	47	ENSG00000109189	USP46	0.027741
Negative	48	ENSG00000100427	MLC1	0.027741
Negative	49	ENSG00000229549	TSPY8	0.027741
Negative	50	ENSG00000164151	ICE1	0.027741
Negative	51	ENSG00000162300	ZFPL1	0.027741
Negative	52	ENSG00000143569	UBAP2L	0.027741
Negative	53	ENSG00000167088	SNRPD1	0.027741
Negative	54	ENSG00000129562	DAD1	0.028144
Negative	55	ENSG00000172409	CLP1	0.029433
Negative	56	ENSG00000198754	OXCT2	0.031206
Negative	57	ENSG00000111859	NEDD9	0.033959

Negative	58	ENSG00000136875	PRPF4	0.034568
Negative	59	ENSG00000008118	CAMK1G	0.035891
Negative	60	ENSG00000160993	ALKBH4	0.035891
Negative	61	ENSG00000185963	BICD2	0.036114
Negative	62	ENSG00000011083	SLC6A7	0.037325
Negative	63	ENSG00000196655	TRAPPC4	0.037325
Negative	64	ENSG00000172967	XKR3	0.038029
Negative	65	ENSG00000156171	DRAM2	0.038029
Negative	66	ENSG00000120948	TARDBP	0.038029
Negative	67	ENSG00000129514	FOXA1	0.040607
Negative	68	ENSG00000140534	TICRR	0.040607
Negative	69	ENSG00000074181	NOTCH3	0.040607
Negative	70	ENSG00000158966	CACHD1	0.040607
Negative	71	ENSG00000112701	SENP6	0.040607
Negative	72	ENSG00000136709	WDR33	0.040607
Negative	73	ENSG00000166189	HPS6	0.040607
Negative	74	ENSG00000102554	KLF5	0.040607
Negative	75	ENSG00000172465	TCEAL1	0.043708
Negative	76	ENSG00000134982	APC	0.043708
Negative	77	ENSG00000185278	ZBTB37	0.044109
Negative	78	ENSG00000105662	CRTC1	0.044109
Negative	79	ENSG00000092201	SUPT16H	0.044109
Negative	80	ENSG00000125818	PSMF1	0.044109

Negative	81	ENSG00000103647	CORO2B	0.044109
Negative	82	ENSG00000196236	XPNPEP3	0.044109
Negative	83	ENSG00000057704	TMCC3	0.044109
Negative	84	ENSG00000182872	RBM10	0.044109
Negative	85	ENSG00000143379	SETDB1	0.044109
Negative	86	ENSG00000176909	MAMSTR	0.044109
Negative	87	ENSG00000157322	CLEC18A	0.044109
Negative	88	ENSG00000182325	FBXL6	0.044109
Negative	89	ENSG00000104921	FCER2	0.044109
Negative	90	ENSG00000189253	TRIM64B	0.04648
Negative	91	ENSG00000159140	SON	0.047492
Negative	92	ENSG00000176020	AMIGO3	0.048913
Negative	93	ENSG00000007866	TEAD3	0.04987
Negative	94	ENSG00000065911	MTHFD2	0.04987
Negative	95	ENSG00000131899	LLGL1	0.04987
Negative	96	ENSG00000095321	CRAT	0.051337
Negative	97	ENSG00000170255	MRGPRX1	0.051337
Negative	98	ENSG00000157110	RBPMS	0.051337
Negative	99	ENSG00000160226	C21orf2	0.051337
Negative	100	ENSG00000161940	BCL6B	0.051337
Negative	101	ENSG00000185352	HS6ST3	0.055632
Negative	102	ENSG00000254709	IGLL5	0.057416
Negative	103	ENSG00000176476	SGF29	0.062722

Negative	104	ENSG00000164048	ZNF589	0.064074
Negative	105	ENSG00000143995	MEIS1	0.064074
Negative	106	ENSG00000204070	SYS1	0.066365
Negative	107	ENSG00000112983	BRD8	0.069446
Negative	108	ENSG00000102265	TIMP1	0.069536
Negative	109	ENSG00000110651	CD81	0.070297
Negative	110	ENSG00000101191	DIDO1	0.070297
Negative	111	ENSG00000145216	FIP1L1	0.070297
Negative	112	ENSG00000110063	DCPS	0.070297
Negative	113	ENSG00000164944	VIRMA	0.070297
Negative	114	ENSG00000259956	RBM15B	0.070297
Negative	115	ENSG00000179564	LSMEM2	0.070297
Negative	116	ENSG00000101104	PABPC1L	0.074091
Negative	117	ENSG00000126226	PCID2	0.074091
Negative	118	ENSG00000160963	COL26A1	0.074091
Negative	119	ENSG00000176978	DPP7	0.074091
Negative	120	ENSG00000186105	LRRC70	0.074413
Negative	121	ENSG00000125449	ARMC7	0.074413
Negative	122	ENSG00000069869	NEDD4	0.074413
Negative	123	ENSG00000168906	MAT2A	0.074413
Negative	124	ENSG00000204228	HSD17B8	0.074413
Negative	125	ENSG00000244405	ETV5	0.074413
Negative	126	ENSG00000110321	EIF4G2	0.074413

Negative	127	ENSG00000175198	PCCA	0.074413
Negative	128	ENSG00000185272	RBM11	0.075688
Negative	129	ENSG00000153113	CAST	0.07702
Negative	130	ENSG00000160310	PRMT2	0.078945
Negative	131	ENSG00000187189	TSPYL4	0.078945
Negative	132	ENSG00000075856	SART3	0.078945
Negative	133	ENSG00000106245	BUD31	0.079318
Negative	134	ENSG00000170613	FAM71B	0.079318
Negative	135	ENSG00000189319	FAM53B	0.079318
Negative	136	ENSG00000079102	RUNX1T1	0.079528
Negative	137	ENSG00000117461	PIK3R3	0.079528
Negative	138	ENSG00000185176	AQP12B	0.079528
Negative	139	ENSG00000184916	JAG2	0.079528
Negative	140	ENSG00000140691	ARMC5	0.079562
Negative	141	ENSG00000159217	IGF2BP1	0.079581
Negative	142	ENSG00000128815	WDFY4	0.079581
Negative	143	ENSG00000105829	BET1	0.079581
Negative	144	ENSG00000128951	DUT	0.079581
Negative	145	ENSG00000184678	HIST2H2BE	0.079581
Negative	146	ENSG00000089818	NECAP1	0.079581
Negative	147	ENSG00000149260	CAPN5	0.08376
Negative	148	ENSG00000103326	CAPN15	0.08376
Negative	149	ENSG00000213937	CLDN9	0.08376

Negative	150	ENSG00000187555	USP7	0.083765
Negative	151	ENSG00000047410	TPR	0.083765
Negative	152	ENSG00000135363	LMO2	0.083998
Negative	153	ENSG00000114784	EIF1B	0.083998
Negative	154	ENSG00000126752	SSX1	0.083998
Negative	155	ENSG00000103510	KAT8	0.084286
Negative	156	ENSG00000029364	SLC39A9	0.086983
Negative	157	ENSG00000134809	TIMM10	0.088699
Negative	158	ENSG00000236334	PPIAL4G	0.089139
Negative	159	ENSG00000135045	C9orf40	0.089139
Negative	160	ENSG00000198133	TMEM229B	0.089139
Negative	161	ENSG00000215704	CELA2B	0.089139
Negative	162	ENSG00000061987	MON2	0.089139
Negative	163	ENSG00000177732	SOX12	0.089139
Negative	164	ENSG00000186086	NBPF6	0.090226
Negative	165	ENSG00000104731	KLHDC4	0.092185
Negative	166	ENSG00000172888	ZNF621	0.092185
Negative	167	ENSG00000011485	PPP5C	0.092185
Negative	168	ENSG00000109101	FOXN1	0.092185
Negative	169	ENSG00000134453	RBM17	0.092185
Negative	170	ENSG00000177519	RPRM	0.092691
Negative	171	ENSG00000100218	RSPH14	0.092901
Negative	172	ENSG00000196890	HIST3H2BB	0.093858

Negative	173	ENSG00000136541	ERMN	0.095433
Negative	174	ENSG00000161800	RACGAP1	0.099409
Negative	175	ENSG00000101412	E2F1	0.099409
Negative	176	ENSG00000111906	HDDC2	0.099409
Negative	177	ENSG00000196666	FAM180B	0.099409
Negative	178	ENSG00000164091	WDR82	0.099409
Negative	179	ENSG00000184863	RBM33	0.099409
Negative	180	ENSG00000100320	RBFOX2	0.099409
Negative	181	ENSG00000168484	SFTPC	0.099409
Negative	182	ENSG00000143493	INTS7	0.099409
Negative	183	ENSG00000235194	PPP1R3E	0.099409
Negative	184	ENSG00000082438	COBLL1	0.099409
Negative	185	ENSG00000137601	NEK1	0.099409
Negative	186	ENSG00000176571	CNBD1	0.099409

Appendix Table S3 A complete list of significant Gene Ontology (GO) terms of RBM3 positive and negative regulators

Positive/Negative regulator	GO	Category	Description	LogP	Hits
**Positive**	**GO:0022618**	**GO Biological Processes**	**ribonucleoprotein complex assembly**	**-5.602**	**EIF3B|EIF3G|PUF60|LUC7L3**
Positive	GO:0071826	GO Biological Processes	ribonucleoprotein complex subunit organization	-5.540	EIF3B|EIF3G|PUF60|LUC7L3
Positive	R-HSA-72689	Reactome Gene Sets	Formation of a pool of free 40S subunits	-4.890	RPLP2|EIF3B|EIF3G
Positive	R-HSA-156827	Reactome Gene Sets	L13a-mediated translational silencing of Ceruloplasmin expression	-4.767	RPLP2|EIF3B|EIF3G

Positive	R-HSA-72706	Reactome Gene Sets	GTP hydrolysis and joining of the 60S ribosomal subunit	-4.756	RPLP2|EIF3B|EIF3G
Positive	R-HSA-72613	Reactome Gene Sets	Eukaryotic Translation Initiation	-4.677	RPLP2|EIF3B|EIF3G
Positive	R-HSA-72737	Reactome Gene Sets	Cap-dependent Translation Initiation	-4.677	RPLP2|EIF3B|EIF3G
Positive	GO:0002181	GO Biological Processes	cytoplasmic translation	-4.645	RPLP2|EIF3B|EIF3G
Positive	GO:0022613	GO Biological Processes	ribonucleoprotein complex biogenesis	-4.338	EIF3B|EIF3G|PUF60|LUC7L3
Positive	R-HSA-72766	Reactome Gene Sets	Translation	-3.526	RPLP2|EIF3B|EIF3G
Positive	GO:0006412	GO Biological Processes	translation	-3.225	RPLP2|EIF3B|EIF3G
Positive	GO:0043043	GO Biological Processes	peptide biosynthetic process	-3.136	RPLP2|EIF3B|EIF3G
Positive	GO:0043604	GO Biological Processes	amide biosynthetic process	-2.816	RPLP2|EIF3B|EIF3G
Positive	GO:0006518	GO Biological Processes	peptide metabolic process	-2.773	RPLP2|EIF3B|EIF3G
Positive	GO:0034645	GO Biological Processes	cellular macromolecule biosynthetic process	-2.346	RPLP2|EIF3B|EIF3G
Positive	GO:0043603	GO Biological Processes	cellular amide metabolic process	-2.278	RPLP2|EIF3B|EIF3G
Positive	GO:0000377	GO Biological Processes	RNA splicing, via transesterification reactions with bulged adenosine as nucleophile	-5.247	HNRNPH1|SNRNP70|PUF60|LUC7L3
**Positive**	**GO:0000398**	**GO Biological Processes**	**mRNA splicing, via spliceosome**	**-5.247**	**HNRNPH1|SNRNP70|PUF60|LUC7L3**
Positive	GO:0000375	GO Biological Processes	RNA splicing, via transesterification reactions	-5.222	HNRNPH1|SNRNP70|PUF60|LUC7L3
Positive	GO:0016071	GO Biological Processes	mRNA metabolic process	-5.220	HNRNPH1|SNRNP70|TAF4|PUF60|LUC7L3
Positive	GO:0008380	GO Biological Processes	RNA splicing	-4.620	HNRNPH1|SNRNP70|PUF60|LUC7L3
Positive	CORUM:351	CORUM	Spliceosome	-4.457	SNRNP70|PUF60|LUC7L3
Positive	GO:0006397	GO Biological Processes	mRNA processing	-4.319	HNRNPH1|SNRNP70|PUF60|LUC7L3
Positive	GO:0043484	GO Biological Processes	regulation of RNA splicing	-4.156	HNRNPH1|SNRNP70|PUF60
Positive	R-HSA-72163	Reactome Gene Sets	mRNA Splicing - Major Pathway	-4.113	HNRNPH1|SNRNP70|PUF60
Positive	R-HSA-72172	Reactome Gene Sets	mRNA Splicing	-4.058	HNRNPH1|SNRNP70|PUF60
Positive	R-HSA-72203	Reactome Gene Sets	Processing of Capped Intron-Containing Pre-mRNA	-3.745	HNRNPH1|SNRNP70|PUF60
Positive	R-HSA-8953854	Reactome Gene Sets	Metabolism of RNA	-3.686	HNRNPH1|RPLP2|SNRNP70|PUF60
Positive	GO:0042176	GO Biological Processes	regulation of protein catabolic process	-3.133	ADAM8|SNRNP70|HUWE1
Positive	GO:0051223	GO Biological Processes	regulation of protein transport	-2.856	ADAM8|KLF7|HUWE1
Positive	GO:0070201	GO Biological Processes	regulation of establishment of protein localization	-2.795	ADAM8|KLF7|HUWE1

Negative	GO:0006397	GO Biological Processes	mRNA processing	-16.709	SNU13|SRSF2|SRSF7|SNRPD1|SON|RBM10|PRPF4|EFTUD2|WTAP|PAN2|CLP1|ECD|TARDBP|PRPF31|HTATSF1|PRPF38B|SMU1|WDR33|UBL5|PPP4R2
**Negative**	**GO:0008380**	**GO Biological Processes**	**RNA splicing**	**-15.577**	**SNU13|SRSF2|SRSF7|SNRPD1|SON|RBM10|PRPF4|EFTUD2|WTAP|CLP1|ECD|TARDBP|PRPF31|HTATSF1|PRPF38B|SMU1|UBL5|PPP4R2**
Negative	GO:0016071	GO Biological Processes	mRNA metabolic process	-14.312	SNU13|SRSF2|SRSF7|SNRPD1|SON|RBM10|PRPF4|EFTUD2|WTAP|PAN2|CLP1|ECD|TARDBP|PRPF31|HTATSF1|PRPF38B|SMU1|WDR33|UBL5|PPP4R2
Negative	R-HSA-72163	Reactome Gene Sets	mRNA Splicing - Major Pathway	-10.798	SNU13|PCBP1|SRSF2|SRSF7|SNRPD1|PRPF4|EFTUD2|CLP1|PRPF31|WDR33|CCAR1
Negative	R-HSA-72203	Reactome Gene Sets	Processing of Capped Intron-Containing Pre-mRNA	-10.728	SNU13|PCBP1|SRSF2|SRSF7|SNRPD1|PRPF4|EFTUD2|WTAP|CLP1|PRPF31|WDR33|CCAR1
Negative	R-HSA-72172	Reactome Gene Sets	mRNA Splicing	-10.598	SNU13|PCBP1|SRSF2|SRSF7|SNRPD1|PRPF4|EFTUD2|CLP1|PRPF31|WDR33|CCAR1
Negative	CORUM:351	CORUM	Spliceosome	-10.590	SNU13|SRSF2|SRSF7|SNRPD1|PRPF4|EFTUD2|WTAP|PRPF31|HTATSF1|SMU1
Negative	GO:0000377	GO Biological Processes	RNA splicing, via transesterification reactions with bulged adenosine as nucleophile	-9.053	SNU13|SRSF2|SRSF7|SNRPD1|RBM10|PRPF4|EFTUD2|PRPF31|HTATSF1|SMU1|UBL5
Negative	GO:0000398	GO Biological Processes	mRNA splicing, via spliceosome	-9.053	SNU13|SRSF2|SRSF7|SNRPD1|RBM10|PRPF4|EFTUD2|PRPF31|HTATSF1|SMU1|UBL5
Negative	hsa03040	KEGG Pathway	Spliceosome	-9.006	SNU13|PCBP1|SRSF2|SRSF7|SNRPD1|PRPF4|EFTUD2|PRPF31|PRPF38B
Negative	GO:0000375	GO Biological Processes	RNA splicing, via transesterification reactions	-8.985	SNU13|SRSF2|SRSF7|SNRPD1|RBM10|PRPF4|EFTUD2|PRPF31|HTATSF1|SMU1|UBL5
Negative	R-HSA-8953854	Reactome Gene Sets	Metabolism of RNA	-7.589	SNU13|PCBP1|SRSF2|SRSF7|SNRPD1|PRPF4|EFTUD2|PSMF1|WTAP|PAN2|CLP1|PRPF31|WDR33|CCAR1
Negative	WP411	WikiPathways	mRNA processing	-6.659	SNU13|SRSF2|SRSF7|SNRPD1|PRPF4|EFTUD2|CLP1
Negative	R-HSA-72165	Reactome Gene Sets	mRNA Splicing - Minor Pathway	-6.193	SNU13|SRSF2|SRSF7|SNRPD1|EFTUD2
Negative	GO:0022618	GO Biological Processes	ribonucleoprotein complex assembly	-2.298	SNU13|SNRPD1|CLP1|PRPF31
Negative	GO:0071826	GO Biological Processes	ribonucleoprotein complex subunit organization	-2.243	SNU13|SNRPD1|CLP1|PRPF31
Negative	GO:0043484	GO Biological Processes	regulation of RNA splicing	-8.273	ATXN7|SRSF2|SRSF7|SON|RBM10|WTAP|TAF5L|SMU1|TADA2B
Negative	GO:0048024	GO Biological Processes	regulation of mRNA splicing, via spliceosome	-5.961	SRSF2|SRSF7|SON|RBM10|WTAP|SMU1
Negative	GO:1903311	GO Biological Processes	regulation of mRNA metabolic process	-5.251	SRSF2|SRSF7|SON|RBM10|WTAP|PAN2|TARDBP|SMU1
Negative	GO:0050684	GO Biological Processes	regulation of mRNA processing	-5.223	SRSF2|SRSF7|SON|RBM10|WTAP|SMU1
Negative	GO:0000381	GO Biological Processes	regulation of alternative mRNA splicing, via spliceosome	-4.407	SRSF2|RBM10|WTAP|SMU1
Negative	WP4352	WikiPathways	Ciliary landscape	-5.210	APC|UBE2H|EFTUD2|YPEL5|GID8|XPNPEP3|WDR26

**Negative**	**GO:0043161**	**GO Biological Processes**	**proteasome-mediated ubiquitin-dependent protein catabolic process**	**-3.170**	**APC|UBE2H|PSMF1|FBXL6|GID8|WDR26**
Negative	GO:0010498	GO Biological Processes	proteasomal protein catabolic process	-2.971	APC|UBE2H|PSMF1|FBXL6|GID8|WDR26
Negative	GO:0006511	GO Biological Processes	ubiquitin-dependent protein catabolic process	-2.719	APC|UBE2H|PSMF1|FBXL6|GID8|USP46|WDR26
Negative	GO:0019941	GO Biological Processes	modification-dependent protein catabolic process	-2.676	APC|UBE2H|PSMF1|FBXL6|GID8|USP46|WDR26
Negative	GO:0043632	GO Biological Processes	modification-dependent macromolecule catabolic process	-2.629	APC|UBE2H|PSMF1|FBXL6|GID8|USP46|WDR26
Negative	GO:0051603	GO Biological Processes	proteolysis involved in cellular protein catabolic process	-2.434	APC|UBE2H|PSMF1|FBXL6|GID8|USP46|WDR26
Negative	GO:0044257	GO Biological Processes	cellular protein catabolic process	-2.328	APC|UBE2H|PSMF1|FBXL6|GID8|USP46|WDR26
Negative	GO:0030163	GO Biological Processes	protein catabolic process	-2.142	APC|UBE2H|PSMF1|FBXL6|GID8|USP46|WDR26
Negative	GO:0016578	GO Biological Processes	histone deubiquitination	-4.946	ATXN7|TAF5L|BABAM1|TADA2B
Negative	GO:0070646	GO Biological Processes	protein modification by small protein removal	-4.820	ATXN7|SENP6|TAF5L|BABAM1|USP46|TADA2B
Negative	GO:0080135	GO Biological Processes	regulation of cellular response to stress	-4.762	PPP1R10|PTEN|ATXN7|TSC1|CORO2B|TMED2|PDCD10|TAF5L|BABAM1|TADA2B|PPP4R2
Negative	CORUM:6664	CORUM	STAGA complex, SPT3-linked	-4.550	ATXN7|TAF5L|TADA2B
**Negative**	**R-HSA-5688426**	**Reactome Gene Sets**	**Deubiquitination**	**-4.350**	**APC|FOXK2|PTEN|ATXN7|PSMF1|BABAM1|TADA2B**
Negative	GO:0035521	GO Biological Processes	monoubiquitinated histone deubiquitination	-3.984	ATXN7|TAF5L|TADA2B
Negative	GO:0035522	GO Biological Processes	monoubiquitinated histone H2A deubiquitination	-3.984	ATXN7|TAF5L|TADA2B
Negative	GO:0016579	GO Biological Processes	protein deubiquitination	-3.955	ATXN7|TAF5L|BABAM1|USP46|TADA2B
Negative	GO:0035520	GO Biological Processes	monoubiquitinated protein deubiquitination	-3.774	ATXN7|TAF5L|TADA2B
Negative	GO:2001020	GO Biological Processes	regulation of response to DNA damage stimulus	-3.368	PPP1R10|ATXN7|TAF5L|BABAM1|TADA2B|PPP4R2
Negative	GO:0006282	GO Biological Processes	regulation of DNA repair	-3.267	ATXN7|TAF5L|BABAM1|TADA2B|PPP4R2
Negative	GO:0043966	GO Biological Processes	histone H3 acetylation	-3.022	ATXN7|TAF5L|TADA2B
Negative	GO:0051052	GO Biological Processes	regulation of DNA metabolic process	-2.809	PPP1R10|ATXN7|TAF5L|BABAM1|TICRR|TADA2B|PPP4R2
Negative	GO:0016570	GO Biological Processes	histone modification	-2.775	ATXN7|SETDB1|TAF5L|BABAM1|TADA2B|PPP4R2
Negative	GO:0018205	GO Biological Processes	peptidyl-lysine modification	-2.545	ATXN7|SETDB1|SENP6|TAF5L|TADA2B
Negative	R-HSA-5689880	Reactome Gene Sets	Ub-specific processing proteases	-2.284	PTEN|ATXN7|PSMF1|TADA2B
Negative	GO:0016573	GO Biological Processes	histone acetylation	-2.178	ATXN7|TAF5L|TADA2B
Negative	GO:0018393	GO Biological Processes	internal peptidyl-lysine acetylation	-2.120	ATXN7|TAF5L|TADA2B

Negative	GO:0006475	GO Biological Processes	internal protein amino acid acetylation	-2.101	ATXN7|TAF5L|TADA2B
Negative	GO:0018394	GO Biological Processes	peptidyl-lysine acetylation	-2.092	ATXN7|TAF5L|TADA2B
Negative	R-HSA-72187	Reactome Gene Sets	mRNA 3'-end processing	-4.436	SRSF2|SRSF7|CLP1|WDR33
Negative	R-HSA-73856	Reactome Gene Sets	RNA Polymerase II Transcription Termination	-4.193	SRSF2|SRSF7|CLP1|WDR33
Negative	R-HSA-72202	Reactome Gene Sets	Transport of Mature Transcript to Cytoplasm	-2.622	SRSF2|SRSF7|WDR33
Negative	hsa03015	KEGG Pathway	mRNA surveillance pathway	-2.445	CLP1|TARDBP|WDR33
Negative	GO:0050657	GO Biological Processes	nucleic acid transport	-3.823	KPNB1|SRSF7|TSC1|BICD2|MRPL18
Negative	GO:0050658	GO Biological Processes	RNA transport	-3.823	KPNB1|SRSF7|TSC1|BICD2|MRPL18
Negative	GO:0051236	GO Biological Processes	establishment of RNA localization	-3.798	KPNB1|SRSF7|TSC1|BICD2|MRPL18
Negative	GO:0006403	GO Biological Processes	RNA localization	-3.595	KPNB1|SRSF7|TSC1|BICD2|MRPL18
Negative	GO:0015931	GO Biological Processes	nucleobase-containing compound transport	-3.221	KPNB1|SRSF7|TSC1|BICD2|MRPL18
Negative	GO:0032388	GO Biological Processes	positive regulation of intracellular transport	-3.383	PDCD10|MLC1|ICE1|TARDBP|UBL5
Negative	GO:0090316	GO Biological Processes	positive regulation of intracellular protein transport	-2.820	PDCD10|ICE1|TARDBP|UBL5
Negative	GO:1903829	GO Biological Processes	positive regulation of protein localization	-2.530	APC|CORO2B|PDCD10|ICE1|TARDBP|UBL5
Negative	GO:1904951	GO Biological Processes	positive regulation of establishment of protein localization	-2.527	CORO2B|PDCD10|ICE1|TARDBP|UBL5
Negative	GO:0032386	GO Biological Processes	regulation of intracellular transport	-2.395	PDCD10|MLC1|ICE1|TARDBP|UBL5
Negative	GO:0033157	GO Biological Processes	regulation of intracellular protein transport	-2.256	PDCD10|ICE1|TARDBP|UBL5
Negative	GO:0070201	GO Biological Processes	regulation of establishment of protein localization	-2.218	LLGL1|CORO2B|PDCD10|ICE1|TARDBP|UBL5
Negative	GO:0060341	GO Biological Processes	regulation of cellular localization	-2.073	APC|PTEN|PDCD10|MLC1|ICE1|TARDBP|UBL5
Negative	GO:0001655	GO Biological Processes	urogenital system development	-3.176	FOXA1|NOTCH3|PTEN|SOX11|TSC1|SEC61A1
Negative	GO:0035239	GO Biological Processes	tube morphogenesis	-2.929	KLF5|FOXA1|NOTCH3|PTEN|SOX11|TSC1|TMED2|PDCD10
Negative	GO:0021915	GO Biological Processes	neural tube development	-2.851	FOXA1|SOX11|TSC1|TMED2
Negative	GO:0002065	GO Biological Processes	columnar/cuboidal epithelial cell differentiation	-2.682	KLF5|FOXA1|SOX11
Negative	GO:0060537	GO Biological Processes	muscle tissue development	-2.527	KLF5|NEB|PTEN|SOX11|TSC1
Negative	GO:0001841	GO Biological Processes	neural tube formation	-2.372	SOX11|TSC1|TMED2
Negative	GO:0001838	GO Biological Processes	embryonic epithelial tube formation	-2.168	SOX11|TSC1|TMED2
Negative	GO:0072175	GO Biological Processes	epithelial tube formation	-2.092	SOX11|TSC1|TMED2

Negative	GO:0006325	GO Biological Processes	chromatin organization	-3.152	BPTF|FOXA1|SETDB1|SUPT16H|BABAM1|ALKBH4|TADA2B|TSPY8
Negative	GO:0048608	GO Biological Processes	reproductive structure development	-2.653	BPTF|FOXA1|PTEN|TMED2|DHH|TSPY8
Negative	GO:0061458	GO Biological Processes	reproductive system development	-2.638	BPTF|FOXA1|PTEN|TMED2|DHH|TSPY8
Negative	GO:0006338	GO Biological Processes	chromatin remodeling	-2.601	BPTF|FOXA1|SUPT16H|TADA2B|TSPY8
Negative	M145	Canonical Pathways	PID P53 DOWNSTREAM PATHWAY	-3.028	APC|FOXA1|PTEN|TADA2B
Negative	GO:0045165	GO Biological Processes	cell fate commitment	-2.974	APC|FOXA1|NOTCH3|TEAD3|DHH
Negative	WP2853	WikiPathways	Endoderm differentiation	-2.926	APC|BPTF|FOXA1|CRTC1
Negative	GO:0001708	GO Biological Processes	cell fate specification	-2.622	APC|FOXA1|DHH
Negative	GO:0043068	GO Biological Processes	positive regulation of programmed cell death	-2.129	APC|FCER2|FOXA1|PTEN|RBM10|CCAR1
Negative	GO:0051091	GO Biological Processes	positive regulation of DNA-binding transcription factor activity	-2.005	FOXA1|PTEN|IL18RAP|CRTC1
Negative	GO:0051640	GO Biological Processes	organelle localization	-2.986	KPNB1|LLGL1|TMED2|PDCD10|BICD2|TRAPPC4|HPS6
Negative	GO:0051656	GO Biological Processes	establishment of organelle localization	-2.923	KPNB1|LLGL1|TMED2|PDCD10|BICD2|TRAPPC4
Negative	GO:0007346	GO Biological Processes	regulation of mitotic cell cycle	-2.903	APC|FOXA1|PPP1R10|PTEN|ECD|BABAM1|TICRR
Negative	GO:0010948	GO Biological Processes	negative regulation of cell cycle process	-2.665	APC|PPP1R10|PTEN|BABAM1|TICRR
Negative	GO:1901991	GO Biological Processes	negative regulation of mitotic cell cycle phase transition	-2.611	APC|PTEN|BABAM1|TICRR
Negative	GO:0000278	GO Biological Processes	mitotic cell cycle	-2.519	APC|KPNB1|SRSF2|SON|FBXL6|BABAM1|TICRR
Negative	GO:1901987	GO Biological Processes	regulation of cell cycle phase transition	-2.502	APC|PPP1R10|PTEN|ECD|BABAM1|TICRR
Negative	GO:1901990	GO Biological Processes	regulation of mitotic cell cycle phase transition	-2.273	APC|PTEN|ECD|BABAM1|TICRR
Negative	GO:1903047	GO Biological Processes	mitotic cell cycle process	-2.241	APC|KPNB1|SON|FBXL6|BABAM1|TICRR
Negative	GO:0045930	GO Biological Processes	negative regulation of mitotic cell cycle	-2.236	APC|PTEN|BABAM1|TICRR
Negative	GO:0045786	GO Biological Processes	negative regulation of cell cycle	-2.174	APC|PPP1R10|PTEN|BABAM1|TICRR
Negative	GO:1901988	GO Biological Processes	negative regulation of cell cycle phase transition	-2.165	APC|PTEN|BABAM1|TICRR
Negative	GO:0007093	GO Biological Processes	mitotic cell cycle checkpoint signaling	-2.158	APC|BABAM1|TICRR
Negative	GO:0010564	GO Biological Processes	regulation of cell cycle process	-2.070	APC|PPP1R10|PTEN|ECD|SENP6|BABAM1|TICRR
Negative	GO:0070507	GO Biological Processes	regulation of microtubule cytoskeleton organization	-2.840	APC|ATXN7|BICD2|SENP6
Negative	GO:0051493	GO Biological Processes	regulation of cytoskeleton organization	-2.837	APC|NEB|ATXN7|TSC1|CORO2B|BICD2|SENP6

Negative	GO:0032886	GO Biological Processes	regulation of microtubule-based process	-2.091	APC|ATXN7|BICD2|SENP6
Negative	GO:0033044	GO Biological Processes	regulation of chromosome organization	-2.091	APC|PPP1R10|SETDB1|SENP6
Negative	GO:0031330	GO Biological Processes	negative regulation of cellular catabolic process	-2.788	FOXK2|TSC1|RBM10|PSMF1|TARDBP
Negative	GO:1903312	GO Biological Processes	negative regulation of mRNA metabolic process	-2.483	SRSF7|RBM10|TARDBP
Negative	GO:0009895	GO Biological Processes	negative regulation of catabolic process	-2.417	FOXK2|TSC1|RBM10|PSMF1|TARDBP
Negative	WP4949	WikiPathways	16p11.2 proximal deletion syndrome	-2.746	BPTF|PTEN|PPP4R2
Negative	GO:0031647	GO Biological Processes	regulation of protein stability	-2.457	DAD1|PTEN|TSC1|PDCD10|TARDBP
Negative	GO:0001933	GO Biological Processes	negative regulation of protein phosphorylation	-2.351	APC|PTEN|TMED2|TARDBP|PPP4R2
Negative	GO:0010256	GO Biological Processes	endomembrane system organization	-2.156	PTEN|TMED2|PDCD10|TARDBP|SEC61A1|HPS6
Negative	GO:0042326	GO Biological Processes	negative regulation of phosphorylation	-2.141	APC|PTEN|TMED2|TARDBP|PPP4R2
Negative	WP5087	WikiPathways	Malignant pleural mesothelioma	-2.535	APC|MAX|PTEN|TEAD3|TSC1|SETDB1
Negative	hsa05165	KEGG Pathway	Human papillomavirus infection	-2.400	APC|LLGL1|NOTCH3|PTEN|TSC1
Negative	GO:0042552	GO Biological Processes	myelination	-2.303	PTEN|TSC1|DHH
Negative	GO:0010810	GO Biological Processes	regulation of cell-substrate adhesion	-2.298	NEDD9|PTEN|TSC1|CORO2B
Negative	GO:0007272	GO Biological Processes	ensheathment of neurons	-2.281	PTEN|TSC1|DHH
Negative	GO:0008366	GO Biological Processes	axon ensheathment	-2.281	PTEN|TSC1|DHH
Negative	GO:0030534	GO Biological Processes	adult behavior	-2.039	PTEN|TSC1|USP46
Negative	GO:0010977	GO Biological Processes	negative regulation of neuron projection development	-2.030	PTEN|TSC1|AMIGO3
Negative	GO:1990830	GO Biological Processes	cellular response to leukemia inhibitory factor	-2.420	KLF5|SRSF7|PDCD10
Negative	GO:1990823	GO Biological Processes	response to leukemia inhibitory factor	-2.408	KLF5|SRSF7|PDCD10
Negative	GO:0061640	GO Biological Processes	cytoskeleton-dependent cytokinesis	-2.337	APC|SON|ALKBH4
Negative	GO:0000910	GO Biological Processes	cytokinesis	-2.314	APC|SON|ALKBH4
Negative	GO:0051301	GO Biological Processes	cell division	-2.286	APC|NEDD9|SON|TEAD3|BABAM1|ALKBH4
Negative	GO:0044089	GO Biological Processes	positive regulation of cellular component biogenesis	-2.332	APC|TSC1|SETDB1|PAN2|ICE1|AMIGO3
Negative	GO:0017148	GO Biological Processes	negative regulation of translation	-2.140	ILF3|TSC1|PAN2|TARDBP
Negative	GO:0042752	GO Biological Processes	regulation of circadian rhythm	-2.208	CRTC1|TARDBP|FBXL6
Negative	R-HSA-157118	Reactome Gene Sets	Signaling by NOTCH	-2.177	FCER2|NOTCH3|PSMF1|TMED2

Negative	GO:0006366	GO Biological Processes	transcription by RNA polymerase II	-2.127	DRAP1|SUPT16H|ICE1|TAF5L

Appendix Table S4 sgRNA sequences used in arrayed target validation

**Gene ID**	**Gene symbol**	**Sequence (5' -> 3')**
NonTargetingGuide001	Non-Targeting	CTTATAGCGGCCTTAGCTCA
NonTargetingGuide002	Non-Targeting	CAACCTTATAGCCCCGGACT
NonTargetingGuide003	Non-Targeting	CATGTAGCAGAAGGCGGCTA
ENSG00000169045	HNRNPH1	TTGGCCGTGTCAGGACTATT
ENSG00000169045	HNRNPH1	AATGTCTGATCACAGATACG
ENSG00000169045	HNRNPH1	AGCAAACCAGTCCAGCTACG
ENSG00000102317	RBM3	GGATGGTCGTCAGATCCGTG
ENSG00000102317	RBM3	GGTGGTTATGACCGCTACTC
ENSG00000102317	RBM3	CATGAGAGCCATGAACGGAG
ENSG00000064419	TNPO3	CCTGCCGGATCTGTAACAAC
ENSG00000064419	TNPO3	TAGCAATGAGTCCCGTAAAG
ENSG00000064419	TNPO3	TTGGGAAGTTGGTTTAACTT
ENSG00000132424	PNISR	CCAATGCAGCCCAATCAATC
ENSG00000132424	PNISR	CCCACTGCAAAATTATCGGG
ENSG00000132424	PNISR	GGTGGCATCCATGGCTGATC
ENSG00000086758	HUWE1	GGACCGCTTCGATGGAATAC
ENSG00000086758	HUWE1	CAGCACCACTTGCATATCAG

ENSG00000104852	SNRNP70	TACGGCGCAATGCCACAATA
ENSG00000104852	SNRNP70	AGGTATGGAATAGGGTCACG
ENSG00000104852	SNRNP70	TGAGCATTGGGATCATTGTG
ENSG00000179950	PUF60	ATGCTCTTGATGGGGCCAAA
ENSG00000179950	PUF60	GGGGACCTCATACTCCACGA
ENSG00000179950	PUF60	CAGATGAACTCGGTGATGCT
ENSG00000118263	KLF7	GCAGCAGGGGGTCTAAGCGA
ENSG00000118263	KLF7	GAAGCCTTATAAGTGCTCAT
ENSG00000118263	KLF7	ACCTACAGACGGAGCCCCGG
ENSG00000130699	TAF4	AACACCTATCATTGCACGGC
ENSG00000130699	TAF4	GAGCGCTGCAGGGTTGCACT
ENSG00000130699	TAF4	CTCCTCAGCGCACGGTACCA
ENSG00000130811	EIF3G	TCCACCTGGTCGGCCCAACT
ENSG00000130811	EIF3G	TCACCAGCGAGCTCCTCAAG
ENSG00000130811	EIF3G	GTTTCCGTTGATGACCTCCT
ENSG00000177600	RPLP2	CGTCCACATCAACTCACCCT
ENSG00000177600	RPLP2	TACCTGCTGGCTGCCCTAGG
ENSG00000177600	RPLP2	CTTGGACAGCGTGGGTATCG
ENSG00000106263	EIF3B	TGTACTCAAAGATCGGCCCC
ENSG00000106263	EIF3B	ATTCCGGGTCAACCTCTTTA
ENSG00000106263	EIF3B	CTTGTCAAGCTTGTAGCCGT
ENSG00000141867	BRD4	GATTTCTCAATCTCGTCCCA
ENSG00000141867	BRD4	ATTAAAACGCCTATGGATAT

ENSG00000108848	LUC7L3	GTAGAACGTAGGATCAGACG
ENSG00000108848	LUC7L3	TTGATGGGAAAACAACACAT
ENSG00000108848	LUC7L3	TAGTAGGAGATGCCCAGTCC
ENSG00000151651	ADAM8	GAGAGGGTGAGCTACGTCCT
ENSG00000151651	ADAM8	CTTCTACCAGGGCCACGTAG
ENSG00000151651	ADAM8	CTGATCGAGCCCCTGGATGA
ENSG00000134684	YARS	TTACTGGGGAACGGCAACCA
ENSG00000134684	YARS	TCATGTTATCCAGGTATGCG
ENSG00000134684	YARS	GAGAAGCTCAAGTTCATCAA
ENSG00000120156	TEK	AGGTGTACTTCTAGAATATC
ENSG00000120156	TEK	GTACAGAGATGGTTGCATTC
ENSG00000120156	TEK	CTACTGAGAAATGATCCGTA
ENSG00000131759	RARA	GAAGGTGCGCAAAGCGCACC
ENSG00000131759	RARA	GAGGGTGATCTGGTCGGCGA
ENSG00000131759	RARA	AGATGGATGATGCGGAGACG
ENSG00000165417	GTF2A1	CTGAGGCTGAAAGATATATT
ENSG00000165417	GTF2A1	ACAAGCTCCTGTTATACAGC
ENSG00000165417	GTF2A1	AGTGGATGAACAAGTACTGA
ENSG00000087095	NLK	ACCAGATTCTGGAAGACGTT
ENSG00000087095	NLK	TCTCTTGCAAAAGGGTCTTC
ENSG00000087095	NLK	GTGGAGGTTGGAGTATGTCA
ENSG00000136485	DCAF7	TTGATACGACATGCACCATC
ENSG00000136485	DCAF7	TTTGTCATGGGCGATCAGCT

ENSG00000099139	PCSK5	ACCATAGCAGGACGATTAAA
ENSG00000099139	PCSK5	AGAAGTGGTAGTAGTCCTTC
ENSG00000099139	PCSK5	ATTGCTTTCAACGCCAAGAT
ENSG00000187758	ADH1A	ACAGACTTTCTCTAGAGGCG
ENSG00000187758	ADH1A	GGCCCATGAAGTTCGTATTA
ENSG00000187758	ADH1A	GTCCAGTGTAAGCAATCCTC
ENSG00000120833	SOCS2	TGCTGACGTGTAGAGCGGTT
ENSG00000120833	SOCS2	AGGTGAACAGTGCCGTTCCG
ENSG00000120833	SOCS2	CGCCATTCCCGGAGGGCTCA
ENSG00000196361	ELAVL3	GTTGGTCTTGCTGTCGTCAG
ENSG00000196361	ELAVL3	TCTGTAATTTGAGGCCGTTG
ENSG00000196361	ELAVL3	ATGCTAACCTGTACGTCAGC
ENSG00000171861	MRM3	TTATTCCTTGTGGCGTTACG
ENSG00000171861	MRM3	GCCACATAGACCCGAGTGTC
ENSG00000062038	CDH3	GATGACTTCACTGTGCGGAA
ENSG00000062038	CDH3	GTGTCTTCGTAAGATACGTT
ENSG00000062038	CDH3	CACCATTCTCTGACACAGCG
ENSG00000135750	KCNK1	GAAGTAGAGGACCGGCCTGC
ENSG00000135750	KCNK1	TATTGCCATGTTGGTAGTTC
ENSG00000135750	KCNK1	TTCAGAGAGCTCTATAAGAT
ENSG00000174125	TLR1	AATTTCAAACGTGAAGCTAC
ENSG00000174125	TLR1	GGAATGGAGTACTGCGGAAT
ENSG00000174125	TLR1	ATAGTGGGCACGATTCTTTC

ENSG00000155545	MIER3	ACTTGGGGAACTATTTGCAC
ENSG00000155545	MIER3	TACTGCATGTGATGGTGATA
ENSG00000155545	MIER3	ACCTTGTTGAGACTTCATTA
ENSG00000102241	HTATSF1	GACGGACACTCCCTACGAGT
ENSG00000102241	HTATSF1	GCTACATATCAGGCCAATTA
ENSG00000102241	HTATSF1	TCTGGCATCAGTGGGTTCCG
ENSG00000119801	YPEL5	TCACATCTCGAACCATGTGG
ENSG00000119801	YPEL5	GACAGCCAGCGATATAAGGA
ENSG00000119801	YPEL5	AGCTCACATCTCGAACCATG
ENSG00000108424	KPNB1	GCACATGAAGGAGTCGACAT
ENSG00000108424	KPNB1	ACCAGCTGAGGAATGAGTTC
ENSG00000108424	KPNB1	GCTAGCTTCACATTATTACT
ENSG00000162923	WDR26	GCATGAGGAGATCAACAGTC
ENSG00000162923	WDR26	CATGACATGATTTCGGAATT
ENSG00000162923	WDR26	GAAGTACCTAGAATACCTGG
ENSG00000146457	WTAP	CTTGGGAAGAGGTTCTTCGT
ENSG00000146457	WTAP	CAAGTTGTGCAATACGTCCC
ENSG00000176887	SOX11	GTCTGGGTCGCTCTCGTCCA
ENSG00000122692	SMU1	ATCCTTAGAAAAGCTTAGAC
ENSG00000122692	SMU1	CTTTTTCAGGCATACCCAGA
ENSG00000122692	SMU1	CCTTCAAGTACTGCATAATA
ENSG00000122882	ECD	GCTGTGAATAGGCGCATCAG
ENSG00000122882	ECD	GCAGGTCAATAGGGTCTCGT

ENSG00000122882	ECD	AGAAGGTTCTGCTCAGTACC
ENSG00000129351	ILF3	TTCCTGCTCGTCTATCCAGT
ENSG00000129351	ILF3	CATCACTCCCCGCAGGGTTC
ENSG00000129351	ILF3	TGCCTGTCCAGAACGTCCGG
ENSG00000165699	TSC1	ATGCTGGACTCCCCCATGCT
ENSG00000165699	TSC1	CACCTTGGTGGATTATTACC
ENSG00000165699	TSC1	GACGTCGTTGTCCTCACAAC
ENSG00000100138	SNU13	CTTGGAGCGCACAAACACGT
ENSG00000186591	UBE2H	GCTTGTATTCTTCTGGTCGG
ENSG00000186591	UBE2H	CATCCAGAAATACGCCACGG
ENSG00000161547	SRSF2	CCAAGTCCAGATCCGCACGA
ENSG00000161547	SRSF2	CCTGGACCGCGAACGAGATC
ENSG00000161547	SRSF2	TCACGACAAGCGCGACGCTG
ENSG00000115875	SRSF7	CATTTGGATCAAAGGGACGT
ENSG00000115875	SRSF7	GATACTCTCGCTCACGCAGC
ENSG00000204569	PPP1R10	AGAAGTTCTTTGGGGTCTAT
ENSG00000204569	PPP1R10	AGTGAGCGGTAGATGCTGCA
ENSG00000171862	PTEN	AATCCCATAGCAATAATGTT
ENSG00000171862	PTEN	AACTTGTCTTCCCGTCGTGT
ENSG00000171862	PTEN	CAATTCAGGACCCACACGAC
ENSG00000114209	PDCD10	GGTTTGATGAATTAGTCGGT
ENSG00000114209	PDCD10	ATATCAATACCAGAACCGCA
ENSG00000105618	PRPF31	TCCTGCACATCCTCGATCGC

ENSG00000105618	PRPF31	CAGCTCGTTTTCGATCTCCA
ENSG00000105618	PRPF31	ACTTATCCCGGATGAACTTA
ENSG00000125952	MAX	ATGCACTGGAACGAAAACGT
ENSG00000125952	MAX	CAGGAAGAAGCTCCGGATGG
ENSG00000125952	MAX	CACCATCTCTGCCTTCGATG
ENSG00000171634	BPTF	GTTCCGCAGTACCTCGTAAA
ENSG00000171634	BPTF	TATATTCGACATGAACCTAT
ENSG00000171634	BPTF	GCTATCTGGATTCCGCAATC
ENSG00000169564	PCBP1	GGAGTCAATTCCGGCGAATC
ENSG00000169564	PCBP1	TGGCGCGCGGATCAACATCT
ENSG00000169564	PCBP1	CGCTATGATCATCGACAAGC
ENSG00000135473	PAN2	CTCCTCGTGCAAGTCAAAGT
ENSG00000135473	PAN2	CTACTCTGAATTGCACAGCG
ENSG00000135473	PAN2	GCAGCACTCTACTCGTTGGT
ENSG00000060339	CCAR1	GAGGACAGAAGAATCCGCCA
ENSG00000060339	CCAR1	TGTGTTAAGAGGGTTTGCTG
ENSG00000060339	CCAR1	GTAGACAGGCTAAGGCTTGT
ENSG00000115607	IL18RAP	GTTGCAGGAGAGCGAATTAA
ENSG00000115608	IL18RAP	TAAAATCATCTTGACACAAC
ENSG00000115609	IL18RAP	CTCACAGGATGCATTTGTCT
ENSG00000134884	ARGLU1	TGAACGAGAAGTTCTCCGAA
ENSG00000134884	ARGLU1	AATAGAAGAAAAACTCATCG
ENSG00000134884	ARGLU1	TGAGGAGCTAGAGCGAATAC

ENSG00000163635	ATXN7	AACGGCCAAAGGCGGCTTGC
ENSG00000163635	ATXN7	GGAAGCAACCGTTCTTCCAG
ENSG00000163635	ATXN7	GCATGTCAAAGACCGGGTGC
ENSG00000135801	TAF5L	CATCTGAGTCCACGTACTGC
ENSG00000135801	TAF5L	CTGTGCTCTTCGGACTGTTT
ENSG00000135801	TAF5L	GGACAACTCGGTGCGCGTCT
ENSG00000086598	TMED2	TTGGAGTCATGGTGGACATC
ENSG00000086598	TMED2	AAAGCACGAACAGGAATACA
ENSG00000086598	TMED2	AACAGGAATACATGGAAGTC
ENSG00000108883	EFTUD2	ATCATGACGATGACCACCCT
ENSG00000108883	EFTUD2	CTTTGTGGACATCTCCACCA
ENSG00000108883	EFTUD2	CAAGATTGACCGGCTGATCC
ENSG00000139549	DHH	TAAGGAGCGGGTGAACGCTT
ENSG00000139549	DHH	AACACCGGTGCAAAGTCGCC
ENSG00000139549	DHH	AGTCACTCGTAGGCGCACTC
ENSG00000083312	TNPO1	TAAAGTCTGTTACACCATTT
ENSG00000083312	TNPO1	GATCACAACTATAGCCTCCA
ENSG00000083312	TNPO1	TTTTCACCGATCGAGGACGG
ENSG00000105576	TNPO2	GTCTGGTCAGGACGAAAATC
ENSG00000105576	TNPO2	AATGGTGGCTCGGATGAGCG
ENSG00000105576	TNPO2	GTGCCTTCACGTTGTTCTTG
ENSG00000114030	KPNA1	AGGCGAAAGTTCTCTTTTCC
ENSG00000114030	KPNA1	GCACACCAGGAGTAGTGGCC

ENSG00000114030	KPNA1	CCATGACCCGGAATGCAGTA
ENSG00000182481	KPNA2	AGCCCGGATTATGTTGTCTA
ENSG00000182481	KPNA2	ACCATGCCAATTCGTTCATT
ENSG00000182481	KPNA2	ATCTGCTCAACAGCATCTAT
ENSG00000102753	KPNA3	CATTACAGTTTGAAGCTGCT
ENSG00000102753	KPNA3	AACAACCTGGGTCTGCTCGT
ENSG00000102753	KPNA3	TTACAGCTTGAACTTGTTGC
ENSG00000186432	KPNA4	AATGCTTCAAGTGATAACCA
ENSG00000186432	KPNA4	TTCTTCTATAAGATTGCCTA
ENSG00000186432	KPNA4	TATAGATGGTGATTATAGAG
ENSG00000196911	KPNA5	TGACCCCAGCATTGTACCTC
ENSG00000196911	KPNA5	TAAAGTTGAAATCCATCCAT
ENSG00000196911	KPNA5	ACTTGATCTATTGGTGGATT
ENSG00000025800	KPNA6	CATGAGAAGACTATCGAACA
ENSG00000025800	KPNA6	ACTTCATCTATTGGAGGACT
ENSG00000025800	KPNA6	GGCAATATTCGTTAGAGCCC
ENSG00000185467	KPNA7	GTTCAGCATACCCGCATCAA
ENSG00000185467	KPNA7	CCCCGCTGCTACGTTGCTCA
ENSG00000196497	IPO4	GACTGAACACCCGCTGGCGA
ENSG00000196497	IPO4	CTGCAGGGCCGTCAGGATCA
ENSG00000196497	IPO4	CTCAGCCACCATTTTTCGAA
ENSG00000065150	IPO5	CAAGAGACGTCTTAGGAGAA
ENSG00000065150	IPO5	GATGGCAGTCTGAACATCAG

ENSG00000205339	IPO7	TAGCCAACAAGCACTGTTAT
ENSG00000205339	IPO7	AACATGATTATCCAAGCCGC
ENSG00000205339	IPO7	ACCAATGGACTCCGCTCCTC
ENSG00000133704	IPO8	TCTTCCGAACTATTATCGAC
ENSG00000133704	IPO8	TGAGGATGATAGACCAGAAC
ENSG00000133704	IPO8	CTTTTTGAAAACCTATGCAG
ENSG00000198700	IPO9	AATCTCCACGGCTCGGGAAC
ENSG00000198700	IPO9	GCTGTACCACGGGAAAGATC
ENSG00000198700	IPO9	ATGGCCCCACATCTGACAGT
ENSG00000086200	IPO11	AACATGGAATTGATCGCTAC
ENSG00000086200	IPO11	GAGAAAACTACTCTGCGTGC
ENSG00000086200	IPO11	TGTGCTGTCGAAGATCATCC
ENSG00000117408	IPO13	CAGCCGAGTCAGTACAATCT
ENSG00000117408	IPO13	CGCCTACCCCAGTACCGCAA
ENSG00000117408	IPO13	TGGCATCTGTCGCATCGCTG
ENSG00000047410	TPR	GCCGCAAGCTTTGACACTCT
ENSG00000047410	TPR	GTGGAATTTCTCTTCCATAC
ENSG00000047410	TPR	AAAGAGCAATGAACTAACCC

Appendix Table S5 TPM of RBM3 mRNA upon specified RBM3 regulator KD compared to the control in K562 and HepG2 cells from ENCODE.

Gene (knocked down)	RBM3 TPM	Gene TPM	Experiment ID	Cell Type
HNRNPH1	44.21	472.5	ENCFF039DFP	K562
HNRNPH1	44.93	412.45	ENCFF713MXN	
Control	111.84	447.63	ENCFF616BYI	
Control	126.13	466.88	ENCFF586TGE	
HNRNPH1	32.46	392.48	ENCFF293ODK	HepG2
HNRNPH1	36.26	361.24	ENCFF266YWO	
Control	105.37	650.81	ENCFF053QJC	
Control	106.98	657.38	ENCFF200BWY	
SNRNP70	22.15	43.97	ENCFF367WUN	HepG2
SNRNP70	21.45	29.54	ENCFF058OGQ	
Control	36.55	125.33	ENCFF873KLR	
Control	43.65	159.84	ENCFF311ACC	
PUF60	85.57	32.65	ENCFF682SFK	K562
PUF60	85.62	23.87	ENCFF461EMF	
Control	197.6	75.15	ENCFF585BAL	
Control	196.86	73.91	ENCFF643OYR	
KPNB1	170.59	104.44	ENCFF933IUO	K562
KPNB1	174.8	120.52	ENCFF298FLZ	

Control	179.89	373.34	ENCFF160UOH	
Control	159.96	356.98	ENCFF116ZHL	
KPNB1	101.47	102.87	ENCFF819AUU	HepG2
KPNB1	98.51	107.14	ENCFF628VDB	
Control	86.41	251.29	ENCFF565TSG	
Control	90.62	253.73	ENCFF073IFA	

Appendix Table S6 Analysis of RBM3 transcript alternative splicing in HNRNPH1 KD versus WT i-neurons, K562 and HepG2 cells by rMATS

Cell type	exonStart_0base	exonEnd	upstreamES	upstreamEE	downstreamES	downstreamEE	FDR	IncLevel1	IncLevel2	IncLevelDifference	Note
i-neuron	48575560	48575630	48575167	48575283	48576313	48576419	1	0.942,0.932,0.988,0.969	0.967,0.971,0.952,0.956	-0.004	
i-neuron	48575560	48575667	48574951	48575283	48575814	48576083	1	1.0,0.996,1.0,1.0	1.0,1.0,1.0,1.0	-0.001	
i-neuron	48575560	48575667	48574951	48575283	48576313	48576419	1	0.984,0.978,0.996,0.991	0.99,0.991,0.984,0.986	0	Exon 3 Inclusion (relative to Exon 2 and 4)
i-neuron	48575560	48575667	48574951	48575283	48577025	48577109	1	1.0,1.0,1.0,0.997	1.0,1.0,1.0,1.0	-0.001	Exon 3 Inclusion (relative to Exon 2 and 6)
i-neuron	48575560	48576083	48575167	48575283	48576313	48576419	1	0.912,0.883,0.979,0.948	0.943,0.947,0.913,0.921	-0.001	
i-neuron	48575814	48576083	48575167	48575283	48576313	48576419	1.09E-14	0.46,0.416,0.793,0.592	0.154,0.114,0.075,0.067	0.463	
i-neuron	48575814	48576083	48575560	48575667	48576313	48576419	0	0.022,0.027,0.02,0.022	0.002,0.002,0.002,0.002	0.021	Exon 3a-L Inclusion
i-neuron	48575921	48576083	48575560	48575667	48576313	48576419	0.03201581	0.014,0.016,0.015,0.014	0.001,0.001,0.002,0.001	0.013	Exon 3a-S Inclusion
i-neuron	48576313	48576419	48575167	48575283	48577025	48577109	1	1.0,1.0,1.0,0.724	1.0,1.0,1.0,1.0	-0.069	Exon 4 Inclusion (relative to Exon 2 and 6)
i-neuron	48576313	48576419	48575560	48575667	48577025	48577109	1	1.0,1.0,1.0,0.997	1.0,1.0,1.0,1.0	-0.001	Exon 4 Inclusion (relative to Exon 3 and 6)
i-neuron	48576507	48576604	48575898	48576419	48577025	48577109	1	1.0,1.0,1.0,1.0	1.0,1.0,1.0,1.0	0	Exon 5 Inclusion (relative to Exon 4 and 6)
K562	48575560	48575630	48575167	48575283	48576313	48576419	1	1.0,1.0	1.0,0.98	0.01	
K562	48575560	48575667	48574951	48575283	48575814	48576083	1	1.0,1.0	1.0,0.985	0.008	

K562	48575560	48575667	48574951	48575283	48576313	48576419	1	1.0,1.0	1.0,0.991	0.004	
K562	48575560	48576083	48575167	48575283	48576313	48576419	1	1.0,1.0	1.0,0.98	0.01	
K562	48575814	48576083	48575167	48575283	48576313	48576419	1	1.0,1.0	1.0,0.973	0.014	
K562	48575814	48576083	48575560	48575667	48576313	48576419	6.78E-09	0.127,0.128	0.411,0.293	-0.224	Exon 3a-L Inclusion
K562	48575921	48576083	48575560	48575667	48576313	48576419	1.46E-07	0.112,0.115	0.396,0.264	-0.217	Exon 3a-S Inclusion
HepG2	48575560	48575630	48575167	48575283	48576313	48576419	1	1.0,1.0	0.833,1.0	0.084	
HepG2	48575560	48575667	48574951	48575283	48576313	48576419	1	1.0,1.0	0.899,1.0	0.05	
HepG2	48575560	48576083	48575167	48575283	48576313	48576419	1	1.0,1.0	0.811,1.0	0.095	
HepG2	48575814	48576083	48575560	48575667	48576313	48576419	2.03E-06	0.081,0.114	0.301,0.487	-0.296	Exon 3a-L Inclusion
HepG2	48575921	48576083	48575560	48575667	48576313	48576419	3.44E-08	0.056,0.094	0.294,0.418	-0.281	Exon 3a-S Inclusion

Appendix Table S7 Key resources used in this study

REAGENT or RESOURCE	SOURCE	IDENTIFIER
**Antibodies**		
Rabbit anti-RBM3 antibody	Proteintech	Cat#14363-1-AP
Rabbit anti-GFP antibody	Abcam	Cat#ab290
Mouse anti-Cas9 antibody	Cell Signaling	Cat#14597S
Mouse anti-GAPDH antibody	Santa Cruz	Cat#sc-32233
Rabbit anti-HNRNPH1 antibody	Bethyl	Cat#A300-511A
Mouse anti-FLAG	Sigma	Cat#F1804
Rabbit anti-HNRNPH1 antibody	Abcam	Cat#ab13074
Rabbit anti-HNRNPH1 antibody	ProteinTech	Cat#14774-1-AP
Mouse anti-HNRNPH/F antibody	Santa Cruz	Cat#sc-32310
Rabbit IgG	Santa Cruz	Cat#sc-2027
Mouse IgG	Santa Cruz	Cat#sc-2025
Rabbit anti-SmD3 antibody	Antibodies Online	Cat#ABIN653228
Mouse anti-SmB (Sm proteins) antibody	(Lerner *et al*, 1981)	N/A

Goat anti-rabbit HRP secondary antibody	Biorad	Cat#1706515
Goat anti-mouse HRP secondary antibody	Biorad	Cat#1706516
Donkey anti-rabbit 680LT	LI-COR	Cat#926-68023
Donkey anti-mouse 680LT	LI-COR	Cat#926-68022
Donkey anti-rabbit 800CW	LI-COR	Cat#926-32213
Donkey anti-mouse 800CW	LI-COR	Cat#926-32212
**Bacterial and virus strains**		
5-alpha Competent E. coli	NEB	Cat#C2987
One Shot ccdB Survival 2 T1R Competent Cells	ThermoFisher Scientific	Cat#A10460
Stable Competent E. coli (High Efficiency)	NEB	Cat#C3040I
Lentivirus: pLVPB-gCherry-PGK-BFP-2A-mCherry	This study	N/A
Lentivirus: whole-genome CRISPR sgRNA library	This study	N/A
Lentivirus: sgRNA-expressing lentivirus (each contains one of the sgRNAs listed in Table S1)	This study	N/A
**Chemicals, peptides, and recombinant proteins**		
TeSR-E8 Medium	Stemcell Technologies	Cat#05990
StemFlex Medium	ThermoFisher Scientific	Cat#A3349401
EDTA, pH8.0	ThermoFisher Scientific	Cat#15575020
Vitronectin	ThermoFisher Scientific	Cat#A14700
Cryostor Cs10 Cryopreservation	Merck	Cat#C2874-100ML
StemPro Accutase Cell Dissociation Reagent	ThermoFisher Scientific	Cat#A1110501
0.25% Trypsin-EDTA	Merck	Cat#T4049
Rho-associated protein kinase (ROCK) inhibitor	BD Biosciences	Cat#Y-27632

Geltrex LDEV-Free, hESC-Qualified, Reduced Growth	Life Technologies	Cat#A1413302
DMEM/F12, GlutaMAX Supplement	Thermo Fisher Scientific	Cat#31331028
N-2 Supplement (100X)	Thermo Fisher Scientific	Cat#17502048
MEM Non-Essential Amino Acids Solution (100X)	Thermo Fisher Scientific	Cat# 11140050
2-Mercaptoethanol (50 mM)	Thermo Fisher Scientific	Cat#31350010
Doxycycline hyclate	Merck	Cat#D9891
Neurobasal medium	Thermo Fisher Scientific	Cat#21103049
Neurobasal medium, minus phenol red	Thermo Fisher Scientific	Cat#12348017
B-27 Supplement (50X), minus vitamin A	Thermo Fisher Scientific	Cat#12587010
GlutaMAX Supplement	Thermo Fisher Scientific	Cat# 35050061
Neurotrophin-3 (NT-3)	PeproTech	Cat#450-03
Brain Derived Neurotrophic Factor (BDNF)	PeproTech	Cat#450-02
Penicillin-Streptomycin	Thermo Fisher Scientific	Cat#15140122
HiFi Cas9 nuclease V3	Integrated DNA Technologies	Cat#1081060
Proteinase K, recombinant, PCR grade	Thermo Fisher Scientific	Cat#EO0491
Sodium Acetate, 3M, pH 5.2	Merck	Cat#567422-100ML
Poly-L-lysine	Merck	Cat#P4707
Laminin	Merck	Cat#L2020-1MG
Dithiothreitol (DTT)	Thermo Fisher Scientific	Cat#R0861, 11896744

Tween-20	Merck	Cat#P9416-50ML
RIPA buffer	Merck	Cat#R0278-500ML
DNase 1	Zymo	Cat#E1011-A
Protein G Dynabeads	Life Technologies	Cat#100090
HALT protease inhibitor cocktail	Thermo Fisher Scientific	Cat#1861278
TRIzol	Invitrogen	Cat#15596018
HALT protease and phosphatase inhibitor cocktail	Thermo Fisher Scientific	Cat#78442
cOmplete, EDTA-free Protease Inhibitor Cocktail	Merck/Roche	Cat#11873580001
Cycloheximide	Biovision	Cat#1041-1
Actinomycin D	Merck	Cat#A1410-2MG
SMG1 inhibitor	ProbeChem	Cat#PC-35788
Pyridostatin hydrochloride	Merck	Cat#SML2690-5MG
Donkey serum	Merck	Cat#D9663
Hoechst	Thermo Fisher Scientific	Cat#62249
**Critical commercial assays**		
RNeasy Plus Mini Kit	Qiagen	Cat#74134
Absolutely RNA Minprep Kit	Agilent	Cat#400800
SuperScript IV First-Strand Synthesis System	Thermo Fisher Scientific	Cat#18091050
Q5 High-Fidelity DNA Polymerase	NEB	Cat#M0492L
NEBuilder HiFi DNA Assembly Master Mix	NEB	Cat#E2621L
GoTaq Taq G2 Green Master Mix	Promega	Cat#M7823
Guide-it Long ssDNA Production System, 25 Rxns	Takara Bio	Cat#632644
P3 Primary Cell 4D-Nucleofector Kit L (12 RXN)	Lonza	Cat#V4XP-3012

Alt-R HDR Enhancer, 500 µL	Integrated DNA Technologies	Cat#226687070
Qubit RNA Broad Range Assay Kit	Thermo Fisher Scientific	Cat#Q10211
RNA Screentape	Agilent	Cat#5067-5576
RNA Screentape Ladder	Agilent	Cat#5067-5578
RNA Screentape Buffer	Agilent	Cat#5067-5577
Loading tips, 10 pk	Agilent	Cat#5067-5599
DNA HS D1000 Screentape	Agilent	Cat#5067-5584
DNA HS D1000 Screentape Buffer	Agilent	Cat#5067-5585
DNA HS D1000 Screentape Ladder	Agilent	Cat#5067-5587
TruSeq Stranded mRNA Library Prep	Illumina	Cat#20020594
Qubit RNA BR Assay Kit	Thermo Fisher Scientific	Cat#Q10211
Qubit dsDNA BR Assay Kit	Thermo Fisher Scientific	Cat#Q32853
BCA Protein Assay Kit	Thermo Fisher Scientific	Cat#23225
Immobilon Western Chemiluminescent HRP Substrate	Merck	Cat#WBKLS0500
Lipofectamine LTX Reagent with Plus Reagent	Thermo Fisher Scientific	Cat#A12621
NEBNext Library Quant Kit for Illumina	NEB	Cat#E7630L
AMPure XP Reagent for PCR Purification	Beckman Coulter	Cat#A63881
NextSeq 500/550 High Output Kit v2.5 (75 Cycles)	Illumina	Cat#20024906
Lipofectamine 2000 reagent	Thermo Fisher Scientific	Cat#11668-027
TransIT-LT1	Mirus	Cat#22043217
dNTP Mix	Thermo Fisher Scientific	Cat#R0192

RiboLock RNAse inhibitor	Thermo Fisher Scientific	Cat#EO0381
AffinityScript Multi temperature Multiple Reverse Transcriptase	Agilent	Cat#600107-51
AffinityScript RT buffer	Agilent	Cat#600100-52
DTT	Agilent	Cat#600100-53
QuickChange Lightning Multi Site-Directed Mutagenesis Kit	Agilent	Cat#210513
**Experimental models: Cell lines**		
Human iPSC/iPSC-derived neuron: Bob (wild-type)	(Pawlowski *et al*, 2017)	N/A
Human iPSC/iPSC-derived neuron: Cas9	This study	N/A
Human iPSC/iPSC-derived neuron: GFP-RBM3	This study	N/A
HeLa	ECACC	Cat#93021013
HEK293T (Lenti-X 293T)	Clontech Takara	Cat#632180
**Oligonucleotides**		
Primers used in this study are listed in Table S1	This study	N/A
RBM3-N gRNA#1 - 5’ CUGCCAUGUCCUCUGAAGA 3’	This study	N/A
RBM3-N gRNA#2 - 5’ UUUCCUUCUUCAGAGGACA 3’	This study	N/A
HNRNPH siRNA#1 - 5’ GGAAAUAGCUGAAAAGGCUdTdT 3’	Microsynth	Cat#2288931
HNRNPH siRNA#2 - 5’ GAGAGUACACAUUGAAAUUdTdT 3’	Microsynth	Cat#2276387
Control siRNA	Dharmacon	Cat#D-001810-10-20
**Recombinant DNA**		
Plasmid: pcDNA3-EGFP	Addgene	Cat#13031
Plasmid: GFP-RBM3 repair template	This study	N/A
Plasmid: pLVPB-gCherry-PGK-BFP-2A-mCherry	This study	N/A
Lentiviral sgRNA cloning vector: pLVPB_U6_sgRNAv2fl_shortccdB_PGK_Puro_BFP	(Metzakopian *et al*, 2017)	N/A

Lentiviral sgRNA expression plasmids (all sgRNA sequences listed in Table S1 individually cloned into the cloning vector)	This study	N/A
Lentiviral packaging plasmid: psPax2	Addgene	Cat#12260
Lentiviral packaging plasmid: pMD2.G	Addgene	Cat#12259
Plasmid: RBM3 Minigene	This study	N/A
Plasmid: delGGGG-RBM3 Minigene	This study	N/A
**Software and algorithms**		
Prism (v9.0.2)	GraphPad	https://www.graphpad.com
Design and Analysis Software (v2.6.0)	Thermo Fisher Scientific	N/A
FIJI (v2.1.0)	(Schindelin *et al*, 2012)	https://fiji.sc/
MATLAB (R2020a)	MathWorks	https://www.mathworks.com
MAGeCK RRA	(Li *et al*, 2014)	https://sourceforge.net/projects/mageck/
Bcl2fastq (v2.2.0)	Illumina	https://emea.support.illumina.com/downloads/bcl2fastq-conversion-software-v2-20.html
Metascape (v3.5)	(Zhou *et al*, 2019)	https://metascape.org/
STRING (v11.5)	(Szklarczyk *et al*, 2020)	https://string-db.org/
FlowJo (v10.7.2)	BD Biosciences	https://www.flowjo.com
nf-core/rnaseq pipeline (v3.3)	(Patel *et al*, 2021)	https://nf-co.re/rnaseq
nf-core/clipseq pipeline (v1.0.0)	(Ewels *et al*, 2020)	https://nf-co.re/clipseq
DESeq2 (v3.15)	(Love *et al*, 2014)	https://bioconductor.org/packages/release/bioc/html/DESeq2.html
rmats2sashimiplot (v2.0.4)	(Shen *et al*, 2014)	https://github.com/Xinglab/rmats2sashimiplot
FastQC (v0.11.8)	Babraham Institute	https://www.bioinformatics.babraham.ac.uk/projects/fastqc/
STAR (v2.6.1b)	(Dobin *et al*, 2013)	https://github.com/alexdobin/STAR
SAMtools (v1.9)	(Li *et al*, 2009)	http://www.htslib.org/
rMATS (v4.1.2)	(Shen *et al*, 2014)	https://github.com/Xinglab/rmats-turbo

clipplotr (v1.0.0)	(Chakrabarti *et al*, 2021)	https://github.com/ulelab/clipplotr
QGRS Mapper	(Kikin *et al*, 2006)	https://bioinformatics.ramapo.edu/QGRS/index.php
Other		
MatTek glass-bottom dishes	MatTek	Cat#P35G-1.0-14-C

References

Chakrabarti AM, Capitanchik C, Ule J & Luscombe NM (2021) clipplotr - a comparative visualisation and analysis tool for CLIP data. *bioRxiv*: 2021.09.10.459763

Dobin A, Davis CA, Schlesinger F, Drenkow J, Zaleski C, Jha S, Batut P, Chaisson M & Gingeras TR (2013) STAR: ultrafast universal RNA-seq aligner. *Bioinformatics* 29: 15–21

Ewels PA, Peltzer A, Fillinger S, Patel H, Alneberg J, Wilm A, Garcia MU, Di Tommaso P & Nahnsen S (2020) The nf-core framework for community-curated bioinformatics pipelines. *Nat Biotechnol* 38: 276–278

Kikin O, D'Antonio L & Bagga PS (2006) QGRS Mapper: a web-based server for predicting G-quadruplexes in nucleotide sequences. *Nucleic Acids Res* 34: W676–82

Lerner MR, Boyle JA, Hardin JA & Steitz JA (1981) Two novel classes of small ribonucleoproteins detected by antibodies associated with lupus erythematosus. *Science* 211: 400–402

Li H, Handsaker B, Wysoker A, Fennell T, Ruan J, Homer N, Marth G, Abecasis G, Durbin R & Others (2009) 1000 genome project data processing subgroup. The sequence alignment/map format and SAMtools. *Bioinformatics* 25: 2078–2079

Li W, Xu H, Xiao T, Cong L, Love MI, Zhang F, Irizarry RA, Liu JS, Brown M & Liu XS (2014) MAGeCK enables robust identification of essential genes from genome-scale CRISPR/Cas9 knockout screens. *Genome Biol* 15: 554

Love MI, Huber W & Anders S (2014) Moderated estimation of fold change and dispersion for RNA-seq data with DESeq2. *Genome Biol* 15: 550

Metzakopian E, Strong A, Iyer V, Hodgkins A, Tzelepis K, Antunes L, Friedrich MJ, Kang Q, Davidson T, Lamberth J, *et al* (2017) Enhancing the genome editing toolbox: genome wide CRISPR arrayed libraries. *Sci Rep* 7: 2244

Patel H, Ewels P, Peltzer A, Hammarén R, Botvinnik O, Sturm G, Moreno D, Vemuri P, silviamorins, Pantano L, *et al* (2021) nf-core/rnaseq: nf-core/rnaseq v3.5 - Copper Chameleon Zenodo

Pawlowski M, Ortmann D, Bertero A, Tavares JM, Pedersen RA, Vallier L & Kotter MRN (2017) Inducible and Deterministic Forward Programming of Human Pluripotent Stem Cells into Neurons, Skeletal Myocytes, and Oligodendrocytes. *Stem Cell Reports* 8: 803–812

Schindelin J, Arganda-Carreras I, Frise E, Kaynig V, Longair M, Pietzsch T, Preibisch S, Rueden C, Saalfeld S, Schmid B, *et al* (2012) Fiji: an open-source platform for biological-image analysis. *Nat Methods* 9: 676–682

Shen S, Park JW, Lu Z-X, Lin L, Henry MD, Wu YN, Zhou Q & Xing Y (2014) rMATS: robust and flexible detection of differential alternative splicing from replicate RNA-Seq data. *Proc Natl Acad Sci U S A* 111: E5593–601

Szklarczyk D, Gable AL, Nastou KC, Lyon D, Kirsch R, Pyysalo S, Doncheva NT, Legeay M, Fang T, Bork P, *et al* (2020) The STRING database in 2021: customizable protein–protein networks, and functional characterization of user-uploaded gene/measurement sets. *Nucleic Acids Res* 49: D605–D612

Zhou Y, Zhou B, Pache L, Chang M, Khodabakhshi AH, Tanaseichuk O, Benner C & Chanda SK (2019) Metascape provides a biologist-oriented resource for the analysis of systems-level datasets. *Nat Commun* 10: 1523
