# Supplementary figures and images for: HNRNPH1 regulates the neuroprotective cold‐shock protein RBM3 expression through poison exon exclusion (part 3 of 3)
Source: EMBO J. 2023 May 30;42(14):e113168. doi: 10.15252/embj.2022113168 (PMC10350819; doi:10.15252/embj.2022113168)

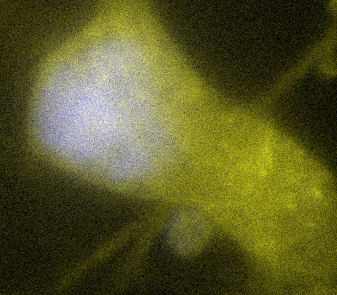

Supplement: Supplementary file 5 — Source Data for Figure 1 [file EMBJ-42-e113168-s008.zip › Figure 1/1A/Images/Replicate 3/32C/32C_S3r6__199.tif]

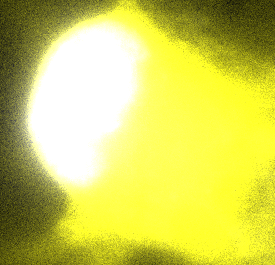

Supplement: Supplementary file 5 — Source Data for Figure 1 [file EMBJ-42-e113168-s008.zip › Figure 1/1A/Images/Replicate 3/32C/32C_S3r6__210.tif]

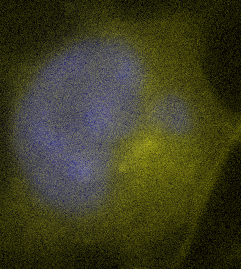

Supplement: Supplementary file 5 — Source Data for Figure 1 [file EMBJ-42-e113168-s008.zip › Figure 1/1A/Images/Replicate 3/32C/32C_S3r6__204.tif]

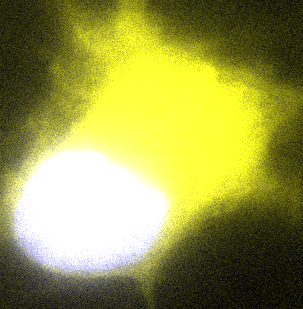

Supplement: Supplementary file 5 — Source Data for Figure 1 [file EMBJ-42-e113168-s008.zip › Figure 1/1A/Images/Replicate 3/32C/32C_S3r6__212.tif]

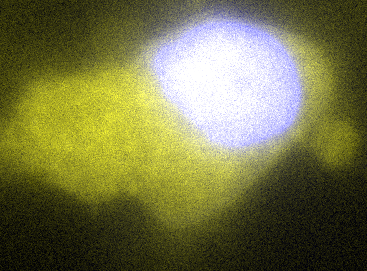

Supplement: Supplementary file 5 — Source Data for Figure 1 [file EMBJ-42-e113168-s008.zip › Figure 1/1A/Images/Replicate 3/32C/32C_S3r6__206.tif]

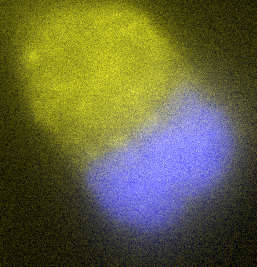

Supplement: Supplementary file 5 — Source Data for Figure 1 [file EMBJ-42-e113168-s008.zip › Figure 1/1A/Images/Replicate 3/32C/32C_S3r1__115.tif]

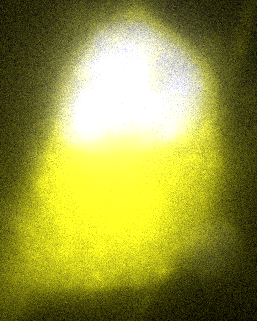

Supplement: Supplementary file 5 — Source Data for Figure 1 [file EMBJ-42-e113168-s008.zip › Figure 1/1A/Images/Replicate 3/32C/32C_S3r1__101.tif]

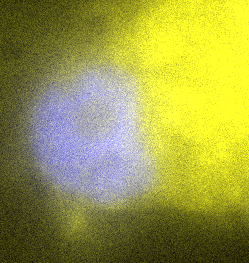

Supplement: Supplementary file 5 — Source Data for Figure 1 [file EMBJ-42-e113168-s008.zip › Figure 1/1A/Images/Replicate 3/32C/32C_S3r1__129.tif]

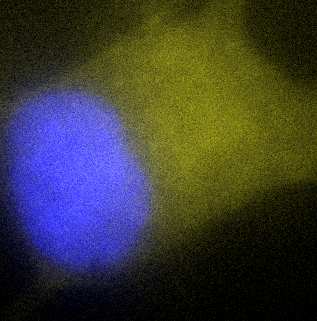

Supplement: Supplementary file 5 — Source Data for Figure 1 [file EMBJ-42-e113168-s008.zip › Figure 1/1A/Images/Replicate 3/32C/32C_S3r3__146.tif]

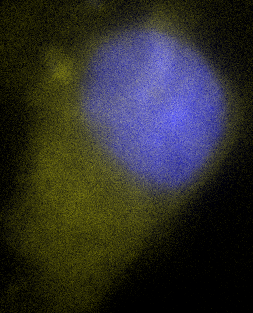

Supplement: Supplementary file 5 — Source Data for Figure 1 [file EMBJ-42-e113168-s008.zip › Figure 1/1A/Images/Replicate 3/32C/32C_S3r3__152.tif]

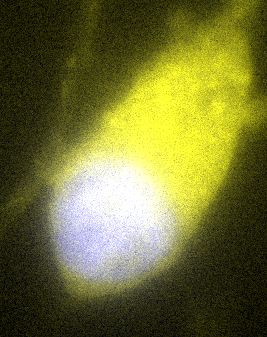

Supplement: Supplementary file 5 — Source Data for Figure 1 [file EMBJ-42-e113168-s008.zip › Figure 1/1A/Images/Replicate 3/32C/32C_S3r3__153.tif]

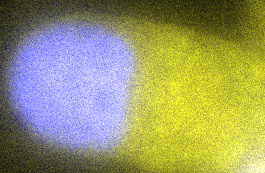

Supplement: Supplementary file 5 — Source Data for Figure 1 [file EMBJ-42-e113168-s008.zip › Figure 1/1A/Images/Replicate 3/32C/32C_S3r3__147.tif]

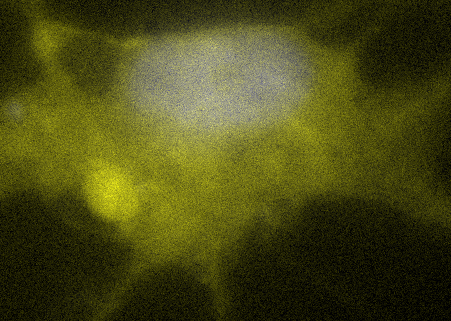

Supplement: Supplementary file 5 — Source Data for Figure 1 [file EMBJ-42-e113168-s008.zip › Figure 1/1A/Images/Replicate 3/32C/32C_S3r1__128.tif]

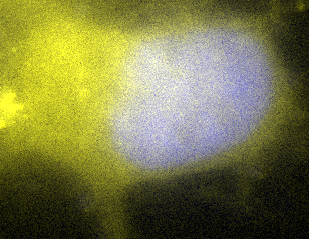

Supplement: Supplementary file 5 — Source Data for Figure 1 [file EMBJ-42-e113168-s008.zip › Figure 1/1A/Images/Replicate 3/32C/32C_S3r1__100.tif]

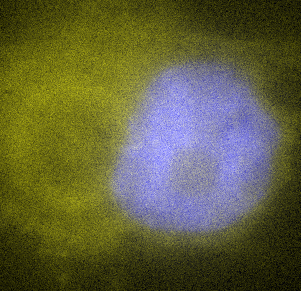

Supplement: Supplementary file 5 — Source Data for Figure 1 [file EMBJ-42-e113168-s008.zip › Figure 1/1A/Images/Replicate 3/32C/32C_S3r1__114.tif]

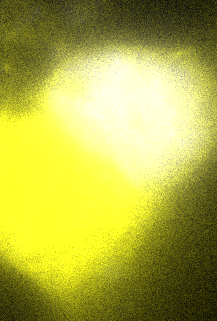

Supplement: Supplementary file 5 — Source Data for Figure 1 [file EMBJ-42-e113168-s008.zip › Figure 1/1A/Images/Replicate 3/32C/32C_S3r6__207.tif]

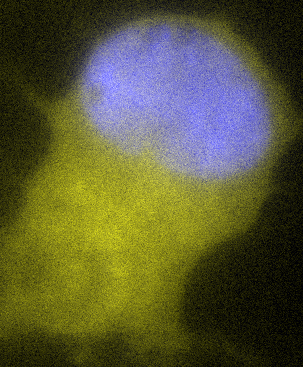

Supplement: Supplementary file 5 — Source Data for Figure 1 [file EMBJ-42-e113168-s008.zip › Figure 1/1A/Images/Replicate 3/32C/32C_S3r6__213.tif]

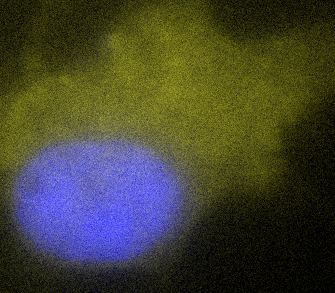

Supplement: Supplementary file 5 — Source Data for Figure 1 [file EMBJ-42-e113168-s008.zip › Figure 1/1A/Images/Replicate 3/32C/32C_S3r6__209.tif]

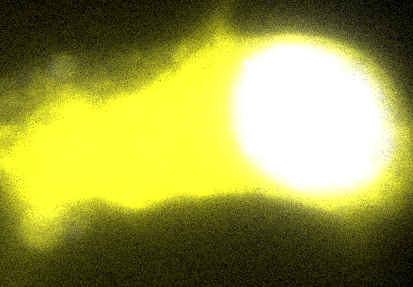

Supplement: Supplementary file 5 — Source Data for Figure 1 [file EMBJ-42-e113168-s008.zip › Figure 1/1A/Images/Replicate 3/32C/32C_S3r3__149.tif]

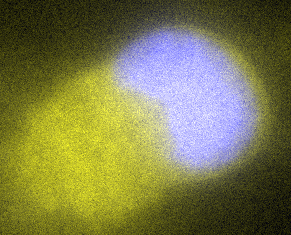

Supplement: Supplementary file 5 — Source Data for Figure 1 [file EMBJ-42-e113168-s008.zip › Figure 1/1A/Images/Replicate 3/32C/32C_S3r3__148.tif]

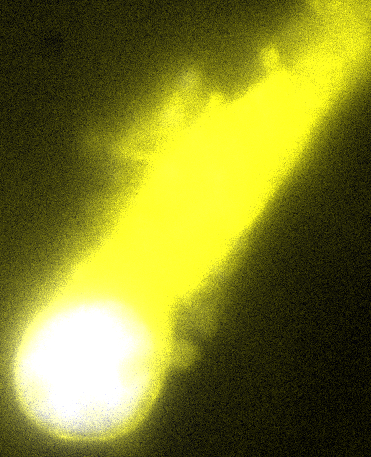

Supplement: Supplementary file 5 — Source Data for Figure 1 [file EMBJ-42-e113168-s008.zip › Figure 1/1A/Images/Replicate 3/32C/32C_S3r6__208.tif]

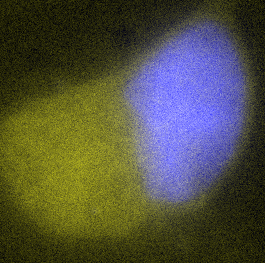

Supplement: Supplementary file 5 — Source Data for Figure 1 [file EMBJ-42-e113168-s008.zip › Figure 1/1A/Images/Replicate 3/32C/32C_S3r6__220.tif]

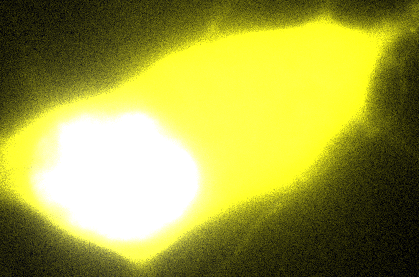

Supplement: Supplementary file 5 — Source Data for Figure 1 [file EMBJ-42-e113168-s008.zip › Figure 1/1A/Images/Replicate 3/32C/32C_S3r6__218.tif]

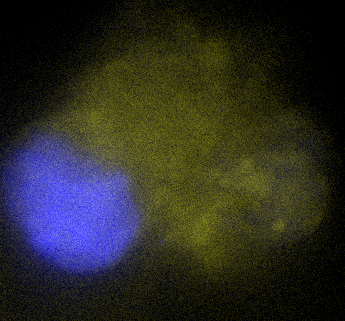

Supplement: Supplementary file 5 — Source Data for Figure 1 [file EMBJ-42-e113168-s008.zip › Figure 1/1A/Images/Replicate 3/32C/32C_S3r6__219.tif]

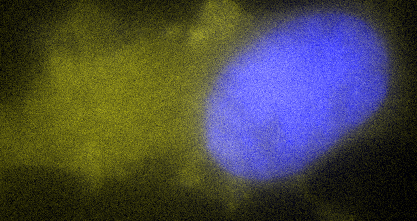

Supplement: Supplementary file 5 — Source Data for Figure 1 [file EMBJ-42-e113168-s008.zip › Figure 1/1A/Images/Replicate 3/32C/32C_S3r1__097.tif]

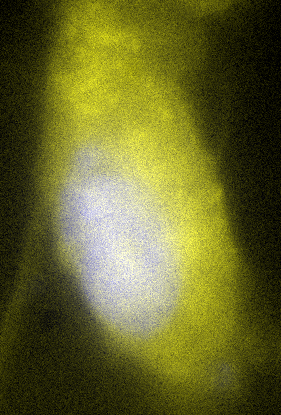

Supplement: Supplementary file 5 — Source Data for Figure 1 [file EMBJ-42-e113168-s008.zip › Figure 1/1A/Images/Replicate 3/32C/32C_S3r1__108.tif]

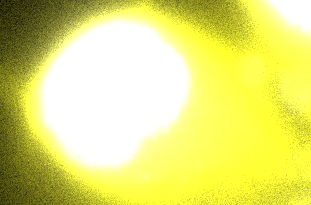

Supplement: Supplementary file 5 — Source Data for Figure 1 [file EMBJ-42-e113168-s008.zip › Figure 1/1A/Images/Replicate 3/32C/32C_S3r1__109.tif]

Set 1

Set 2

Set 3

37°C

32°C

37°C

32°C

32°C

32°C

SMG1 i - - +

- + - +

- +

-

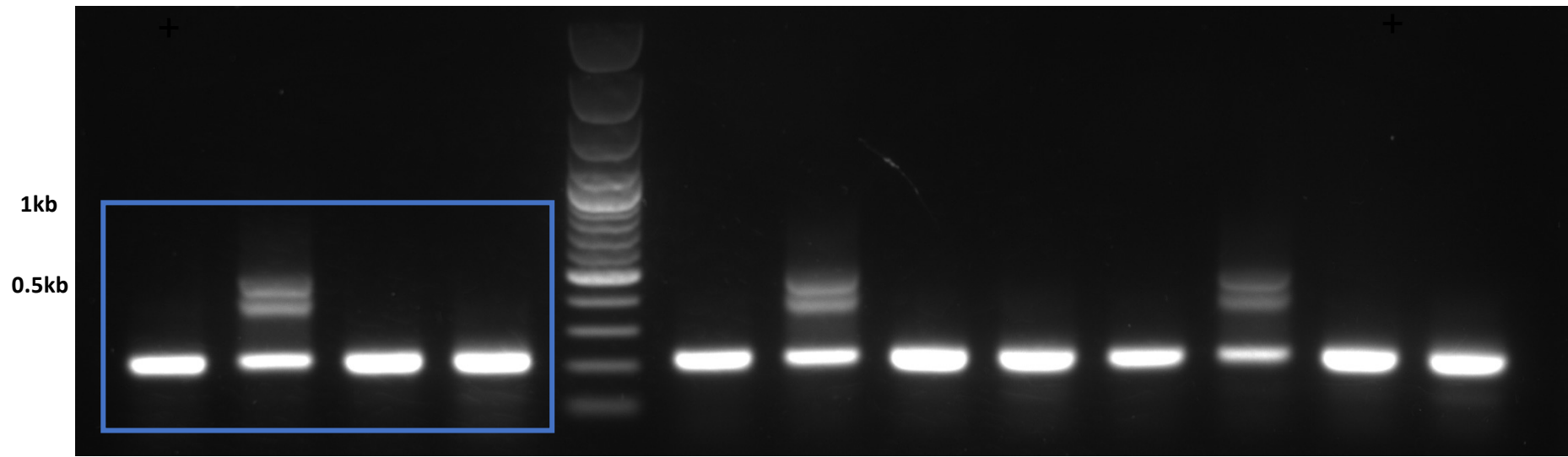

Supplement: Supplementary file 7 — Source Data for Figure 3 [file EMBJ-42-e113168-s006.zip › Figure 3/3D/Labelled RBM3 PE i_neurons_all_3 sets.pdf]

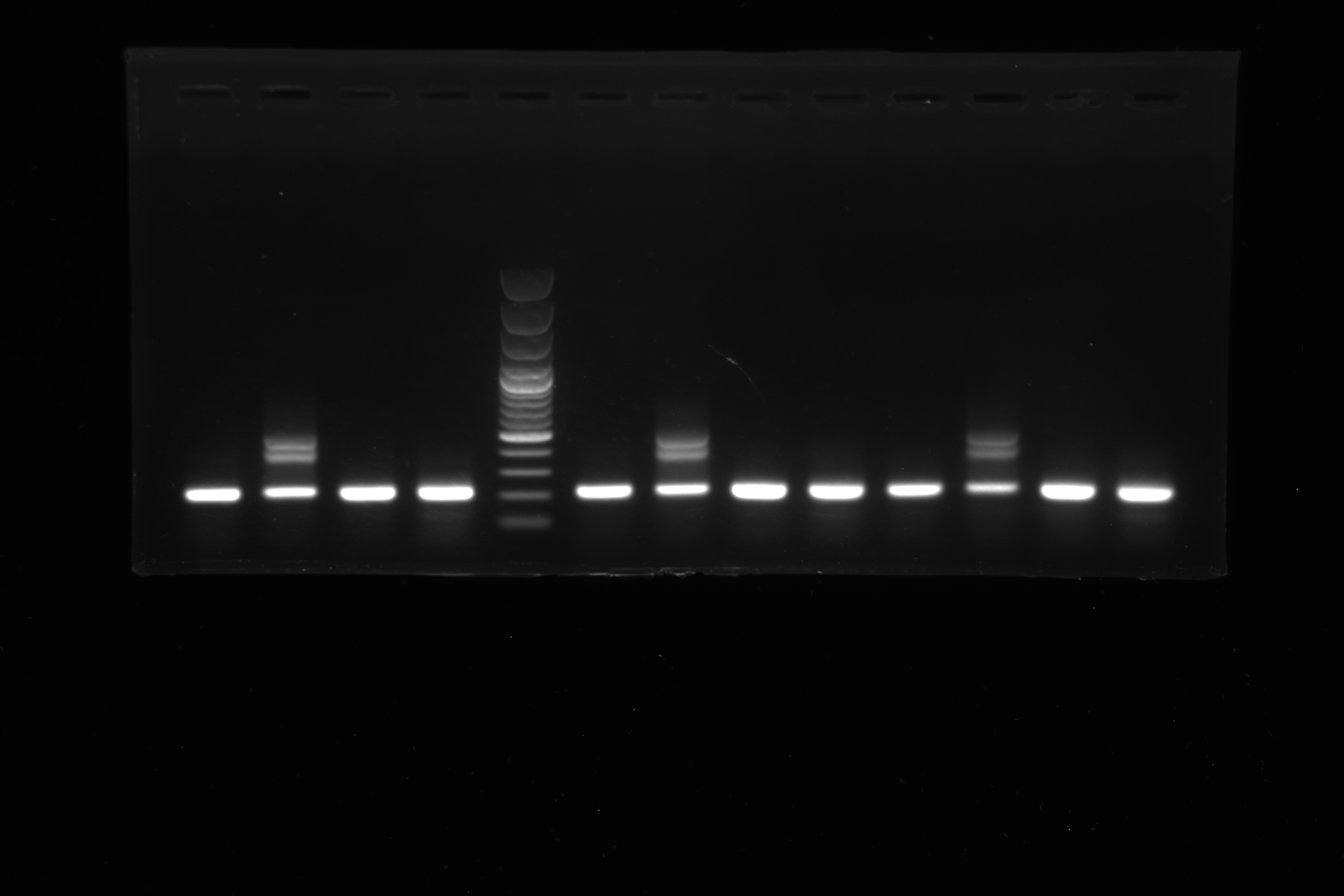

Supplement: Supplementary file 7 — Source Data for Figure 3 [file EMBJ-42-e113168-s006.zip › Figure 3/3D/RBM3 PE i_neurons_all_3 sets.Tif]

## Set 2

37°C

32°C

SMG1 i

-

+

-

+

1kb  
0.5kb

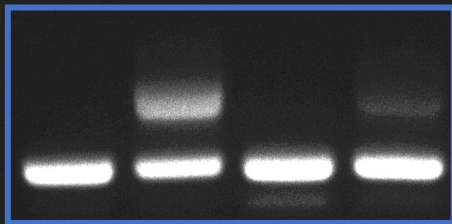

Supplement: Supplementary file 7 — Source Data for Figure 3 [file EMBJ-42-e113168-s006.zip › Figure 3/3F/Labelled Endo RBM3 set 2 Used in the figure.pdf]

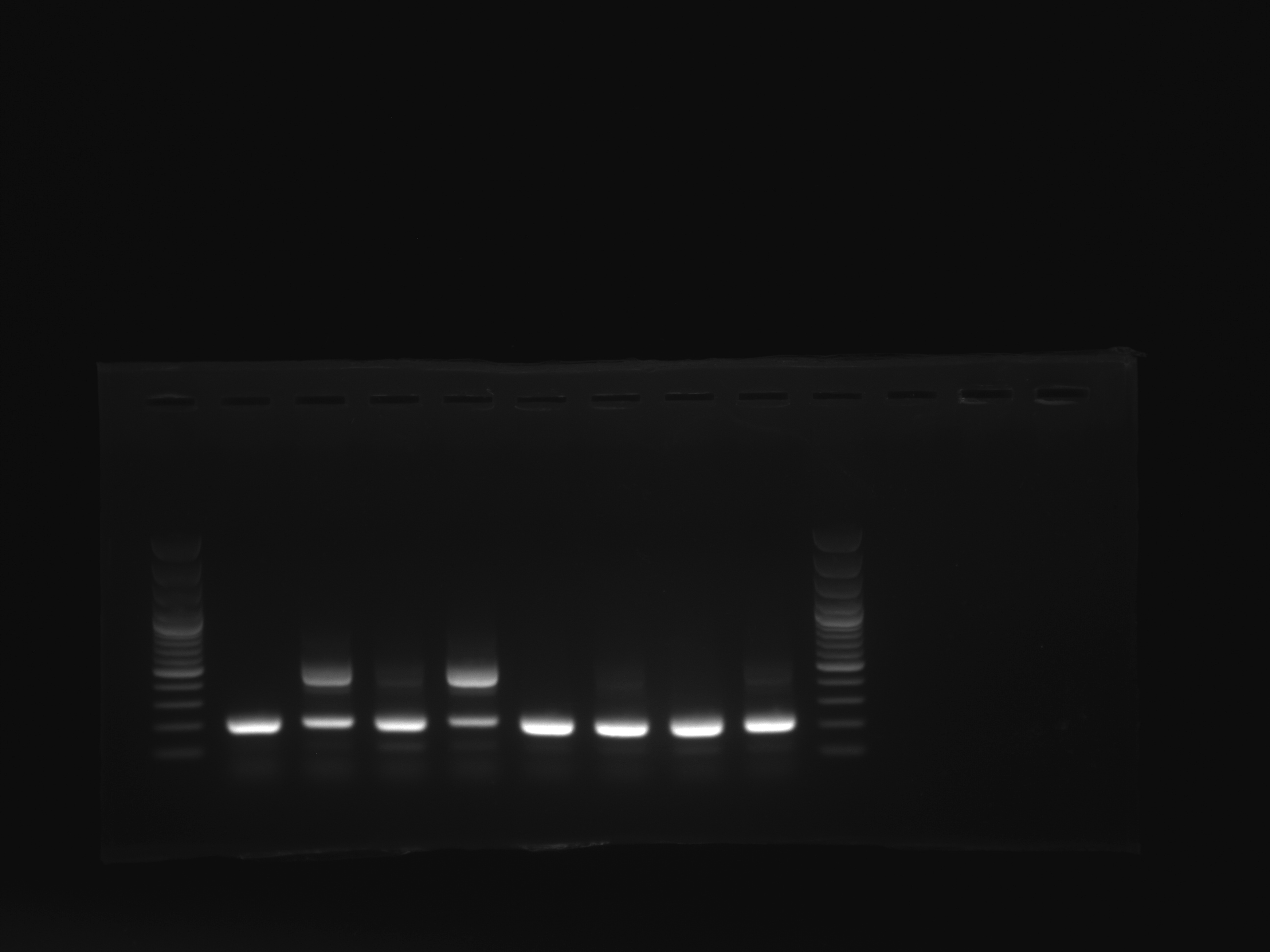

Supplement: Supplementary file 7 — Source Data for Figure 3 [file EMBJ-42-e113168-s006.zip › Figure 3/3F/Endo RBM3 set 3.jpg]

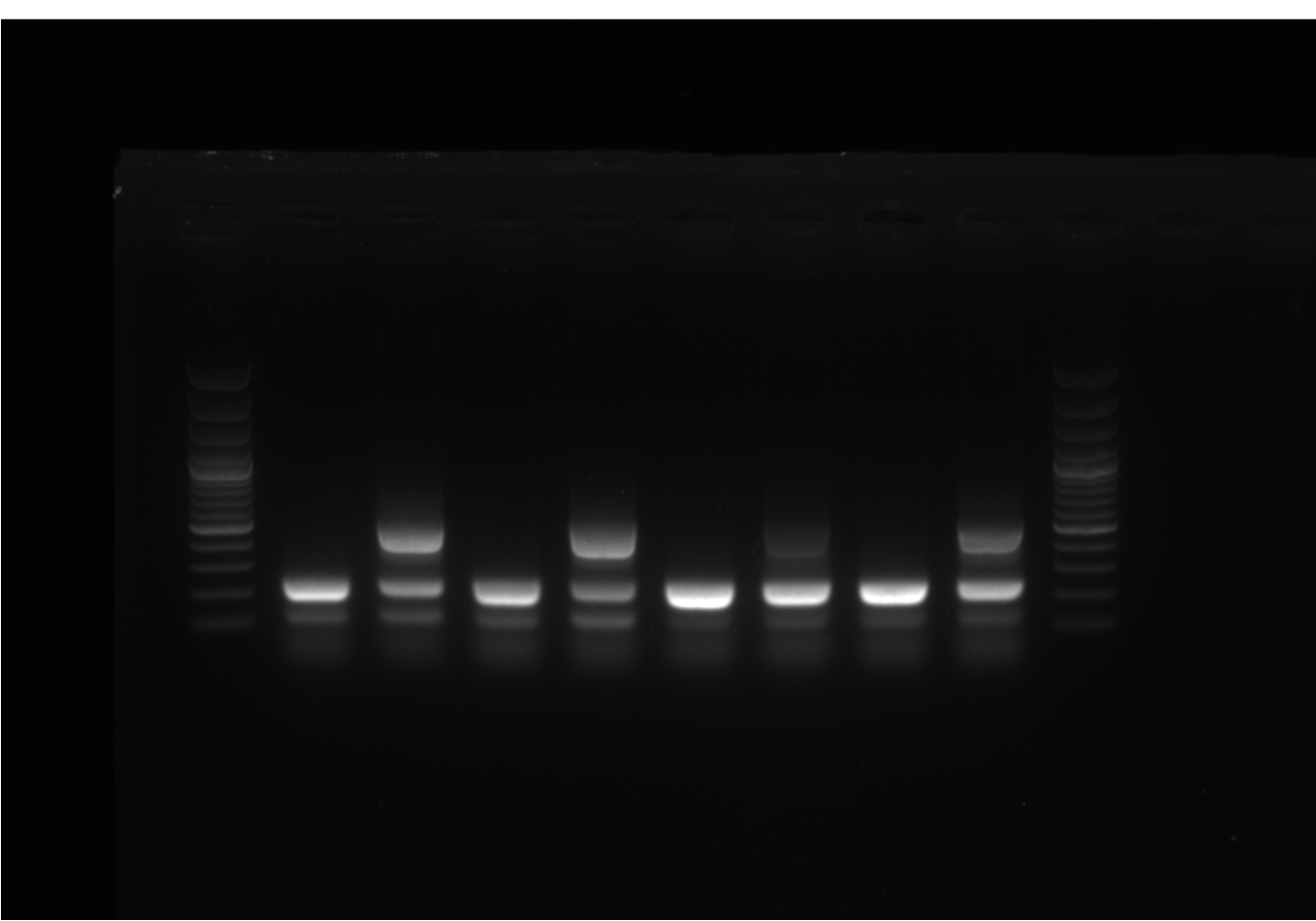

Supplement: Supplementary file 7 — Source Data for Figure 3 [file EMBJ-42-e113168-s006.zip › Figure 3/3F/Endo RBM3 set 1.png]

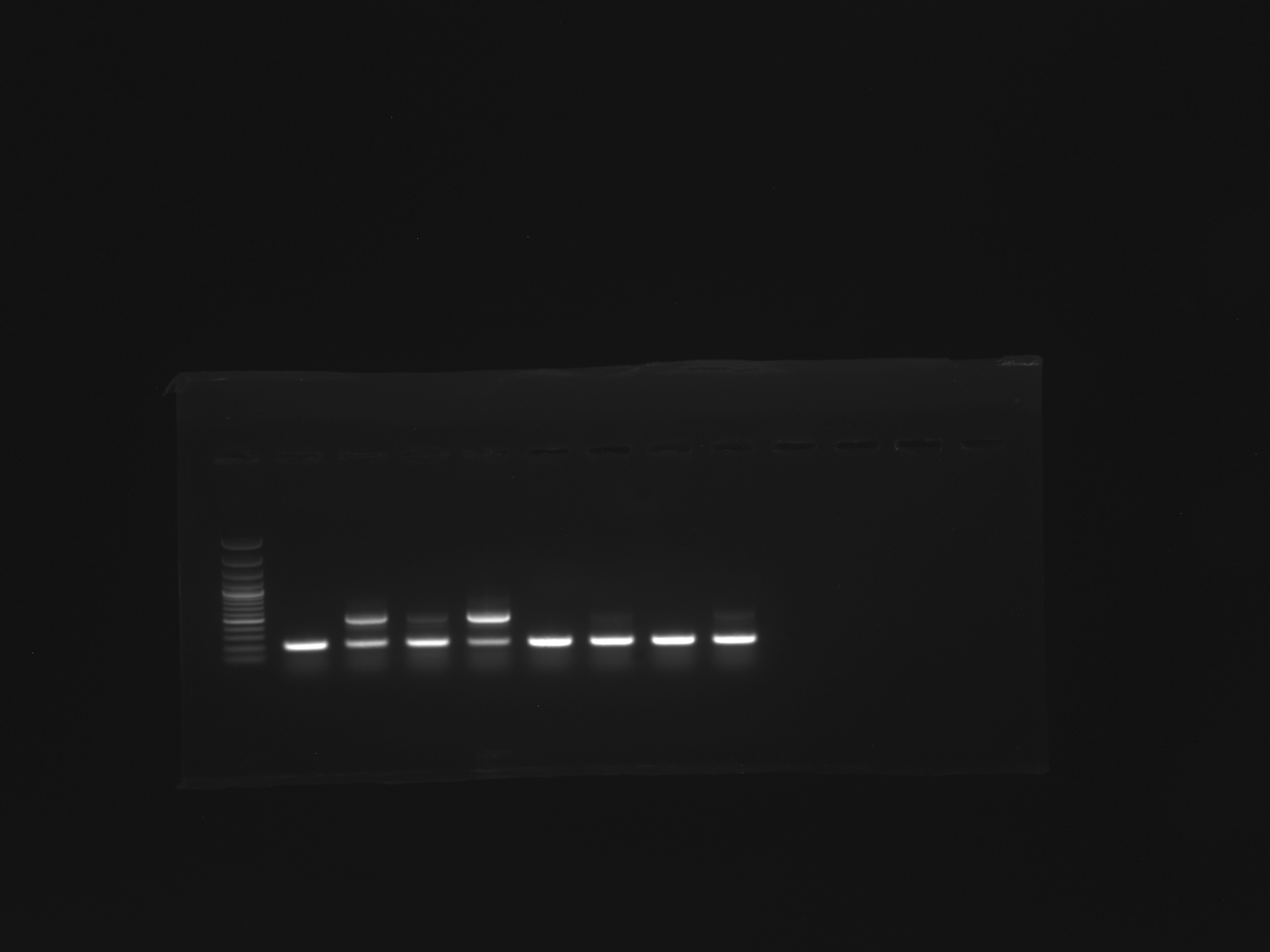

Supplement: Supplementary file 7 — Source Data for Figure 3 [file EMBJ-42-e113168-s006.zip › Figure 3/3F/Endo RBM3 set 4.jpg]

# Set 4

37°C

32°C

SMG1 i

-

+

-

+

1kb

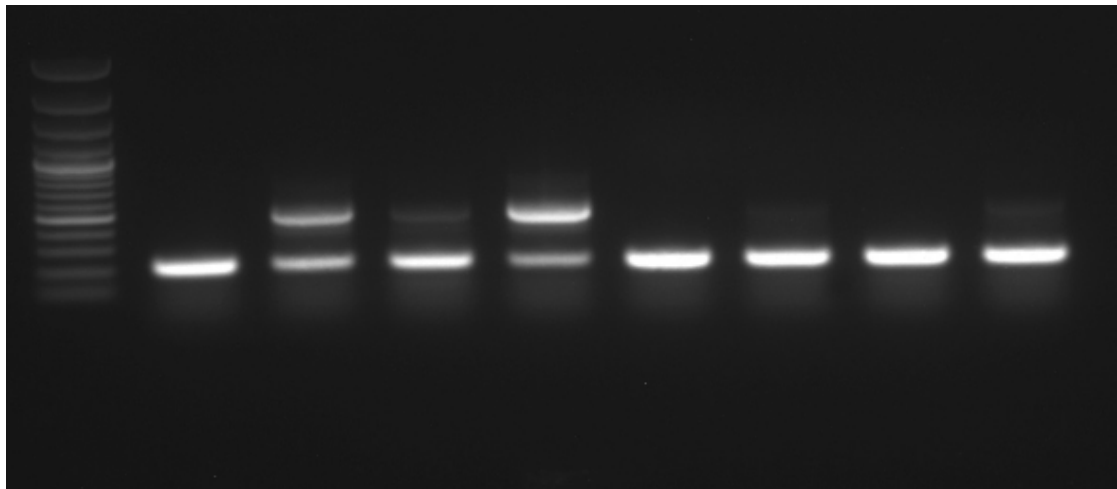

Supplement: Supplementary file 7 — Source Data for Figure 3 [file EMBJ-42-e113168-s006.zip › Figure 3/3F/Labelled Endo RBM3 set 4.pdf]

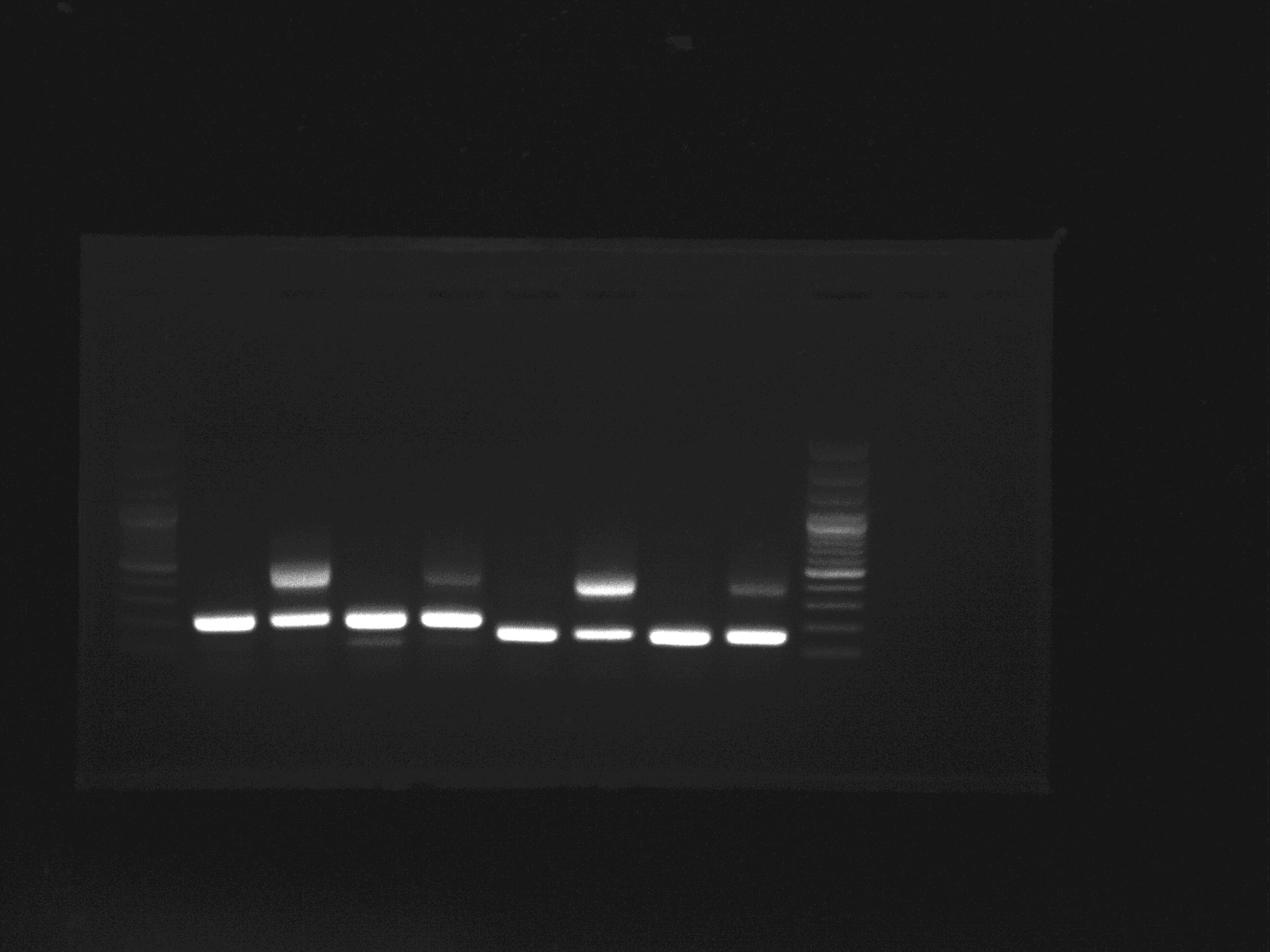

Supplement: Supplementary file 7 — Source Data for Figure 3 [file EMBJ-42-e113168-s006.zip › Figure 3/3F/Endo RBM3 set 2 Used in the figure.jpg]

# Set 3

37°C

32°C

SMG1 i

-

+

-

+

1kb

0.5kb

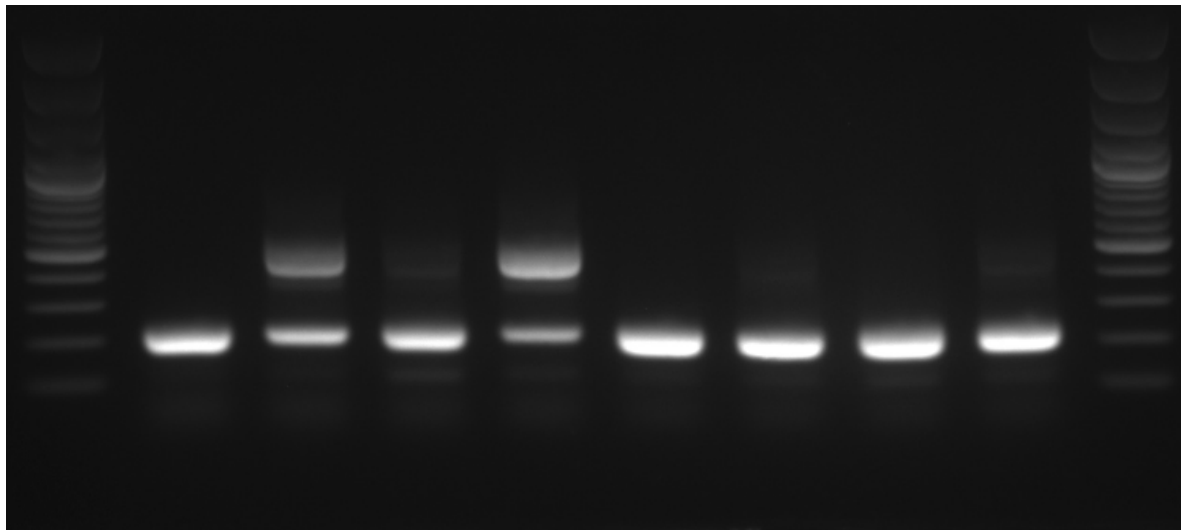

Supplement: Supplementary file 7 — Source Data for Figure 3 [file EMBJ-42-e113168-s006.zip › Figure 3/3F/Labelled Endo RBM3 set 3.pdf]

# Set 1

37°C

32°C

SMG1i

-

+

-

+

1kb

0.5kb

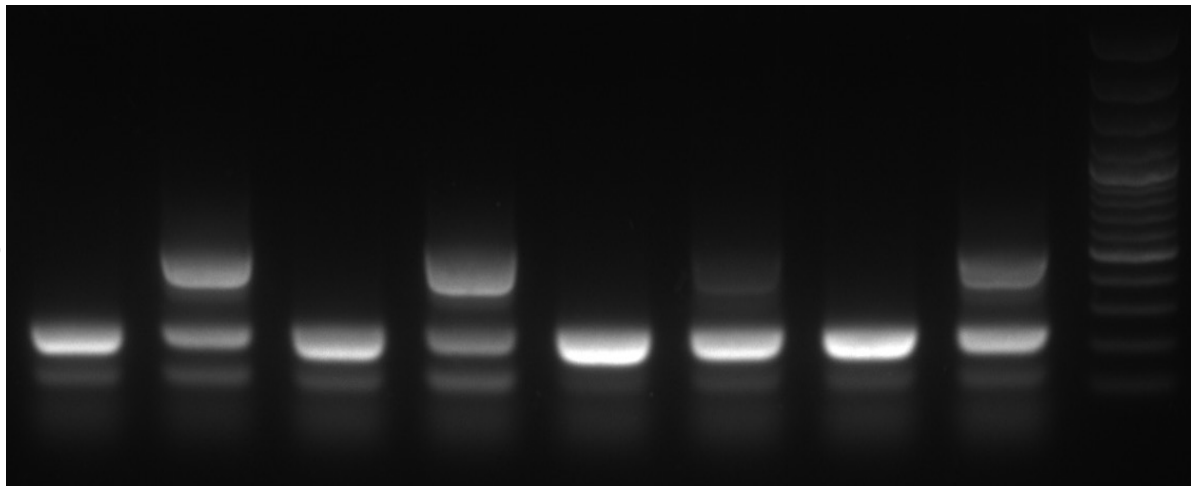

Supplement: Supplementary file 7 — Source Data for Figure 3 [file EMBJ-42-e113168-s006.zip › Figure 3/3F/Labelled Endo RBM3 set 1.pdf]

## Set 2

37°C

32°C

SMG1i

-

+

-

+

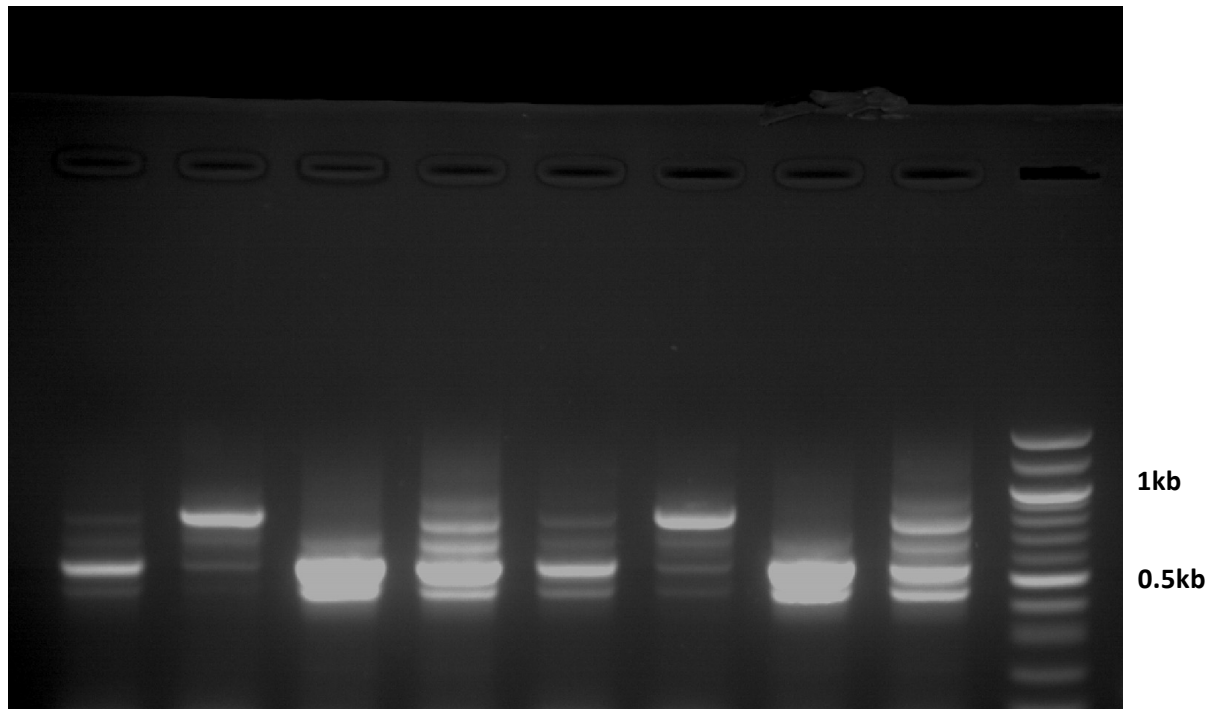

Supplement: Supplementary file 7 — Source Data for Figure 3 [file EMBJ-42-e113168-s006.zip › Figure 3/3G/Labelled RBM3 Minigene WT set 2 HeLa.pdf]

# Set 3

|       | 37°C |   | 32°C |   |
|-------|------|---|------|---|
| SMG1i | -    | + | -    | + |

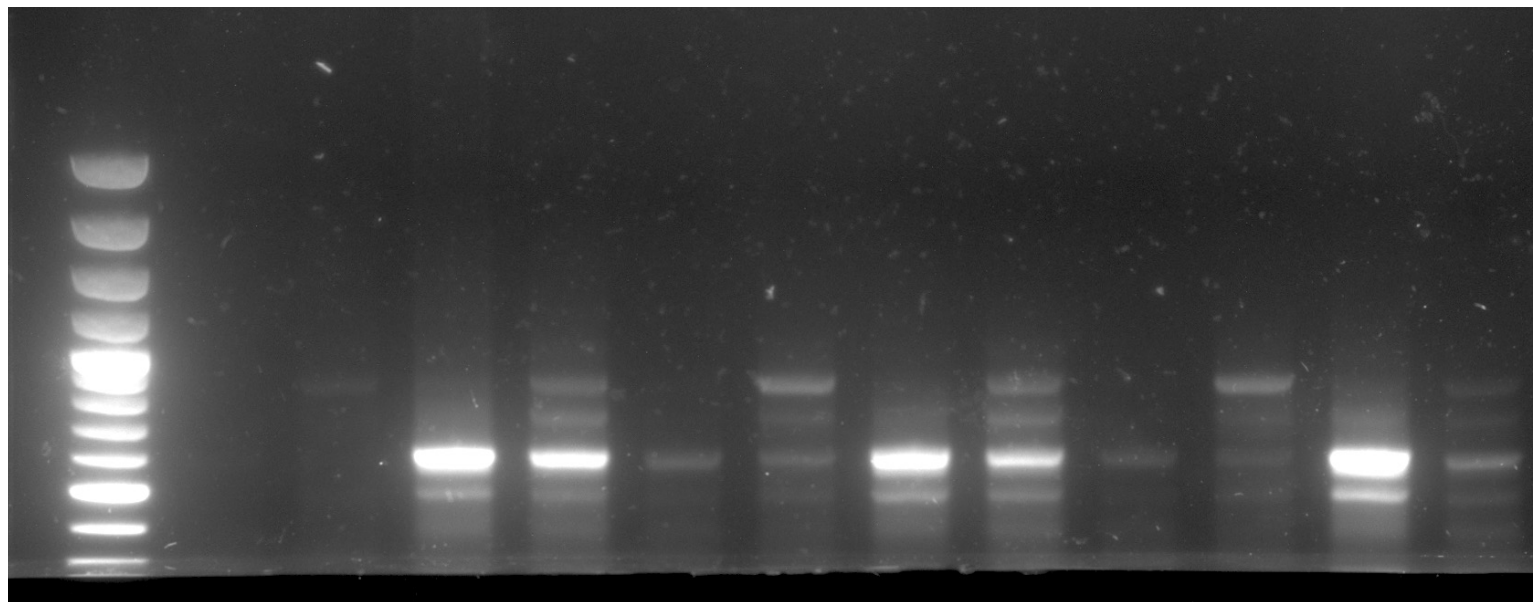

Supplement: Supplementary file 7 — Source Data for Figure 3 [file EMBJ-42-e113168-s006.zip › Figure 3/3G/Labelled RBM3 Minigene WT set 3 HeLa.pdf]

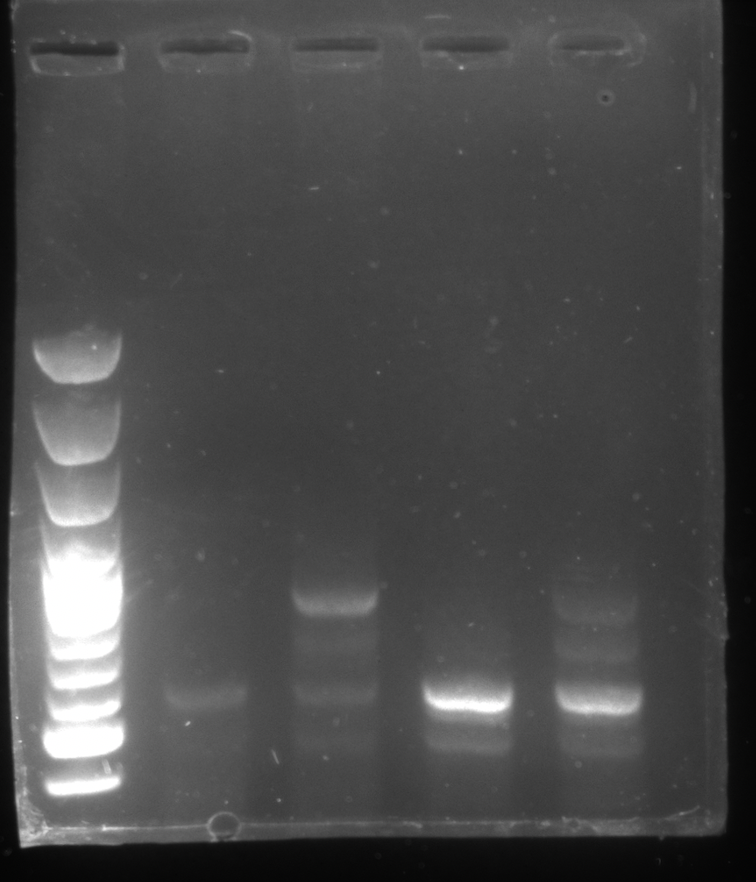

Supplement: Supplementary file 7 — Source Data for Figure 3 [file EMBJ-42-e113168-s006.zip › Figure 3/3G/RBM3 Minigene WT set 1 HeLa Used in the figure.tiff]

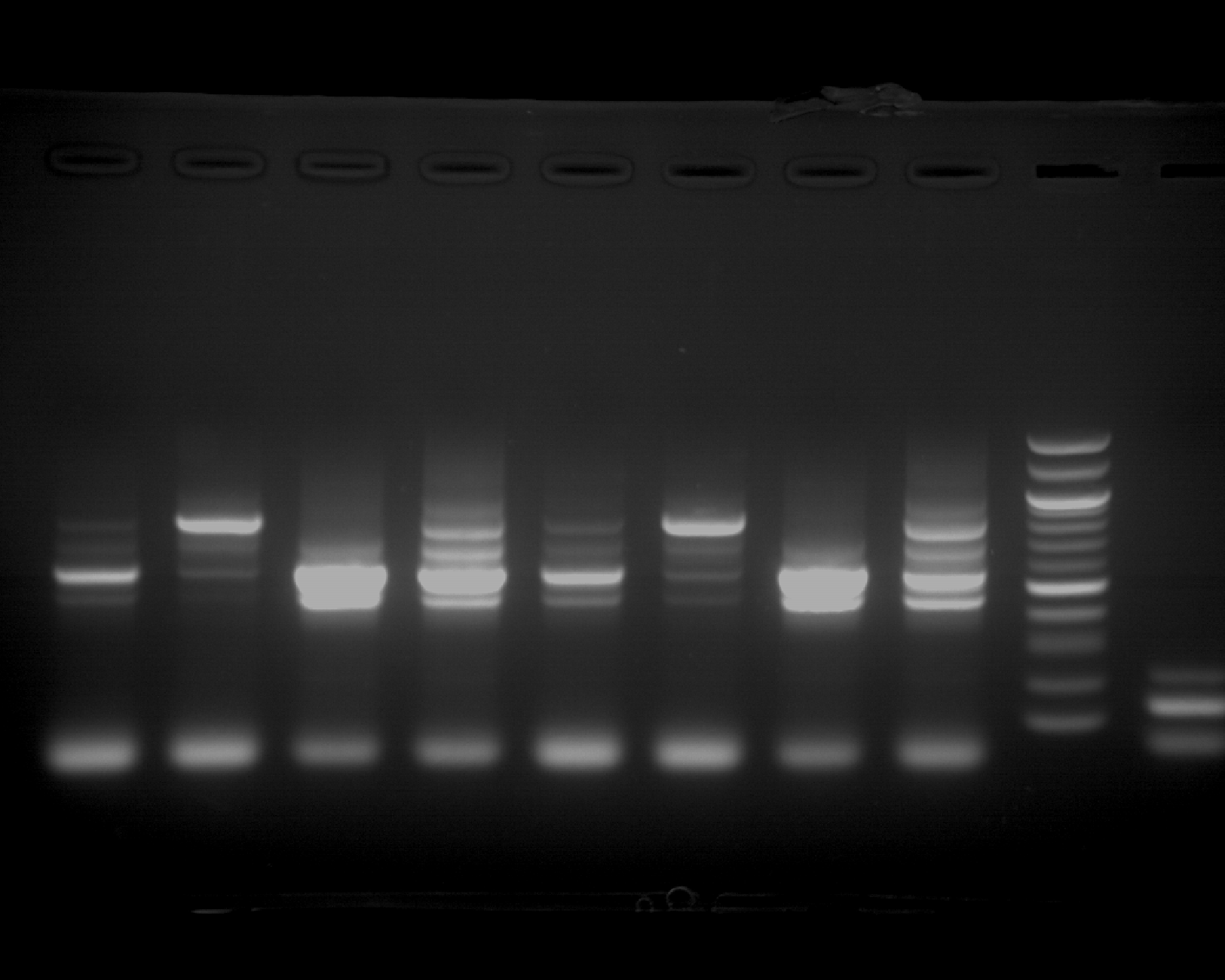

Supplement: Supplementary file 7 — Source Data for Figure 3 [file EMBJ-42-e113168-s006.zip › Figure 3/3G/RBM3 Minigene WT set 2 Hela.tif]

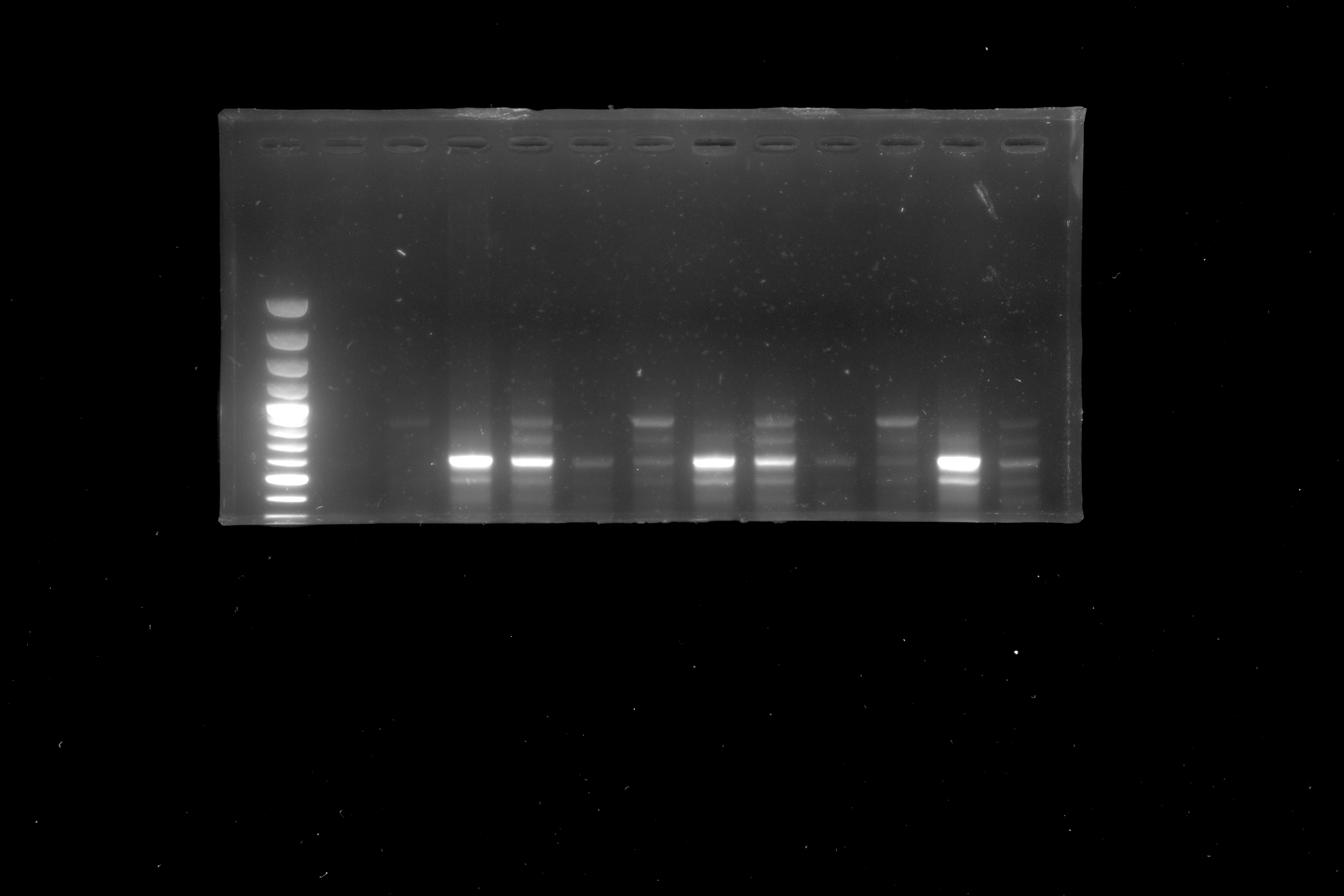

Supplement: Supplementary file 7 — Source Data for Figure 3 [file EMBJ-42-e113168-s006.zip › Figure 3/3G/RBM3 Minigene WT set 3 HeLa.tiff]

# Set 1

37°C

32°C

SMG1i

-

+

-

+

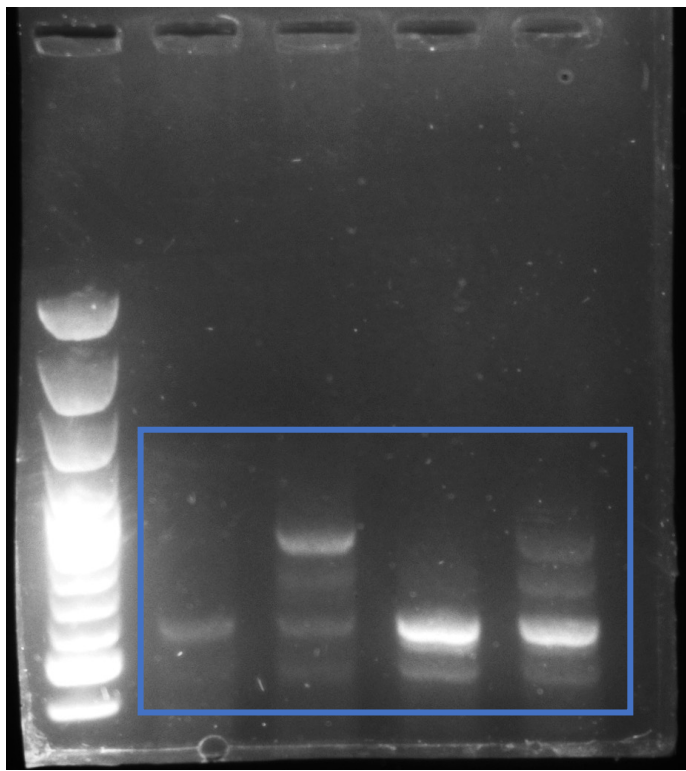

Supplement: Supplementary file 7 — Source Data for Figure 3 [file EMBJ-42-e113168-s006.zip › Figure 3/3G/Labelled RBM3 Minigene WT set 1 HeLa Used in the figure.pdf]

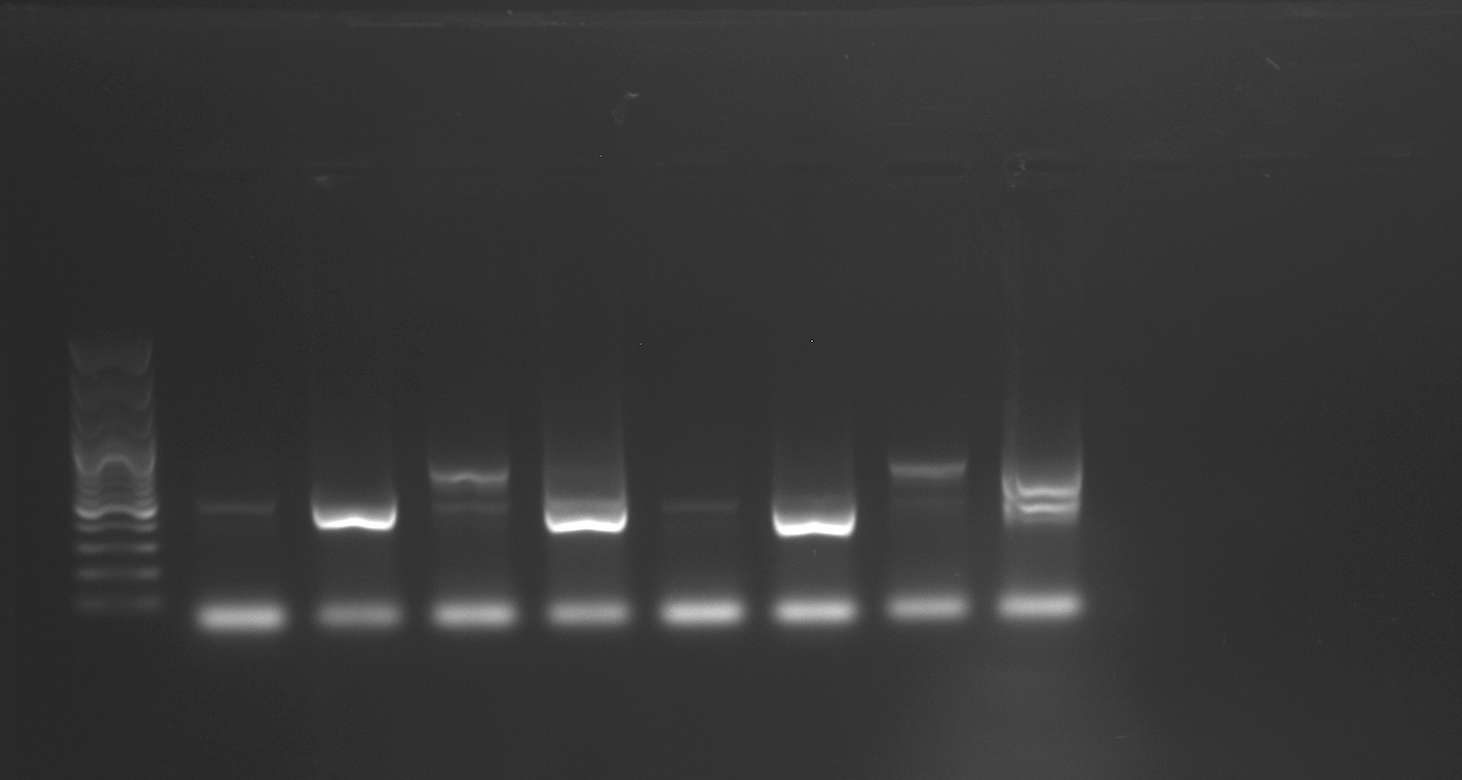

Supplement: Supplementary file 8 — Source Data for Figure 4 [file EMBJ-42-e113168-s010.zip › Figure 4/4E/HNRNPH KD RBM3 Minigene set 2.tiff]

# Set 1

37 °C      32 °C

Control KD      HNRNPH1 KD      Control KD      HNRNPH1 KD

SMG1i      -      +      -      +      -      +      -      +

1kb

0.5kb

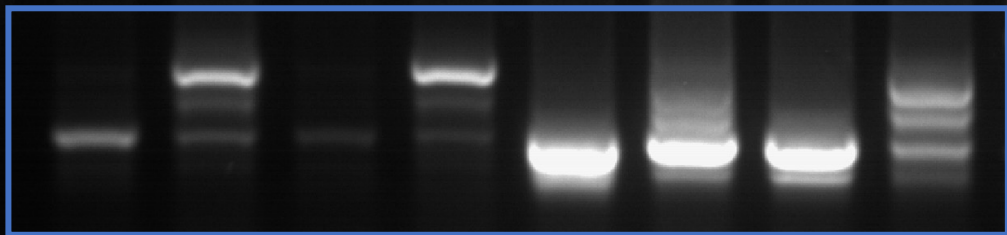

Supplement: Supplementary file 8 — Source Data for Figure 4 [file EMBJ-42-e113168-s010.zip › Figure 4/4E/Labelled HNRNPH KD RBM3 Minigene set 1 Used in the figure.pdf]

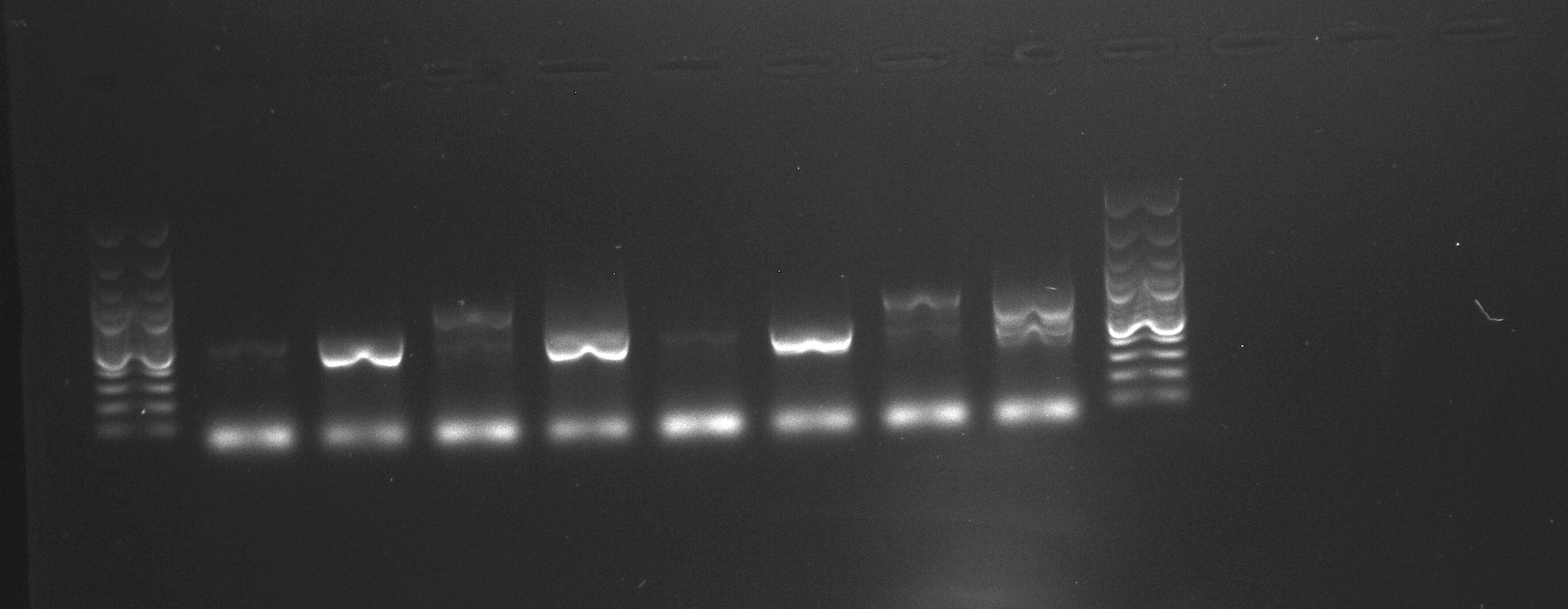

Supplement: Supplementary file 8 — Source Data for Figure 4 [file EMBJ-42-e113168-s010.zip › Figure 4/4E/HNRNPH KD RBM3 Minigene set 3.tiff]

# Set 3

Control KD

HNRNPH

~~KD~~

37 32

37 32

37 32

37 32

SMG1i

-

-

+

-

-

+

1kb  
0.5kb

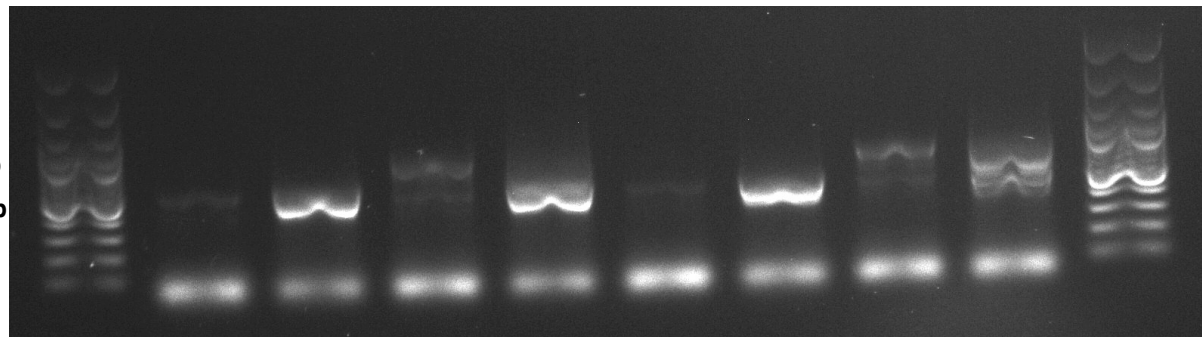

Supplement: Supplementary file 8 — Source Data for Figure 4 [file EMBJ-42-e113168-s010.zip › Figure 4/4E/Labelled HNRNPH KD RBM3 Minigene set 3.pdf]

## Set 2

Control KD

HNRNPH

~~KD~~

37

32

37

32

37

32

37

32

SMG1i

-

-

+

-

-

+

1kb

0.5kb

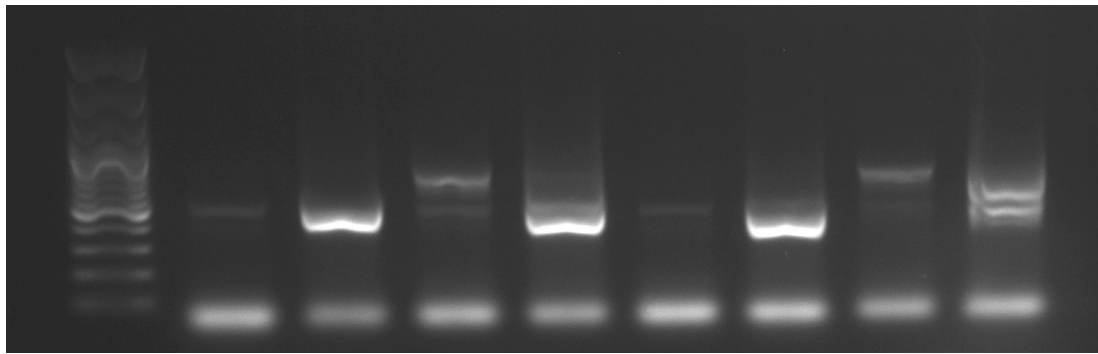

Supplement: Supplementary file 8 — Source Data for Figure 4 [file EMBJ-42-e113168-s010.zip › Figure 4/4E/Labelled HNRNPH KD RBM3 Minigene set 2.pdf]

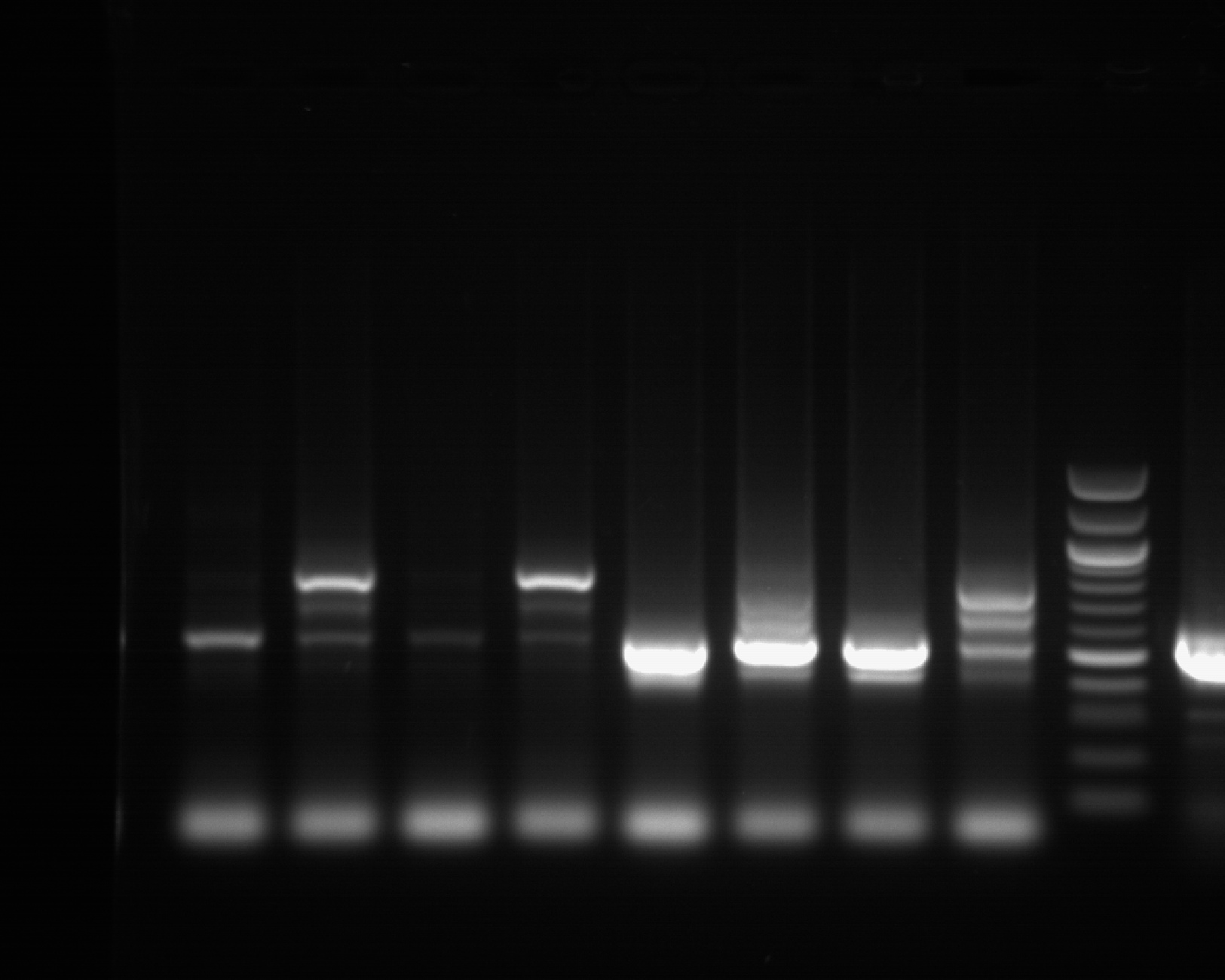

Supplement: Supplementary file 8 — Source Data for Figure 4 [file EMBJ-42-e113168-s010.zip › Figure 4/4E/HNRNPH KD RBM3 Minigene set 1 Used in the figure.tiff]

## Set 2

|       | <u>Control KD</u> |    |    |    | <u><del>HNRNPH</del><br/>KD</u> |    |    |    |
|-------|-------------------|----|----|----|---------------------------------|----|----|----|
|       | 37                | 32 | 37 | 32 | 37                              | 32 | 37 | 32 |
| SMG1i | -                 | -  | +  |    | -                               | -  | +  |    |
|       |                   |    | +  |    |                                 |    | +  |    |

1kb  
0.5kb

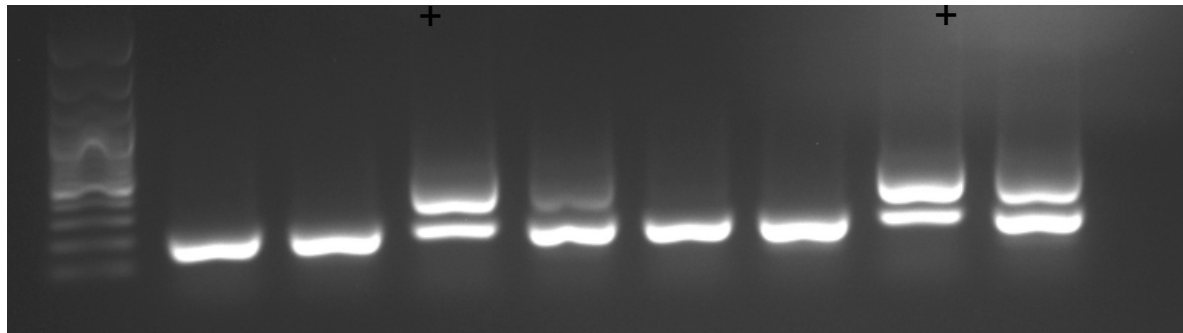

Supplement: Supplementary file 8 — Source Data for Figure 4 [file EMBJ-42-e113168-s010.zip › Figure 4/4D/Labelled HNRNPH KD Endo RBM3 set 2.pdf]

# Set 3

|       | Control KD |    |    |    | <del>HNRNPH</del><br>KD |    |    |    |
|-------|------------|----|----|----|-------------------------|----|----|----|
|       | 37         | 32 | 37 | 32 | 37                      | 32 | 37 | 32 |
| SMG1i | -          | -  | +  |    | -                       | -  | +  |    |
|       |            |    | +  |    |                         |    | +  |    |

1kb  
0.5kb

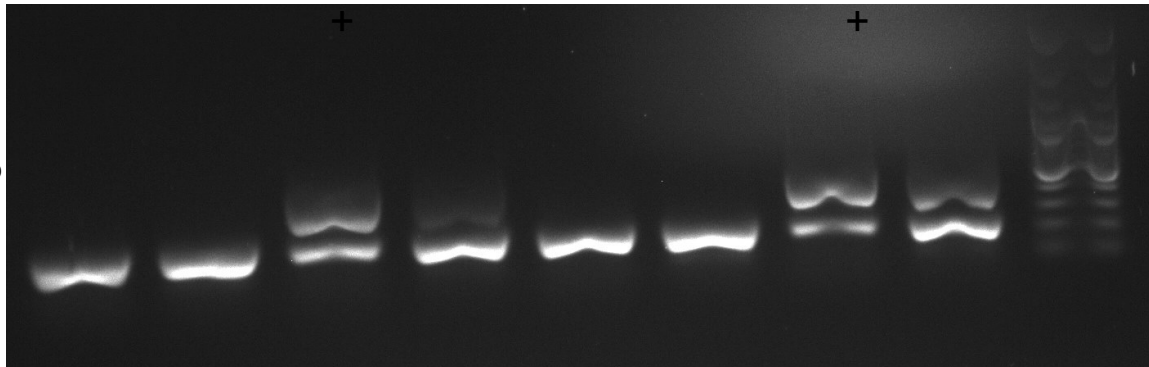

Supplement: Supplementary file 8 — Source Data for Figure 4 [file EMBJ-42-e113168-s010.zip › Figure 4/4D/Labelled HNRNPH KD Endo RBM3 set 3.pdf]

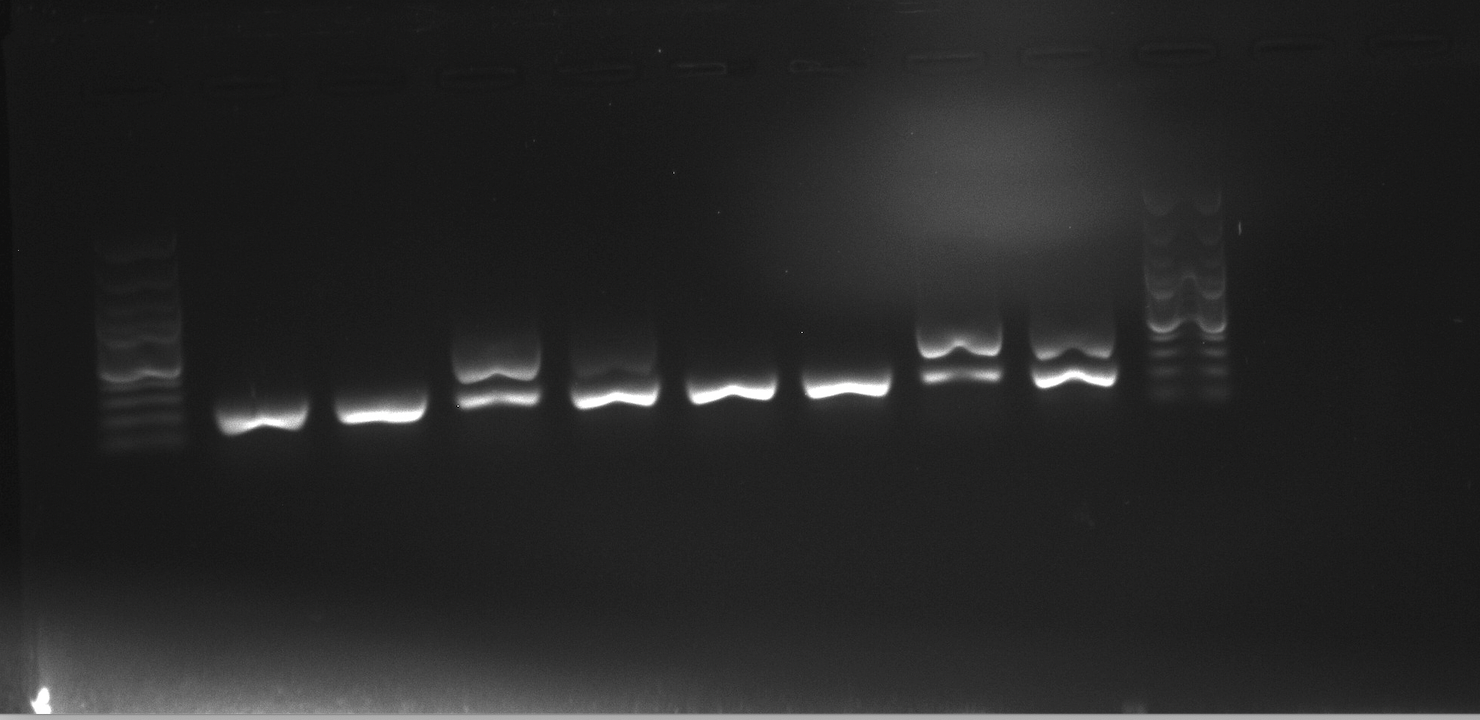

Supplement: Supplementary file 8 — Source Data for Figure 4 [file EMBJ-42-e113168-s010.zip › Figure 4/4D/HNRNPH KD Endo RBM3 set 3.tiff]

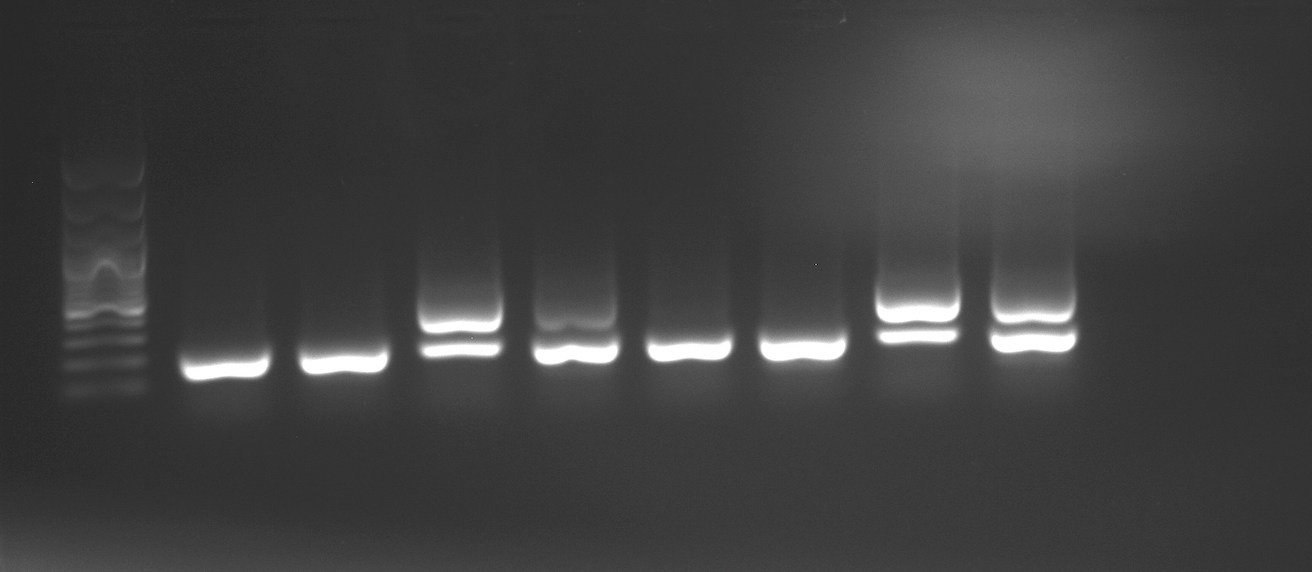

Supplement: Supplementary file 8 — Source Data for Figure 4 [file EMBJ-42-e113168-s010.zip › Figure 4/4D/HNRNPH KD Endo RBM3 set 2.tiff]

# Set 1

37 °C

32 °C

Control KD

HNRNPH1 KD

Control KD

HNRNPH1 KD

SMG1i

-

+

-

+

-

+

-

+

1kb

0.5kb

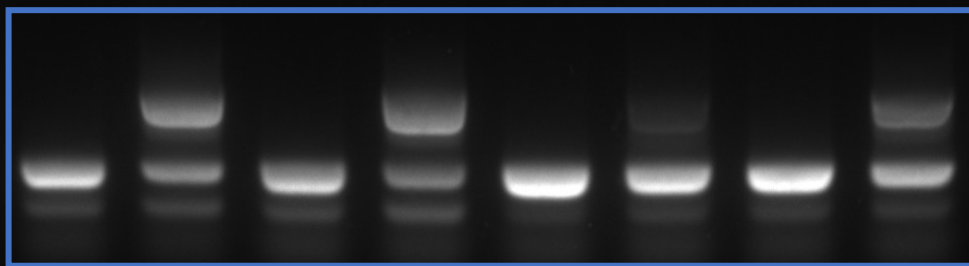

Supplement: Supplementary file 8 — Source Data for Figure 4 [file EMBJ-42-e113168-s010.zip › Figure 4/4D/Labelled HNRNPH KD Endo RBM3 set 1 used in the figure.pdf]

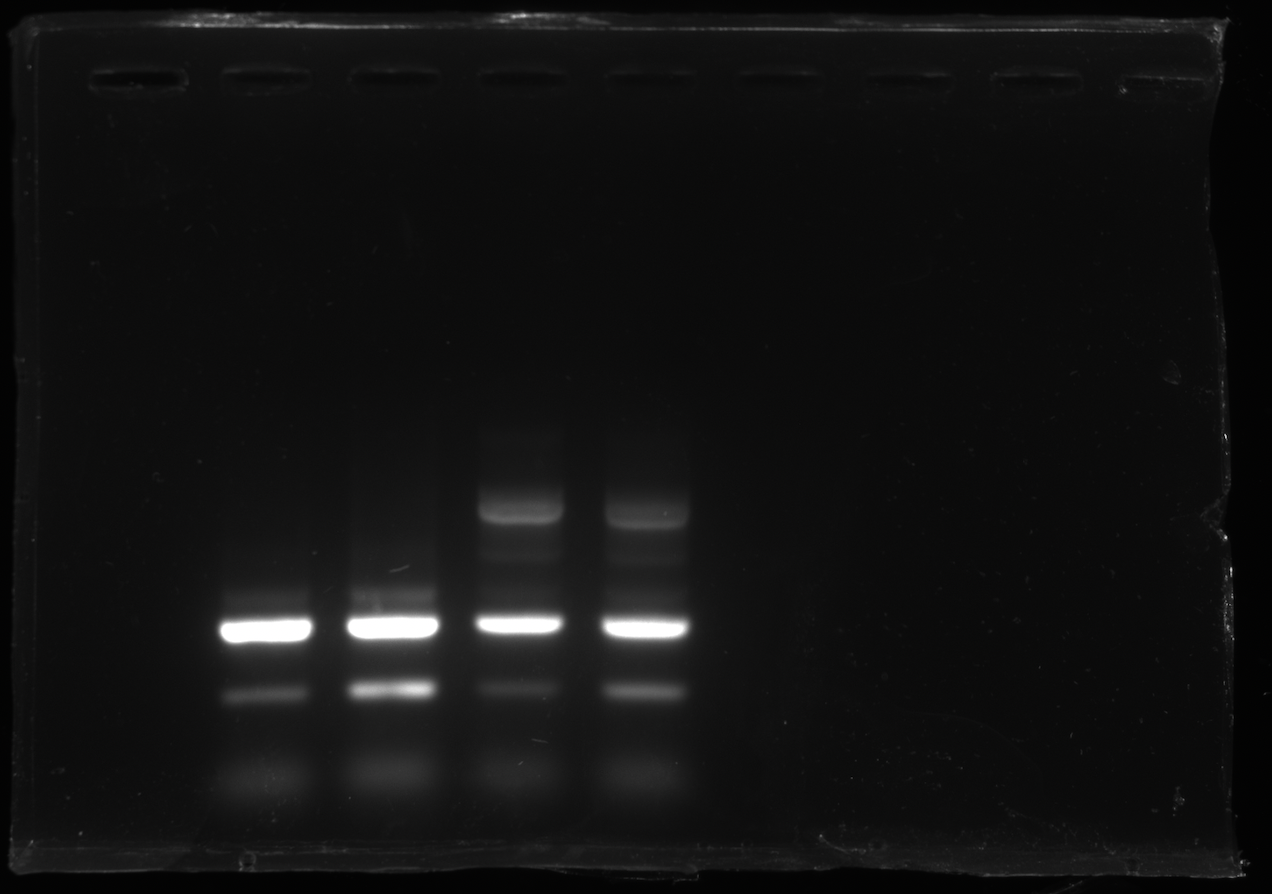

Supplement: Supplementary file 8 — Source Data for Figure 4 [file EMBJ-42-e113168-s010.zip › Figure 4/4F/HNRNPH1 OX Endo RBM3 Hek set 1 Used in the figure.tiff]

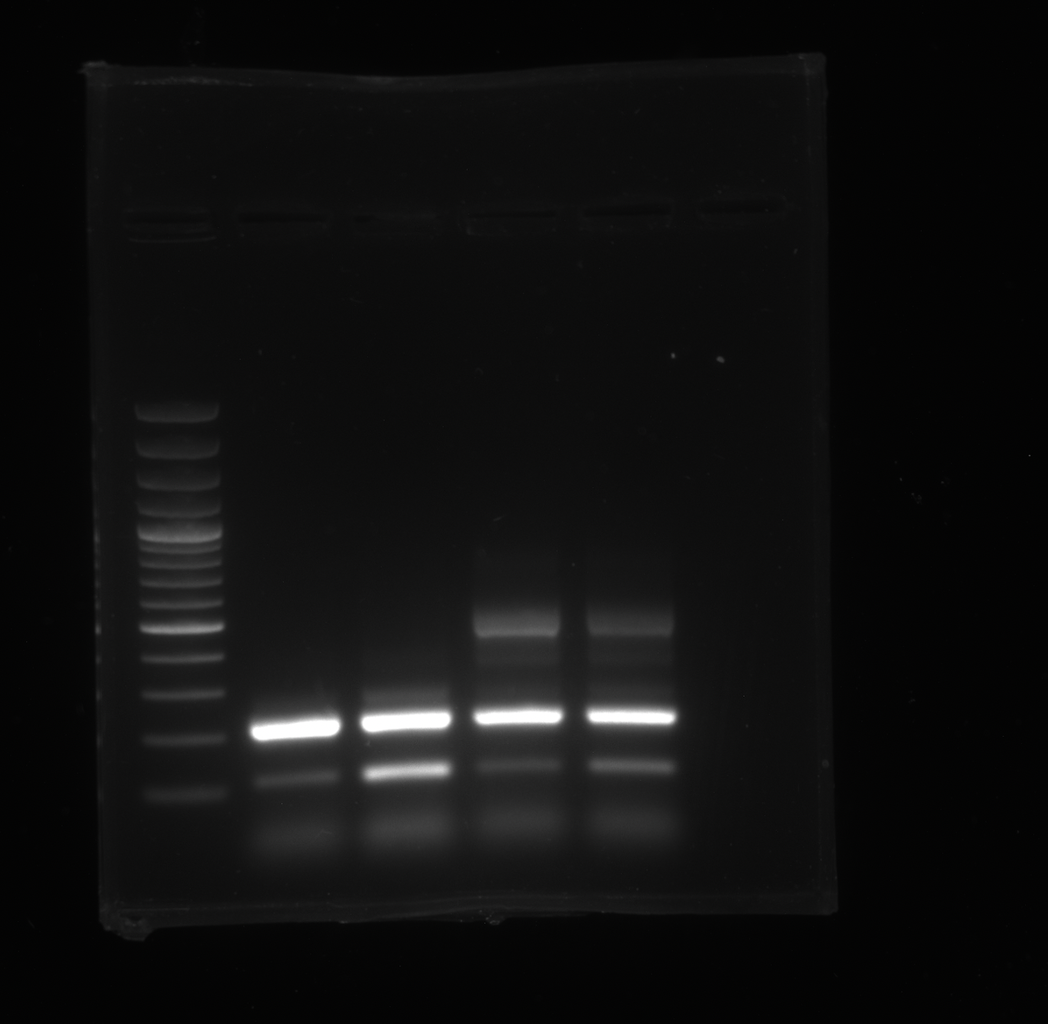

Supplement: Supplementary file 8 — Source Data for Figure 4 [file EMBJ-42-e113168-s010.zip › Figure 4/4F/HNRNPH1 OX Endo RBM3 Hek set 2.tiff]

|       | Set 2   |             |         |             | Set 3   |             |         |             |
|-------|---------|-------------|---------|-------------|---------|-------------|---------|-------------|
|       | Control | FLAG-HNRNPH | Control | FLAG-HNRNPH | Control | FLAG-HNRNPH | Control | FLAG-HNRNPH |
| SMG1i | -       |             | +       | +           | -       |             | +       | +           |

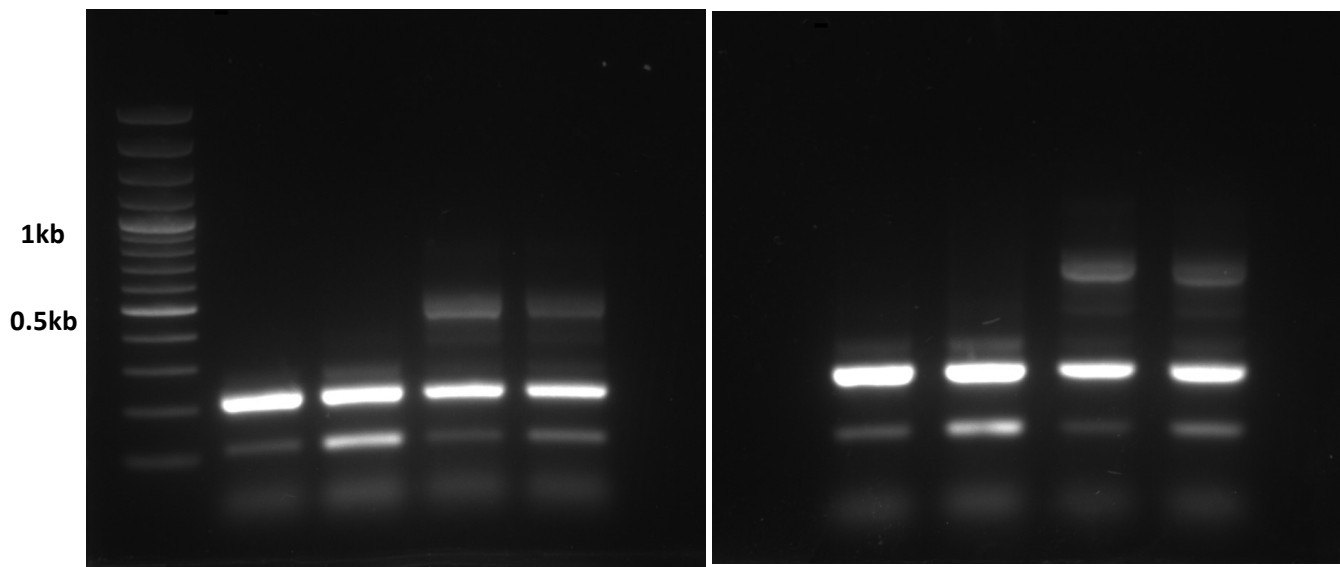

Supplement: Supplementary file 8 — Source Data for Figure 4 [file EMBJ-42-e113168-s010.zip › Figure 4/4F/Labelled HNRNPH1 OX Endo RBM3 Hek sets 2 and 3.pdf]

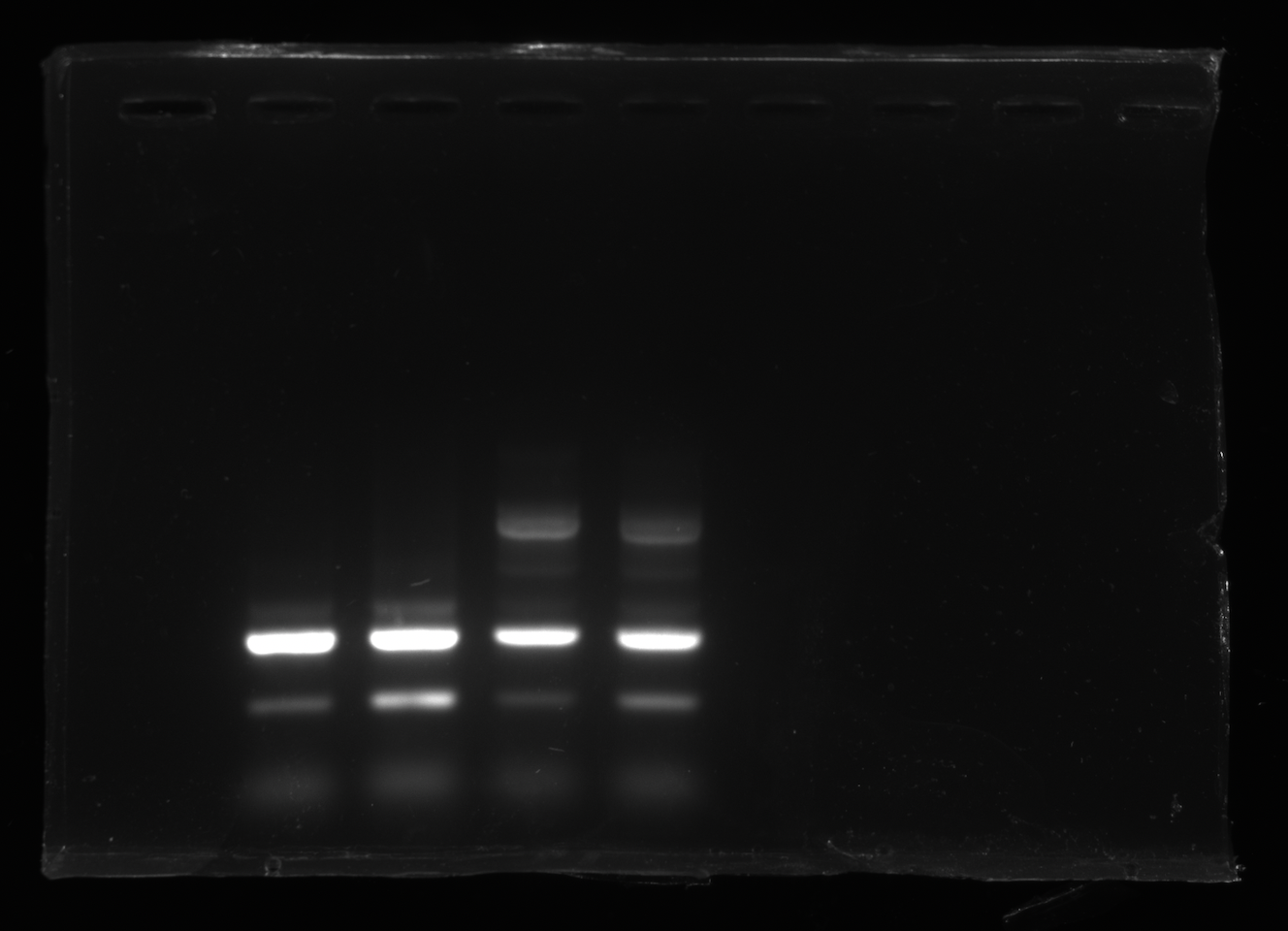

Supplement: Supplementary file 8 — Source Data for Figure 4 [file EMBJ-42-e113168-s010.zip › Figure 4/4F/HNRNPH1 OX Endo RBM3 Hek set 3.tiff]

Set 1

SMG1i

Control

FLAG-HNRNPH

Control

FLAG-HNRNPH

-

+

+

1kb

0.5kb

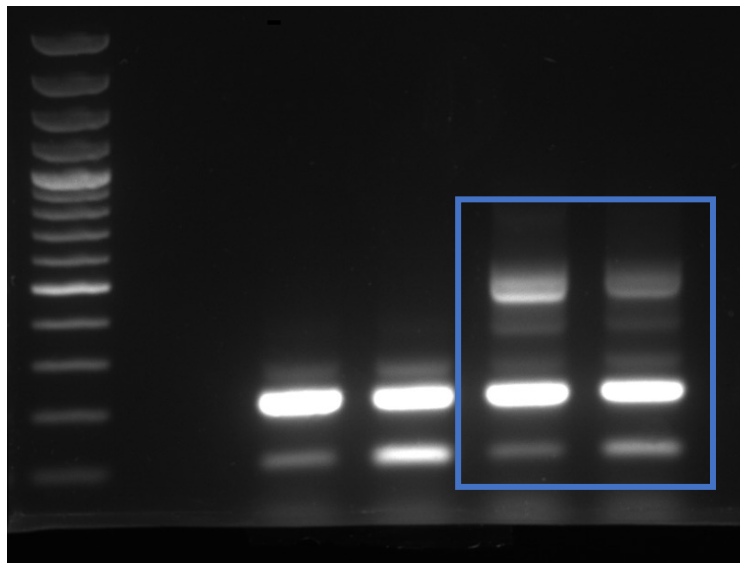

Supplement: Supplementary file 8 — Source Data for Figure 4 [file EMBJ-42-e113168-s010.zip › Figure 4/4F/Labelled HNRNPH1 OX Endo RBM3 Hek set 1 Used in the figure.pdf]

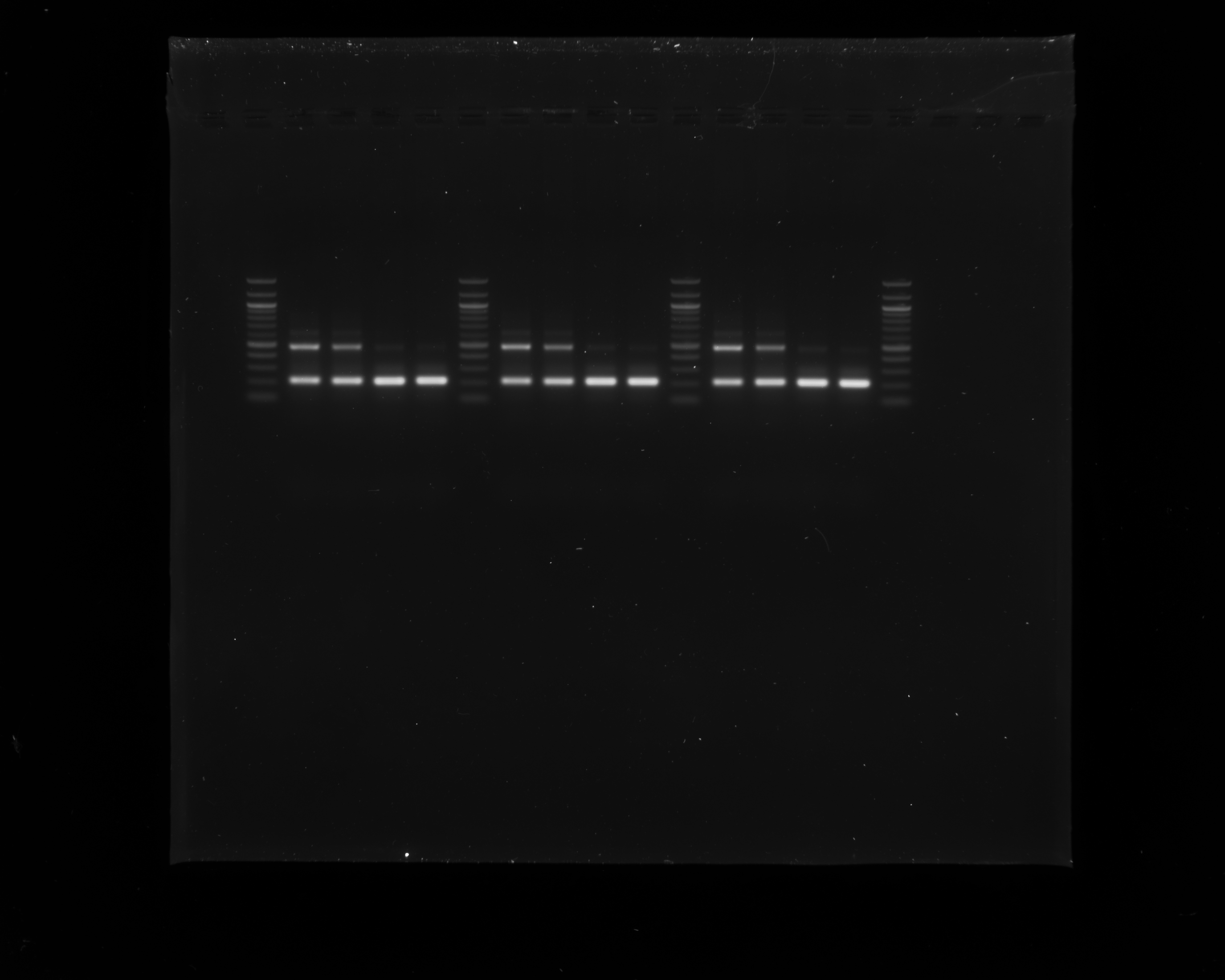

Supplement: Supplementary file 8 — Source Data for Figure 4 [file EMBJ-42-e113168-s010.zip › Figure 4/4H/RT-PCR HNRNPH1 OX Endo RBM3 PE i-neurons set 1-3.tif]

Set  
2

Set 3

Control

FLAG-HNRNPH

Control

FLAG-HNRNPH

SMG1i

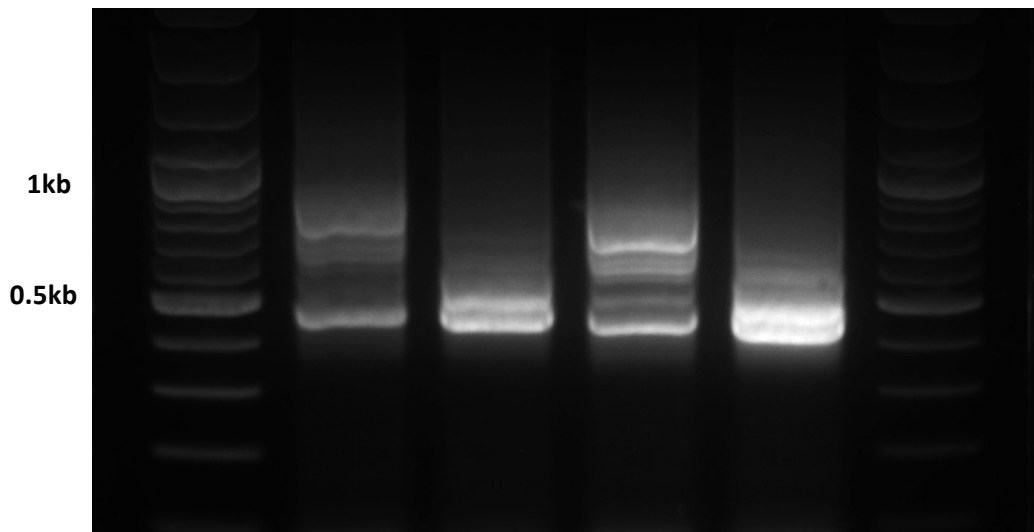

Supplement: Supplementary file 8 — Source Data for Figure 4 [file EMBJ-42-e113168-s010.zip › Figure 4/4G/Labelled HNRNPH OX Minigene WT old sets 2 and 3.pdf]

Set 1

SMG1i

Control

FLAG-HNRNPH

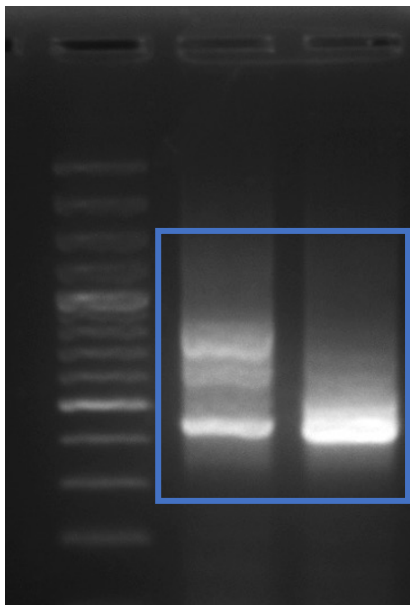

1kb

0.5kb

Supplement: Supplementary file 8 — Source Data for Figure 4 [file EMBJ-42-e113168-s010.zip › Figure 4/4G/Labelled HNRNPH OX Minigene WT old set 1 Used in the figure.pdf]

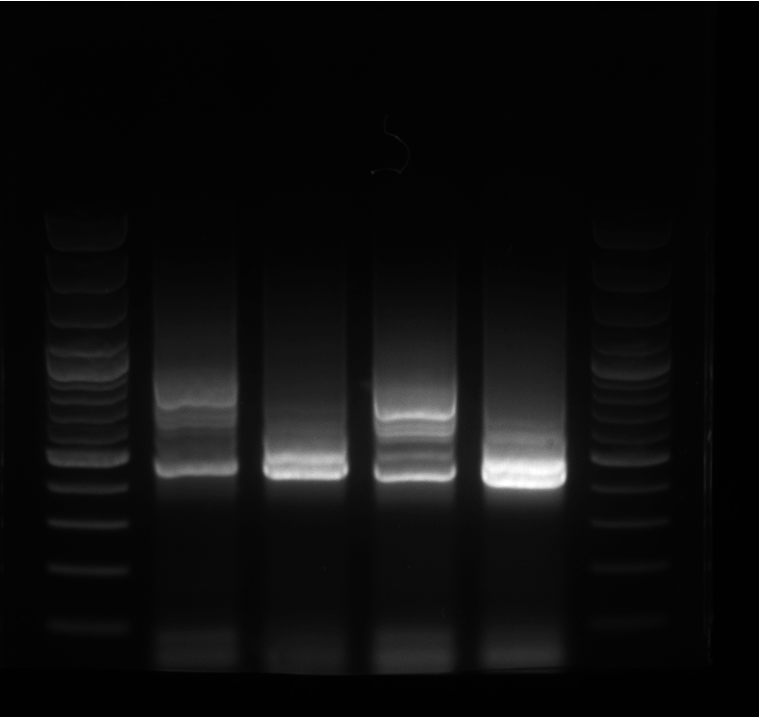

Supplement: Supplementary file 8 — Source Data for Figure 4 [file EMBJ-42-e113168-s010.zip › Figure 4/4G/HNRNPH OX Minigene wt old sets 2 and 3.png]

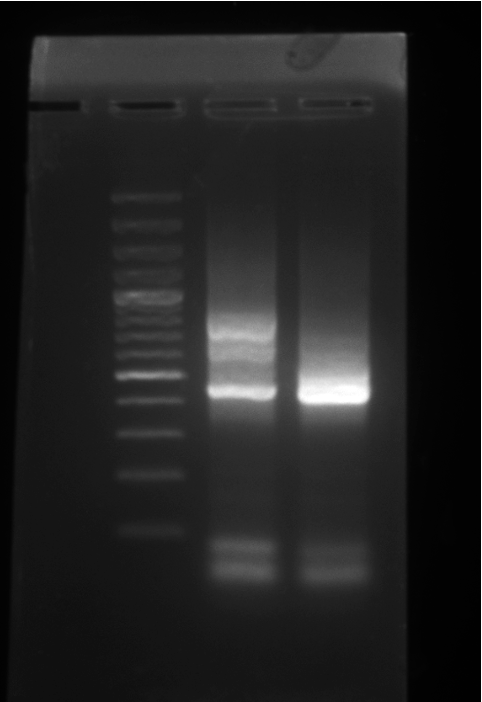

Supplement: Supplementary file 8 — Source Data for Figure 4 [file EMBJ-42-e113168-s010.zip › Figure 4/4G/HNRNPH OX Minigene wt old set 1 Used in the figure.png]

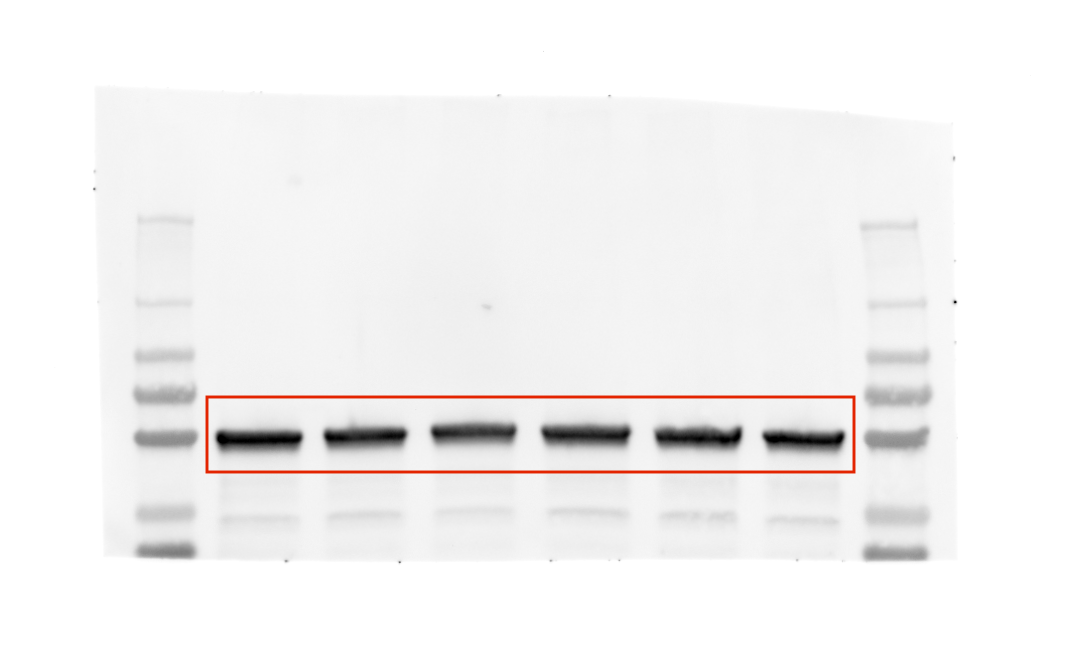

Supplement: Supplementary file 9 — Source Data for Figure 5 [file EMBJ-42-e113168-s002.zip › Figure 5/5A/western_HNRNPH1.tif]

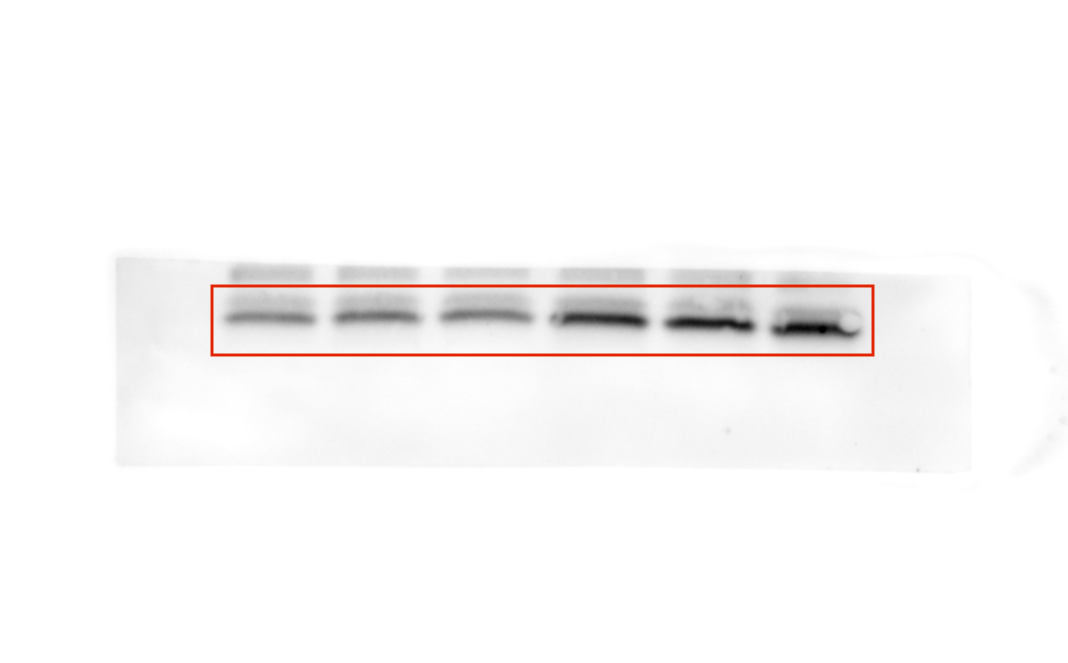

Supplement: Supplementary file 9 — Source Data for Figure 5 [file EMBJ-42-e113168-s002.zip › Figure 5/5A/western_RBM3.tif]

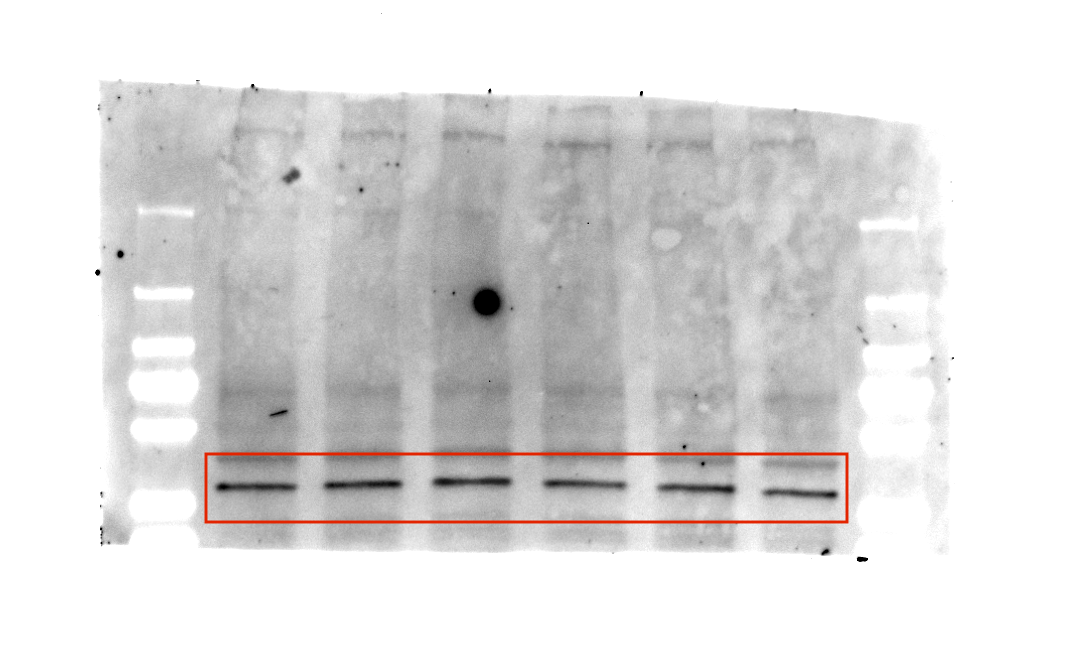

Supplement: Supplementary file 9 — Source Data for Figure 5 [file EMBJ-42-e113168-s002.zip › Figure 5/5A/western_GAPDH.tif]

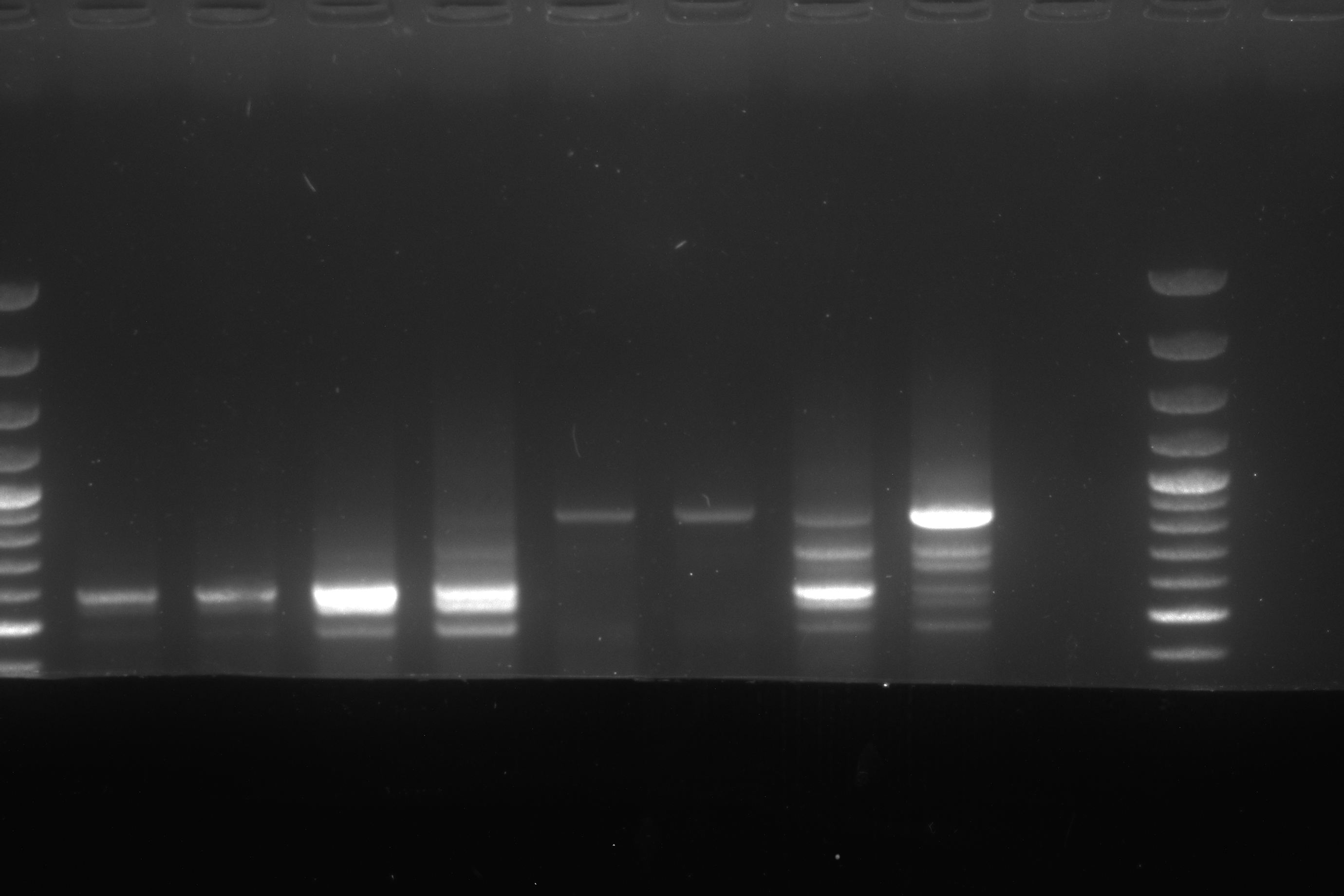

Supplement: Supplementary file 9 — Source Data for Figure 5 [file EMBJ-42-e113168-s002.zip › Figure 5/5D/RBM3 Minigene mut set 3 HeLa.Tif]

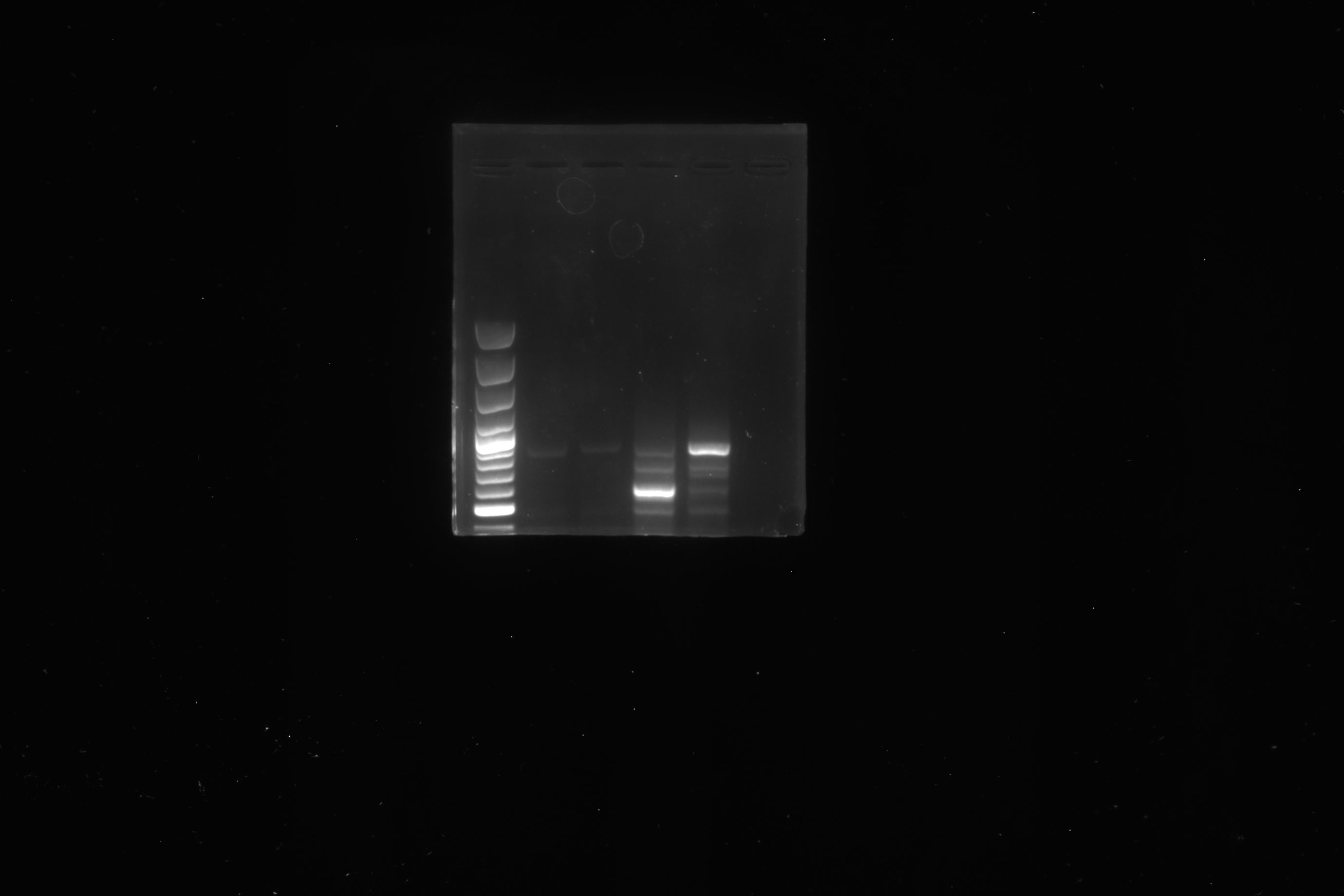

Supplement: Supplementary file 9 — Source Data for Figure 5 [file EMBJ-42-e113168-s002.zip › Figure 5/5D/RBM3 Minigene mut set 2 HeLa.Tif]

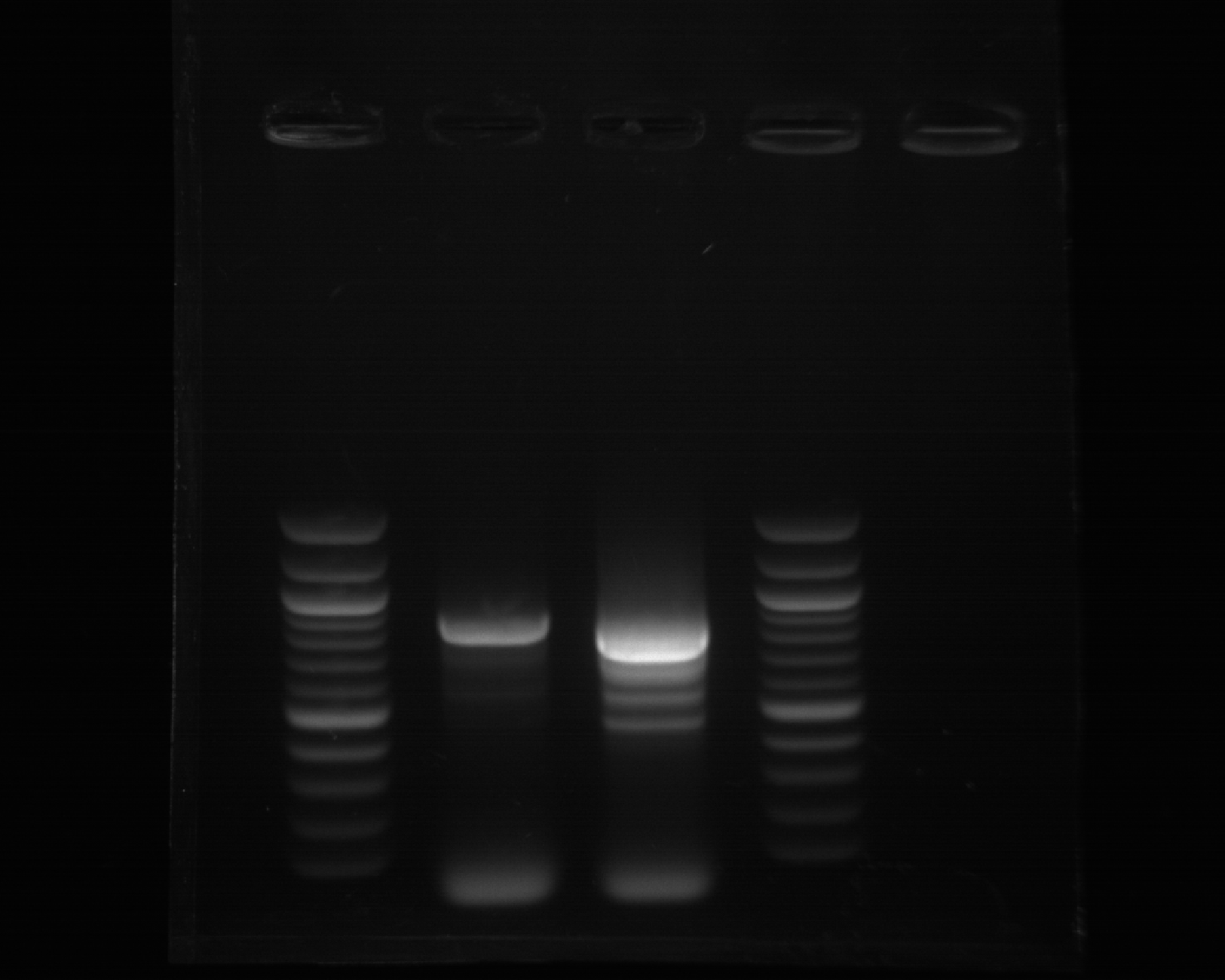

Supplement: Supplementary file 9 — Source Data for Figure 5 [file EMBJ-42-e113168-s002.zip › Figure 5/5D/RBM3 Minigene mut set 1 HeLa.png]

### Set 3

SMG1i -

SMG1i +

WT

mut

WT

mut

37

32

37

32

37

32

37

32

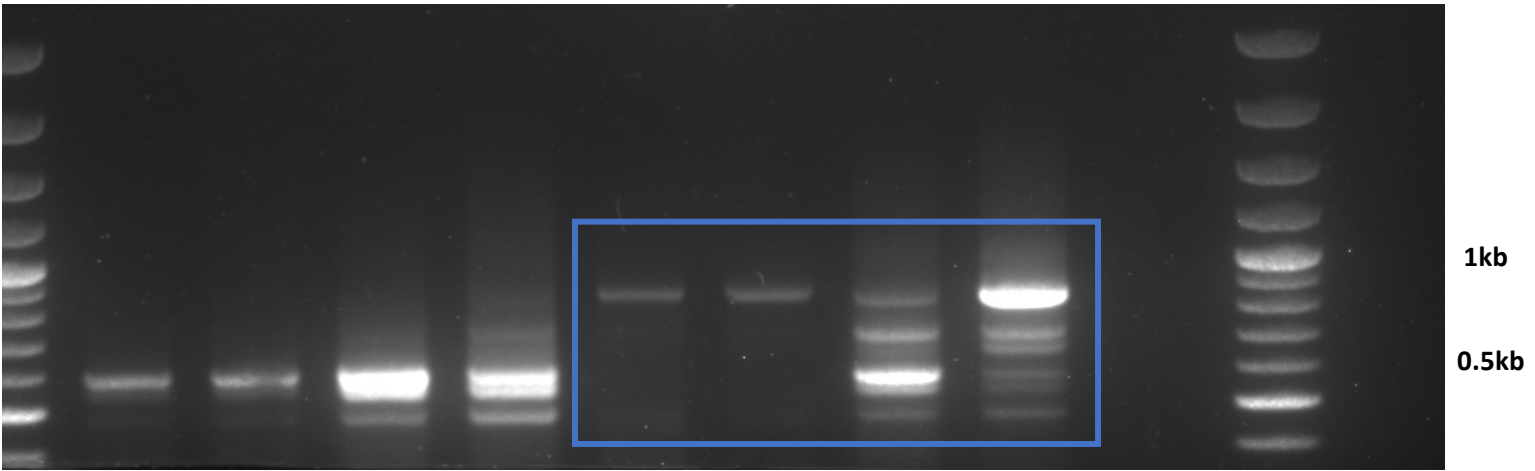

Supplement: Supplementary file 9 — Source Data for Figure 5 [file EMBJ-42-e113168-s002.zip › Figure 5/5D/Labelled HNRNPH OX Minigene wt mut HeLa set 3 Used in the figure.pdf]

# Set 1

SMG1i      37°C    32°C  
              +        +

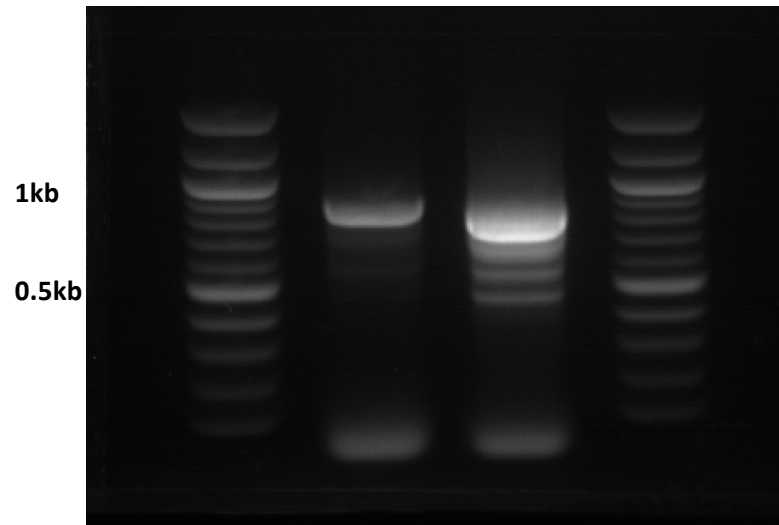

delGGGGMut

SMG1i      37°C      32°C  
              -        +        -        +

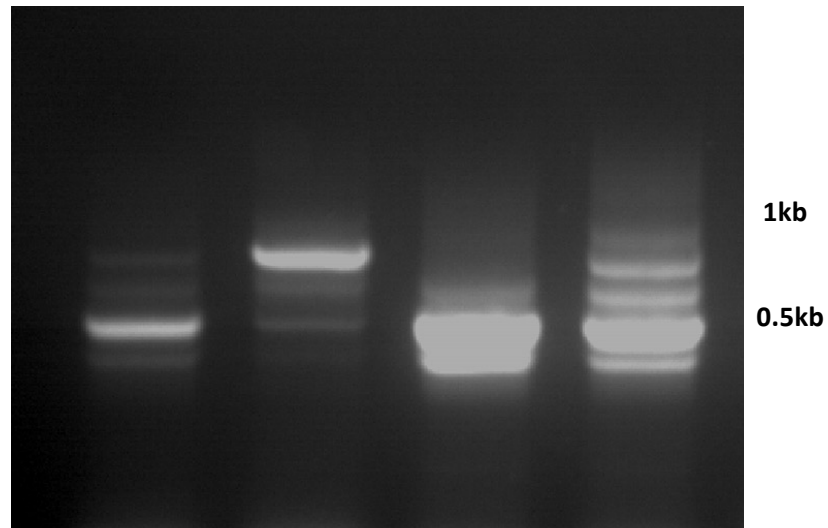

WT

Supplement: Supplementary file 9 — Source Data for Figure 5 [file EMBJ-42-e113168-s002.zip › Figure 5/5D/Labelled HNRNPH OX Minigene wt mut HeLA set 1.pdf]

## Set 2

WT

mut

All SMG1i

37

32

37

32

1kb

0.5kb

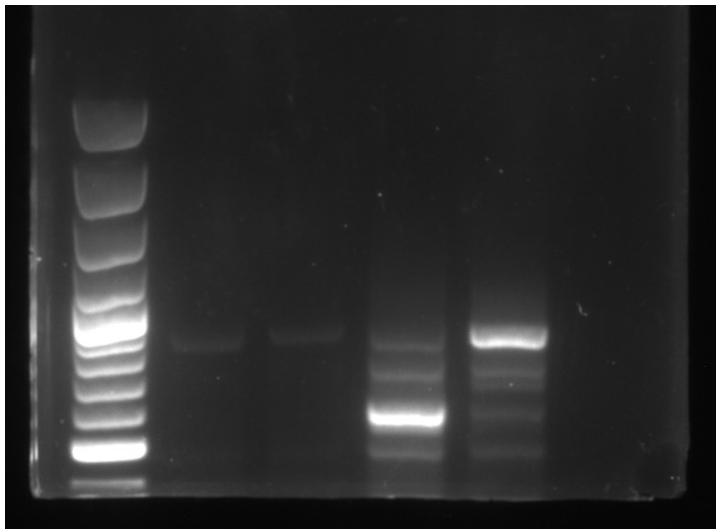

Supplement: Supplementary file 9 — Source Data for Figure 5 [file EMBJ-42-e113168-s002.zip › Figure 5/5D/Labelled HNRNPH OX Minigene wt mut HeLa set 2.pdf]
