# Supplementary figures and images for: HNRNPH1 regulates the neuroprotective cold‐shock protein RBM3 expression through poison exon exclusion (part 2 of 3)
Source: EMBO J. 2023 May 30;42(14):e113168. doi: 10.15252/embj.2022113168 (PMC10350819; doi:10.15252/embj.2022113168)

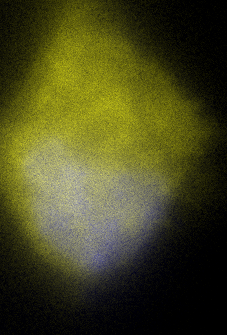

Supplement: Supplementary file 5 — Source Data for Figure 1 [file EMBJ-42-e113168-s008.zip › Figure 1/1A/Images/Replicate 2/37C/37C_S2r1__070.tif]

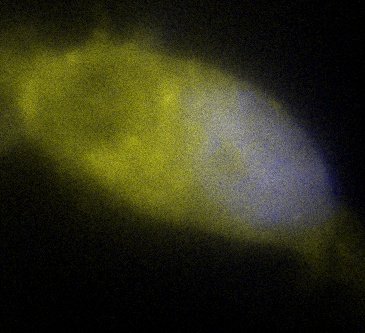

Supplement: Supplementary file 5 — Source Data for Figure 1 [file EMBJ-42-e113168-s008.zip › Figure 1/1A/Images/Replicate 2/37C/37C_S2r1__064.tif]

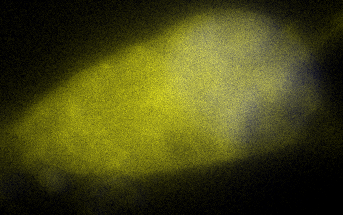

Supplement: Supplementary file 5 — Source Data for Figure 1 [file EMBJ-42-e113168-s008.zip › Figure 1/1A/Images/Replicate 2/37C/37C_S2r1__059.tif]

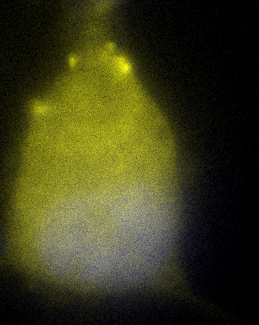

Supplement: Supplementary file 5 — Source Data for Figure 1 [file EMBJ-42-e113168-s008.zip › Figure 1/1A/Images/Replicate 2/37C/37C_S2r1__065.tif]

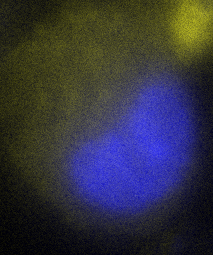

Supplement: Supplementary file 5 — Source Data for Figure 1 [file EMBJ-42-e113168-s008.zip › Figure 1/1A/Images/Replicate 2/37C/37C_S2r1__071.tif]

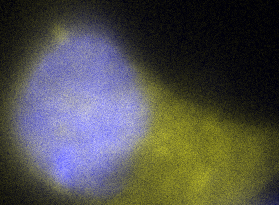

Supplement: Supplementary file 5 — Source Data for Figure 1 [file EMBJ-42-e113168-s008.zip › Figure 1/1A/Images/Replicate 2/37C/37C_S2r1__067.tif]

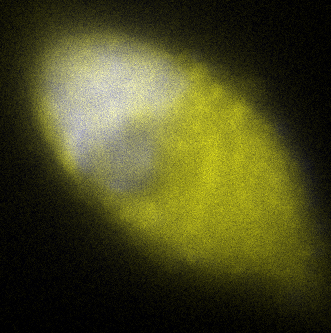

Supplement: Supplementary file 5 — Source Data for Figure 1 [file EMBJ-42-e113168-s008.zip › Figure 1/1A/Images/Replicate 2/37C/37C_S2r1__073.tif]

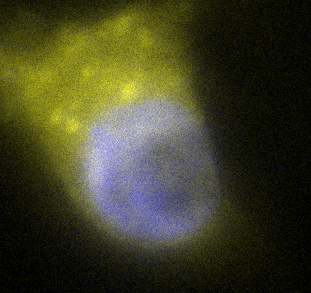

Supplement: Supplementary file 5 — Source Data for Figure 1 [file EMBJ-42-e113168-s008.zip › Figure 1/1A/Images/Replicate 2/37C/37C_S2r1__072.tif]

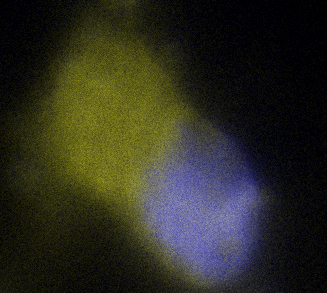

Supplement: Supplementary file 5 — Source Data for Figure 1 [file EMBJ-42-e113168-s008.zip › Figure 1/1A/Images/Replicate 2/37C/37C_S2r1__066.tif]

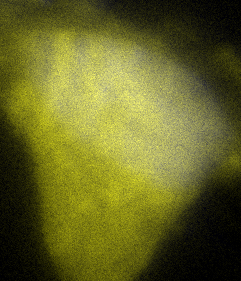

Supplement: Supplementary file 5 — Source Data for Figure 1 [file EMBJ-42-e113168-s008.zip › Figure 1/1A/Images/Replicate 2/37C/37C_S2r1__062.tif]

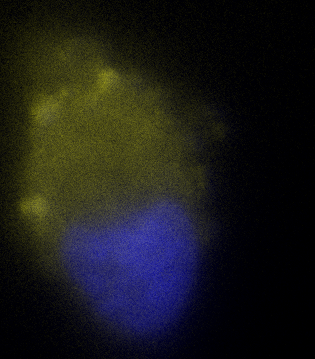

Supplement: Supplementary file 5 — Source Data for Figure 1 [file EMBJ-42-e113168-s008.zip › Figure 1/1A/Images/Replicate 2/37C/37C_S2r1__076.tif]

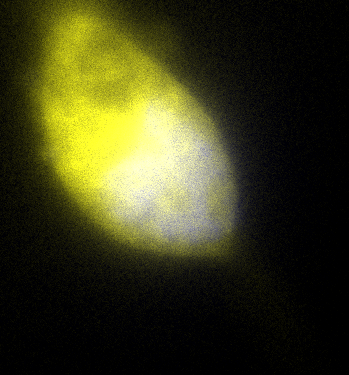

Supplement: Supplementary file 5 — Source Data for Figure 1 [file EMBJ-42-e113168-s008.zip › Figure 1/1A/Images/Replicate 2/37C/37C_S2r1__077.tif]

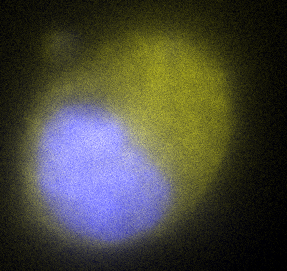

Supplement: Supplementary file 5 — Source Data for Figure 1 [file EMBJ-42-e113168-s008.zip › Figure 1/1A/Images/Replicate 2/37C/37C_S2r1__063.tif]

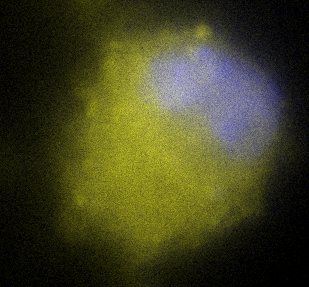

Supplement: Supplementary file 5 — Source Data for Figure 1 [file EMBJ-42-e113168-s008.zip › Figure 1/1A/Images/Replicate 2/37C/37C_S2r1__075.tif]

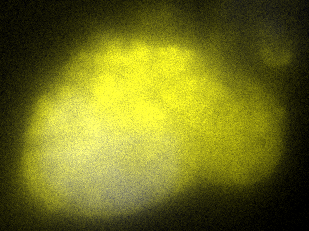

Supplement: Supplementary file 5 — Source Data for Figure 1 [file EMBJ-42-e113168-s008.zip › Figure 1/1A/Images/Replicate 2/37C/37C_S2r1__061.tif]

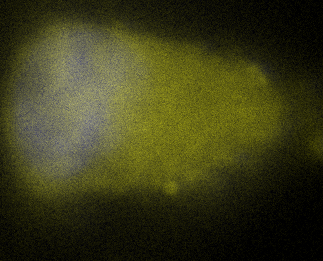

Supplement: Supplementary file 5 — Source Data for Figure 1 [file EMBJ-42-e113168-s008.zip › Figure 1/1A/Images/Replicate 2/37C/37C_S2r1__060.tif]

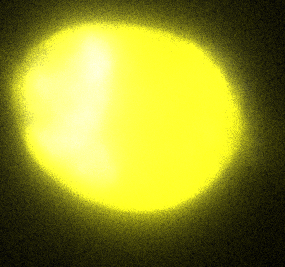

Supplement: Supplementary file 5 — Source Data for Figure 1 [file EMBJ-42-e113168-s008.zip › Figure 1/1A/Images/Replicate 2/37C/37C_S2r1__074.tif]

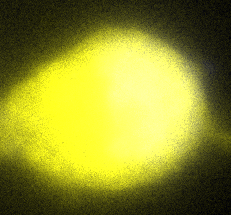

Supplement: Supplementary file 5 — Source Data for Figure 1 [file EMBJ-42-e113168-s008.zip › Figure 1/1A/Images/Replicate 2/37C/37C_S2r2__083.tif]

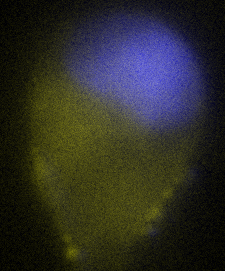

Supplement: Supplementary file 5 — Source Data for Figure 1 [file EMBJ-42-e113168-s008.zip › Figure 1/1A/Images/Replicate 2/37C/37C_S2r2__082.tif]

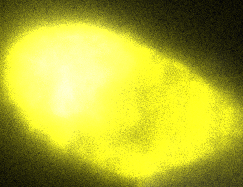

Supplement: Supplementary file 5 — Source Data for Figure 1 [file EMBJ-42-e113168-s008.zip › Figure 1/1A/Images/Replicate 2/37C/37C_S2r2__080.tif]

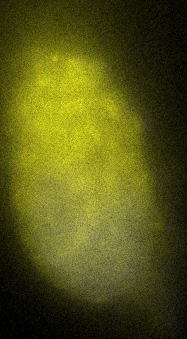

Supplement: Supplementary file 5 — Source Data for Figure 1 [file EMBJ-42-e113168-s008.zip › Figure 1/1A/Images/Replicate 2/37C/37C_S2r2__081.tif]

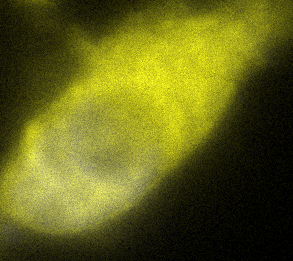

Supplement: Supplementary file 5 — Source Data for Figure 1 [file EMBJ-42-e113168-s008.zip › Figure 1/1A/Images/Replicate 2/37C/37C_S2r2__085.tif]

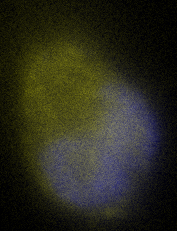

Supplement: Supplementary file 5 — Source Data for Figure 1 [file EMBJ-42-e113168-s008.zip › Figure 1/1A/Images/Replicate 2/37C/37C_S2r2__084.tif]

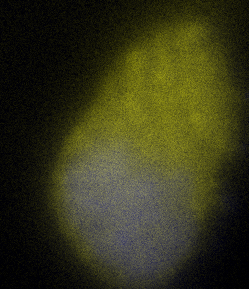

Supplement: Supplementary file 5 — Source Data for Figure 1 [file EMBJ-42-e113168-s008.zip › Figure 1/1A/Images/Replicate 2/37C/37C_S2r2__086.tif]

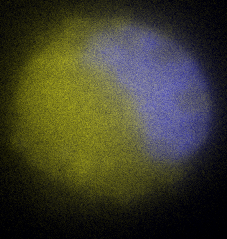

Supplement: Supplementary file 5 — Source Data for Figure 1 [file EMBJ-42-e113168-s008.zip › Figure 1/1A/Images/Replicate 2/37C/37C_S2r2__079.tif]

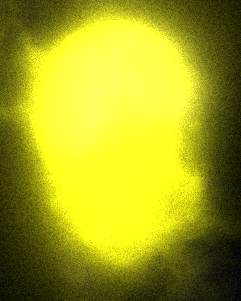

Supplement: Supplementary file 5 — Source Data for Figure 1 [file EMBJ-42-e113168-s008.zip › Figure 1/1A/Images/Replicate 2/37C/37C_S2r2__087.tif]

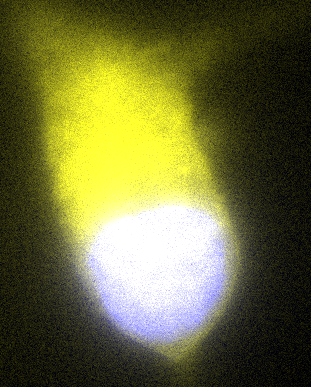

Supplement: Supplementary file 5 — Source Data for Figure 1 [file EMBJ-42-e113168-s008.zip › Figure 1/1A/Images/Replicate 3/32C/32C_S3r5__186.tif]

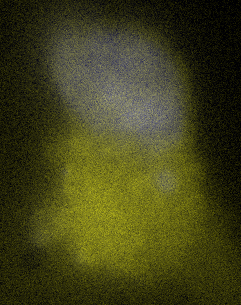

Supplement: Supplementary file 5 — Source Data for Figure 1 [file EMBJ-42-e113168-s008.zip › Figure 1/1A/Images/Replicate 3/32C/32C_S3r5__192.tif]

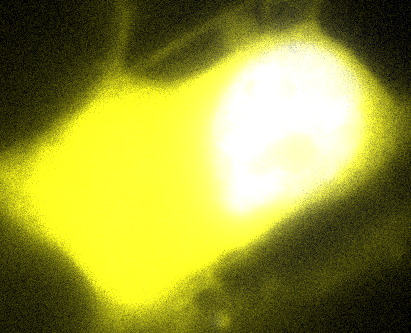

Supplement: Supplementary file 5 — Source Data for Figure 1 [file EMBJ-42-e113168-s008.zip › Figure 1/1A/Images/Replicate 3/32C/32C_S3r5__179.tif]

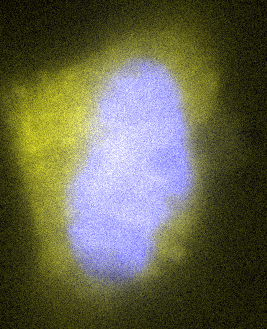

Supplement: Supplementary file 5 — Source Data for Figure 1 [file EMBJ-42-e113168-s008.zip › Figure 1/1A/Images/Replicate 3/32C/32C_S3r2__134.tif]

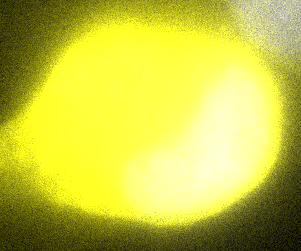

Supplement: Supplementary file 5 — Source Data for Figure 1 [file EMBJ-42-e113168-s008.zip › Figure 1/1A/Images/Replicate 3/32C/32C_S3r2__120.tif]

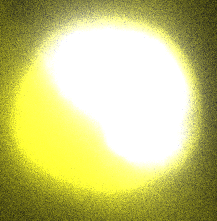

Supplement: Supplementary file 5 — Source Data for Figure 1 [file EMBJ-42-e113168-s008.zip › Figure 1/1A/Images/Replicate 3/32C/32C_S3r3__135.tif]

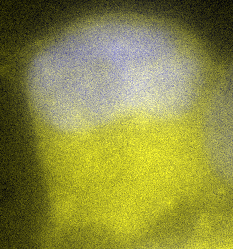

Supplement: Supplementary file 5 — Source Data for Figure 1 [file EMBJ-42-e113168-s008.zip › Figure 1/1A/Images/Replicate 3/32C/32C_S3r2__121.tif]

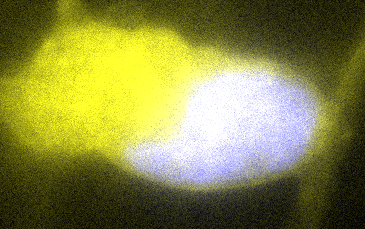

Supplement: Supplementary file 5 — Source Data for Figure 1 [file EMBJ-42-e113168-s008.zip › Figure 1/1A/Images/Replicate 3/32C/32C_S3r4__178.tif]

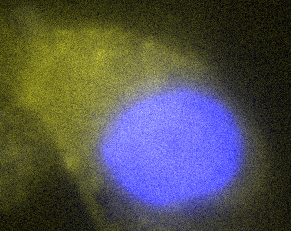

Supplement: Supplementary file 5 — Source Data for Figure 1 [file EMBJ-42-e113168-s008.zip › Figure 1/1A/Images/Replicate 3/32C/32C_S3r5__193.tif]

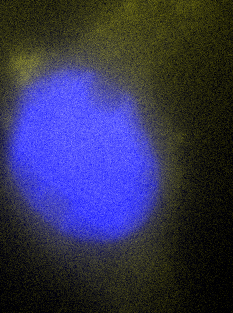

Supplement: Supplementary file 5 — Source Data for Figure 1 [file EMBJ-42-e113168-s008.zip › Figure 1/1A/Images/Replicate 3/32C/32C_S3r5__187.tif]

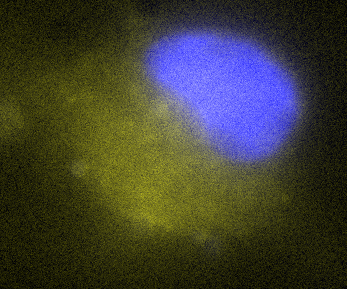

Supplement: Supplementary file 5 — Source Data for Figure 1 [file EMBJ-42-e113168-s008.zip › Figure 1/1A/Images/Replicate 3/32C/32C_S3r5__191.tif]

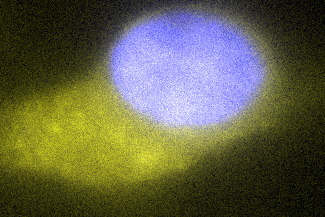

Supplement: Supplementary file 5 — Source Data for Figure 1 [file EMBJ-42-e113168-s008.zip › Figure 1/1A/Images/Replicate 3/32C/32C_S3r5__185.tif]

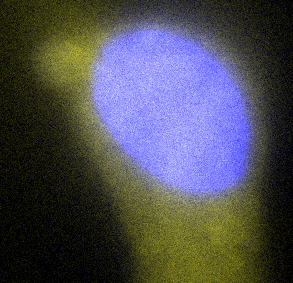

Supplement: Supplementary file 5 — Source Data for Figure 1 [file EMBJ-42-e113168-s008.zip › Figure 1/1A/Images/Replicate 3/32C/32C_S3r2__123.tif]

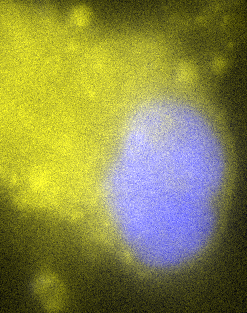

Supplement: Supplementary file 5 — Source Data for Figure 1 [file EMBJ-42-e113168-s008.zip › Figure 1/1A/Images/Replicate 3/32C/32C_S3r3__137.tif]

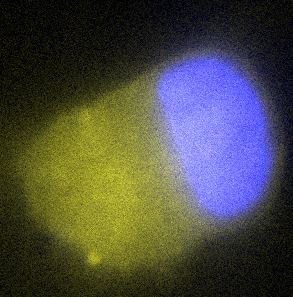

Supplement: Supplementary file 5 — Source Data for Figure 1 [file EMBJ-42-e113168-s008.zip › Figure 1/1A/Images/Replicate 3/32C/32C_S3r2__122.tif]

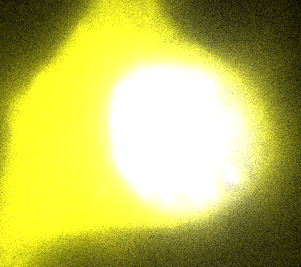

Supplement: Supplementary file 5 — Source Data for Figure 1 [file EMBJ-42-e113168-s008.zip › Figure 1/1A/Images/Replicate 3/32C/32C_S3r3__136.tif]

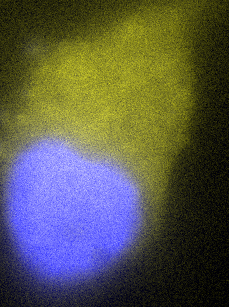

Supplement: Supplementary file 5 — Source Data for Figure 1 [file EMBJ-42-e113168-s008.zip › Figure 1/1A/Images/Replicate 3/32C/32C_S3r5__184.tif]

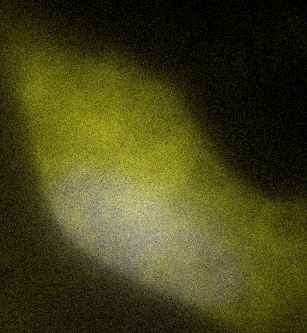

Supplement: Supplementary file 5 — Source Data for Figure 1 [file EMBJ-42-e113168-s008.zip › Figure 1/1A/Images/Replicate 3/32C/32C_S3r5__190.tif]

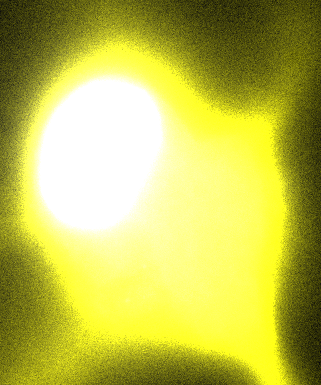

Supplement: Supplementary file 5 — Source Data for Figure 1 [file EMBJ-42-e113168-s008.zip › Figure 1/1A/Images/Replicate 3/32C/32C_S3r5__194.tif]

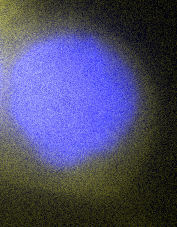

Supplement: Supplementary file 5 — Source Data for Figure 1 [file EMBJ-42-e113168-s008.zip › Figure 1/1A/Images/Replicate 3/32C/32C_S3r5__180.tif]

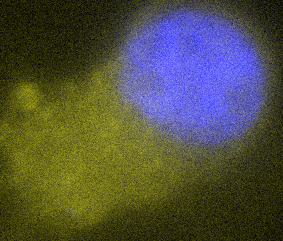

Supplement: Supplementary file 5 — Source Data for Figure 1 [file EMBJ-42-e113168-s008.zip › Figure 1/1A/Images/Replicate 3/32C/32C_S3r4__157.tif]

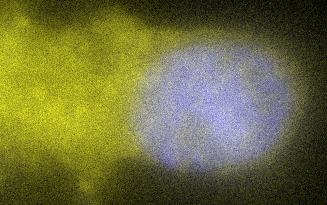

Supplement: Supplementary file 5 — Source Data for Figure 1 [file EMBJ-42-e113168-s008.zip › Figure 1/1A/Images/Replicate 3/32C/32C_S3r2__126.tif]

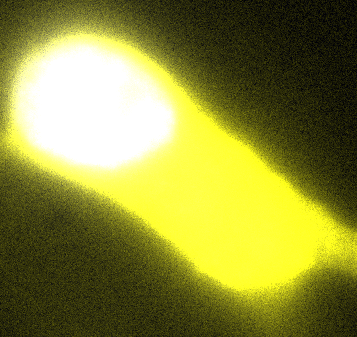

Supplement: Supplementary file 5 — Source Data for Figure 1 [file EMBJ-42-e113168-s008.zip › Figure 1/1A/Images/Replicate 3/32C/32C_S3r2__132.tif]

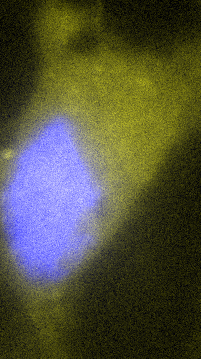

Supplement: Supplementary file 5 — Source Data for Figure 1 [file EMBJ-42-e113168-s008.zip › Figure 1/1A/Images/Replicate 3/32C/32C_S3r2__133.tif]

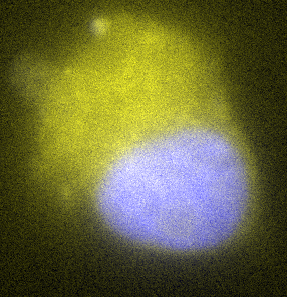

Supplement: Supplementary file 5 — Source Data for Figure 1 [file EMBJ-42-e113168-s008.zip › Figure 1/1A/Images/Replicate 3/32C/32C_S3r2__127.tif]

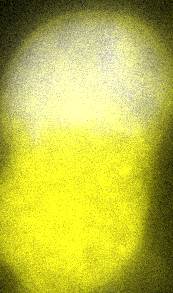

Supplement: Supplementary file 5 — Source Data for Figure 1 [file EMBJ-42-e113168-s008.zip › Figure 1/1A/Images/Replicate 3/32C/32C_S3r5__181.tif]

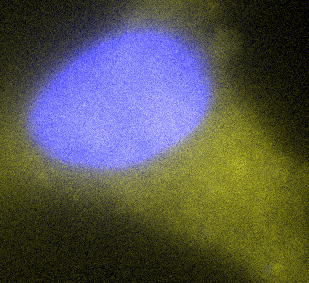

Supplement: Supplementary file 5 — Source Data for Figure 1 [file EMBJ-42-e113168-s008.zip › Figure 1/1A/Images/Replicate 3/32C/32C_S3r5__195.tif]

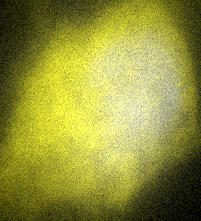

Supplement: Supplementary file 5 — Source Data for Figure 1 [file EMBJ-42-e113168-s008.zip › Figure 1/1A/Images/Replicate 3/32C/32C_S3r5__183.tif]

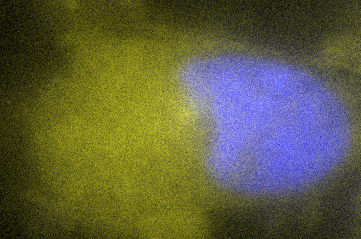

Supplement: Supplementary file 5 — Source Data for Figure 1 [file EMBJ-42-e113168-s008.zip › Figure 1/1A/Images/Replicate 3/32C/32C_S3r5__197.tif]

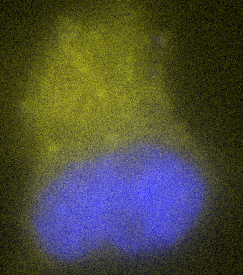

Supplement: Supplementary file 5 — Source Data for Figure 1 [file EMBJ-42-e113168-s008.zip › Figure 1/1A/Images/Replicate 3/32C/32C_S3r4__168.tif]

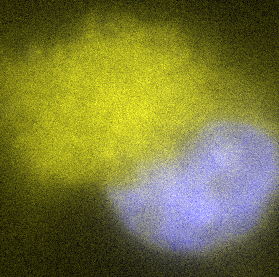

Supplement: Supplementary file 5 — Source Data for Figure 1 [file EMBJ-42-e113168-s008.zip › Figure 1/1A/Images/Replicate 3/32C/32C_S3r2__119.tif]

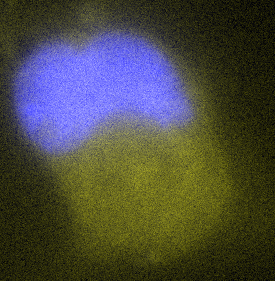

Supplement: Supplementary file 5 — Source Data for Figure 1 [file EMBJ-42-e113168-s008.zip › Figure 1/1A/Images/Replicate 3/32C/32C_S3r2__131.tif]

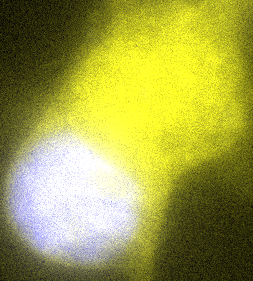

Supplement: Supplementary file 5 — Source Data for Figure 1 [file EMBJ-42-e113168-s008.zip › Figure 1/1A/Images/Replicate 3/32C/32C_S3r2__125.tif]

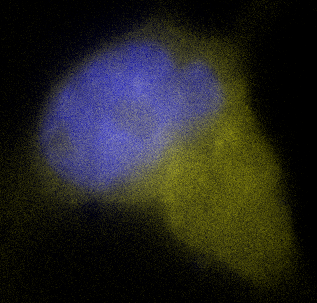

Supplement: Supplementary file 5 — Source Data for Figure 1 [file EMBJ-42-e113168-s008.zip › Figure 1/1A/Images/Replicate 3/32C/32C_S3r2__124.tif]

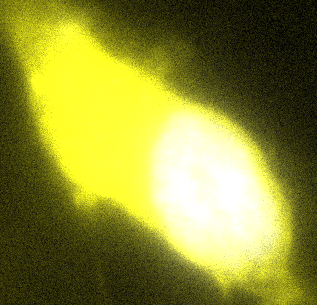

Supplement: Supplementary file 5 — Source Data for Figure 1 [file EMBJ-42-e113168-s008.zip › Figure 1/1A/Images/Replicate 3/32C/32C_S3r2__130.tif]

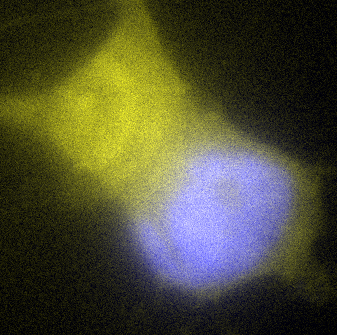

Supplement: Supplementary file 5 — Source Data for Figure 1 [file EMBJ-42-e113168-s008.zip › Figure 1/1A/Images/Replicate 3/32C/32C_S3r2__118.tif]

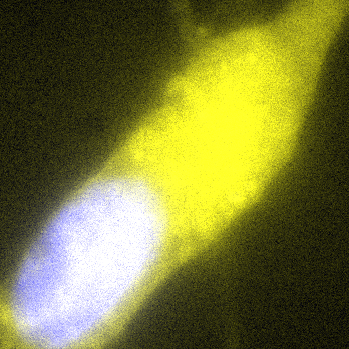

Supplement: Supplementary file 5 — Source Data for Figure 1 [file EMBJ-42-e113168-s008.zip › Figure 1/1A/Images/Replicate 3/32C/32C_S3r4__169.tif]

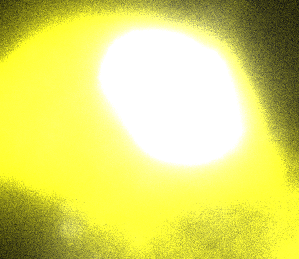

Supplement: Supplementary file 5 — Source Data for Figure 1 [file EMBJ-42-e113168-s008.zip › Figure 1/1A/Images/Replicate 3/32C/32C_S3r5__196.tif]

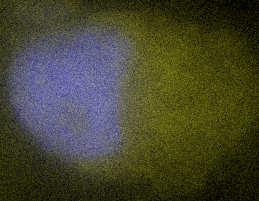

Supplement: Supplementary file 5 — Source Data for Figure 1 [file EMBJ-42-e113168-s008.zip › Figure 1/1A/Images/Replicate 3/32C/32C_S3r5__182.tif]

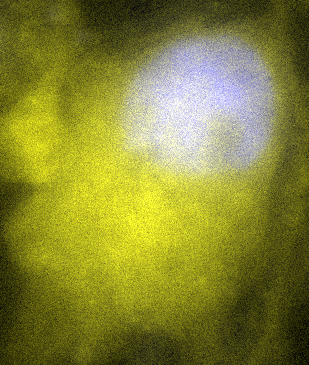

Supplement: Supplementary file 5 — Source Data for Figure 1 [file EMBJ-42-e113168-s008.zip › Figure 1/1A/Images/Replicate 3/32C/32C_S3r6__217.tif]

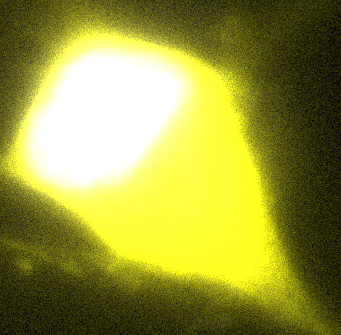

Supplement: Supplementary file 5 — Source Data for Figure 1 [file EMBJ-42-e113168-s008.zip › Figure 1/1A/Images/Replicate 3/32C/32C_S3r6__203.tif]

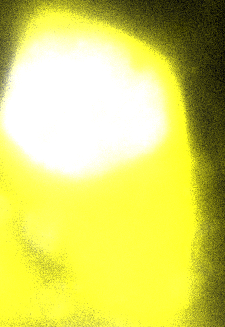

Supplement: Supplementary file 5 — Source Data for Figure 1 [file EMBJ-42-e113168-s008.zip › Figure 1/1A/Images/Replicate 3/32C/32C_S3r1__110.tif]

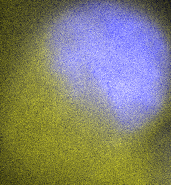

Supplement: Supplementary file 5 — Source Data for Figure 1 [file EMBJ-42-e113168-s008.zip › Figure 1/1A/Images/Replicate 3/32C/32C_S3r1__104.tif]

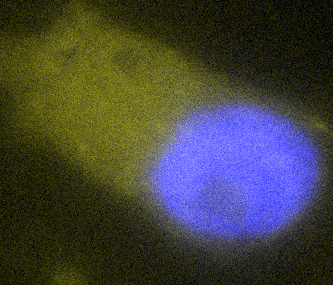

Supplement: Supplementary file 5 — Source Data for Figure 1 [file EMBJ-42-e113168-s008.zip › Figure 1/1A/Images/Replicate 3/32C/32C_S3r3__143.tif]

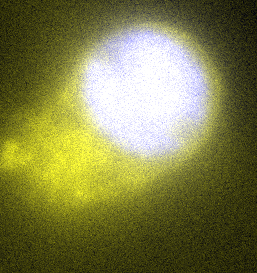

Supplement: Supplementary file 5 — Source Data for Figure 1 [file EMBJ-42-e113168-s008.zip › Figure 1/1A/Images/Replicate 3/32C/32C_S3r3__156.tif]

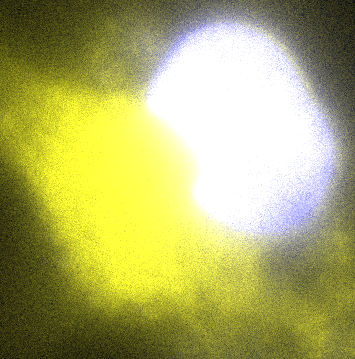

Supplement: Supplementary file 5 — Source Data for Figure 1 [file EMBJ-42-e113168-s008.zip › Figure 1/1A/Images/Replicate 3/32C/32C_S3r3__142.tif]

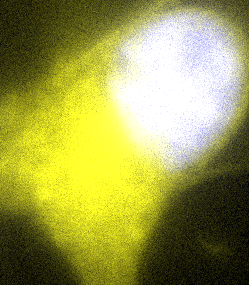

Supplement: Supplementary file 5 — Source Data for Figure 1 [file EMBJ-42-e113168-s008.zip › Figure 1/1A/Images/Replicate 3/32C/32C_S3r1__105.tif]

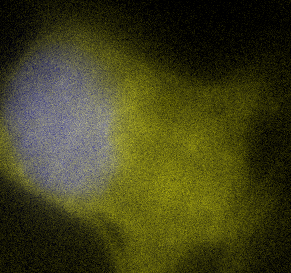

Supplement: Supplementary file 5 — Source Data for Figure 1 [file EMBJ-42-e113168-s008.zip › Figure 1/1A/Images/Replicate 3/32C/32C_S3r1__111.tif]

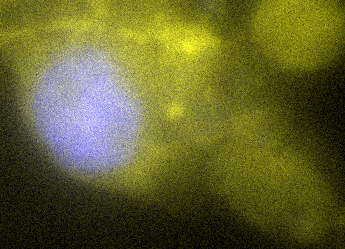

Supplement: Supplementary file 5 — Source Data for Figure 1 [file EMBJ-42-e113168-s008.zip › Figure 1/1A/Images/Replicate 3/32C/32C_S3r6__202.tif]

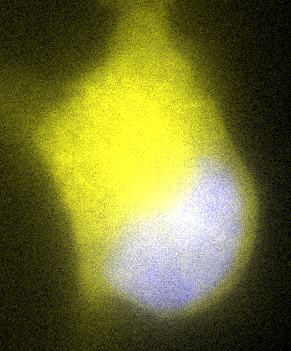

Supplement: Supplementary file 5 — Source Data for Figure 1 [file EMBJ-42-e113168-s008.zip › Figure 1/1A/Images/Replicate 3/32C/32C_S3r6__216.tif]

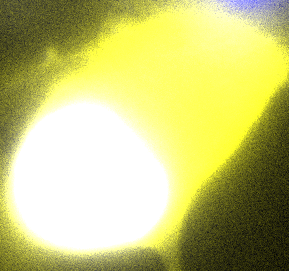

Supplement: Supplementary file 5 — Source Data for Figure 1 [file EMBJ-42-e113168-s008.zip › Figure 1/1A/Images/Replicate 3/32C/32C_S3r6__200.tif]

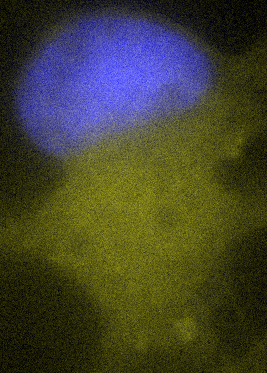

Supplement: Supplementary file 5 — Source Data for Figure 1 [file EMBJ-42-e113168-s008.zip › Figure 1/1A/Images/Replicate 3/32C/32C_S3r6__214.tif]

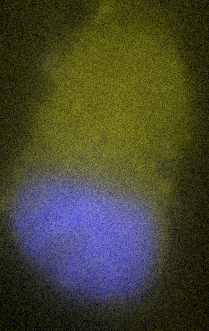

Supplement: Supplementary file 5 — Source Data for Figure 1 [file EMBJ-42-e113168-s008.zip › Figure 1/1A/Images/Replicate 3/32C/32C_S3r1__098.tif]

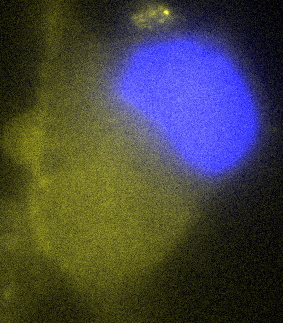

Supplement: Supplementary file 5 — Source Data for Figure 1 [file EMBJ-42-e113168-s008.zip › Figure 1/1A/Images/Replicate 3/32C/32C_S3r1__107.tif]

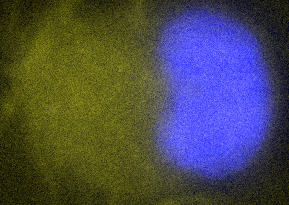

Supplement: Supplementary file 5 — Source Data for Figure 1 [file EMBJ-42-e113168-s008.zip › Figure 1/1A/Images/Replicate 3/32C/32C_S3r1__113.tif]

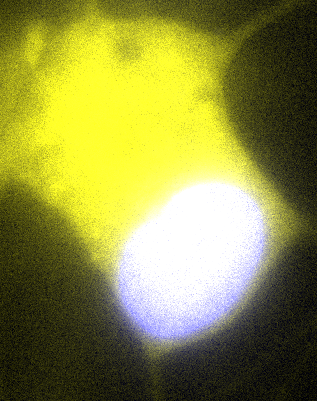

Supplement: Supplementary file 5 — Source Data for Figure 1 [file EMBJ-42-e113168-s008.zip › Figure 1/1A/Images/Replicate 3/32C/32C_S3r3__154.tif]

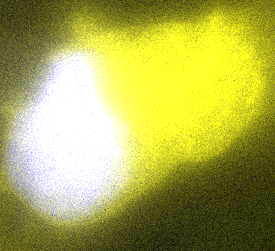

Supplement: Supplementary file 5 — Source Data for Figure 1 [file EMBJ-42-e113168-s008.zip › Figure 1/1A/Images/Replicate 3/32C/32C_S3r3__140.tif]

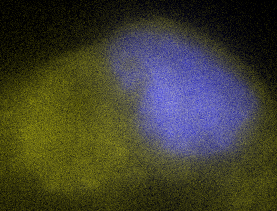

Supplement: Supplementary file 5 — Source Data for Figure 1 [file EMBJ-42-e113168-s008.zip › Figure 1/1A/Images/Replicate 3/32C/32C_S3r3__141.tif]

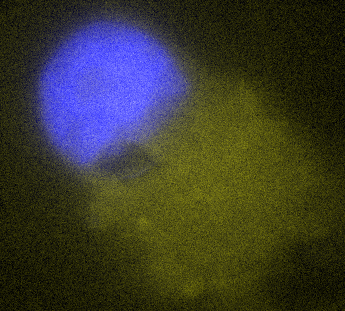

Supplement: Supplementary file 5 — Source Data for Figure 1 [file EMBJ-42-e113168-s008.zip › Figure 1/1A/Images/Replicate 3/32C/32C_S3r3__155.tif]

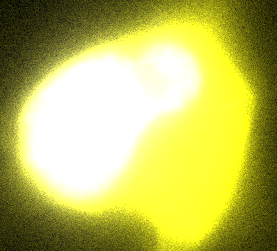

Supplement: Supplementary file 5 — Source Data for Figure 1 [file EMBJ-42-e113168-s008.zip › Figure 1/1A/Images/Replicate 3/32C/32C_S3r1__112.tif]

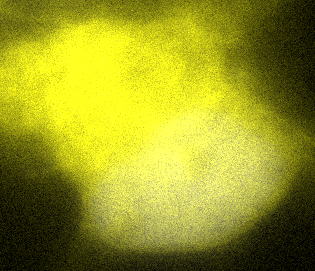

Supplement: Supplementary file 5 — Source Data for Figure 1 [file EMBJ-42-e113168-s008.zip › Figure 1/1A/Images/Replicate 3/32C/32C_S3r1__106.tif]

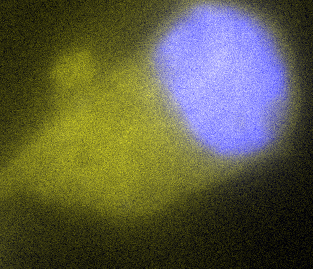

Supplement: Supplementary file 5 — Source Data for Figure 1 [file EMBJ-42-e113168-s008.zip › Figure 1/1A/Images/Replicate 3/32C/32C_S3r1__099.tif]

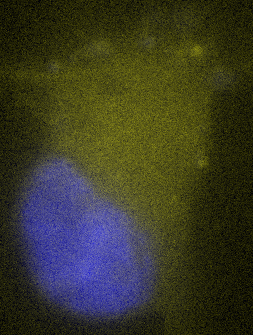

Supplement: Supplementary file 5 — Source Data for Figure 1 [file EMBJ-42-e113168-s008.zip › Figure 1/1A/Images/Replicate 3/32C/32C_S3r6__215.tif]

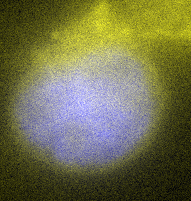

Supplement: Supplementary file 5 — Source Data for Figure 1 [file EMBJ-42-e113168-s008.zip › Figure 1/1A/Images/Replicate 3/32C/32C_S3r6__201.tif]

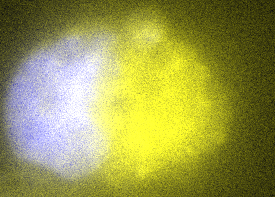

Supplement: Supplementary file 5 — Source Data for Figure 1 [file EMBJ-42-e113168-s008.zip › Figure 1/1A/Images/Replicate 3/32C/32C_S3r6__205.tif]

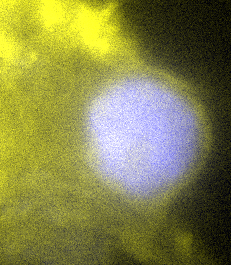

Supplement: Supplementary file 5 — Source Data for Figure 1 [file EMBJ-42-e113168-s008.zip › Figure 1/1A/Images/Replicate 3/32C/32C_S3r6__211.tif]

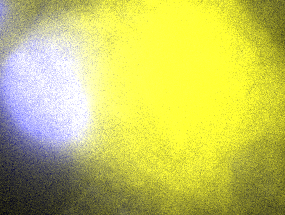

Supplement: Supplementary file 5 — Source Data for Figure 1 [file EMBJ-42-e113168-s008.zip › Figure 1/1A/Images/Replicate 3/32C/32C_S3r1__102.tif]

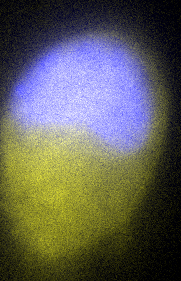

Supplement: Supplementary file 5 — Source Data for Figure 1 [file EMBJ-42-e113168-s008.zip › Figure 1/1A/Images/Replicate 3/32C/32C_S3r1__116.tif]

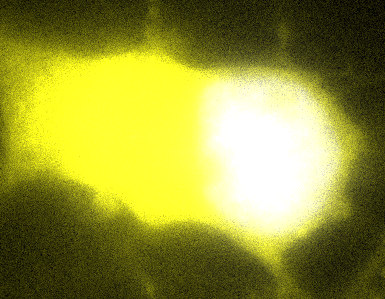

Supplement: Supplementary file 5 — Source Data for Figure 1 [file EMBJ-42-e113168-s008.zip › Figure 1/1A/Images/Replicate 3/32C/32C_S3r3__151.tif]

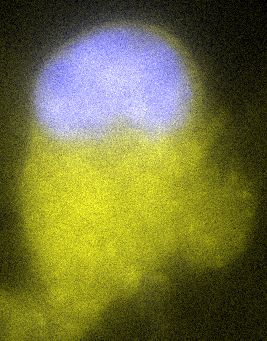

Supplement: Supplementary file 5 — Source Data for Figure 1 [file EMBJ-42-e113168-s008.zip › Figure 1/1A/Images/Replicate 3/32C/32C_S3r3__145.tif]

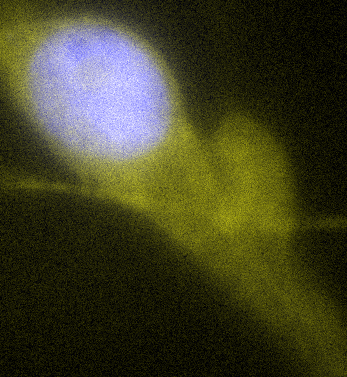

Supplement: Supplementary file 5 — Source Data for Figure 1 [file EMBJ-42-e113168-s008.zip › Figure 1/1A/Images/Replicate 3/32C/32C_S3r3__144.tif]

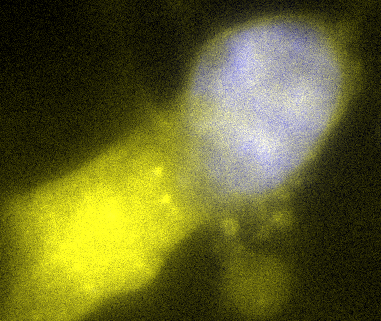

Supplement: Supplementary file 5 — Source Data for Figure 1 [file EMBJ-42-e113168-s008.zip › Figure 1/1A/Images/Replicate 3/32C/32C_S3r3__150.tif]

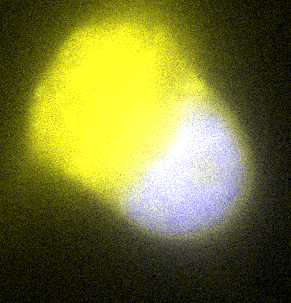

Supplement: Supplementary file 5 — Source Data for Figure 1 [file EMBJ-42-e113168-s008.zip › Figure 1/1A/Images/Replicate 3/32C/32C_S3r1__117.tif]

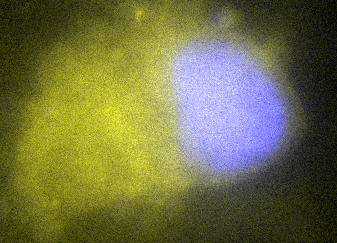

Supplement: Supplementary file 5 — Source Data for Figure 1 [file EMBJ-42-e113168-s008.zip › Figure 1/1A/Images/Replicate 3/32C/32C_S3r1__103.tif]
