# Supplementary material for: HNRNPH1 regulates the neuroprotective cold‐shock protein RBM3 expression through poison exon exclusion
Source: EMBO J. 2023 May 30;42(14):e113168. doi: 10.15252/embj.2022113168 (PMC10350819; doi:10.15252/embj.2022113168)
Supplement: Supplementary file 10 [file EMBJ-42-e113168-s005.pdf]

THE EMBO JOURNAL

HNRNPH1 regulates the neuroprotective cold-shock protein RBM3 expression through poison exon exclusion

Julie Qiaojin Lin, Deepak Khuperkar, Sofia Pavlou, Stanislaw Makarchuk, Nikolaos Patikas, Flora Lee, Julia Zbiegly, Jianning Kang, Sarah Field, David Bailey, Joshua Freeman, Jernej Ule, Emmanouil Metzakopian, Marc-David Ruepp, and Giovanna Mallucci
DOI: 10.15252/embj.2022113168

Corresponding author(s): Giovanna Mallucci (gmallucci@altoslabs.com) , Marc-David Ruepp (Marc-david.ruepp@kcl.ac.uk), Julie Qiaojin Lin (ql256@cam.ac.uk)

Review Timeline:

Submission Date:	29th Nov 22
Editorial Decision:	9th Jan 23
Revision Received:	28th Mar 23
Editorial Decision:	4th May 23
Revision Received:	7th May 23
Accepted:	11th May 23

Editor: Karin Dumstrei

Transaction Report:

(Note: With the exception of the correction of typographical or spelling errors that could be a source of ambiguity, letters and reports are not edited. Depending on transfer agreements, referee reports obtained elsewhere may or may not be included in this compilation. Referee reports are anonymous unless the Referee chooses to sign their reports.)

Dear Dr. Mallucci,

Thank you for submitting your manuscript to The EMBO Journal. Your study has now been seen by the three referees and the comments are provided below.

As you can see from the comments, the referees find the analysis interesting and insightful. Should you be able to add data to address the raised concerns then I would like to invite you to submit a revised manuscript.

I think it would be helpful to discuss the raised points further and I am available to do so via email or video.

When preparing your letter of response to the referees' comments, please bear in mind that this will form part of the Review Process File, and will therefore be available online to the community. For more details on our Transparent Editorial Process, please visit our website: https://www.embopress.org/page/journal/14602075/authorguide#transparentprocess

Thank you for the opportunity to consider your work for publication. I look forward to your revision.

With best wishes

Karin

Karin Dumstrei, PhD
Senior Editor
The EMBO Journal

Instructions for preparing your revised manuscript:

Please make sure you upload a letter of response to the referees' comments together with the revised manuscript.

Please also check that the title and abstract of the manuscript are brief, yet explicit, even to non-specialists.

When assembling figures, please refer to our figure preparation guideline in order to ensure proper formatting and readability in print as well as on screen:
https://bit.ly/EMBOPressFigurePreparationGuideline
See also guidelines for figure legends: https://www.embopress.org/page/journal/14602075/authorguide#figureformat

At EMBO Press we ask authors to provide source data for the main manuscript figures. Our source data coordinator will contact you to discuss which figure panels we would need source data for and will also provide you with helpful tips on how to upload and organize the files.

IMPORTANT: When you send the revision we will require
- a point-by-point response to the referees' comments, with a detailed description of the changes made (as a word file).
- a word file of the manuscript text.
- individual production quality figure files (one file per figure)
- a complete author checklist, which you can download from our author guidelines (https://www.embopress.org/page/journal/14602075/authorguide).
- Expanded View files (replacing Supplementary Information)
Please see out instructions to authors
https://www.embopress.org/page/journal/14602075/authorguide#expandedview

Please remember: Digital image enhancement is acceptable practice, as long as it accurately represents the original data and conforms to community standards. If a figure has been subjected to significant electronic manipulation, this must be noted in the figure legend or in the 'Materials and Methods' section. The editors reserve the right to request original versions of figures and the original images that were used to assemble the figure.

Further information is available in our Guide For Authors: https://www.embopress.org/page/journal/14602075/authorguide

We realize that it is difficult to revise to a specific deadline. In the interest of protecting the conceptual advance provided by the work, we recommend a revision within 3 months (9th Apr 2023).

As a matter of policy, competing manuscripts published during this period will not negatively impact on our assessment of the conceptual advance presented by your study. However, we request that you contact the editor as soon as possible upon publication of any related work, to discuss how to proceed. Should you foresee a problem in meeting this three-month deadline, please let us know in advance and we may be able to grant an extension.

Referee #1:

General summary:

In this manuscript the authors elucidate the molecular mechanism by which the neuroprotective cold shock RNA binding protein RBM3 is induced during cooling. Performing a genome-wide CRISPR-Cas9 knockout screen in human iPSC-derived neurons they identified heterogeneous nuclear ribonucleoprotein H1 (HNRNPH1) as a strong positive regulator of RBM3 protein levels. Using an array of different experiments and analyses they further showed convincingly that HNRNPH1 represses the inclusion of a poison cassette exon (PCE) during hypothermia via its interaction with a G-rich motif within the PCE. In contrast, inclusion of this PCE during normal temperatures targets the transcript for nonsense-mediated decay. Their findings are of broad interest and increase the range of therapeutic targets for RBM3 induction and neuroprotection without cooling.

General comments:

There is a discrepancy between the results of the screen where KO of HNRNPH1 significantly reduced GFP-RBM3 levels at 37{degree sign}C and at 32{degree sign}C and the results with the poison exon inclusion where HNRNPH1 binding to the GGG tract and PE suppression is only effective at 32 degrees. Also, the experiments with ActD and CHX work similarly at both temperatures. The authors should discuss how the levels of RBM3 are reduced at 37{degree sign}C by HNRNPH1?

The authors did nor further pursue what mediates the difference in HNRNPH1 activity between 37{degree sign}C and 32{degree sign}C. Actually, in Figure S4F the IPs and its quantification suggest that HNRNPH1 binds less to snRNPs at 32 degrees. To complete the story, the authors could test binding of HNRNPH1 to the RBM3 transcript at both temperatures by RIP? They could also compare PTM levels, changes in subcellular localization or the formation of foci at the different temperatures with their GFP reporter line.

Specific comments:

The first paragraph is not well understandable for non-experts and lacks some information. The GFP reporter cell line and its induction to i-neurons is not enough motivated and also insufficiently described. Why was the reporter done like this? How were the neurons induced, how were clones generated and selected. How were the lentiviral library transduced during differentiation, how was the editing efficiency measured, what is a BFP reporter and what are the controls in the screen.
Page 9: Please clarify this sentence: Particularly, both the mRNA and protein levels of RBM3 were most significantly reduced across the whole genome upon HNRNPH1 KD in HeLa cells.

Referee #2:

In this study, the authors investigate the molecular mechanism behind the cold-dependent upregulation of the neuroprotective protein RBM3. It is known that RBM3 is upregulated in response to cold and that this increase in RBM3 levels contributes to the neuroprotective effect of lowering the brain temperature after stroke or brain injuries. However, the molecular mechanism triggering this regulation was not known.
Using an unbiased Crispr screen, the authors reveal that RBM3 protein level is strongly regulated by spliceosomal components and splicing factors. Specifically, HNRNPH1 has a significant effect on RBM3 mRNA and protein levels and its regulation by cold. In response to cold, HNRNP1 prevents the inclusion of a poison exon that targets Rbm3 transcripts for NMD-dependent degradation. The authors' data support that HNRNPH1 is recruited at a GGGG motif within the poison exon.
Overall, this is a well-designed and rigorous study, and the conclusions are strongly supported by the data. The findings also bring new insights to manipulate RBM3 expression for neuroprotection in clinical interventions.

Major points:

- With their Crispr-screen, the authors show that RBM3 regulation is happening at multiple levels beyond splicing, including

translation.
To further strengthen the study, it would be important to evaluate to what extent HNRNPH1 and the poison exon regulation contribute to the elevation of Rbm3 mRNAs and protein levels in response to low temperature. This question could be addressed using the minigene approach described in the study.

- The link between HNRNP1 and the GGGG motif should be strengthened.
Is the association of HNRNPH1 at the Rbm3 poison exon higher when lowering the temperature (Fig4H)?
It would also be helpful to demonstrate that, as expected, the overexpression of HNRNPH1 does not revert the inclusion of the poison exon in minigene with GGGG deletion (Figs 4G, 4I).

Minor points:

- The efficiency of hnRNPH1 knockout at the protein level in i-neurons should be controlled.

- Fig1: it was not clear to me how was assessed the editing efficiency: is it based on BFP positive cells?

- FigS1: typos in the legend (D) instead of (E) and (D) missing

- Figs 3 and 4: When PSI are plotted, the values should be indicated in percentage.

- In Figs 2 and 4: in i-neurons HNRNP1 appears to affect RBM3 expression (RNA and protein) at both 37{degree sign}C and 32{degree sign}C while in HeLa cells the effect is almost exclusively observed at 32{degree sign}C. This difference should be discussed.

Referee #3:

In this paper, Lin et al. shed some light on the mechanism by which the cold-protective protein RBM3 is induced upon hypothermia. Identifying the molecular effectors involved in RBM3 overexpression in situations of stress, including hypothermia, is of great relevance to better understand neuroprotection and possibly identifying therapeutic targets. The authors perform a very elegant knock-out screen, using GFP intensity as a readout of RBM3 expression, in i-neurons, and identify several negative and positive regulators. Interestingly, the top regulators are splicing factors, with the protein HNRNPH1 standing out as the strongest positive regulator candidate of RBM3 induction. The authors show that HNRNPH1 is required for high RNA and protein expression of an RBM3 reporter; that the expression of a poison exon, 3a-L/S, which would target the mRNA for NMD, is repressed in the cold, and that this cold-induced exon skipping is mediated by HNRNPH1. The findings are interesting and represent an important contribution to our knowledge on the mechanisms of neuroprotection. The paper is well written and excellent data support the conclusions. However, I think that the physiological relevance of this mechanism and how it is embedded in the process of neuroprotection is not clearly demonstrated.

Major points:

1) It is not clear that the overexpression of RBM3 RNA and protein is due to the mechanism presented in this paper. The authors should show the contribution of NMD inhibition to RBM3 overexpression: indeed, this aspect is key to understand whether the identified mechanism has an impact on neuroprotection. Transcription of RBM3 seems upregulated upon cold stress as well. The data shown indicate that few transcripts possess a poison exon (Fig. 3A), even upon NMD inactivation (Fig. 3D), raising the question whether HNRNPH1-induced exon skipping actually matters for RBM3 induction.
For example, RNA levels of RBM3 upon ActD treatment could be shown to estimate how much RNA degradation (vs. transcription) contributes to RBM3 expression, in normal and cold conditions. Transcriptional activation of RBM3 can also be measured by qPCR, using exon-intron spanning primers, or using RNA sequencing technologies that detect nascent RNAs.

2) The effect of HNRNPH1 on RBM3 expression occurs in cold as well as in warm conditions (Fig. 3E), and HNRNPH1 is not induced upon cold (Fig. S4), which raises the question of the importance of the mechanism in cold induction vs. as a general mechanism to eliminate aberrant RBM3 transcripts. The authors should show more direct evidence that the mechanism they describe is relevant for the cold response.

3) The authors' model would be more convincing if they clearly showed that HNRNPH1 overexpression causes an upregulation of RBM3 RNA and protein levels in wild-type (not reporter) neurons. Likewise, knockdown should cause RBM3 RNA and protein downregulation. These quantifications should all be done in cold and warm conditions, and/or mentioned more explicitly.

Minor points:

a) Does the very mild (1.3-fold) increase in the GFP-RBM3 protein upon cold-shock represent the physiological situation? The authors show that RBM3 RNA increases 4-5-fold in wild-type cells, the same should be done for RBM3 protein levels.

b) Statistical tests and important information are missing for several figure panels (starting with Fig. 1A).

c) Fig. 2A, B: Why was a different control (non-targeting and reporter) used in panels A and B to normalize levels? The same control should be used, or a valid reason stated.

d) Fig. S1C: how is the editing efficiency calculated? Is this actually transfection efficiency? This should be stated in the legend (i.e., proportion of BFP+ cells) as well as in the Methods section. Similarly, in Fig. 1A, what does one dot represent?

e) The legends in Fig. S1D, E are shifted.

f) Fig. 1F, G: All significant GO terms should be listed in a supplemental table. Also, the figure legend 1F states GO terms for 14 positive regulators, whereas there are 15 (page 6).

g) Fig. 4: PCR gels are not quantitative; the panels should either include qRT-PCR experiments, or only show the (quite convincing on their own) qualitative gels.

h) On page 12, last paragraph of the Results section, it is speculated that HNRNPH1 destabilizes G-structures, which may cause exon skipping. Following this thought, deleting the G-rich sequence should also disrupt this structure and thereby mimic HNRNP1 binding, however the opposite result is found (exon inclusion, Fig. 4I). The authors should reconcile their observations with the model, or abandon the rG4 hypothesis.

Comments/suggestions:

- Typo (?) in Fig. 2, $p<0.5$, do the authors mean $p<0.05$?

- Fig. 3B: it would be nice to display the (unchanged) PSI of a constitutive exon, for comparison.

Referee #1

General summary:

In this manuscript the authors elucidate the molecular mechanism by which the neuroprotective cold shock RNA binding protein RBM3 is induced during cooling. Performing a genome-wide CRISPR-Cas9 knockout screen in human iPSC-derived neurons they identified heterogeneous nuclear ribonucleoprotein H1 (HNRNPH1) as a strong positive regulator of RBM3 protein levels. Using an array of different experiments and analyses they further showed convincingly that HNRNPH1 represses the inclusion of a poison cassette exon (PCE) during hypothermia via its interaction with a G-rich motif within the PCE. In contrast, inclusion of this PCE during normal temperatures targets the transcript for nonsense-mediated decay. ***Their findings are of broad interest and increase the range of therapeutic targets for RBM3 induction and neuroprotection without cooling.***

General comments:

There is a discrepancy between the results of the screen where KO of HNRNPH1 significantly reduced GFP-RBM3 levels at 37°C and at 32°C and the results with the poison exon inclusion where HNRNPH1 binding to the GGG tract and PE suppression is only effective at 32 degrees. Also, the experiments with ActD and CHX work similarly at both temperatures. The authors should discuss how the levels of RBM3 are reduced at 37°C by HNRNPH1?

The reviewer is correct that our data suggest that HNRNPH1 knockdown leads to a proportionally similar reduction in RBM3 expression at the two temperatures: a) in reporter i-neurons, ~35% decrease in RBM3 protein (**Fig 2A**) and 20-25% decrease in RBM3 mRNA at both 37°C and 32°C (**Figs 2D and 2E**); b) in HeLa cells, 55-60% decrease in RBM3 mRNA at both temperatures (**Fig EV2I**).

However, we show that HNRNPH1-mediated poison exon exclusion is more effective at 32°C: a) in wild-type i-neurons, poison exon inclusion increased by 30% at 37°C and **>50% at 32°C** upon HNRNPH1 knockdown (**Figs 4A and 4B**); b) in HeLa cells, no change at 37°C compared to 2-fold increase at 32°C (**Figs 4D and 4E**).

This discrepancy may be explained by factors fine-tuning RBM3 expression at additional levels of regulation, especially RNA degradation via NMD. We did not assess the temperature-dependent capacity of NMD in this study, and the altered NMD efficacy may account for the differences between HNRNPH1-regulated RBM3 poison exon inclusion and RBM3 expression at different temperatures.

We have included a section on this possibility in the discussion (Page 11): *The discrepancy between the temperature-independent regulation of RBM3 expression and the temperature-dependent control of RBM3 PE inclusion by HNRNPH1 may be a result of non-splicing regulatory factors, such as the NMD pathway, which may also function with different capacity in a temperature-sensitive manner.*

The authors did not further pursue what mediates the difference in HNRNPH1 activity between 37°C and 32°C. Actually, in Figure S4F the IPs and its quantification suggest that HNRNPH1 binds less to snRNPs at 32 degrees. To complete the story, the authors could test binding of HNRNPH1 to the RBM3 transcript at both temperatures by RIP? They could also compare PTM levels, changes in subcellular localization or the formation of foci at the different temperatures with their GFP reporter line.

We thank the reviewer for the experimental suggestions to further investigate mechanisms mediating temperature-dependent HNRNPH1 activities. We now include new data from HNRNPH1 RIP and HNRNPH1 immunostaining to provide additional mechanisms in the revised manuscript:

1) HNRNPH1 RIP followed by qRT-PCR detection of RBM3 mRNA levels reveals stronger HNRNPH1-RBM3 mRNA interaction at 32°C compared to 37°C (new **Fig 5B**), providing a plausible explanation for a stronger impact of HNRNPH1 on RBM3 poison exon inclusion at lower temperatures.

2) HNRNPH1 staining did not show any change in nuclear or cytoplasmic HNRNPH1 localisation at 32°C compared to 37°C (new **Fig EV5C**), confirming that the cold-enhanced HNRNPH1 splicing activity was not due to an increase in HNRNPH1 nuclear localisation.

We agree that examining the PTM levels and foci formation will be valuable to understand the temperature-dependent splicing regulatory functions of HNRNPH1, which will be the focus of our next in-depth mechanistic study and we include in the discussion.

Specific comments:

The first paragraph is not well understandable for non-experts and lacks some information. The GFP reporter cell line and its induction to i-neurons is not enough motivated and also insufficiently described. Why was the reporter done like this? How were the neurons induced, how were clones generated and selected. How were the lentiviral library transduced during differentiation, how was the editing efficiency measured, what is a BFP reporter and what are the controls in the screen.

The revised manuscript contains the following information to help readers understand our experimental design of the CRISPR screen:

1) The reason to generate a fluorescent RBM3 reporter iPSC line and the method of its neuronal differentiation are included in the Results (Page 4). Detailed differentiation protocol is available in Methods (Page 13), under “iPSC differentiation into iPSC-derived neurons (i-neuron)”.

2) Generation of reporter iPSC clones and selection are described step-wise in Methods (Page 16), under “Generation of GFP-RBM3 iPSCs by CRISPR”.

3) The protocol of the pooled screen, including lentiviral library transduction, is available in Methods (Page 19), under “RBM3 CRISPR knockout screen”.

4) The measurement of editing efficiency using the reporter lentivirus is described in the revised Results (Page 4) and in new **Figs EV1C-E**.

5) The control of the screen is the non-targeting guide RNAs, which is described in Methods (Page 24), under “Whole-genome CRISPR screen next-generation sequencing analysis”.

Page 9: Please clarify this sentence: Particularly, both the mRNA and protein levels of RBM3 were most significantly reduced across the whole genome upon HNRNPH1 KD in HeLa cells.

This sentence (revised Page 7) is replaced with “*In HNRNPH1-knocked down HeLa cells, RBM3 is among the top 5% downregulated genes detected by RNA-Seq and proteomics, with its mRNA and protein levels decreased by 3 and 0.55 folds, respectively.*”

Referee #2

In this study, the authors investigate the molecular mechanism behind the cold-dependent upregulation of the neuroprotective protein RBM3. It is known that RBM3 is upregulated in response to cold and that this increase in RBM3 levels contributes to the neuroprotective effect of lowering the brain temperature after stroke or brain injuries. However, the molecular mechanism triggering this regulation was not known.
Using an unbiased Crispr screen, the authors reveal that RBM3 protein level is strongly regulated by spliceosomal components and splicing factors. Specifically, HNRNPH1 has a significant effect on RBM3 mRNA and protein levels and its regulation by cold. In response to cold, HNRNP1 prevents the inclusion of a poison exon that targets Rbm3 transcripts for NMD-dependent degradation. The authors' data support that HNRNPH1 is recruited at a GGGG motif within the poison exon. ***Overall, this is a well-designed and rigorous study, and the conclusions are strongly supported by the data. The findings also bring new insights to manipulate RBM3 expression for neuroprotection in clinical interventions.***

Major points:

- With their Crispr-screen, the authors show that RBM3 regulation is happening at multiple levels beyond splicing, including translation. To further strengthen the study, it would be important to evaluate to what extent HNRNPH1 and the poison exon regulation contribute to the elevation of Rbm3 mRNAs and protein levels in response to low temperature. This question could be addressed using the minigene approach described in the study.

We would like to refer the reviewer to our parallel study (Preußner et al., ASO targeting temperature-controlled RBM3 poison exon splicing prevents neurodegeneration in vivo, 2023, EMBO Mol Med). In Figs 2B and 2C (shown below) of this paper, the deletion of RBM3 poison exon resulted in constantly high levels of RBM3 mRNA and protein expression at 35-39°C, which were no longer temperature-sensitive. This observation confirms that poison exon regulation is the determinant of temperature-dependent RBM3 expression.

Preußner et al., 2023, EMBO Mol Med, Figure 2:

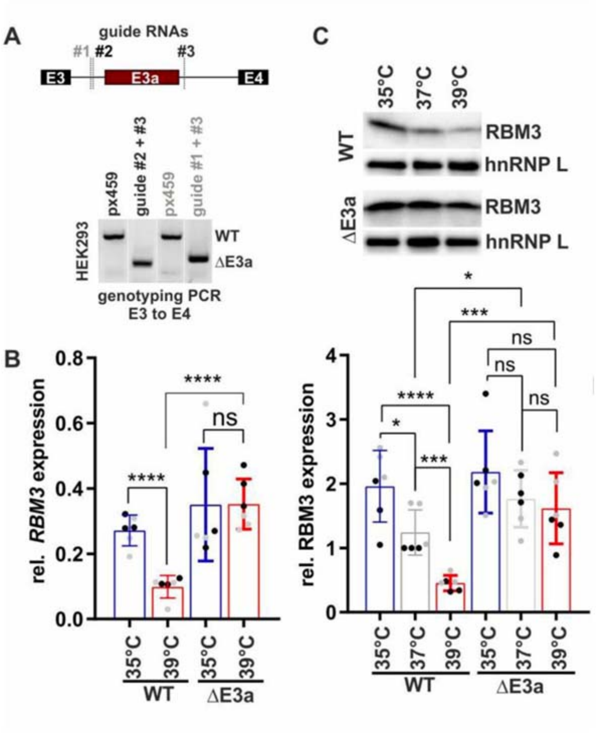

A.CRISPR/CAS9-mediated removal of RBM3 E3a*. One of two guide RNAs targeting the upstream intron (#1, #2) was co-transfected with a guide RNA targeting the downstream intron (#3). Below, genotyping PCR after clonal selection with px459 transfected cells serving as a negative control. See also Fig EV2A.*
B, C.RT–qPCR (B) and Western blot (C) analysis of RBM3 levels in edited cell lines*. Clonal cell lines from A were incubated at the indicated temperatures for 24 h. In (B), isolated RNA was investigated by qPCR, and RBM3 expression is shown relative to GAPDH levels. In (C), lysates from an independent experiment were investigated for RBM3 protein expression, hnRNP L served as a loading control, a representative gel (top) and quantification (bottom) are shown (mean ± s.d., n = 6 [n = 3 per clone, indicated in black/gray], all individual data points are shown).*

- The link between HNRNP1 and the GGGG motif should be strengthened.
Is the association of HNRNPH1 at the Rbm3 poison exon higher when lowering the temperature (Fig4H)?

We have performed HNRNPH1 RNA immunoprecipitation (RIP) at 37°C and 32°C, followed by qRT-PCR detection of RBM3 mRNA levels, which reveals stronger HNRNPH1-RBM3 mRNA interaction at 32°C compared to 37°C (new **Fig 5B**), providing a plausible explanation for a stronger impact of HNRNPH1 on RBM3 poison exon inclusion at lower temperatures.

(Note: the original Fig 4H is the revised Fig 5C)

It would also be helpful to demonstrate that, as expected, the overexpression of HNRNPH1 does not revert the inclusion of the poison exon in minigene with GGGG deletion (Figs 4G, 4I).

We performed this experiment as suggested, and indeed, the overexpression of HNRNPH1 did not significantly change the poison exon inclusion in the delGGGG minigene (new **Fig EV5G**).

(Note: the original Fig 4I is the revised Fig 5D)

Minor points:

- The efficiency of hnRNPH1 knockout at the protein level in i-neurons should be controlled.

In Fig. 2A and 2B, only BFP-positive (successfully transduced) cells are included in the analysis and the editing efficiency is estimated to be 60-70% (**Fig EV1E**). 75% decrease in HNRNPH1 protein levels is shown by western blot (new **Fig EV4A**).

- Fig1: it was not clear to me how was assessed the editing efficiency: is it based on BFP positive cells?

We now include schematics and flow cytometry data (new **Figs EV1C-E**) to explain how the editing efficiency is calculated, which is different from the transduction efficiency (percentage of BFP-positive cells). Briefly, i-neurons 4 days post differentiation were transduced with a reporter lentivirus expressing BFP, mCherry and mCherry-targeted sgRNA. Cells successfully edited by mCherry sgRNA showed decreased mCherry intensity compared to unedited cells. Therefore, the editing efficiency is measured to be the percentage of BFP-positive cells with reduced mCherry expression among all BFP-positive cells.

- FigS1: typos in the legend (D) instead of (E) and (D) missing

Corrected.

- Figs 3 and 4: When PSI are plotted, the values should be indicated in percentage.

Corrected in all figures.

- In Figs 2 and 4: in i-neurons HNRNPH1 appears to affect RBM3 expression (RNA and protein) at both 37°C and 32°C while in HeLa cells the effect is almost exclusively observed at 32°C. This difference should be discussed.

HNRNPH1 KD affects RBM3 protein and mRNA expression both at 37°C and 32°C in i-neurons (**Figs 2A, D and E**) as well as mRNA expression in HeLa cells (**Fig EV2I**). The reviewer may be referring to the poison exon inclusion discrepancy upon HNRNPH1 KD at 37°C between i-neurons and HeLa. In i-neurons, poison exon inclusion increased by 30% at 37°C and >50% at 32°C (**Figs 4A and B**). But no difference was seen at 37°C upon HNRNPH1 KD in HeLa cells (**Figs 4D and E**). The discrepancy between HNRNPH1-mediated RBM3 poison exon inclusion and RBM3 expression at different temperatures may be explained by factors fine-tuning RBM3 expression at additional levels of regulation, especially RNA degradation via NMD. We did not assess the temperature-dependent capacity of NMD in this study, and the altered NMD efficacy may account for the differences.

We have included a section on this possibility in the discussion (Page 11): *The discrepancy between the temperature-independent regulation of RBM3 expression and the temperature-dependent control of RBM3 PE inclusion by HNRNPH1 may be a result of non-splicing regulatory factors, such as the NMD pathway, which may also function with different capacity in a temperature-sensitive manner.*

Referee #3

In this paper, Lin et al. shed some light on the mechanism by which the cold-protective protein RBM3 is induced upon hypothermia. Identifying the molecular effectors involved in RBM3 overexpression in situations of stress, including hypothermia, is of great relevance to better understand neuroprotection and possibly identifying therapeutic targets. The authors perform a very elegant knock-out screen, using GFP intensity as a readout of RBM3 expression, in i-neurons, and identify several negative and positive regulators. Interestingly, the top regulators are splicing factors, with the protein HNRNPH1 standing out as the strongest positive regulator candidate of RBM3 induction. The authors show that HNRNPH1 is required for high RNA and protein expression of an RBM3 reporter; that the expression of a poison exon, 3a-L/S, which would target the mRNA for NMD, is repressed in the cold, and that this cold-induced exon skipping is mediated by HNRNPH1. ***The findings are interesting and represent an important contribution to our knowledge on the mechanisms of neuroprotection. The paper is well written and excellent data support the conclusions.***

However, I think that the physiological relevance of this mechanism and how it is embedded in the process of neuroprotection is not clearly demonstrated.

We address this last general comment below under Major point 1.

Major points:

1) It is not clear that the overexpression of RBM3 RNA and protein is due to the mechanism presented in this paper. The authors should show the contribution of NMD inhibition to RBM3 overexpression: indeed, this aspect is key to understand whether the identified mechanism has an impact on neuroprotection.

We addressed the neuroprotective role of RBM3 poison exon skipping in a parallel study (Preußner et al., ASO targeting temperature-controlled RBM3 poison exon splicing prevents neurodegeneration in vivo, 2023, EMBO Mol Med). We showed that ASO-mediated RBM3 poison exon exclusion resulted in increased RBM3 expression, which was profoundly neuroprotective in a neurodegenerative disease mouse model.

Preußner et al., 2023, EMBO Mol Med, Synopsis:

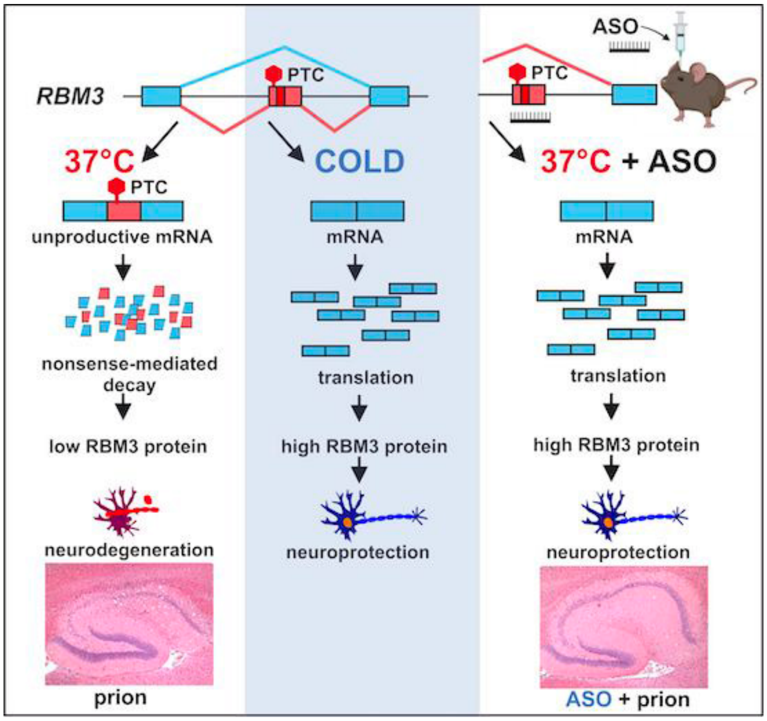

Transcription of RBM3 seems upregulated upon cold stress as well. The data shown indicate that few transcripts possess a poison exon (Fig. 3A), even upon NMD inactivation (Fig. 3D), raising the question whether HNRNPH1-induced exon skipping actually matters for RBM3 induction. For example, RNA levels of RBM3 upon ActD treatment could be shown to estimate how much RNA degradation (vs. transcription) contributes to RBM3 expression, in normal and cold conditions. Transcriptional activation of RBM3 can also be measured by qPCR, using exon-intron spanning primers, or using RNA sequencing technologies that detect nascent RNAs.

In this study, we showed splicing and NMD-mediated mRNA degradation together control the final RBM3 mRNA expression. SMG1 (NMD) inhibitor was used to study the effect of temperature or HNRNPH1-regulated splicing without the influence of degradation. But the subsequent mRNA degradation of the poison exon-containing isoform determines the temperature-sensitive RBM3 mRNA levels. Therefore, we believe that degradation is also key to regulating temperature-dependent RBM3 expression.

As suggested by the reviewer, we examined the potential transcriptional activation of RBM3 on cooling by qRT-PCR of RBM3 pre-mRNA (Exon 2-Intron 2) and showed that RBM3 pre-mRNA levels were not changed between 37°C and 32°C (new **Figs EV2E and EV2G**). We thus excluded the contribution of transcriptional regulation to RBM3 cold induction.

As supporting evidence for the critical role of poison exon in temperature-dependent RBM3 expression, Figs 2B and 2C of the above-mentioned *Preußner et al., 2023, EMBO Mol Med* (shown below) show that the deletion of RBM3 poison exon resulted in constantly high levels

of RBM3 mRNA and protein expression at 35-39°C, which were no longer temperature-dependent for expression.

Preußner et al., 2023, EMBO Mol Med, Figure 2:

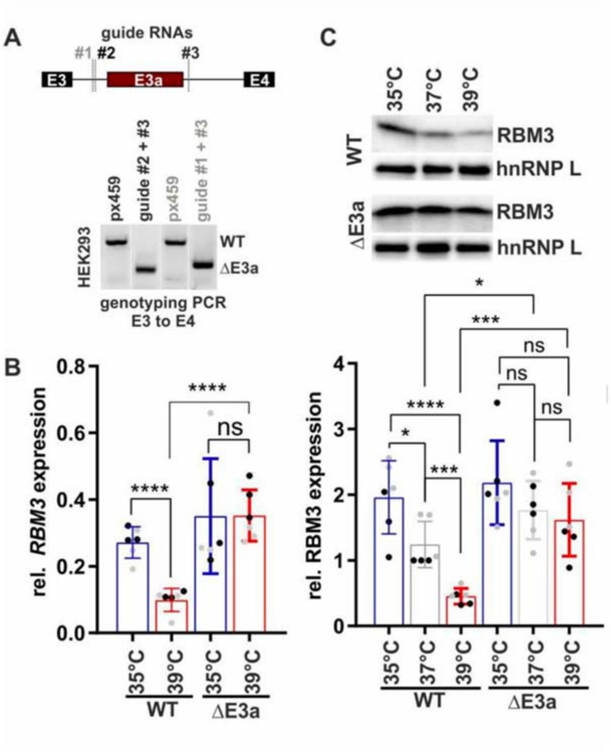

***A. CRISPR/CAS9-mediated removal of RBM3 E3a**. One of two guide RNAs targeting the upstream intron (#1, #2) was co-transfected with a guide RNA targeting the downstream intron (#3). Below, genotyping PCR after clonal selection with px459 transfected cells serving as a negative control. See also Fig EV2A.*
***B, C. RT–qPCR (B) and Western blot (C) analysis of RBM3 levels in edited cell lines**. Clonal cell lines from A were incubated at the indicated temperatures for 24 h. In (B), isolated RNA was investigated by qPCR, and RBM3 expression is shown relative to GAPDH levels. In (C), lysates from an independent experiment were investigated for RBM3 protein expression, hnRNP L served as a loading control, a representative gel (top) and quantification (bottom) are shown (mean ± s.d., n = 6 [n = 3 per clone, indicated in black/gray], all individual data points are shown).*

2) The effect of HNRNPH1 on RBM3 expression occurs in cold as well as in warm conditions (Fig. 3E), and HNRNPH1 is not induced upon cold (Fig. S4), which raises the question of the importance of the mechanism in cold induction vs. as a general mechanism to eliminate aberrant RBM3 transcripts. The authors should show more direct evidence that the mechanism they describe is relevant for the cold response.

To provide more direct evidence, we performed HNRNPH1 RNA immunoprecipitation (RIP) at 37°C and 32°C, followed by qRT-PCR detection of RBM3 mRNA levels, which revealed stronger HNRNPH1-RBM3 mRNA interaction at 32°C compared to 37°C (new **Fig 5B**). Although HNRNPH1 interacts with RBM3 to regulate its splicing at 37°C, this observation supports a strengthened physical interaction between HNRNPH1 and RBM3 mRNA at the lower temperature.

(Note: the original Fig S4 is the revised Figs EV4 and EV5)

3) The authors' model would be more convincing if they clearly showed that HNRNPH1 overexpression causes an upregulation of RBM3 RNA and protein levels in wild-type (not reporter) neurons. Likewise, knockdown should cause RBM3 RNA and protein downregulation. These quantifications should all be done in cold and warm conditions, and/or mentioned more explicitly.

We examined the effect of HNRNPH1 overexpression on RBM3 mRNA and protein expression in wild-type HeLa, HEK293T and i-neurons. Although we observed significant poison exon exclusion upon HNRNPH1 overexpression in endogenous RBM3 transcripts in HEK cells (**Fig 4F**) and i-neurons (**Fig 4H**) and in minigene expressed in HEK cells (**Fig 4G**), we were not able to detect any change of RBM3 mRNA and protein levels using qRT-PCR and western blot. We think this may be because the high abundance of endogenous HNRNPH1 and a moderate level of HNRNPH1 overexpression by transfection or lentiviral transduction was not sufficient to elevate RBM3 expression that is visible using a bulk detection method like qRT-PCR and western blot.

To overcome this obstacle, we generated lentivirus with a BFP reporter for HNRNPH1 overexpression in GFP-RBM3 i-neurons (new **Fig 4H**), so we can quantify the GFP-RBM3 expression in i-neurons with high levels of exogenous HNRNPH1 expression using flow cytometry providing single cell readings. HNRNPH1-T2A-BFP-expressing i-neurons showed a mild but significant increase of GFP-RBM3 at 37°C and 32°C compared to the control cells expressing BFP (new **Fig EV4I**). In fact, a close examination of GFP/BFP correlation plots reveals that only i-neurons expressing a high level of HNRNPH1-T2A-BFP (the coloured dots) showed large upregulation of GFP-RBM3 (new **Figs 4I and EV4J**), which indicates that high levels of HNRNPH1 overexpression promote RBM3 expression.

We have shown that HNRNPH1 KD reduces RBM3 mRNA in wild-type i-neurons at 37°C and 32°C (**Fig 2D**) and reduces GFP-RBM3 protein levels in the reporter i-neurons (**Fig 2A**). We specify the temperature condition of all figures in the revised legends (e.g. **Fig 4F, 4G**).

Minor points:

a) Does the very mild (1.3-fold) increase in the GFP-RBM3 protein upon cold-shock represent the physiological situation? The authors show that RBM3 RNA increases 4-5-fold in wild-type cells, the same should be done for RBM3 protein levels.

In WT i-neurons, RBM3 protein levels increased by ~50% on cooling (new **Fig EV1H**), which is comparable to the ~50% increase in primary hippocampal neuron cultures after 24h cooling at 32°C (Peretti *et al.*, 2021, Life Science Alliance, Fig. 3A). The attenuated mRNA translation activity at lower temperatures may account for the milder RBM3 protein cold induction compared to its mRNA.

b) Statistical tests and important information are missing for several figure panels (starting with Fig. 1A).

We thank the reviewer for noticing them. We have now added all statistical information in the figure legends.

c) Fig. 2A, B: Why was a different control (non-targeting and reporter) used in panels A and B to normalize levels? The same control should be used, or a valid reason stated.

Since we observed differences between the two controls, we chose the more stringent control for identifying the negative or positive regulators. We have provided an explanation for this in the main text (Page 6): *"Moderate spectral crossover and activation of specific signalling pathways due to target-specific genome editing may account for the discrepancy between non-targeting sgRNA and reporter controls. To apply a more stringent standard, we performed statistical analysis between specific gene knockout (KO) groups and one of the two control groups with high p values, for instance, comparing to the non-targeting sgRNA control showing lower GFP intensity to identify positive regulators, and to the reporter control for negative regulators."*

d) Fig. S1C: how is the editing efficiency calculated? Is this actually transfection efficiency? This should be stated in the legend (i.e., proportion of BFP+ cells) as well as in the Methods section. Similarly, in Fig. 1A, what does one dot represent?

We now include schematics and flow cytometry data (new **Figs EV1C-E**) to explain how the editing efficiency is calculated, which is different from the transduction efficiency (percentage of BFP-positive cells). Briefly, i-neurons 4 days post differentiation were transduced with a reporter lentivirus expressing BFP, mCherry and mCherry-targeted sgRNA. Cells successfully edited by mCherry sgRNA showed decreased mCherry intensity compared to unedited cells. Therefore, the editing efficiency is measured to be the percentage of BFP-positive cells with reduced mCherry expression among all BFP-positive cells.

Measurement and calculation of editing and transduction efficiencies are described in the Results (Page 4) and the legends of **Fig EV1C-E**. Additional information is provided in the Methods, under the "Flow cytometry" section (Page 18).

Each dot in **Fig 1A** (also **Figs EV1G, EV2A and EV5C**) represents a measurement from one cell from one of the 3 biological replicates.

e) The legends in Fig. S1D, E are shifted.

Corrected.

f) Fig. 1F, G: All significant GO terms should be listed in a supplemental table. Also, the figure legend 1F states GO terms for 14 positive regulators, whereas there are 15 (page 6).

All significant GO terms are now included in new **Appendix Table S3**. One of the 15 is RBM3 itself, which we did not include in the GO analysis - we specify this in the revised legend of **Fig 1F**.

g) Fig. 4: PCR gels are not quantitative; the panels should either include qRT-PCR experiments, or only show the (quite convincing on their own) qualitative gels.

We acknowledge that gel-based RT-PCR, similar to western blots, are only semi-quantitative. However, we wish to keep the quantification along with the gel images to show the result of all biological replicates.

h) On page 12, last paragraph of the Results section, it is speculated that HNRNPH1 destabilizes G-structures, which may cause exon skipping. Following this thought, deleting the G-rich sequence should also disrupt this structure and thereby mimic HNRNP1 binding, however the opposite result is found (exon inclusion, Fig. 4I). The authors should reconcile their observations with the model, or abandon the rG4 hypothesis.

We thank the reviewer for pointing out the oversight of our proposed model, and we agree that the rG4 hypothesis needs to be discarded for now. We have revised this section by removing the last Results paragraph, which contained the hypothesis that HNRNPH1 destabilizes rG4s at low temperatures. Instead, we discuss alternative hypotheses in the Discussion (Page 11) to encourage further investigation into the temperature-sensitive roles of rG4s and rG4-binding proteins in RBM3 poison exon regulation and universal RNA splicing.

(Note: the original Fig 4I is the revised Fig 5D)

Comments/suggestions:

- Typo (?) in Fig. 2, p<0.5, do the authors mean p<0.05?

All corrected.

- Fig. 3B: it would be nice to display the (unchanged) PSI of a constitutive exon, for comparison.

The PSI of the constitutive Exon 3 (revised **Fig 3B**), and Exon 4 and 5 (new **Fig EV3A**) are now shown. Additional PSI values are shown numerically (**Appendix Table S6**).

1st Revision - Editorial Decision 4th May 2023

Dear Dr. Mallucci,

Thank you for submitting your revised manuscript to The EMBO journal. Your study has now been seen by the three referees and their comments are provided below.

As you can see, the referees appreciate the introduced changes and support publication here. Referee #3 brings up the related MS that was recently published in EMM and I have discussed that further with the referees. Overall, the referees find that there is enough of a distinction between the two studies with the focus on the mechanism in the current submission.

I note that the related study was cited in the initial submission and in the revised version as well. However, I would like to ask you to discuss the related MS a bit better also so that the reader gets a better feel for how the two studies are distinct.

When you submit the revised version will you also take care of the following editorial points:

- Please add the funding information in the online submission system as well (Alzheimer's Society and Alzheimer's Research UK)

- We need 3-5 keywords

- Re-label COI to Disclosure Statement and Competing Interests

- Please remove the Authors Contributions from the manuscript. The 'Author Contributions' section is replaced by the CRediT contributor roles taxonomy to specify the contributions of each author in the journal submission system. Please use the free text box in the 'author information' section of the manuscript submisssion system to provide more detailed descriptions (e.g., 'X provided intracellular Ca++ measurements in fig Y')

- APPENDIX tables should be provided in one PDF (Appendix) with a ToC and page numbers.

- Please ensure that the deposited dataset(s) is/are open and accessible (Data Availability Section)

- Our publisher has made some stylistic comments regarding figure legends - please see "Data Edited Manuscript file" and carry the suggestions over to the final MS.

- source data for EV figures should all be zipped up into one file

Please upload a point-by-point response as well

With best wishes

Karin

Karin Dumstrei, PhD
Senior Editor
The EMBO Journal

Instructions for preparing your revised manuscript:

Guide For Authors: https://www.embopress.org/page/journal/14602075/

authorguide

Referee #1:

The authors have adressed most of my concerns.

Although it is clear that PCE suppression is responsible for RBM3 induction upon cooling, and the PE is only suppressed by HNRNPH1 binding at 32 degrees, we still do not understand why the protein levels of RBM3 also increase upon HNRNPH1 KD at 37 degrees, where PE inclusion does not change. HNRNPH1 might have an additional function here. But this mechanism could be addressed in a follow up study.

Referee #2:

The authors have addressed all my comments.
I fully support the publication of this very nice story!

Referee #3:

I thank the authors for the careful revision of their manuscript, and their thoughtful answer to the reviewers' comments. All the points I raised have been addressed satisfactorily.

I have to point out that some of my (and other reviewers') questions were answered by the authors pointing to data published, very recently, in another article by a different lab -though the present corresponding author is also an author on the published paper (Preußner et al., EMBO mol med 2023). There is substantial conceptual overlap between the two studies. The editors should evaluate whether the findings in this manuscript still present enough novelty for publication in the EMBO Journal.

Referee #1

The authors have addressed most of my concerns.

Although it is clear that PCE suppression is responsible for RBM3 induction upon cooling, and the PE is only suppressed by HNRNPH1 binding at 32 degrees, we still do not understand why the protein levels of RBM3 also increase upon HNRNPH1 KD at 37 degrees, where PE inclusion does not change. HNRNPH1 might have an additional function here. But this mechanism could be addressed in a follow up study.

We agree with the reviewer that a follow-up study is needed to identify the exact role of HNRNPH1 as a regulator of RBM3 expression, which is our next plan. One possibility is that other HNRNPH1 targets may also influence RBM3 protein abundance.

Referee #2

The authors have addressed all my comments.
I fully support the publication of this very nice story!

We thank the reviewer for his/her full support.

Referee #3

I thank the authors for the careful revision of their manuscript, and their thoughtful answer to the reviewers' comments. All the points I raised have been addressed satisfactorily.

I have to point out that some of my (and other reviewers') questions were answered by the authors pointing to data published, very recently, in another article by a different lab -though the present corresponding author is also an author on the published paper (Preußner et al., EMBO mol med 2023). There is substantial conceptual overlap between the two studies. The editors should evaluate whether the findings in this manuscript still present enough novelty for publication in the EMBO Journal.

The therapeutic induction of RBM3 and the mechanisms of its induction have been the focus of the Mallucci laboratory for several years, following our discovery of the neuroprotective effects of RBM3 (Peretti *et al*, 2015).

This study focuses on the mechanisms underlying temperature-dependent RBM3 expression. In addition to the cis-regulatory element (the poison exon), we also show RBM3 expression is modulated by trans-acting factors, **including HNRNPH1** and several other splicing factors (first paragraph of the Discussion, Page 10). In it, we further investigate the direct interaction between HNRNPH1 and its binding sites at the RBM3 poison exon and show this interaction is strengthened at lower temperatures. **These are mechanistic insights unique to this study and provide the basis for alternative genetic tool designs for therapeutic modulation of RBM3 expression** (last paragraph of the Discussion, Page 12). Therefore,

this manuscript presents specific new findings valuable to the understanding of temperature-related RNA processing and opens up further therapeutic opportunities.

In the related paper, Preußner et al., 2023, which we cited and discussed in this paper, we collaborated with the Heyd laboratory (who have expertise in body temperature-controlled alternative splicing) to exploit alternative splicing of the RBM3 poison exon therapeutically. Preußner et al., describes the design and characterisation of RBM3-targeted antisense oligonucleotides (ASO) to exclude the poison exon and enhance RBM3 expression for neuroprotection in a mouse model of neurodegenerative disease. The two studies are complementary but distinct, and the ASO paper provides compelling translational support for pursuit of the mechanistic findings in this study, including how RBM3 PE retention is regulated by its interacting RBPs.

2nd Revision - Editorial Decision 11th May 2023

Dear Giovanna,

Thank you for submitting your revised manuscript to The EMBO Journal. I have now had a chance to take a careful look at it and all looks good.

I am therefore very pleased to accept the MS for publication here.

Congratulations on a nice study!

With best wishes

Karin

Karin Dumstrei, PhD
Senior Editor
The EMBO Journal

Please note that it is EMBO Journal policy for the transcript of the editorial process (containing referee reports and your response letter) to be published as an online supplement to each paper. If you do NOT want this, you will need to inform the Editorial Office via email immediately. More information is available here: https://www.embopress.org/page/journal/14602075/authorguide#transparentprocess

Your manuscript will be processed for publication in the journal by EMBO Press. Manuscripts in the PDF and electronic editions of The EMBO Journal will be copy edited, and you will be provided with page proofs prior to publication. Please note that supplementary information is not included in the proofs.

You will be contacted by Wiley Author Services to complete licensing and payment information. The required 'Page Charges Authorization Form' is available here: https://www.embopress.org/pb-assets/embo-site/tej_apc.pdf - please download and complete the form and return to embopressproduction@wiley.com

EMBO Press participates in many Publish and Read agreements that allow authors to publish Open Access with reduced/no publication charges. Check your eligibility: https://authorservices.wiley.com/author-resources/Journal-Authors/open-access/affiliation-policies-payments/index.html

Should you be planning a Press Release on your article, please get in contact with embojournal@wiley.com as early as possible, in order to coordinate publication and release dates.

If you have any questions, please do not hesitate to call or email the Editorial Office. Thank you for your contribution to The EMBO Journal.

EMBO Press Author Checklist

Corresponding Author Name: Giovanna Mallucci, Marc-David Ruepp, Julie Qiaojin Lin
Journal Submitted to: The EMBO Journal
Manuscript Number: EMBOJ-2022-113168

USEFUL LINKS FOR COMPLETING THIS FORM
The EMBO Journal - Author Guidelines
EMBO Reports - Author Guidelines
Molecular Systems Biology - Author Guidelines
EMBO Molecular Medicine - Author Guidelines

Reporting Checklist for Life Science Articles (updated January
This checklist is adapted from Materials Design Analysis Reporting (MDAR) Checklist for Authors. MDAR establishes a minimum set of requirements in transparent reporting in the life sciences (see Statement of Task: 10.31222/osf.io/9sm4x). Please follow the journal's guidelines in preparing your manuscript.
Please note that a copy of this checklist will be published alongside your article.

Abridged guidelines for figures
1. Data
The data shown in figures should satisfy the following conditions:
- the data were obtained and processed according to the field's best practice and are presented to reflect the results of the experiments in an accurate and unbiased manner.
- ideally, figure panels should include only measurements that are directly comparable to each other and obtained with the same assay.
- plots include clearly labeled error bars for independent experiments and sample sizes. Unless justified, error bars should not be shown for technical
- if n<5, the individual data points from each experiment should be plotted. Any statistical test employed should be justified.
- Source Data should be included to report the data underlying figures according to the guidelines set out in the authorship guidelines on Data

2. Captions
Each figure caption should contain the following information, for each panel where they are relevant:
- a specification of the experimental system investigated (eg cell line, species name).
- the assay(s) and method(s) used to carry out the reported observations and measurements.
- an explicit mention of the biological and chemical entity(ies) that are being measured.
- an explicit mention of the biological and chemical entity(ies) that are altered/varied/perturbed in a controlled manner.
- the exact sample size (n) for each experimental group/condition, given as a number, not a range;
- a description of the sample collection allowing the reader to understand whether the samples represent technical or biological replicates (including how many animals, litters, cultures, etc.).
- a statement of how many times the experiment shown was independently replicated in the laboratory.
- definitions of statistical methods and measures:
  - common tests, such as t-test (please specify whether paired vs. unpaired), simple χ2 tests, Wilcoxon and Mann-Whitney tests, can be unambiguously identified by name only, but more complex techniques should be described in the methods section;
  - are tests one-sided or two-sided?
  - are there adjustments for multiple comparisons?
  - exact statistical test results, e.g., P values = x but not P values < x;
  - definition of 'center values' as median or average;
  - definition of error bars as s.d. or s.e.m.

Please complete ALL of the questions below.
Select "Not Applicable" only when the requested information is not relevant for your study.

Materials

Newly Created Materials	Information included in the manuscript?	In which section is the information available? (Reagents and Tools Table, Materials and Methods, Figures, Data Availability Section)
New materials and reagents need to be available; do any restrictions apply?	Yes	Reagents and tools table (Appendix Table S7)

Antibodies	Information included in the manuscript?	In which section is the information available? (Reagents and Tools Table, Materials and Methods, Figures, Data Availability Section)
For **antibodies** provide the following information:<br>- Commercial antibodies: RRID (if possible) or supplier name, catalogue number and or/clone number<br>- Non-commercial: RRID or citation	Yes	Reagents and tools table (Appendix Table S7)

DNA and RNA sequences	Information included in the manuscript?	In which section is the information available? (Reagents and Tools Table, Materials and Methods, Figures, Data Availability Section)
**Short novel DNA or RNA including primers, probes**: provide the sequences.	Yes	Reagents and tools table (Appendix Table S1)

Cell materials	Information included in the manuscript?	In which section is the information available? (Reagents and Tools Table, Materials and Methods, Figures, Data Availability Section)
**Cell lines:** Provide species information, strain. Provide accession number in repository **OR** supplier name, catalog number, clone number, and/**OR** RRID.	Yes	Reagents and tools table (Appendix Table S7)
**Primary cultures:** Provide species, strain, sex of origin, genetic modification status.	Yes	Reagents and tools table (Appendix Table S7)
Report if the cell lines were recently **authenticated** (e.g., by STR profiling) and tested for mycoplasma contamination.	Not Applicable	

Experimental animals	Information included in the manuscript?	In which section is the information available? (Reagents and Tools Table, Materials and Methods, Figures, Data Availability Section)
**Laboratory animals or Model organisms:** Provide species, strain, sex, age, genetic modification status. Provide accession number in repository **OR** supplier name, catalog number, clone number, **OR** RRID.	Not Applicable	
**Animal observed in or captured from the field:** Provide species, sex, and age where possible**.**	Not Applicable	
Please detail **housing and husbandry conditions**.	Not Applicable	

Plants and microbes	Information included in the manuscript?	In which section is the information available? (Reagents and Tools Table, Materials and Methods, Figures, Data Availability Section)
**Plants:** provide species and strain, ecotype and cultivar where relevant, unique accession number if available, and source (including location for collected wild specimens).	Not Applicable	
**Microbes:** provide species and strain, unique accession number if available, and source.	Not Applicable	

Human research participants	Information included in the manuscript?	In which section is the information available? (Reagents and Tools Table, Materials and Methods, Figures, Data Availability Section)
If collected and within the bounds of privacy constraints report on age, sex and gender or ethnicity for all study participants.	Not Applicable	

Core facilities	Information included in the manuscript?	In which section is the information available? (Reagents and Tools Table, Materials and Methods, Figures, Data Availability Section)
If your work benefited from core facilities, was their service mentioned in the acknowledgments section?	Yes	Acknowledgements

Design

Study protocol	Information included in the manuscript?	In which section is the information available? (Reagents and Tools Table, Materials and Methods, Figures, Data Availability Section)
If study protocol has been **pre-registered, provide DOI in the manuscript**. For clinical trials, provide the trial registration number **OR** cite DOI.	Not Applicable	
Report the **clinical trial registration number** (at ClinicalTrials.gov or equivalent), where applicable.	Not Applicable	

Laboratory protocol	Information included in the manuscript?	In which section is the information available? (Reagents and Tools Table, Materials and Methods, Figures, Data Availability Section)
Provide DOI OR other citation details if **external detailed step-by-step protocols** are available.	Not Applicable	All protocols available in Materials and Methods

Experimental study design and statistics	Information included in the manuscript?	In which section is the information available? (Reagents and Tools Table, Materials and Methods, Figures, Data Availability Section)
Include a statement about **sample size** estimate even if no statistical methods were used.	Yes	Figure legends and Materials and Methods section
Were any steps taken to minimize the effects of subjective bias when allocating animals/samples to treatment (e.g. **randomization procedure**)? If yes, have they been described?	Not Applicable	
Include a statement about **blinding** even if no blinding was done.	Not Applicable	
Describe **inclusion/exclusion criteria** if samples or animals were excluded from the analysis. Were the criteria pre-established?<br><br>If sample or data points were omitted from analysis, report if this was due to attrition or intentional exclusion and provide justification.	Not Applicable	
For every figure, are **statistical tests** justified as appropriate? Do the data meet the assumptions of the tests (e.g., normal distribution)? Describe any methods used to assess it. Is there an estimate of variation within each group of data? Is the variance similar between the groups that are being statistically compared?	Yes	Figure legends and Materials and Methods section

Sample definition and in-laboratory replication	Information included in the manuscript?	In which section is the information available? (Reagents and Tools Table, Materials and Methods, Figures, Data Availability Section)
In the figure legends: state number of times the experiment was **replicated** in laboratory.	Yes	Figure legends
In the figure legends: define whether data describe **technical or biological replicates**.	Yes	Figure legends

Ethics

Ethics	Information included in the manuscript?	In which section is the information available? (Reagents and Tools Table, Materials and Methods, Figures, Data Availability Section)
Studies involving **human participants**: State details of **authority granting ethics approval** (IRB or equivalent committee(s), provide reference number for approval.	Not Applicable	
Studies involving **human participants**: Include a statement confirming that **informed consent** was obtained from all subjects and that the experiments conformed to the principles set out in the WMA Declaration of Helsinki and the Department of Health and Human Services Belmont Report.	Not Applicable	
Studies involving **human participants:** For publication of **patient photos**, include a statement confirming that consent to publish was obtained.	Not Applicable	
Studies involving experimental **animals**: State details of **authority granting ethics approval** (IRB or equivalent committee(s), provide reference number for approval. Include a statement of compliance with ethical regulations.	Not Applicable	
Studies involving **specimen and field samples:** State if relevant **permits** obtained, provide details of authority approving study; if none were required, explain why.	Yes	Materials and Methods include the orignial source of human iPSCs used in this study

Dual Use Research of Concern (DURC)	Information included in the manuscript?	In which section is the information available? (Reagents and Tools Table, Materials and Methods, Figures, Data Availability Section)
Could your study fall under dual use research restrictions? Please check biosecurity documents and list of **select agents and toxins** (CDC): https://www.selectagents.gov/sat/list.htm	Not Applicable	
If you used a select agent, is the security level of the lab appropriate and reported in the manuscript?	Not Applicable	
If a study is subject to dual use research of concern regulations, is the name of the **authority granting approval and reference number** for the regulatory approval provided in the manuscript?	Not Applicable	

Reporting

The MDAR framework recommends adoption of discipline-specific guidelines, established and endorsed through community initiatives. Journals have their own policy about requiring specific guidelines and recommendations to complement MDAR.

Adherence to community standards	Information included in the manuscript?	In which section is the information available? (Reagents and Tools Table, Materials and Methods, Figures, Data Availability Section)
State if relevant guidelines or checklists (e.g., **ICMJE, MIBBI, ARRIVE, PRISMA**) have been followed or provided.	Not Applicable	
For **tumor marker prognostic studies,** we recommend that you follow the **REMARK** reporting guidelines (see link list at top right). See author guidelines, under 'Reporting Guidelines'. Please confirm you have followed these guidelines.	Not Applicable	
For **phase II and III randomized controlled trials**, please refer to the **CONSORT** flow diagram (see link list at top right) and submit the CONSORT checklist (see link list at top right) with your submission. See author guidelines, under 'Reporting Guidelines'. Please confirm you have submitted this list.	Not Applicable	

Data Availability

Data availability	Information included in the manuscript?	In which section is the information available? (Reagents and Tools Table, Materials and Methods, Figures, Data Availability Section)
Have **primary datasets** been deposited according to the journal's guidelines (see 'Data Deposition' section) and the respective accession numbers provided in the Data Availability Section?	Yes	Data Availability section
Were **human clinical and genomic datasets** deposited in a public access-controlled repository in accordance to ethical obligations to the patients and to the applicable consent agreement?	Not Applicable	
Are **computational models** that are central and integral to a study available without restrictions in a machine-readable form? Were the relevant accession numbers or links provided?	Not Applicable	
If publicly available data were reused, provide the respective **data citations in the reference list**.	Yes	Materials and Methods and references
